# Synthetic biology based construction of biological activity-related library of fungal decalin-containing diterpenoid pyrones

**DOI:** 10.1038/s41467-020-15664-4

**Published:** 2020-04-14

**Authors:** Kento Tsukada, Shono Shinki, Akiho Kaneko, Kazuma Murakami, Kazuhiro Irie, Masatoshi Murai, Hideto Miyoshi, Shingo Dan, Kumi Kawaji, Hironori Hayashi, Eiichi N. Kodama, Aki Hori, Emil Salim, Takayuki Kuraishi, Naoya Hirata, Yasunari Kanda, Teigo Asai

**Affiliations:** 1 Department of Life Sciences, Graduate School of Arts and Sciences, The University of Tokyo, Komaba, Meguro-ku, Tokyo, 153-8902 Japan; 2 Division of Food Science and Biotechnology, Graduate School of Agriculture, Kyoto University, Kyoto, 606-8502 Japan; 3 Division of Applied Life Sciences, Graduate School of Agriculture, Kyoto University, Kyoto, 606-8502 Japan; 4 Division of Molecular Pharmacology, Cancer Chemotherapy Center, Japanese Foundation for Cancer Research, 3-8-31 Ariake, Koto-ku, Tokyo, 135-8550 Japan; 5 Department of Infectious Diseases, International Research Institute of Disaster Science, Tohoku Medical Megabank Organization, Tohoku University, 2-1, Seiryocho, Aoba-ku, Sendai, 980-0875 Japan; 6 Department of Intelligent Network for Infection Control, Graduate School of Medicine, Tohoku University, 2-1, Seiryocho, Aoba-ku, Sendai, 980-0875 Japan; 7 Faculty of Pharmacy, Institute of Medical, Pharmaceutical and Health Sciences, Kanazawa University, Kanazawa Ishikawa, Japan; 8 Division of Pharmacology, National Institute of Health Sciences, Tonomachi, Kawasaki-ku, Kawasaki, Kanagawa 210-9501 Japan; 9 Graduate School of Pharmaceutical Sciences, Tohoku University, Aoba-yama, Aoba-ku, Sendai, 980-8578 Japan

**Keywords:** Genetic engineering, Metabolic engineering, Combinatorial libraries, Natural product synthesis

## Abstract

A synthetic biology method based on heterologous biosynthesis coupled with genome mining is a promising approach for increasing the opportunities to rationally access natural product with novel structures and biological activities through total biosynthesis and combinatorial biosynthesis. Here, we demonstrate the advantage of the synthetic biology method to explore biological activity-related chemical space through the comprehensive heterologous biosynthesis of fungal decalin-containing diterpenoid pyrones (DDPs). Genome mining reveals putative DDP biosynthetic gene clusters distributed in five fungal genera. In addition, we design extended DDP pathways by combinatorial biosynthesis. In total, ten DDP pathways, including five native pathways, four extended pathways and one shunt pathway, are heterologously reconstituted in a genetically tractable heterologous host, *Aspergillus oryzae*, resulting in the production of 22 DDPs, including 15 new analogues. We also demonstrate the advantage of expanding the diversity of DDPs to probe various bioactive molecules through a wide range of biological evaluations.

## Abstract (web summary)

Combining genome mining and heterologous expression in a genetically tractable host can lead to bioactive natural products discovery and production. Here, the authors employ this strategy for new decalin-containing diterpenoid pyrenes production by expressing native, extended, and shunt pathways in *Aspergillus oryzae*.

## Introduction

Natural products are historically unparalleled successful sources for drug discovery^1,2^. During secondary metabolite biosynthesis in living organisms, natural products are formed in vivo through multi-step enzymatic reactions^3,4^. In other words, natural product structures are constructed and tailored in repeated interaction with proteins in cells. This biosynthetic manner provides natural product with drug-advantageous properties such as potential protein interactivity, water solubility, and membrane permeability^5^. For this reason, the natural product chemical space is highly relevant to biological space^6^. Therefore, the discovery of natural products with novel structures and biological activities is an important challenge in the pharmaceutical field. Natural products with valuable biological activities are recognized to contain biologically relevant privileged structures,^7,8^ whose analogues and congeners serve as excellent guides to discover not only more potent bioactive compounds but also various additional biological activities^9,10^. Thus, expanding the chemical space around biologically important natural products may accelerate drug discovery research^11^. Chemical synthesis, including divergent and diverted total syntheses, is a powerful strategy to produce bioactive natural product analogues and congeners^12,13^. A late-stage modification strategy is also a beneficial tool to generate natural product derivatives, such as diversity-oriented synthesis using natural products and their intermediate starting points^14–17^. However, the structural complexity and limited availability of natural products remain obstacles to synthesizing a large collection of natural products and their structural analogues in sufficient amounts. Thus, the development of a method that enables rapid and rational access to the chemical space around bioactive natural products and a reliable supply is required for drug discovery.

**Fig. 1:**
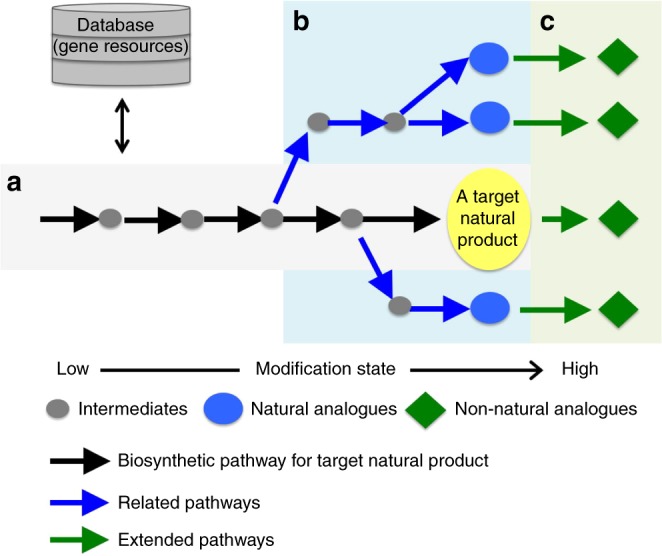
Strategy for expanding the chemical space of a target bioactive natural product via a synthetic biology approach. **a** Genome mining and reconstruction of the target natural product biosynthetic pathway. **b** Genome mining and reconstruction of the related pathways to produce natural analogues. **c** Pathway extension for combinatorial biosynthesis by adding additional enzymes to the native pathways to produce non-natural analogues (additionally modified analogues), Grey circles, blue circles and green diamonds show intermediates, natural analogues and non-natural analogues, respectively. Black, blue and green arrows show biosynthetic pathway for target natural product, its related pathways and extended pathways, respectively.

Fungi are among the most important microbial resources for drug discovery because of their ability to produce structurally diverse and biologically important natural products^18^. It is also well known that fungi possess extraordinary biosynthetic gene clusters that may encode highly diverse natural product biosynthetic pathways, and biosynthetic gene information has been accumulating at a rapidly accelerating rate in the past a decade because of genome sequencing innovation^19^. However, most biosynthetic pathways distributed in fungal genomes have not been linked with structural information^20^. This implies the presence of a large number of novel natural products that may be produced via those untapped pathways. A synthetic biology method based on heterologous biosynthesis coupled with genome mining is a promising approach to translate enormous amounts of biosynthetic gene information to richly diverse natural products. Genome mining based on biosynthetic studies has enabled rapid access to biosynthetic pathways not only for a target natural product and its related analogues but also for unexplored natural products. Heterologous biosynthesis has enabled to access compounds encoded by biosynthetic pathways found by genome mining and a number of total biosynthesis of natural products and discovering novel natural products have been reported^21–28^. In addition, combinatorial biosynthesis approach is powerful tool to generate non-natural analogues, which may be a great advantage to construct pharmaceutical beneficial screening library^29–32^. Thus, we apply the synthetic biology approach for rationally expanding the chemical space around a target natural product as follows (Fig. 1). The genome mining and reconstruction of a biosynthetic pathway for a target natural product in a heterologous host achieves its total biosynthesis, and the biosynthetic information allows us to mine its related biosynthetic pathways and access its analogues. We, then, apply pathway extension for combinatorial biosynthesis that can produce new natural product analogues that are not programmed in nature and more highly modified than programmed natural products.

**Fig. 2:**
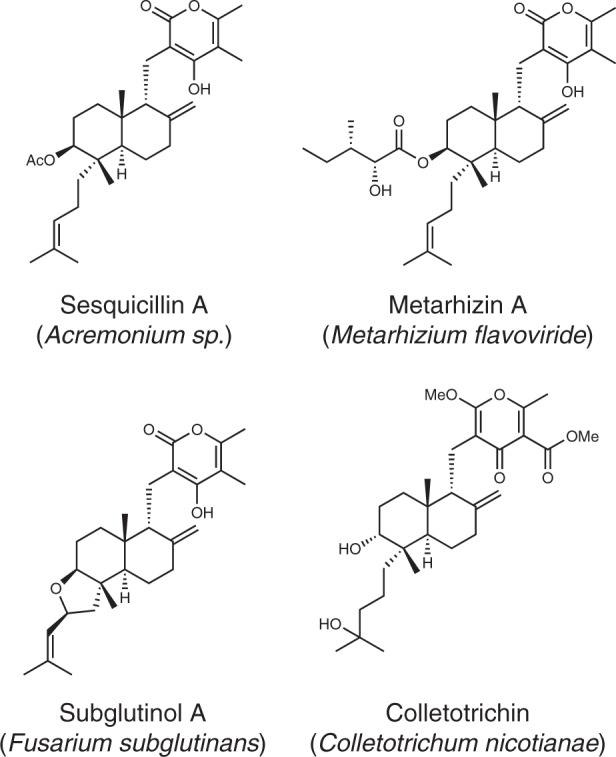
Examples of fungal decalin-containing diterpenoid pyrones (DDPs). DDPs have two privileged structures, pyrone (PKS) and decalin (terpenoid), and show a wide range of biological activities.

Fungi produce decalin-containing diterpenoid pyrones (DDPs), a type of meroterpenoid natural products composed of a diterpenoid-derived decalin ring system linked to pyrone biosynthesized via polyketide pathways^3^. DDPs are found only in fungi, and over 20 DDPs have been reported to date (Supplementary Fig. 1). Interestingly, even small structural differences in each DDP show a wide range of biological activities, such as antiproliferative activity against cancer cell lines and immunosuppressive activity, implying that DDPs contain a privileged structure (Fig. 2)^33–41^. Thus, the chemical space of DDPs is likely relevant to a diverse biological space and expanding this space leads to the development of a valuable screening library for drug discovery. In addition to promising biological activities, the structural features of DDPs render them exciting targets for total synthesis^42–44^. Recently, excellent divergent total synthesis has been reported, which enables access to four DDPs via a common intermediate in less than twenty chemical reaction steps^45^. Nevertheless, the synthesis of diverse DDPs as well as non-natural analogues is a rather elaborate effort; and therefore, it is difficult to prepare a large collection of DDPs and expand their chemical space by chemical means.

**Fig. 3:**
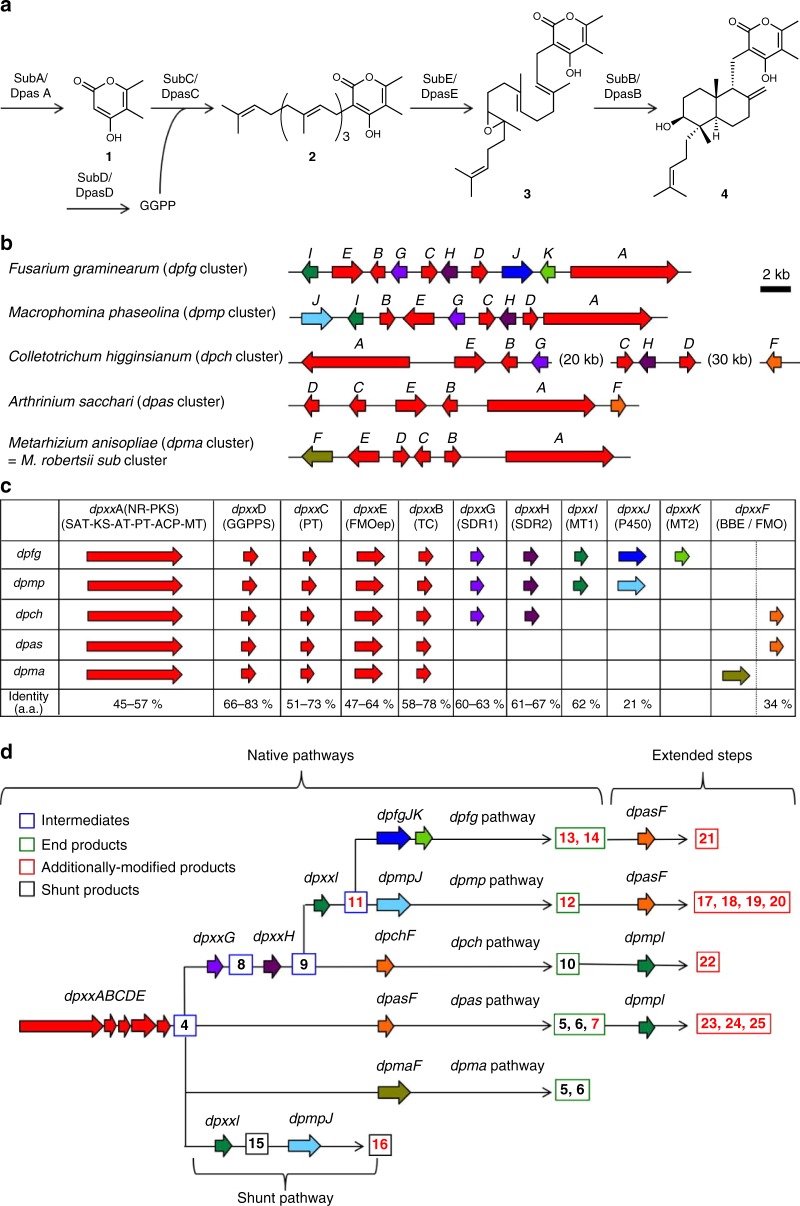
Genome mining and design of DDP pathways. **a** Biosynthetic pathway for common intermediate **4**. **b** DDP biosynthetic gene clusters distributed in five fungal genera. **c** Comparative analysis of each biosynthetic gene cluster. **d** Design of native pathways, extended steps and one shunt pathway heterologously reconstituted in this study and summary of products (red numbers show compounds that have not been reported.) produced through the DDP pathways (blue square, green square, red square and black square show intermediates, end products, additionally modified products and shunt products, respectively).

Here, we demonstrate the advantage of the synthetic biology approach based on heterologous and combinatorial biosynthesis coupled with genome mining for constructing a biologically relevant DDP-focused library composed of a variety of DDPs. Although biosynthetic studies on DDPs are limited, a putative biosynthetic gene cluster and pathway for subglutinols (*subA*–*F*) in an entomopathogenic fungus, *Metarhizium robertsii*, has been estimated (Fig. 3a). In addition, the functions of a non-reducing polyketide synthase (NR-PKS) SubA, geranylgeranyl diphosphate synthase (GGPPS) SubD and prenyltransferase (PT) SubC have been identified by a heterologous expression study^46^. We perform genome mining based on this information and find putative DDP gene clusters distributed in five fungal genera *Arthrinium*, *Metarhizium*, *Colletotrichum*, *Macrophomina* and *Fusarium* fungi (Fig. 3b). According to bioinformatics analyses, we design five native pathways from those biosynthetic gene clusters and reconstruct them in *Aspergillus oryzae* NSAR1^22–27,47^, an excellent heterologous host for the production of fungal natural products, to give intermediates and end products encoded in all the pathways. Subsequently, we conduct pathway extension for combinatorial biosynthesis by adding additional modification enzymes to the native DDP pathways, yielding unnatural DDP analogues. Overall, we successfully produce 22 DDPs including 15 analogues that have not been reported, which include intermediates, end products and additionally modified analogues. Because they all can be easily re-supplied by cultivation of the corresponding transformant, we are able to evaluate a variety of biological activities of the DDP-focused library and find wide range of potent bioactivities, such as cell cytotoxicity against cancer cell lines through mitochondrial complex III inhibition, antiproliferative activity against cancer stem-like cells, anti-HIV, preventing amyloid β(Aβ) aggregation in nucleation phase, paralysing activity against adult *Drosophila* and suppressing insect innate immune signal transduction. Most these biological activities firstly have been found in DDPs in this study.

## Results

### Genome mining and design of DDP biosynthetic pathways

To find biosynthetic gene clusters that may encode DDP pathways, we performed genome mining of the public databases and our original gene resources by using SubA (NR-PKS) as a query. As a result, five candidate gene clusters with *subA*–*E* orthologous genes that may encode NR-PKS, GGPPS, PT, flavin adenine dinucleotide (FAD)-dependent epoxidase (FMOep) and terpene cyclase (TC) were found in five fungal genomes, *Fusarium graminearum* PH-1 (*dpfgABCDEGHIJK*), *Macrophomina phaseolina* MS6 (*dpmpABCDEGHIJ*) *Colletotrichum higginsianum* IMI349063 (*dpchABCDEFGH*), *Metarhizium anisopliae* E6 (*dpmaABCDEF*), and *Arthrinium sacchari* (*dpasABCDEF*), which we previously isolated from a spider (Fig. 3b and Supplementary Table 1). The *dpma* gene cluster, which is identical to the *sub* gene cluster in *M. robertsii*, is widely conserved across the genus *Metarhizium*, and the *dpfg* gene cluster is also broadly distributed in the genus *Fusarium* (Supplementary Fig. 2). To design native DDP biosynthetic pathways distributed in the five fungal genera, the five gene clusters were comparatively analysed based on amino acid sequence homology and reordered them as shown in Fig. 3c (Supplementary Tables 2 and 3). The five genes (*dpxxABCDE*) are highly conserved in each cluster, suggesting that all the pathways share the biosynthetic pathway for a common intermediate **4**. The differences in the genes at the tailoring steps in each pathway may diversify DDP biosynthesis. The three biosynthetic gene clusters in *F. graminearum* (*dpfg* cluster), *M. phaseolina* (*dpmp* cluster) and *C. higginsianum* (*dpch* cluster) contain two types of short chain dehydrogenase reductase (SDR) genes, *dpxxG* (SDR1) and *dpxxH* (SDR2). Each of the *dpxxG* and *dpxxH* genes can be recognized as orthologous because of their high similarity (their encoded enzymes are approximately 60% identical each other); that is, each set of SDRs, *dpfgGH*, *dpmpGH* and *dpchGH*, may provide the same product from **4**. Both the *dpfg* and *dpmp* clusters include an orthologous methyltransferase gene, *dpxxI* (MT1), which it is absent in the *dpch* cluster, indicating that the *dpch* pathway is probably divided from the *dpfg* and *dpmp* pathways after SDR modification steps and that a remaining *dpchF* (FMO) would lead to the end product in the *dpch* pathway. Both the *dpmp* and *dpfg* clusters contain a P450 gene, *dpmpJ* and *dpfgJ*, respectively, but they show low similarity to each other (their encoded enzymes are ~20% identical to each other), suggesting that the *dpfg* and *dpmp* pathways may have branched after the MT1 modification step. Subsequently, *dpmpJ* and *dpfgJK* afford the final products in each pathway. However, the *dpas* and *dpma* gene clusters possess only a modifying enzyme, FMO *dpasF* and FAD-dependent BBE domain-containing oxidoreductase (BBE) *dpmaF*, respectively. Therefore, both pathways are likely branched at the initial tailoring stage, leading to the final products. As a result, we predicted the treelike native DDP pathways, as depicted in Fig. 3d. Considering previously reported DDPs and their producing fungi, the *dpch* and *dpma* pathways may produce higginsianin A^40^ and subglutinol A^38,46^, respectively. However, the orphan *dpfg*, *dpmp* and *dpas* pathways may provide new DDP analogues.

### Reconstitution of DDP pathways

We reconstituted all the pathways by stepwise introduction of the biosynthetic genes in a heterologous host *A. oryzae* NSAR1 according to the hypothetical biosynthetic pathways (Fig. 3d and Supplementary Fig. 5). Because it is difficult to obtain genome-sequenced strains of *F. graminearum* PH-1, *M. phaseolina* MS6, *C. higginsianum* IMI349063 and *M. anisopliae* E6, we used *F. graminearum* 50218, *M. phaseolina* NBRC7317, *C. higginsianum* MAFF305635 and *M. anisopliae* NBRC103233 instead as gene donors.

Initially, we introduced *dpasACD* into *A. oryzae* to construct the *A. oryzae* transformant with *dpasACD* (*AO-dpasACD*), which, as expected, produced prenylated (C_20_) α-pyrone **2** (Fig. 3a and Supplementary Fig. 7). We subsequently introduced *dpasBE* into *AO-dpasACD* to construct *AO*-*dpasABCDE*, which provided the common intermediate **4** (87 mg L^−1^) (Supplementary Fig. 7), demonstrating its biosynthetic machinery for the first time. We also characterized *dpfgBE*, *dpmpBE* or *dpchBE* as sharing the same function of *dpasBE* by introducing them into *AO-dpasACD* (Supplementary Fig. 7).

**Fig. 4:**
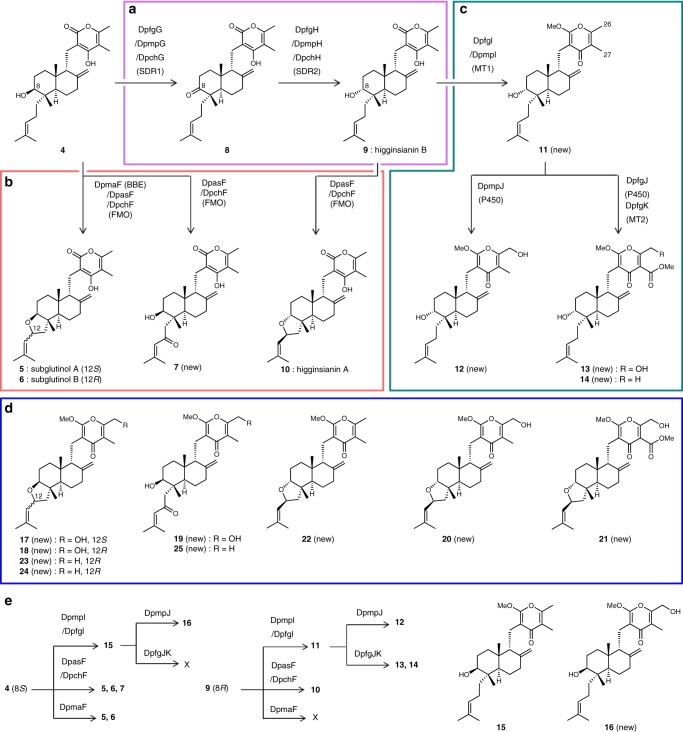
Summary of enzymatic conversions in the DDP pathways. **a** C-8 stereochemistry inversion steps. **b** Modifications at the C_5_ unit. **c** Modifications on the pyrone rings. **d** Structures of additionally modified DDPs produced through the extended pathways. **e** Summary of substrate selectivities of the modification steps in the DDP pathways.

Next, we aimed to comprehensively reconstitute modification steps in native DDP pathways by using the **4**-producing transformants as a platform. We reconstituted the *dpas* and *dpma* pathways by introducing an FMO gene *dpasF* or a BBE gene *dpmaF* into *AO-dpasABCDE* to give *AO-dpasABCDEF* and *AO-dpasABCDE-dpmaF*. *AO-dpasABCDE-dpmaF* provided subglutinols A (**5**, 89 mg L^−1^) and B (**6**, 6 mg L^−1^) as previously suggested in the *sub* cluster^46^ (Supplementary Fig. 12). On the other hand, *AO-dpasABCDEF* produced a new DDP analogue with an enone system at the C_5_ unit (**7**, 6 mg L^−1^) as well as subglutinols A (**5**, 62 mg L^−1^) and B (**6**, 5 mg L^−1^) (Supplementary Figs. 12 and 13). These results suggested that both DpasF and DpmaF are involved in tetrahydrofuran (THF) ring formation at the C_5_ unit, while DpasF possesses an additional catalytic ability of multi-step oxidations to generate the enone at the C_5_ unit in **7** (Fig. 4b).

We then aimed to reconstitute the *dpch*, *dpmp* and *dpfg* pathways in *A. oryzae*. The introduction of *dpmpG* or *dpmpGH* into *AO-dpasABCDE* afforded two transformants, *AO-dpasABCDE-dpmpG* and *AO-dpasABCDE-dpmpGH*. *AO-dpasABCDE-dpmpG* accumulated ketone **8** (23 mg L^−1^), while *AO-dpasABCDE-dpmpGH* gave higginsianin B (**9**, 28 mg L^−1^) (Supplementary Fig. 17). This result showed that SDR1, DpmpG, oxidized the 8*S* hydroxy group to a ketone and SDR2, DpmpH, reduced the ketone to the 8 *R* hydroxy group in a similar manner to that in andrastin biosynthesis^48^ (Fig. 4a). We also demonstrated that *dpfgGH* and *dpchGH* were involved in the same inversion of the stereochemistry at C-8 (Supplementary Fig. 17). The entire *dpch* pathway was heterologously reconstituted by introducing *dpchGHF* into a **4**-producing transformant, and the resulting transformant, as expected, produced higginsianin A (**10**, 28 mg L^−1^) (Fig. 4b and Supplementary Fig. 21). We reconstituted whole *dpmp* and *dpfg* pathways by using higginsianin B (**9**) producing transformants as a platform. The introduction of *dpmpI* and *dpfgI* into the platforms yielded *AO-dpasABCDE-dpmpGHI* and *AO-dpasABCDE-dpfgGHI*, both of which produced a new intermediate **11** (29 mg L^−1^ and 20 mg L^−1^, respectively). Moreover, *dpmpIJ* and *dpfgIJK* were introduced to construct *AO-dpasABCDE-dpmpGHIJ* and *AO-dpasABCDE-dpfgGHIJK*. The transformant expressing the whole *dpmp* gene cluster, *AO-dpasABCDE-dpmpGHIJ*, afforded a new DDP with a C-26 primary alcohol on the γ-pyrone moiety (**12**, 21 mg L^−1^) (Fig. 4c and Supplementary Fig. 25). On the other hand, the transformant expressing the whole *dpfg* pathway, *AO-dpasABCDE-dpfgGHIJK*, produced the new DDPs **13** (major product, 5 mg L^−1^) and **14** (minor product, 2 mg L^−1^) with a highly oxidized γ-pyrone moiety including a methyl ester (Fig. 4c and Supplementary Fig. 25), like colletotrichin obtained from *C. nicotianae*^41^. The results suggested that a P450, DpmpJ, oxidized C-26 methyl to primary alcohol, DpfgJ, catalysed a three-step oxidation at C-27 to generate a carboxylic acid as well as C-26 hydroxylation. The results also indicated that an MT1, DpfgI, is involved in the same methylation as DpmpI, while an MT2, DpfgK, methylates the carboxylic acid generated by DpfgJ. We thus completely reconstructed all the DPP native pathways distributed in five fungi, resulting in the production of 11 DDPs, including five new analogues. As expected, the *dpma* and *dpch* pathways encoded the biosynthetic pathways for subglutinols (**5**, **6**) and higginsianin A (**10**), respectively, and the orphan *dpas*, *dpmp* and *dpfg* pathways provided new DDP analogues.

We also investigated substrate selectivities of the modification enzymes using *A. oryzae* heterologous expression system. In summary, DpmaF and DpfgJ strictly recognized the C-8 configuration, while DpasF, DpchF, DpmpI, DpfgI and DpmpJ showed tolerant substrate selectivity, which became an advantage for the generating diversity in combinatorial biosynthesis. Through the experiments, we found and reconstituted a shunt DDP pathway in *A. oryzae*, which afforded viridoxin A hydrolysate **15**^33^ (42 mg L^−1^) and a new DDP analogue **16** (6 mg L^−1^) (Figs. 3d, 4e, Supplementary Figs. 29, 31 and Supplementary Note 15).

### Pathway extension for combinatorial biosynthesis

The reactions of each enzyme in all the pathways are summarized in Fig. 4a–c. The enzymes that catalysed C_5_ unit modifications were specifically distributed in the *dpma*, *dpas* and *dpch* pathways, while the enzymes involved in the pyrone moiety modifications were localized in the other pathways. However, no pathway that containing both C_5_ unit and pyrone moiety modification enzymes is encoded in native DDP biosynthetic gene clusters. Therefore, we aimed to generate unnatural DDPs with further modified structures than those of the end products in each DDP pathway and conducted combinatorial biosynthesis by combining C_5_ unit-modifying pathways with pyrone-decorating pathways (Fig. 3d). We chose *dpasF* among C_5_ unit-modifying enzymes as an additional modification enzyme, because DpasF (FMO) enables the generation of four types of C_5_ unit moieties through its multifunctional oxidative ability, and we designed two extended pathways by adding *dpasF* to the *dpmp* and *dpfg* pathways. In addition, MT1 (*dpmpI*) was connected to the *dpas* and *dpch* pathways. Thus, we designed and reconstituted the four extended pathways in *A. oryzae* (Fig. 3d). One transformant (*dpmp* pathway + *dpasF*), as expected, produced four new analogues, **17** (5 mg L^−1^), **18** (3 mg L^−1^), **19** (16 mg L^−1^), and **20** (5 mg L^−1^), due to the promiscuity of DpmpJ (Figs. 3d, 4d and Supplementary Fig. 35). Another transformant (*dpfg* pathway + *dpasF*) afforded only the new analogue **21** (6 mg L^−1^), the most modified compound in this study because DpfgJ (P450) strictly recognizes C-8 stereochemistry (Figs. 3d, 4d and Supplementary Fig. 36). In this transformant, another expected product originating from **14** could not be observed in HPLC analysis. The third transformant (*dpch* pathway + *dpmpI*), as expected, produced *O*-methylated higginsianin A (**22**, 17 mg L^−1^), and the fourth transformant (*dpas* pathway + *dpmpI*) provided **23** (23 mg L^−1^), **24** (7 mg L^−1^) and **25** (3 mg L^−1^) (Figs. 3d, 4d and Supplementary Fig. 40). As expected, all the non-natural analogues produced through combinatorial biosynthesis contained modifications on both the C_5_ unit and the pyrone moiety. Since the four re-designed extended pathways are not found in the genome databases, of course, every compound produced through the pathways had new structures.

Thus, we achieved the comprehensive production of fungal DDPs through the reconstitution of five-native pathways, one shunt pathway and four extended pathways in *A. oryzae* and produced 22 DDPs, including 15 new compounds. Among these 22 compounds, 11 compounds came from native pathways, 2 compounds were biosynthesized through shunt pathways, and 9 compounds were produced via extended pathways (Supplementary Fig. 43, Supplementary Table 19). The new compounds, **7**, **11**–**14** and **16**–**25** were named as FDDP A–O, respectively. All the compounds produced in this study were purified, and their structures were fully determined by spectral analyses. The absolute configuration of the common intermediate (**4**) was determined by the modified Mosher’s method^49^ (Supplementary Fig. 10), while those of **5**, **6**, **9** and **10** were identified by comparing their optical rotation with reported values^40,43^. The absolute configurations of the other DDPs were determined based on their biosynthetic relationships. The titres of all the DDPs based on HPLC analysis of all the transformants are summarized in Supplementary Table 19.

### Antiproliferative effects on cancer stem-like cells

Initially, we evaluated new DDP analogues for their antiproliferative activities across the panel of 39 human cancer cell lines, JFCR39^50,51^. The assay not only showed their cytotoxic effects but also provided the characteristic profiles similar to those of antimycin A and myxothiazol, known inhibitor of mitochondrial complex III (Supplementary Figs. 44, 45 and Supplementary Tables 21, 22). We then revealed that most DDPs, except for enone-containing compounds, selectively prevented mitochondrial complex III, and the effect was the same degree as that of antimycin A in vitro assay (Supplementary Table 20).

**Fig. 5:**
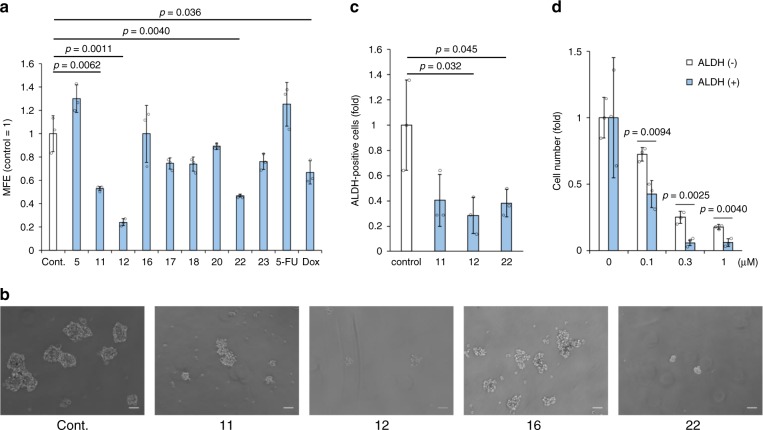
DDPs show anti-CSC activity in breast cancer cell line MCF-7. **a** Effect of DDPs on mammosphere formation. After the cells were cultured in the presence of 5 μM DDPs for 7 days, the number of mammospheres was microscopically counted and the percentage of mammosphere-forming cells was determined as mammosphere-forming efficiency (MFE; %). Data represent mean ± s.d. from three independent replicate experiments. *P*-values were calculated by a two-sided unpaired Student’s *t*-test, compared with control. **b** Micrographs show representative images of mammosphere formation from three independent replicate experiments using compound **11, 12, 16, 22**. The scale bar, 50 mm. **c** Effect of DDPs (1 μM, 3 days) on ALDH-positive cells in MCF-7 cells. Data represent mean ± s.d. from three independent replicate experiments. *P*-values were calculated by a two-sided unpaired Student’s *t*-test, compared with control. **d** Dose-dependent effect of compound **12** on ALDH-positive cells in MCF-7 cells. Data represent mean ± s.d. from three independent replicate experiments. *P*-values were calculated by a two-sided unpaired Student’s *t*-test. Source data underlying Fig. 5a, c, d are provided in a Source Data file.

We also evaluated antiproliferative activity against cancer stem cells (CSCs), which are a small sub-population in tumour bulk identified in most cancer cells and clinical samples^52^. CSCs have been a major problem in cancer therapy because they are responsible for recurrence, metastasis and drug resistance to chemotherapeutic agents that affect proliferative cells^53–56^. Anti-CSC activity was evaluated by the inhibition of sphere-forming ability, a well-studied method to enrich the CSC-like population, and selective cytotoxicity against one of the most reliable CSC markers, aldehyde dehydrogenase (ALDH)-positive cells^55^. We selected compounds **5**, **11**, **12**, **17**, **18**, **20**, **22** and **23** according to dose-dependent cytotoxicity against the bulk of MCF-7 cells for the mammosphere formation assay (Supplementary Fig. 46a). Low-toxicity **16** was also added as a negative control. Each compound was tested at 5 μM, and **11**, **12** and **22** clearly inhibited sphere formation (Fig. 5a, b) more effectively than the anticancer drugs 5-fluorouracil (5-FU) and doxorubicin. Interestingly, **12** showed the strongest effect on sphere formation, while its C-8 epimer **16** did not affect sphere formation, suggesting that the 8*R* configuration significantly contributed to this activity. We next evaluated the three DDPs that inhibited mammosphere formations against ALDH-positive cells, a functional marker for breast CSCs. MCF-7 cells were treated with 1 μM of **11**, **12** or **22**, and the ALDH-positive cell population was analysed by using a fluorescence activated cell sorter (Supplementary Fig. 46b). The results showed that **11**, **12** and **22** reduced the population of ALDH-positive cells (Fig. 5c). The most effective compound, **12**, was tested at lower concentrations and showed selective and potent cytotoxicity against ALDH-positive cells (Fig. 5d). Thus, for the first time, we successfully found DDPs with potent antiproliferative effects on the CSC-like population in MCF-7 cells, which may lead to new anti-CSC drugs. Interestingly, subtle structural differences in DDPs generated the difference in anti-CSC activity.

### Biological activity against *Drosophila*

We screened the DDPs on the *Drosophila* assay system for cytotoxicity, inhibitory activity of innate immune signalling and insecticidal activity^57^. The cytotoxicity of the DDPs was evaluated using two cell lines, embryonic macrophage-derived DL1 cells^58–60^ and larval blood cell-derived l(2)mbn cells^61^, and their IC_50_ values are listed in Supplementary Table 23. Most DDPs except for compounds with the enone at the C_5_ unit (**7**, **19** and **25**) showed potent antiproliferative activity, and they affected DL1 cell lines more potently. Notably, subglutinol A (**5**) and **21** strongly inhibited cell growth in the DL-1 cell line (IC_50_ = 12 nM (**5**) and 15 nM (**21**)).

**Fig. 6:**
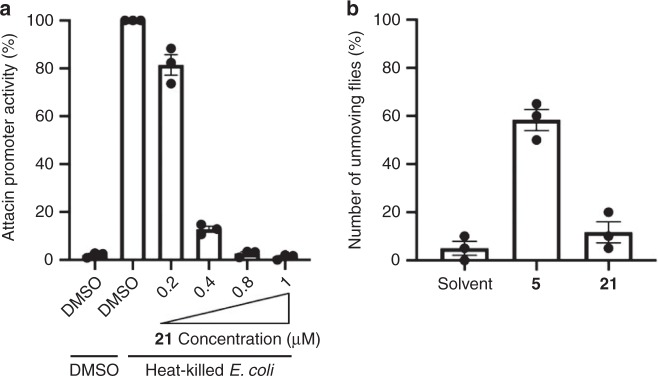
Biological activity against *Drosophila*. **a** Inhibitory effect of compound **21** on the IMD pathway *Drosophila* l(2)mbn cells were stimulated with heat-killed *E. coli* and the activity of the Attacin promoter, a read-out of the activation of the IMD pathway, was monitored by a luciferase assay. Data are means ± SEM of triplicate wells from a single experiment and are representative of two independent experiments. **b** Toxic effect of compound **5** on *Drosophila* adult. Adult flies (c.a. 1 mg each) were injected with the indicated compounds (3.5 ng each), and the flies that did not show movement or only faint shaking of their legs were counted as unmoving flies. Data are the means ± SEM of triplicate samples from a single experiment and are representative of two independent experiments. Source data underlying Fig. 6a, b are provided in a Source Data file.

We next tested the ability of the DDPs to affect the innate immune pathways, namely, the Toll and immune deficiency (IMD) pathways, which are the front line of defence against infection by microorganisms^57^. No compound showed a remarkable effect on the Toll pathway, while **21** inhibited the IMD pathway with an IC_50_ of 0.27 ± 0.01 µM (Fig. 6a). This result implies that the γ-pyrone moiety that is observed in **21** might have a role in the activity and that the THF ring at the C_5_ unit enhances the effect, since **13** tend to show a moderate inhibitory effect on the IMD pathway. Then, we evaluated the insecticidal activity of the most potent cytotoxic **5** and IMD pathway inhibitor **21** by using adult *Drosophila* (Fig. 6b). The result clearly indicates that **5** efficiently paralysed adult *Drosophila* 1 h after injection, implying that subglutinol A (**5**) is one of the virulence factors of entomopathogenic *Metarhizium* fungi against insects. Thus, we successfully found unique biological activity against *Drosophila* in **5** and **21**, which may potentially be developed as insecticides.

### Anti-HIV assay

We examined the inhibitory activities of DDPs against various species of bacteria (both gram-negative and gram-positive bacteria), fungi and viruses. No DDPs showed significant anti-bacterial or anti-fungal activities. In contrast, preliminary screening of the DDPs for anti-viral agents with hepatitis B virus (HBV), measles virus (MV), Epstein-Barr virus (EBV), herpes simplex virus (HSV) and human immunodeficiency virus-1 (HIV-1) indicated their selective inhibition of HIV. We further investigated the anti-HIV activity of the DDPs by the MAGI assay^62^ using AZT as positive control (EC_50_ = 29.7 nM and CC_50_ > 10000 nM). Some of compounds, such as **5**, **10**, **11**, **12**, **14**, **17**, **18**, **20**, **23** and **24**, exerted specific anti-HIV activity (EC_50_ < 200 nM) without affecting other viruses (Supplementary Table 24). Among them, **20** showed the strongest anti-HIV activity without apparent cytotoxicity (EC_50_ = 8.5 nM and CC_50_ > 10,000 nM). Other compounds also showed moderate to substantial activity but the effective concentrations were high; therefore, their toxicity made it difficult to clearly distinguish their anti-HIV effect.

### Screening of amyloid Aβ 42 aggregation inhibitors

The aggregation of the 42-mer-amyloid β (Aβ42) is involved in the pathogenesis of Alzheimer’s disease (AD)^63^. A nucleation-dependent polymerization model that is composed of nucleation and elongation phases is generally accepted as the aggregation mechanism of Aβ42^64^. In the nucleation phase, the monomer of Aβ42 gradually forms low-molecular-weight oligomers (called nuclei), which cause synaptotoxicity and memory loss^65^.

**Fig. 7:**
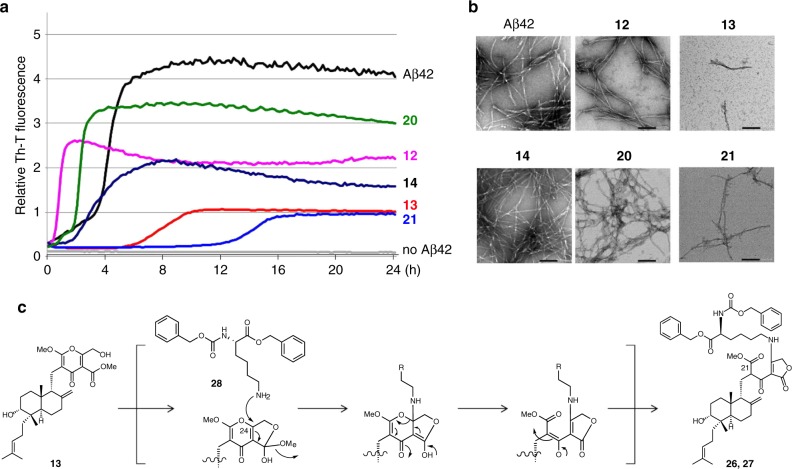
Inhibitory activity of Aβ aggregation. **a** Selected results of the Th-T assay (*n* = 3). **b** TEM analysis of typical amyloid fibrils formed by Aβ42. Scale bar =  100 nm. Representative micrographs are selected from at least six micrographs taken in one grid. **c** Hypothetical reaction mechanism of **13** and lysine derivative **28** (**13** was reacted with **28** (5.0 eq) in THF at r.t. for 10 h to give adducts **26** and **27**). Source data underlying Fig. 7a, b are provided in a Source Data file.

We evaluated the ability of DDPs to inhibit Aβ42 aggregation by using the Th-T (thioflavin-T) assay, which is a conventional and useful method for quantifying Aβ aggregates (Supplementary Fig. 47). Consideration of the preliminary results and SARs, we picked up **12**, **13**, **14**, **20**, and **21**, and tested them again (Fig. 7a). The fluorescence of Aβ42 and the Th-T complex began to increase after 4 h of incubation, and only **13** and **21** delayed the nucleation phase of Aβ42 until 6 and 13 h, respectively. In contrast, the congeners of **13** and **21** regarding the primary alcohol or methyl ester group, such as **12**, **14** and **20** showed no delay even though **12**, **14** and **20** partly suppressed the elongation of Aβ42. In TEM analysis, the formation of typical amyloid fibrils of Aβ42 was strongly prevented only by **13** and **21**, leading to the fragmentation of fibrils (Fig. 7b).

To further analyse the inhibitory mechanism, we subjected a mixture of Aβ42 and **21** after 1 h of incubation at room temperature to liquid chromatography/quadrupole time-of-flight mass spectrometry (LC/Q Tof-MS). The possible peaks of Aβ42–**21** adducts were detected, and then the mass envelopes at +7, +6, +5, and +4 charge distribution corresponded to the Michael adduct based on the loss of 32 Da (Supplementary Figs. 48b–d, 49). However, no such adduct was found in the presence of **20** as a negative control (Supplementary Fig. 48a). To identify the specific amino acids involved in the interaction of **21** with the Aβ monomer, we performed LC–MS/MS analysis with collision-induced dissociation (CID) using E22P, M35ox-Aβ9-35 as a toxic conformer surrogate^66,67^. Based on a large number of fragmented b ions and y ions, Lys16 in Aβ42 (DAEFRHDSGYEVHHQ**K**LVFFAEDVGSNKGAIIGLMVGGVVIA) seemed to be one of the target amino acid residues of **21** (Supplementary Fig. 50).

To investigate the reaction mechanism between the highly oxidized γ-pyrone observed in **13** and **21**, and the lysine residue in Aβ42 with a decrease of 32 Da, we carried out the following experiment. Benzyl-protected lysine **28** was reacted with **13** and **21** as well as the structurally related **14** and **20** in THF at room temperature (Supplementary Note 30), and each reaction was monitored by HPLC analysis at 1 h and 10 h after mixing (Supplementary Fig. 51a). In the reaction of **13**, two new peaks gradually appeared at 1 h later, and **13** completely converted to the new peaks **26** and **27** (Supplementary Fig. 51b) in 10 h. LCMS analysis showed the same molecular ion peak of **26** and **27** at *m*/*z* 863 [M + Na]^+^, which suggested that **13** connected to **28** with a decreasing of 32 Da in the same situation described above. The reaction of **21** proceeded in the same manner as that of **13**, whereas **14** and **20** did not afford any adducts under the same reaction conditions (Supplementary Fig. 51), suggesting that the γ-pyrone structure, including a hydroxyl group and carboxylic methyl ester, was necessary for the reaction. After scaling up the reaction, we isolated **26** and **27**, which are in the C-21 epimer relationship, and determined their structures (Fig. 7c, Supplementary Note 31). From their structures, we proposed the following reaction mechanism: **13** took the ortho ester form, which increased electrophilicity at C-24, and the amine in lysine performed a nucleophilic attack at C-24 with elimination of the methoxy group followed by electron transfer to form **26** and **27** (Fig. 7c and Supplementary Fig. 52). However, the pyrone moiety did not react with the primary thiol in the cysteine derivative, the hydroxyl group in the serine derivative or the guanidine group in the arginine derivative (Supplementary Fig. 53 and Supplementary Note 32). Thus, we successfully discovered that DDPs markedly inhibited Aβ aggregation in the nucleation phase via a lysine-selective binding motif.

## Discussion

Herein, we report the advantage of the synthetic biology approach based on heterologous biosynthesis coupled with genome mining for rationally expanding the chemical space of biologically active natural products. In this study, we focused on fungal DDPs as a natural product family including a privileged structure and aimed to produce a diverse set of DDPs. Genome mining revealed putative gene clusters for DDPs distributed in five fungal genera and bioinformatics analyses were performed to draw the five treelike DDP biosynthetic pathways. Stepwise reconstruction of all the pathways in *A. oryzae* allowed us to make transformants corresponding to all the intermediates and end products in the pathways, resulting in the isolation of all the DDPs produced in the native DDP pathways. From their structures, we determined the function of all the modification enzymes in the pathways. Subsequently, we designed four extended pathways by using the five native pathways as tool for combinatorial biosynthesis. This combinatorial biosynthesis enabled us to access non-natural analogues that equipped further modifications than those of compounds produced via native pathways. Finally, we achieved heterologous biosynthesis of 22 DDPs, including 15 new analogues, which were included intermediates, end products, shunt products and additionally modified analogues in *A. oryzae*.

We then screened the DDP-focused library with various biological activity assays. DDPs produced in this study shared same skeleton and showed similar antiproliferative activities against cancer cells and inhibition of mitochondrial complex III each other. However, interestingly, small structure differences in each DDP gave unique functions of markedly reducing CSC-like populations in MCF-7 bulk cells (**11**, **12** and **22**), potent inhibitory activity of an insect innate immune system, the IMD pathway (**21**), potent paralysing activity against adult *Drosophila* (**5**), selectively inhibited HIV proliferation (**20**), and Aβ aggregation in the nucleation phase through trapping lysine residue on highly modified γ-pyrone motif (**13** and **21**). Notably, compounds produced via extended pathways indeed showed unique biological activities. Thus, this study showcases the capability of combinatorial synthetic biology in acceleration of drug discovery.

In the post-genomic era, a synthetic biology approach is undoubtedly one of the most powerful methods to achieve not only the generation of natural products from gene resources but also the rational expansion of bioactive natural product chemical space. The gene resources available in the method are rapidly increasing; therefore, the method would infinitely expand the chemical diversity of natural products and their analogues. In addition, the method solves the supply issues and permits natural products to be subjected to enough biological evaluations. When the method becomes advanced and widely used, natural products will be easier available for drug discovery and definitely increase the opportunity to develop natural products as drug seeds^68^.

## Methods

### General methods

Polymerase chain reaction (PCR) was performed using a TaKaRa PCR Thermal Cycler Dice® Gradient (Takara Bio) and Thermal Cycler LifeECO (Nippon Genetics). Oligonucleotide primers for PCR were purchased from Hokkaido System Science Co., Ltd. (Hokkaido, Japan) and listed in Supplementary Table 4. Analytical and preparative TLC was performed on silica gel 60 F_254_ (Merck) and RP-18 F_254_S (Merck). Column chromatography and flash chromatography were carried out on silica gel 60 N (100–210 µm, Kanto Chemical) and silica gel 60 N (40–50 µm, Kanto Chemical), respectively. NMR spectra were recorded on a Burker AVANCE III spectrometer. Chemical shifts for ^1^H and ^13^C NMR are given in parts per million (δ) relative to tetramethylsilane (δ_H_ 0.00) and residual solvent signals (δ_C_ 77.0) for CDCl_3_ as internal standard. Reverse phase HPLC analysis was performed on HITACHI LaChrom Elite series equipped with L-2130 pump, L-2200 autosampler, L-2455 Diode Array Detector, and D-2000 system manager. LC-MS analysis was performed on HITACHI Chromaster series equipped with 5110 pump, 5430 Diode Array Detector, 5610 MS Detector, MSD system manager. High-resolution mass spectra were measured on a Thermo Fischer Scientific Exactive Mass spectrometer. UV spectra were recorded on a JASCO-V-730 spectrophotometer. IR spectra were recorded on JASCO-FT/IR-4200 spectrometer. Optical rotations were recorded on JASCO-P-1030.

### Genome mining and bio-informatics analyses of DDP pathways

Draft genome sequence of *A. sacchari* Kumo-3 was obtained in previous study (Supplementary Data 1)^69^. Draft genome sequences of *F. graminearum* PH-1, *M. phaseolina* MS6, *C. higginsianum* IMI349063 and *M. anisopliae* E6 were obtained from National Center for Biotechnology Information (NCBI). Genome mining was performed by Protein BLAST search against *A. sacchari* Kumo-3 genome sequence and the NCBI non-redundant database and 2ndFind program (http://biosyn.nih.go.jp/2ndfind/). The results of bioinformatics analyses are shown in Supplementary Tables 1–3.

### Fugal strains used as genomic DNA donor

*Arthrinium sacchari* (strain Kumo-3) was isolated from a spider previously^69^. Genomic DNA of *Fusarium graminearum* 50218 was obtained from the Medical Mycology Research Center, Chiba University (Chiba, Japan). *Macrophomina phaseolina* NBRC 7317 and *Metarhizium anisopliae* NBRC 103233 were obtained from the Biological Resource Center, National Institute of Technology and Evaluation (Chiba, Japan). *Colletotrichum higginsianum* MAFF 305635 was obtained from the Genetic Resource Center, National Agriculture and Food Research Organization (Ibaraki, Japan).

### Heterologous host

*Escherichia coli* DH5a (Competent quick, TOYOBO) was used for cloning experiments. *Aspergillus oryzae* NSAR1^47^, a quadruple auxotrophic mutant (*niaD*^−^, *sC*^−^, Δ*argB*, *adeA*^−^) was used as the host for fungal expression.

### Preparation of fungal genomic DNA

*A. sacchari* Kumo-3, *M. phaseolina* NBRC 7317, *M. anisopliae* NBRC 103233, and *C. higginsianum* MAFF 305635 were cultivated on Potato dextrose agar (PDA) (2.4% Potato dextrose broth (Difco), 1.5% agar). The spores and mycelium from the plate were inoculated into 60 mL of Potato dextrose medium (2.4% Potato dextrose broth). After several days cultivation at 30 °C (reciprocal shaking), the mycelia of each strain were collected by filtration, washed with water, and frozen at −80 °C. The frozen mycelium was ground to fine powder, suspended in TE buffer (pH 8.0), and equal volume of lytic buffer (2% SDS, 0.1 M NaCl, 10 mM EDTA, 50 mM Tris-HCl) was added. After incubation at room temperature for 5 min, the supernatant was extracted with phenol: chloroform: isoamyl alcohol (25:24:1) solution (pH 7.9). After ethanol precipitation, the isolated DNA was dissolved in TE buffer (pH 8.0) and stored at −20 °C before use.

### General methods for DNA engineering experiments

PCR was performed with PrimeSTAR® Max DNA Polymerase (Takara Bio) unless otherwise noted. PCR products and linearized plasmids were purified with QIAEX® II Gel Extraction Kit (QIAGEN). DNA cloning experiments were performed using *E. coli* DH5α (Competent Quick, TOYOBO) following standard techniques and recombinant plasmids were extracted with GenElute^TM^ Plasmid Miniprep Kit (Sigma-Aldrich) from *E. coli* cells.

### Construction of fungal expression plasmids

The genes in *dpfg*, *dpmp*, *dpch*, *dpas*, and *dpma* clusters were amplified by PCR from genomic DNA of *F. graminearum* 50218, *M. phaseolina* NBRC 7317, *C. higginsianum* MAFF 305635, *A. sacchari* Kumo-3, and *M. anisopliae* NBRC 103233 as templates by using gene specific primers listed in Supplementary Table 4, respectively. The full-length genes in each cluster were purified and inserted into restriction site (*Asp*718 or *Not*I) of the pUARA2, pUADEA2, pUSCA2, or pUPTRA2 (Supplementary Fig. 3 and Supplementary Note 1) to yield fungal expression plasmid. For construction of one of two genes containing plasmids, each gene was ligated with *Asp*718 or/and *Not*I-digested pUARA2, pUADEA2, pUSCA2, or pUPTRA2 vectors using In-Fusion® HD Cloning Kit (Takara Bio) (Supplementary Fig. 4a). For construction of three or four genes containing plasmids, two-gene containing DNA fragments were amplified by PCR from the corresponding plasmids with two genes and ligated with the linearized vector fragment(s) as described above (Supplementary Fig. 4b). All the overexpression plasmid vectors made in this study are listed Supplementary Table 5.

### Transformation of *A. oryzae*

Transformation of *A. oryzae* was performed using the protoplast-polyethylene glycol (PEG) method as follows: *A. oryzae* host strains were cultivated on agar plate (PDA supplemented with 0.05% L-arginine and 0.05% adenine for NSAR1, CD agar supplemented with appropriate nutrients and chemicals (Supplementary Table 6) for transformants). The spores and mycelia of the host strain were inoculated into 60 mL of Czapek-Dox -Casamino acids medium (3.5% Czapek-Dox broth (Difco), 0.5% Casamino acids vitamin assay (Difco, when *ptrA* was used as a selectable marker) or Casamino acid (Difco, when *ptrA* was not used as a selectable marker), 0.01% adenine (for adenine auxotrophic strains)). After 20 hour cultivation at 30 °C (reciprocal shaking), mycelium was collected by filtration and washed with water. The mycelium was incubated with 10 mL protoplasting buffer (5 mg mL^−1^ Yatalase (Takara Bio), 1 mg mL^−1^ Cellulase Onozuka R-10 (SERVA Electrophoresis GmbH), 0.8 M NaCl, 10 mM phosphate buffer (pH 6.0)). After 3-h incubation at 30 °C, undigested mycelia was removed by filtration with Miracloth (Merck), and the protoplasts were collected by centrifugation at 1300 × *g* for 5 min. The protoplasts were washed with 0.8 M NaCl, resuspended in solution I (0.8 M NaCl, 10 mM CaCl_2_, 10 mM Tris-HCl (pH 8.0)) to obtain 2.0 × 10^8^ cells mL^−1^. 0.2 Volumes of solution II (40% PEG4000, 50 mM CaCl_2_, 50 mM Tris-HCl (pH 8.0)) was added with gentle mixing, and then 200 µL of this protoplast suspension was combined with 10–20 µg of plasmid DNA (<20 µL). After 40 min incubation on ice, 1 mL of solution II was added to the aliquot with gentle mixing. After another 15 min of incubation at room temperature, 20 mL of solution I was added to the mixture and the protoplasts were collected by centrifugation at 2000 rpm for 5 min. The protoplasts were resuspended in 600 µL of solution I, and the suspension was combined with molten soft-top agar (3.5% Czapek-Dox broth, 0.8 M NaCl, 0.6% agar) supplemented with appropriate nutrients (Supplementary Table 6). Subsequently, the protoplasts were plated on CD agar plates (3.5% Czapek-Dox broth, 0.8 M NaCl, 1.5% agarose) with appropriate nutrients (Supplementary Table 6). After 3–7 days incubation at 30 °C, the resultant cells (transformats) were transferred and subcultured on CD agar plates with appropriate nutrients (Supplementary Table 6).

### Reconstitution of the DDP pathways

The DDP biosynthetic genes were stepwisely introduced in *A. oryzae* NSAR1 using expression plasmids containing the aforementioned genes to construct all the transformants expressed the native DDP pathways (Supplementary Figs. 5, 6, 11, 16, 20, 24, 28 and Supplementary Notes 2, 7, 9, 11, 13), shunt pathway (Supplementary Fig. 30 and Supplementary Notes 15, 16, 17) and extended pathways (Supplementary Figs. 34, 39 and Supplementary Notes 19, 21)

### Gene integration analysis of each *A. oryzae* transformant

We obtained about four to ten transformants per one-transformation experiments. Thus, we checked gene insertion of all the transformant by PCR. Target gene integration into the *A. oryzae* transformants were confirmed by PCR with the genomic DNA template and primers in Supplementary Table 4. Genomic DNA of the transformant was extracted as follows; small pieces of mycelium of the *A. oryzae* strain were collected from an agar plate after several days growth at 30 °C and incubated at 95 °C in TE (pH 8.0) buffer for 10 min. The genomic DNA solution was diluted with water to prepare the PCR template at appropriate concentration.

### Cultivation and metabolite analysis of *A. oryzae* transformants

All the transformant that pass the gene integration check were cultivated and analysed their metabolite profiles by reverse phase HPLC and LC-MS analyses. Each transformant was inoculated into 60 mL of CPS medium (3.5% Czapek-Dox broth, 0.3% casein peptone (Nacalai Tesque), 0.3% meat peptone (Nacalai Tesque), 0.3% soy peptone, 2% soluble starch (Nacalai Tesque), 1% maltose monohydrate (Nacalai Tesque), 0.01 % adenine (for adenine auxotrophic strains)) or 1/2 CPS medium (1.75% Czapek-Dox broth, 0.15% casein peptone (Nacalai Tesque), 0.15% meat peptone (Nacalai Tesque), 0.15% soy peptone, 1% soluble starch (Nacalai Tesque), 0.5% maltose monohydrate (Nacalai Tesque), 0.01 % adenine (for adenine auxotrophic strains). About 1 week (6–10 days) after cultivation at 30 °C, 150 rpm, the culture media and mycelium were separated by filtration. The culture media (3 mL) was extracted with EtOAc (2 mL), and the organic layer was concentrated in vacuo. The residue was then dissolved in 150 µL of MeOH to prepare sample for reverse phase HPLC and LC-MS analysis. The freeze-dried mycelium (20 mg) was extracted with MeOH (1 mL), and the extract was concentrated in vacuo. The residue was then dissolved in 100 µL of MeOH to prepare sample for HPLC and LC-MS analysis. For the HPLC and LC-MS analysis, 10 µL of the samples were used. The HPLC analysis was performed on COSMOSIL 5C_18_ Packed Column (4.6 mm I.D. × 150 mm, Nacalai Tesque) with acetonitrile and water containing 0.01% trifluoroacetic acid (0–1.5 min: 20:80, 1.5–11.5 min: a linear gradient from 20:80 to 100:0, 11.5–20 min: 100:0) at a flow rate of 1.0 mL min^−1^. The LC-MS analysis was performed on COSMOSIL 5C_18_ Packed Column (4.6 mm I.D. × 150 mm, Nacalai Tesque) with acetonitrile containing 0.1% formic acid and water containing 0.1% formic acid (0–2 min: 20:80, 2–12 min: a linear gradient from 20:80 to 100:0, 12–20 min: 100:0) at a flow rate of 1.0 mL min^−1^ using positive mode electro spray ionization.

### Isolation and structure determination of each compound

To isolate DDPs produced by the transformants, we picked up the transformants that produced target DDPs the most and performed scaling up cultivation of the transformant. Each *A. oryzae* transformant was cultivated in 1/2 CPS medium (1.75% Czapek-Dox broth, 0.15% casein peptone (Nacalai Tesque), 0.15% meat peptone (Nacalai Tesque), 0.15% soy peptone, 1% soluble starch (Nacalai Tesque), 0.5% maltose monohydrate (Nacalai Tesque), 0.01% adenine (for adenine auxotrophic strains) for 1 week at 30 °C, and the culture media and mycelium were separated by filtration. The culture media was extracted with EtOAc two times, and the organic layer were combined and concentrated in vacuo to give crude extract. The freeze-dried mycelium was extracted with EtOAc (20% MeOH), and the extract was concentrated in vacuo. The mycelia extract was then dissolved in MeOH (0.1% water), washed with hexane three times, concentrated in vacuo to give crude extract. Each of the crude extract of culture and mycelium was fractionated by silica gel column chromatography, and subjected to further purification by flash chromatography and preparative TLC (SiO_2_ or ODS). All the DDP structures were fully determined by spectral analyses (Supplementary Figs. 8–10, 14, 15, 18, 19, 22, 23, 26, 27, 32, 33, 37, 38, 41, 42, 52, 54–77, Supplementary Tables 7–18, 25 and Supplementary Notes 3–6, 8, 10, 12, 14, 18, 20, 22, 31).

### Evaluation of biological activities of DDP-focused library

Purified DDPs was dissolved in DMSO (Specially Prepared Reagent, Nuclease and Protease tested, NACALAI, 09659-14) to prepare 5 mM solutions. The Each DDP solution (5 mM) was used for antiproliferative effects on cancer cell lines (Supplementary Notes 23, 24, 25, 26), biological activity against *Drosophila* (Supplementary Note 27), anti-HIV assay (Supplementary Note 28) and screening of amyloid Ab42 aggregation inhibitors (Supplementary Notes 29).

### Reporting summary

Further information on research design is available in the Nature Research Reporting Summary linked to this article.

## Supplementary information

Supplementary Information

Peer Review

Reporting Summary

Description of Additional Supplementary Files

Supplementary Data 1

## Data Availability

The data supporting the findings of this work are available within the paper and its Supplementary Information files. A reporting summary for this Article is available as a Supplementary Information file. The data sets generated and analyzed during this study are available from the corresponding author upon request. The sequences data of *dpasA-F* and Dpas A-F are provided as a Supplementary Data 1. The source data underlying Figs. 5a, c, d, 6a, b, 7a, b, Supplementary Figs. 44, 46a, 47, and Supplementary Tables 20, 23, and 24 are provided as a Source Data file.

## Source Data

Source Data
