# Supplementary material for: Synthetic biology based construction of biological activity-related library of fungal decalin-containing diterpenoid pyrones
Source: Nat Commun. 2020 Apr 14;11:1830. doi: 10.1038/s41467-020-15664-4 (PMC7156458; doi:10.1038/s41467-020-15664-4)
Supplement: Supplementary file 1 — Supplementary Information [file 41467_2020_15664_MOESM1_ESM.pdf]

Synthetic biology based construction of biological activity-related library of fungal decalin-containing diterpenoid pyrones

Tsukada *et al.*

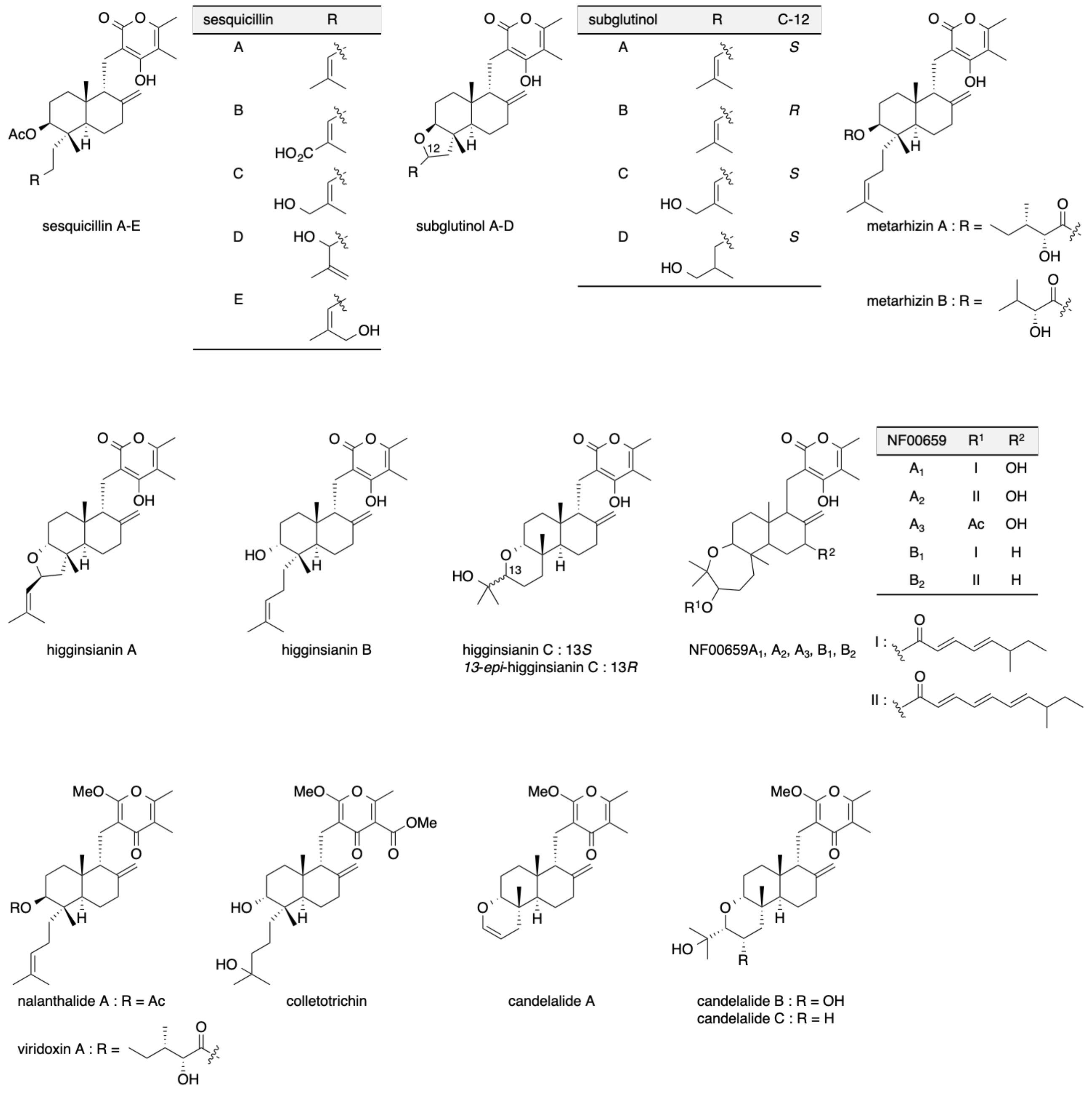

Supplementary Fig. 1. Previously reported fungal DDPs.

a

b

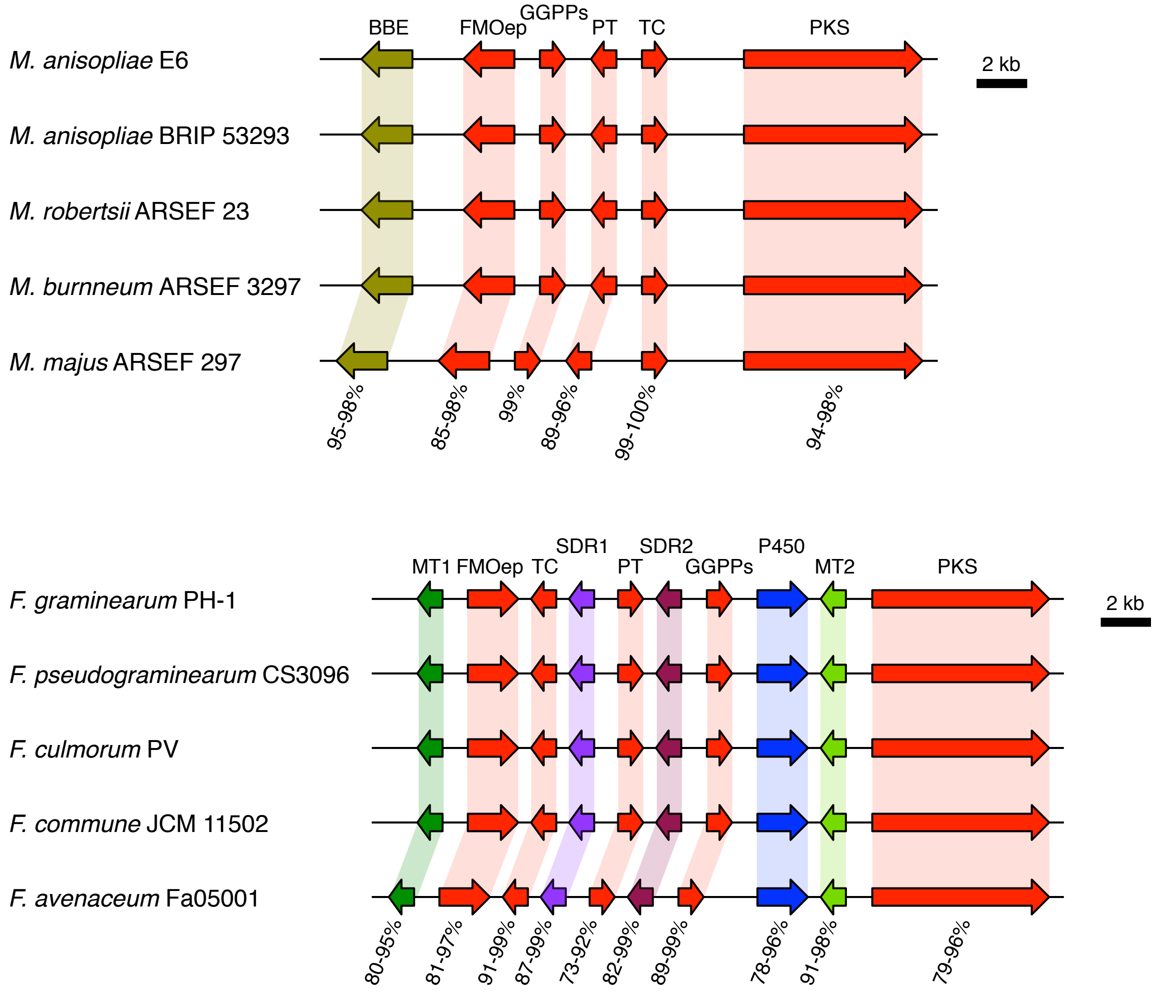

Supplementary Fig. 2. Conserved DDP biosynthetic gene clusters in *Metarhizium* and *Fusarium* fungi. **a,** Conserved *dpma* homologous biosynthetic gene clusters in genus *Metarhizium* (amino acid sequence of conserved 6 genes is 85-100% identical), **b,** Conserved *dpfg* homologous biosynthetic gene clusters in genus *Fusarium* (amino acid sequence of conserved 10 genes is 73-99% identical). Comparison of each gene was performed by protein BLAST.

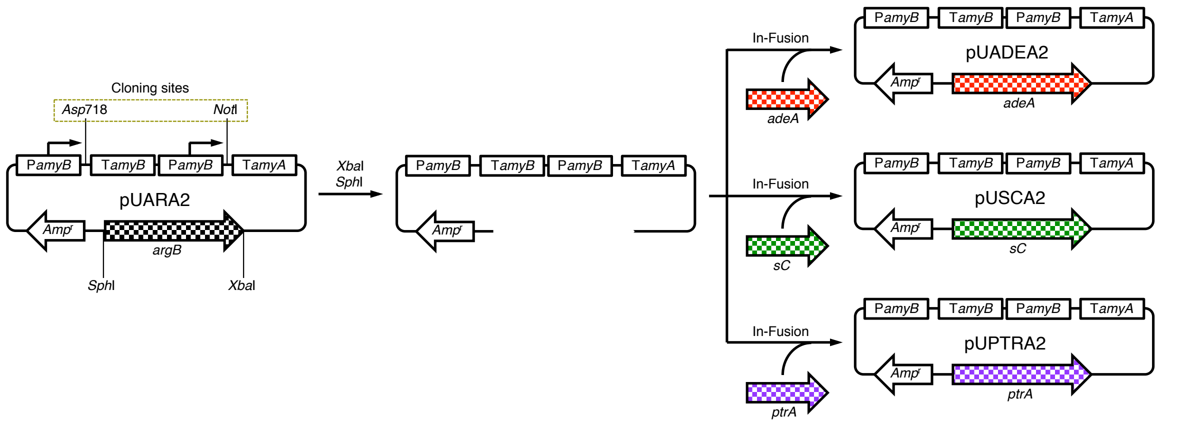

Supplementary Fig. 3. Construction of the pUADEA2, pUSCA2 and pUPTRA2 vector. P*amyB*, *A. oryzae amyB* promoter (inducible by starch or maltose); T*amyB*, *A. oryzae amyB* terminator; T*amyA*, *A. nidulans amyA* terminator; *Amp*^{r}, ampicillin resistance gene as an *E. coli* selectable marker.

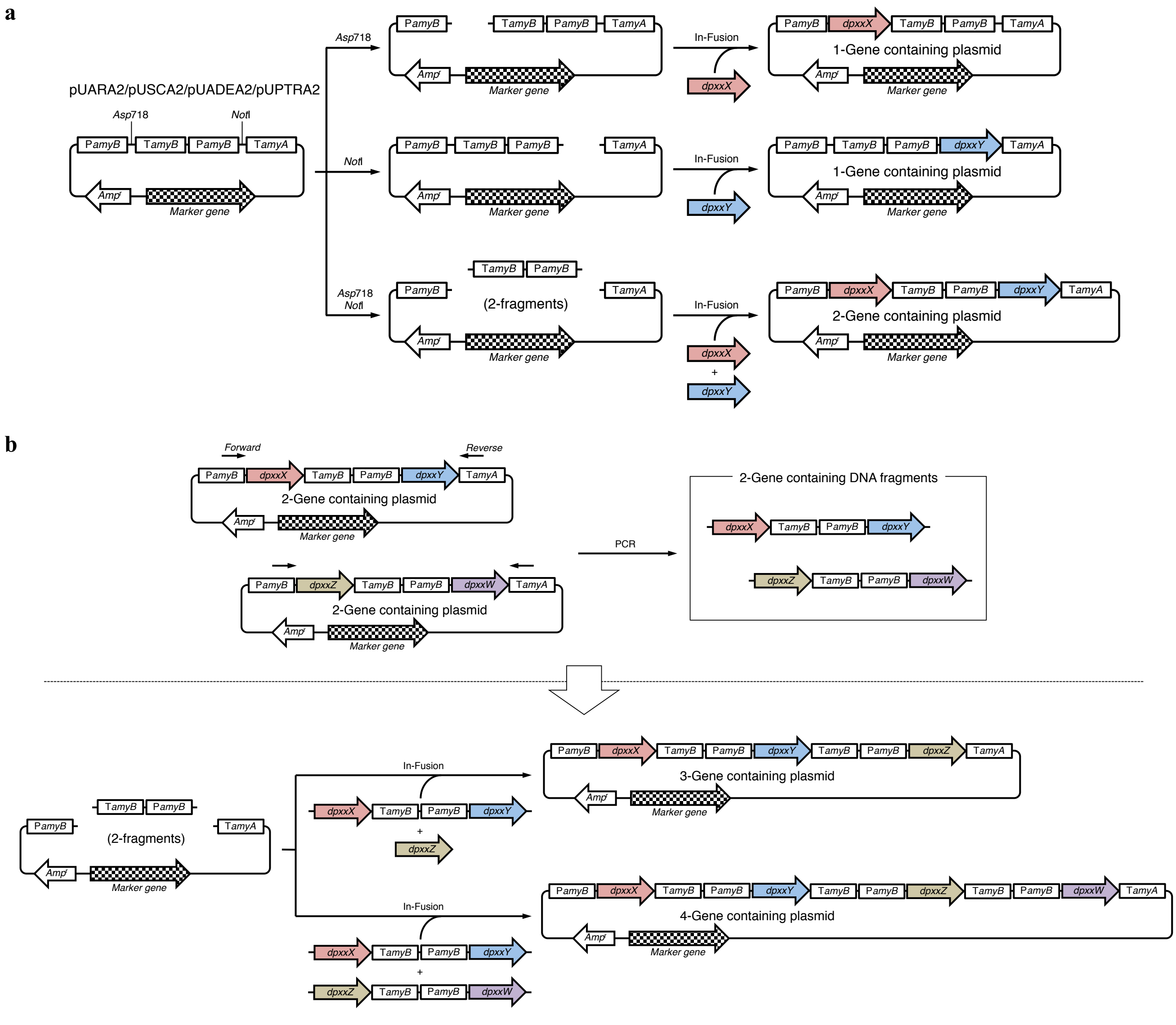

Supplementary Fig. 4. General scheme of constructing fungal expression plasmids. **a**, Construction of plasmid vector with one or two DDP biosynthetic genes; **b**, Construction of plasmid vector with three or four DDP biosynthetic genes.

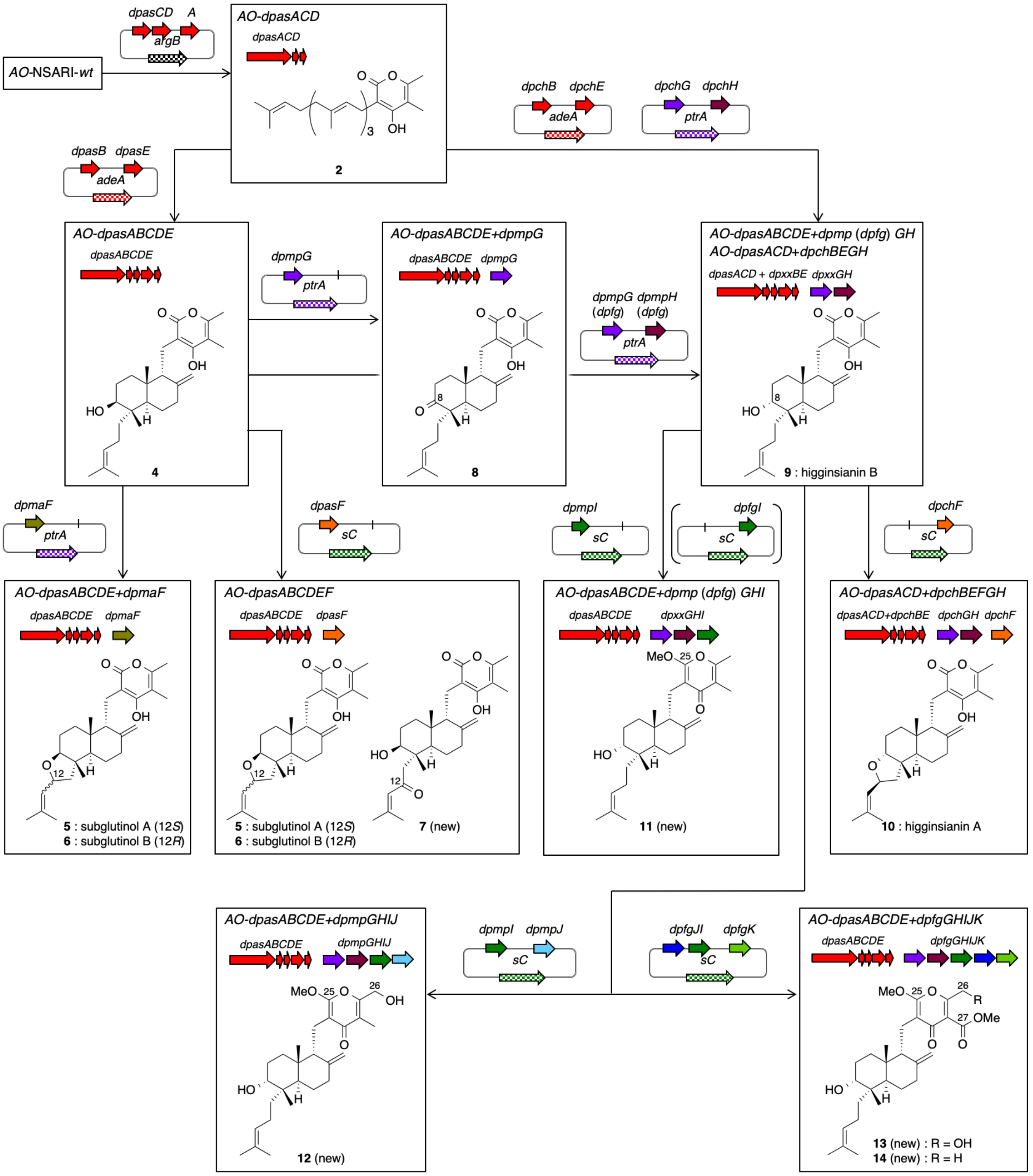

Supplementary Fig. 5. Reconstitution scheme of the native DDP pathways in *A. oryzae*.

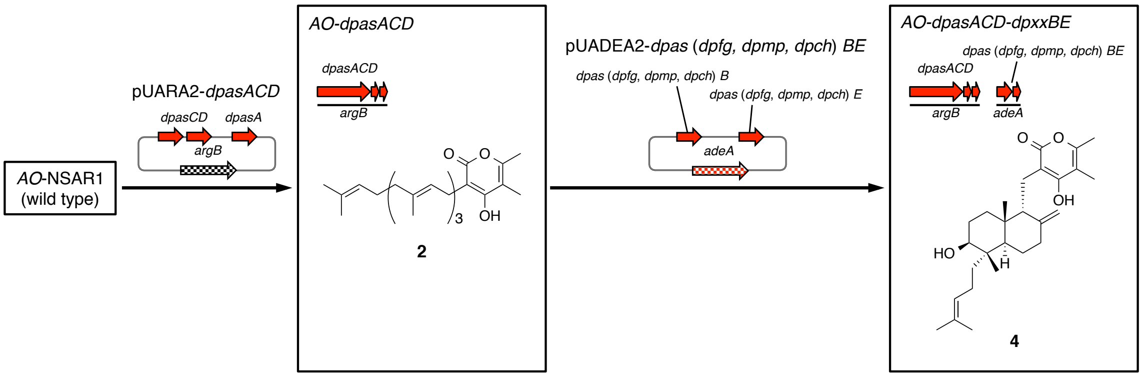

Supplementary Fig. 6. Reconstitution scheme of *AO-dpasACD* and *AO-dpasACD-dpxxBE*.

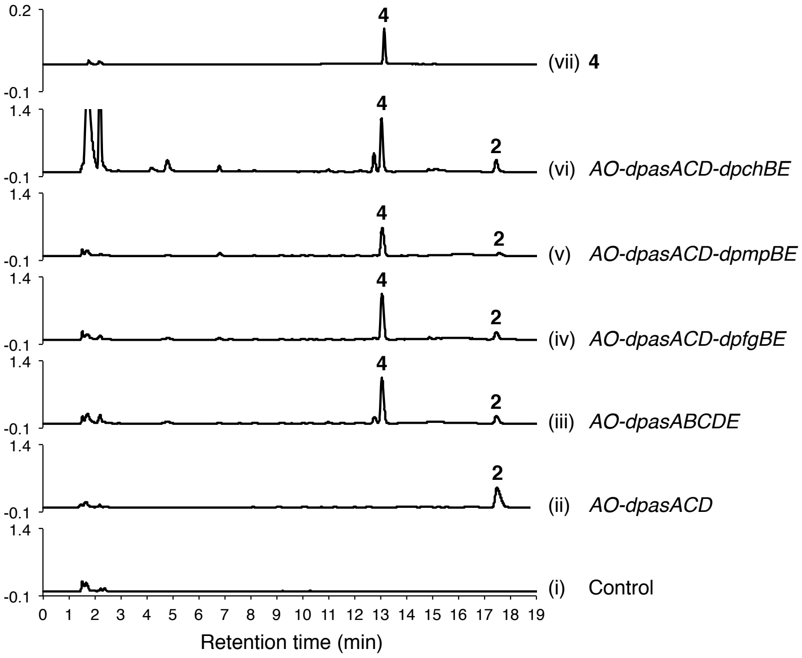

Supplementary Fig. 7. Reverse phase HPLC profiles of mycelia extracts from *A. oryzae* transformants. (i) *A. oryzae* NSAR1 harboring pUARA2 (empty vector); (ii) *AO-dpasACD*; (iii) *AO-dpasABCDE*; (iv) *AO-dpasACD-dpfgBE*; (v) *AO-dpasACD-dpmpBE*; (vi) *AO-dpasACD-dpchBE*; (vii) purified **4** (1 μg). The chromatograms were monitored at 280 nm. Non-marked peaks were not DDPs. We didn't characterize them, but they may be derived from polyketide **1**, see Supplementary Figure 13 (ii).

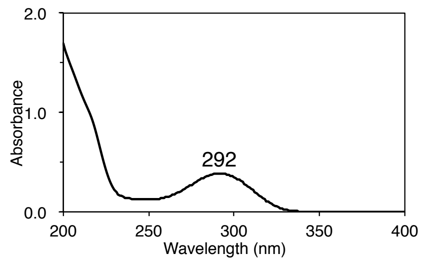

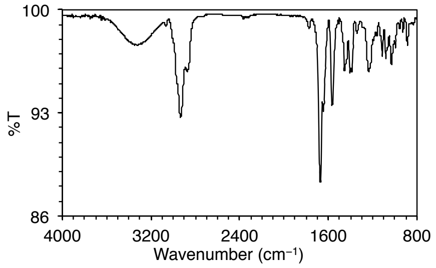

Supplementary Fig. 8. UV and IR spectra of **4**.

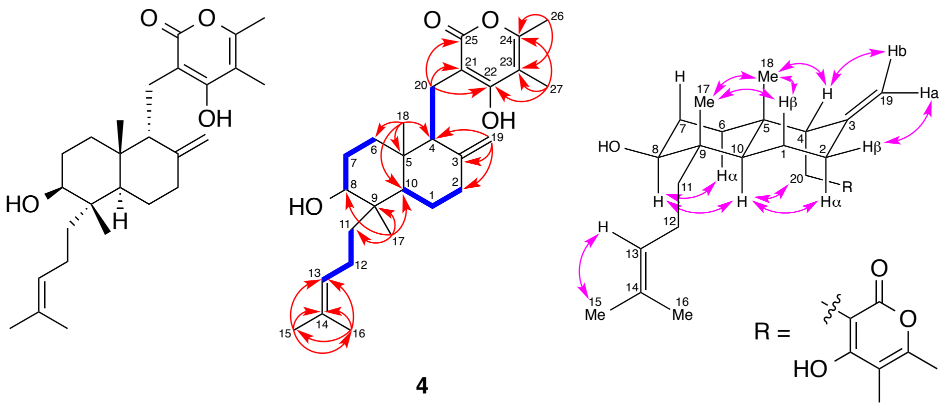

Supplementary Fig. 9. Key HMBC (red arrow), ^{1}H-^{1}H COSY (blue bold line) and NOE (purple arrow) correlations of **4**.

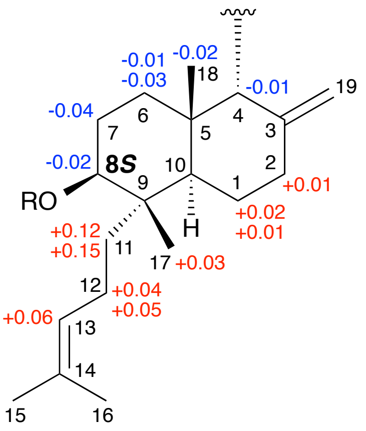

Supplementary Fig. 10. Δδ(*S*–*R*) values calculated from the NMR spectra of **15b** and **15c**.

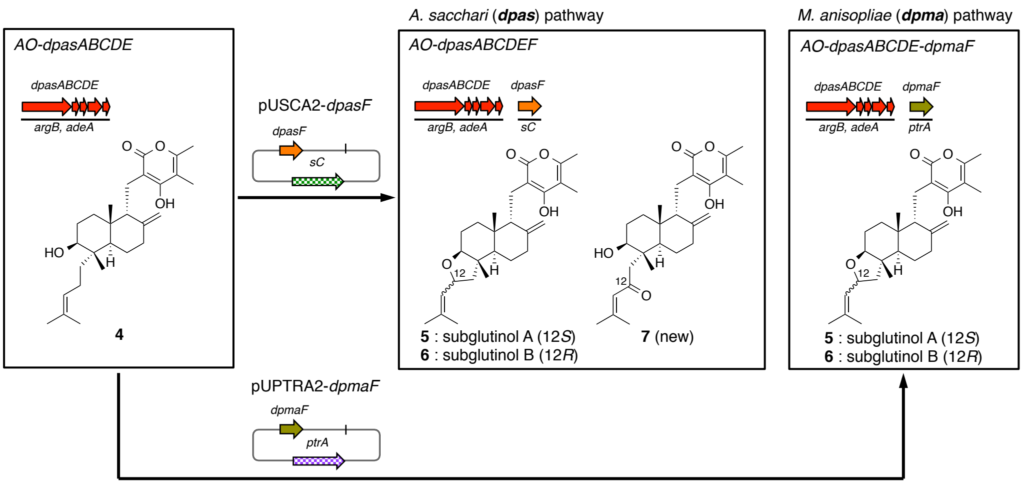

Supplementary Fig. 11. Reconstitution scheme of *AO-dpasABCDEF* and *AO-dpasABCDE-dpmaF*.

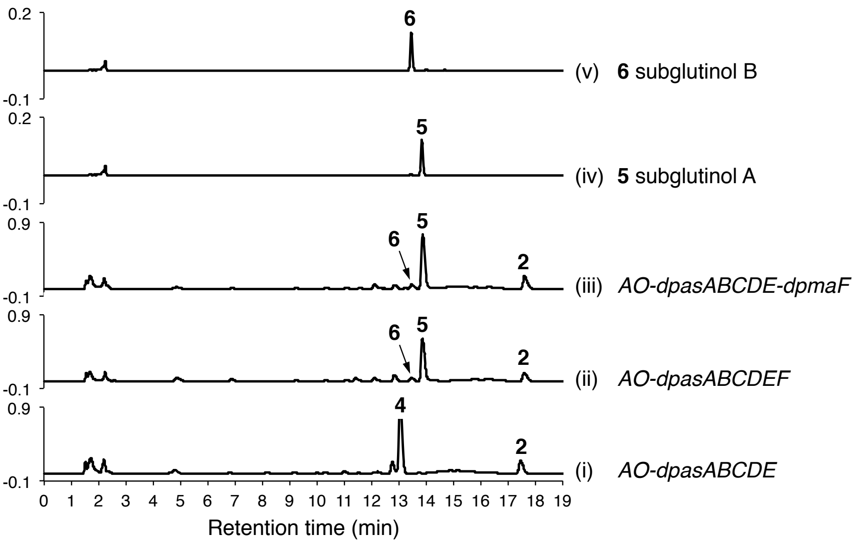

Supplementary Fig. 12. Reverse phase HPLC profiles of mycelia extracts from *A. oryzae* transformants. (i) *AO-dpasABCDE*; (ii) *AO-dpasABCDEF*; (iii) *AO-dpasABCDE-dpmaF*; (iv) purified subglutinol A (**5**) (1 μg); (v) purified subglutinol B (**6**) (1 μg). The chromatograms were monitored at 280 nm. Non-marked peaks were not DDPs. We didn't characterize them, but they may be derived from polyketide **1**, see Supplementary Figure 13 (ii).

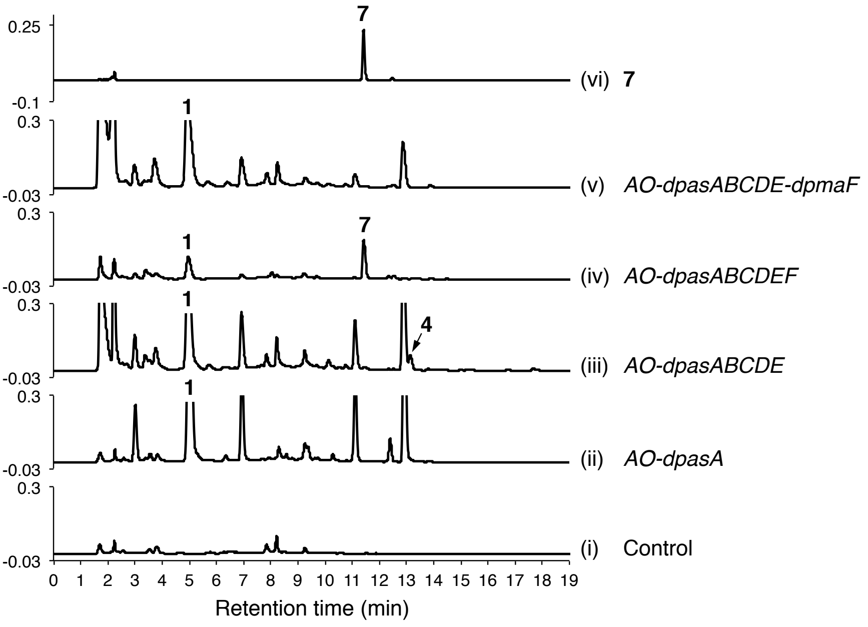

Supplementary Fig. 13. Reverse phase HPLC profiles of culture supernatant extracts from *A. oryzae* transformants. (i) *A. oryzae* NSAR1 harboring pUARA2 (empty vector); (ii) *AO-dpasA*; (iii) *AO-dpasABCDE*; (iv) *AO-dpasABCDEF*; (v) *AO-dpasABCDE-dpmaF*; (vi) purified **7** (1 μg). The chromatograms were monitored at 254 nm.

Compound 5

UV

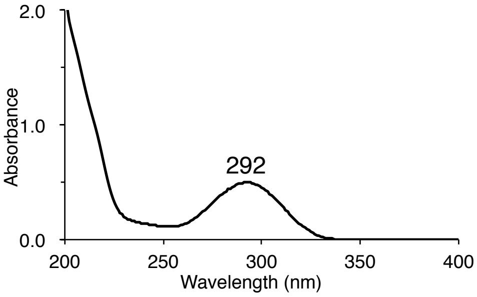

IR

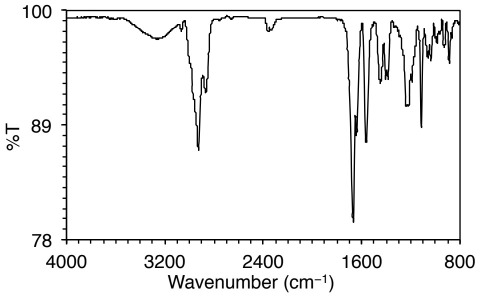

Compound 6

UV

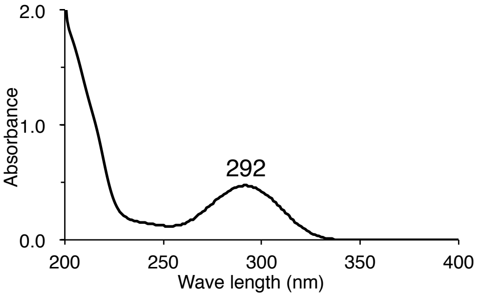

IR

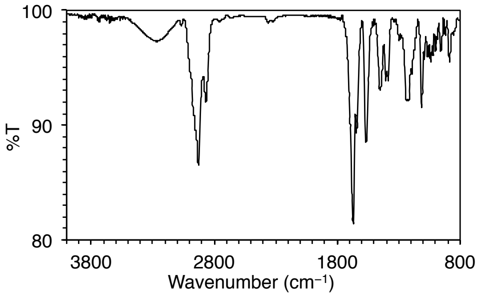

Compound 7

UV

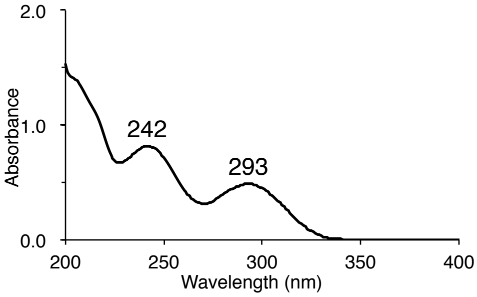

IR

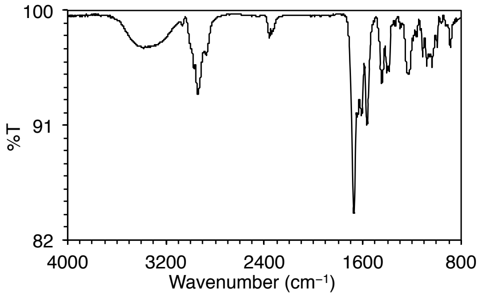

Supplementary Fig. 14. UV and IR spectra of **5**, **6** and **7**.

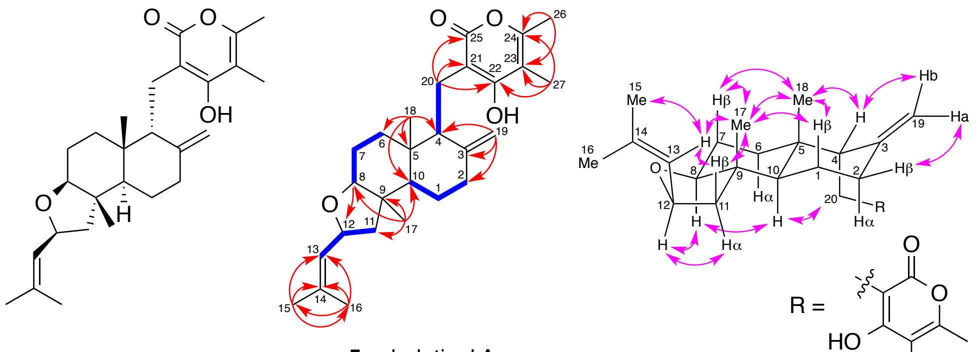

5 subglutinol A

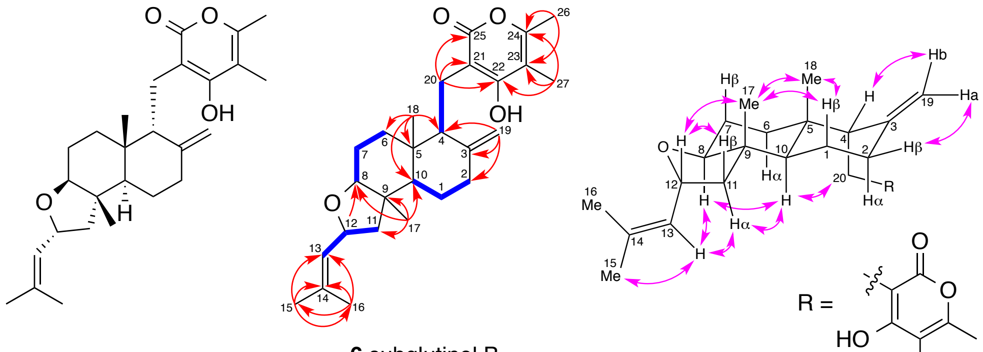

6 subglutinol B

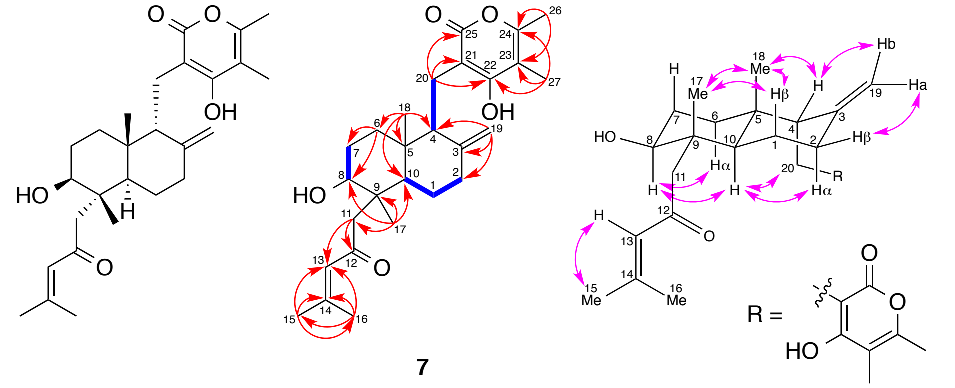

7

Supplementary Fig. 15. Key HMBC (red arrow), ^{1}H-^{1}H COSY (blue bold line) and NOE (purple arrow) correlations of **5**-**7**.

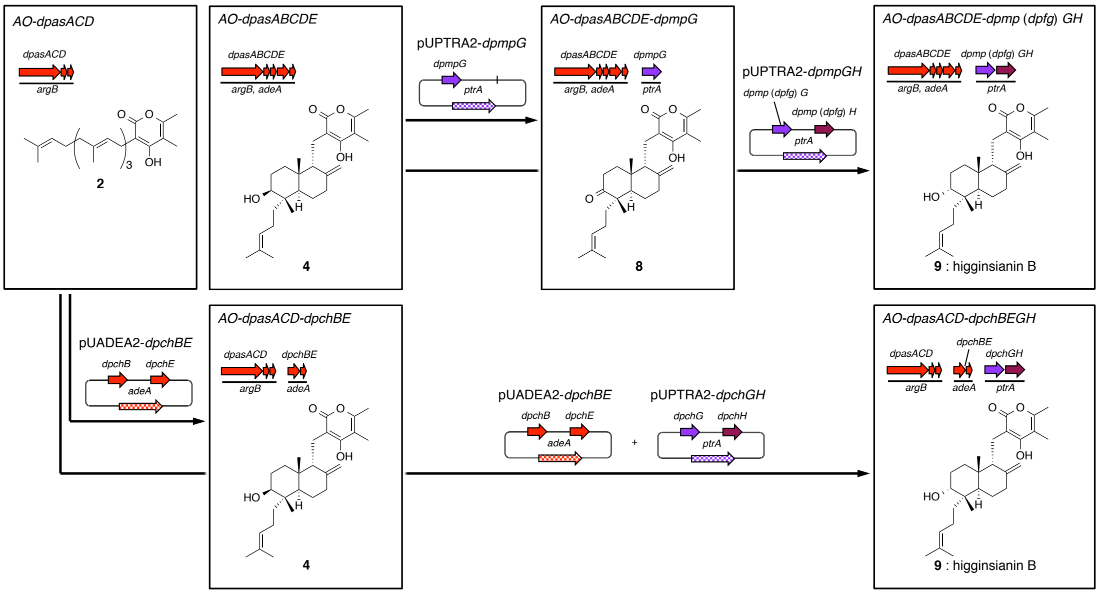

Supplementary Fig. 16. Reconstitution scheme of *AO-dpasABCDE-dpmpG*, *AO-dpasABCDE-dpmpGH*, *AO-dpasABCDE-dpfgGH* and *AO-dpasACD-dpchBEGH*.

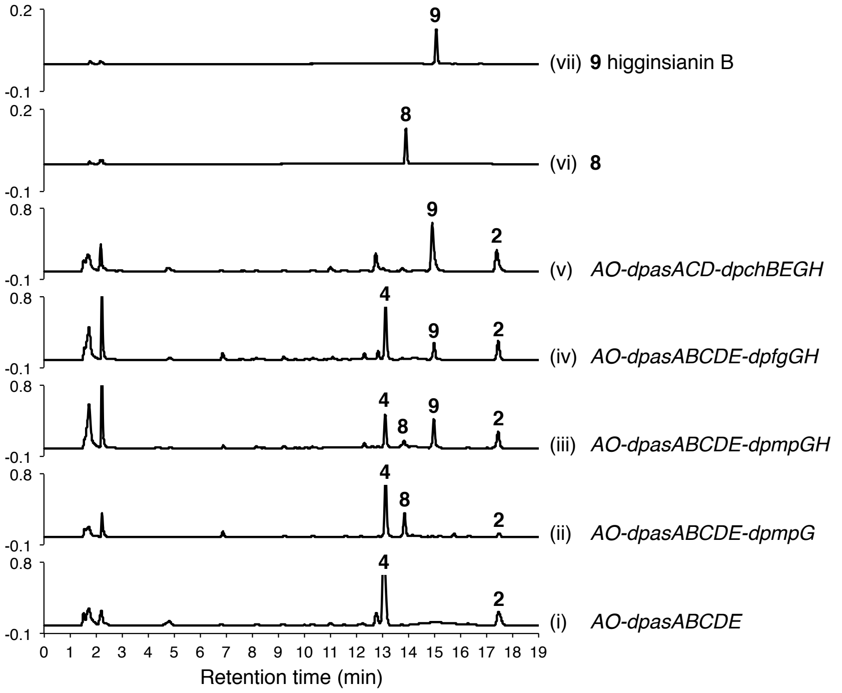

Supplementary Fig. 17. Reverse phase HPLC profiles of mycelia extracts from *A. oryzae* transformants. (i) *AO-dpasABCDE*; (ii) *AO-dpasABCDE-dpmpG*; (iii) *AO-dpasABCDE-dpmpGH*; (iv) *AO-dpasABCDE-dpfgGH*; (v) *AO-dpasACD-dpchBEGH*; (vi) purified **8** (1 μg); (vii) purified higginsianin B (**9**) (1 μg). The chromatograms were monitored at 280 nm. Compound **8** (BR-051) was reported in a patent (WO119950066464A1) https://patents.google.com/patent/WO1995006646A1/en. PCT. Int. Appl. (1995). Non-marked peaks were not DDPs. We didn't characterize them, but they may be derived from polyketide **1**, see Supplementary Figure 13 (ii).

Compound 8

UV

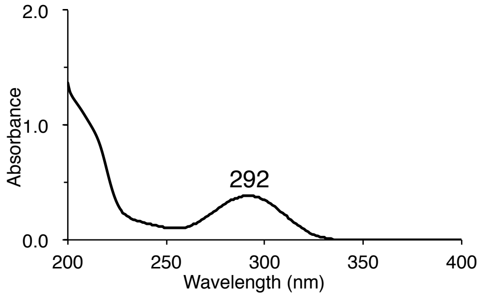

IR

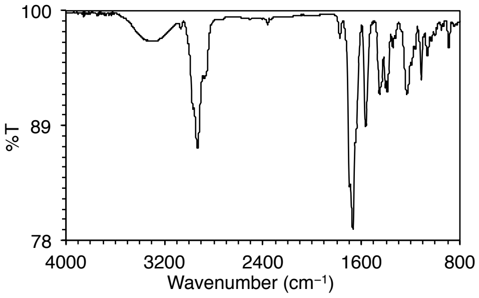

Compound 9

UV

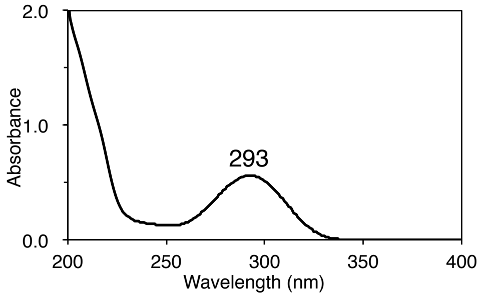

IR

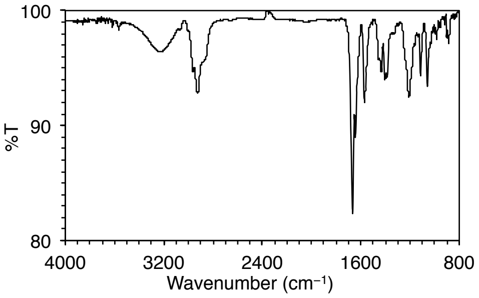

Supplementary Fig. 18. UV and IR spectra of **8** and **9**.

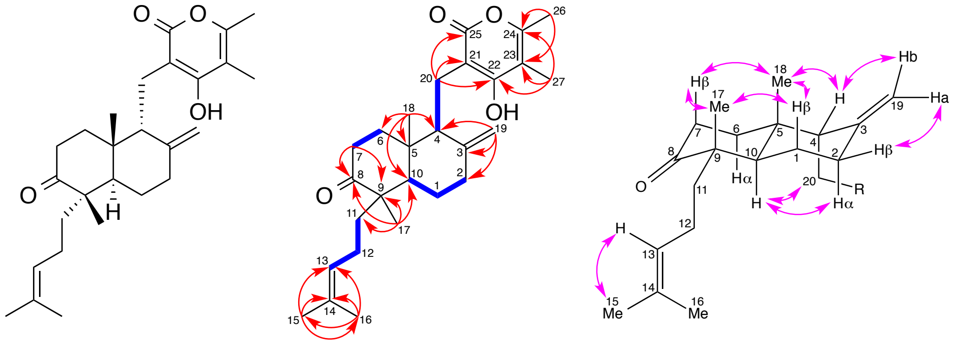

8

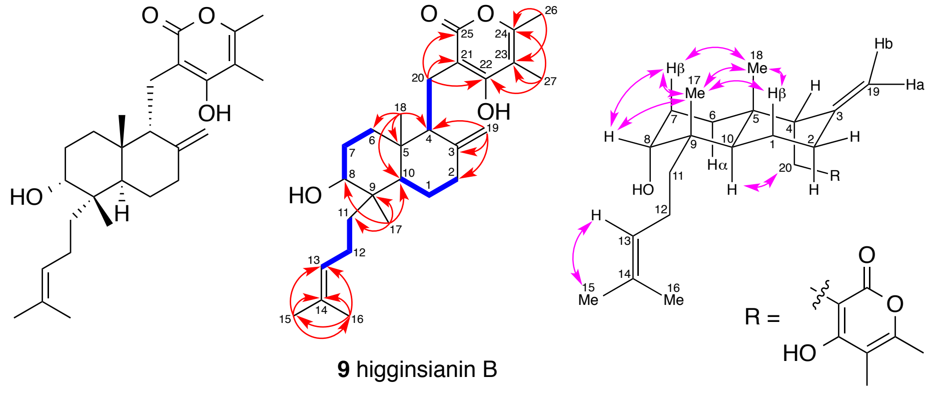

9 higginsianin B

Supplementary Fig. 19. Key HMBC (red arrow), ^{1}H-^{1}H COSY (blue bold line) and NOE (purple arrow) correlations of **8** and **9**.

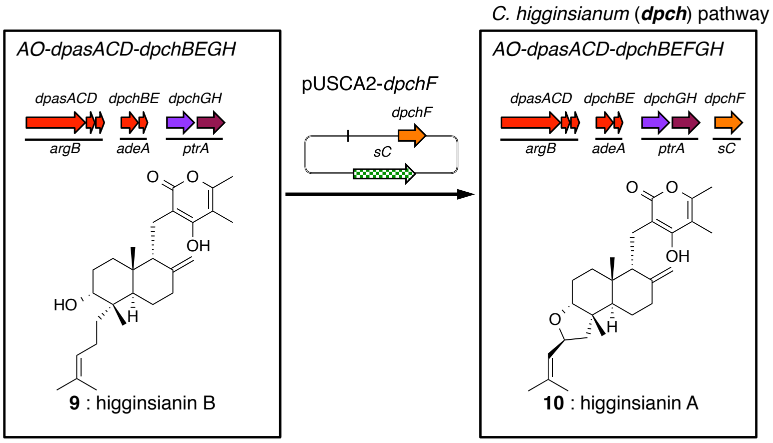

Supplementary Fig. 20. Reconstitution scheme of *AO-dpasACD-dpchBEFGH*.

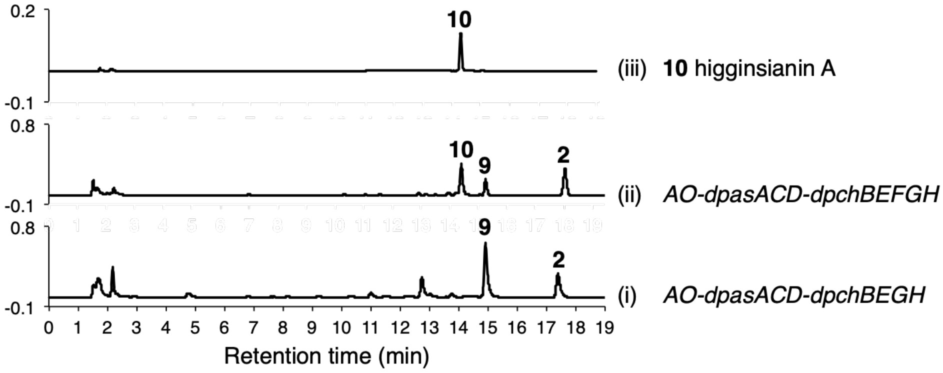

Supplementary Fig. 21. Reverse phase HPLC profiles of mycelia extracts from *A. oryzae* transformants. (i) *AO-dpasACD-dpchBEGH*; (ii) *AO-dpasACD-dpchBEFGH*; (iii) purified higginsianin A (**10**) (1 μg). The chromatograms were monitored at 280 nm. Non-marked peaks were not DDPs. We didn't characterize them, but they may be derived from polyketide **1**, see Supplementary Fig. 13 (ii).

Compound 9

UV

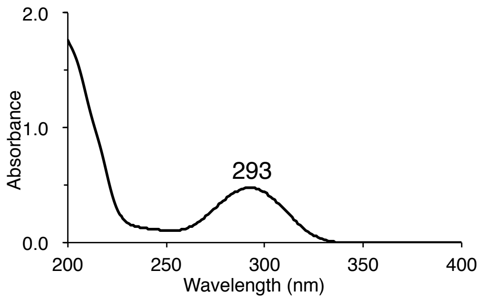

IR

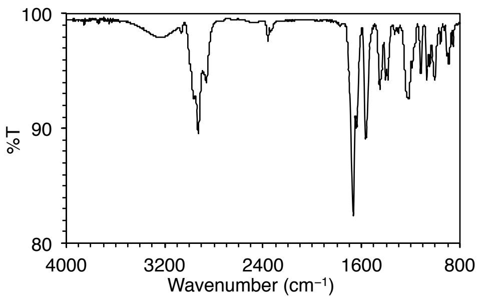

Supplementary Fig. 22. UV and IR spectra of **10**.

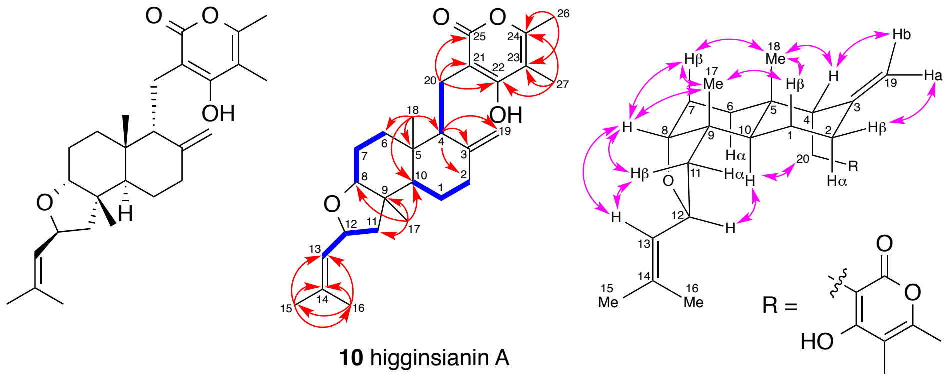

Supplementary Fig. 23. Key HMBC (red arrow), ^{1}H-^{1}H COSY (blue bold line) and NOE (purple arrow) correlations of **10**.

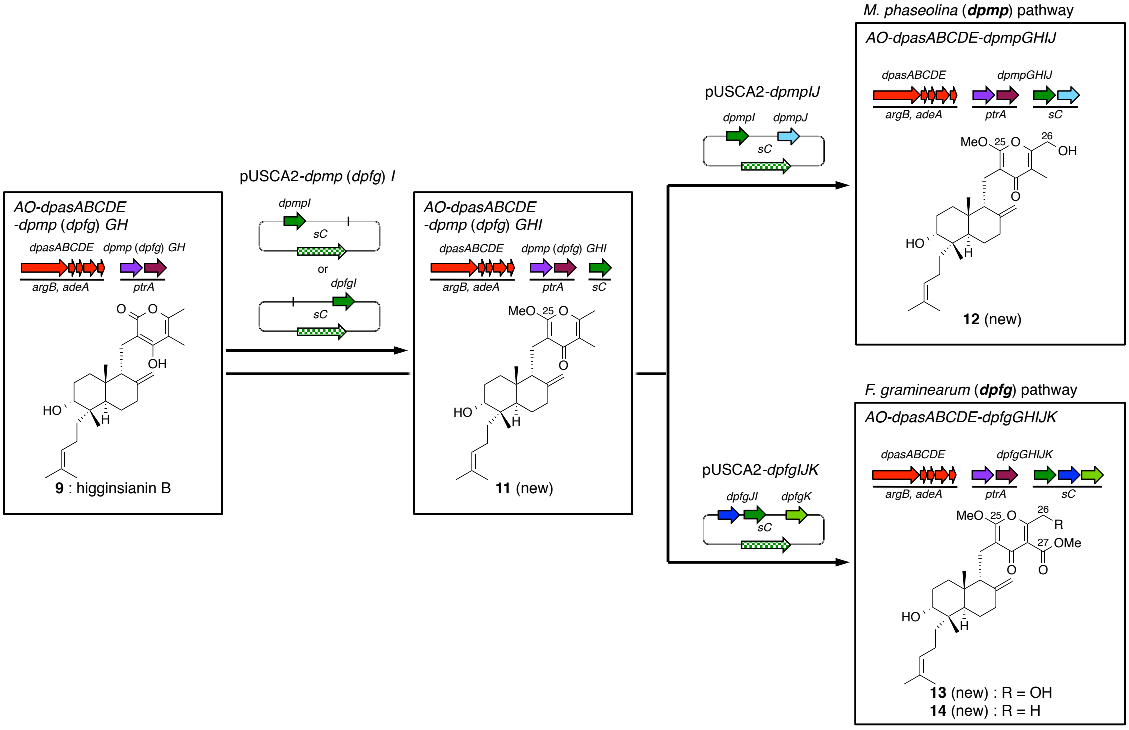

Supplementary Fig. 24. Reconstitution scheme of *AO-dpasABCDE-dpmpGHI*, *AO-dpasABCDE-dpmpGHIJ*, *AO-dpasABCDE-dpfgGHI* and *AO-dpasABCDE-dpfgGHIJK*.

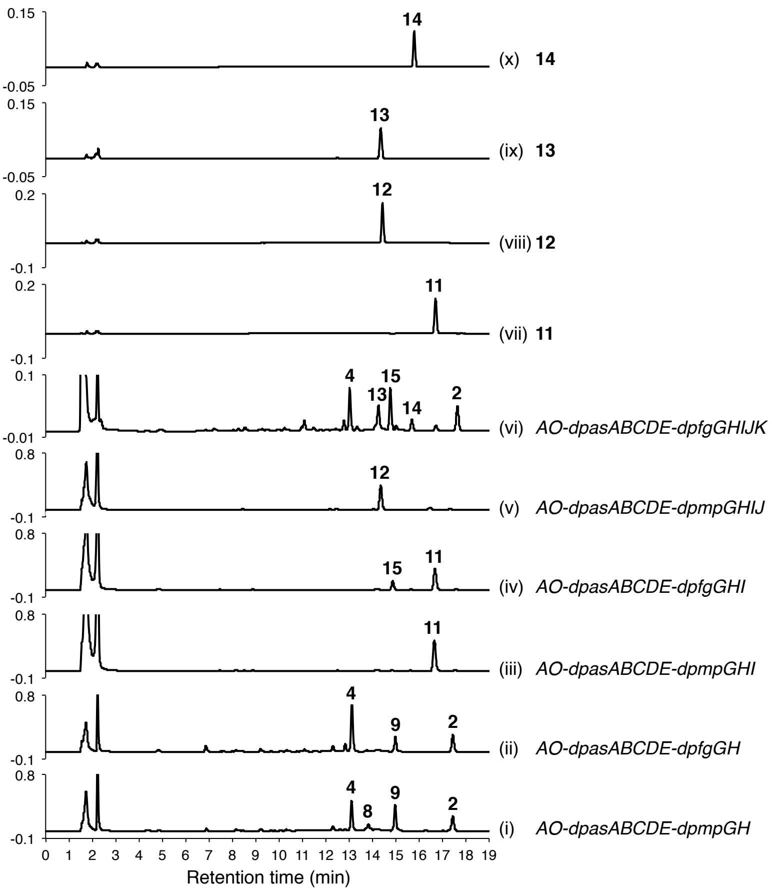

Supplementary Fig. 25. Reverse phase HPLC profiles of mycelia extracts from *A. oryzae* transformants. (i) *AO-dpasABCDE-dpmpGH*; (ii) *AO-dpasABCDE-dpfgGH*; (iii) *AO-dpasABCDE-dpmpGHI*; (iv) *AO-dpasABCDE-dpfgGHI*; (v) *AO-dpasABCDE-dpmpGHIJ*; (vi) *AO-dpasABCDE-dpfgGHIJK*; (vii) purified **11** (1 μg); (viii) purified **12** (1 μg); (ix) purified **13** (1 μg); (x) purified **14** (1 μg). The chromatograms were monitored at 280 nm (lane i and ii) and 254 nm (lane iii-x). Non-marked peaks were not DDPs. We didn't characterize them, but they may be derived from polyketide **1**, see Supplementary Fig. 13 (ii).

Compound 11

UV

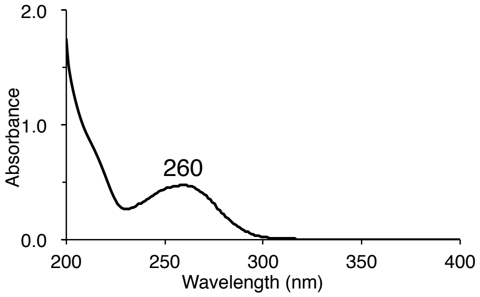

IR

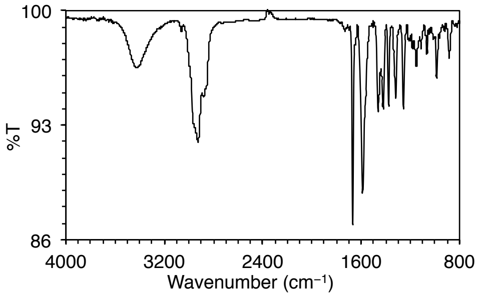

Compound 12

UV

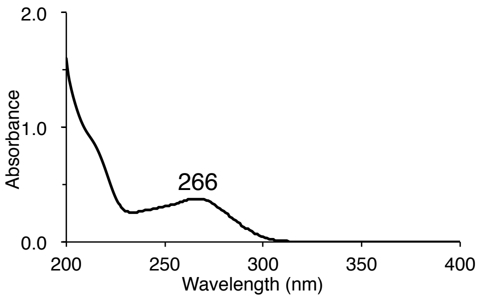

IR

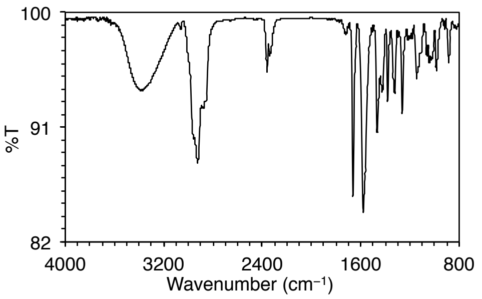

Compound 13

UV

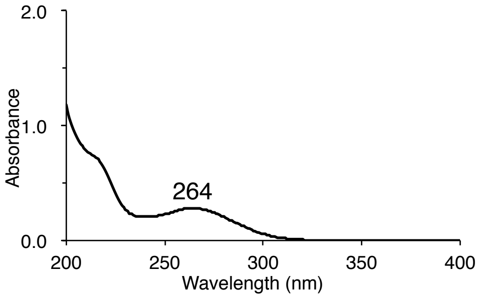

IR

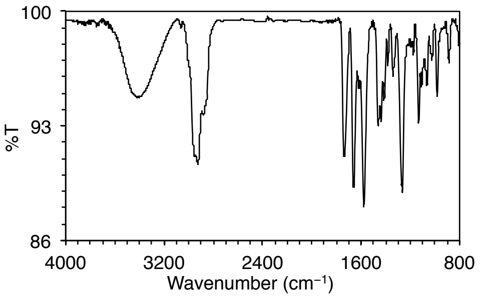

Compound 14

UV

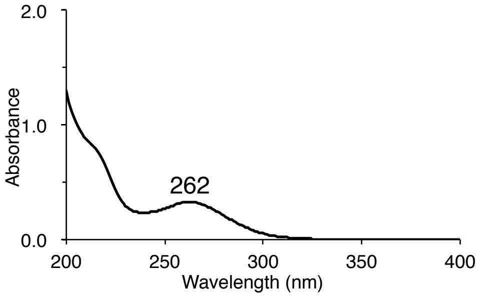

IR

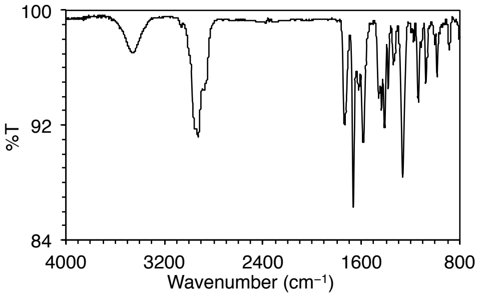

Supplementary Fig. 26. UV and IR spectra of **11–14**.

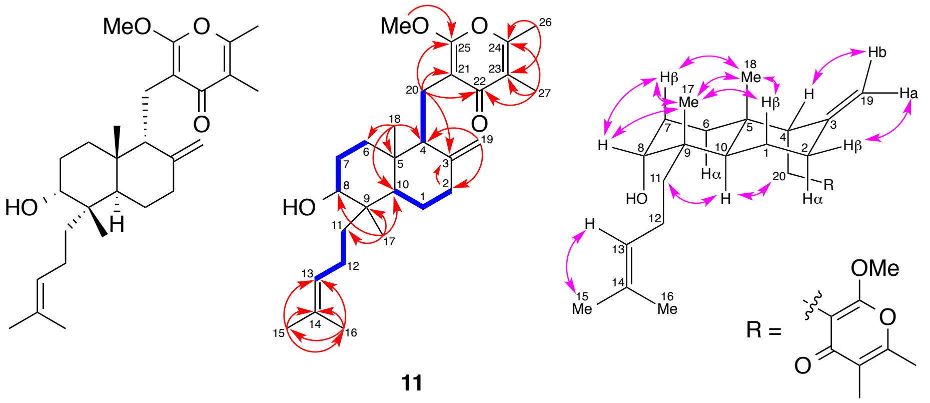

11

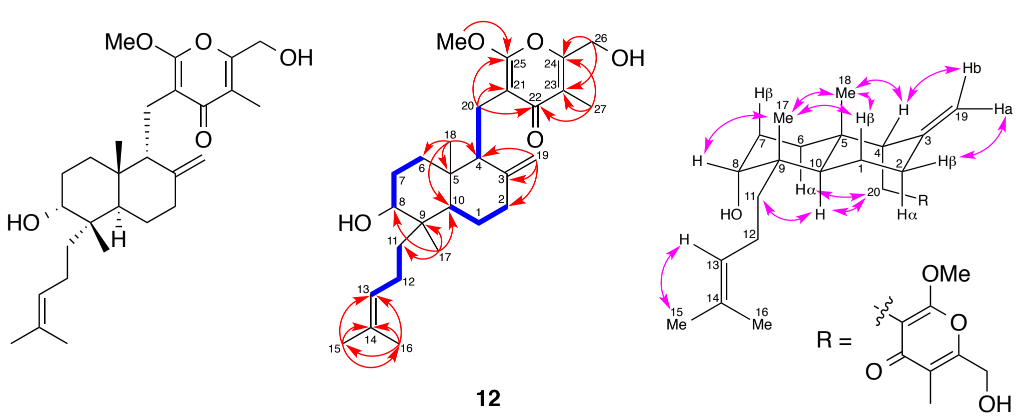

12

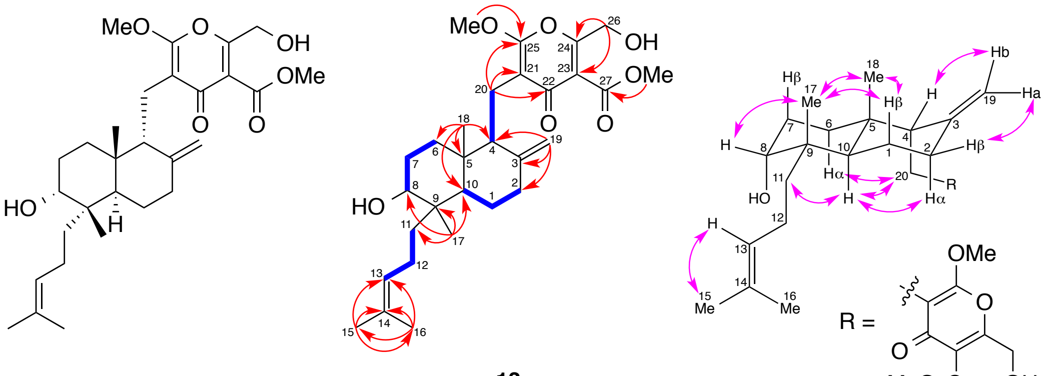

13

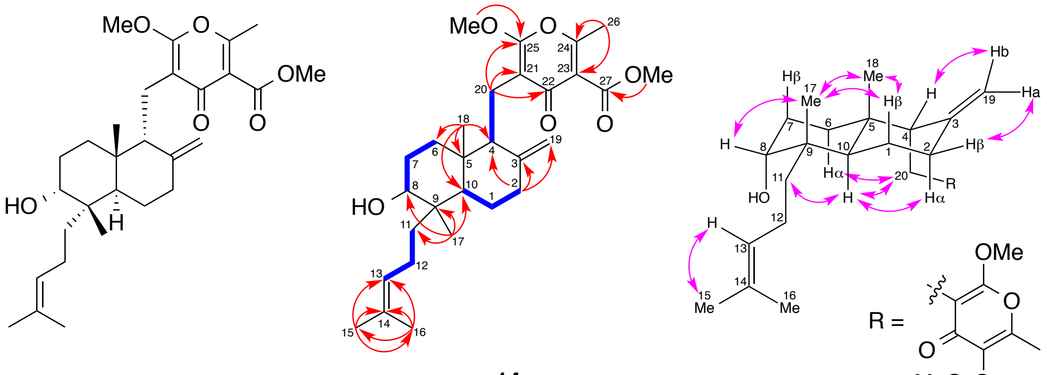

14

Supplementary Fig. 27. Key HMBC (red arrow), ^{1}H-^{1}H COSY (blue bold line) and NOE (purple arrow) correlations of **11**–**14**.

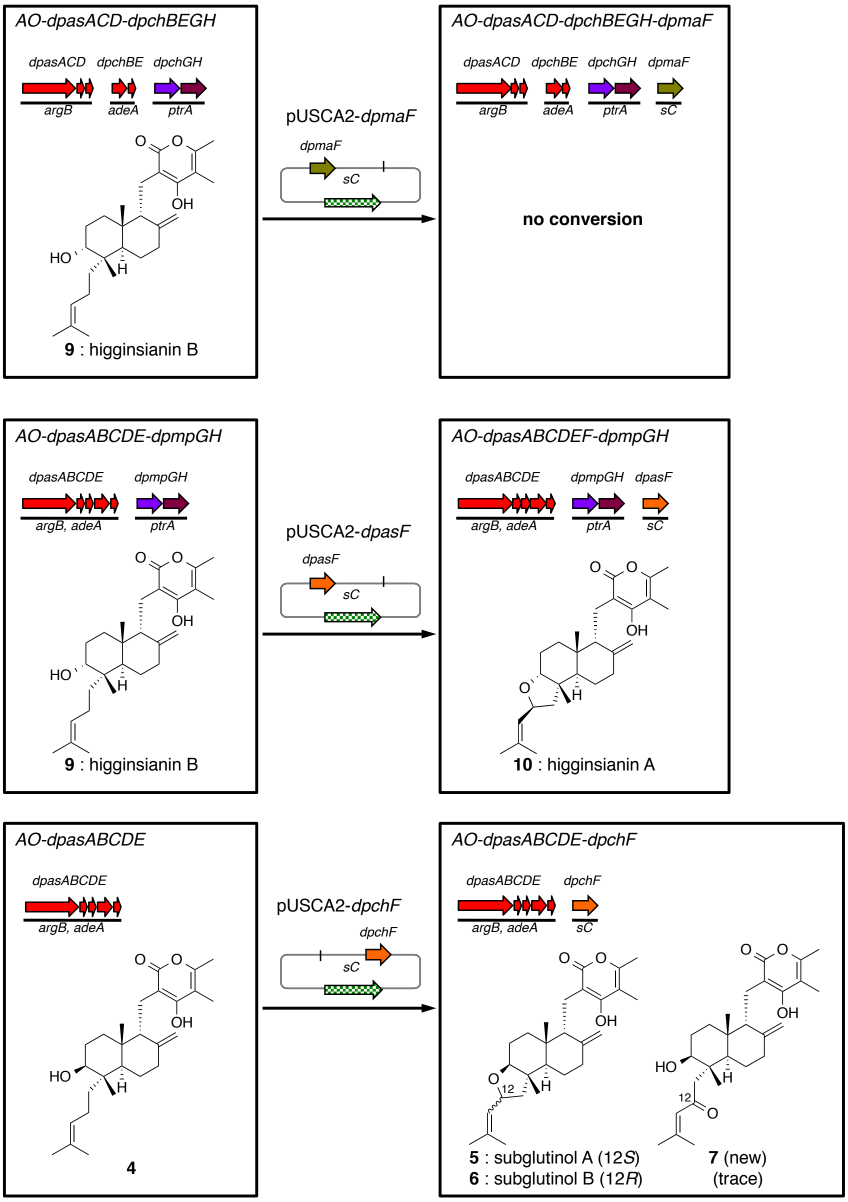

Supplementary Fig. 28. Reconstitution scheme of *AO-dpasABCDE-dpchF*, *AO-dpasABCDEF-dpmpGH*, *AO-dpasACD-dpchBEGH-dpmpF*.

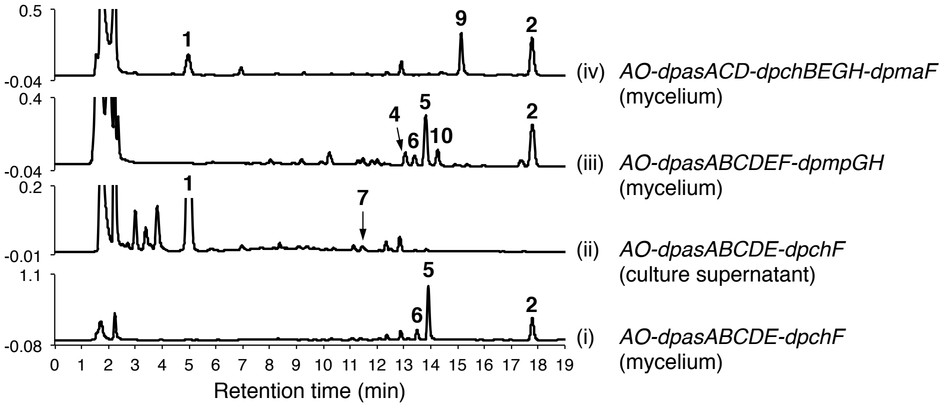

Supplementary Fig. 29. Reverse phase HPLC profiles of mycelia extracts (lane i, iii and iv) and culture supernatant (lane ii) extract from *A. oryzae* transformants. (i) *AO-dpasABCDE-dpchF*; (ii) *AO-dpasABCDE-dpchF*; (iii) *AO-dpasABCDEF-dpmpGH*; (iv) *AO-dpasACD-dpchBEGH-dpmaF*. The chromatograms were monitored at 280 nm. Non-marked peaks were not DDPs. We didn't characterize them, but they may be derived from polyketide **1**, see Supplementary Fig. 13 (ii).

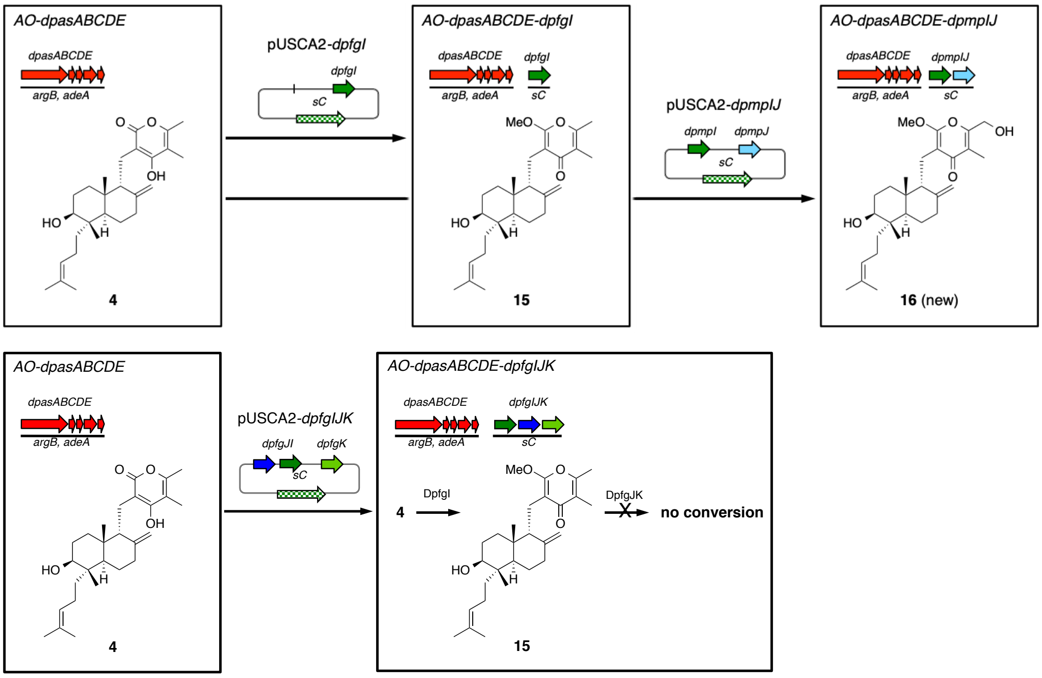

Supplementary Fig. 30. Reconstitution scheme of *AO-dpasABCDE-dpfgI*, *AO-dpasABCDE-dpmpIJ* and *AO-dpasABCDE-dpfgIJK*.

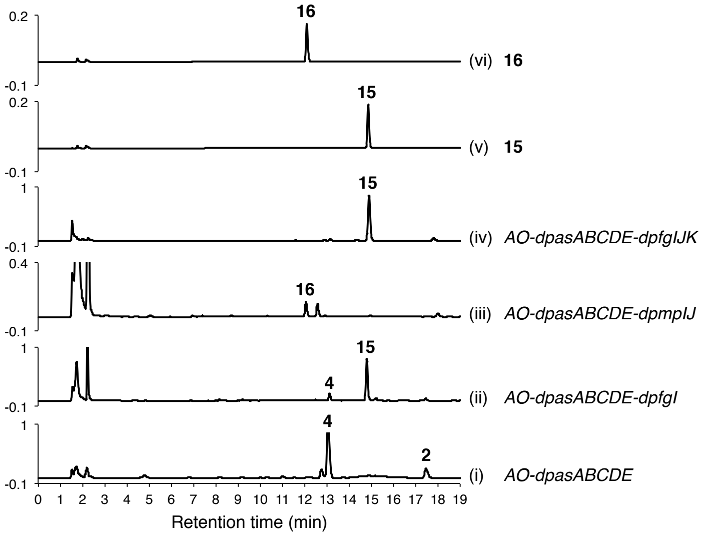

Supplementary Fig. 31. Reverse phase HPLC profiles of mycelia extracts from *A. oryzae* transformants. (i) *AO-dpasABCDE*; (ii) *AO-dpasABCDE-dpfgI*; (iii) *AO-dpasABCDE-dpmpIJ*; (iv) *AO-dpasABCDE-dpfgIJK*; (v) purified **15** (1 µg); (vi) purified **16** (1 µg). The chromatograms were monitored at 280 nm (lane i) and 254 nm (lane ii-vi). Non-marked peaks were not DDPs. We didn't characterize them, but they may be derived from polyketide **1**, see Supplementary Fig. 13 (ii).

Compound 15

UV

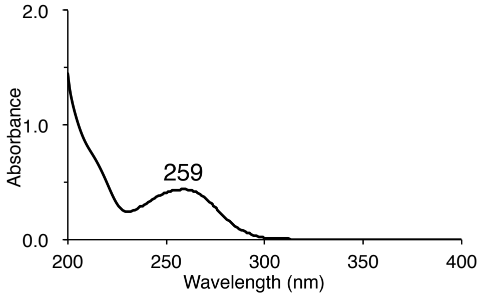

IR

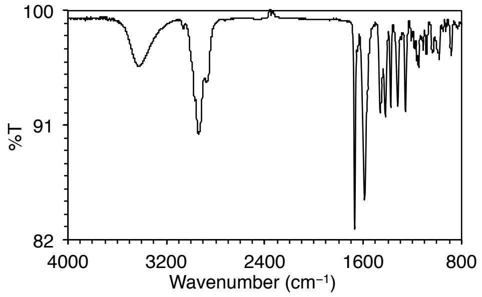

Compound 16

UV

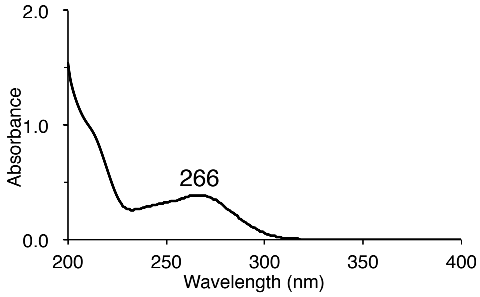

IR

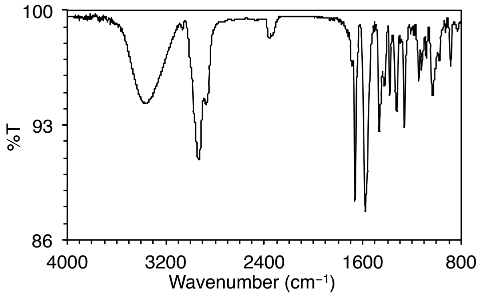

Supplementary Fig. 32. UV and IR spectra of **15** and **16**.

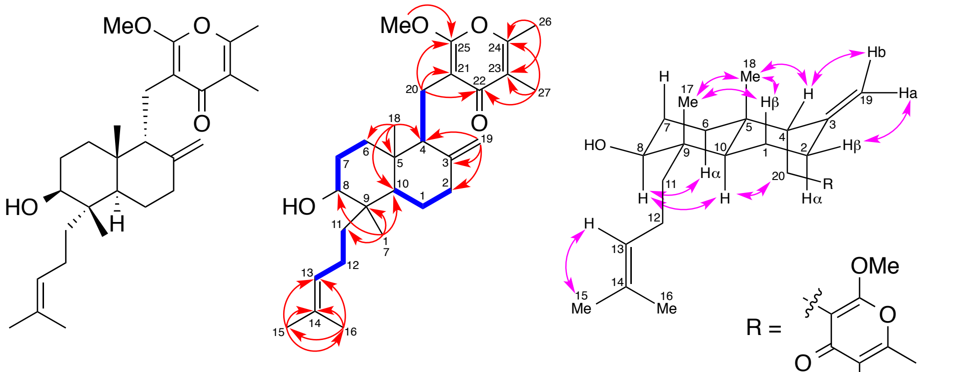

15

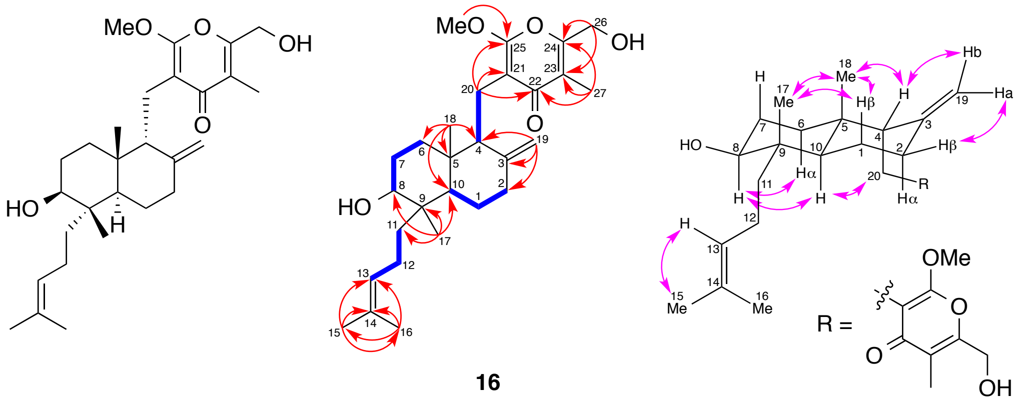

16

Supplementary Fig. 33. Key HMBC (red arrow), ^{1}H-^{1}H COSY (blue bold line) and NOE (purple arrow) correlations of **15** and **16**.

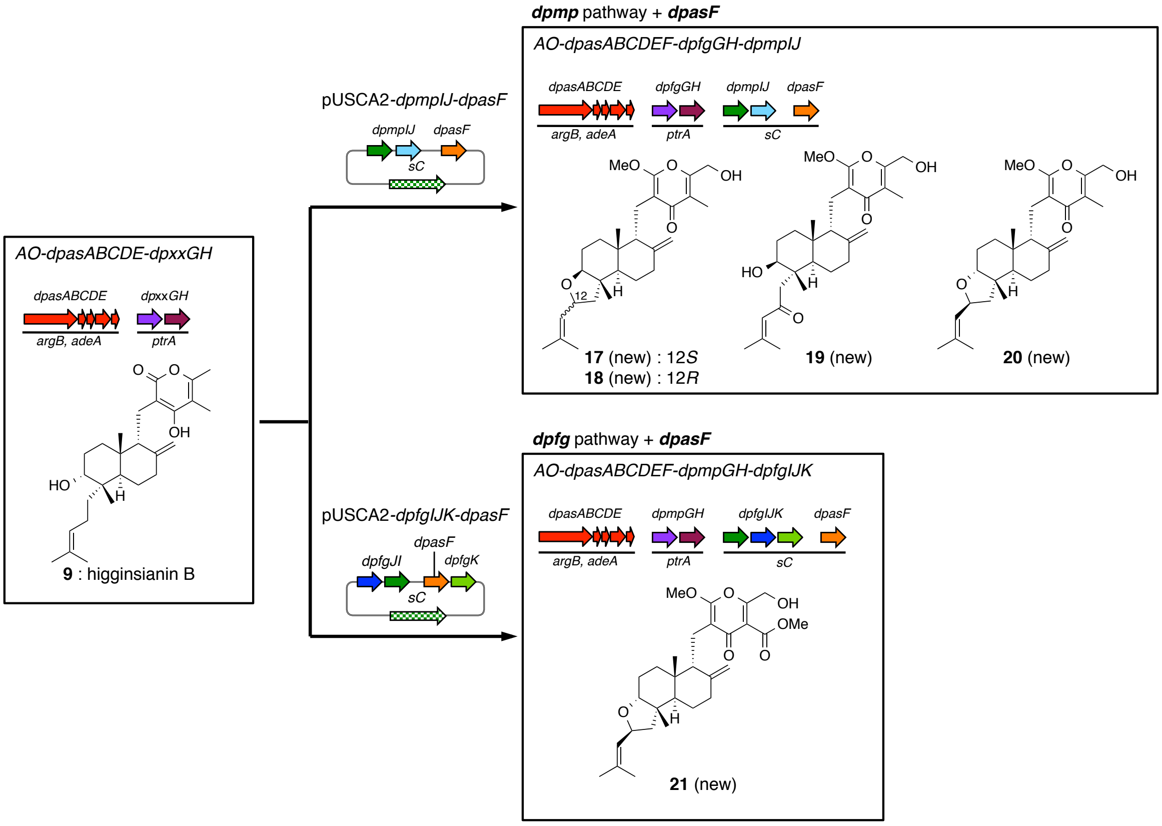

Supplementary Fig. 34. Construction of *dpmp* pathway + *dpasF* (*AO-dpasABCDEF-dpfgGH-dpmpIJ*) and *dpfg* pathway + *dpasF* (*AO-dpasABCDEF-dpmpGH-dpfgIJK*).

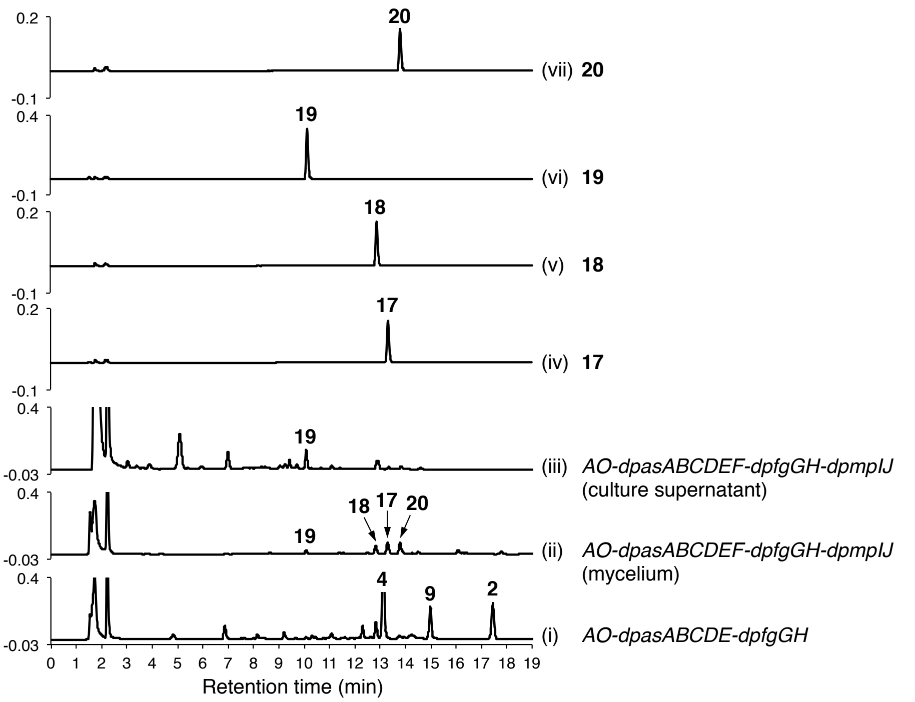

Supplementary Fig. 35. Reverse phase HPLC profiles of mycelia (lane i and ii) and culture supernatant (lane iii) extract from *A. oryzae* transformants. (i) *AO-dpasABCDE-dpfgGH*; (ii) *AO-dpasABCDEF-dpfgGH-dpmpIJ*; (iii) *AO-dpasABCDEF-dpfgGH-dpmpIJ*; (iv) purified **17** (1 μg); (v) purified **18** (1 μg); purified (vi) purified **19** (1 μg); (vii) purified **20** (1 μg). The chromatograms were monitored at 280 nm (lane i) and 254 nm (lane ii-vii). Non-marked peaks were not DDPs. We didn't characterize them, but they may be derived from polyketide **1**, see Supplementary Fig. 13 (ii).

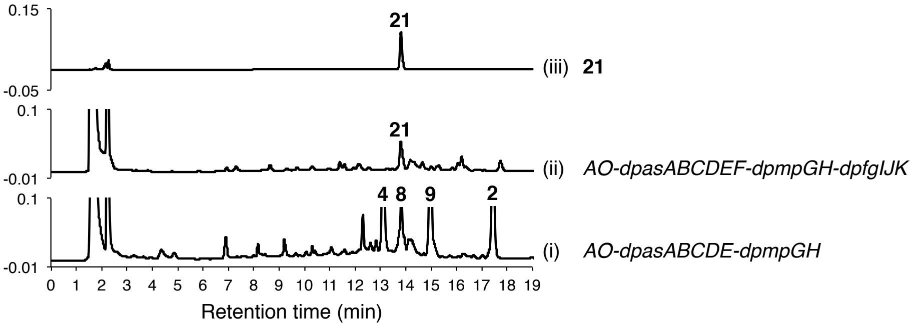

Supplementary Fig. 36. Reverse phase HPLC profiles of mycelia extracts from *A. oryzae* transformants. (i) *AO-dpasABCDE-dpmpGH*; (ii) *AO-dpasABCDEF-dpmpGH-dpfgIJK*, and purified compound (1 μg): (iii) **21**. The chromatograms were monitored at 280 nm (lane i) and 254 nm (lane ii and iii). Non-marked peaks were not DDPs. We didn't characterize them, but they may be derived from polyketide **1**, see Supplementary Fig. 13 (ii).

Compound 17

UV

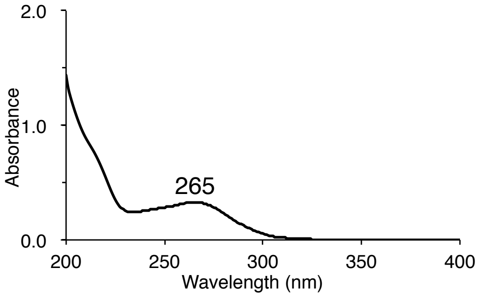

IR

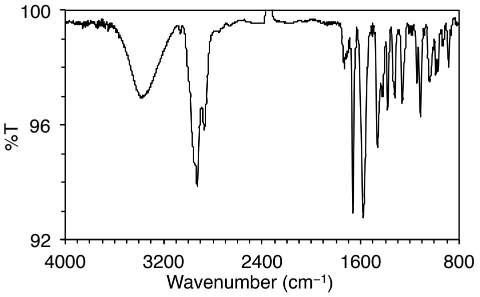

Compound 18

UV

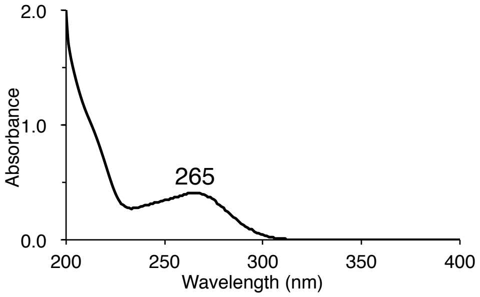

IR

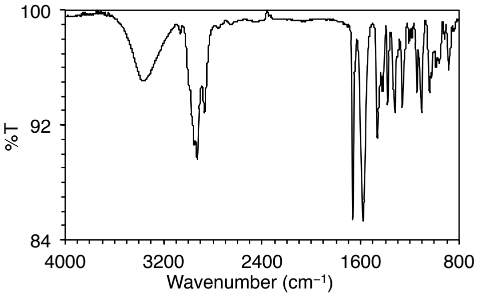

Compound 19

UV

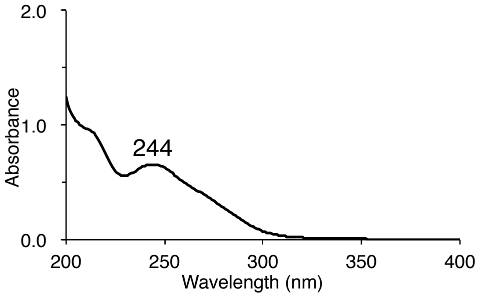

IR

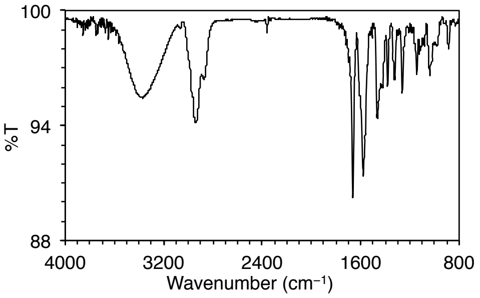

Compound 20

UV

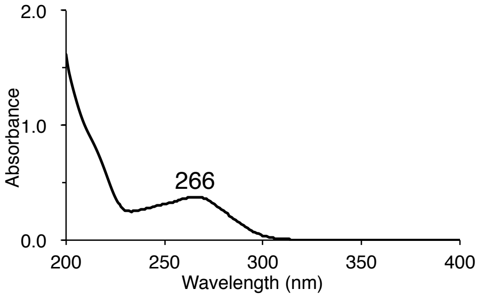

IR

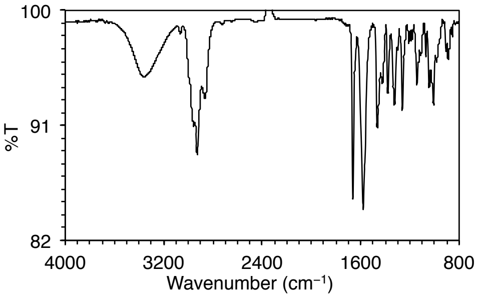

Compound 21

UV

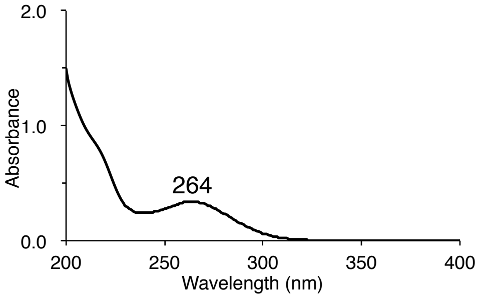

IR

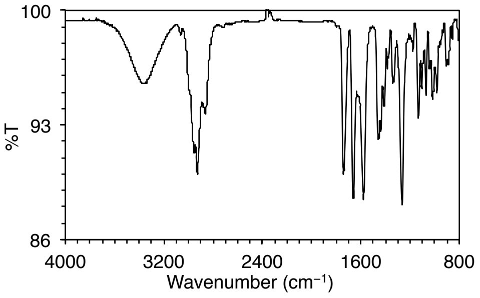

Supplementary Fig. 37. UV and IR spectra of **17**–**21**.

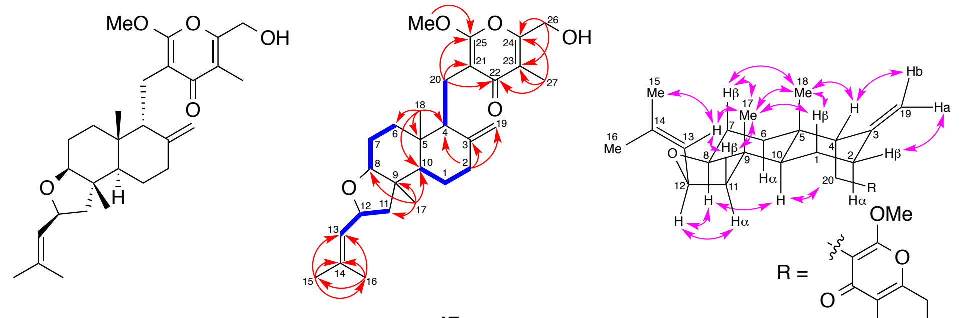

17

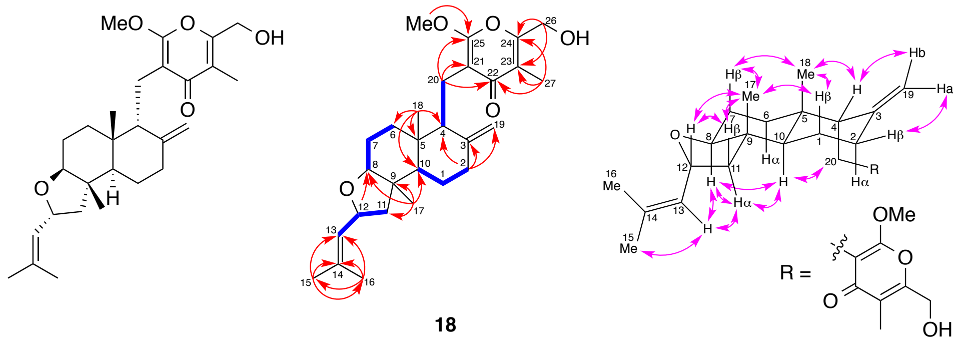

18

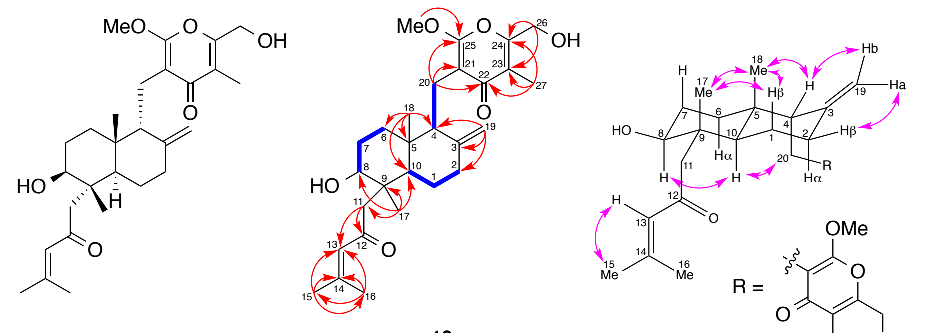

19

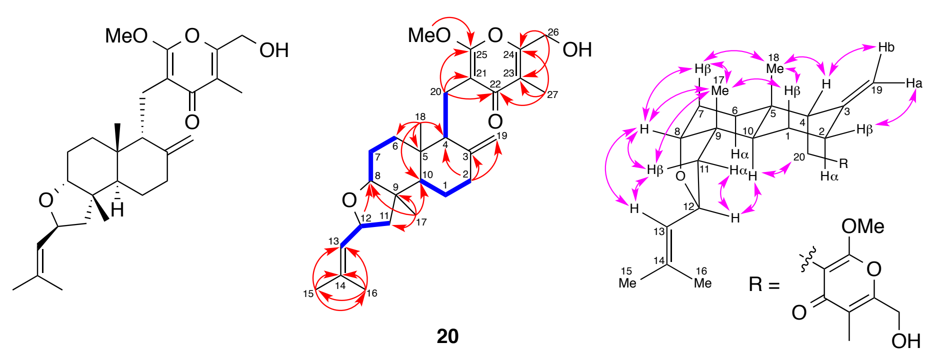

20

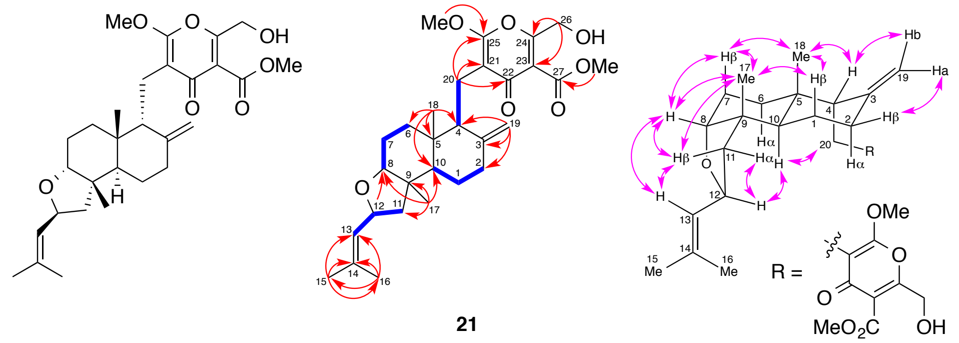

21

Supplementary Fig. 38. Key HMBC (red arrow), ^{1}H-^{1}H COSY (blue bold line) and NOE (purple arrow) correlation of **17–21**.

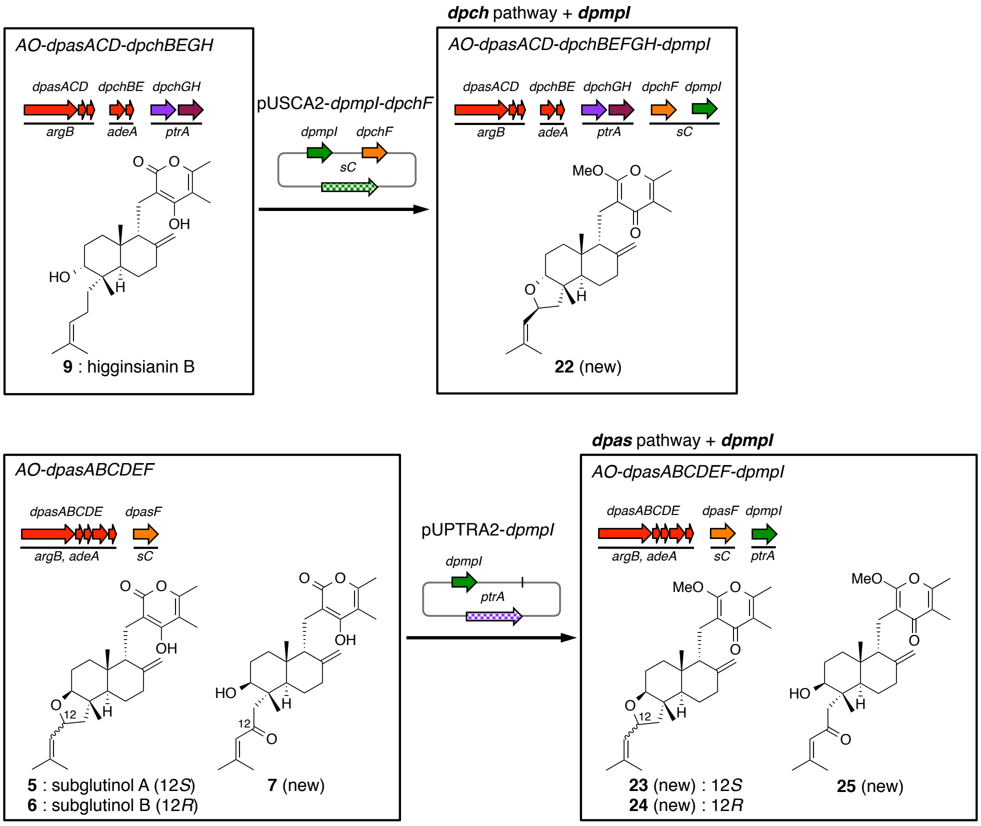

Supplementary Fig. 39. Construction of *dpch* pathway + *dpmpI* (*AO-dpasACD-dpchBEFGH-dpmpI*) and *dpas* pathway + *dpmpI* (*AO-dpasABCDEF-dpmpI*).

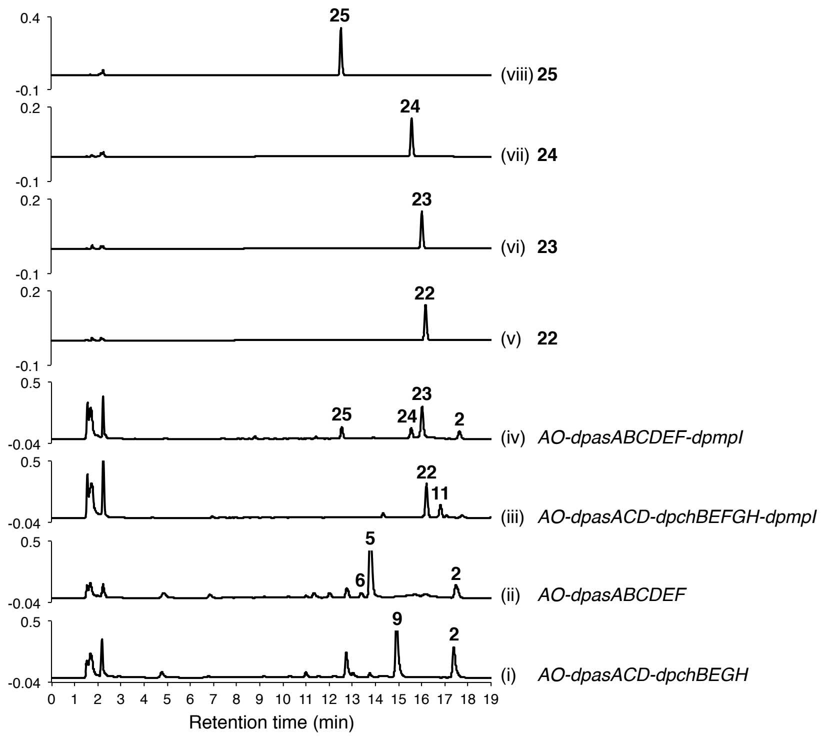

Supplementary Fig. 40. Reverse phase HPLC profiles of mycelia extracts from *A. oryzae* transformants. (i) *AO-dpasACD-dpchBEGH*; (ii) *AO-dpasABCDEF*; (iii) *AO-dpasACD-dpchBEFGH-dpmpI*; (iv) *AO-dpasABCDEF-dpmpI*; (v) purified **22** (1 μg); (vi) purified **23** (1 μg); (vii) purified **24** (1 μg); (viii) purified **25** (1 μg). The chromatograms were monitored at 280 nm (lane i and ii) and 254 nm (lane iii-viii). Non-marked peaks were not DDPs. We didn't characterize them, but they may be derived from polyketide **1**, see Supplementary Fig. 13 (ii).

Compound 22

UV

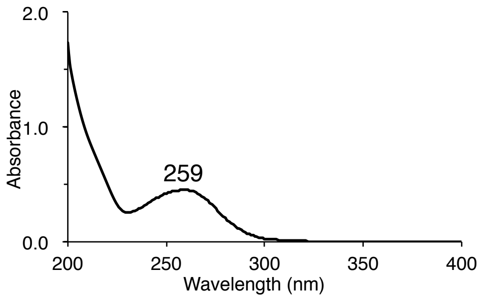

IR

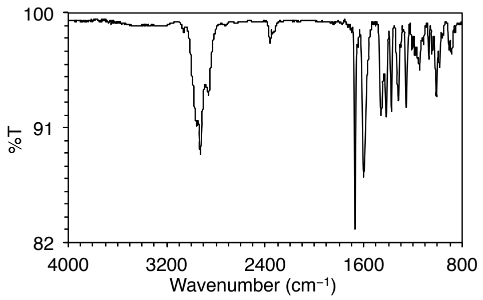

Compound 23

UV

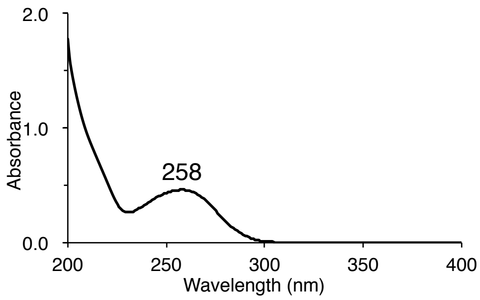

IR

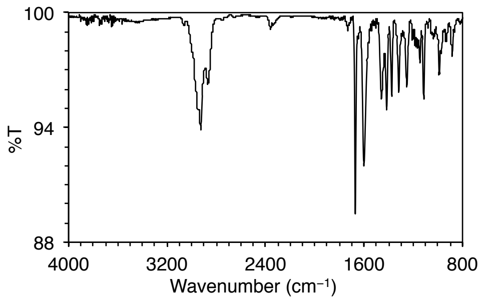

Compound 24

UV

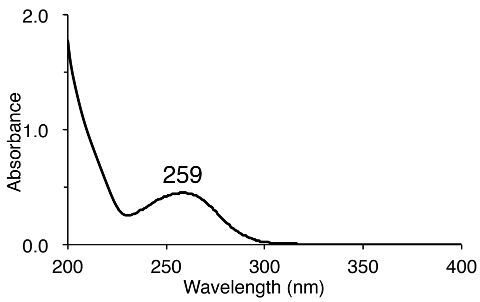

IR

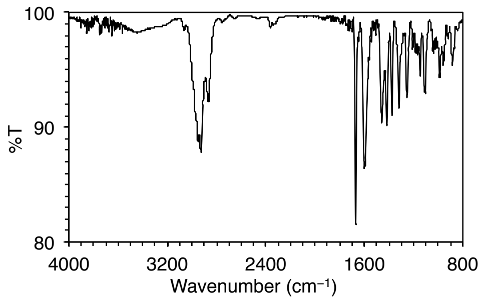

Compound 25

UV

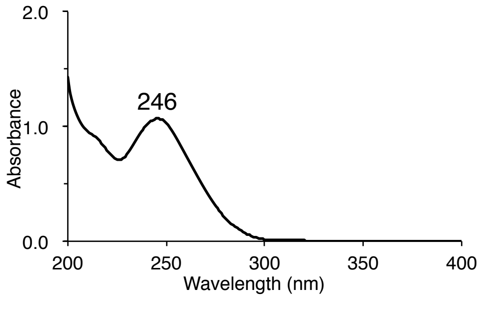

IR

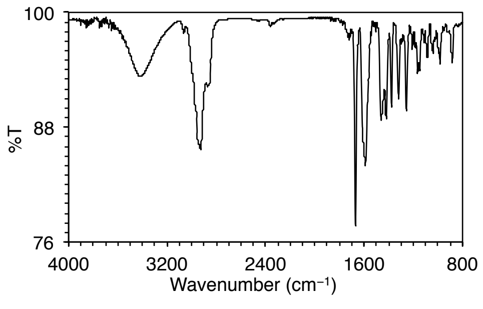

Supplementary Fig. 41. UV and IR spectra of **22**–**25**.

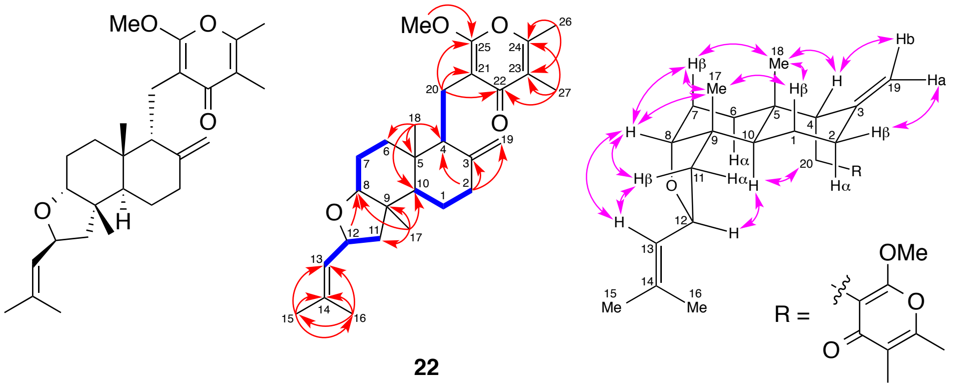

22

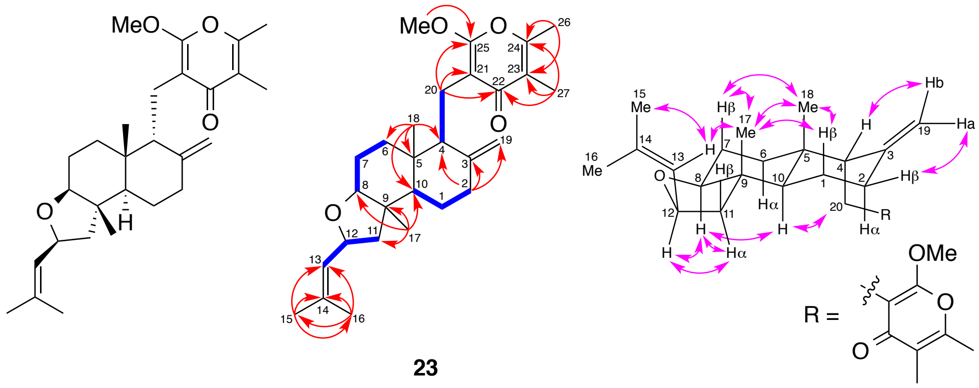

23

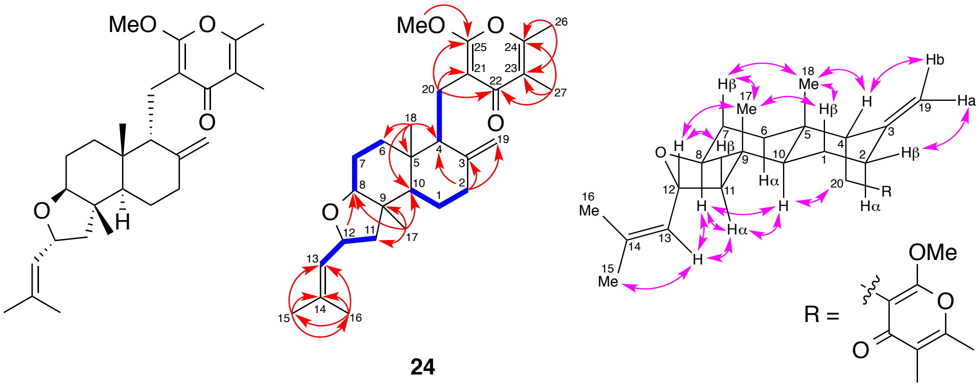

24

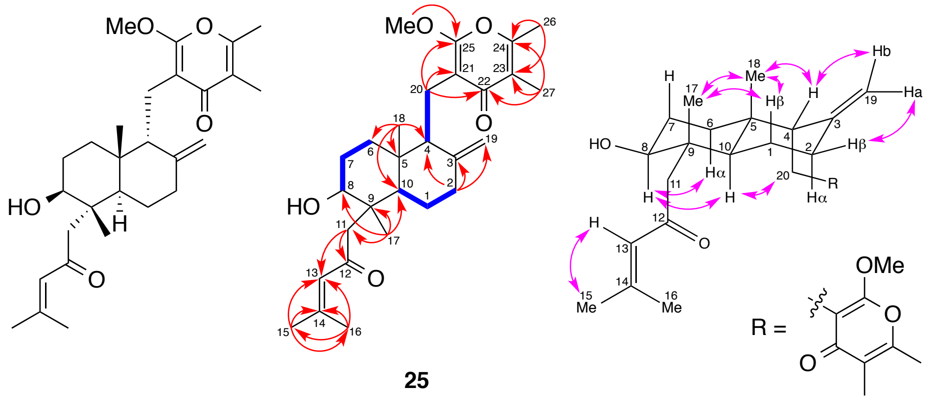

25

Supplementary Fig. 42. Key HMBC (red arrow), ^{1}H-^{1}H COSY (blue bold line) and NOE (purple arrow) correlations of **22**–**25**.

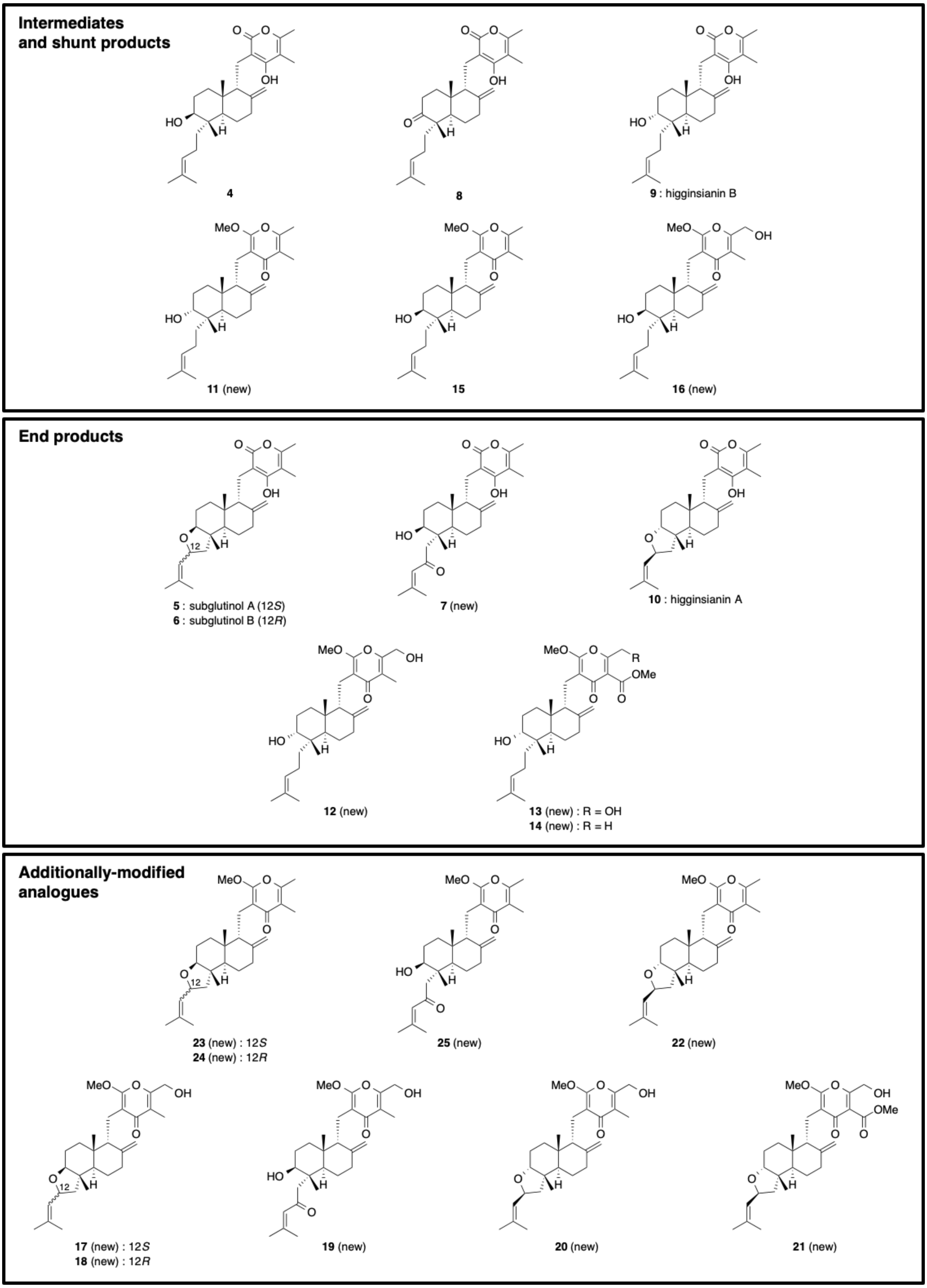

Supplementary Fig. 43. Structures of DDPs produced in this study.

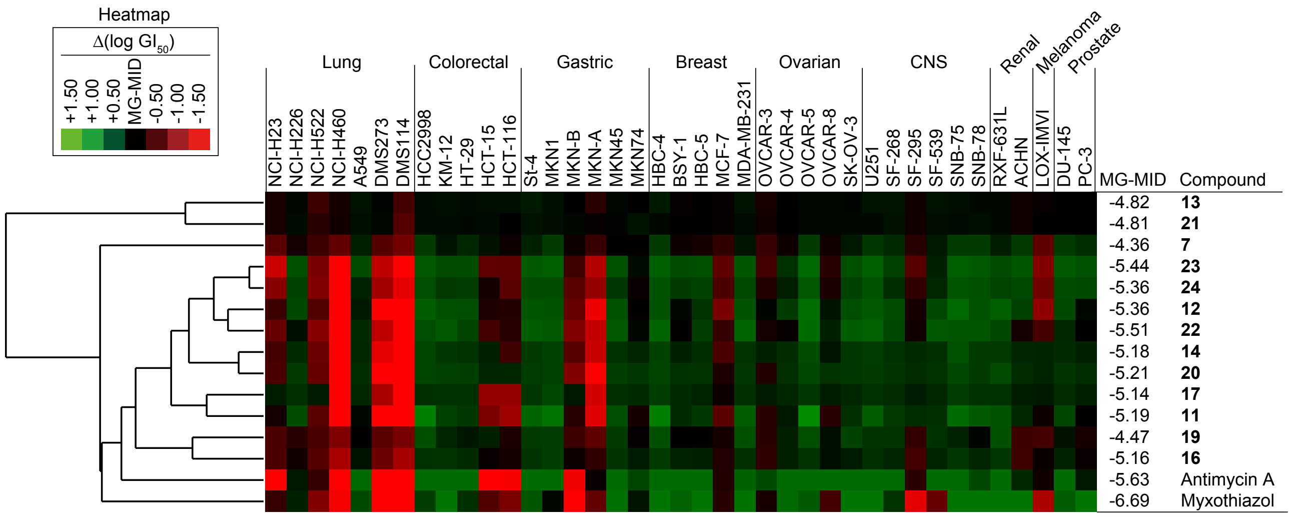

Supplementary Fig. 44. Hierarchical clustering of the antiproliferative fingerprints of novel DDPs across the JFCR39 cell lines and their relative antitumour activities visualized via a heatmap. The GI_{50} concentration (M) in each cell line was determined and log-transformed. MG-MID (mean graph midpoint), i.e., the mean log GI_{50} concentration across the JFCR39 panel, was indicated for each compound. Thirteen DDPs in conjunction with two mitochondrial respiratory complex III inhibitors (myxothiazol and antimycin A) were clustered on the basis of the correlations of their antitumour fingerprints with those of other compounds (average-linkage clustered with Pearson correlation metric). The heat map represents Δ(log GI_{50}), i.e., the difference in log GI_{50} concentration in each cell line from the MG-MID. Black represents the MG-MID for each compound across the panel. Red and green represent sensitivity and resistance, respectively, by approx. 31.6-fold (10^1.5) compared to the MG-MID values. Cluster analysis and visualization by heatmap was performed by using Cluster3.0/TreeView software (Stanford University). Source data are provided in a Source Data file.

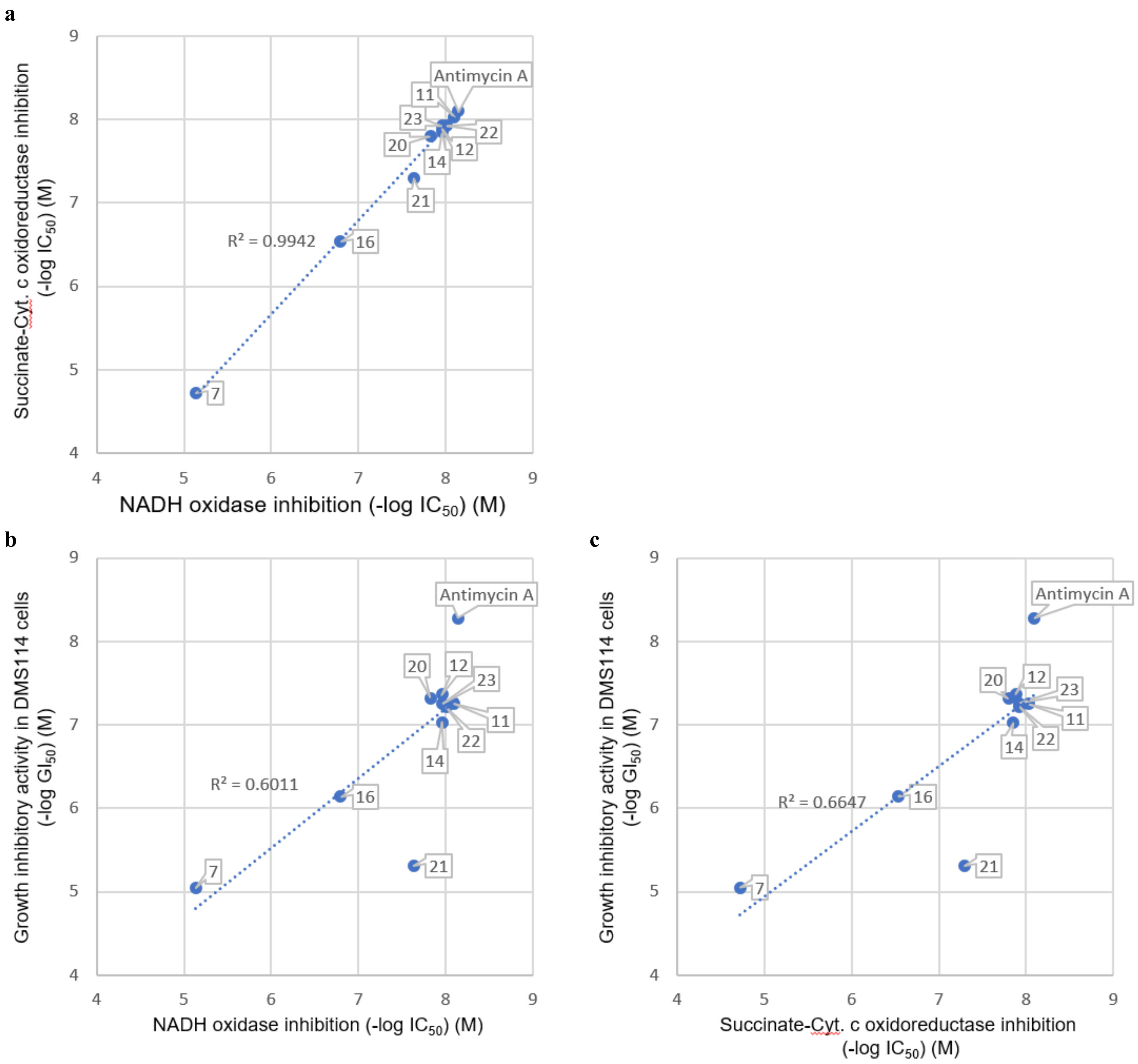

Supplementary Fig. 45. Comparison between mitochondrial respiratory complex III inhibition and cell growth inhibition on complex III inhibitor-sensitive lung cancer DMS114 cells. **a**, Correlation between half inhibitory concentrations (IC_{50}s) of the 10 compounds including nine DDPs and antimycin A required to inhibit NADH oxidase and those to inhibit succinate-cytochrome c oxidoreductase. **b**, Correlation between IC_{50}s of the 10 compounds to inhibit NADH oxidase and the half growth inhibitory concentrations (GI_{50}s) in human lung cancer DMS114 cells. **c**, Correlation between IC_{50}s of the 10 compounds to inhibit succinate-cytochrome c oxidoreductase and the GI_{50}s in human lung cancer DMS114 cells.

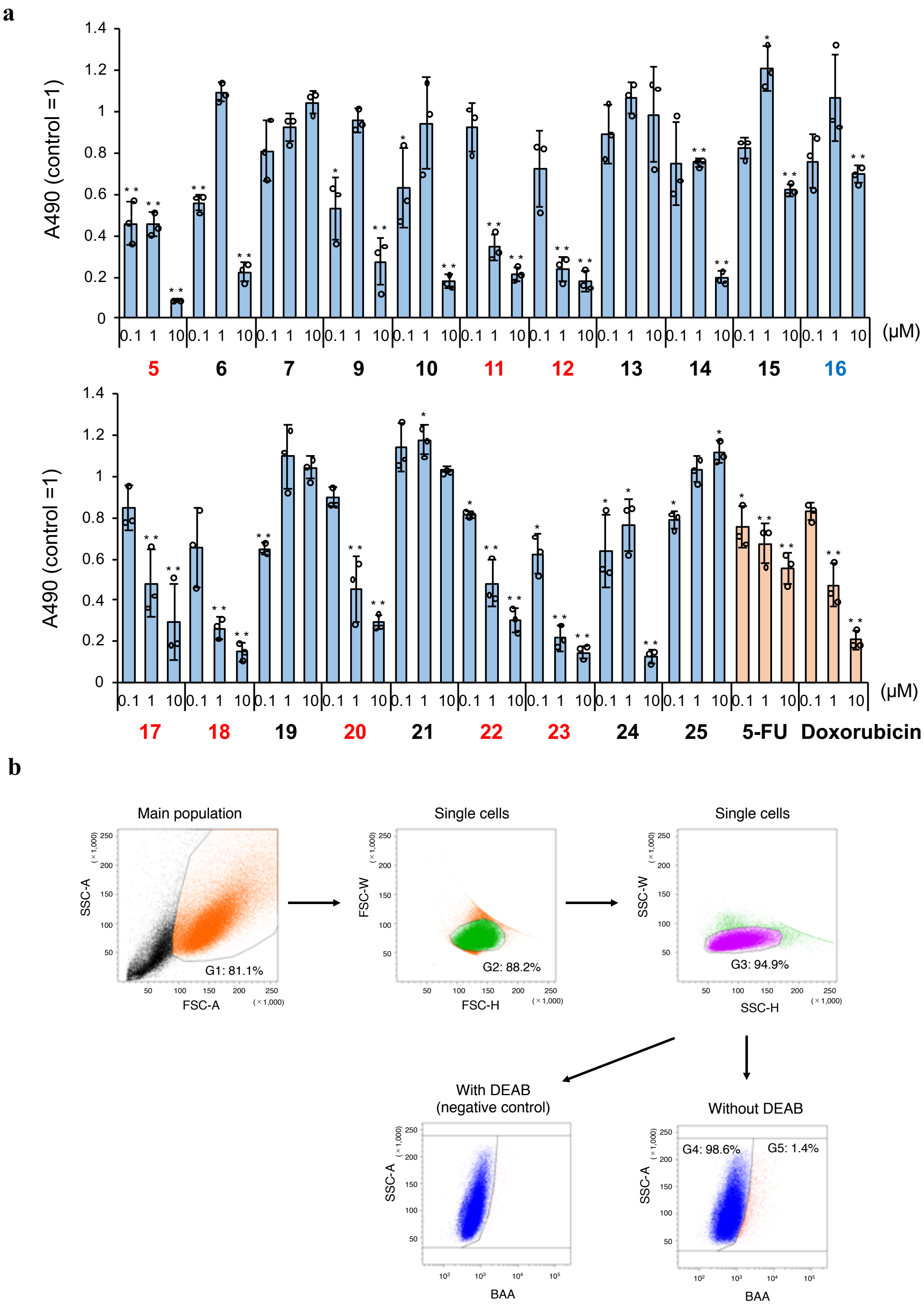

Supplementary Fig. 46. a, Cell cytotoxic effects of DDPs against MCF-7 cells (72 h). Compounds in red that showed dose dependent cytotoxic effects against MCF-7 cells were tested their inhibitory activity against mammosphere formation. Compound in blue (**16**) was also performed mammosphere assay as negative control. Data represent mean±s.d. from three independent replicate experiments. *P*-values are calculated using a two-sided unpaired Student's t-test; * $p < 0.05$, ** $p < 0.01$, compared with control. **b**, Gating strategy of flow cytometry analysis for detecting ALDH-positive cells. All samples were analyzed by sequential gating including main population (G1), single cells (G2, G3). Negative control with DEAB was used to establish G4 (ALDH-negative cells) and G5 (ALDH-positive cells). Source data underlying Supplementary Figure 46a are provided in a Source Data file.

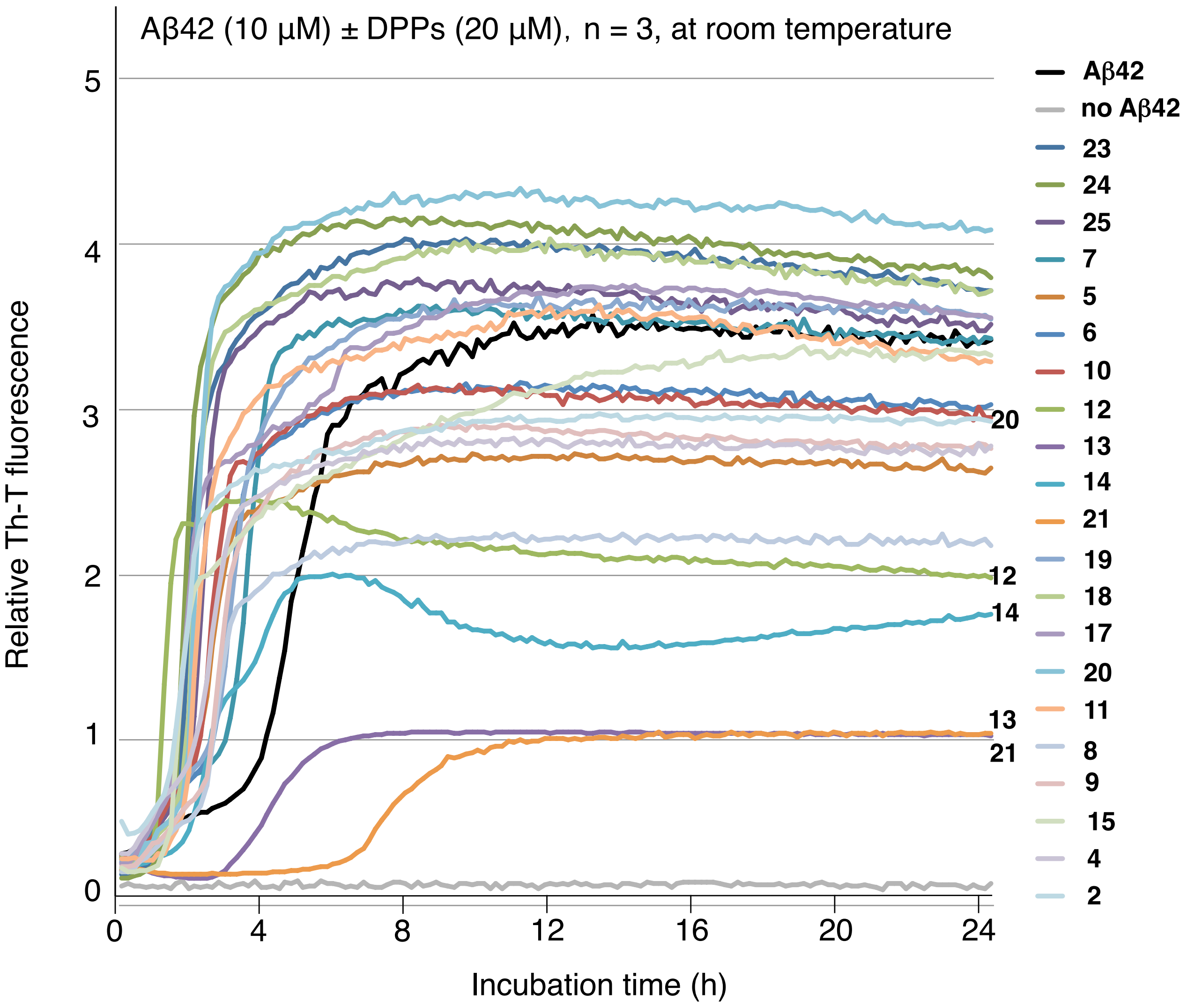

Supplementary Fig. 47. First Th-T assay of DDPs. Compounds **13** and **21** clearly delayed nuclei formation of Aβ42. Compounds **12** and **14** suppressed Aβ42 aggregation, although they did not delay the nuclei formation. Compound **20** slightly suppressed Aβ42 aggregation. Thus, we picked up the five DDPs and performed further investigation. Source data are provided in a Source Data file.

a

【Total ion chromatogram】

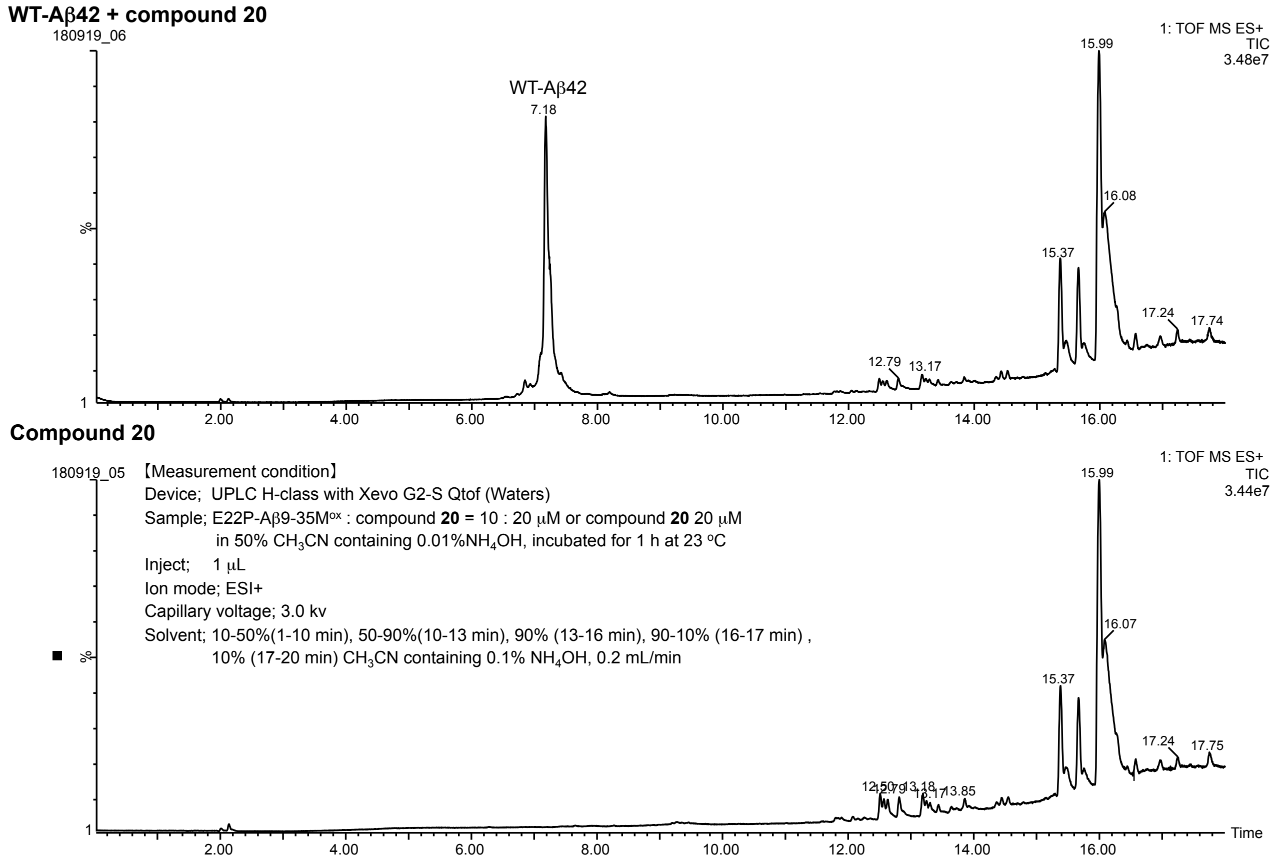

b

【Total ion chromatogram】

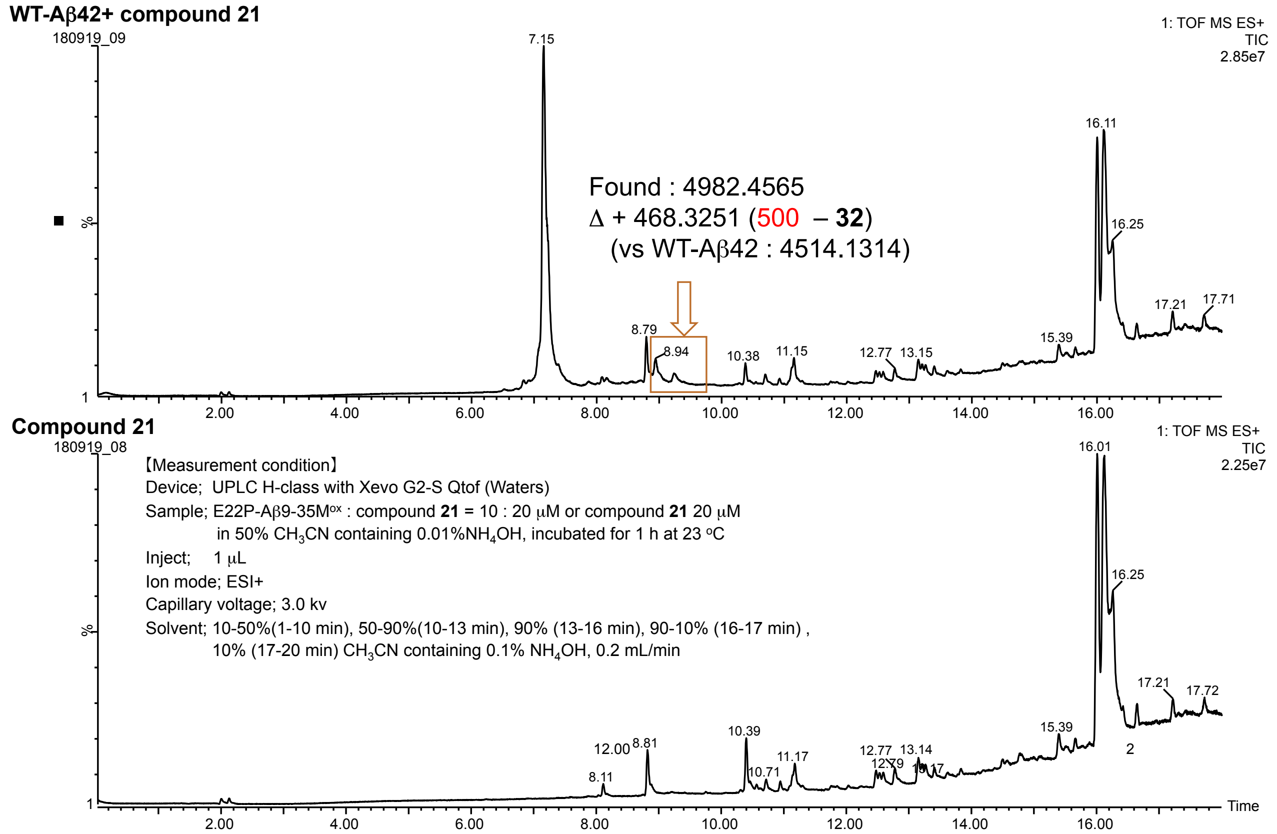

c

【Raw data】

WT-Aβ42 + compound 21 Mass spectrum

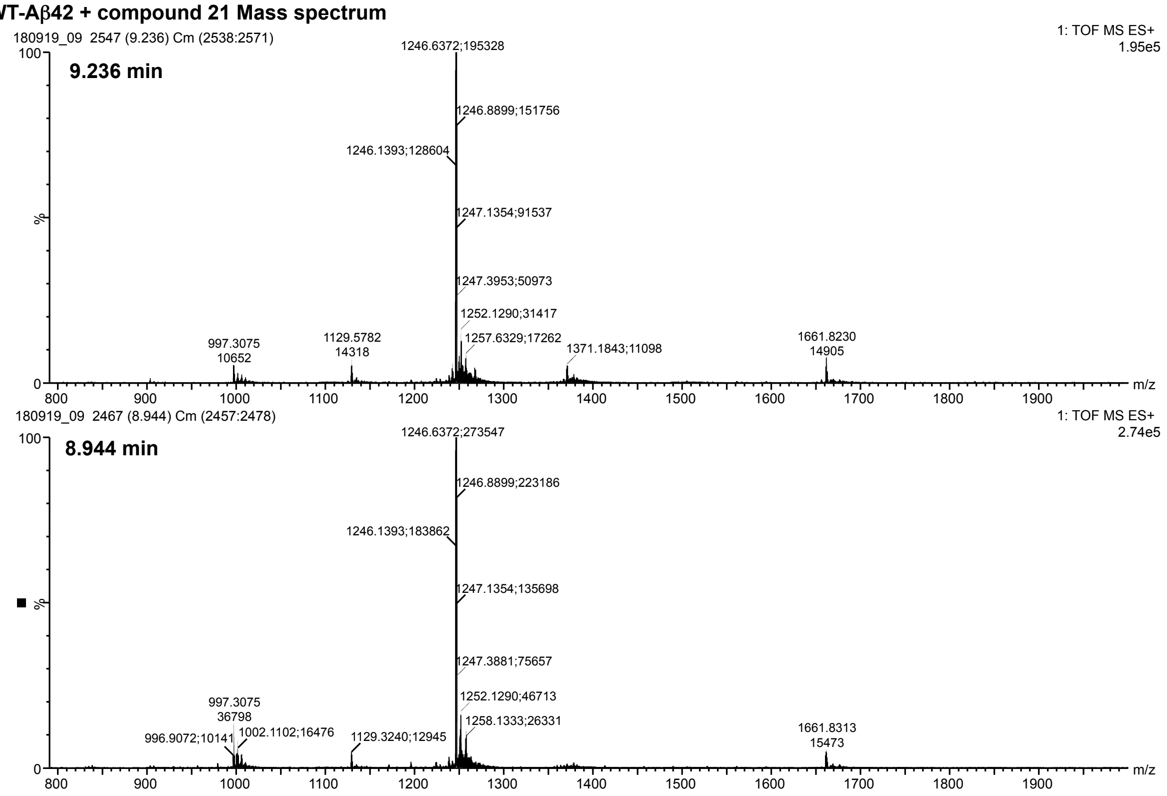

d

【Raw data】

WT-Aβ42 + compound 21 Deconvolution Mass spectrum

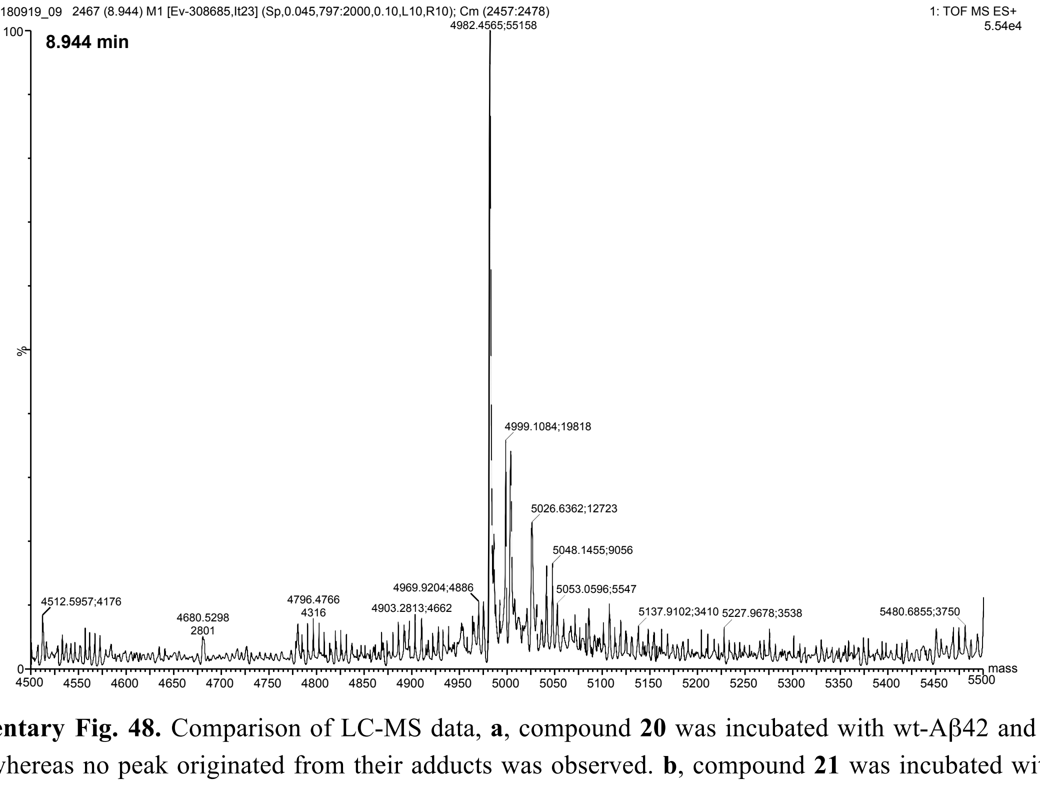

Supplementary Fig. 48. Comparison of LC-MS data, **a**, compound **20** was incubated with wt-Aβ42 and analyzed by LC-MS, whereas no peak originated from their adducts was observed. **b**, compound **21** was incubated with Aβ42 and analyzed by LC-MS, and their Michael adducts were observed around 9 min. **c**, ESI mass spectra of the Michael adducts between Aβ42 and compound **21**. **d**, Deconvolution Mass spectrum of a Michael adduct observed at 8.944 min.

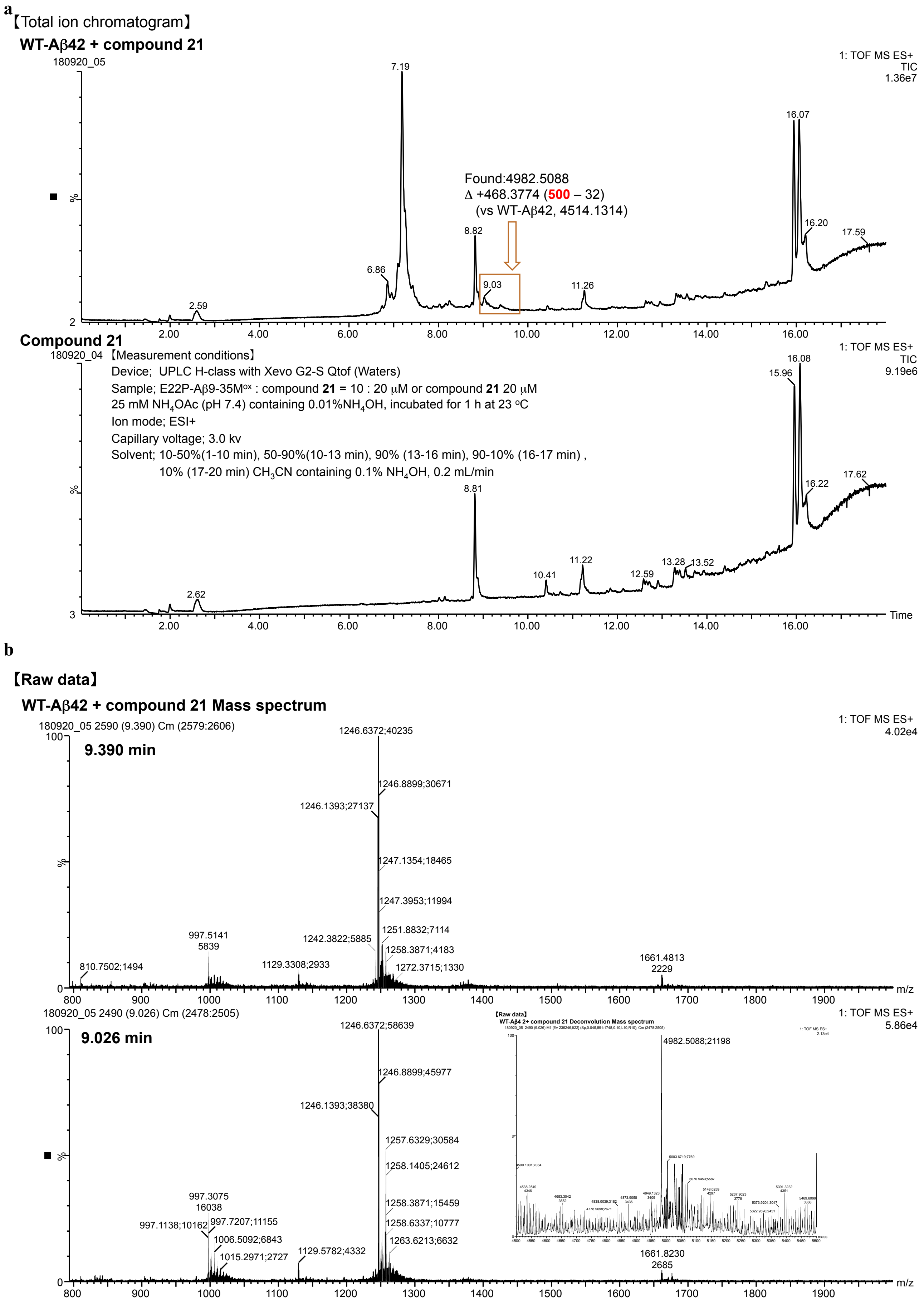

Supplementary Fig. 49. LC-MS analysis of the reaction mixture of wt-Aβ42 and compound **21** in ammonium acetate buffer, and the Michael adducts were observed in the condition.

a

【Total ion chromatogram】

E22P-Aβ9-35(Mox) + compound 21

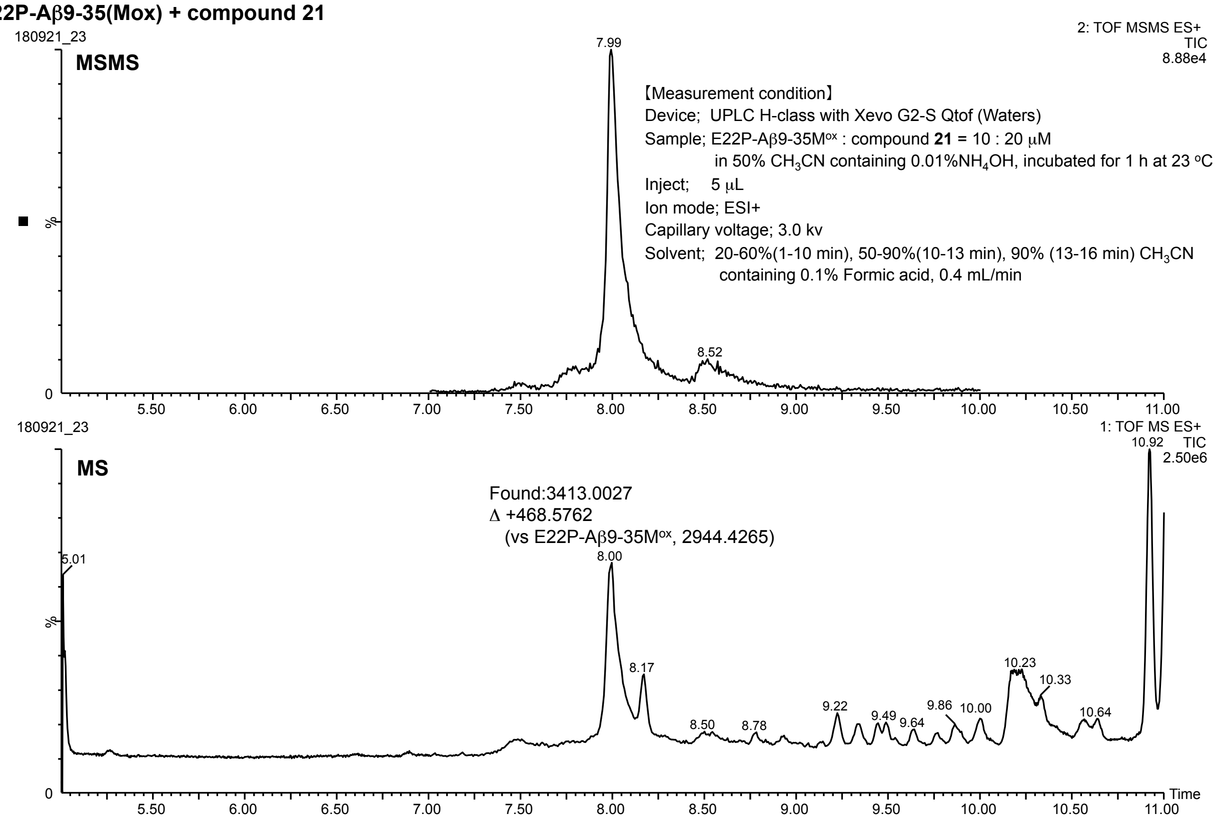

b

【Raw data】

E22P-Aβ9-35(Mox) + compound 21 MS spectra

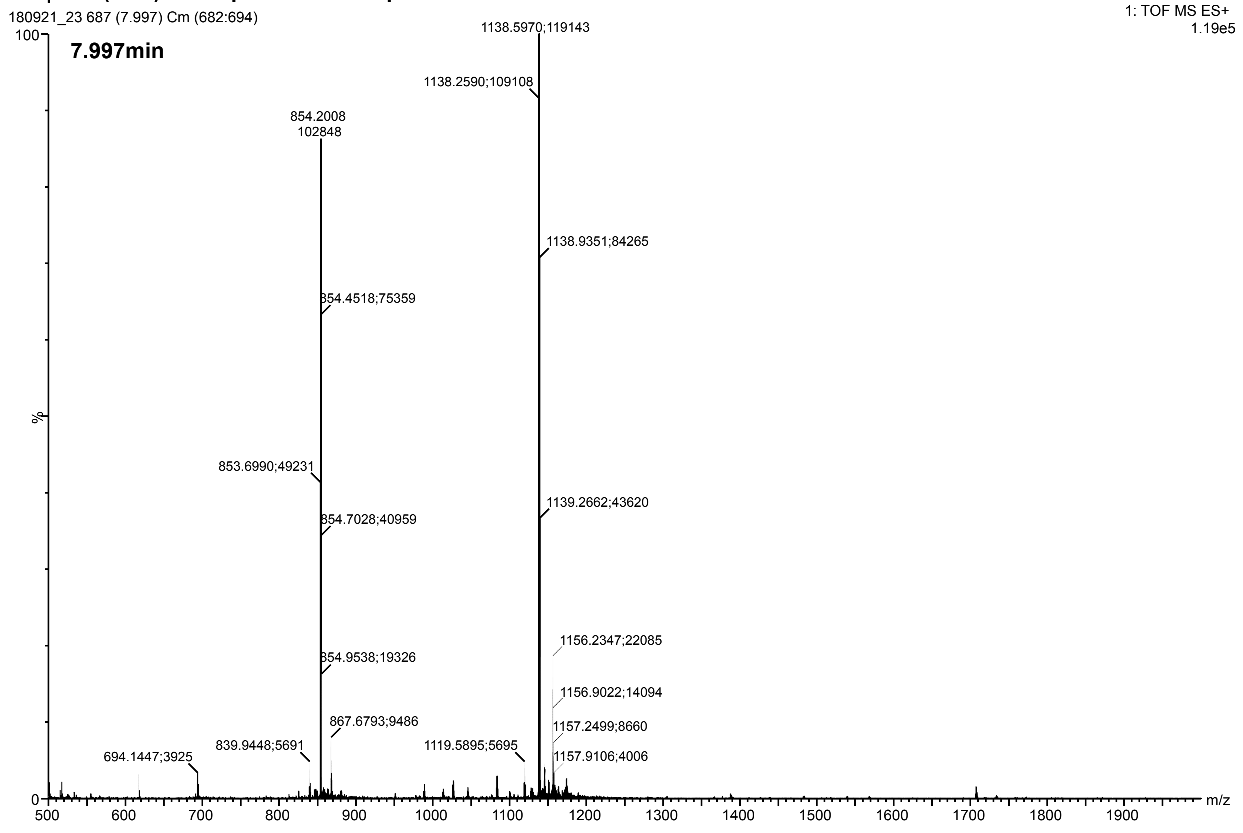

c

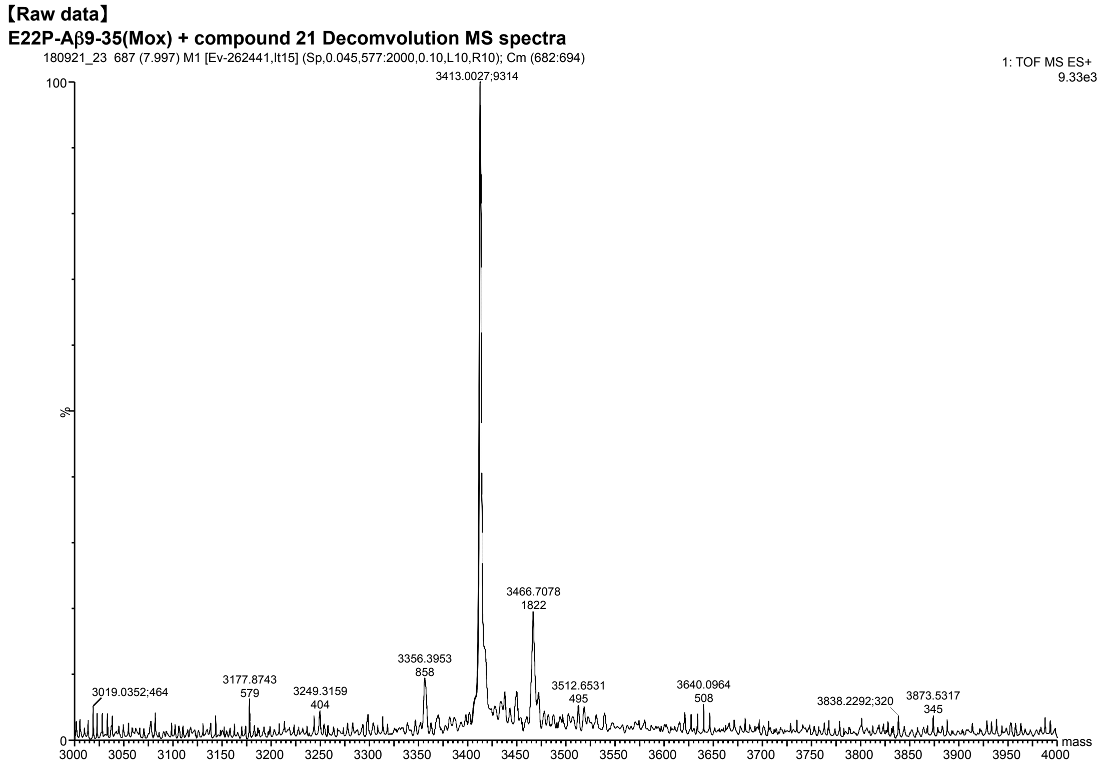

d

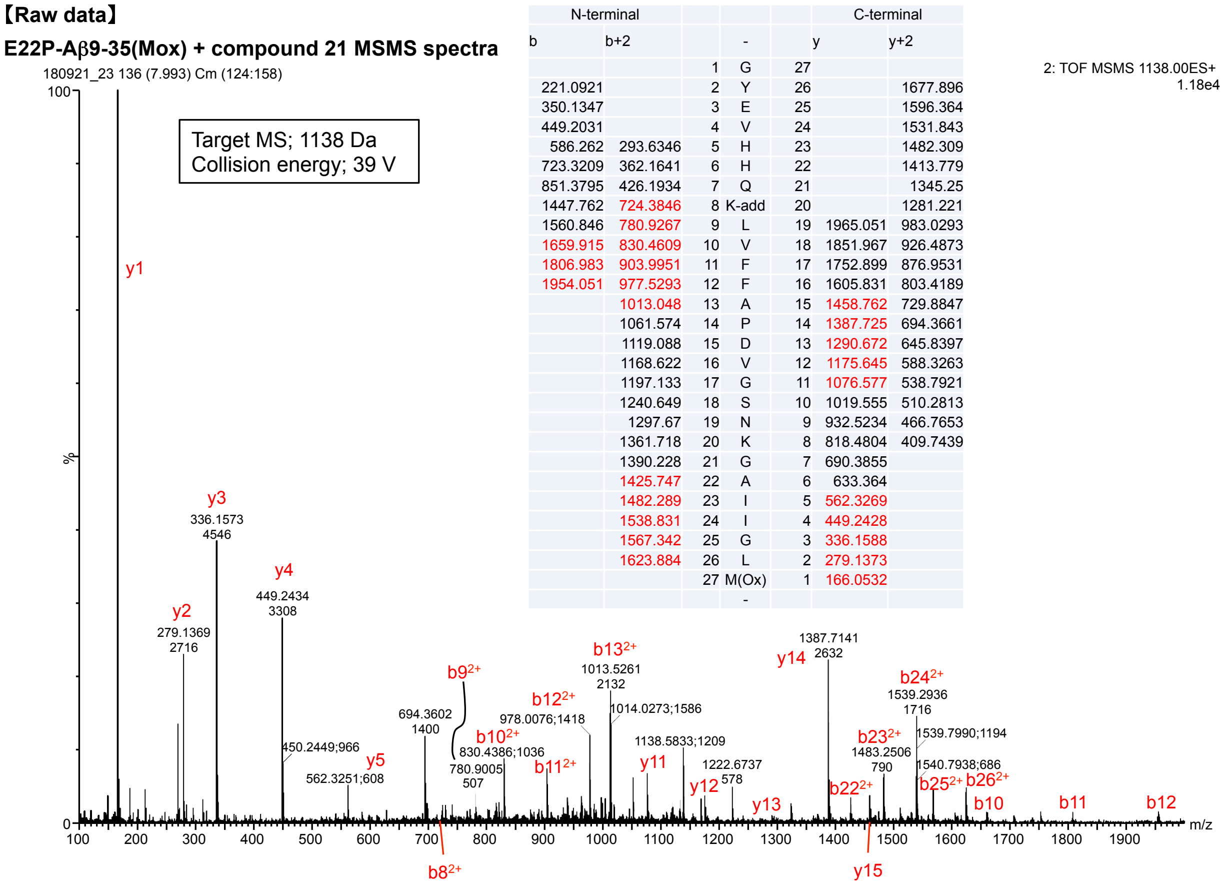

b	b+2		N-terminal / C-terminal		y	y+2
		1	G	27		
221.0921		2	Y	26		1677.896
350.1347		3	E	25		1596.364
449.2031		4	V	24		1531.843
586.262	293.6346	5	H	23		1482.309
723.3209	362.1641	6	H	22		1413.779
851.3795	426.1934	7	Q	21		1345.25
1447.762	724.3846	8	K-add	20		1281.221
1560.846	780.9267	9	L	19	1965.051	983.0293
1659.915	830.4609	10	V	18	1851.967	926.4873
1806.983	903.9951	11	F	17	1752.899	876.9531
1954.051	977.5293	12	F	16	1605.831	803.4189
	1013.048	13	A	15	1458.762	729.8847
	1061.574	14	P	14	1387.725	694.3661
	1119.088	15	D	13	1290.672	645.8397
	1168.622	16	V	12	1175.645	588.3263
	1197.133	17	G	11	1076.577	538.7921
	1240.649	18	S	10	1019.555	510.2813
	1297.67	19	N	9	932.5234	466.7653
	1361.718	20	K	8	818.4804	409.7439
	1390.228	21	G	7	690.3855	
	1425.747	22	A	6	633.364	
	1482.289	23	I	5	562.3269	
	1538.831	24	I	4	449.2428	
	1567.342	25	G	3	336.1588	
	1623.884	26	L	2	279.1373	
		27	M(Ox)	1	166.0532	
			-			

Supplementary Fig. 50. LC-MS/MS analyses of the adduct of E22P, M35M$_{ox}$-Aβ9-35 and compound **21**, **a**, Total ion chromatogram of LC-MS and LC-MS/MS. **b**, LC-MS data of the adduct of E22P, M35M$_{ox}$-Aβ9-35 and compound **21**. **c**, Deconvolution Mass spectrum of the adduct of E22P, M35M$_{ox}$-Aβ9-35 and compound **21**. **d**, MSMS spectra of the adduct of E22P, M35M$_{ox}$-Aβ9-35 and compound **21** showed **21** added at Lys16 in wt-Aβ42.

a

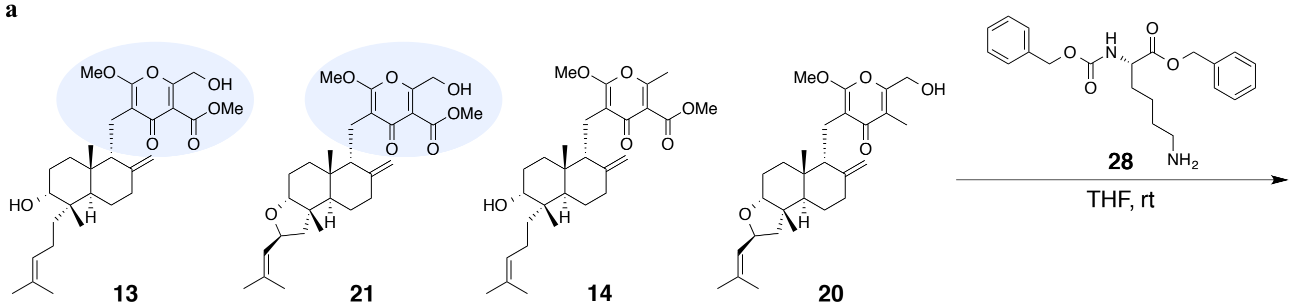

b

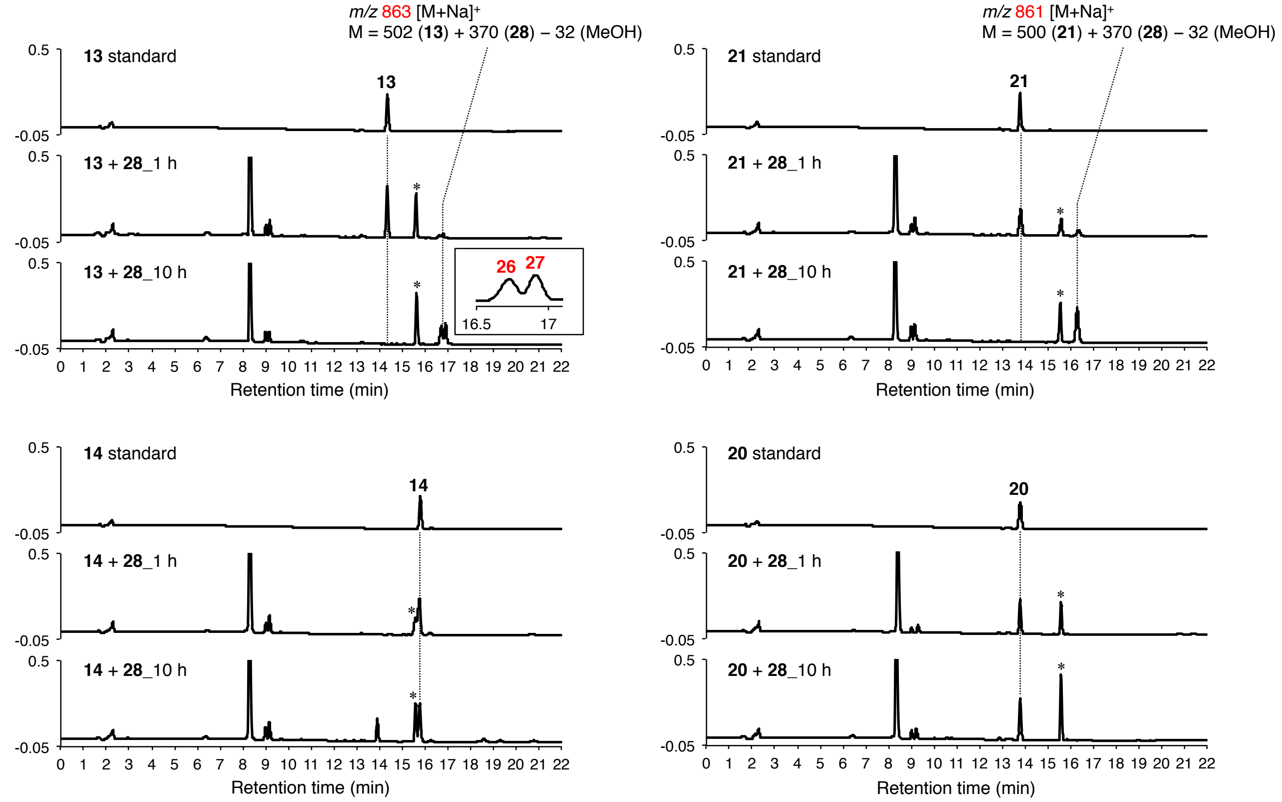

Supplementary Fig. 51. Reaction between lysine derivative **28** and DDPs (**13**, **14**, **20**, **21**). **a**, DDPs were reacted with **28** in THF at r.t. **b**, Monitoring of the reaction by HPLC analysis (detected by 215 nm). * stabilizer in THF.

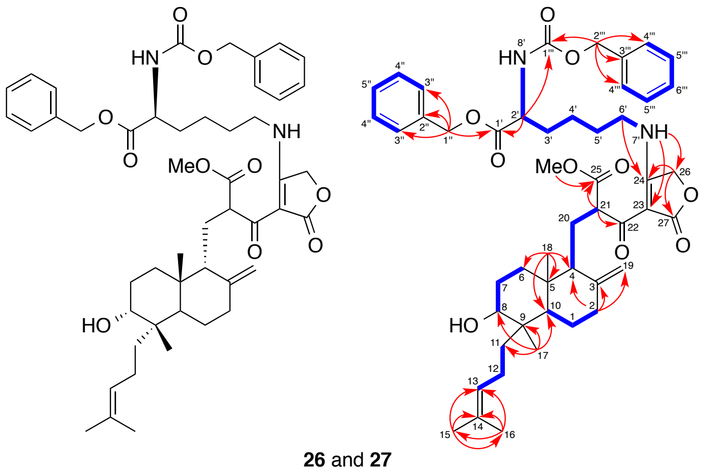

Supplementary Fig. 52. Key HMBC (red arrow) and ^{1}H-^{1}H COSY (blue bold line) correlations of **26** and **27**.

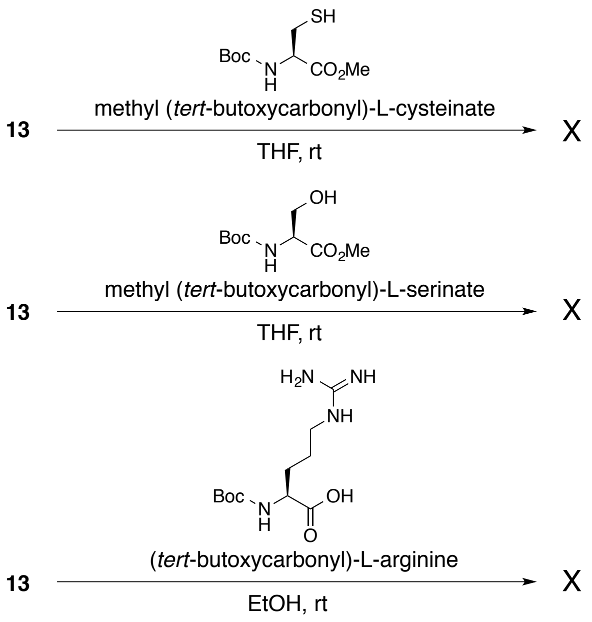

Supplementary Fig. 53. Reaction of **13** with methyl (tert-butoxycarbonyl)-L-cysteinate, methyl (tert-butoxycarbonyl)-L-serinate, or *N*α-(tert-butoxycarbonyl)-L-arginine.

^{1}H NMR (500 MHz, $CDCl_3$ (10% CD_3OD)) spectrum of **4**

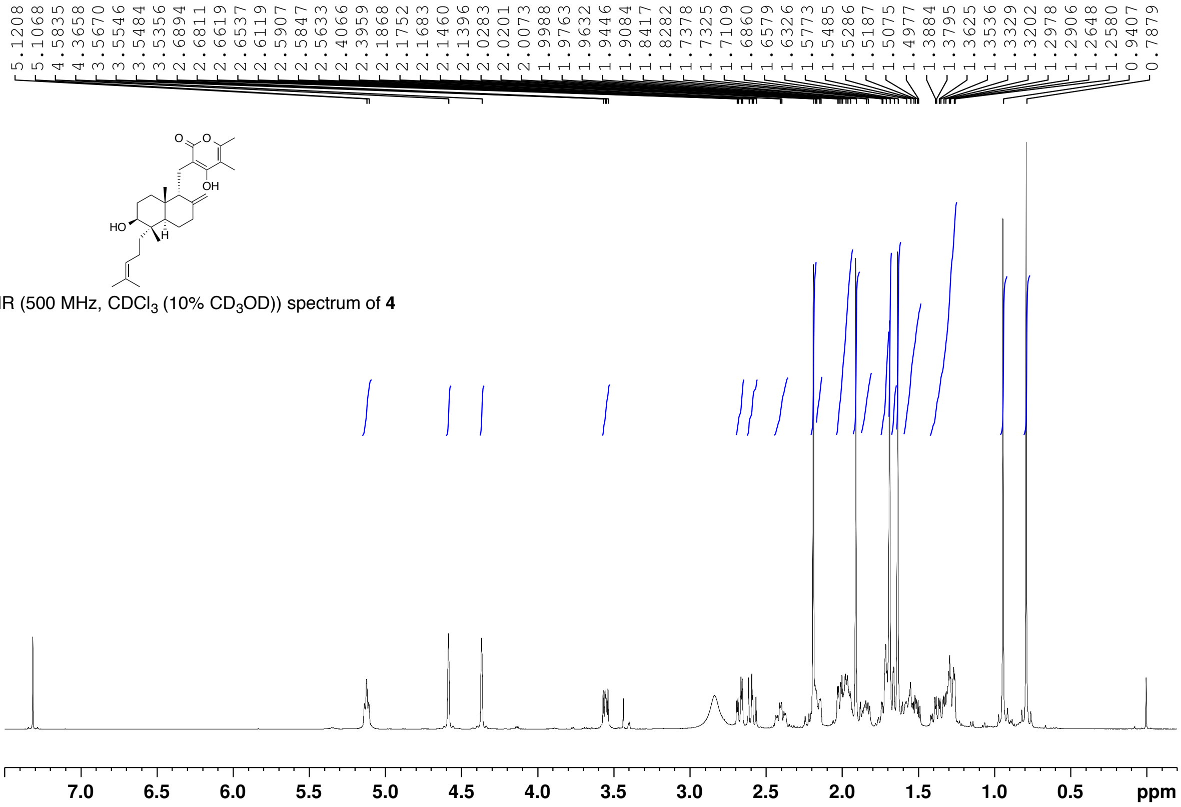

^{13}C NMR (125 MHz, $CDCl_3$ (10% CD_3OD)) spectrum of **4**

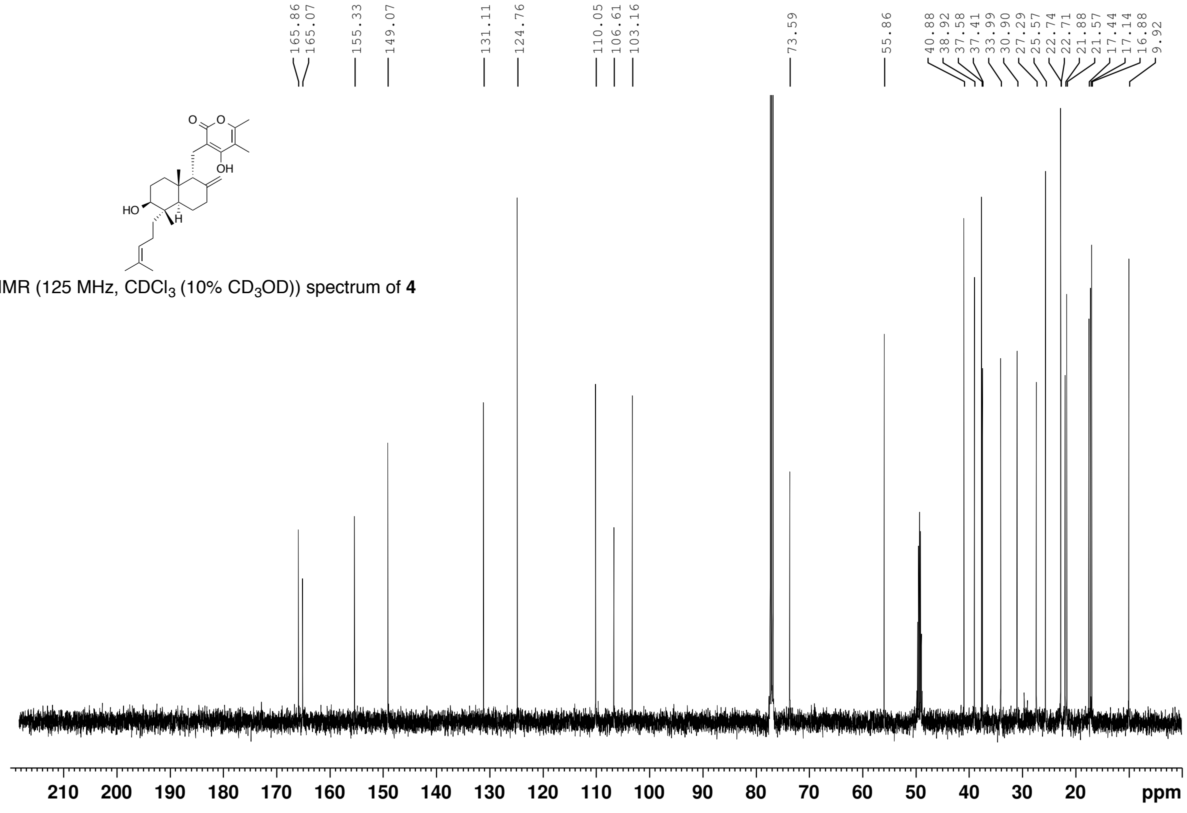

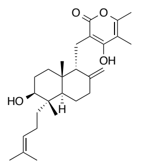

^1H-^1H COSY ($CDCl_3$ (10% CD_3OD)) spectrum of **4**

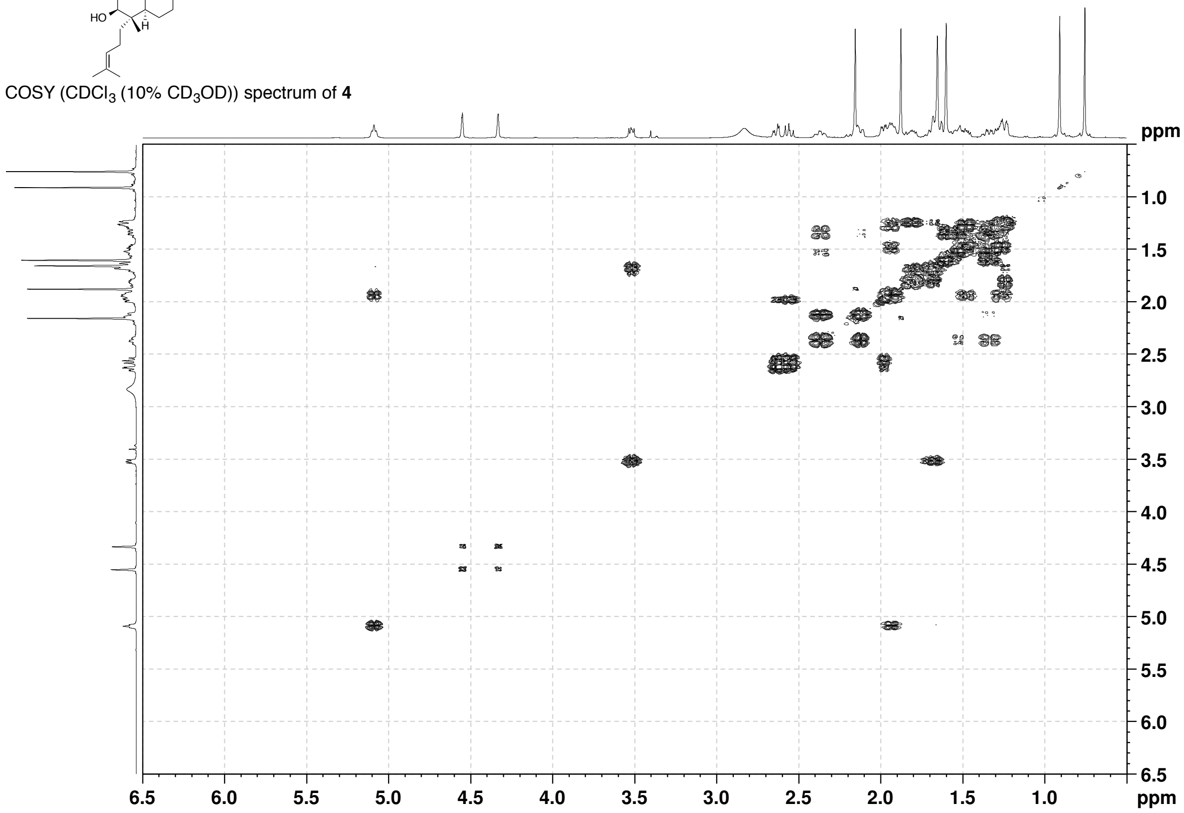

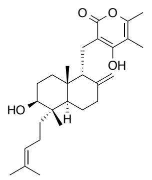

HMBC ($CDCl_3$ (10% CD_3OD)) spectrum of **4**

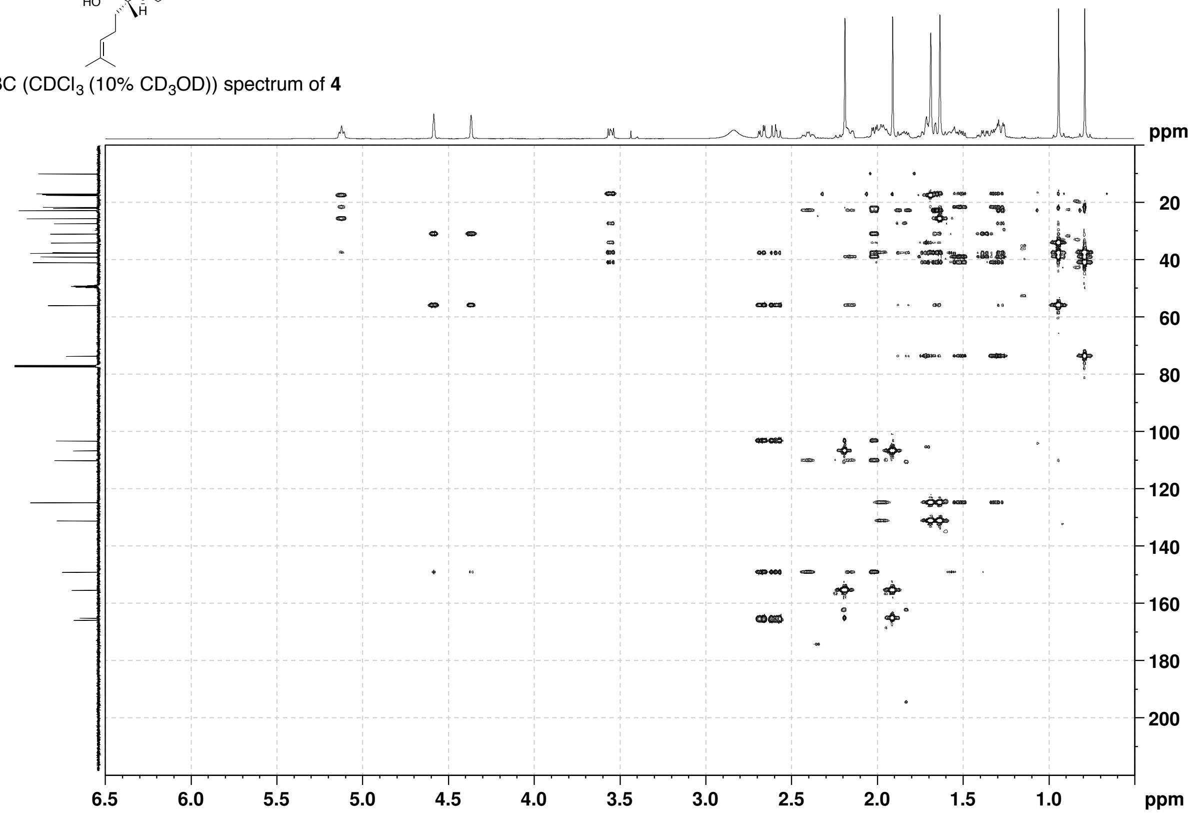

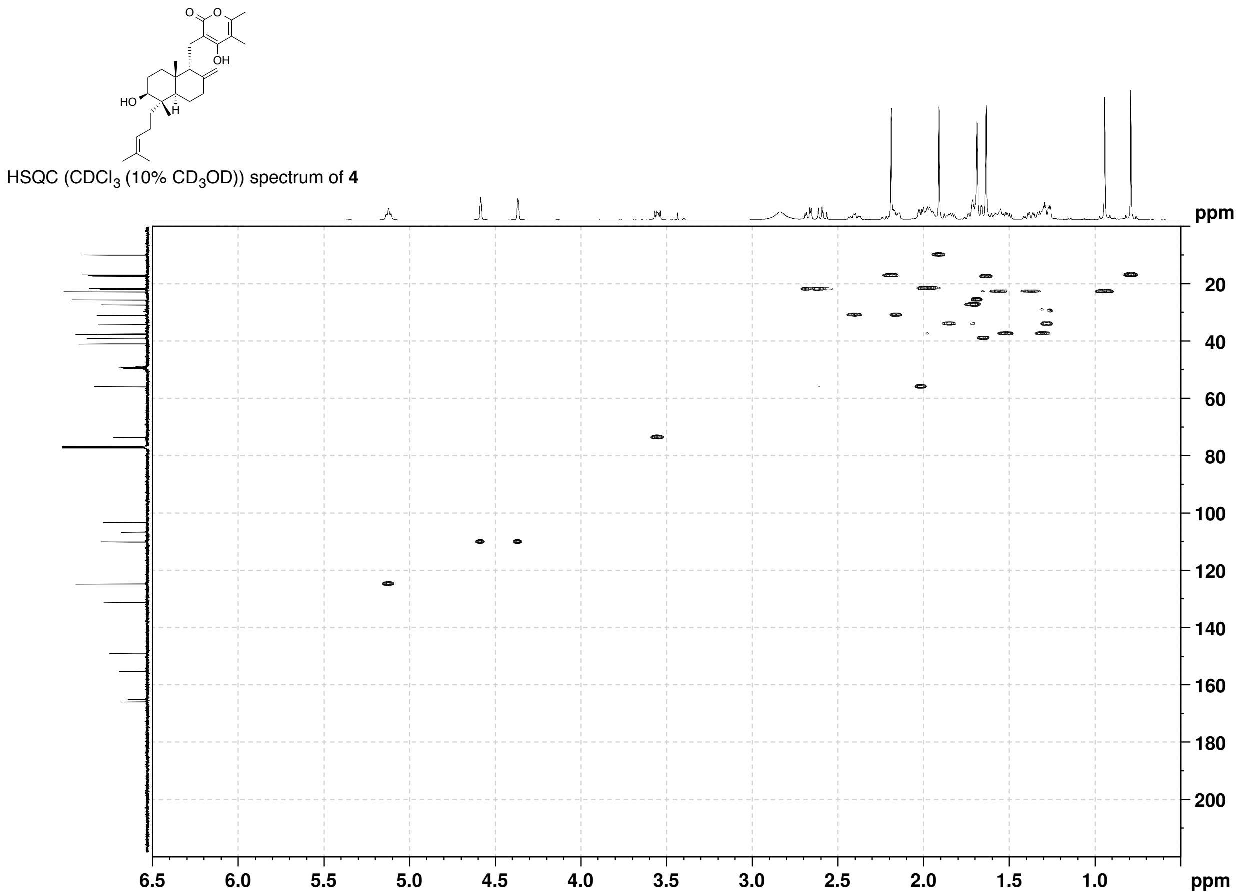

HSQC ($CDCl_3$ (10% CD_3OD)) spectrum of **4**

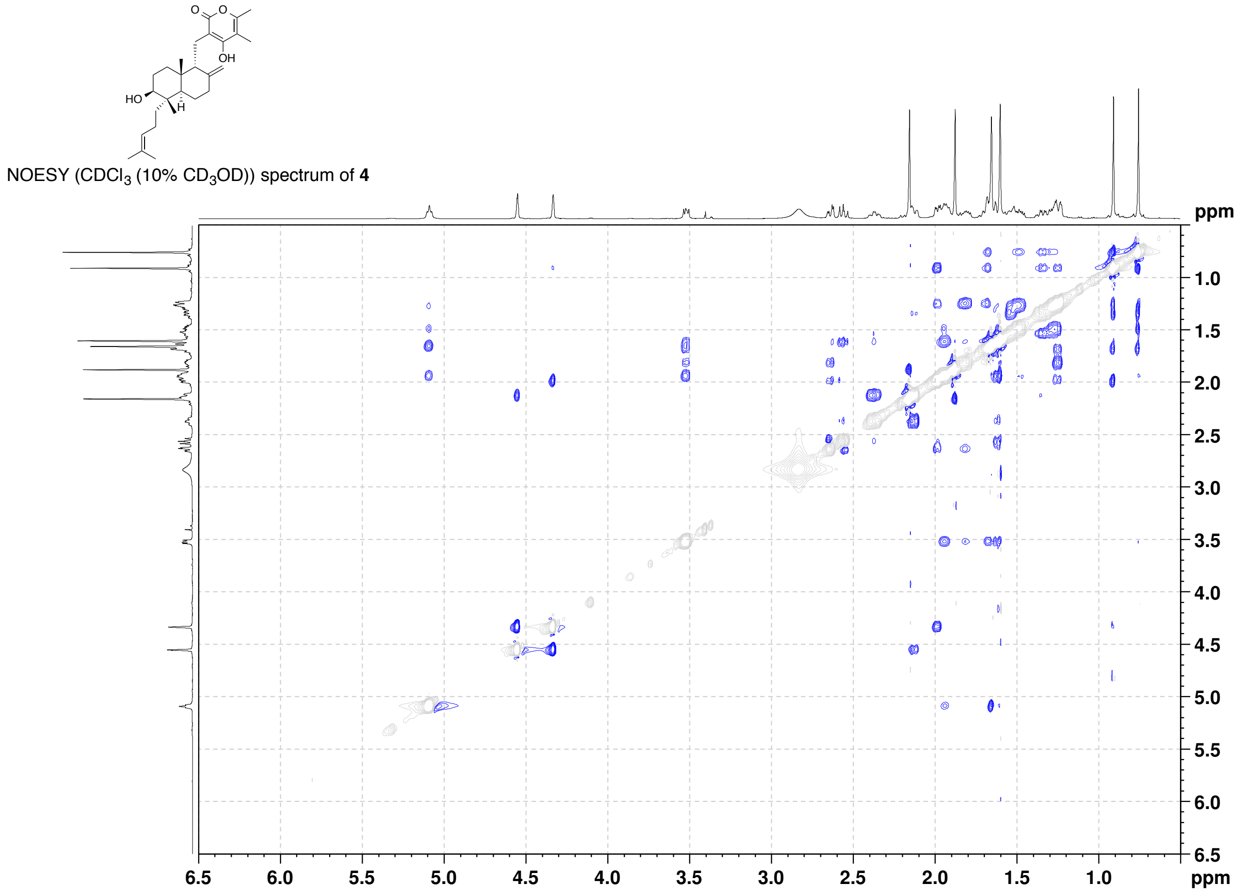

NOESY ($CDCl_3$ (10% CD_3OD)) spectrum of **4**

Supplementary Fig. 54. NMR spectra of 4 ($CDCl_3$ (10% CD_3OD)

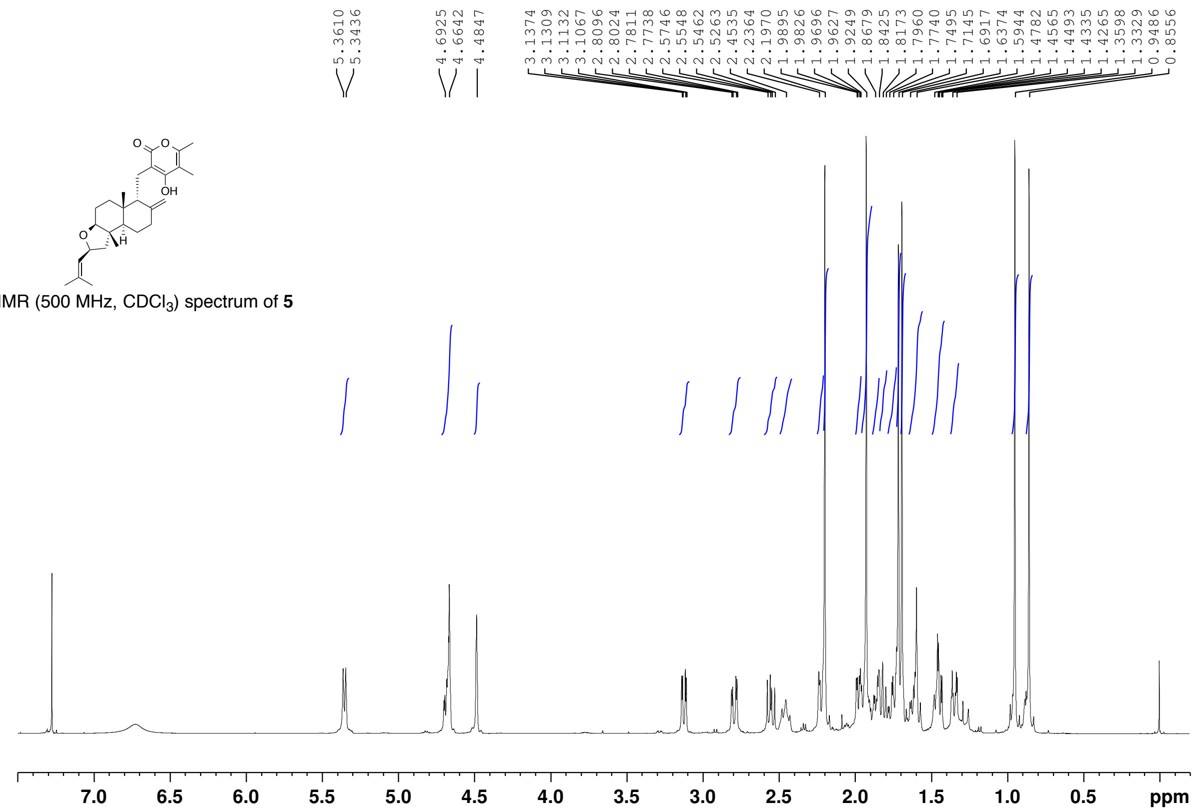

^{1}H NMR (500 MHz, $CDCl_3$) spectrum of **5**

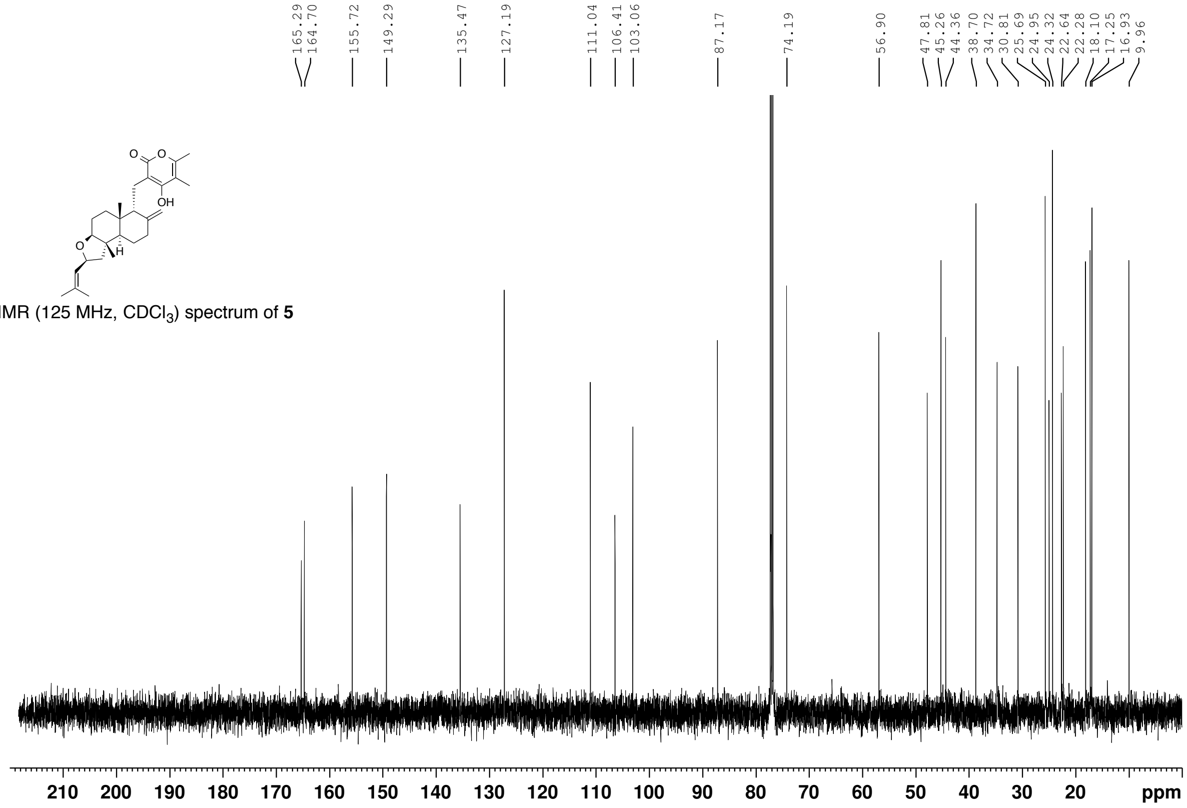

^{13}C NMR (125 MHz, $CDCl_3$) spectrum of **5**

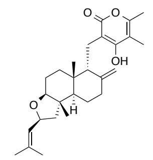

^1H-^1H COSY ($CDCl_3$) spectrum of **5**

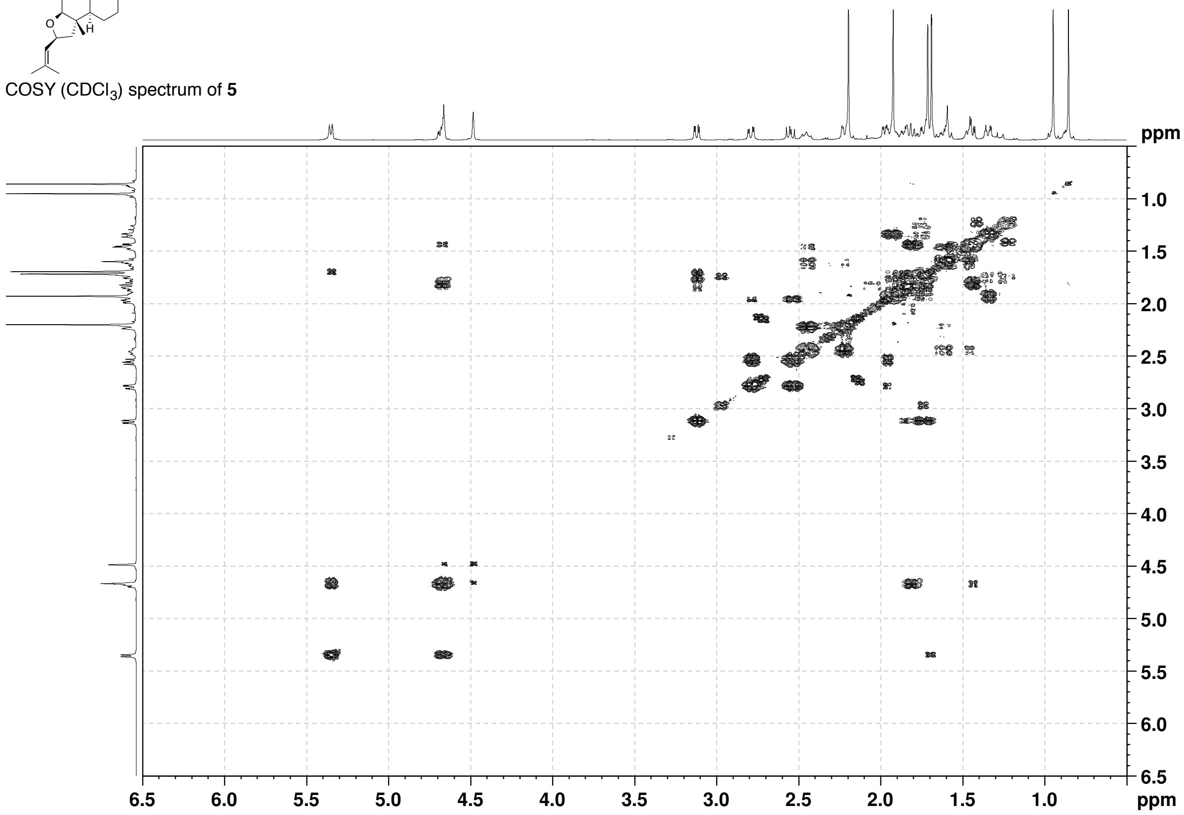

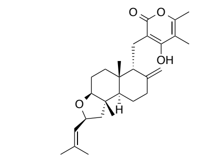

HMBC ($CDCl_3$) spectrum of **5**

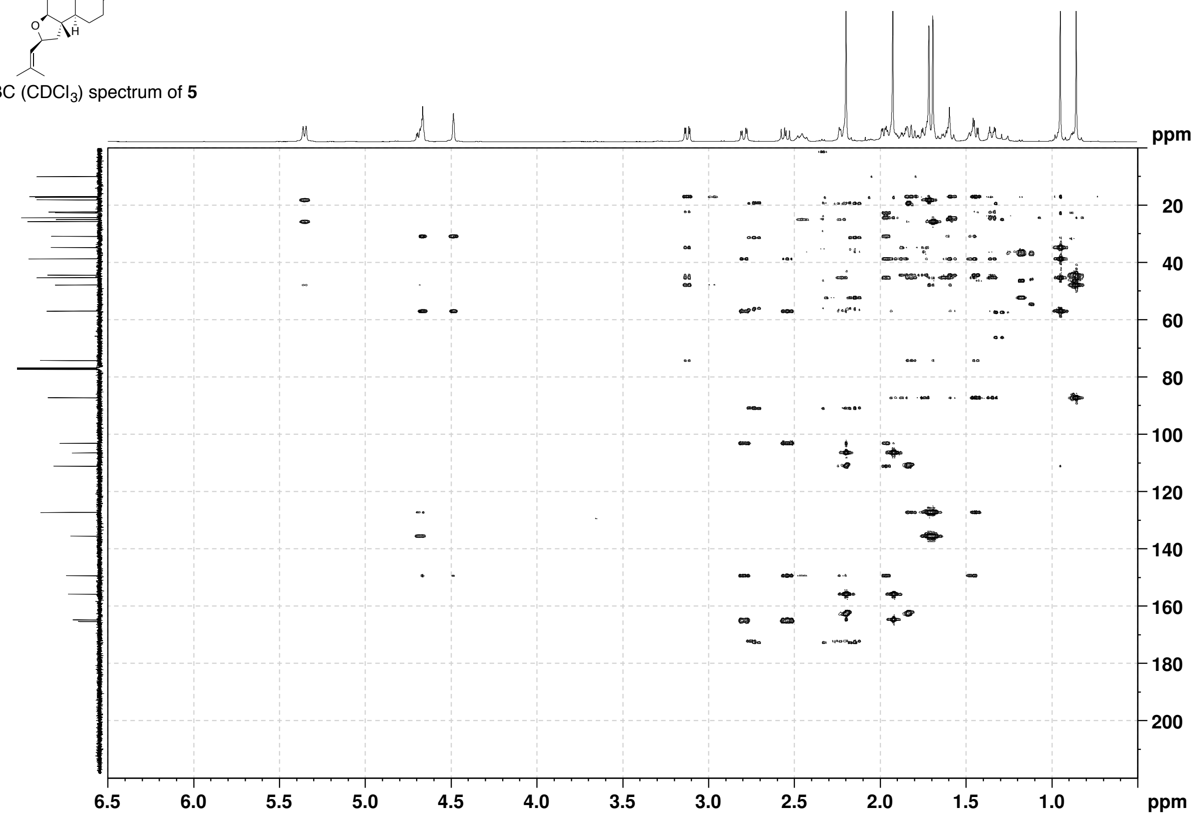

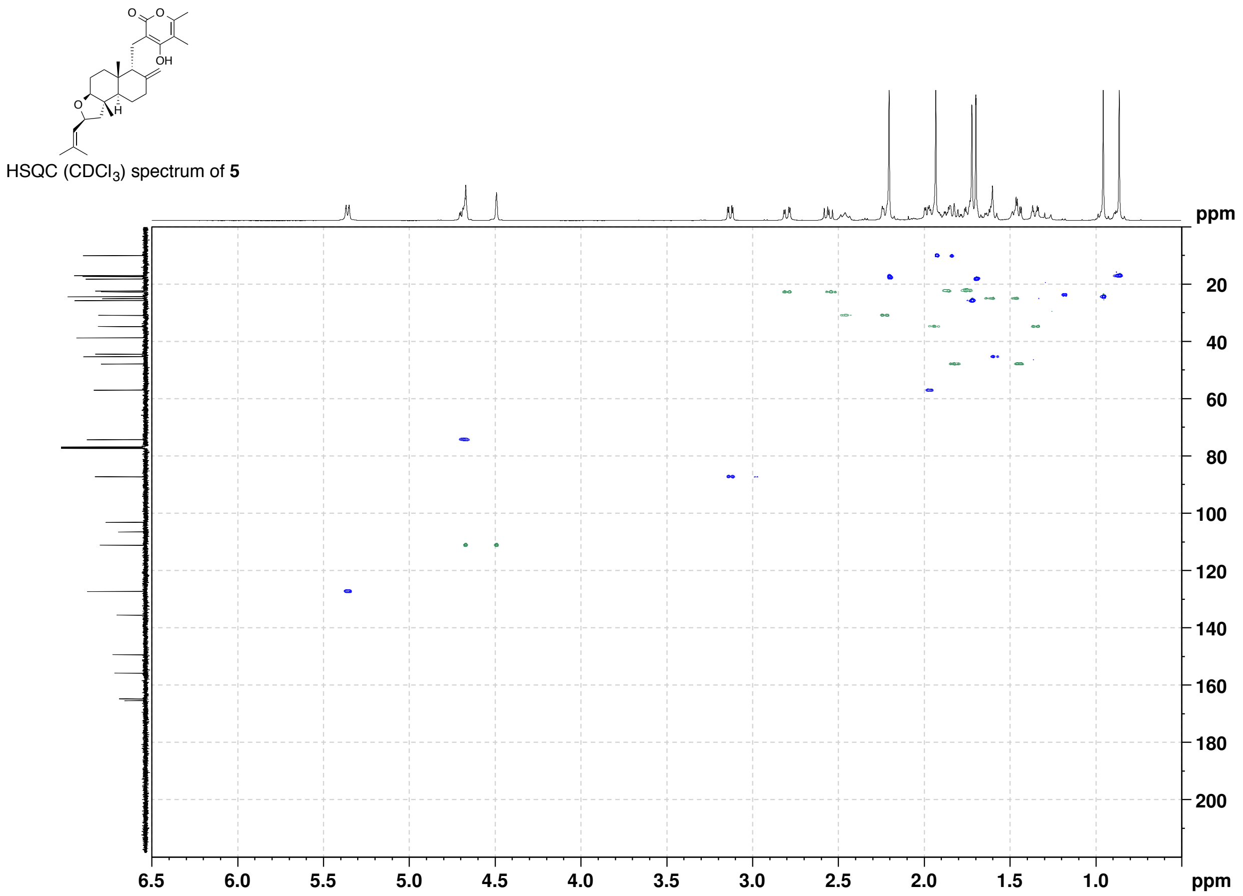

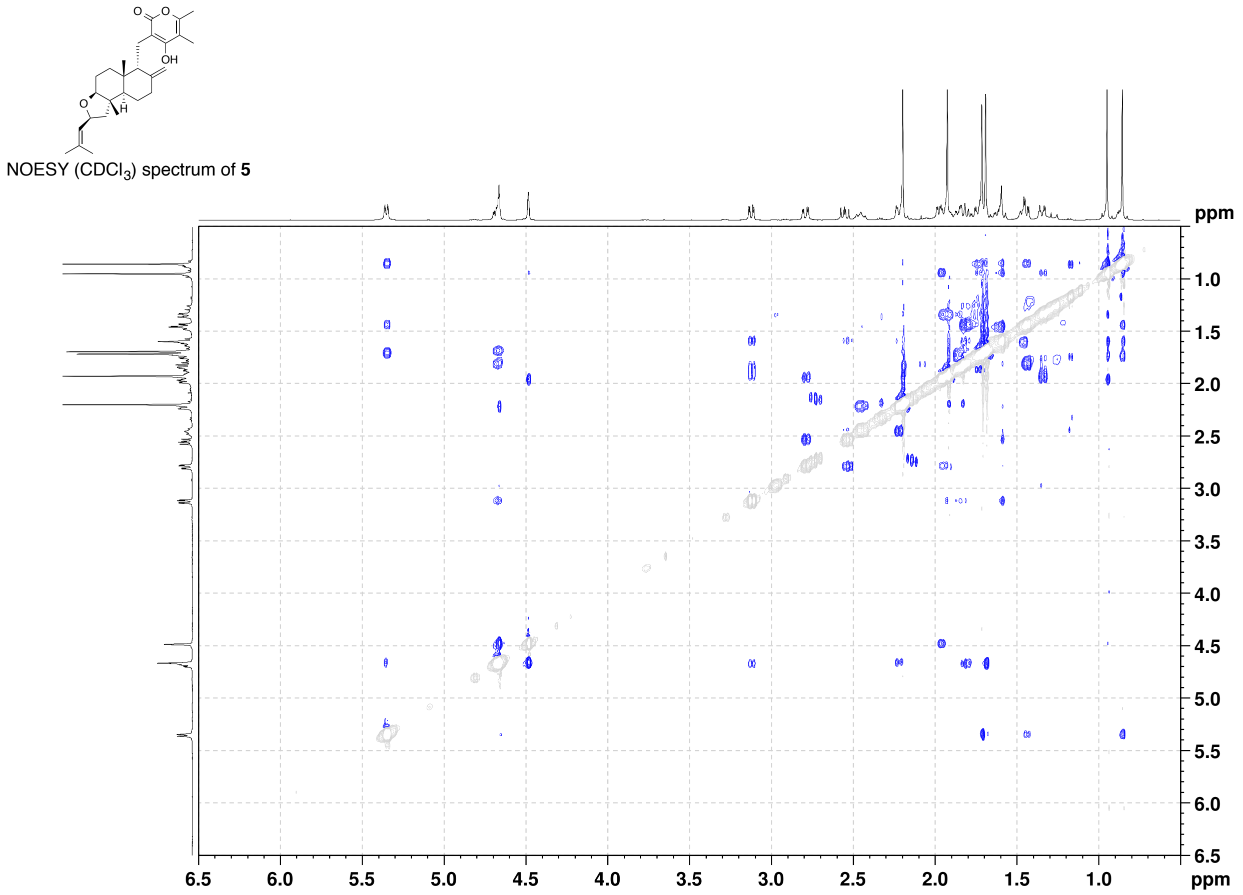

Supplementary Fig. 55. NMR spectra of 5 ($CDCl_3$)

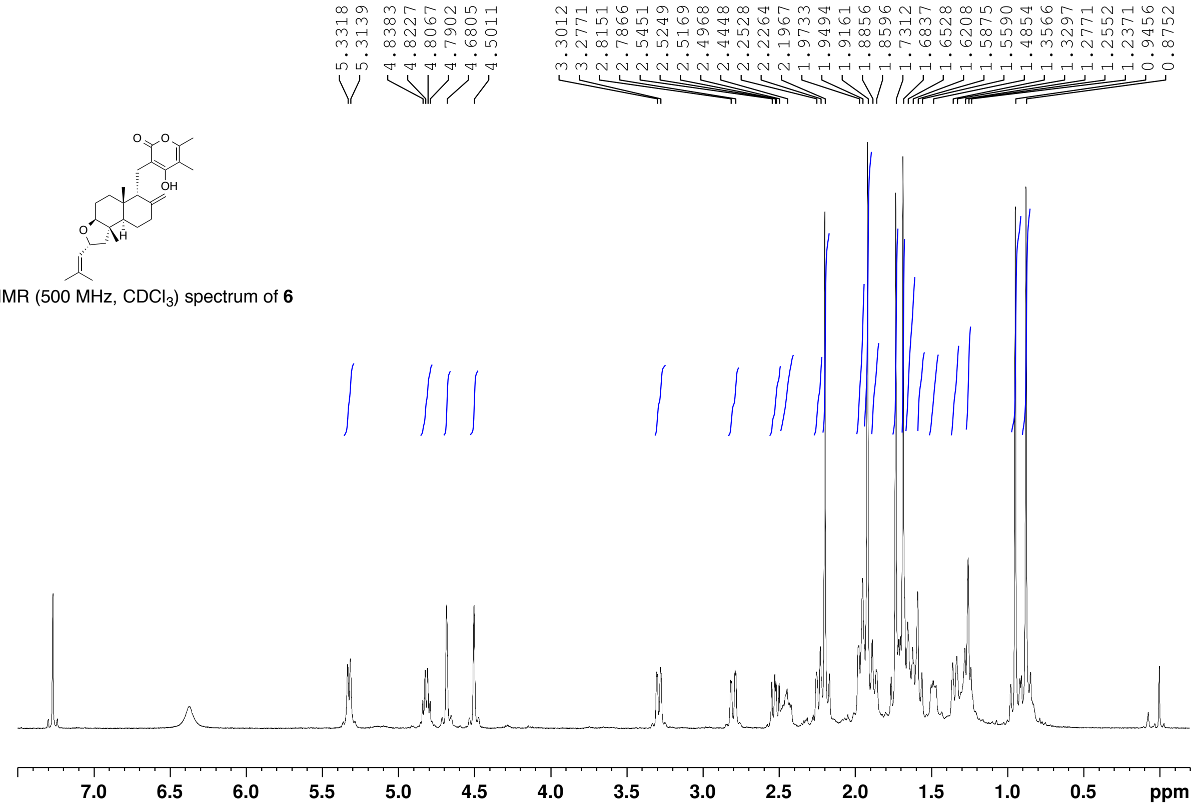

^{1}H NMR (500 MHz, $CDCl_3$) spectrum of **6**

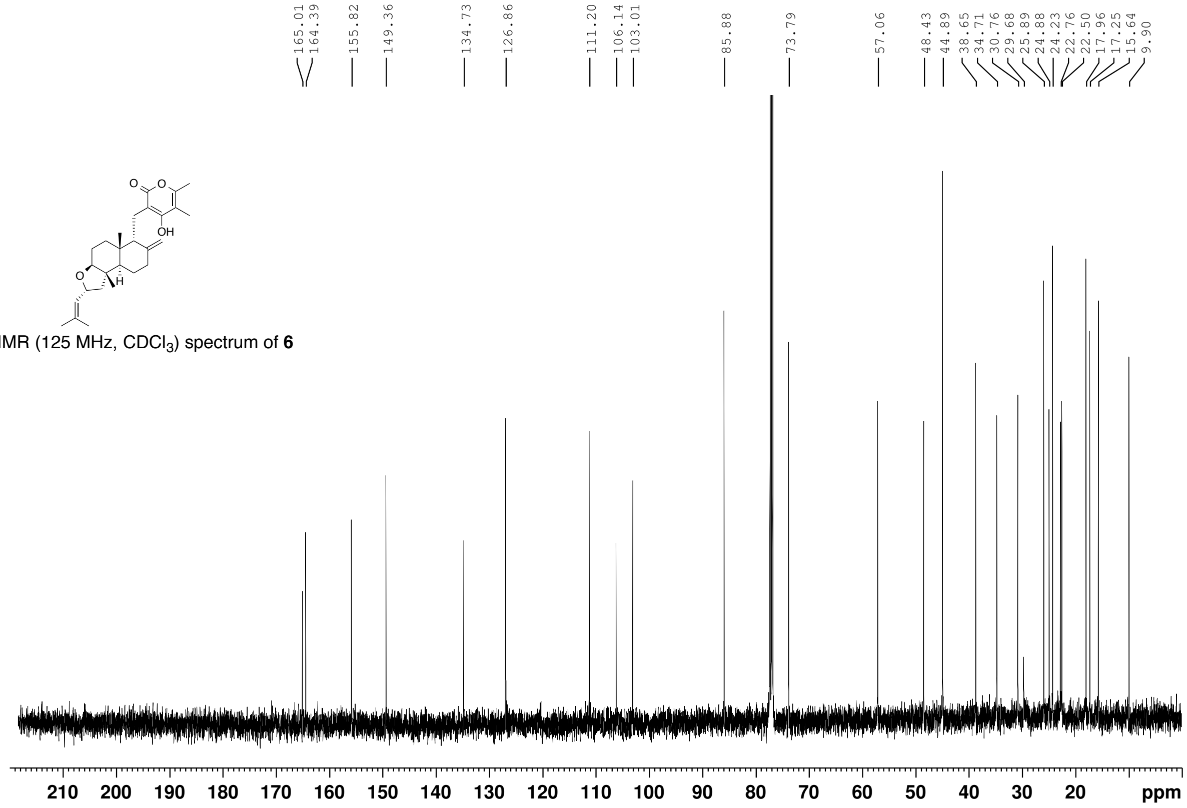

^{13}C NMR (125 MHz, $CDCl_3$) spectrum of **6**

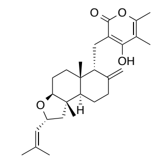

^{1}H-^{1}H COSY ($CDCl_3$) spectrum of **6**

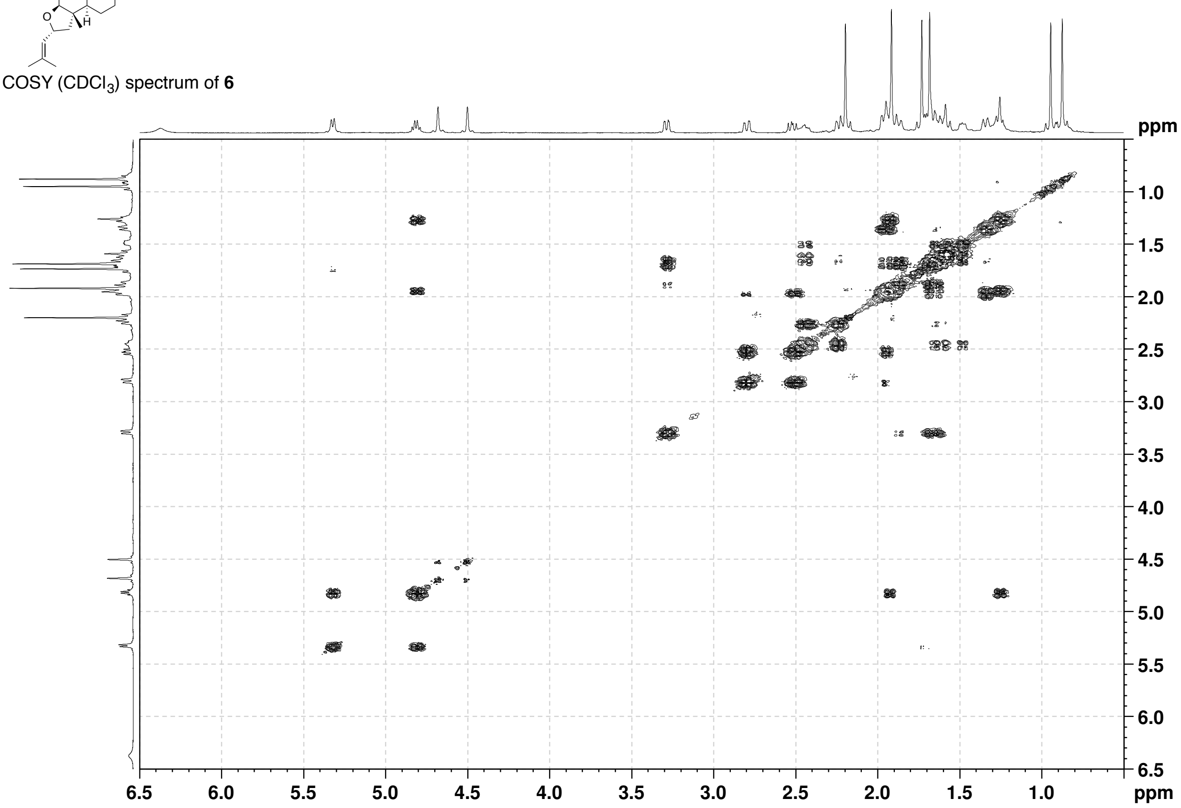

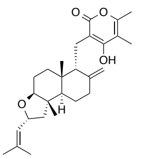

HMBC ($CDCl_3$) spectrum of **6**

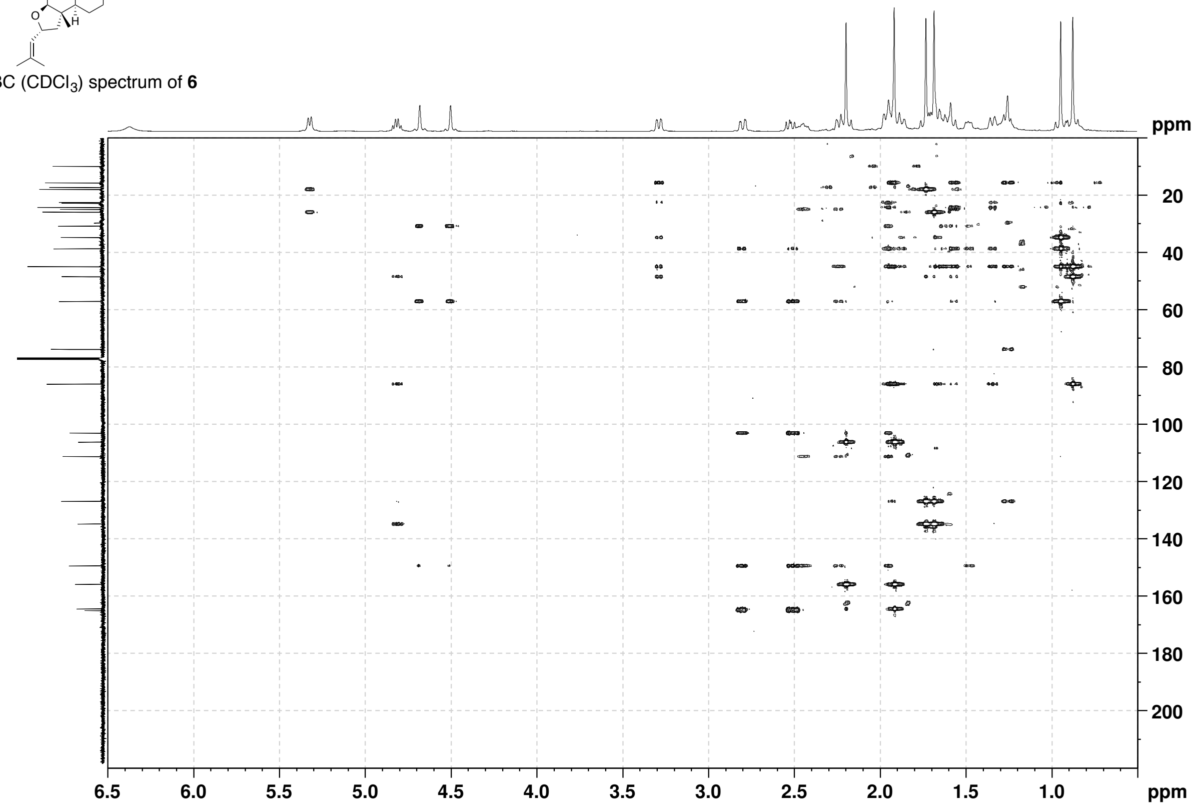

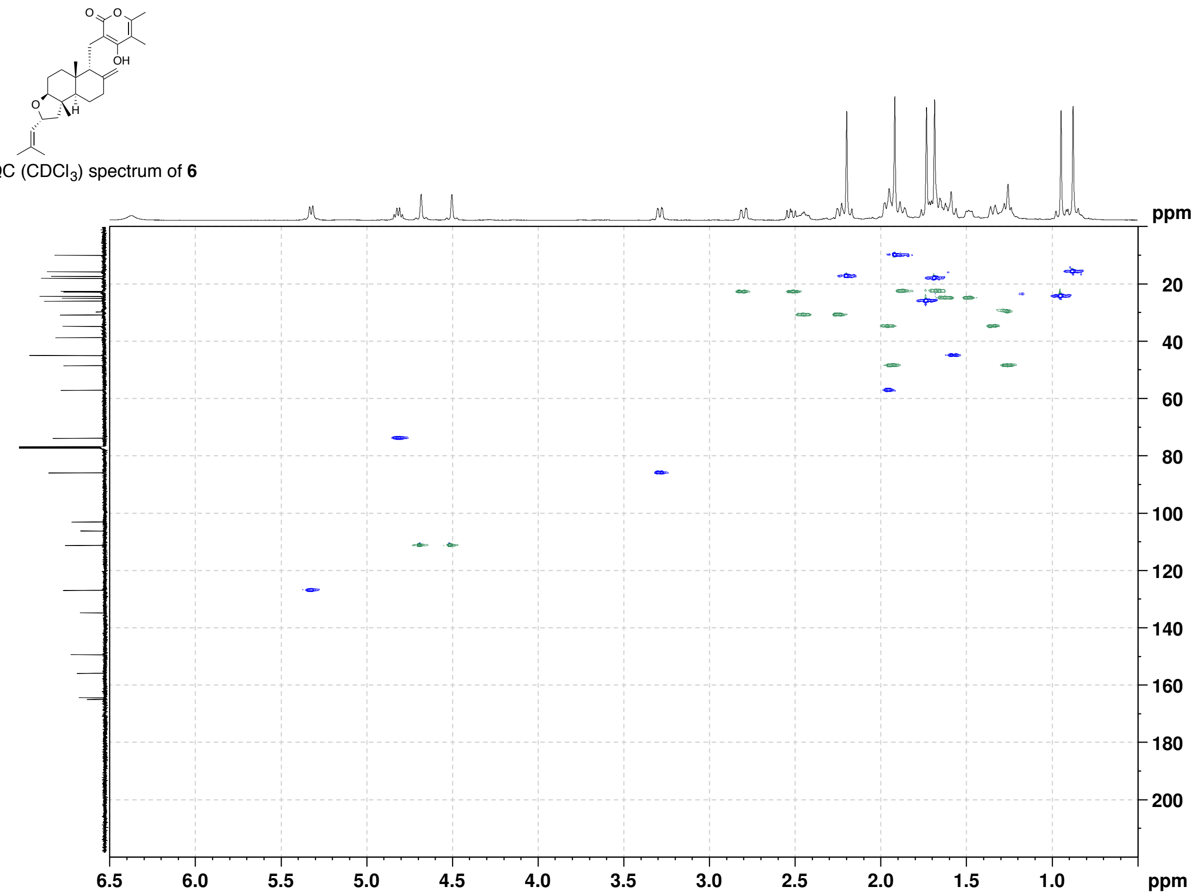

HSQC ($CDCl_3$) spectrum of **6**

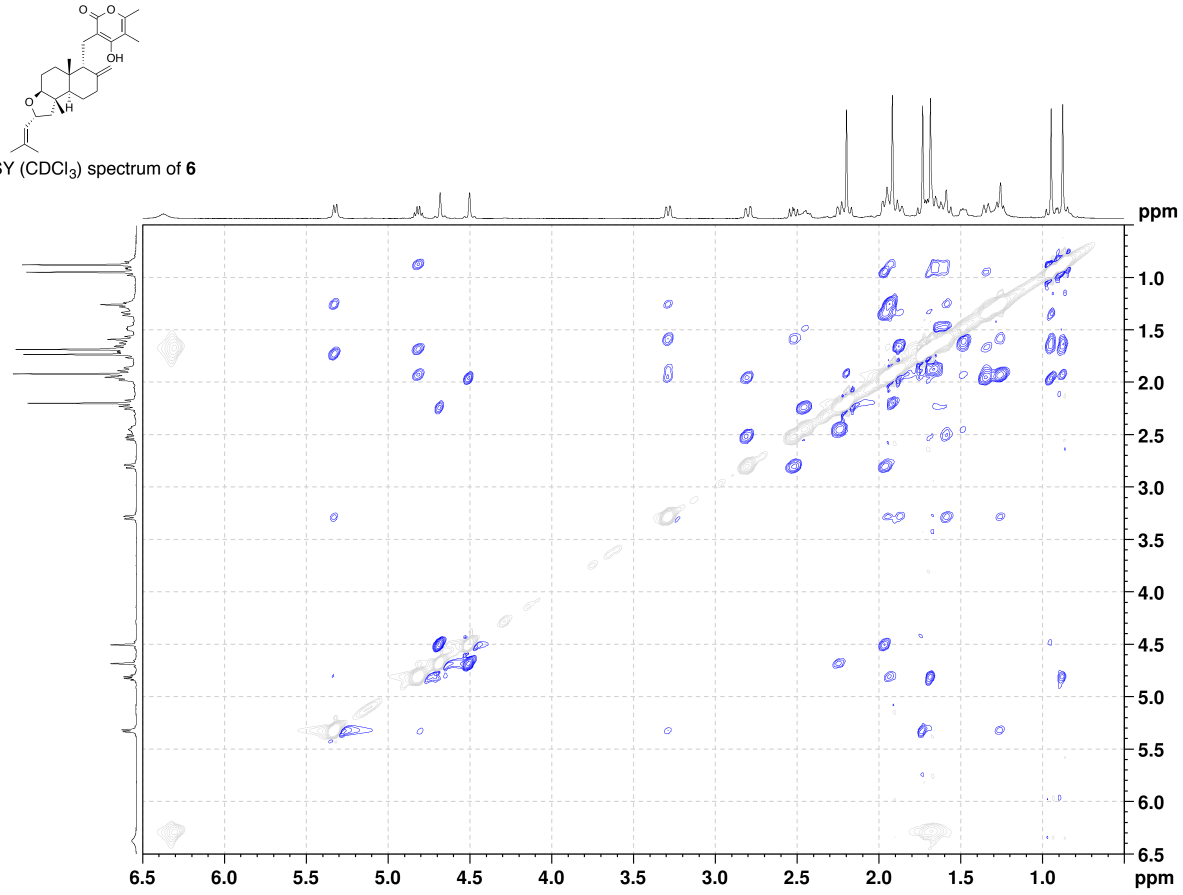

NOESY ($CDCl_3$) spectrum of **6**

Supplementary Fig. 56. NMR spectra of 6 ($CDCl_3$)

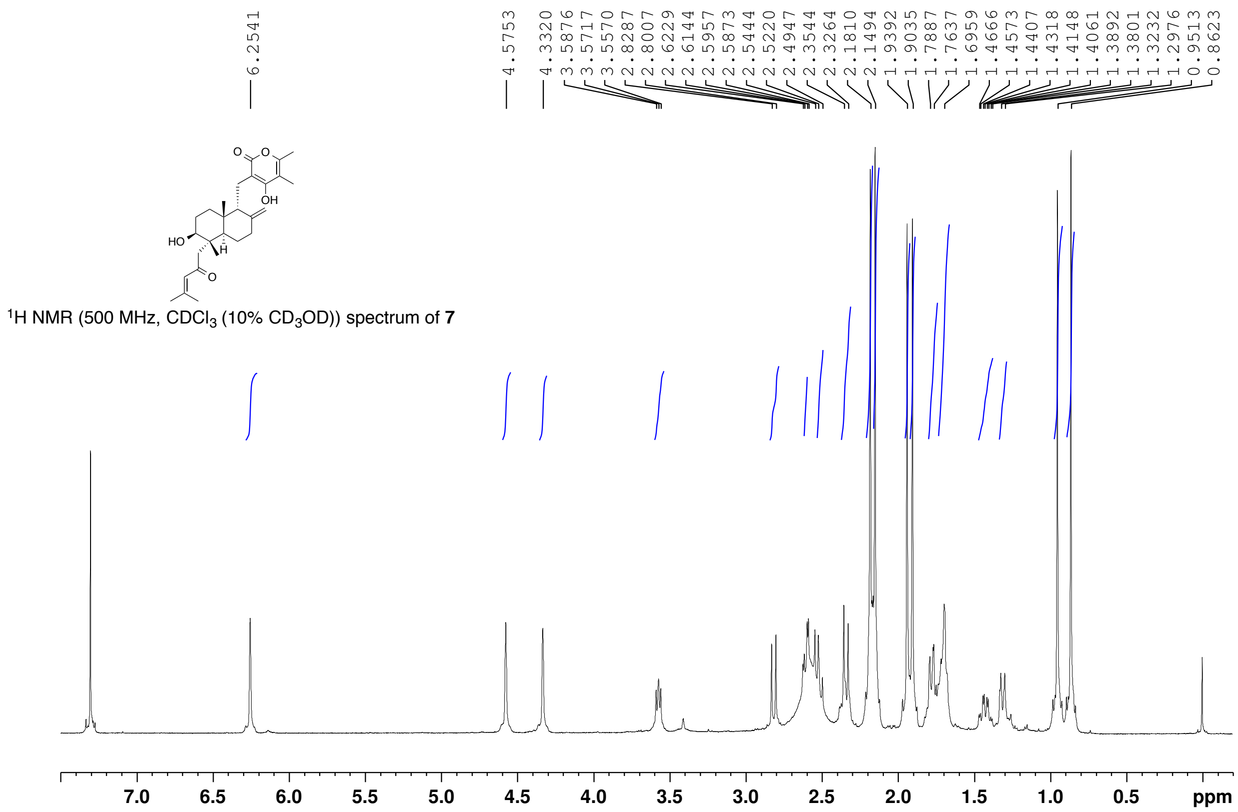

^{1}H NMR (500 MHz, $CDCl_3$ (10% CD_3OD)) spectrum of **7**

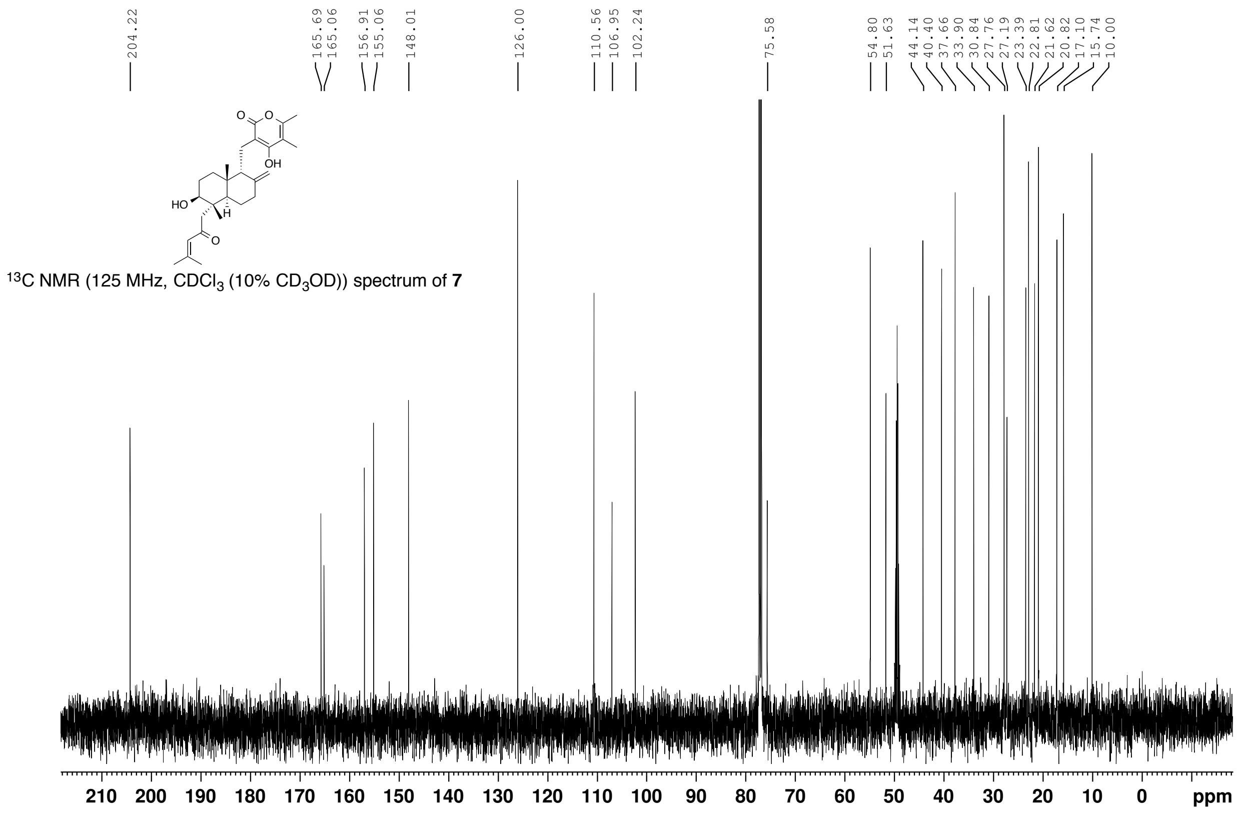

^{13}C NMR (125 MHz, $CDCl_3$ (10% CD_3OD)) spectrum of **7**

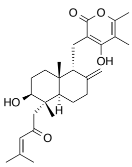

^{1}H-^{1}H COSY ($CDCl_3$ (10% CD_3OD)) spectrum of **7**

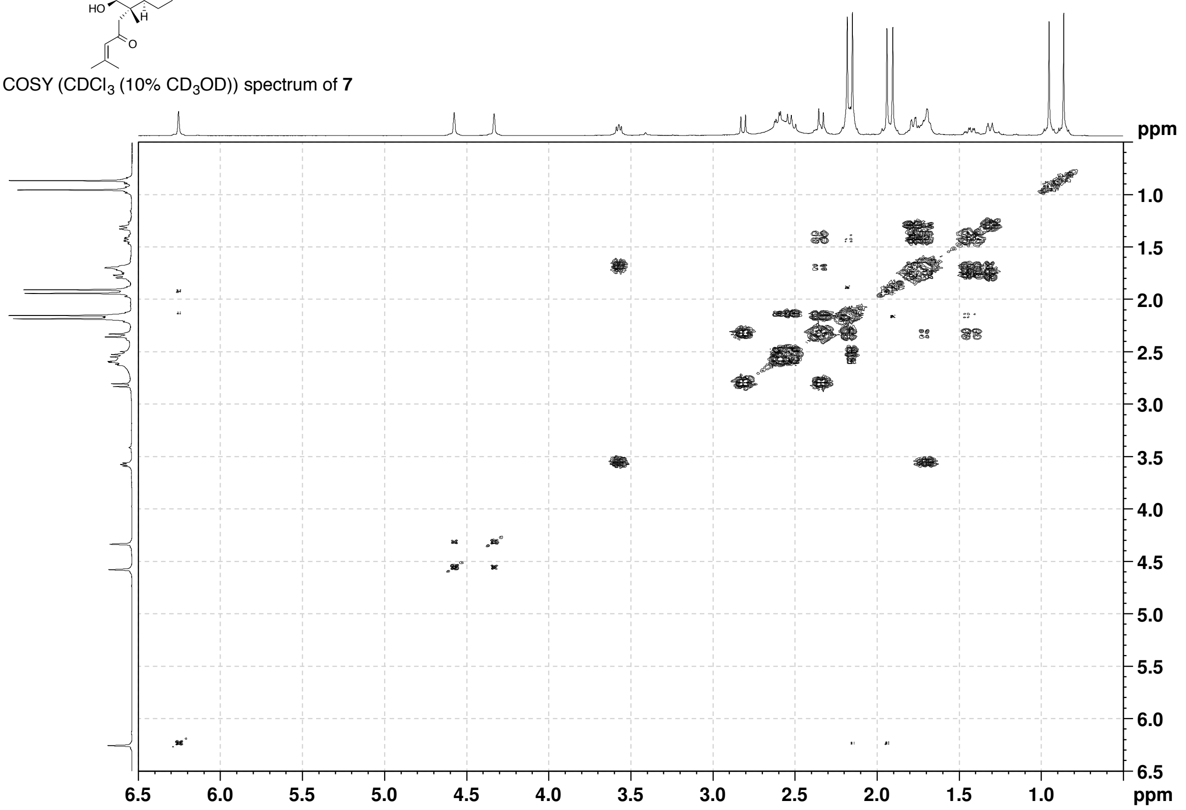

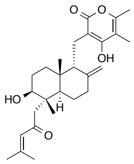

HMBC ($CDCl_3$ (10% CD_3OD)) spectrum of **7**

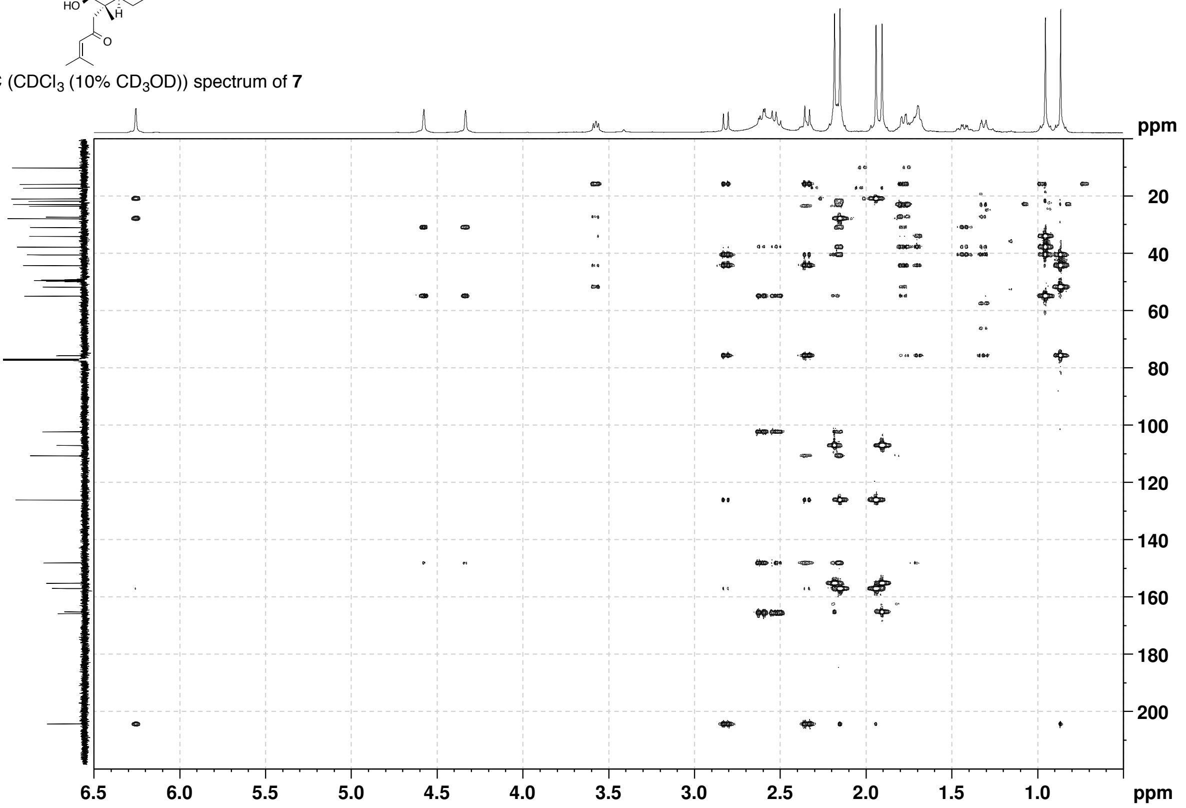

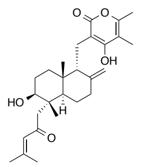

HSQC ($CDCl_3$ (10% CD_3OD)) spectrum of **7**

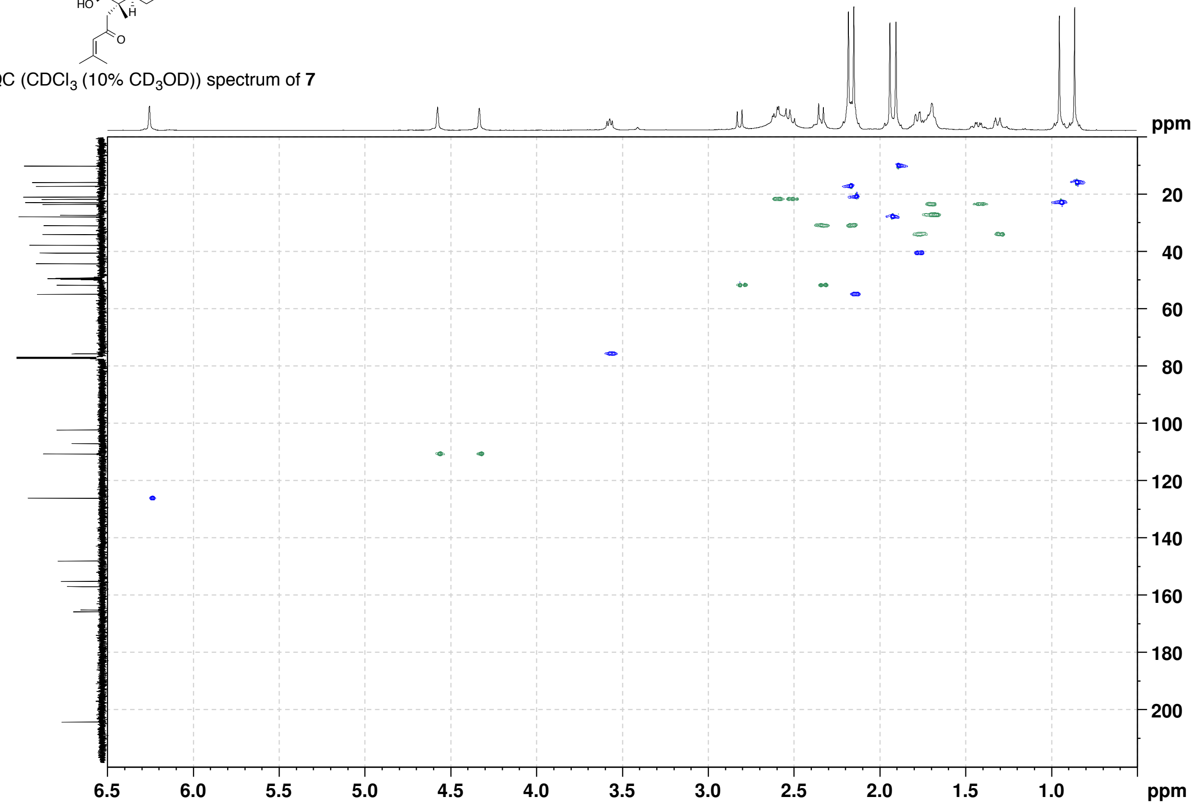

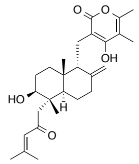

NOESY ($CDCl_3$ (10% CD_3OD)) spectrum of **7**

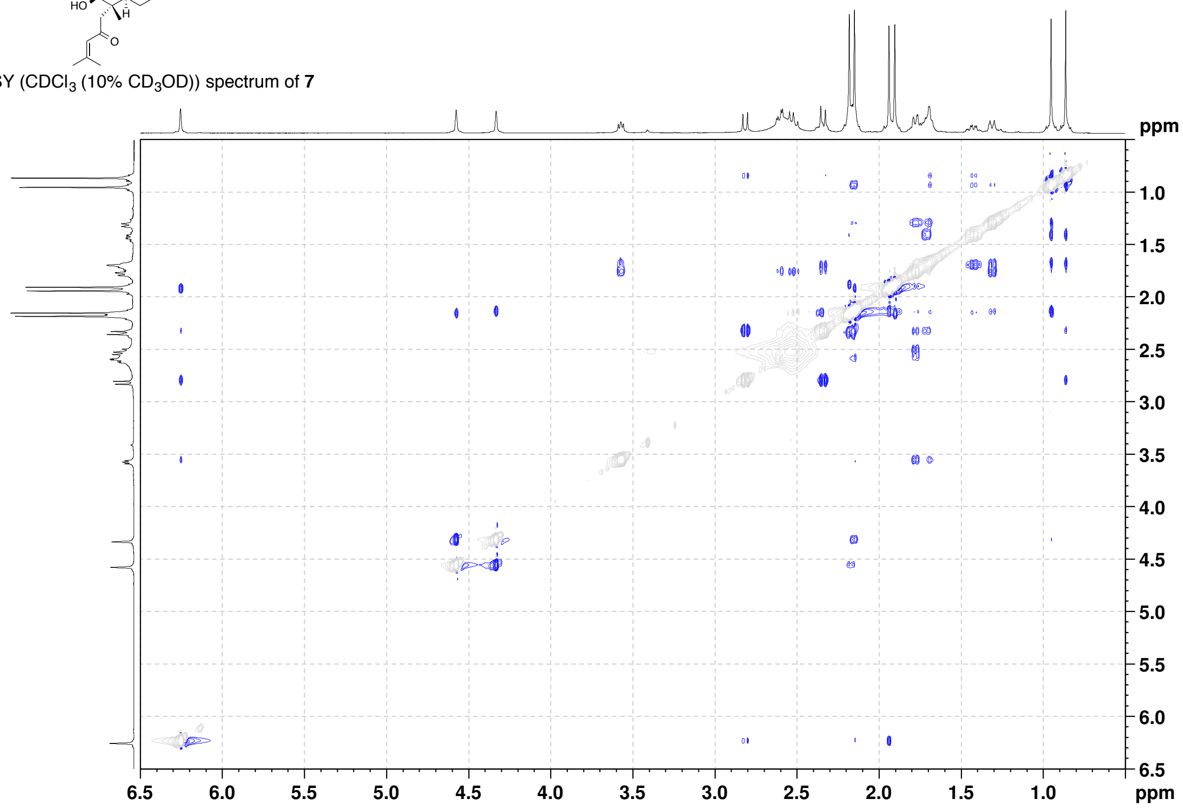

Supplementary Fig. 57. NMR spectra of 7 ($CDCl_3$ (10% CD_3OD)

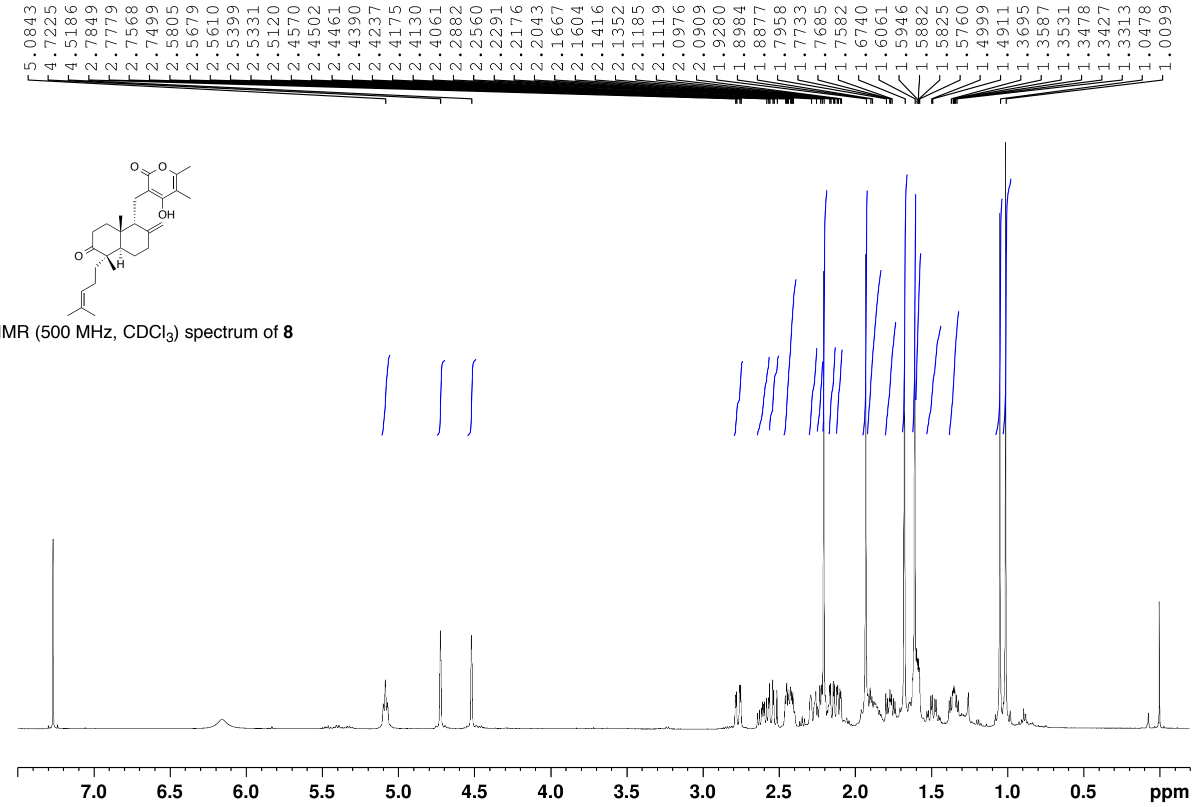

^{1}H NMR (500 MHz, $CDCl_3$) spectrum of **8**

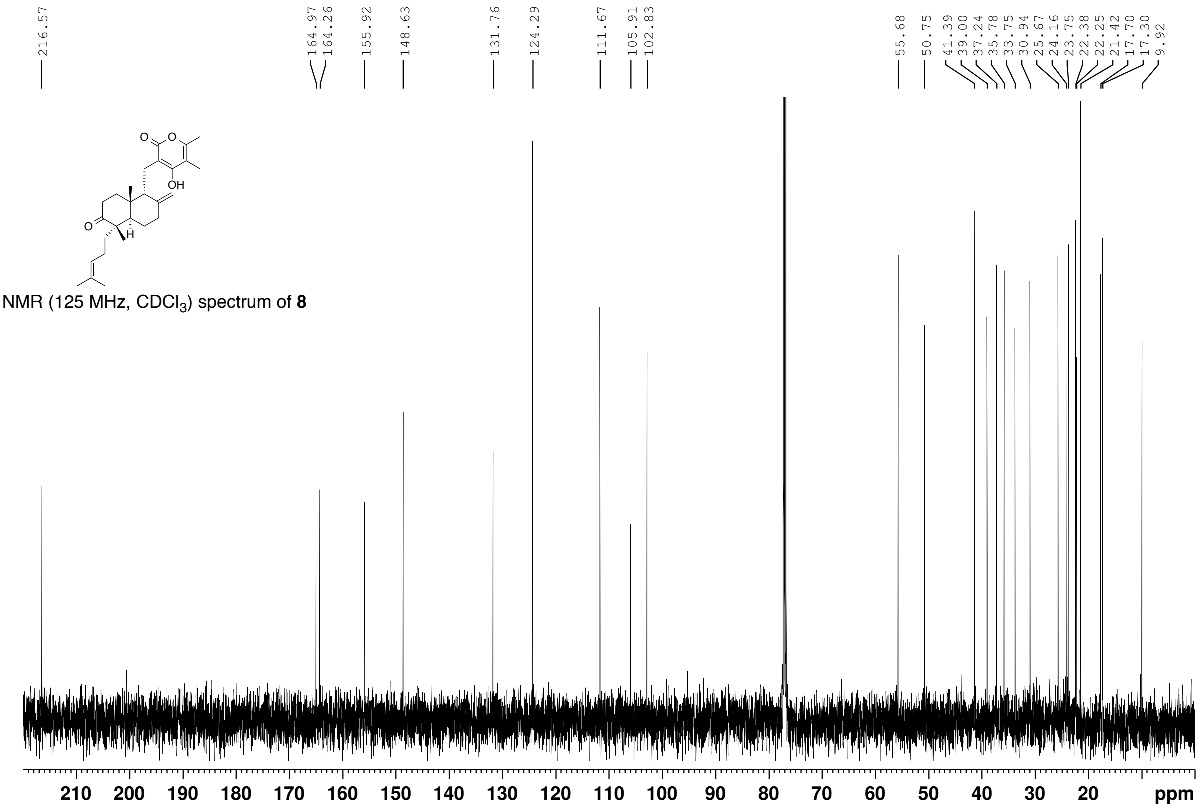

^{13}C NMR (125 MHz, $CDCl_3$) spectrum of **8**

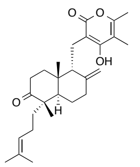

^{1}H-^{1}H COSY ($CDCl_3$) spectrum of **8**

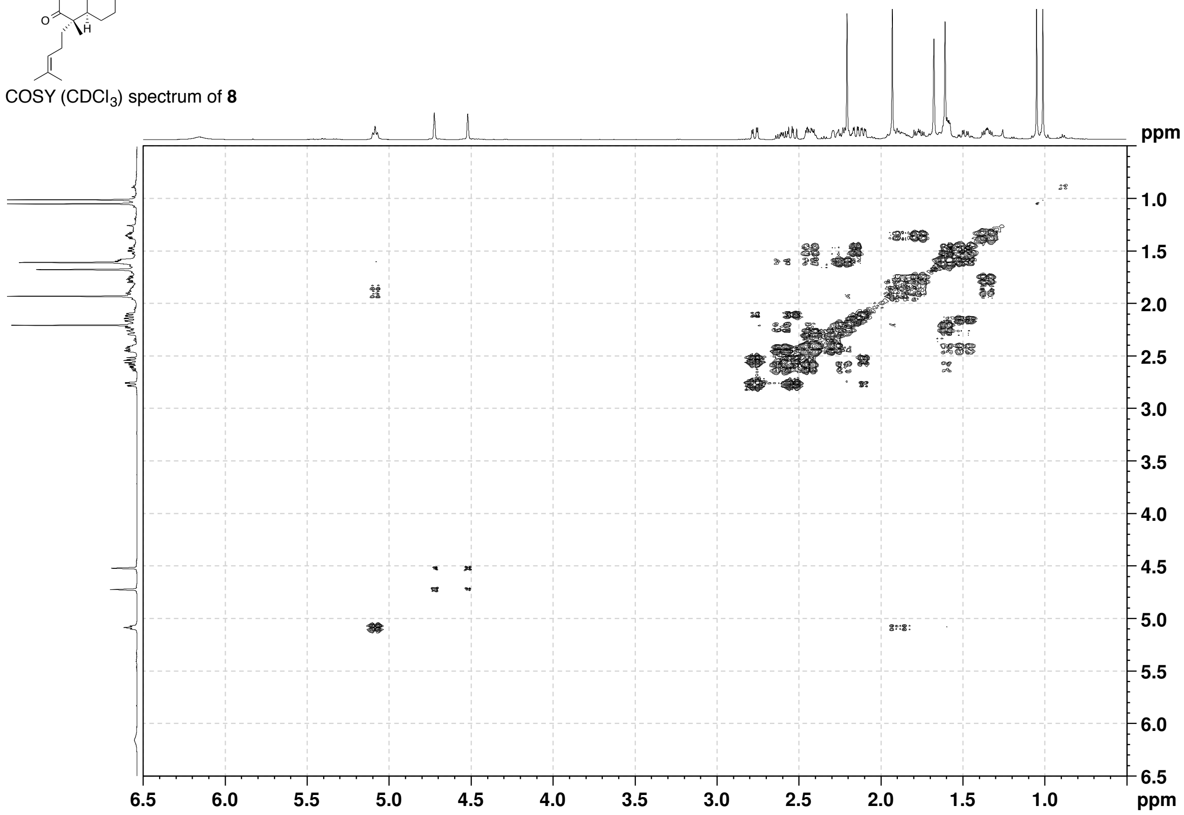

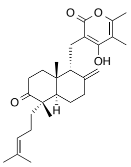

HMBC ($CDCl_3$) spectrum of **8**

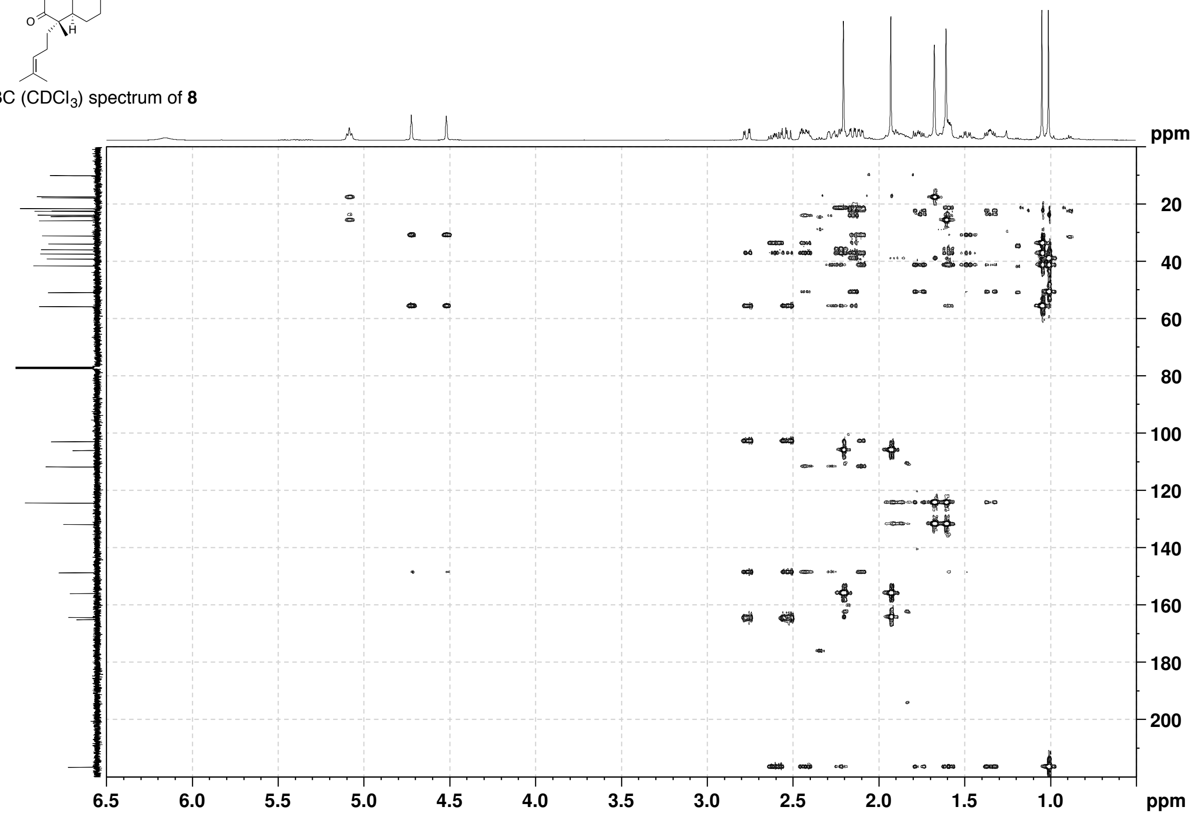

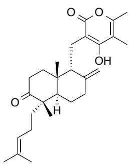

HSQC (CDCl$_3$) spectrum of **8**

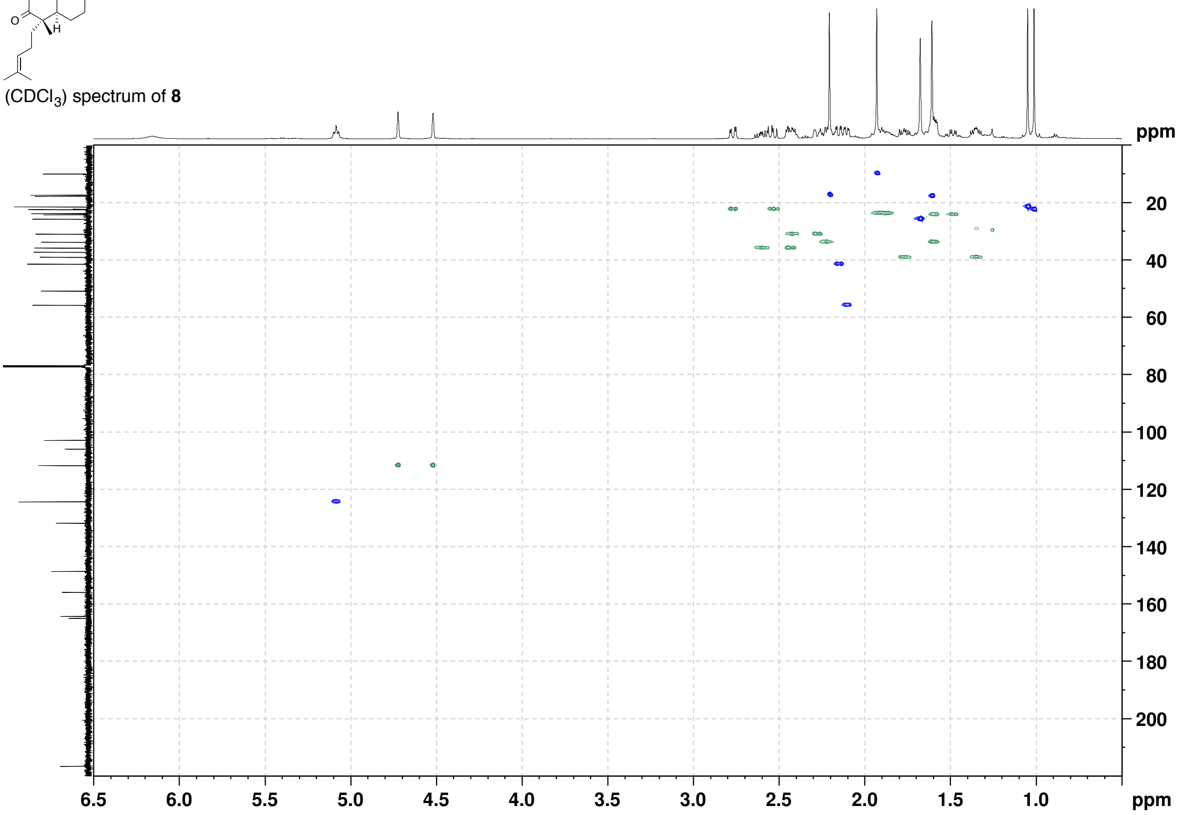

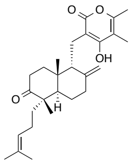

NOESY (CDCl$_3$) spectrum of **8**

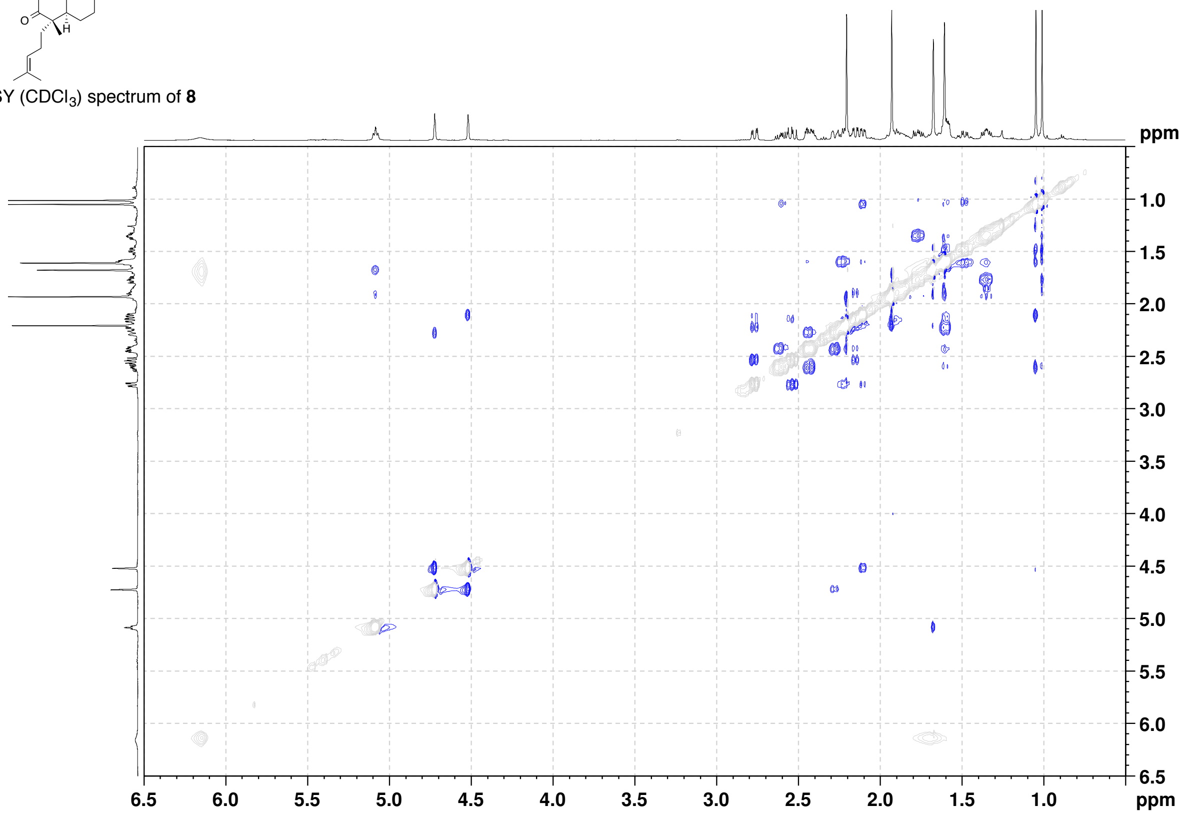

Supplementary Fig. 58. NMR spectra of 8 (CDCl$_3$)

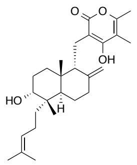

^{1}H NMR (500 MHz, $CDCl_3$ (10% CD_3OD)) spectrum of **9**

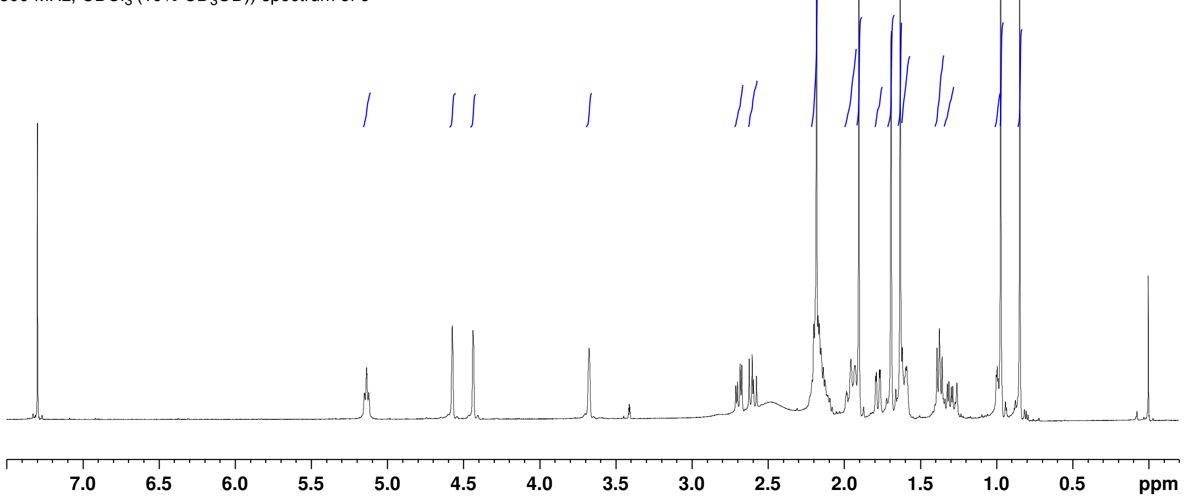

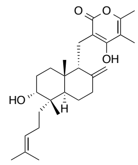

^{13}C NMR (125 MHz, $CDCl_3$ (10% CD_3OD)) spectrum of **9**

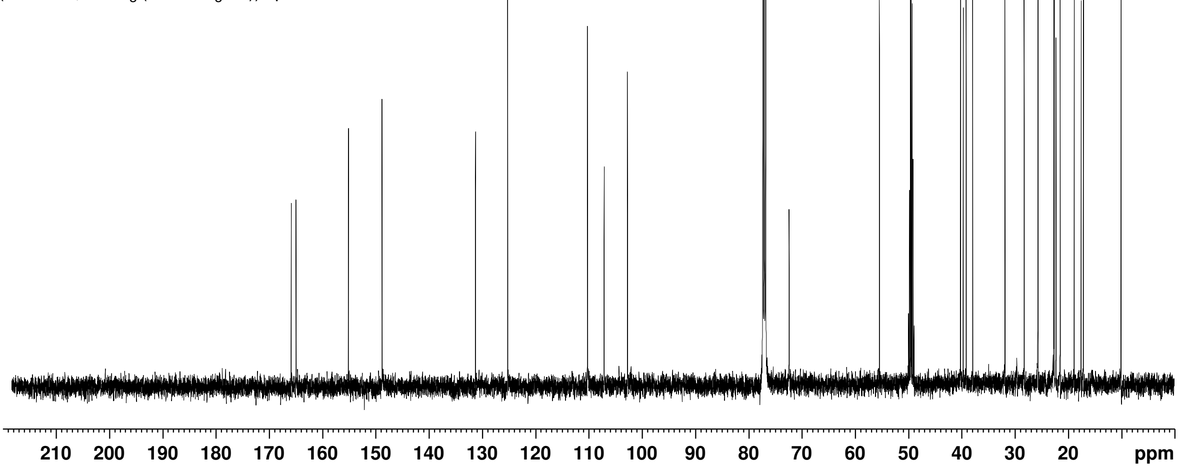

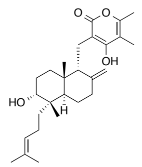

^{1}H-^{1}H COSY ($CDCl_3$ (10% CD_3OD)) spectrum of **9**

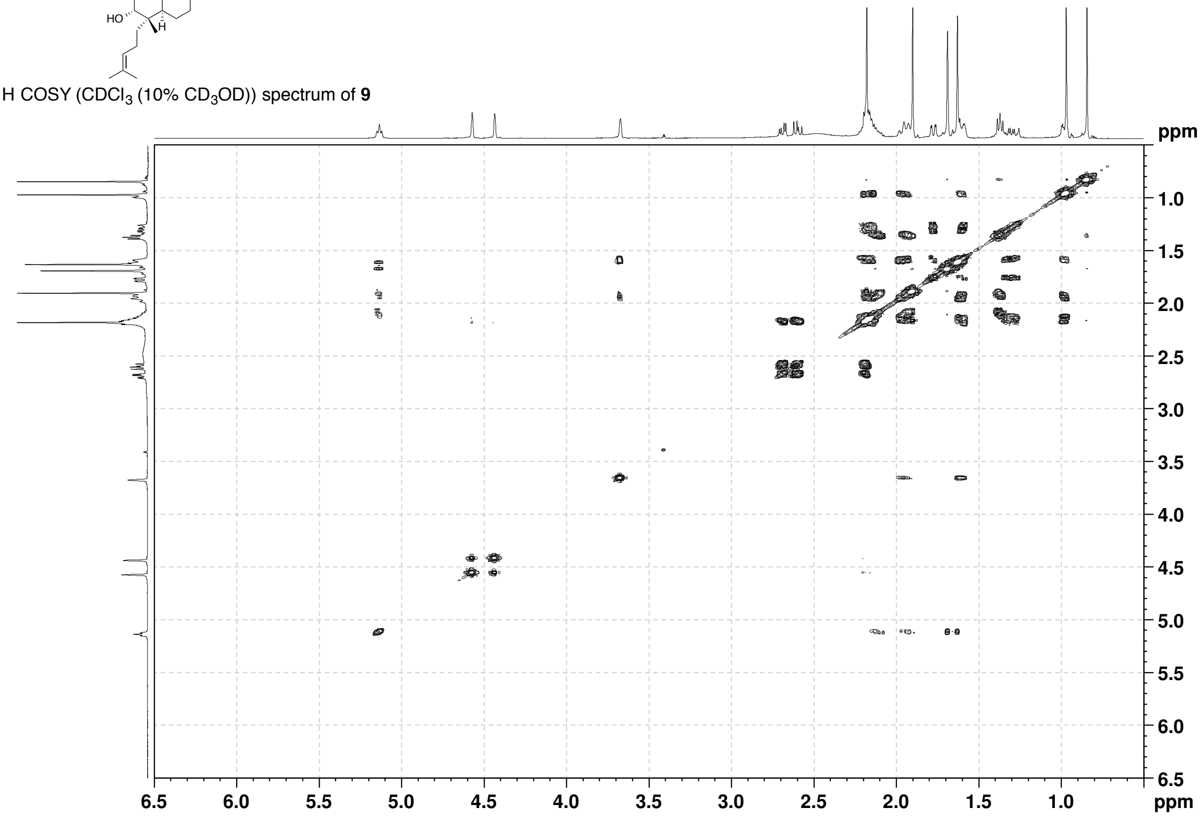

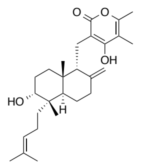

HMBC ($CDCl_3$ (10% CD_3OD)) spectrum of **9**

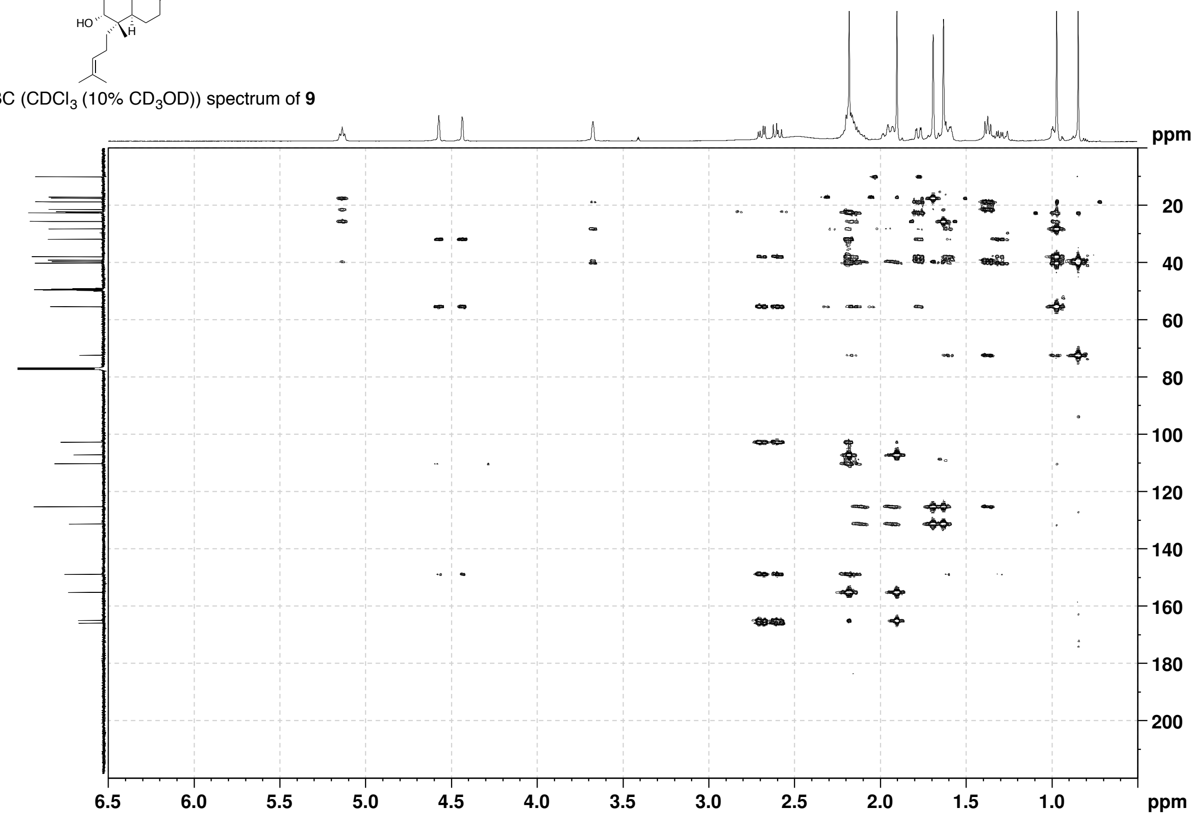

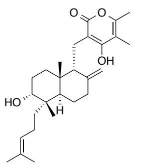

HSQC ($CDCl_3$ (10% CD_3OD)) spectrum of **9**

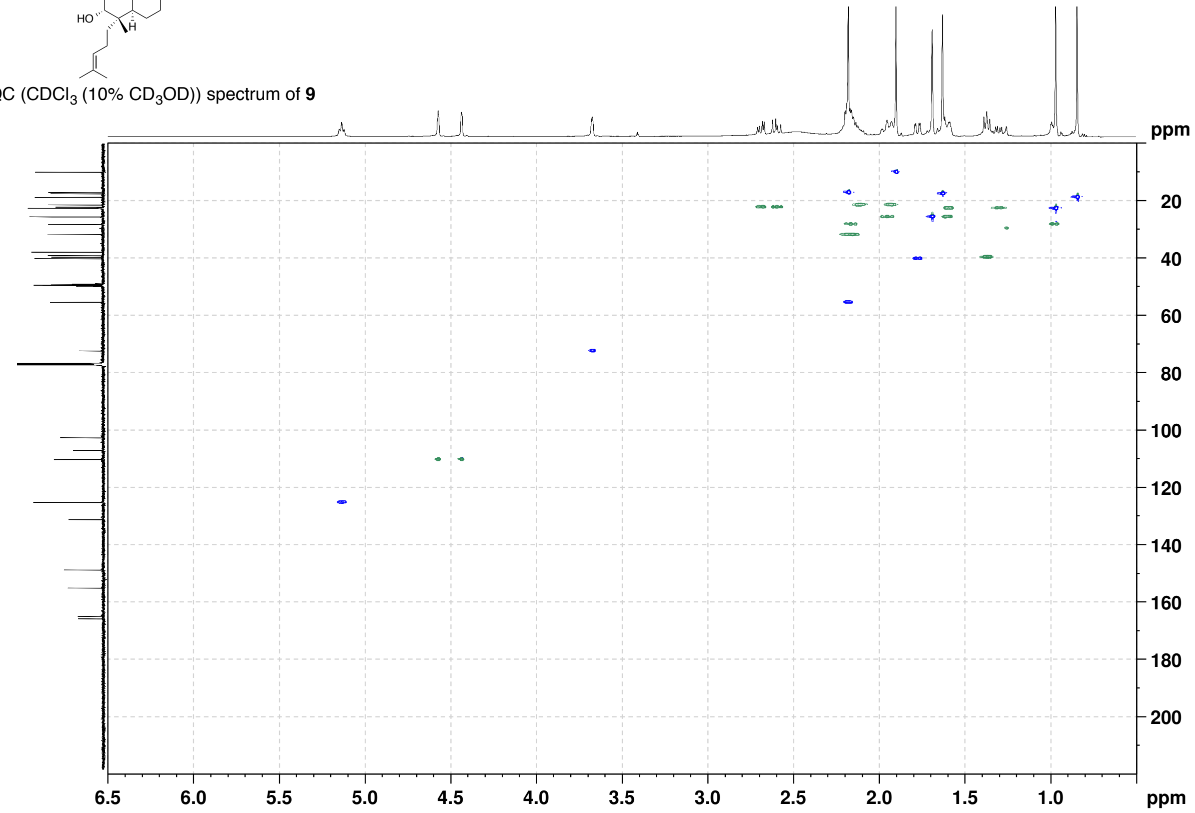

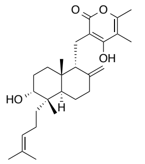

NOESY ($CDCl_3$ (10% CD_3OD)) spectrum of **9**

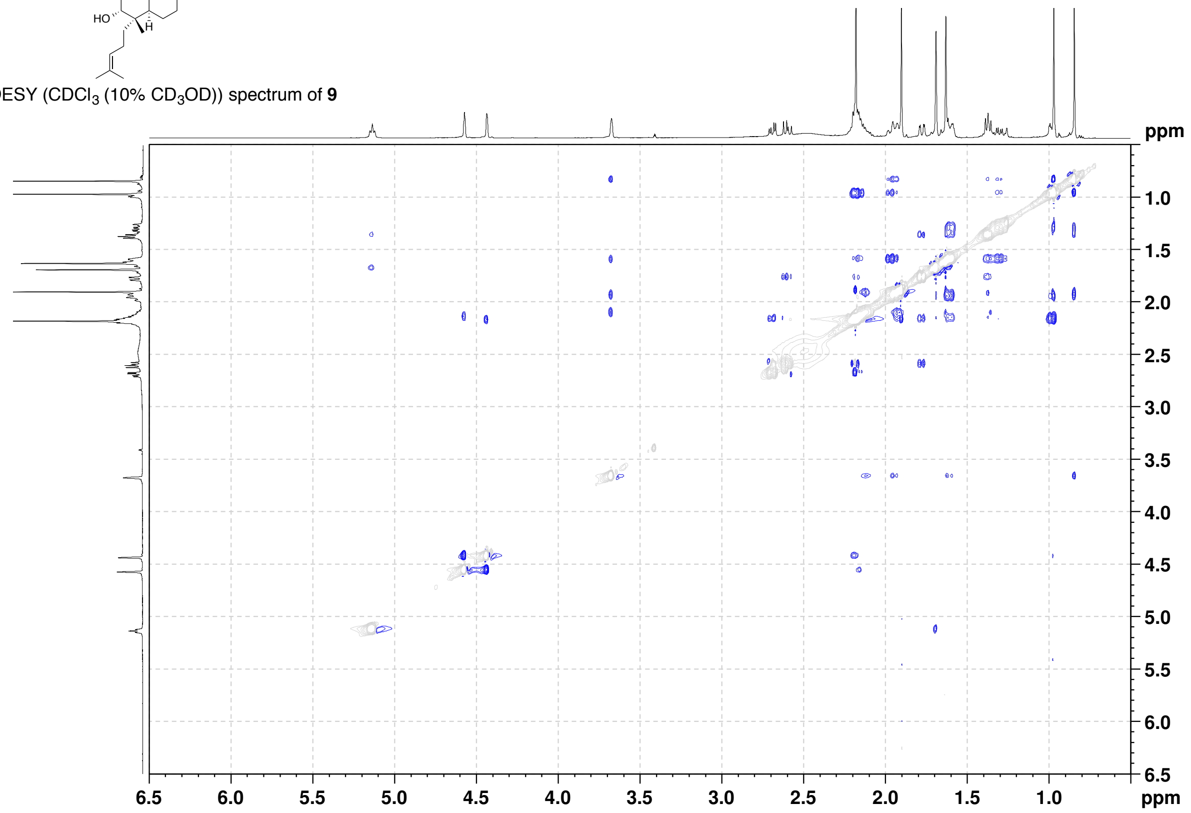

Supplementary Fig. 59. NMR spectra of 9 ($CDCl_3$ (10% CD_3OD))

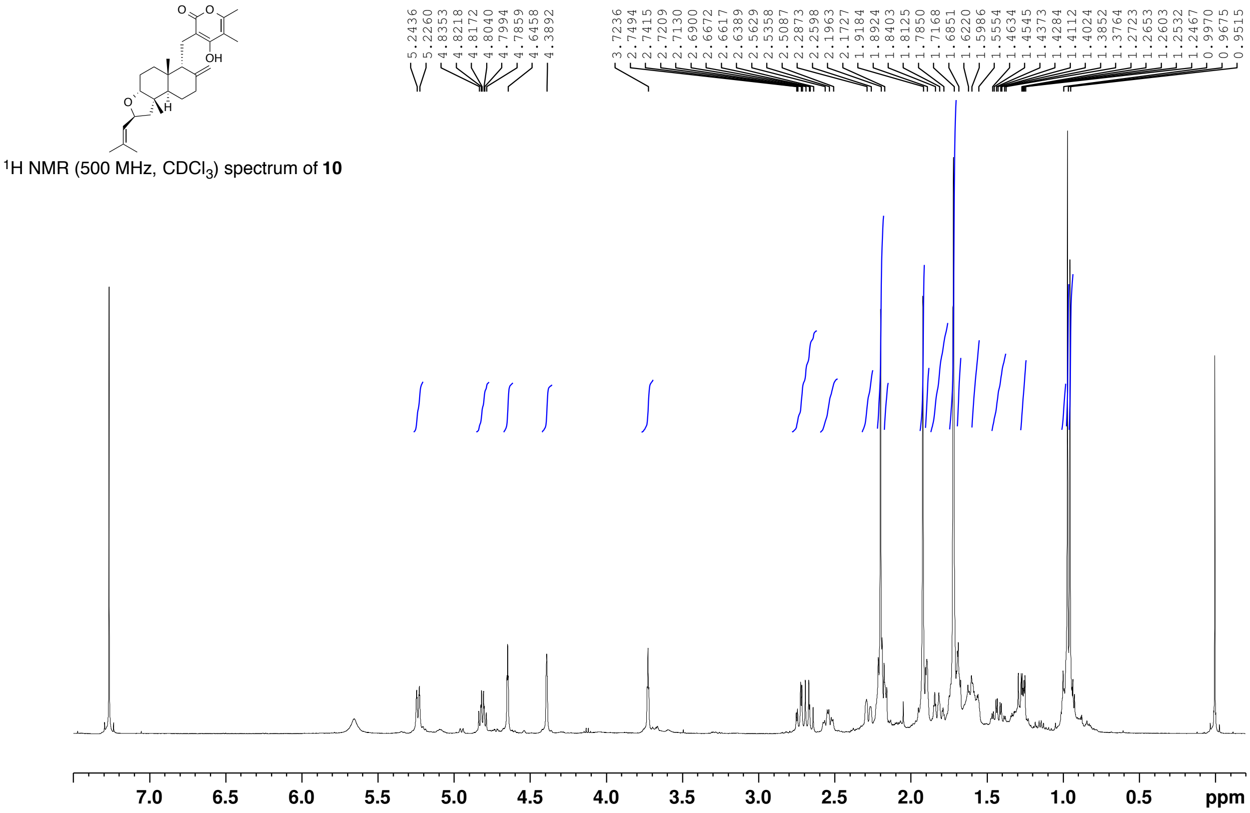

^{1}H NMR (500 MHz, $CDCl_3$) spectrum of **10**

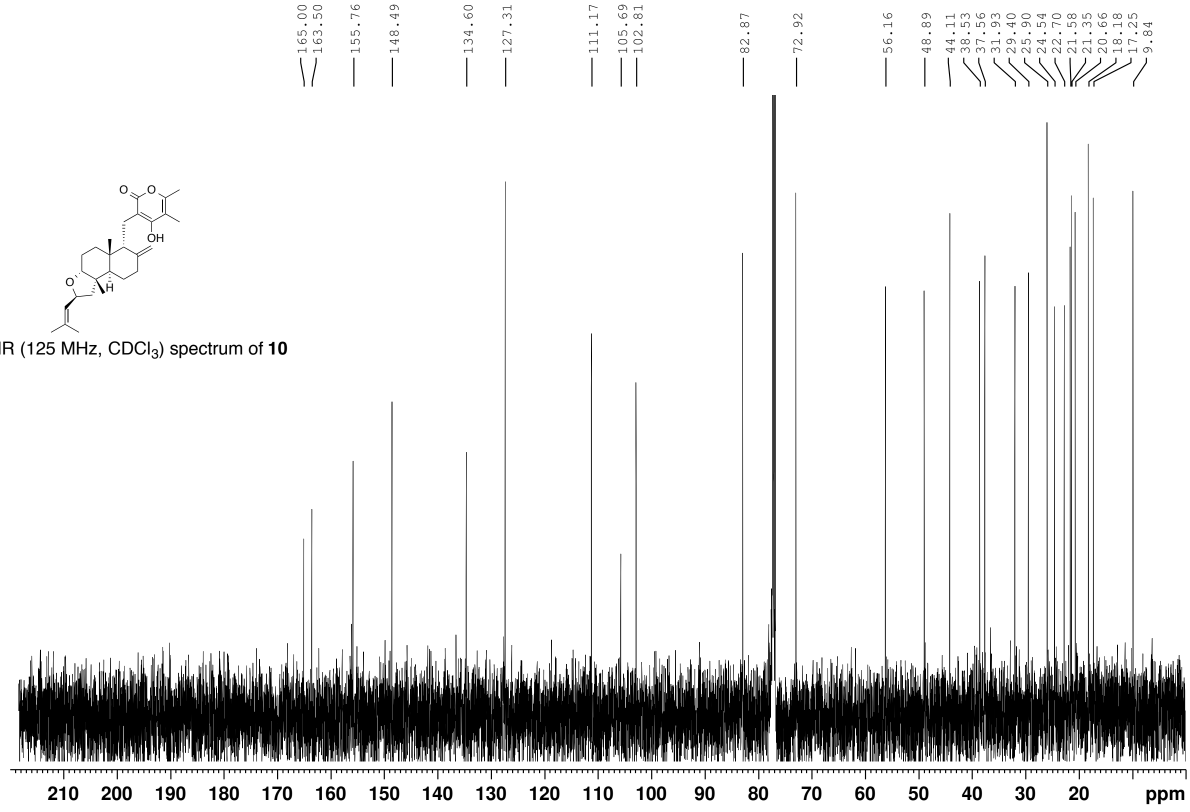

^{13}C NMR (125 MHz, $CDCl_3$) spectrum of **10**

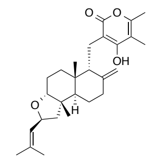

^1H-^1H COSY ($CDCl_3$) spectrum of **10**

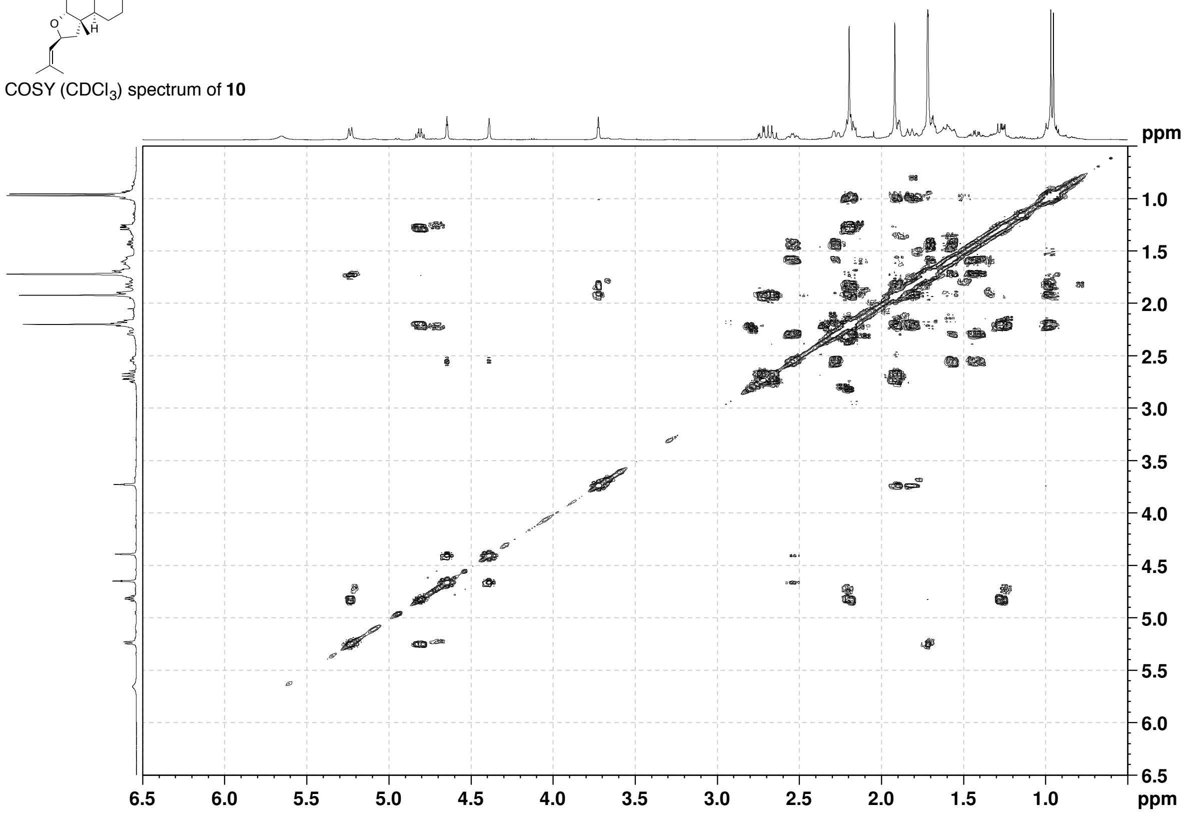

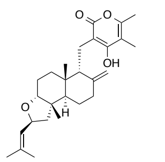

HMBC ($CDCl_3$) spectrum of **10**

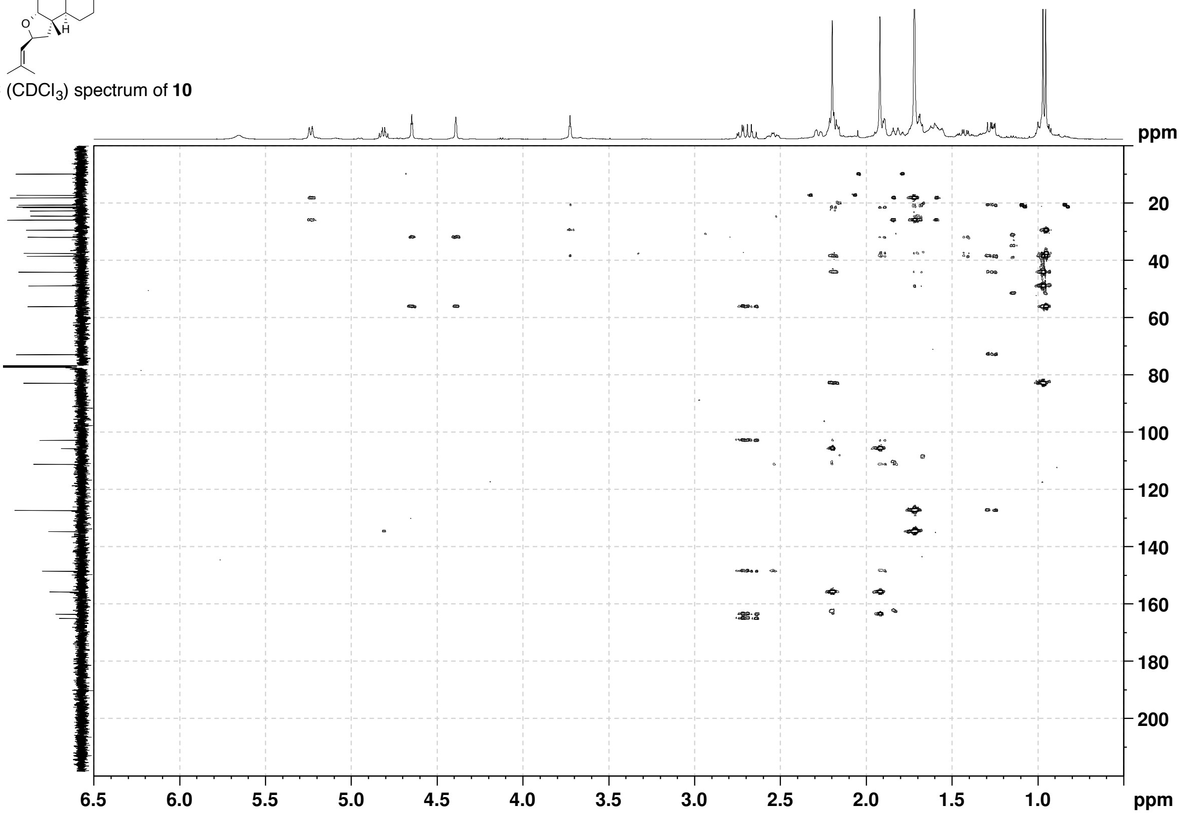

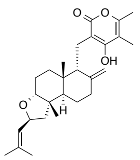

HSQC ($CDCl_3$) spectrum of **10**

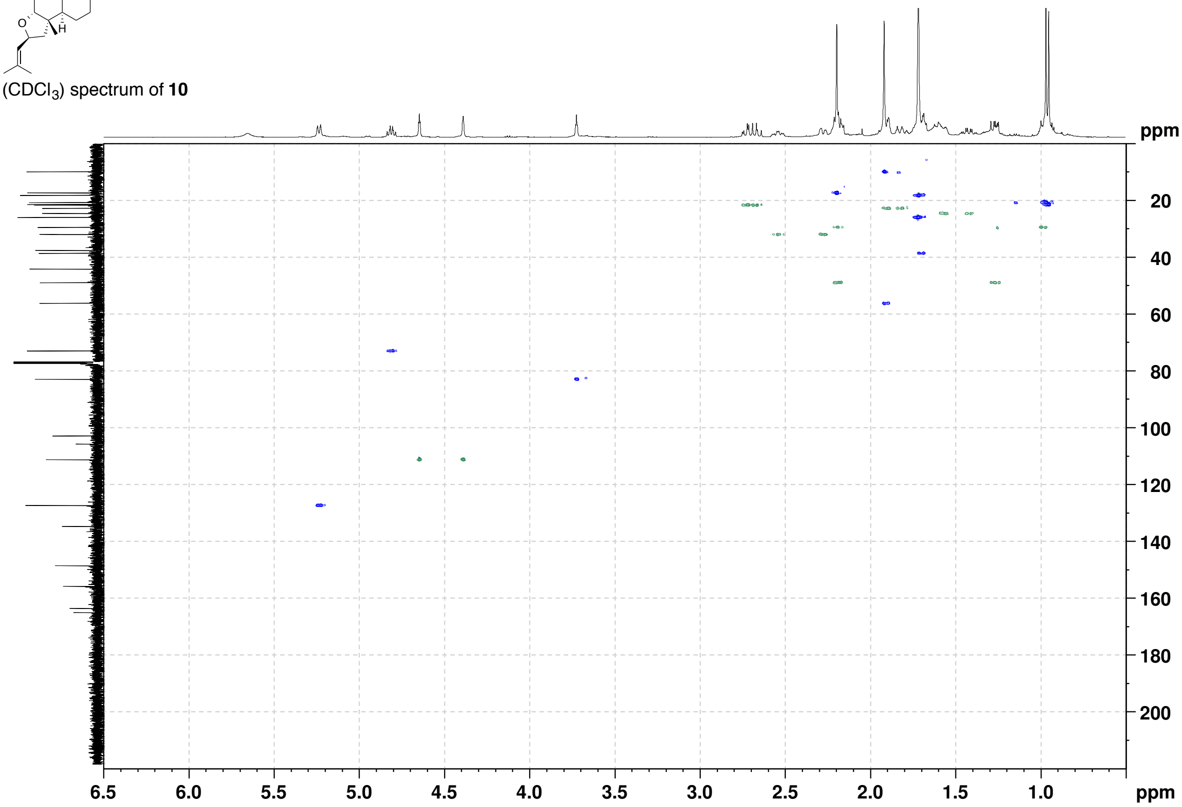

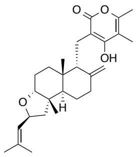

NOESY ($CDCl_3$) spectrum of **10**

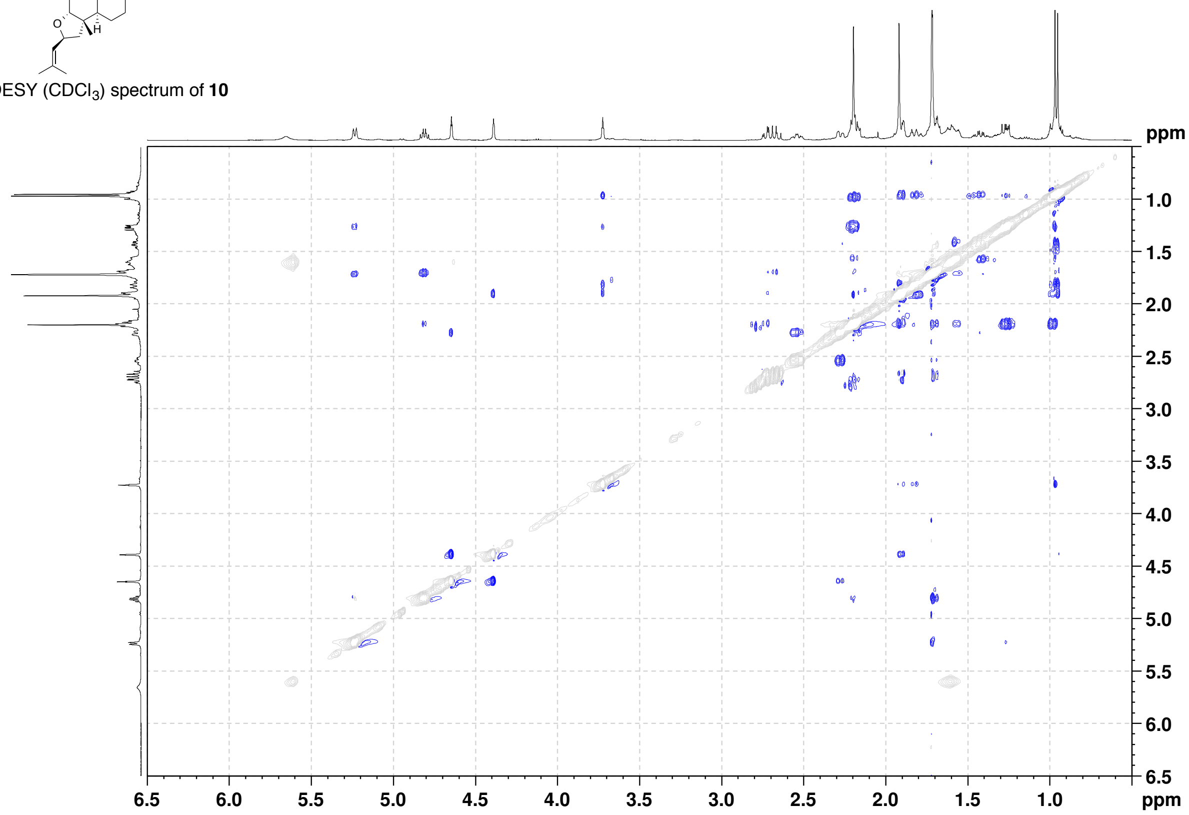

Supplementary Fig. 60. NMR spectra of 10 ($CDCl_3$)

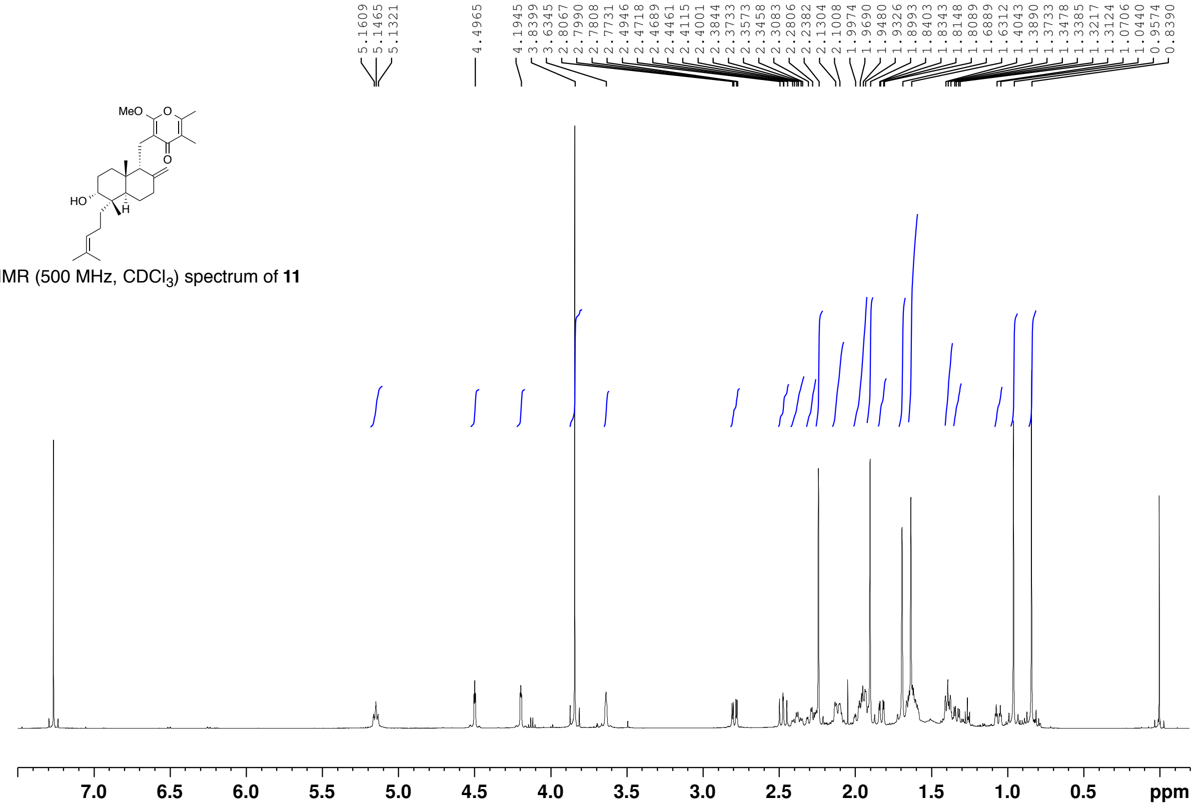

^{1}H NMR (500 MHz, $CDCl_3$) spectrum of **11**

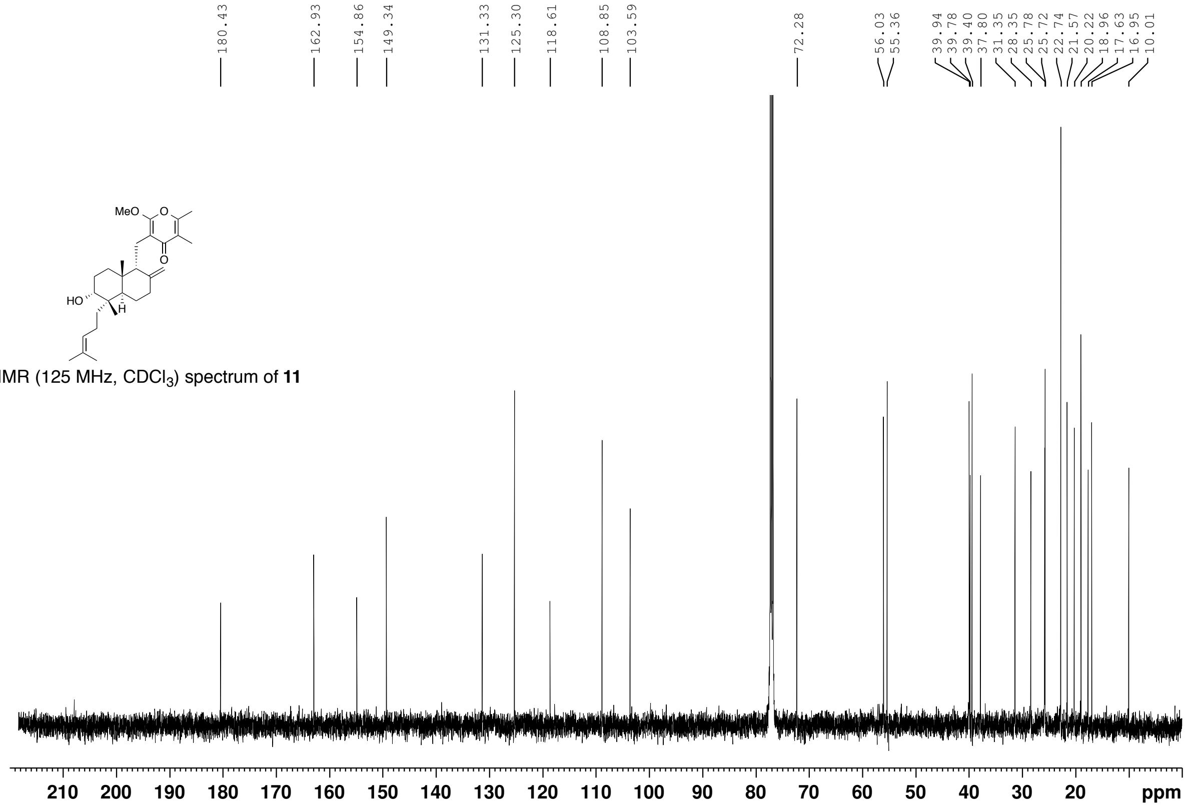

^{13}C NMR (125 MHz, $CDCl_3$) spectrum of **11**

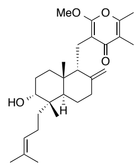

^1H-^1H COSY ($CDCl_3$) spectrum of **11**

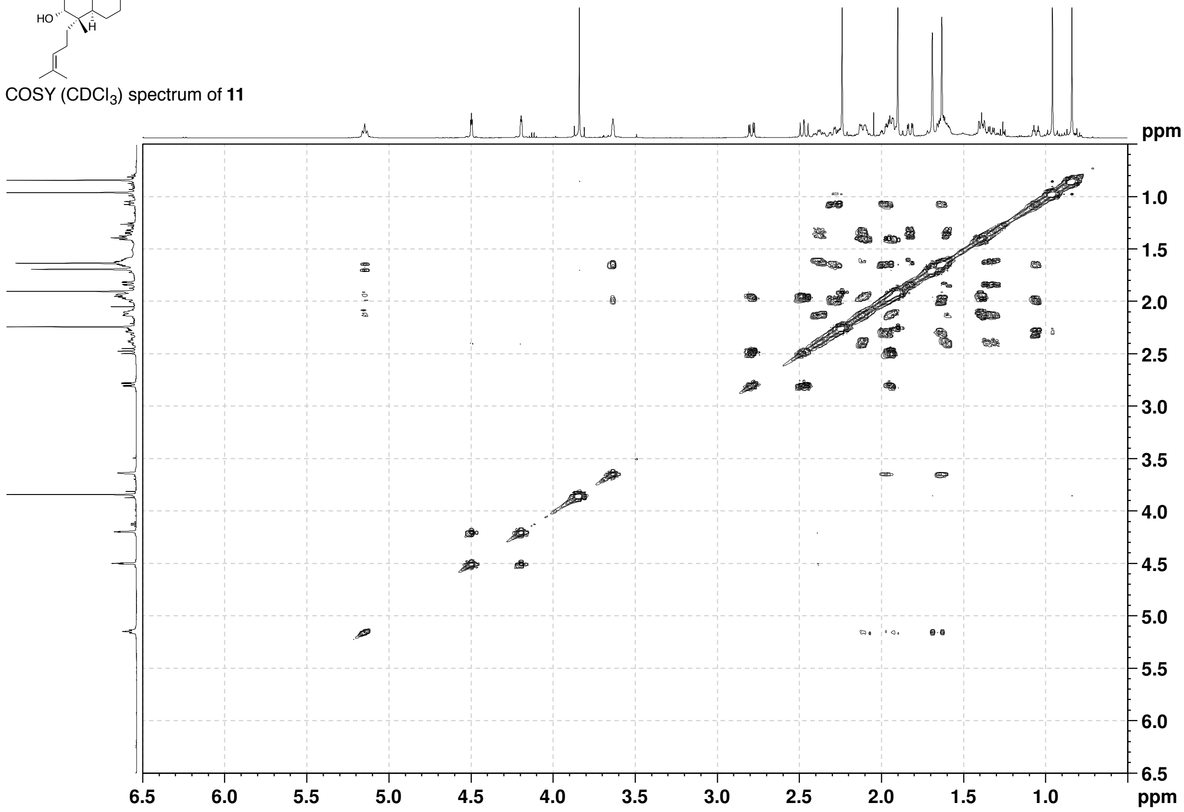

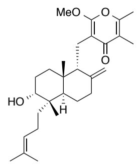

HMBC ($CDCl_3$) spectrum of **11**

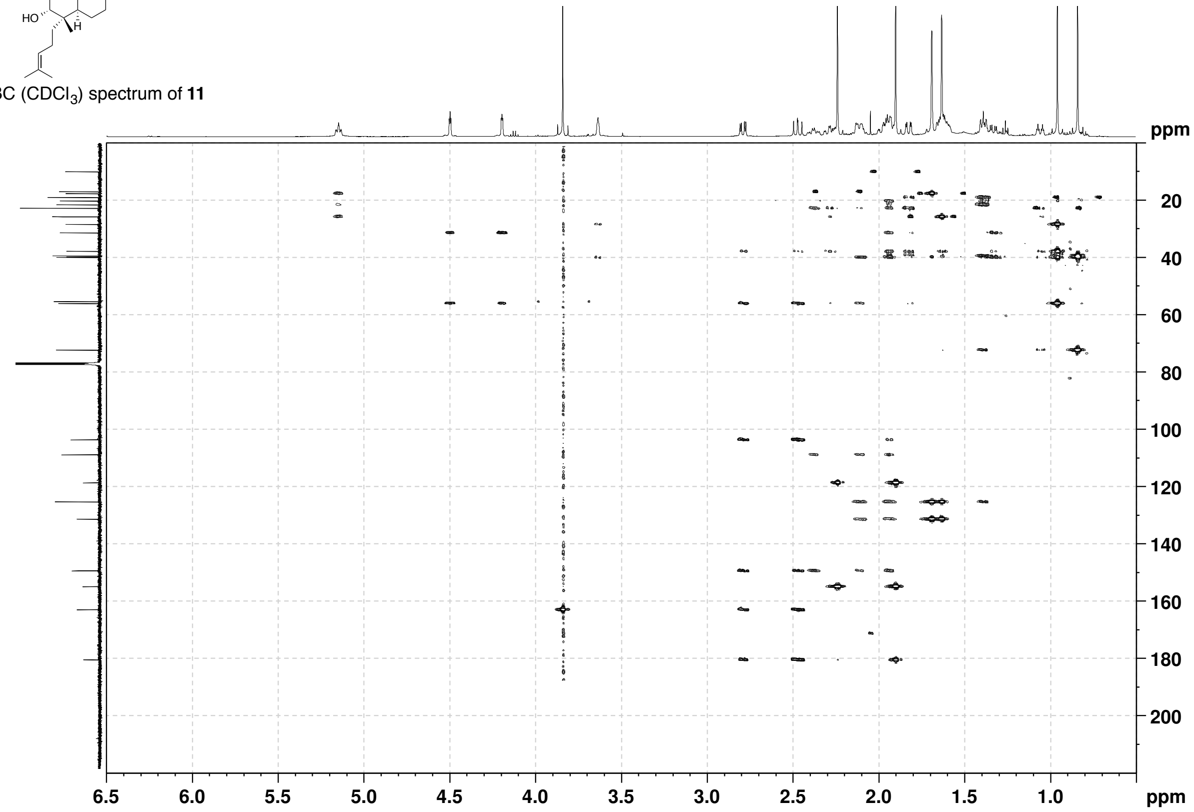

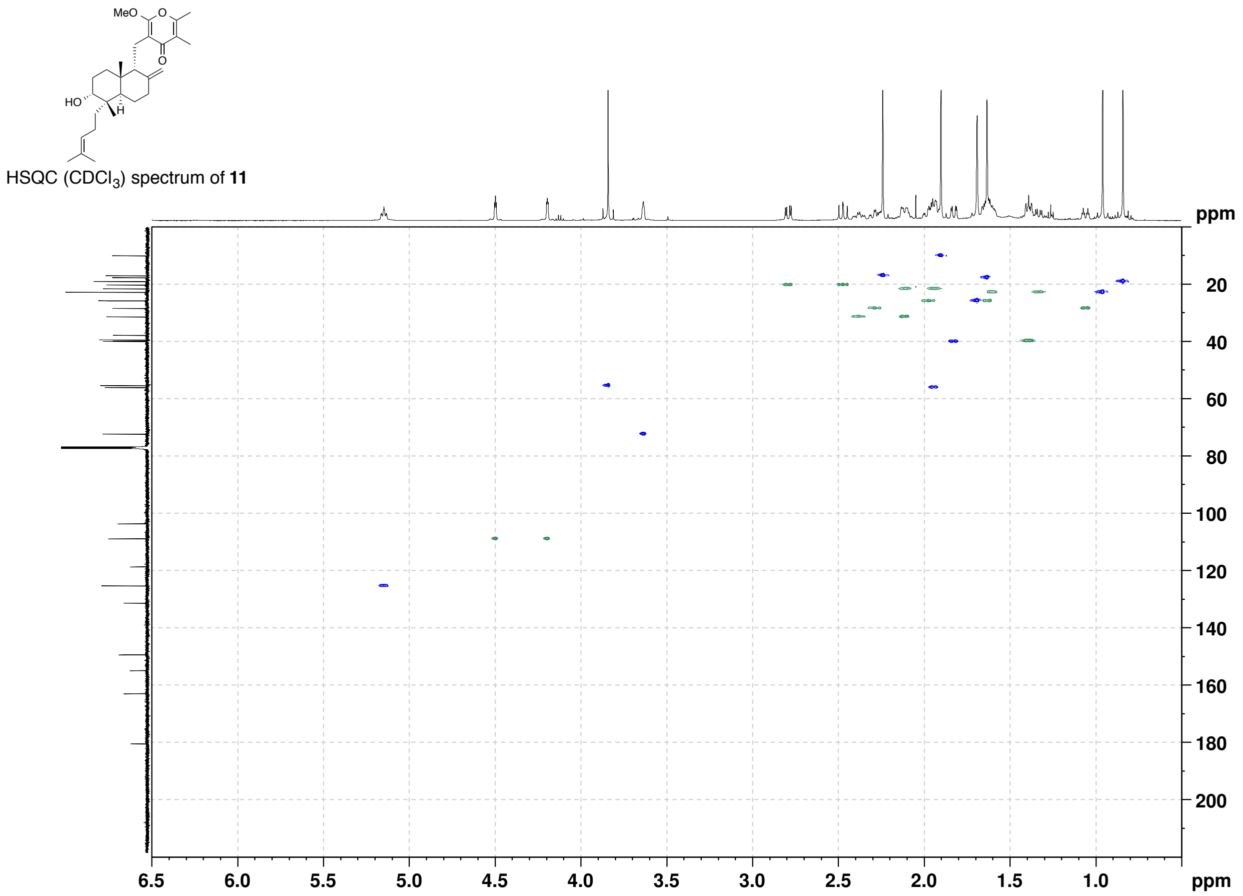

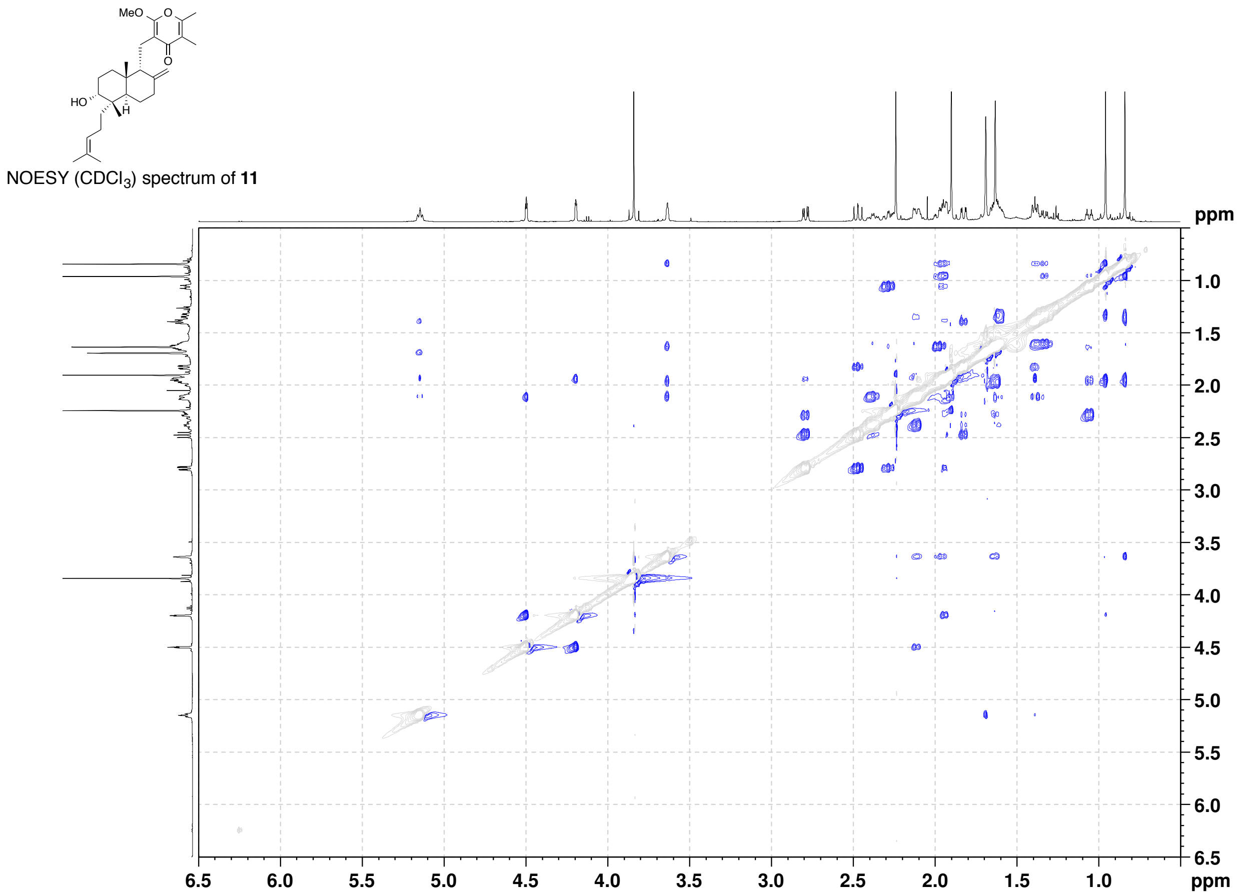

Supplementary Fig. 61. NMR spectra of 11 ($CDCl_3$)

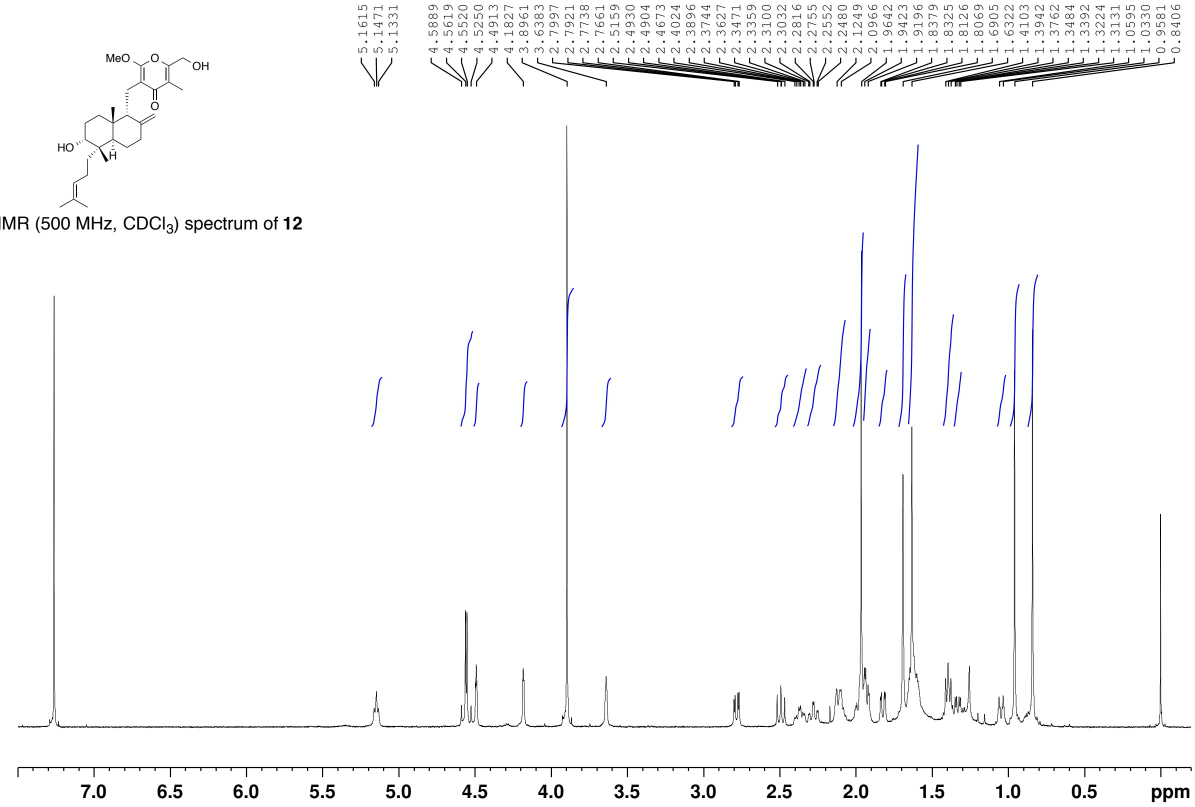

^{1}H NMR (500 MHz, $CDCl_3$) spectrum of **12**

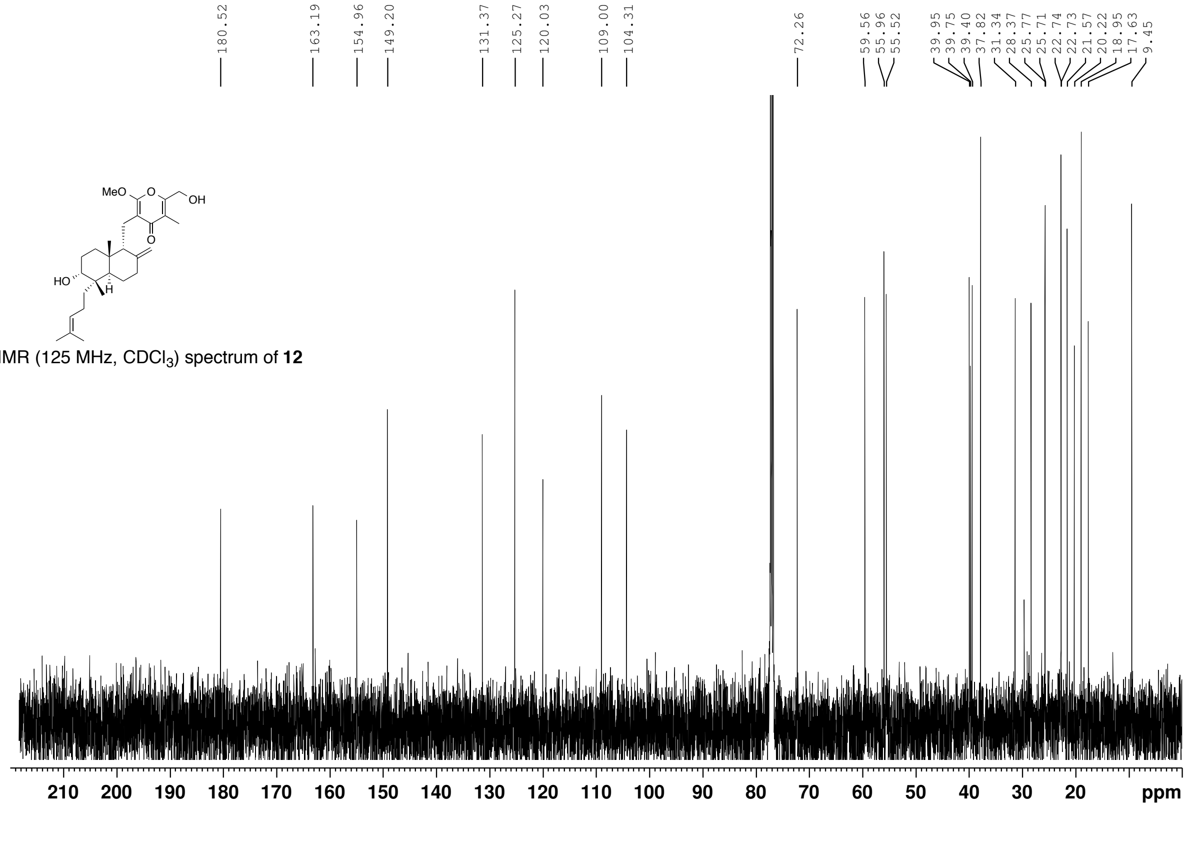

^{13}C NMR (125 MHz, $CDCl_3$) spectrum of **12**

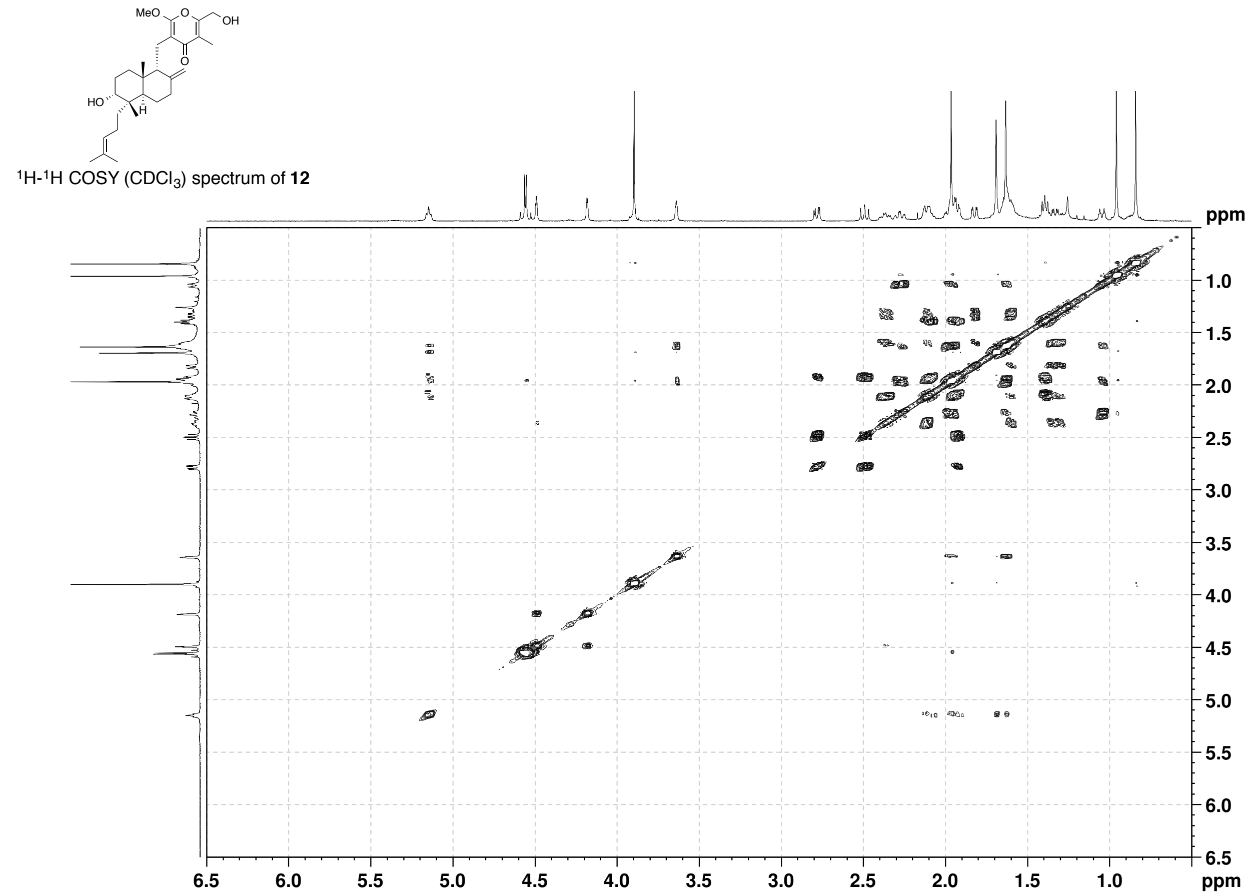

^{1}H-^{1}H COSY ($CDCl_3$) spectrum of **12**

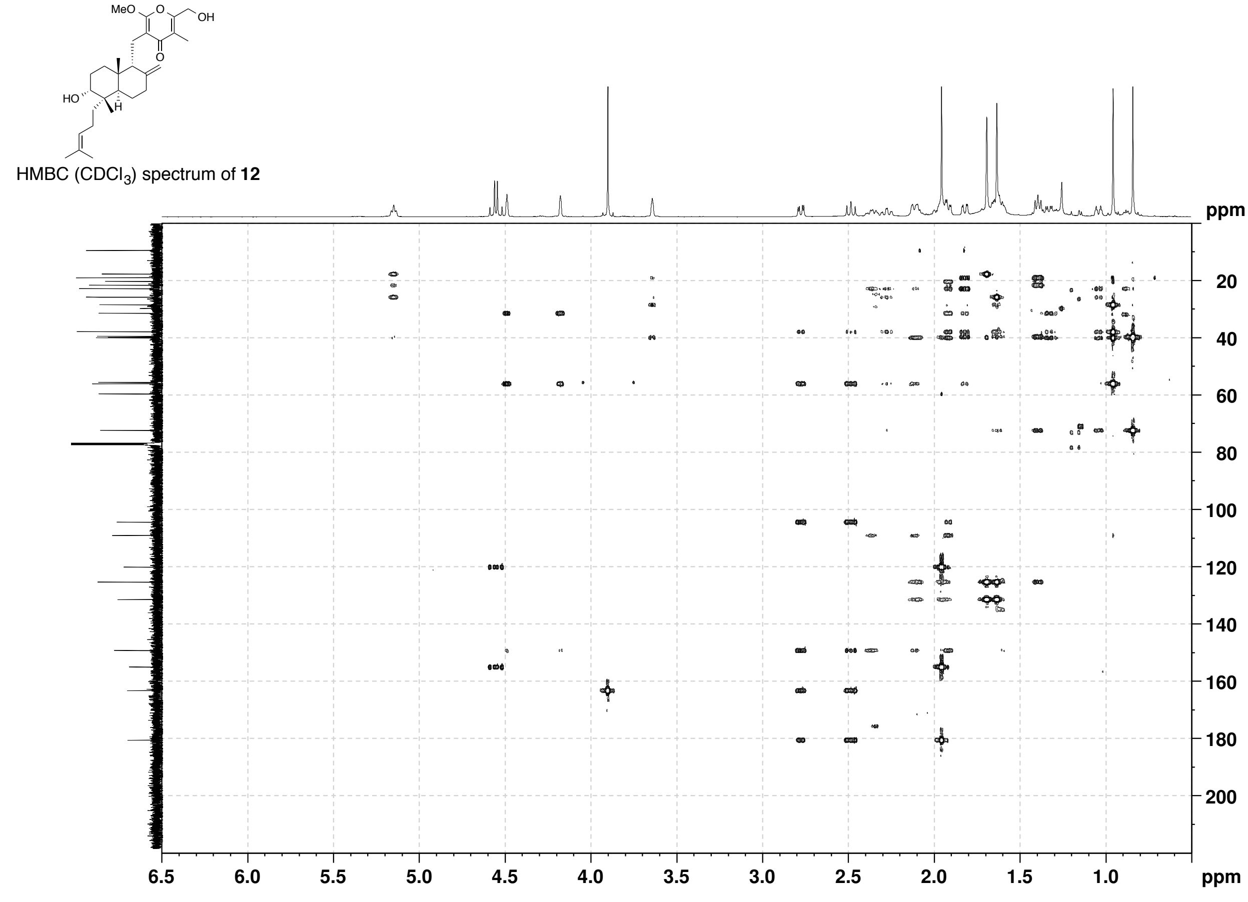

HMBC ($CDCl_3$) spectrum of **12**

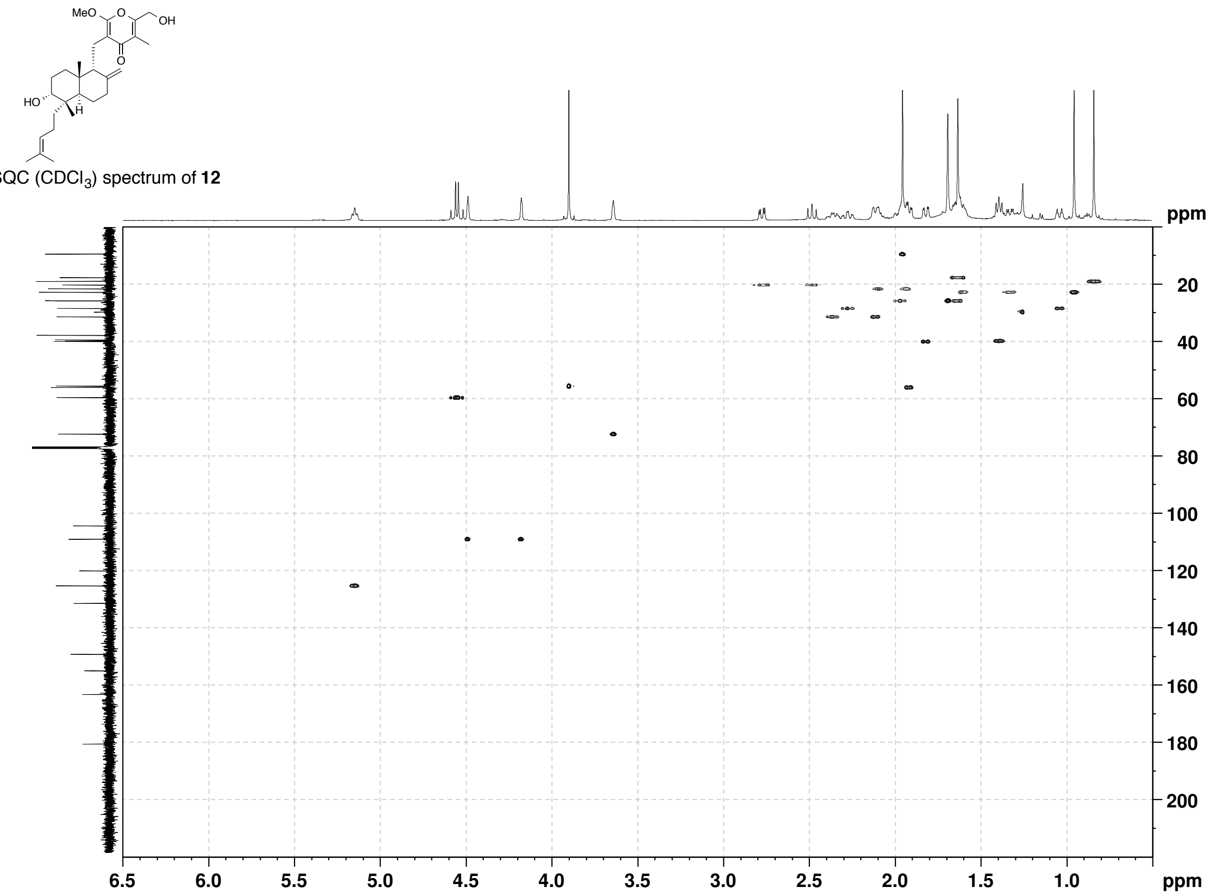

HSQC ($CDCl_3$) spectrum of **12**

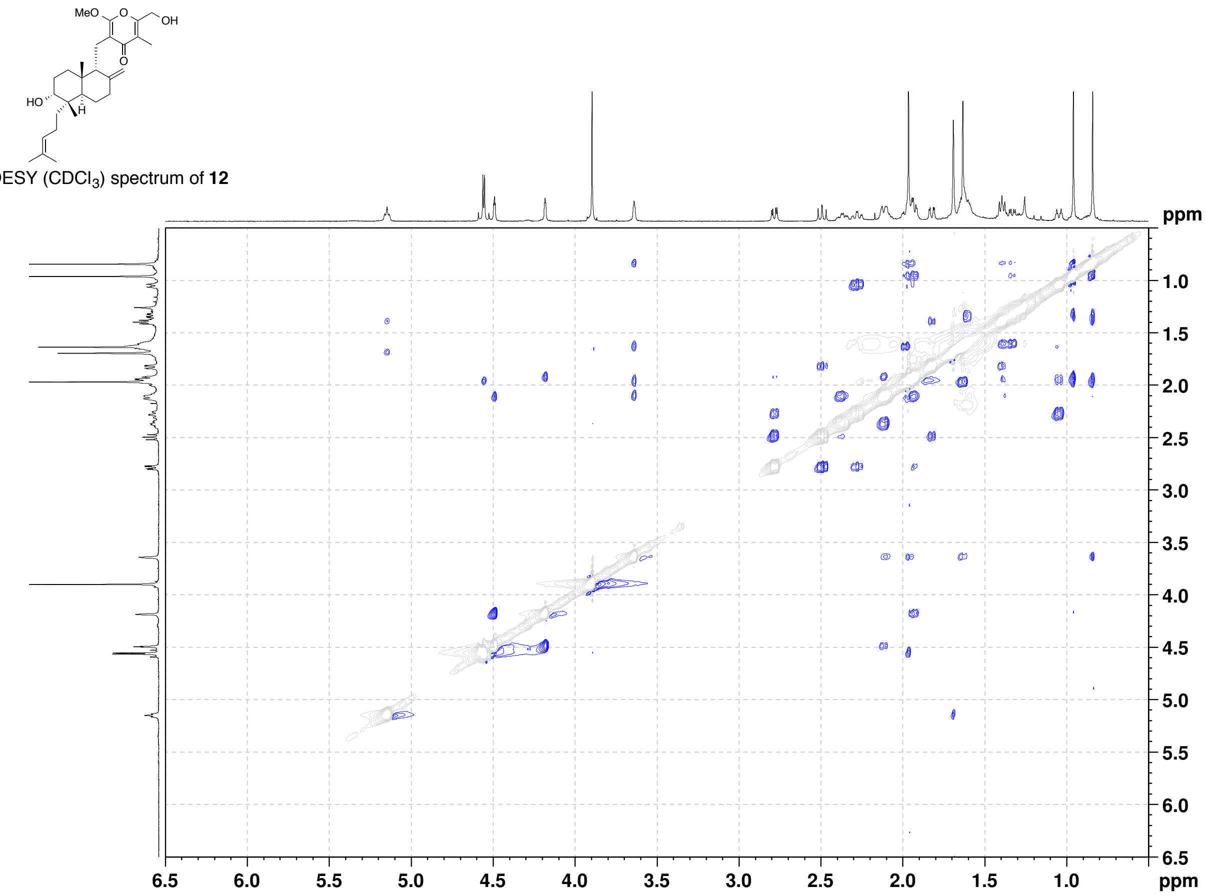

NOESY ($CDCl_3$) spectrum of **12**

Supplementary Fig. 62. NMR spectra of 12 ($CDCl_3$)

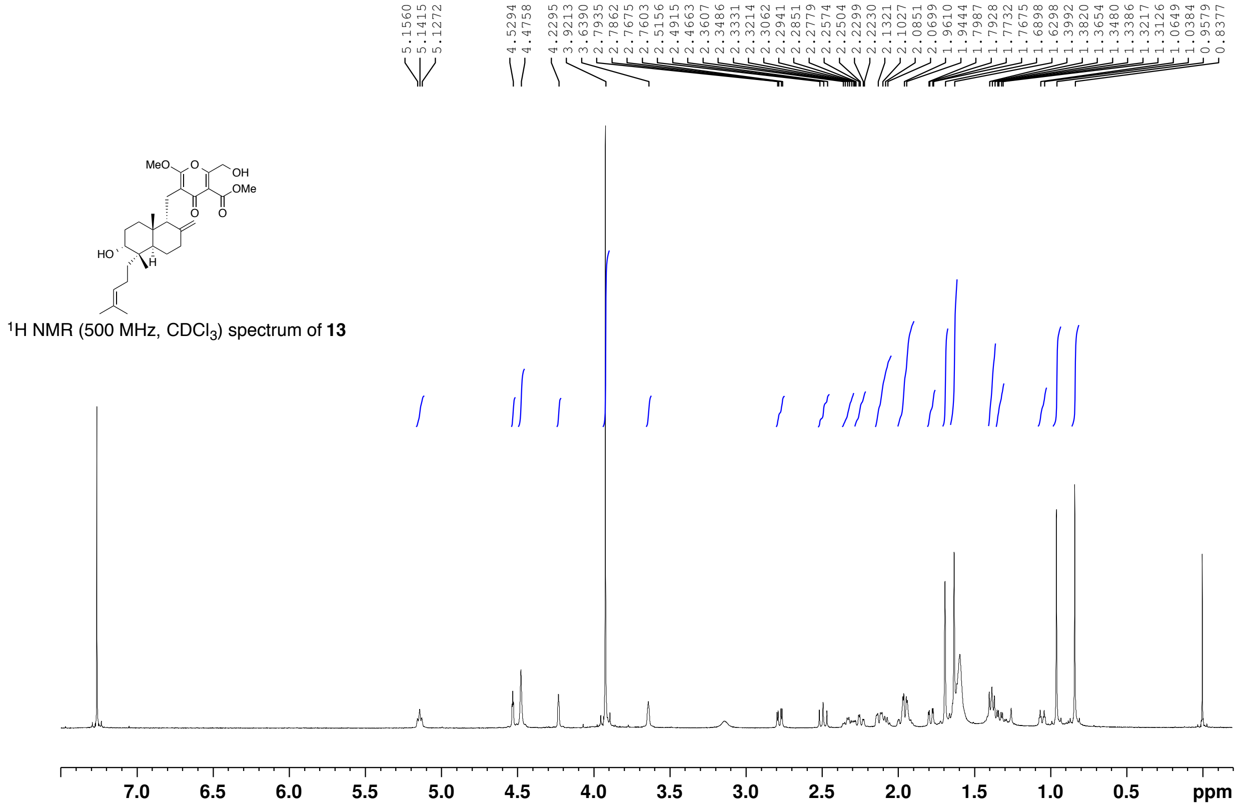

^{1}H NMR (500 MHz, $CDCl_3$) spectrum of **13**

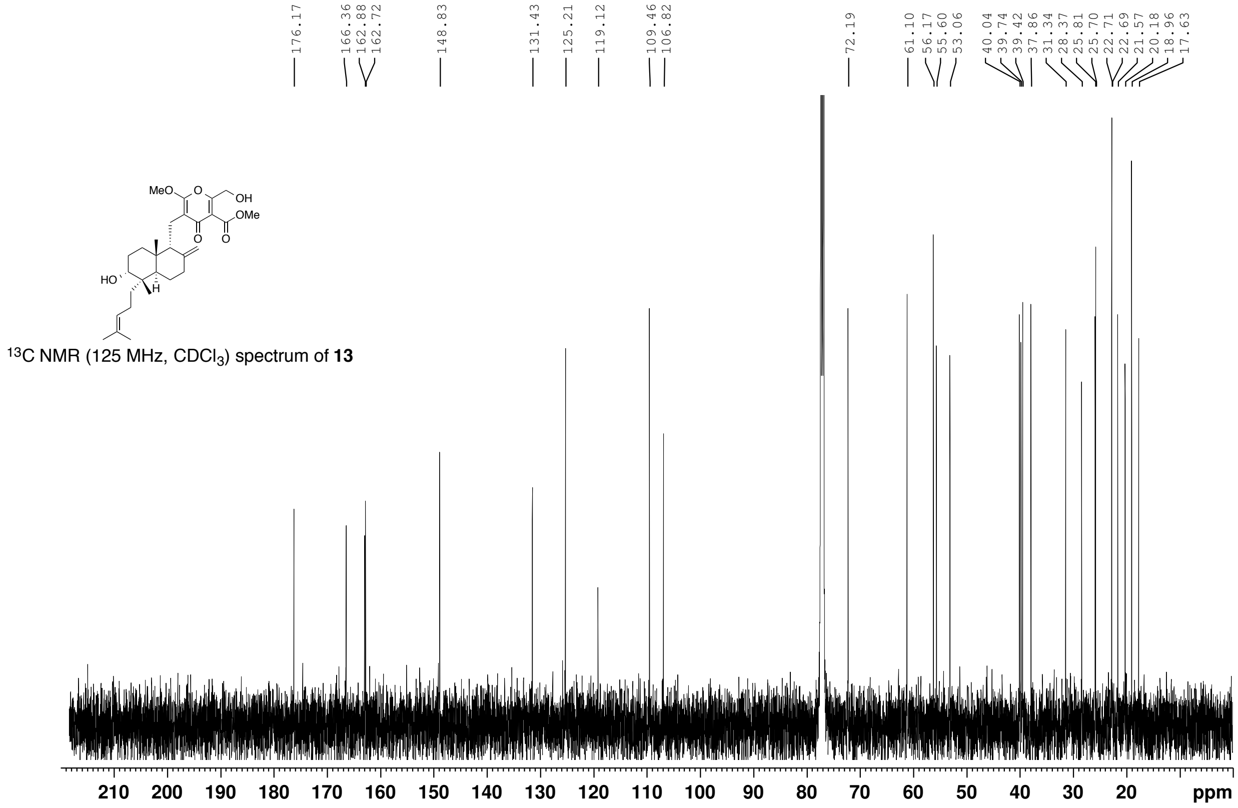

^{13}C NMR (125 MHz, $CDCl_3$) spectrum of **13**

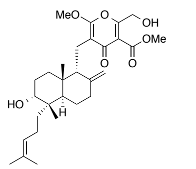

^{1}H-^{1}H COSY ($CDCl_3$) spectrum of **13**

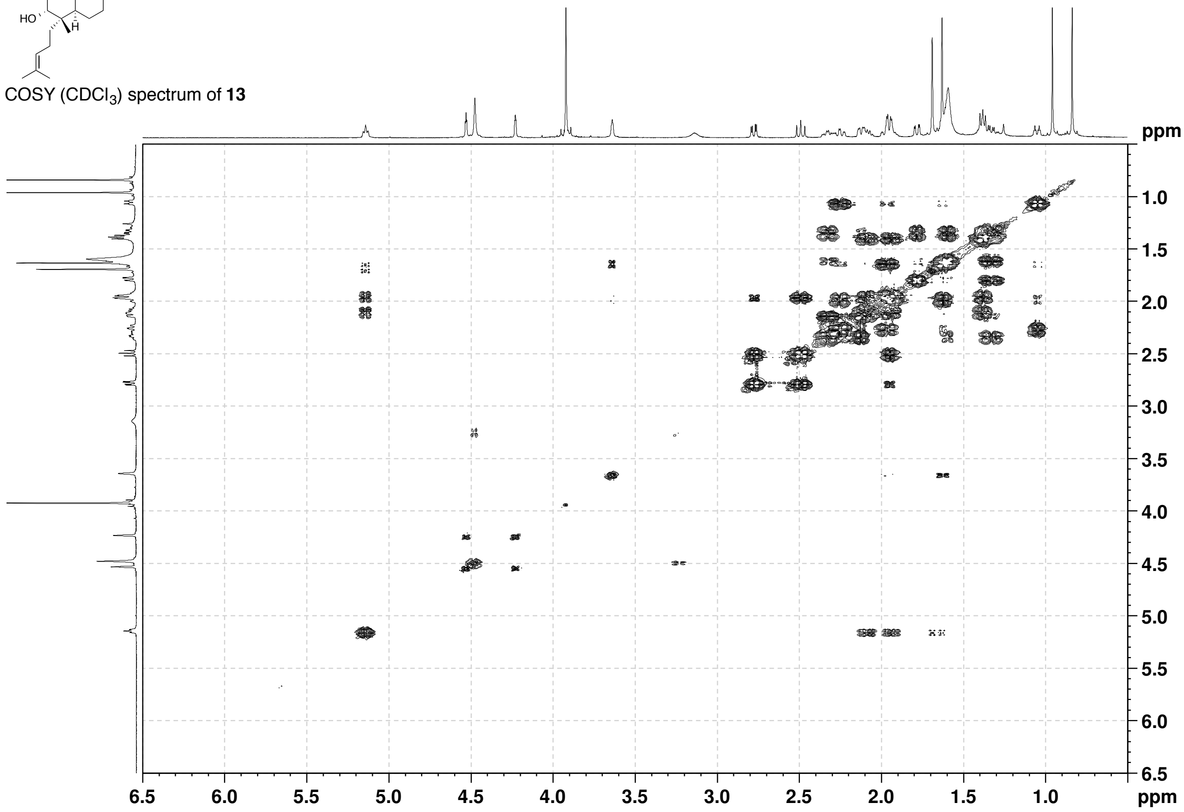

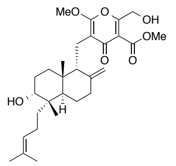

HMBC ($CDCl_3$) spectrum of **13**

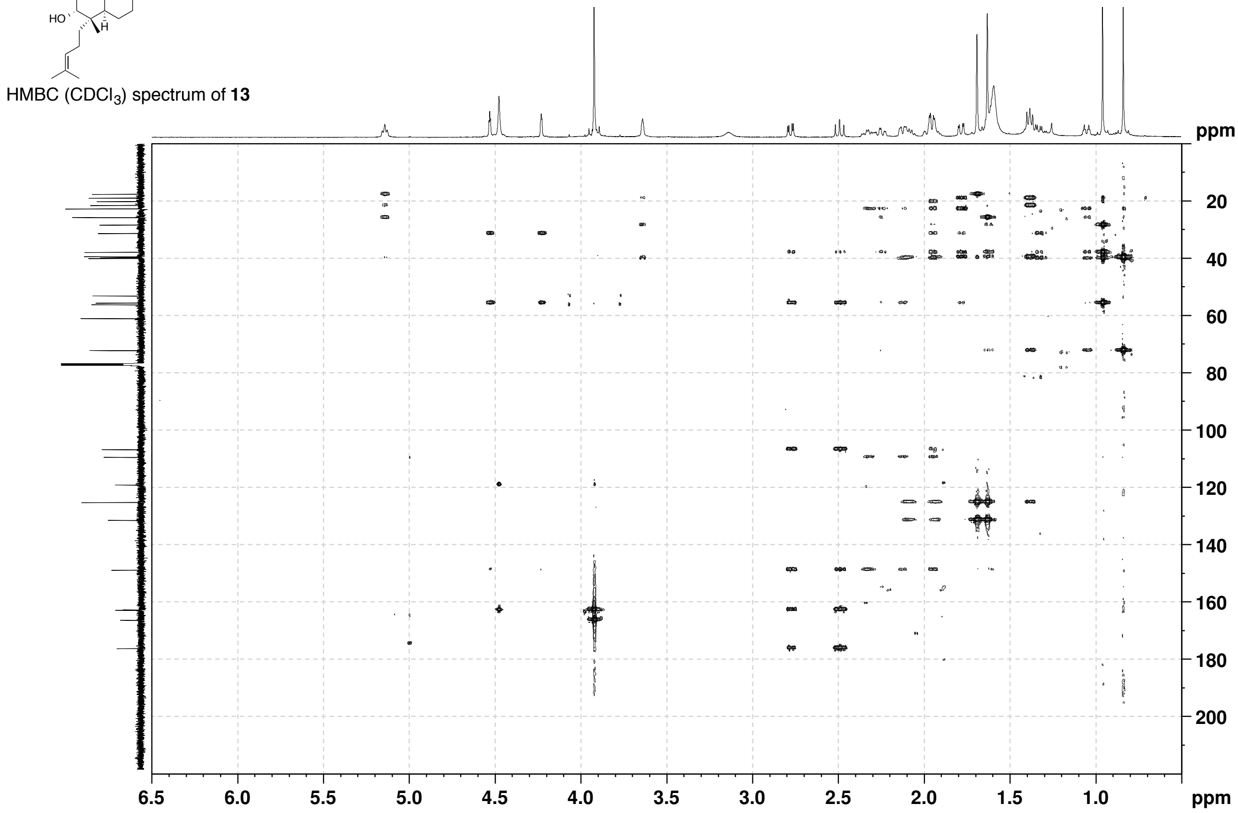

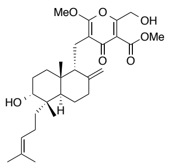

HSQC ($CDCl_3$) spectrum of **13**

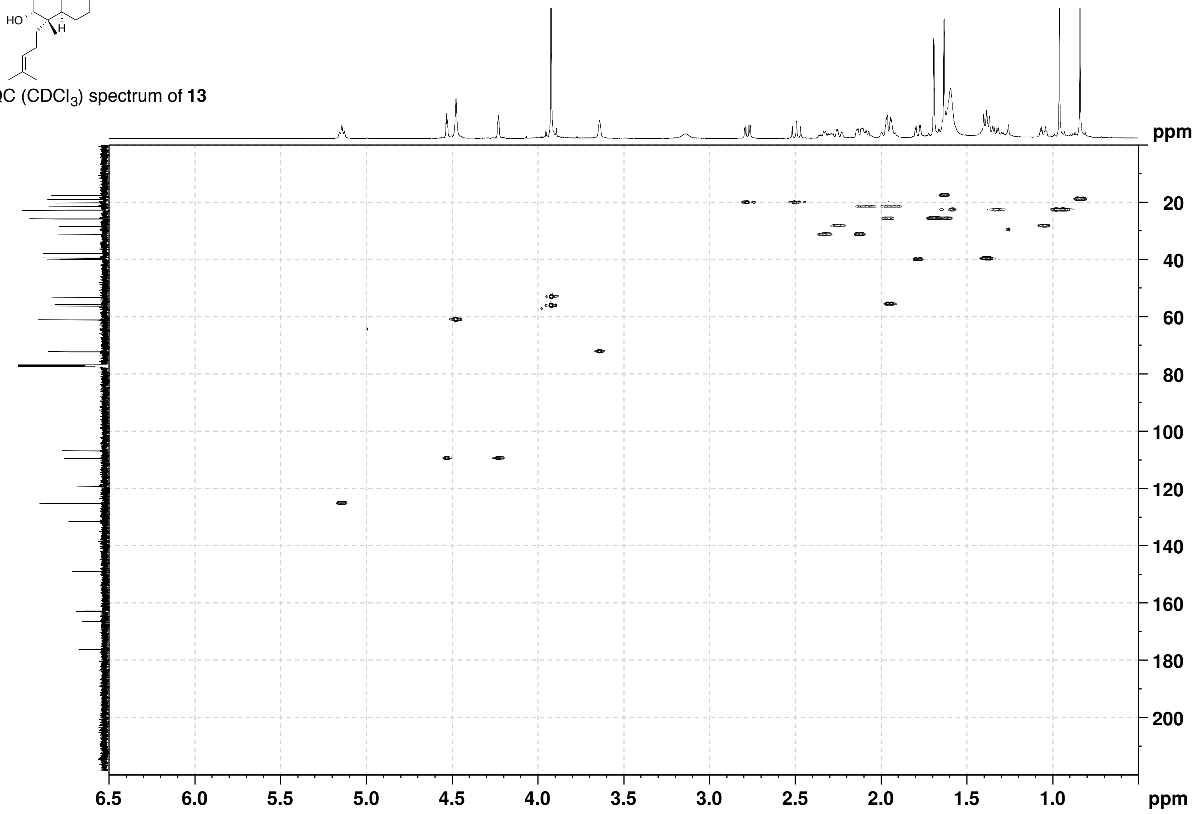

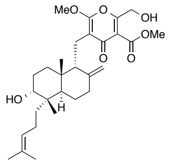

NOESY ($CDCl_3$) spectrum of **13**

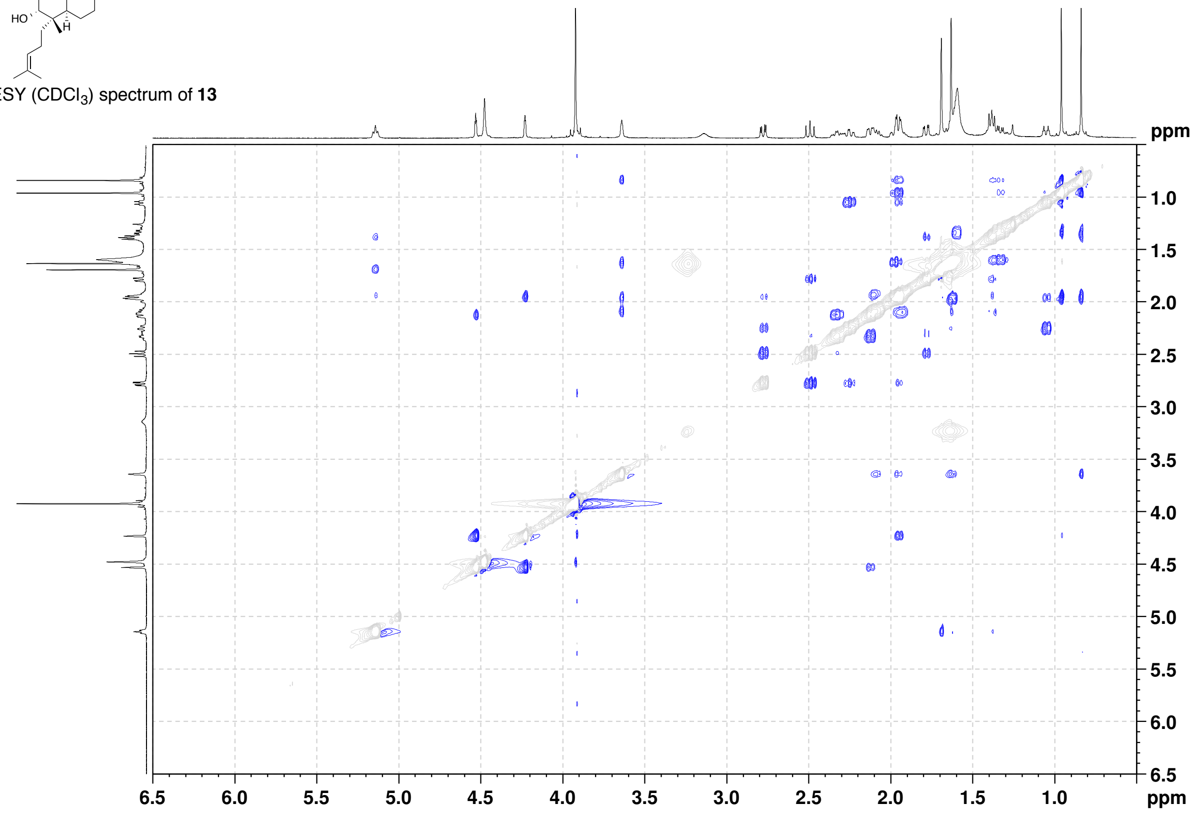

Supplementary Fig. 63. NMR spectra of 13 ($CDCl_3$)

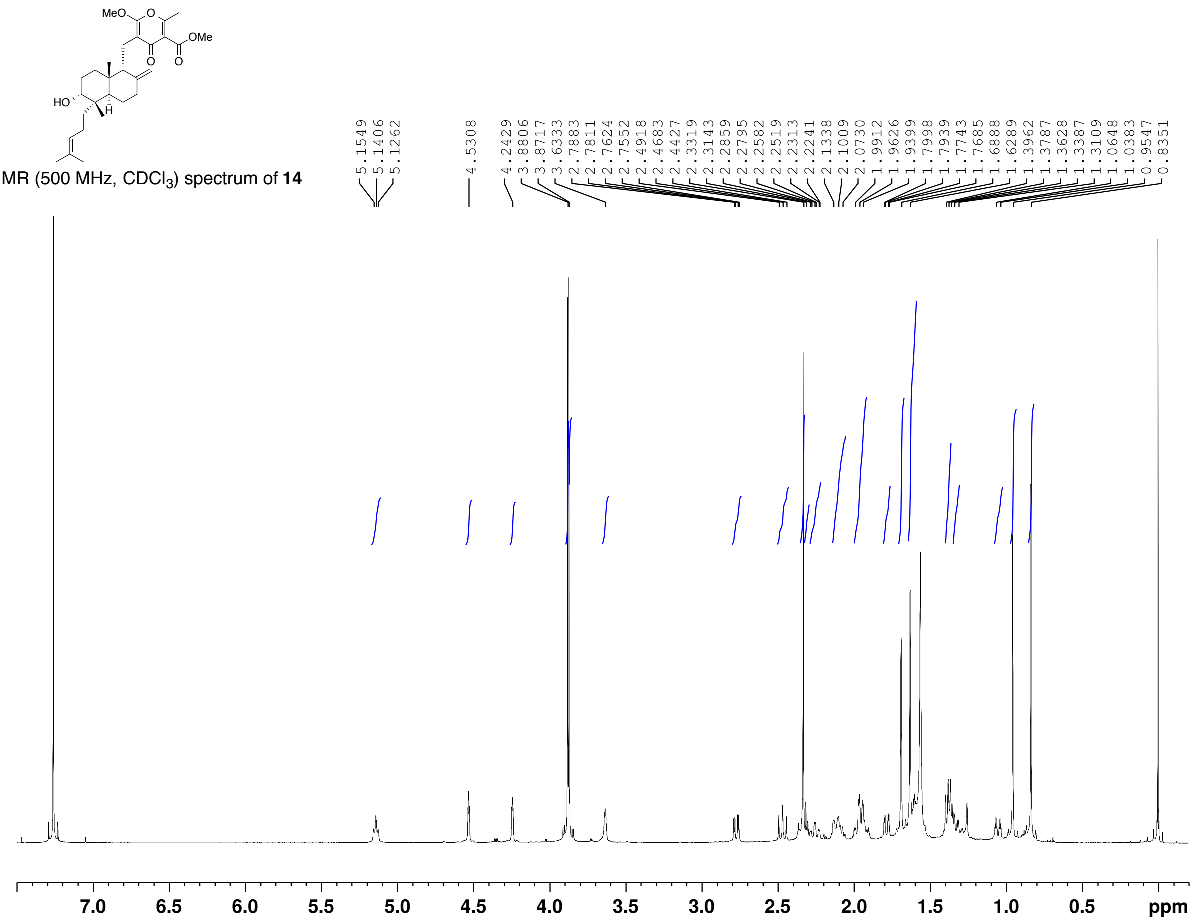

^{1}H NMR (500 MHz, $CDCl_3$) spectrum of **14**

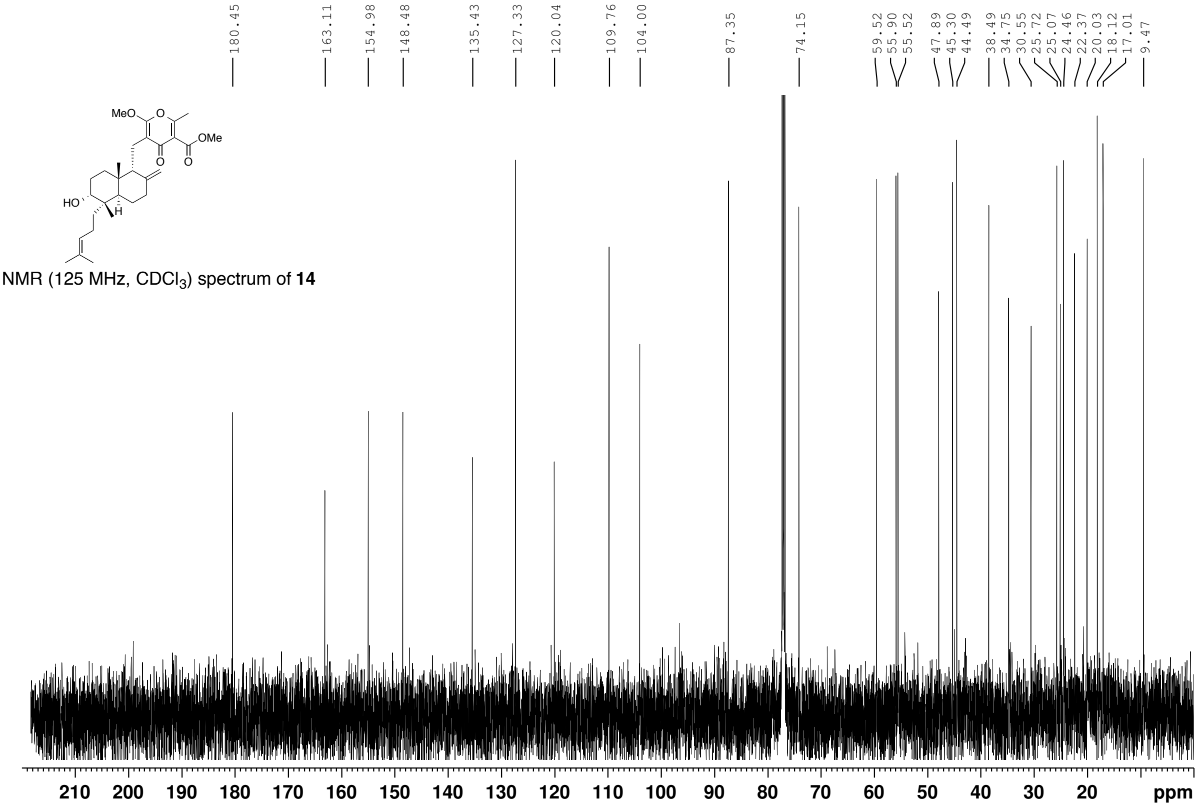

^{13}C NMR (125 MHz, $CDCl_3$) spectrum of **14**

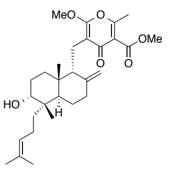

^{1}H-^{1}H COSY ($CDCl_3$) spectrum of **14**

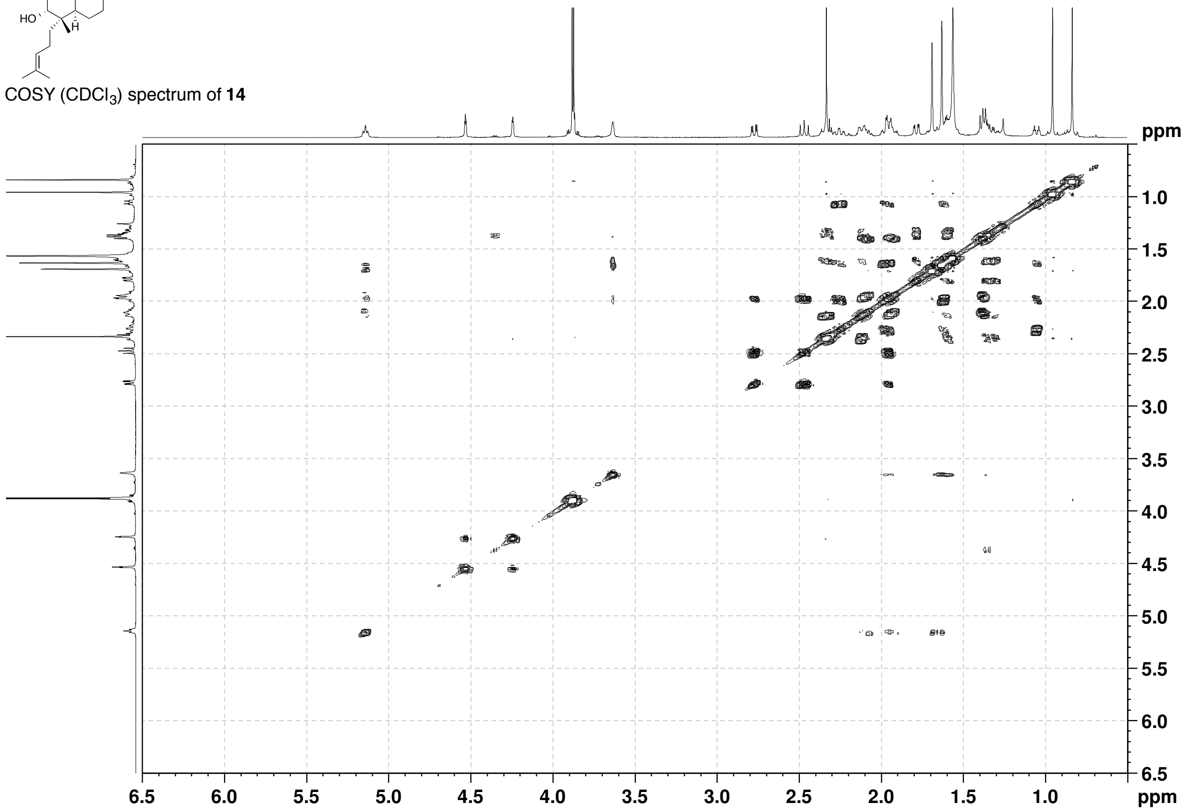

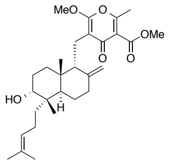

HMBC ($CDCl_3$) spectrum of **14**

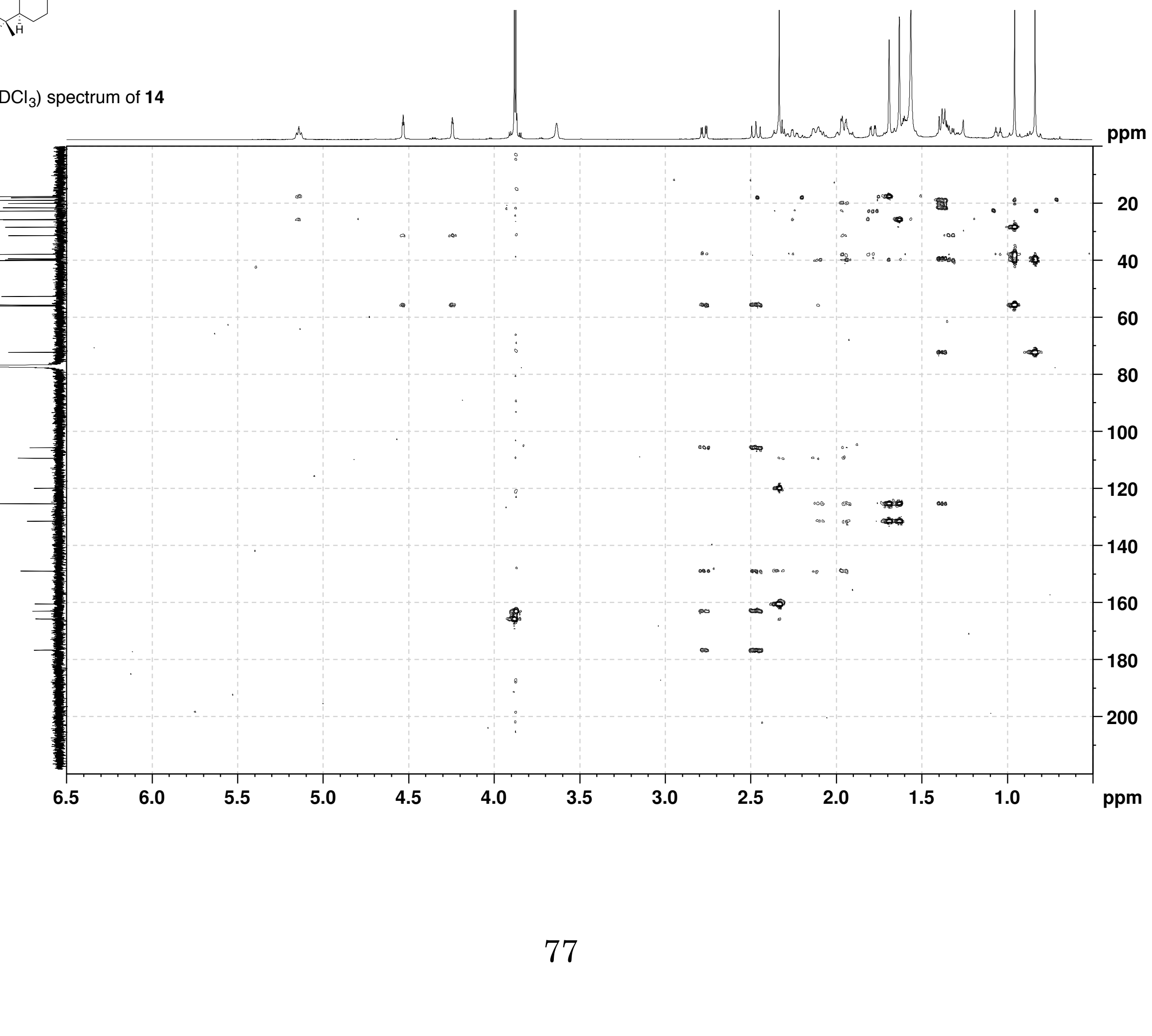

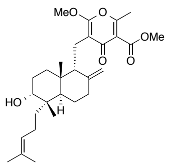

HSQC ($CDCl_3$) spectrum of **14**

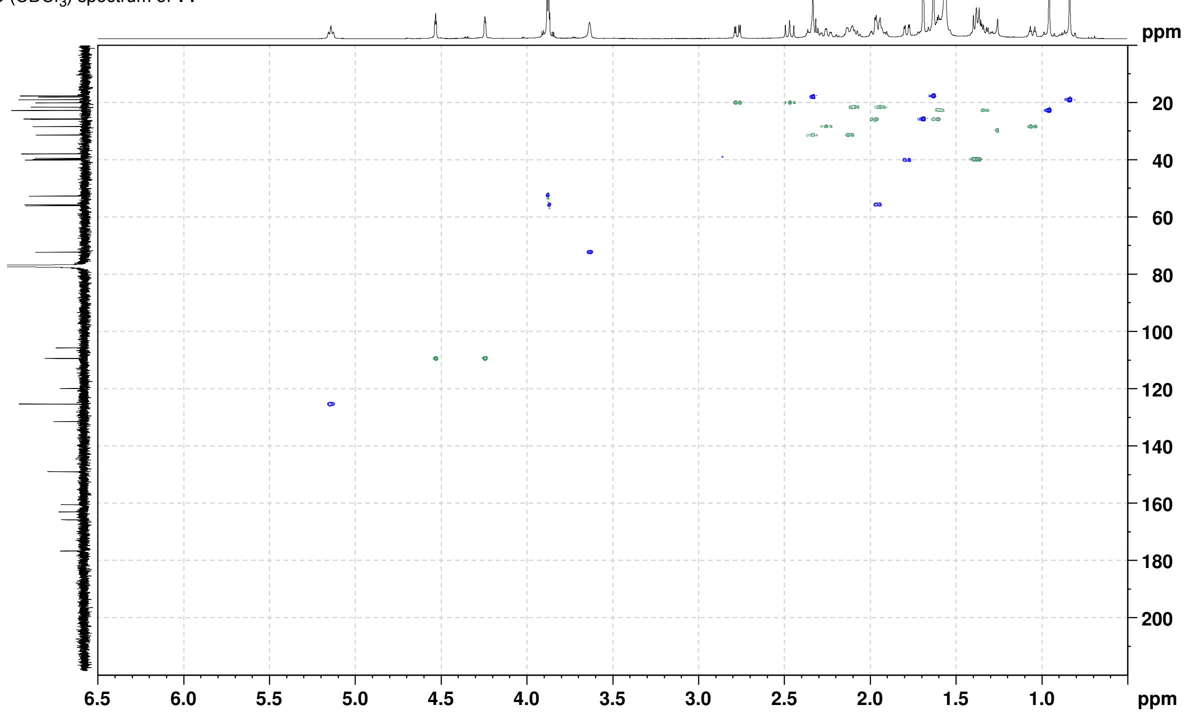

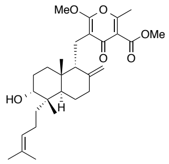

NOESY ($CDCl_3$) spectrum of **14**

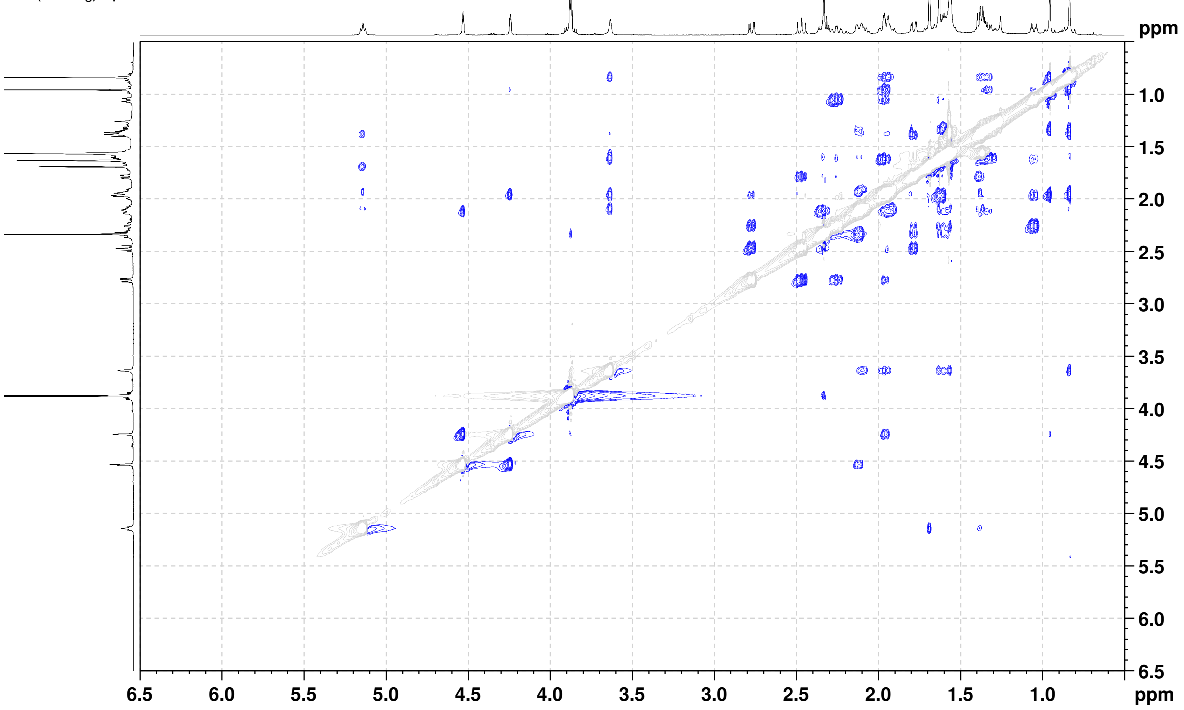

Supplementary Fig. 64. NMR spectra of 14 ($CDCl_3$)

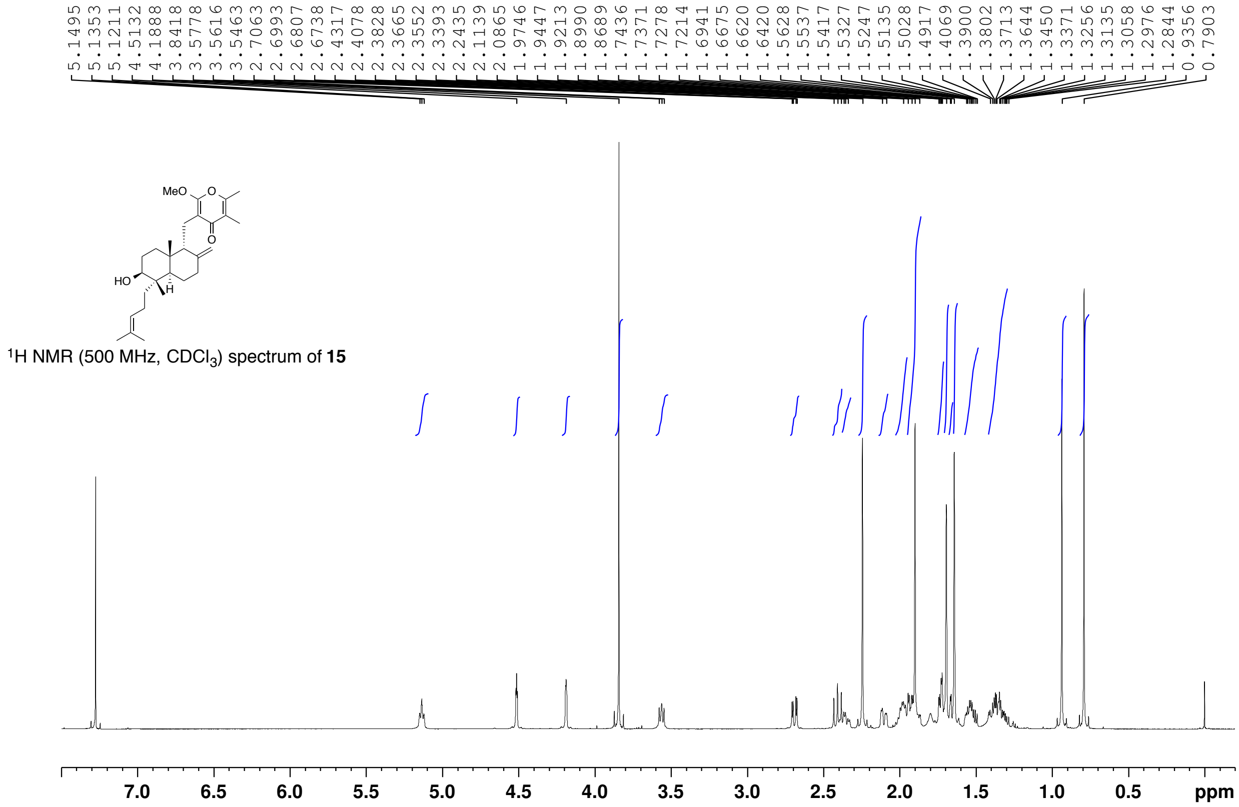

^{1}H NMR (500 MHz, $CDCl_3$) spectrum of **15**

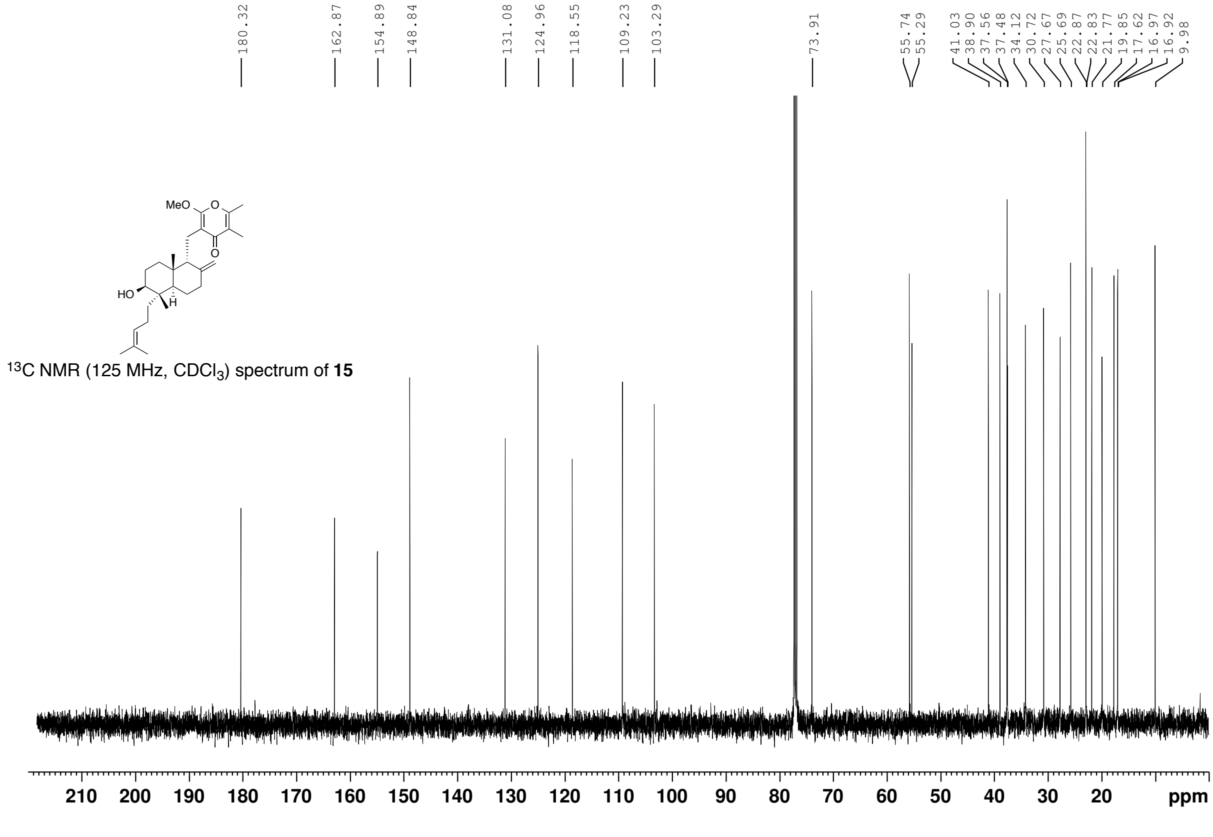

^{13}C NMR (125 MHz, $CDCl_3$) spectrum of **15**

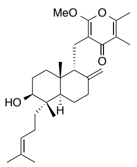

^{1}H-^{1}H COSY ($CDCl_3$) spectrum of **15**

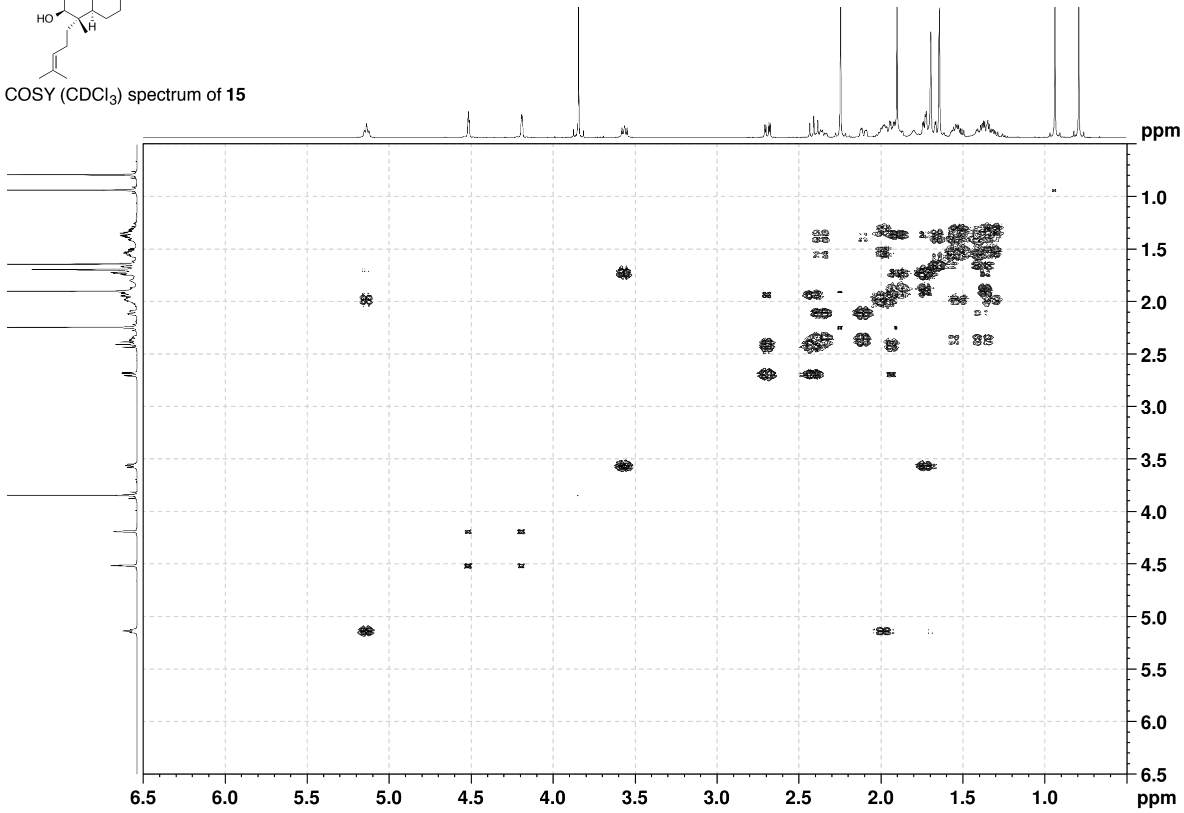

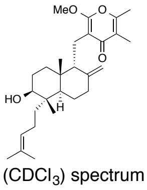

HMBC ($CDCl_3$) spectrum of **15**

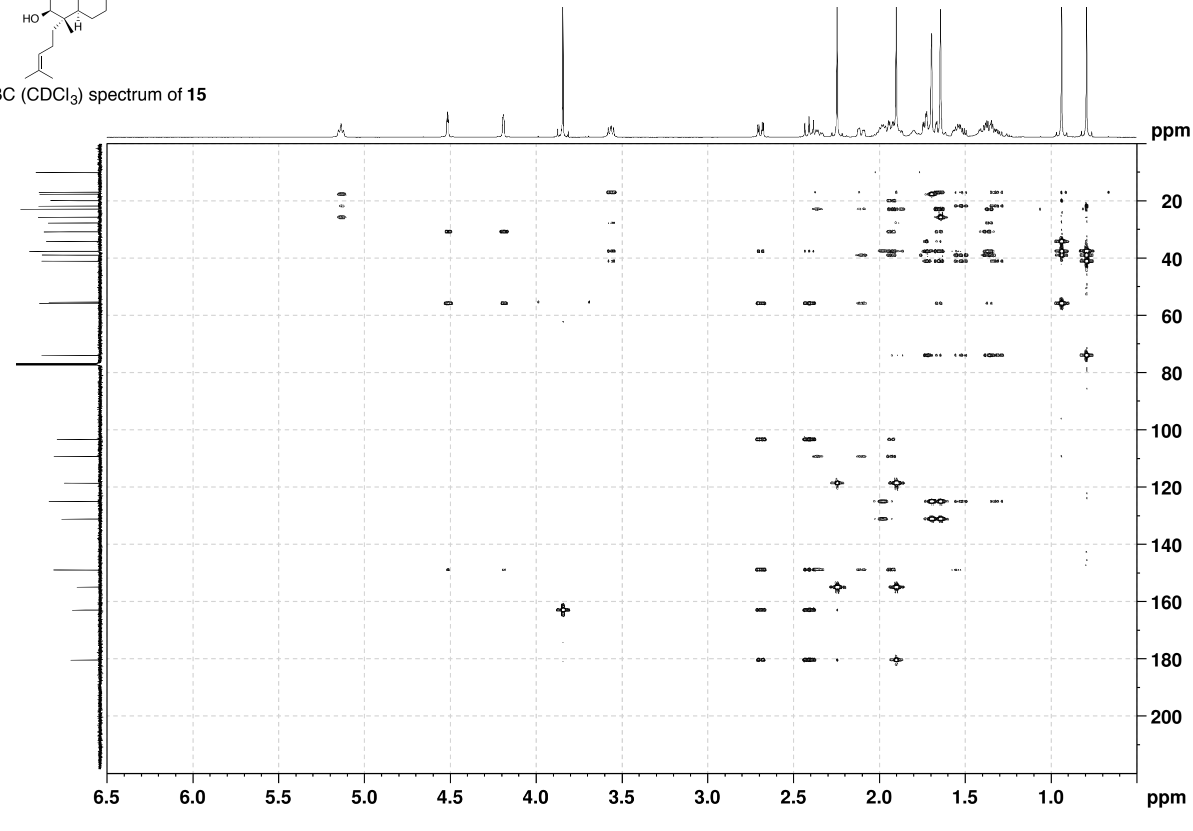

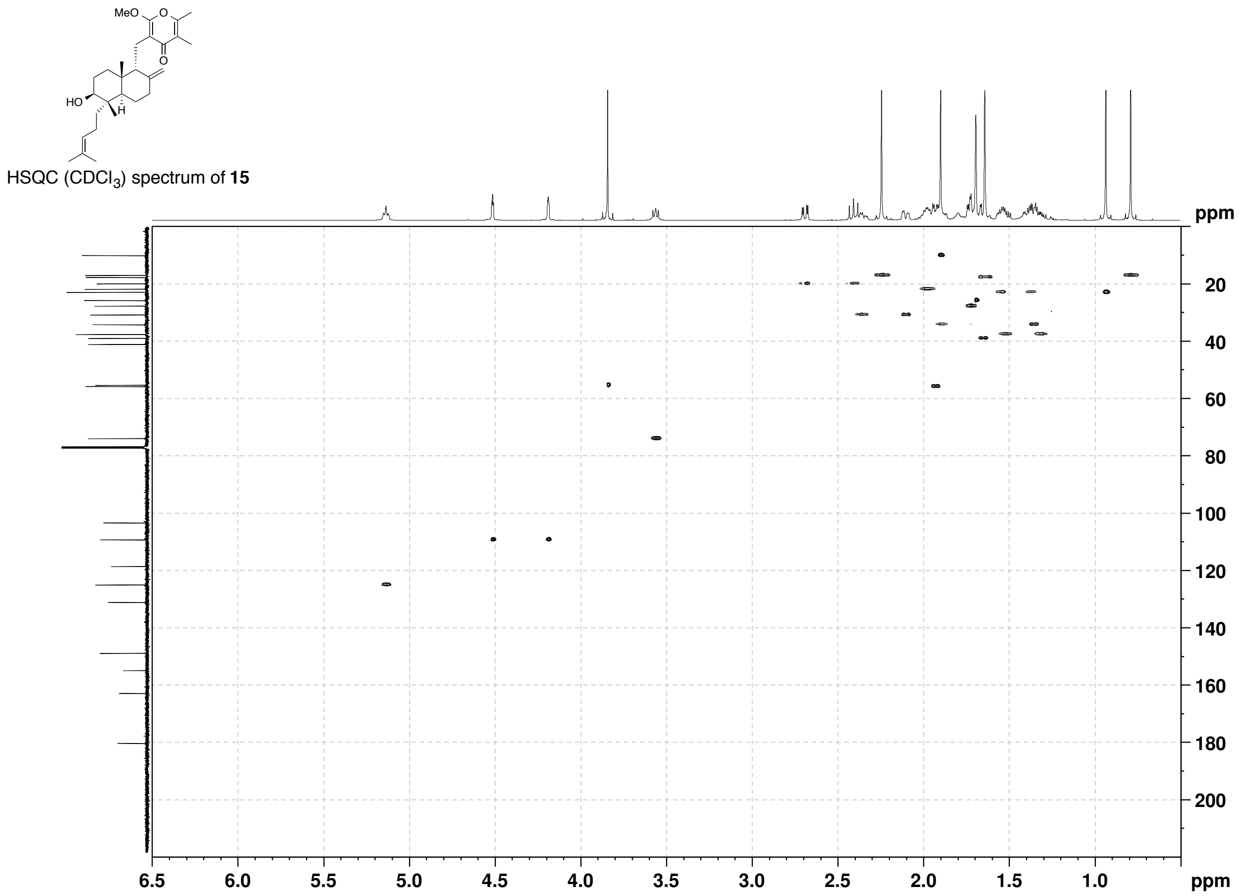

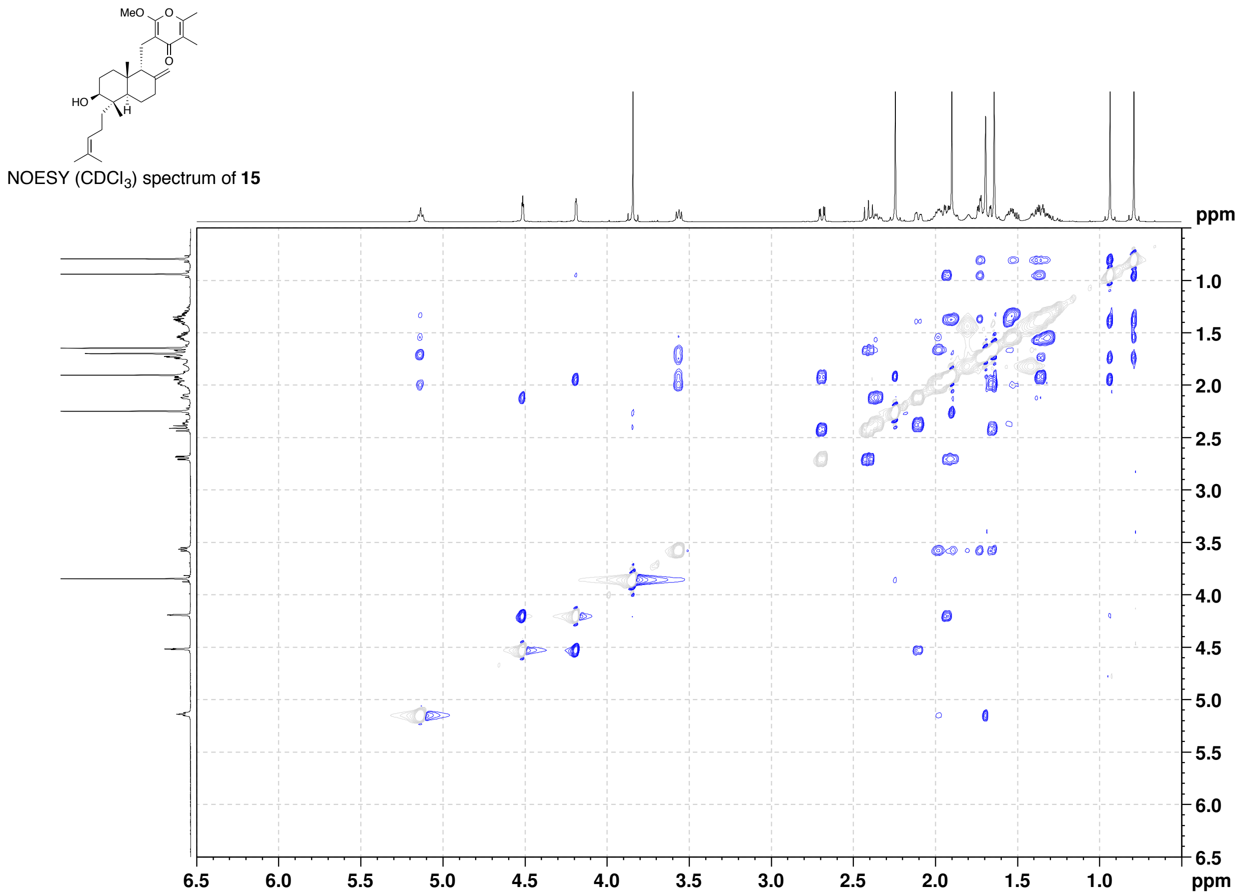

Supplementary Fig. 65. NMR spectra of 15 (CDCl$_3$)

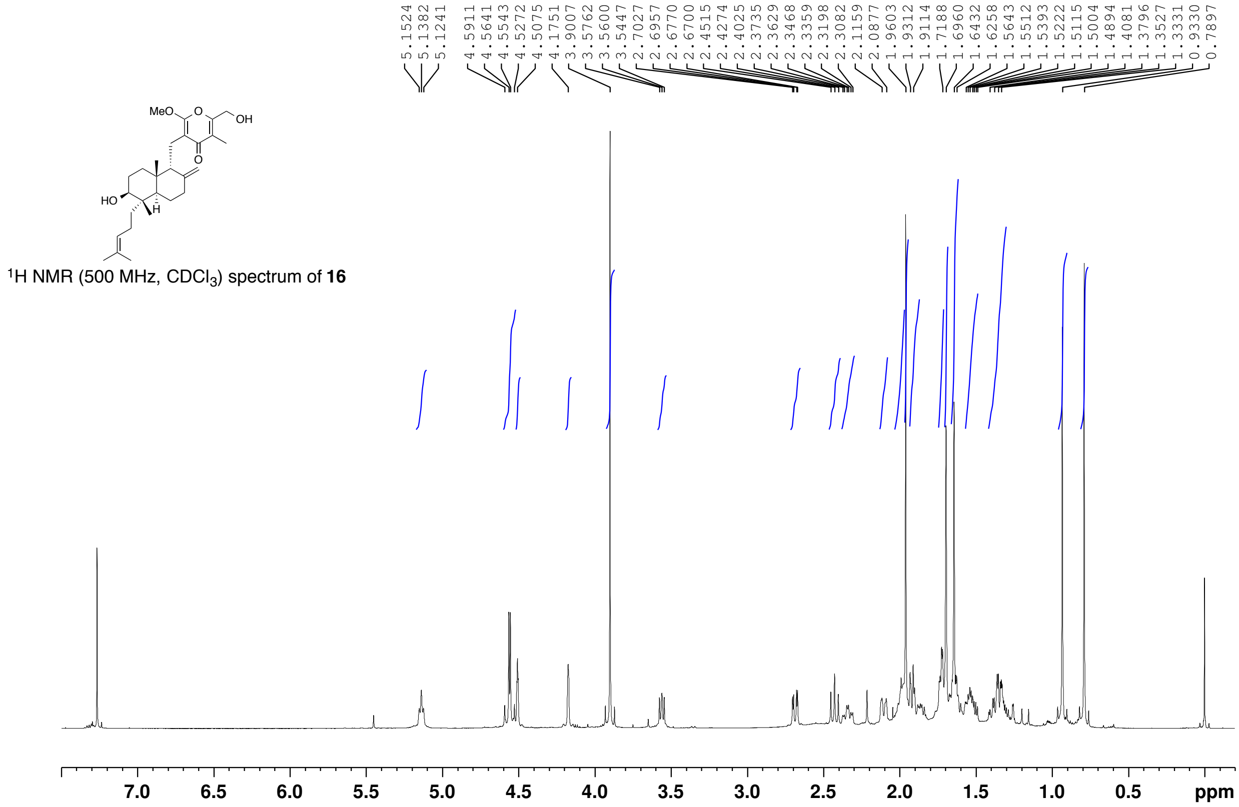

^{1}H NMR (500 MHz, $CDCl_3$) spectrum of **16**

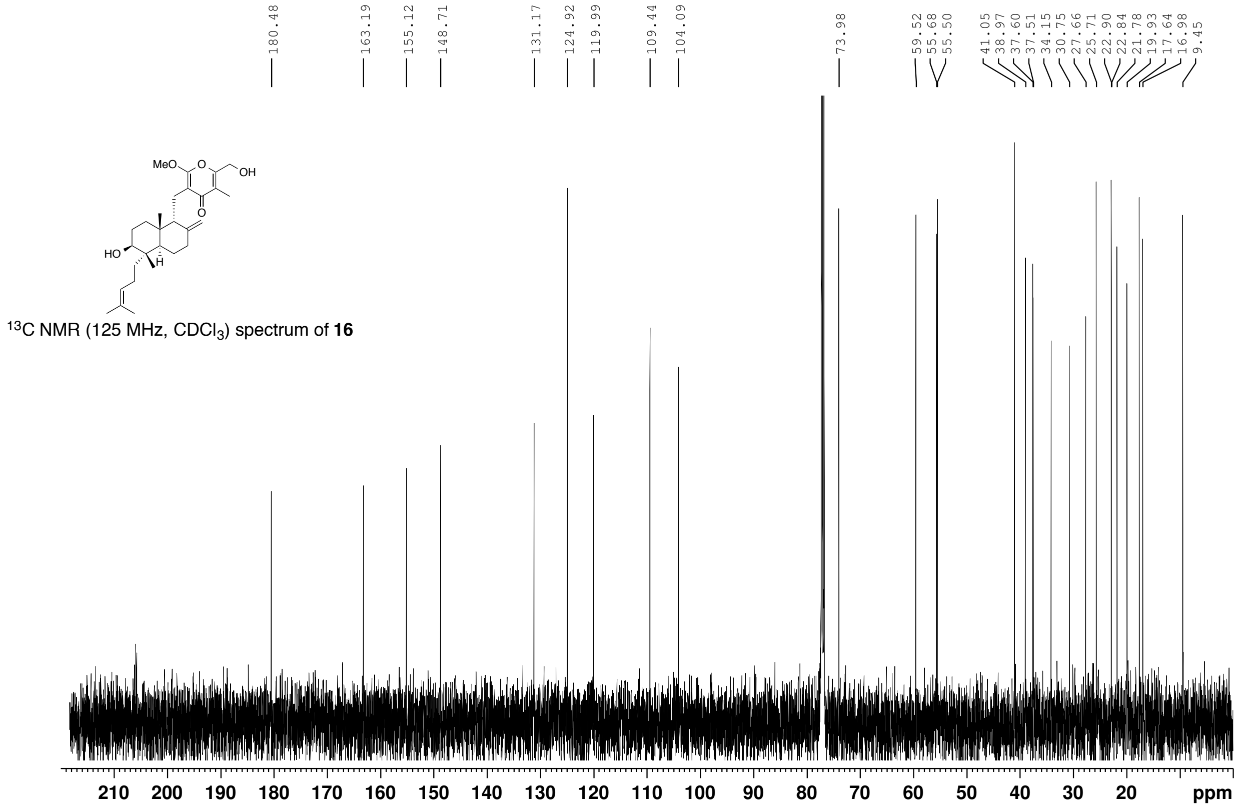

^{13}C NMR (125 MHz, $CDCl_3$) spectrum of **16**

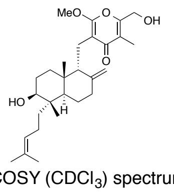

^{1}H-^{1}H COSY ($CDCl_3$) spectrum of **16**

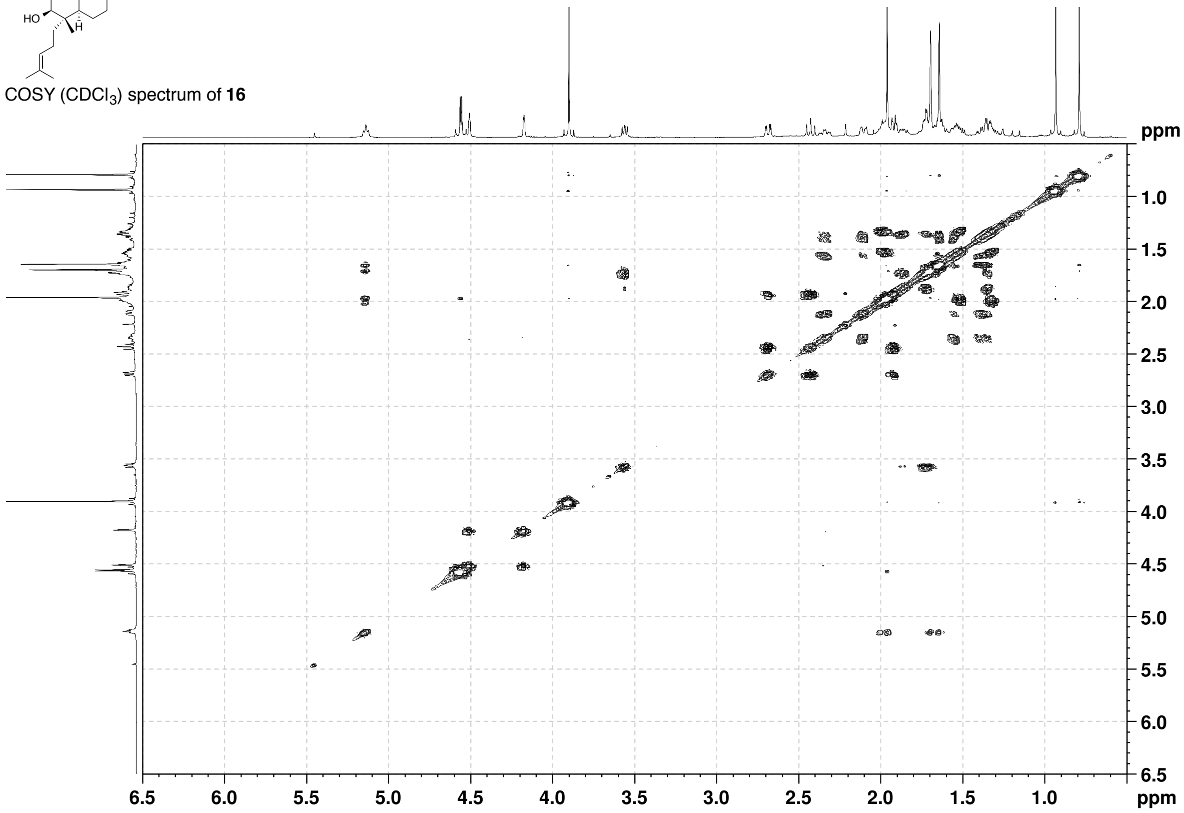

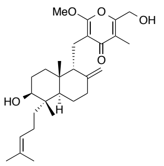

HMBC ($CDCl_3$) spectrum of **16**

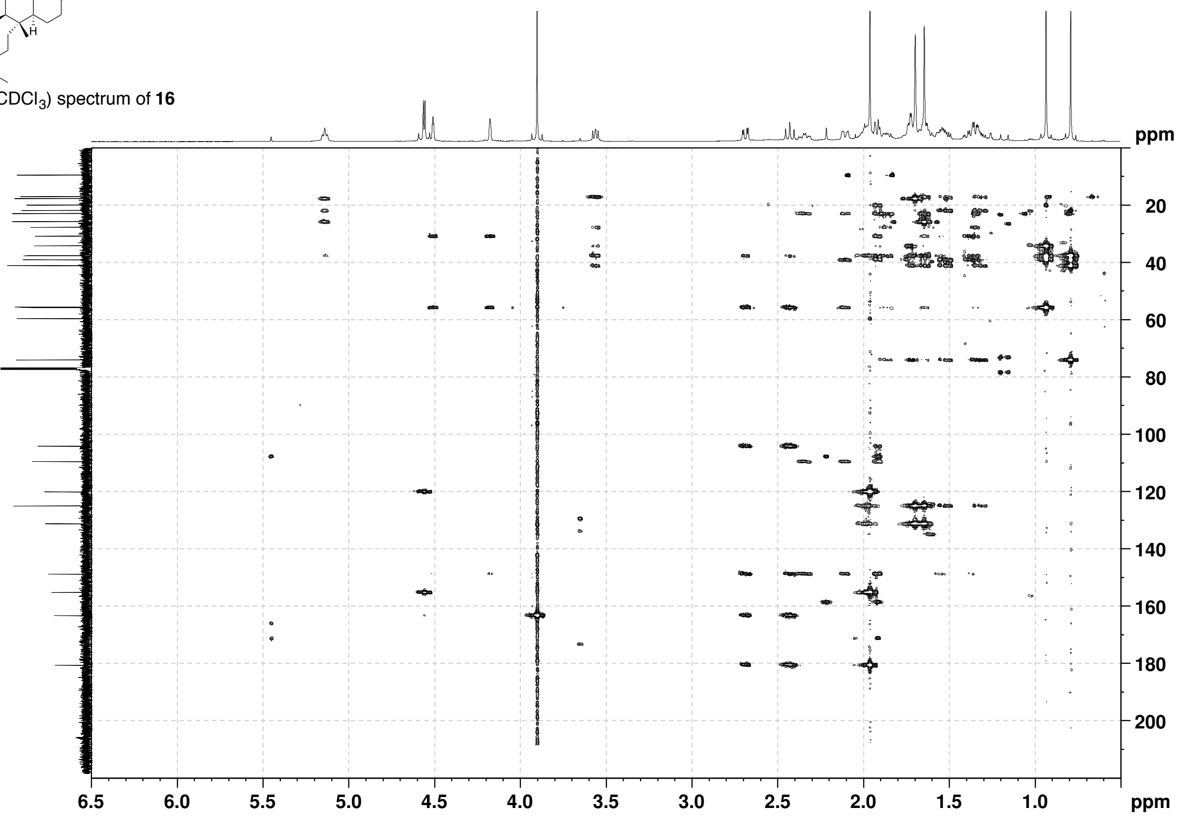

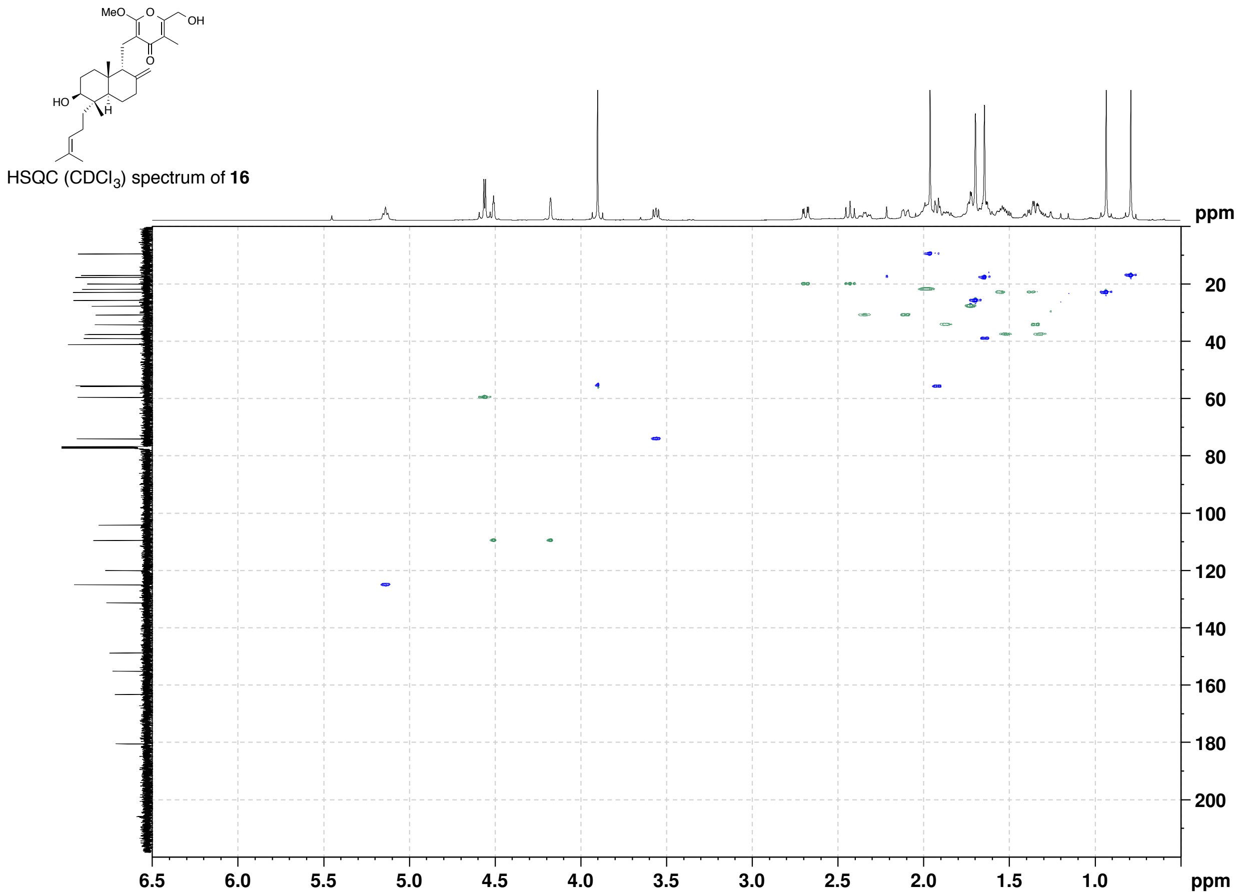

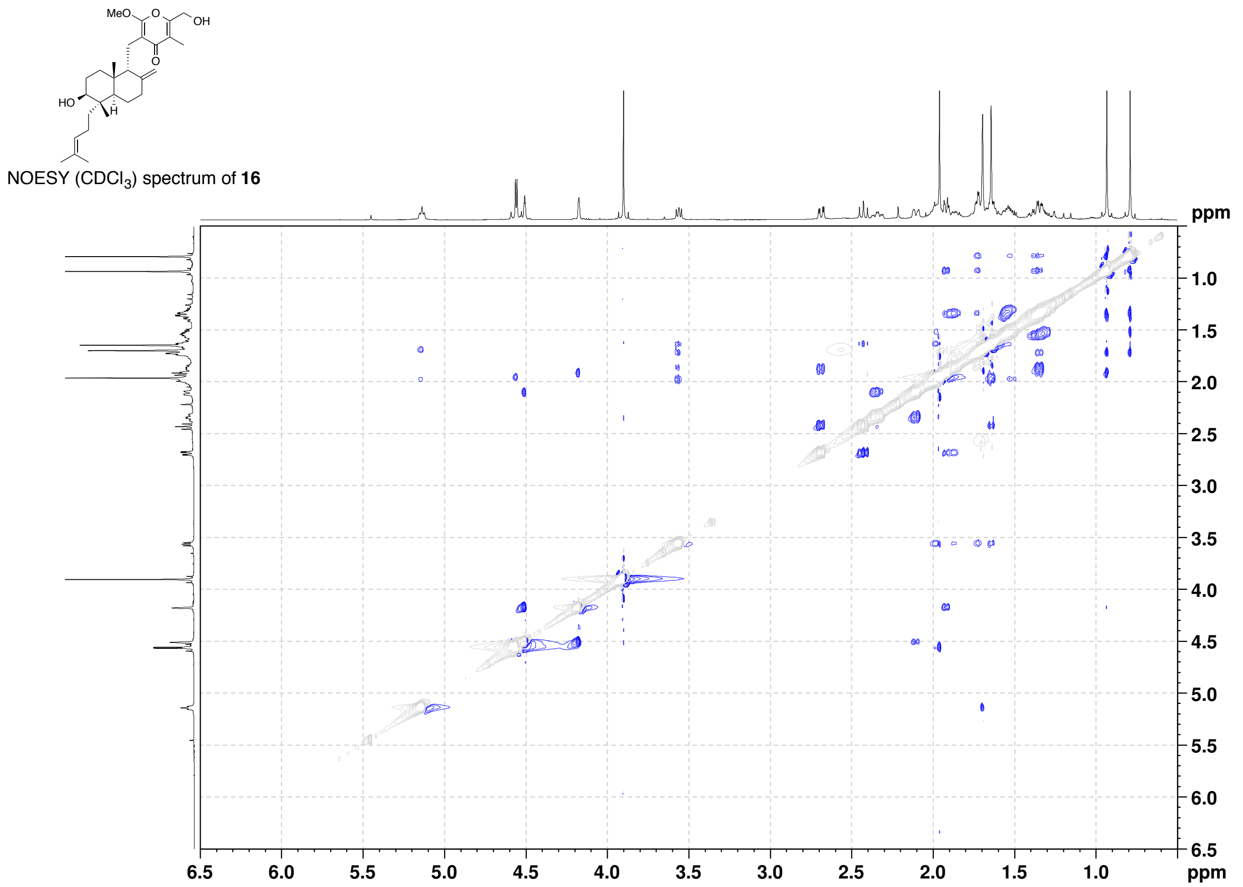

Supplementary Fig. 66. NMR spectra of 16 ($CDCl_3$)

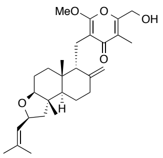

¹H NMR (500 MHz, $CDCl_3$) spectrum of **17**

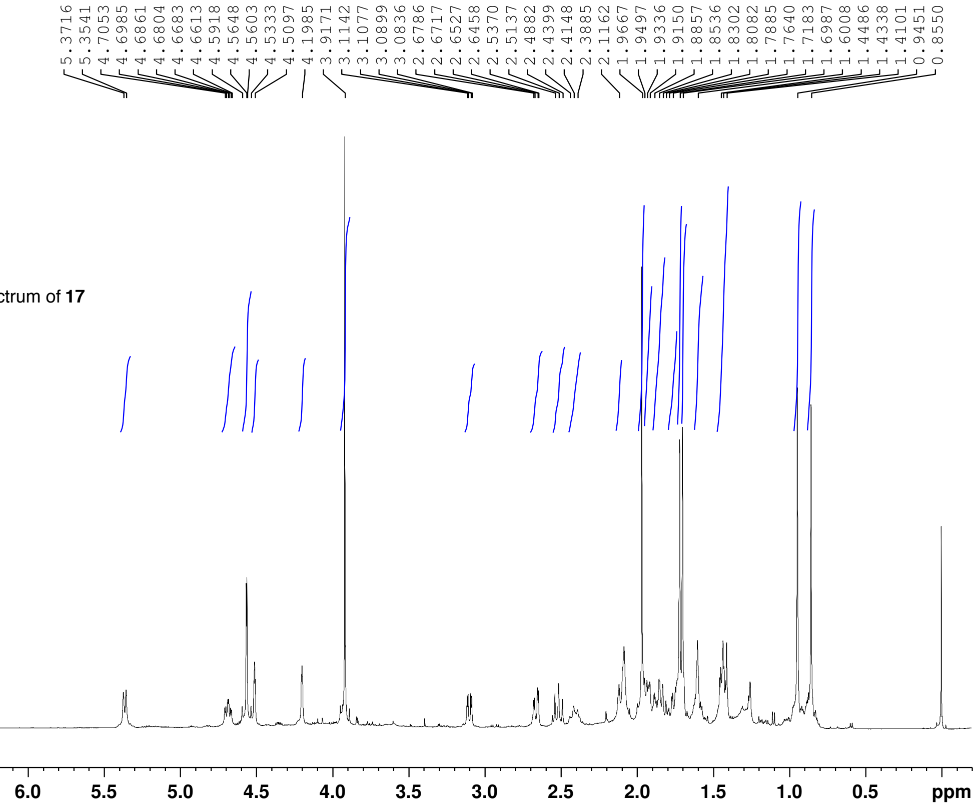

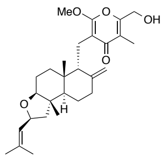

¹³C NMR (125 MHz, $CDCl_3$) spectrum of **17**

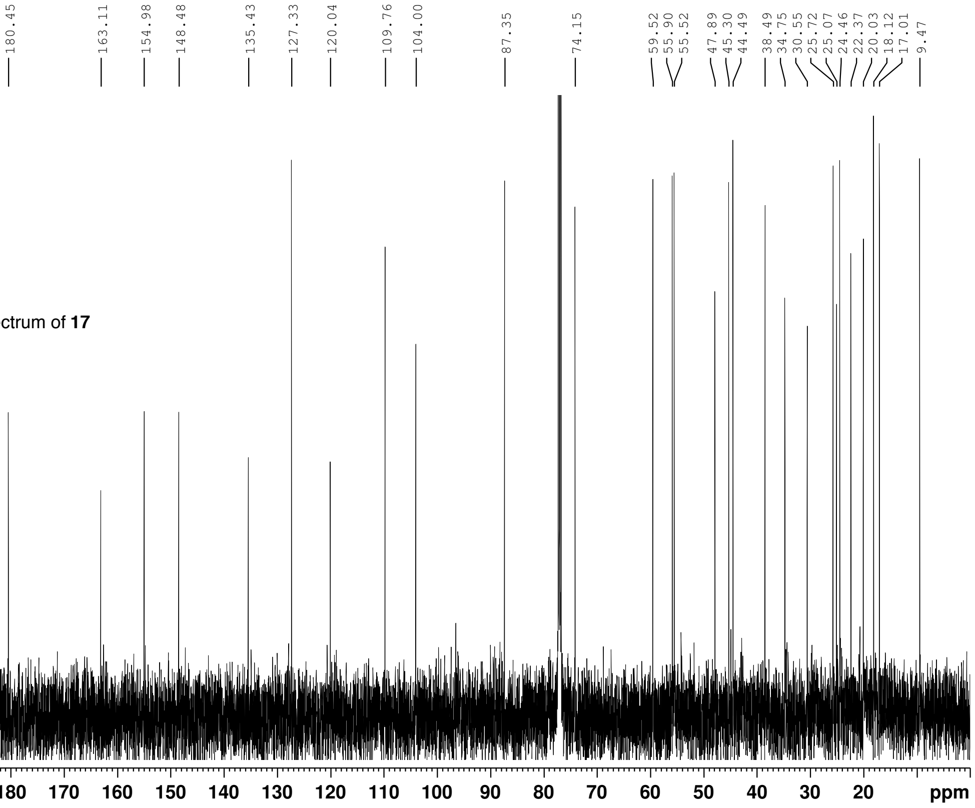

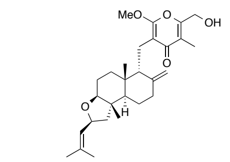

^{1}H-^{1}H COSY ($CDCl_3$) spectrum of **17**

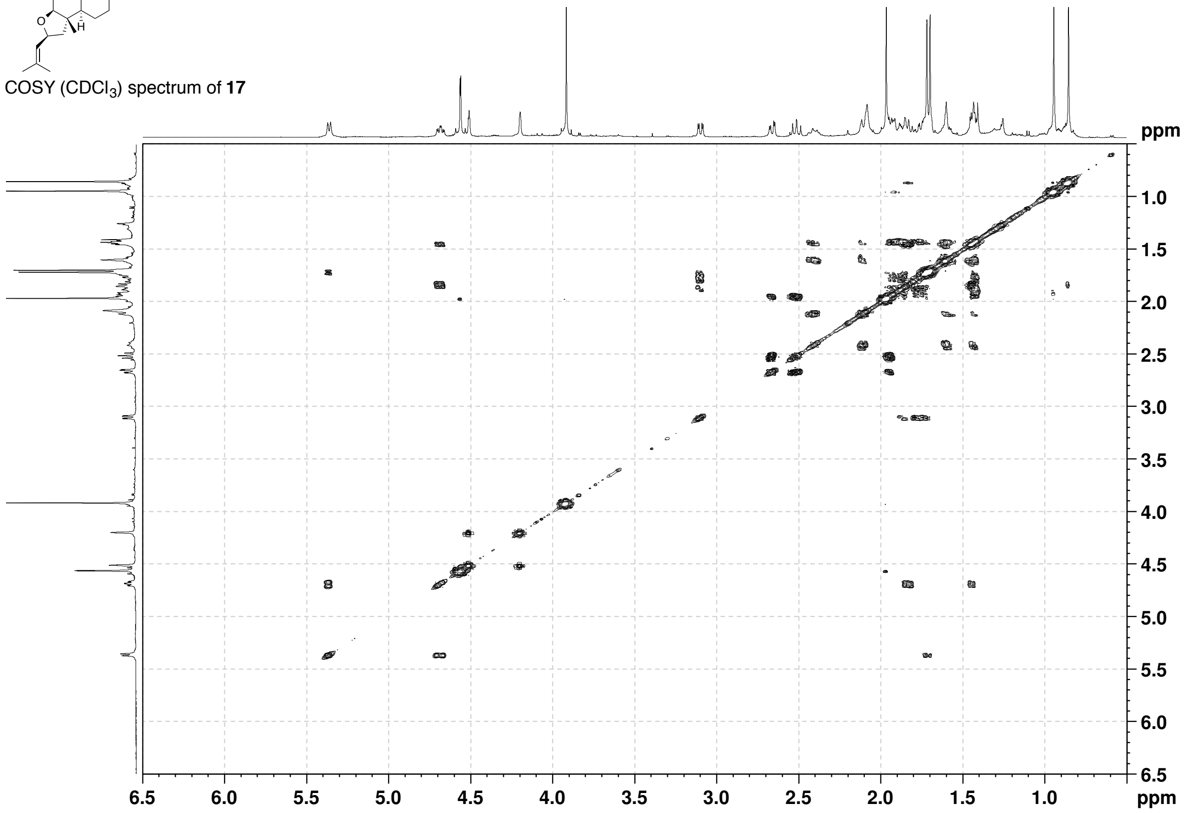

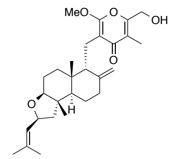

HMBC ($CDCl_3$) spectrum of **17**

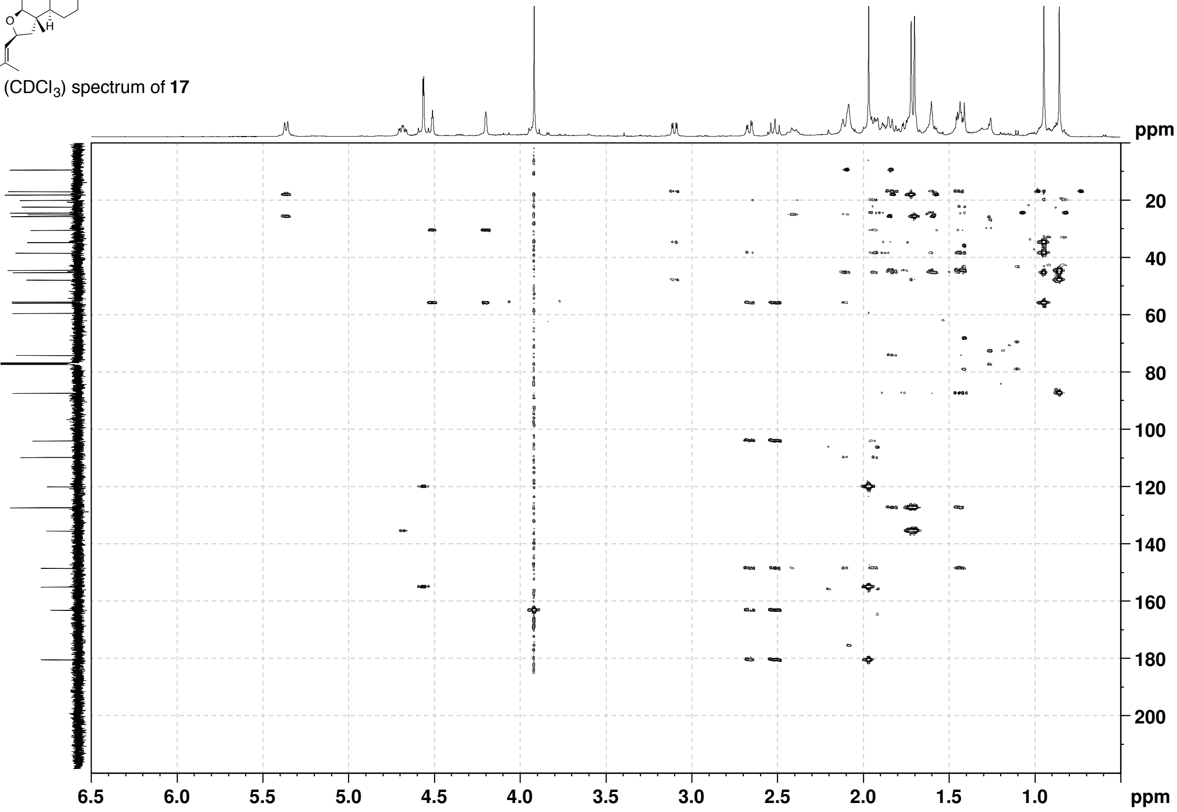

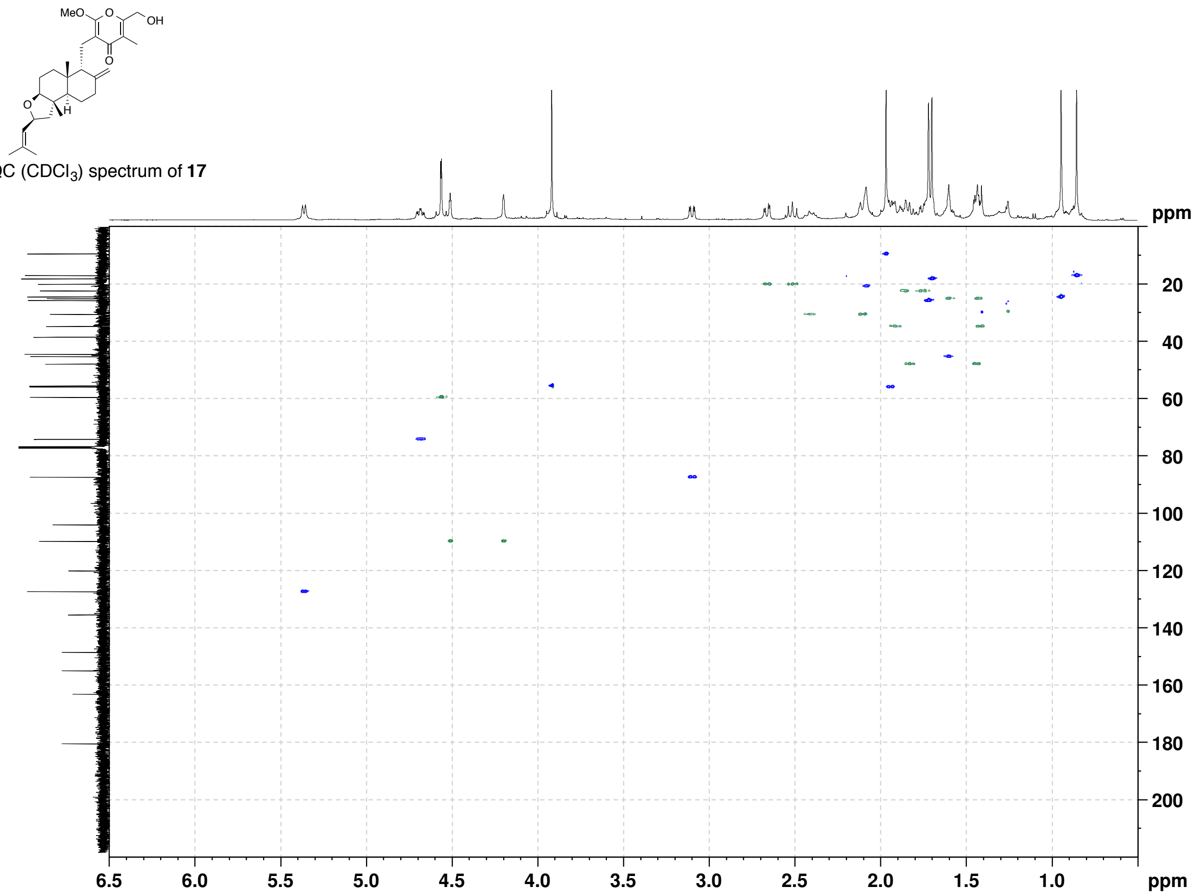

HSQC ($CDCl_3$) spectrum of **17**

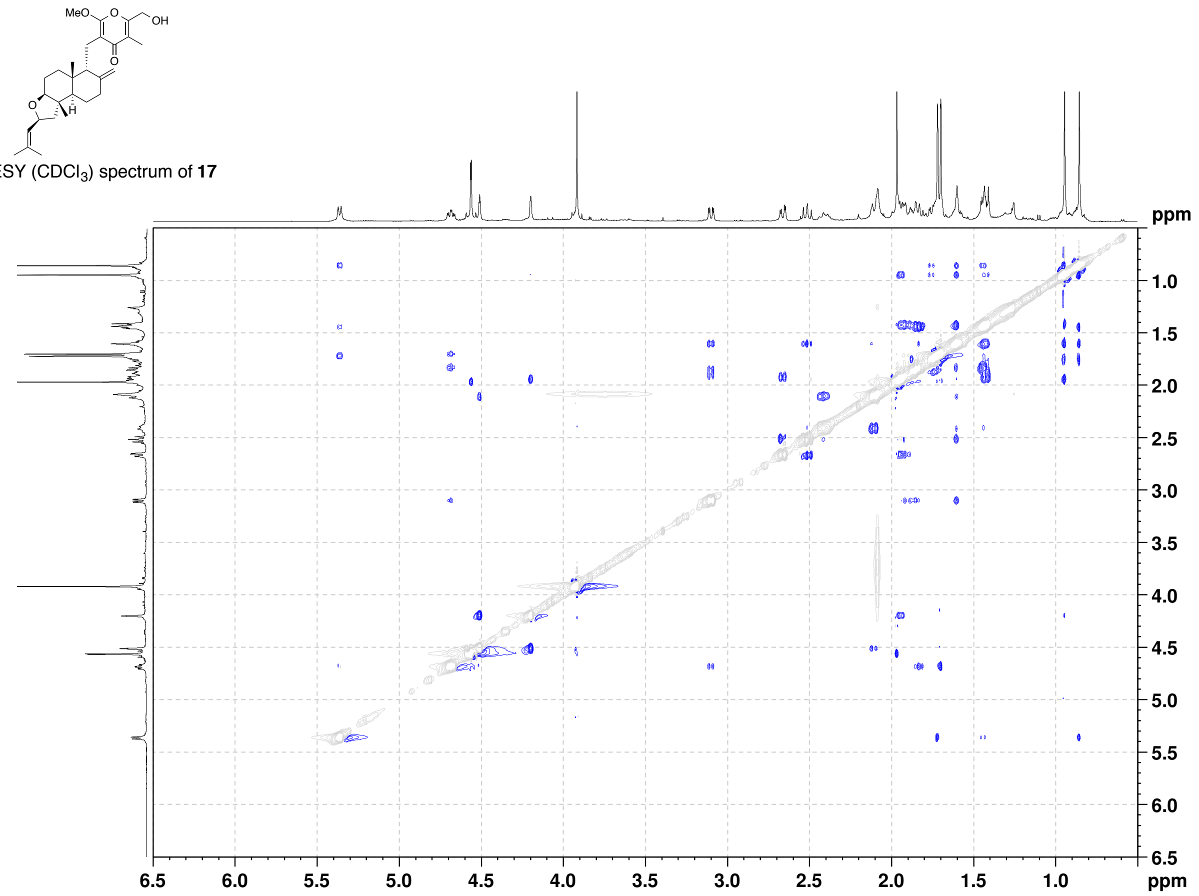

NOESY ($CDCl_3$) spectrum of **17**

Supplementary Fig. 67. NMR spectra of 17 ($CDCl_3$)

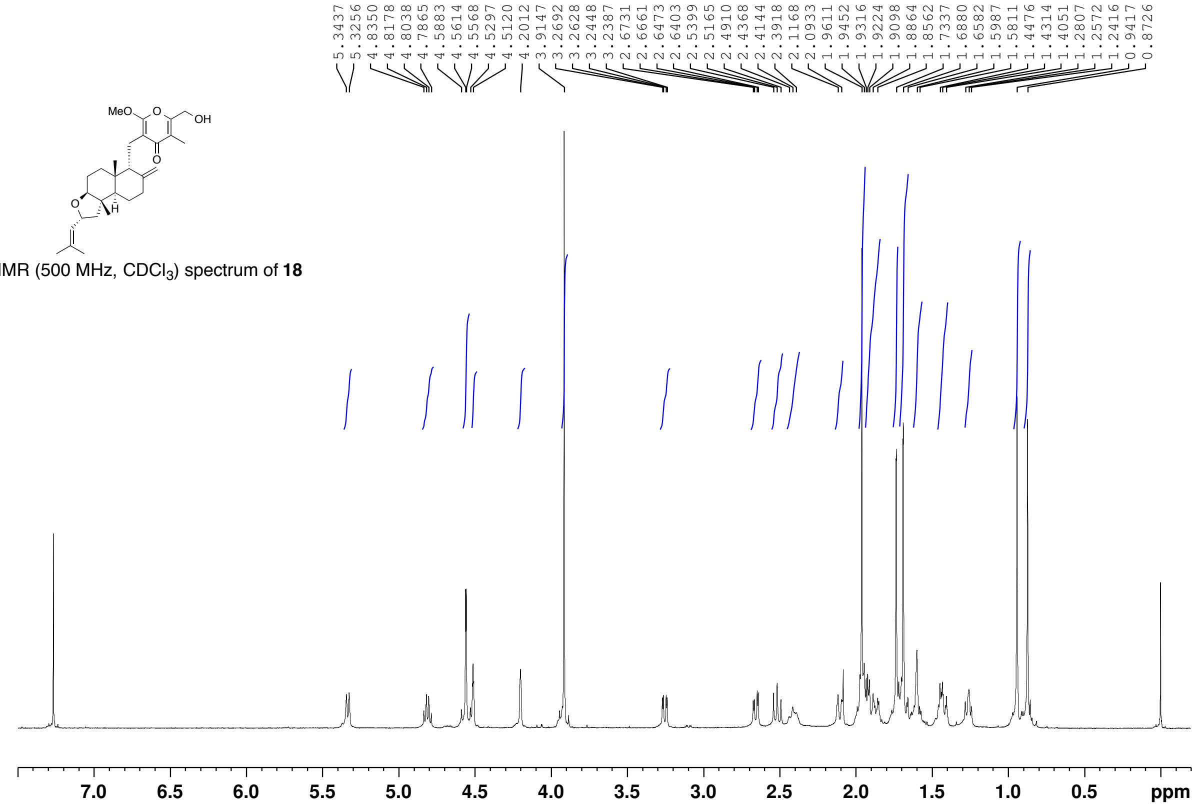

^{1}H NMR (500 MHz, $CDCl_3$) spectrum of **18**

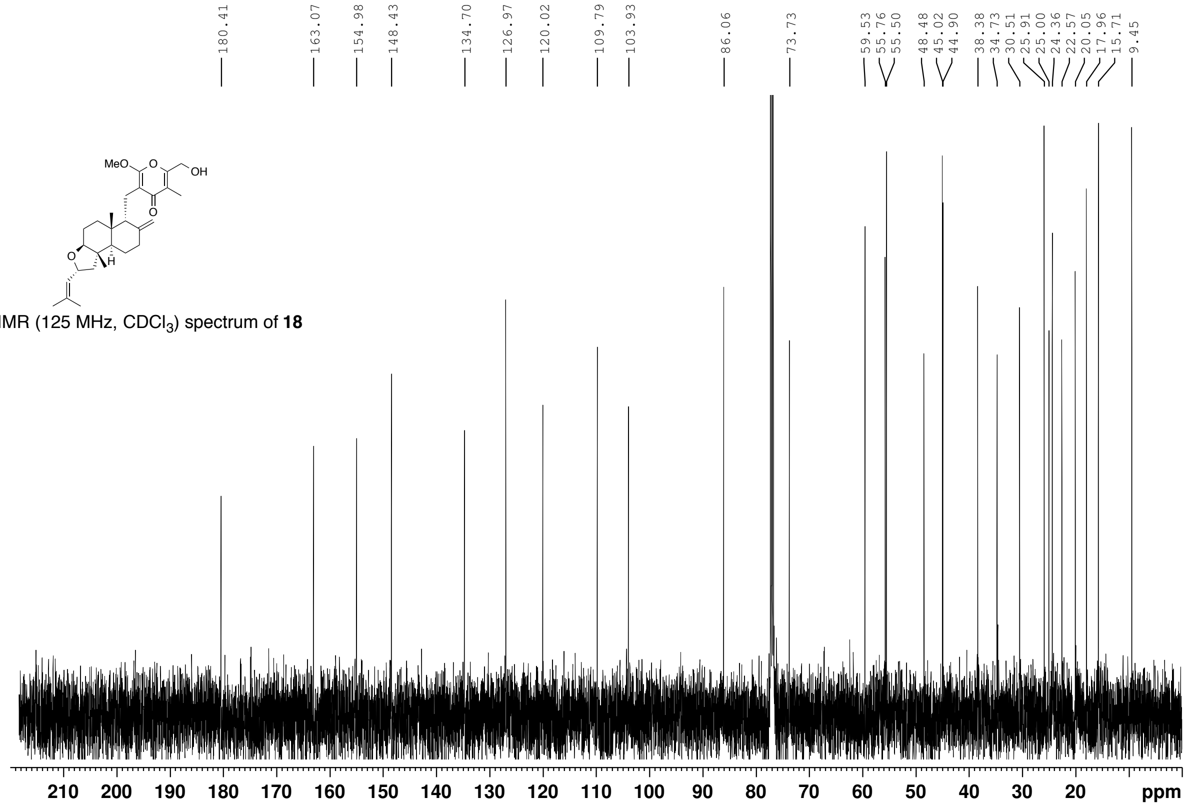

^{13}C NMR (125 MHz, $CDCl_3$) spectrum of **18**

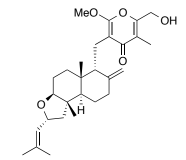

^{1}H-^{1}H COSY ($CDCl_3$) spectrum of **18**

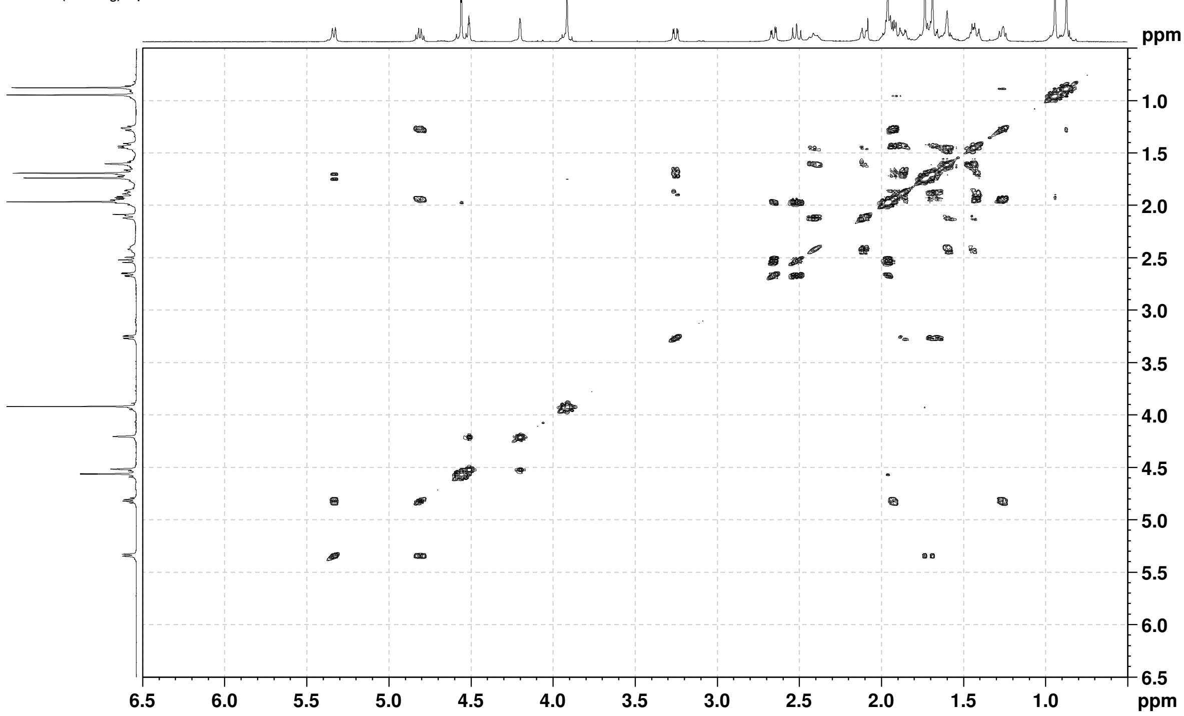

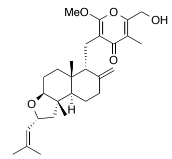

HMBC ($CDCl_3$) spectrum of **18**

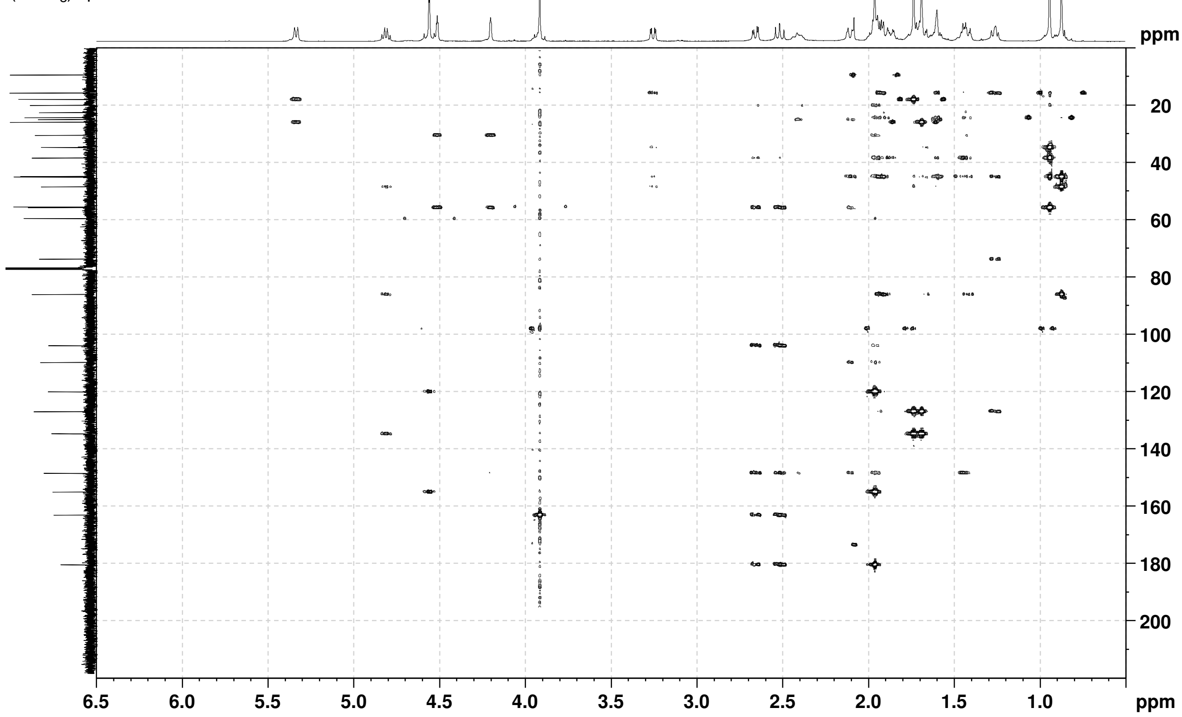

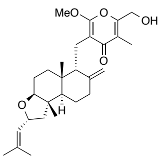

HSQC ($CDCl_3$) spectrum of **18**

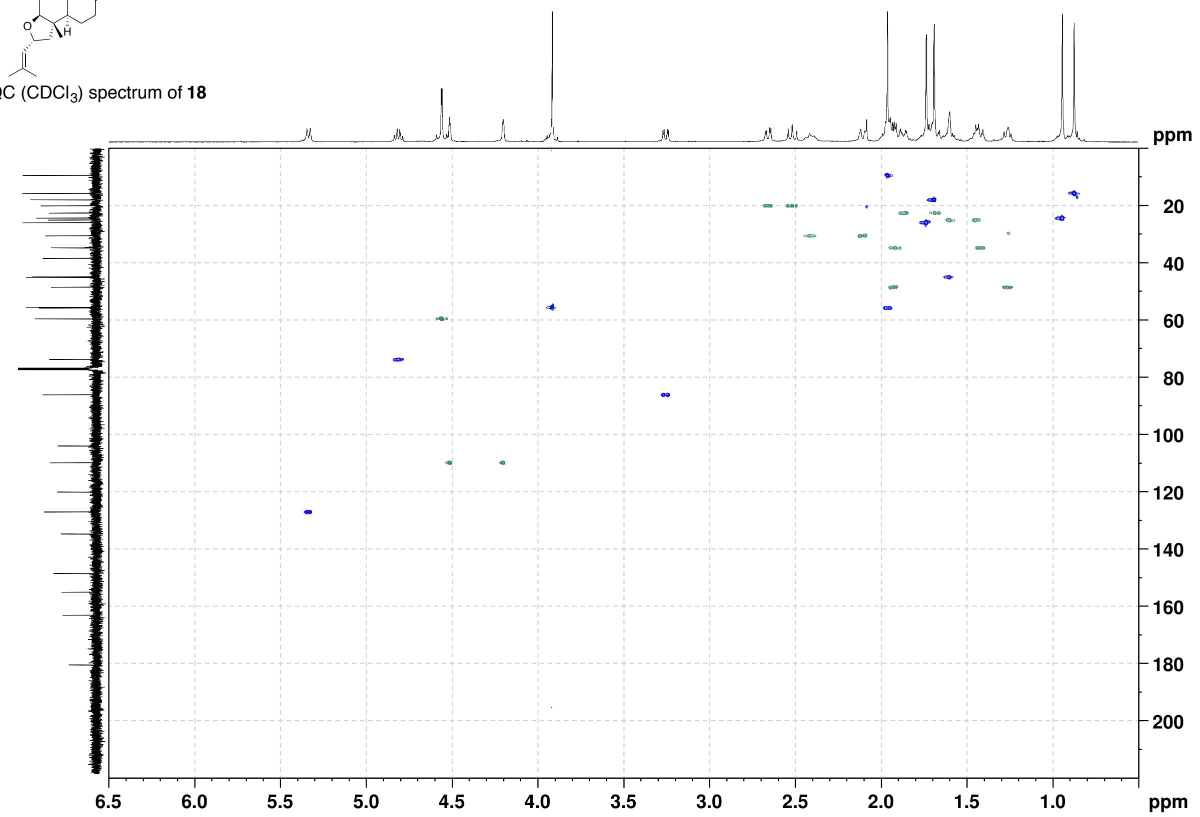

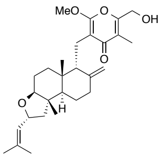

NOESY ($CDCl_3$) spectrum of **18**

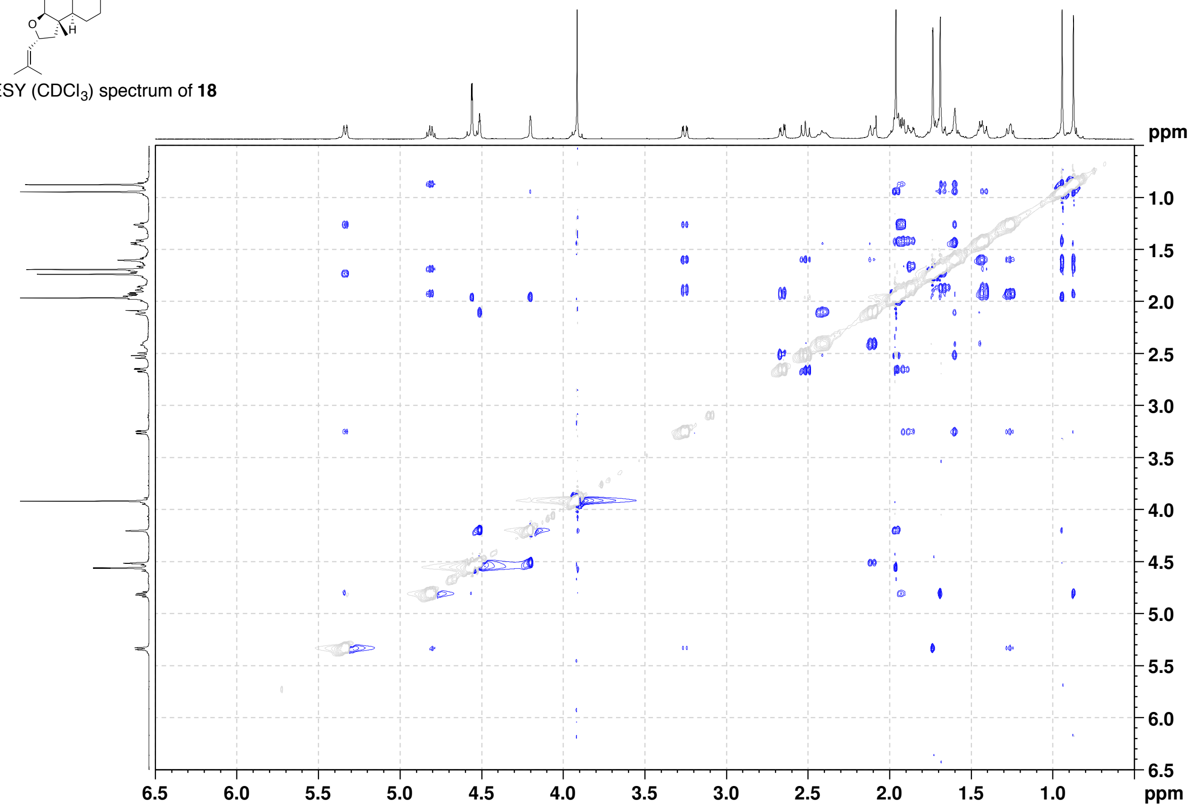

Supplementary Fig. 68. NMR spectra of 18 ($CDCl_3$)

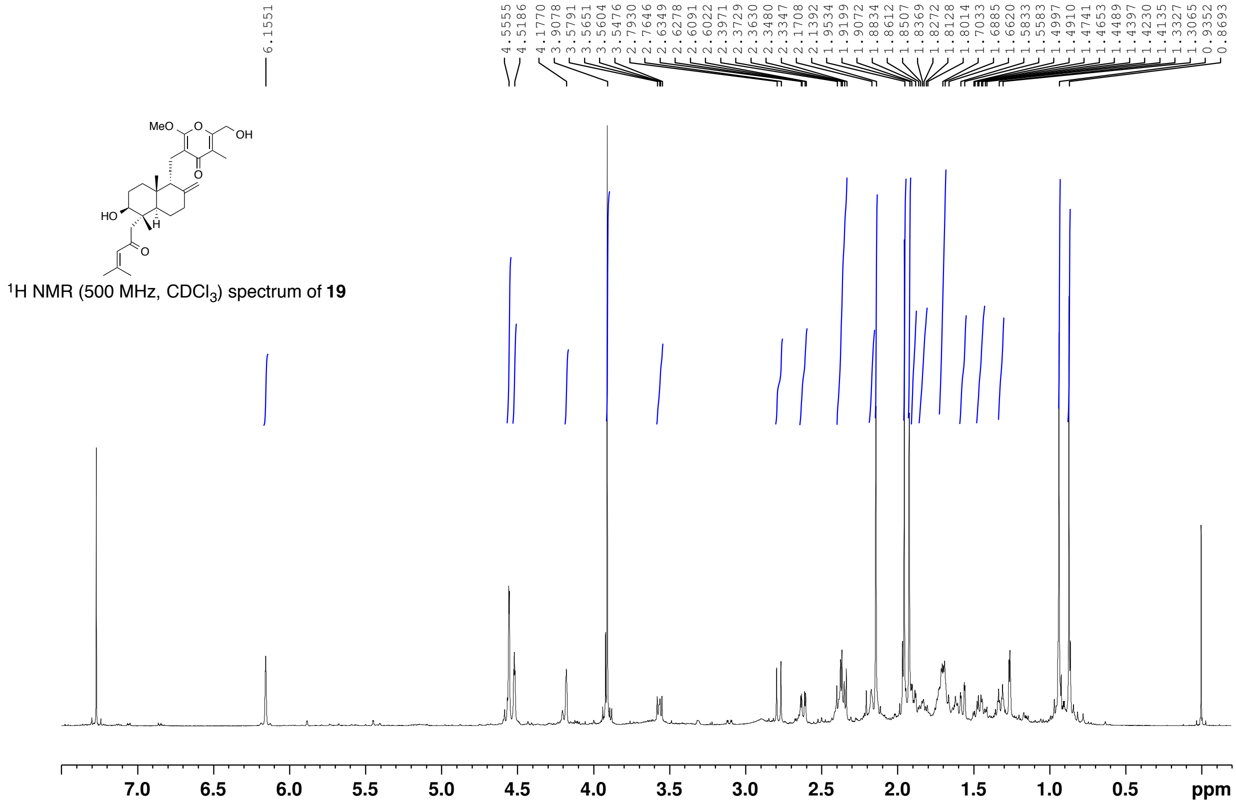

^{1}H NMR (500 MHz, $CDCl_3$) spectrum of **19**

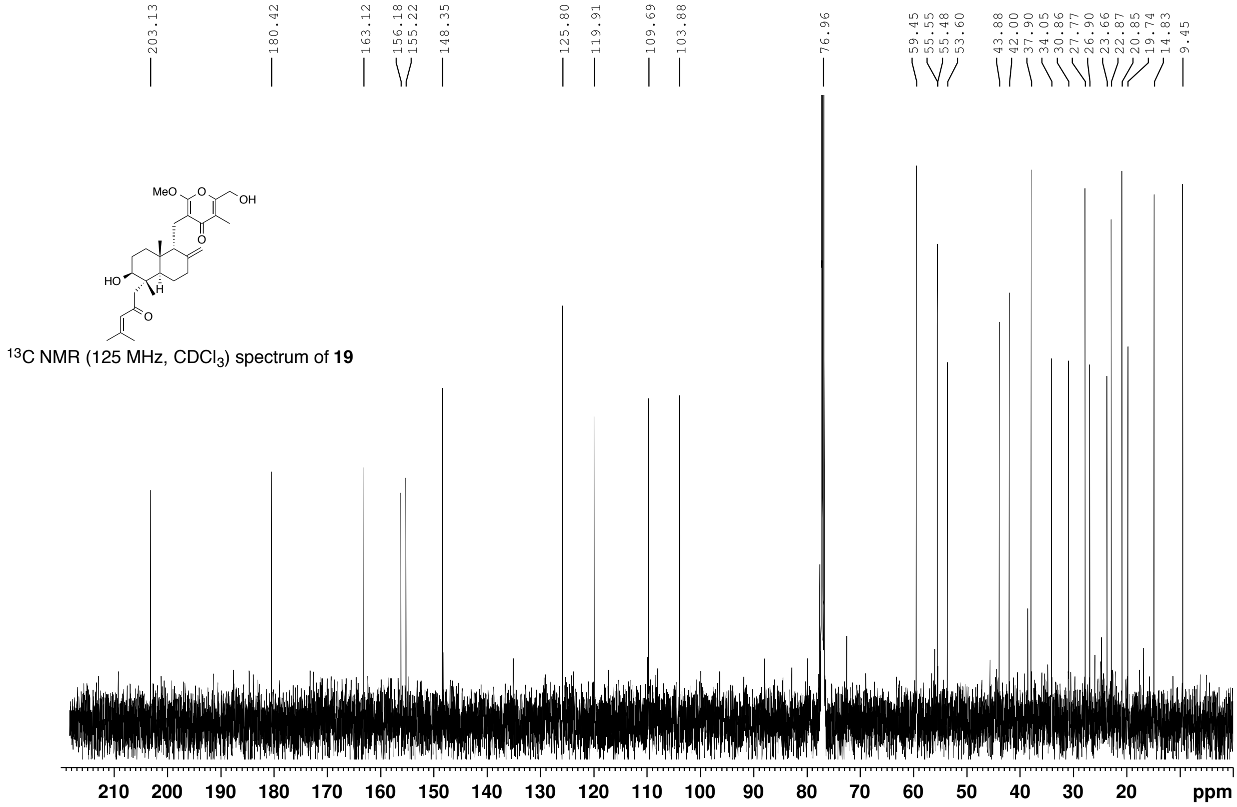

^{13}C NMR (125 MHz, $CDCl_3$) spectrum of **19**

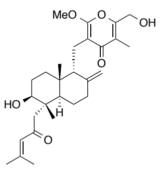

^{1}H-^{1}H COSY ($CDCl_3$) spectrum of **19**

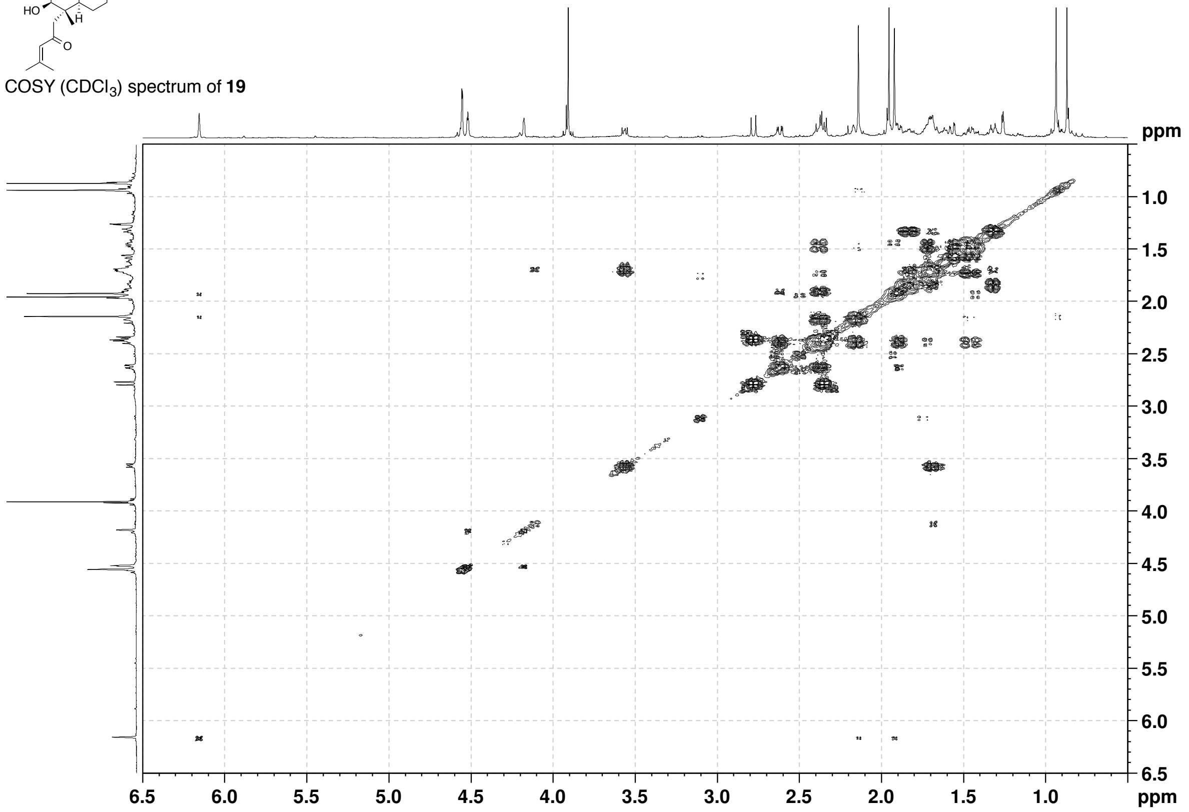

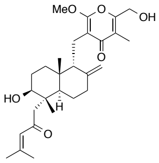

HMBC ($CDCl_3$) spectrum of **19**

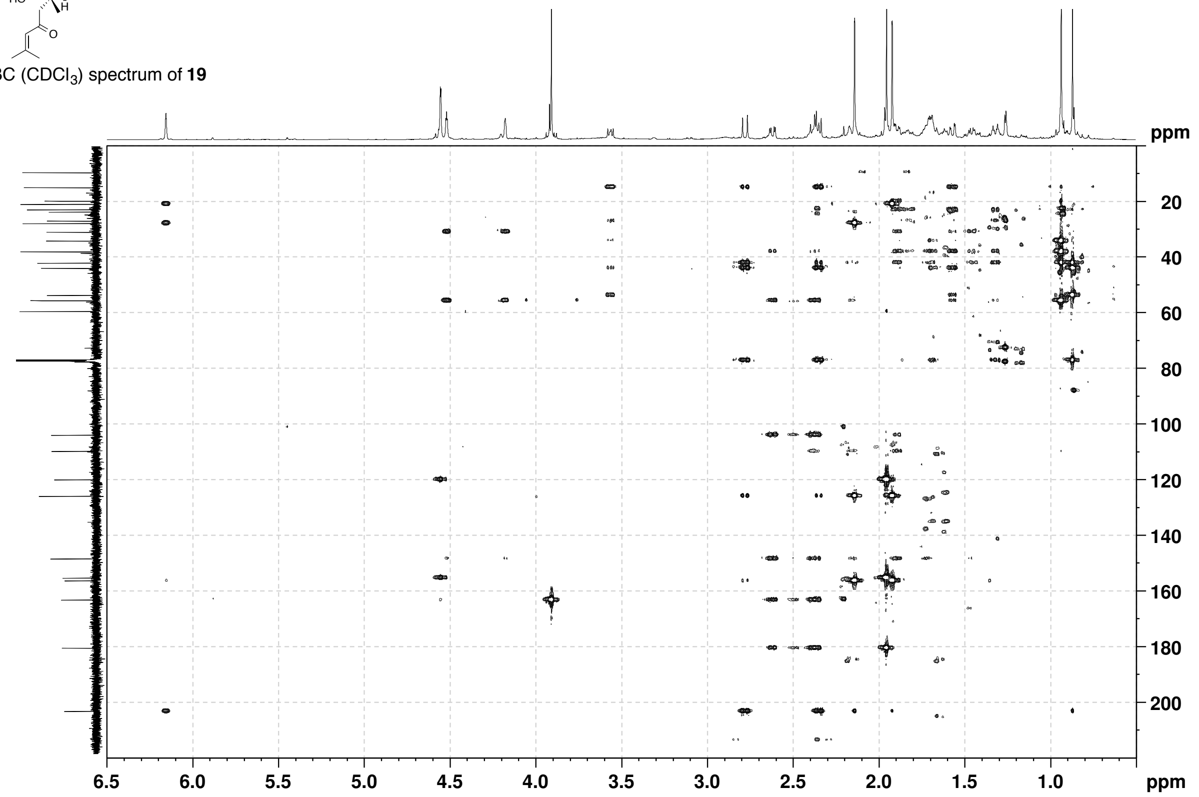

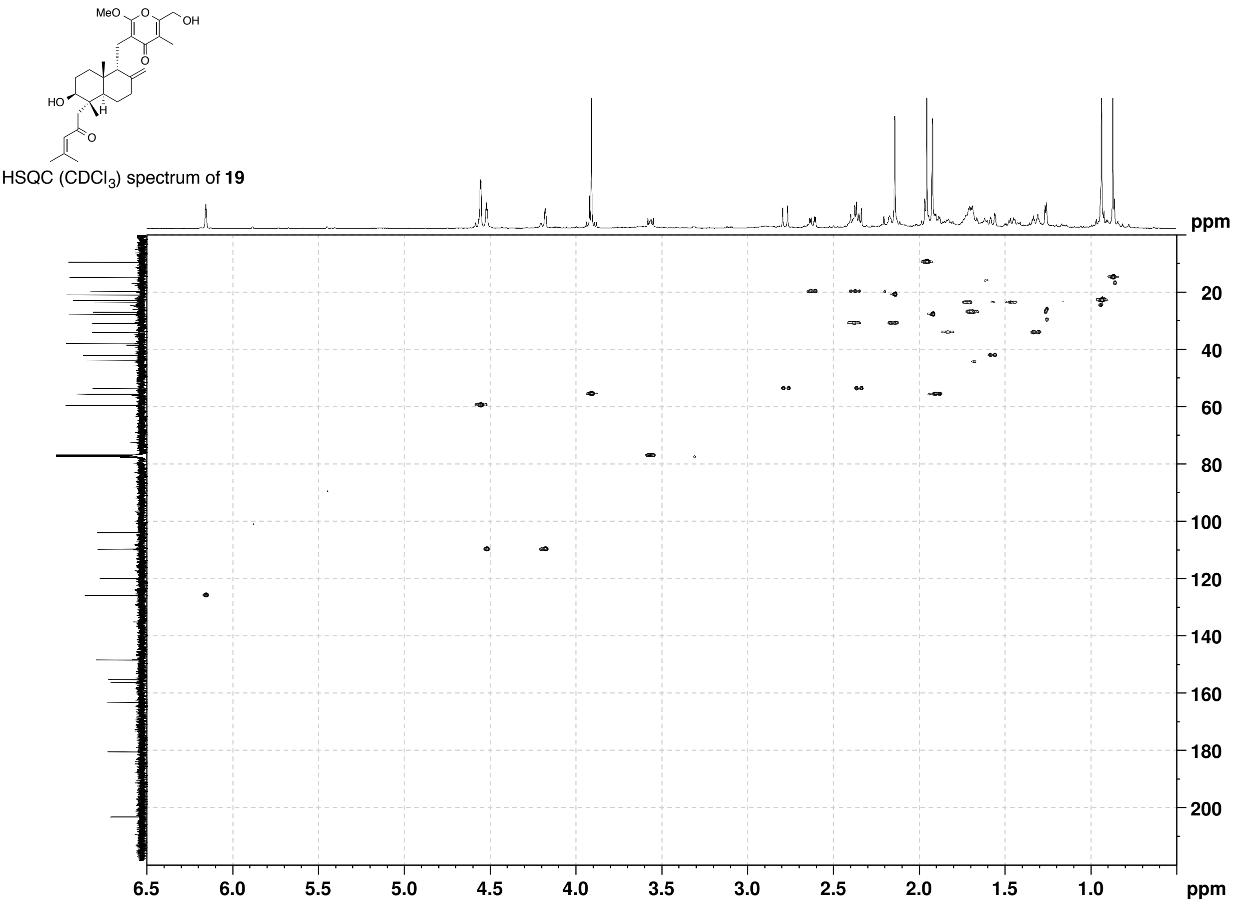

HSQC ($CDCl_3$) spectrum of **19**

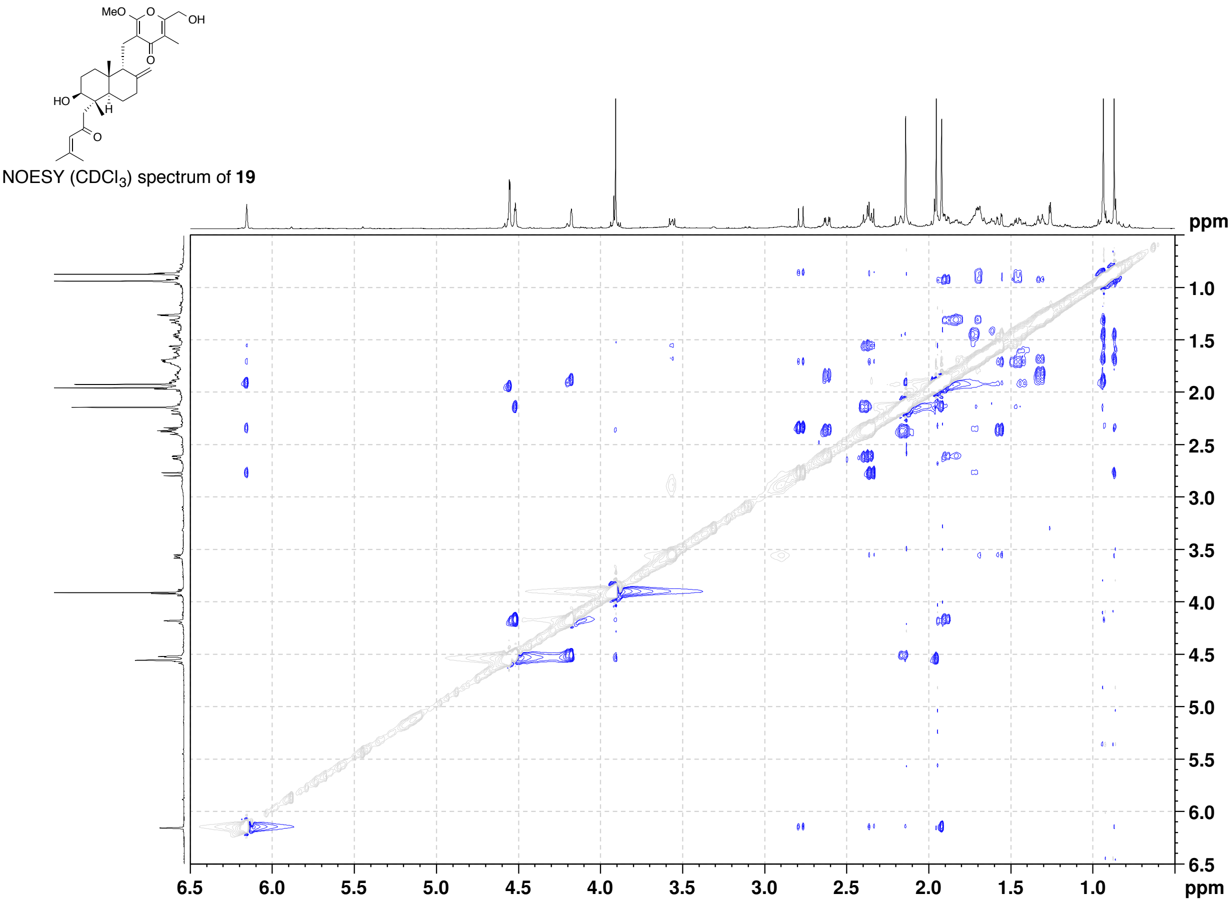

NOESY ($CDCl_3$) spectrum of **19**

Supplementary Fig. 69. NMR spectra of 19 ($CDCl_3$)

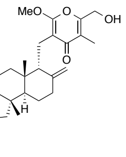

^{1}H NMR (500 MHz, $CDCl_3$) spectrum of **20**

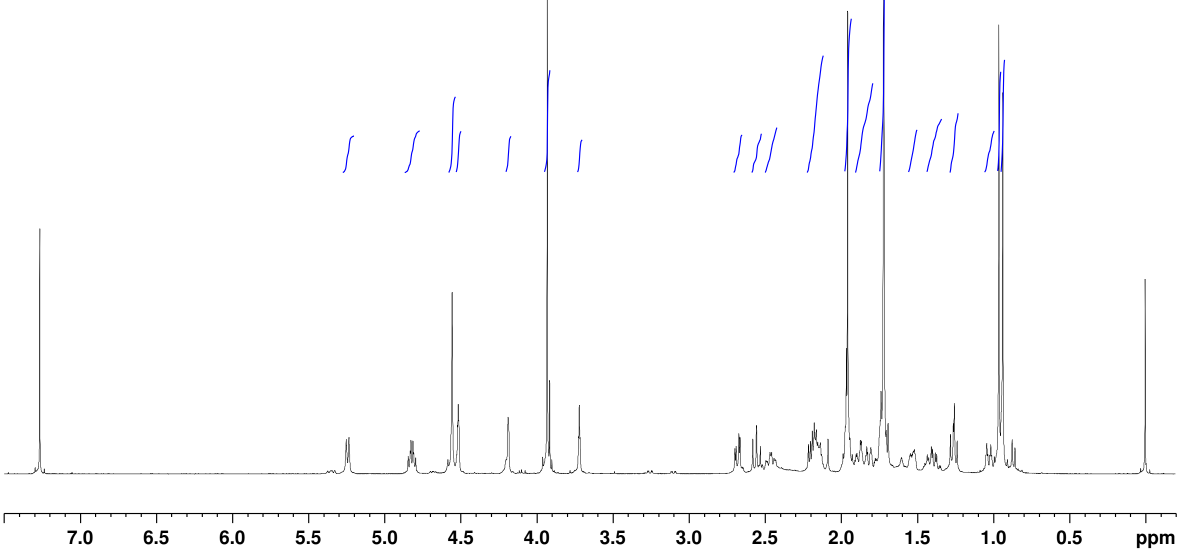

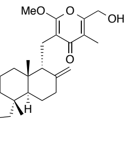

^{13}C NMR (125 MHz, $CDCl_3$) spectrum of **20**

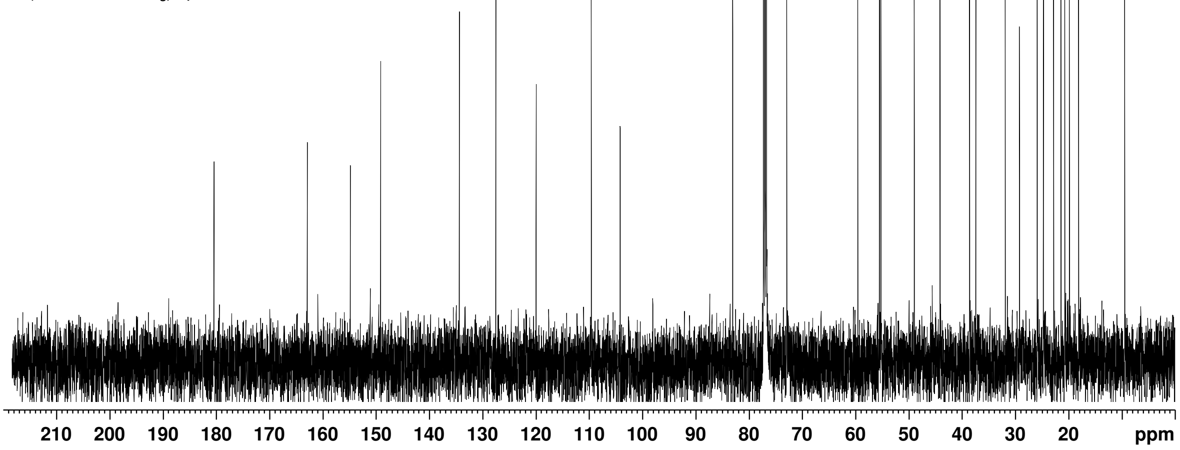

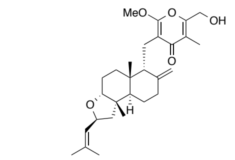

^{1}H-^{1}H COSY ($CDCl_3$) spectrum of **20**

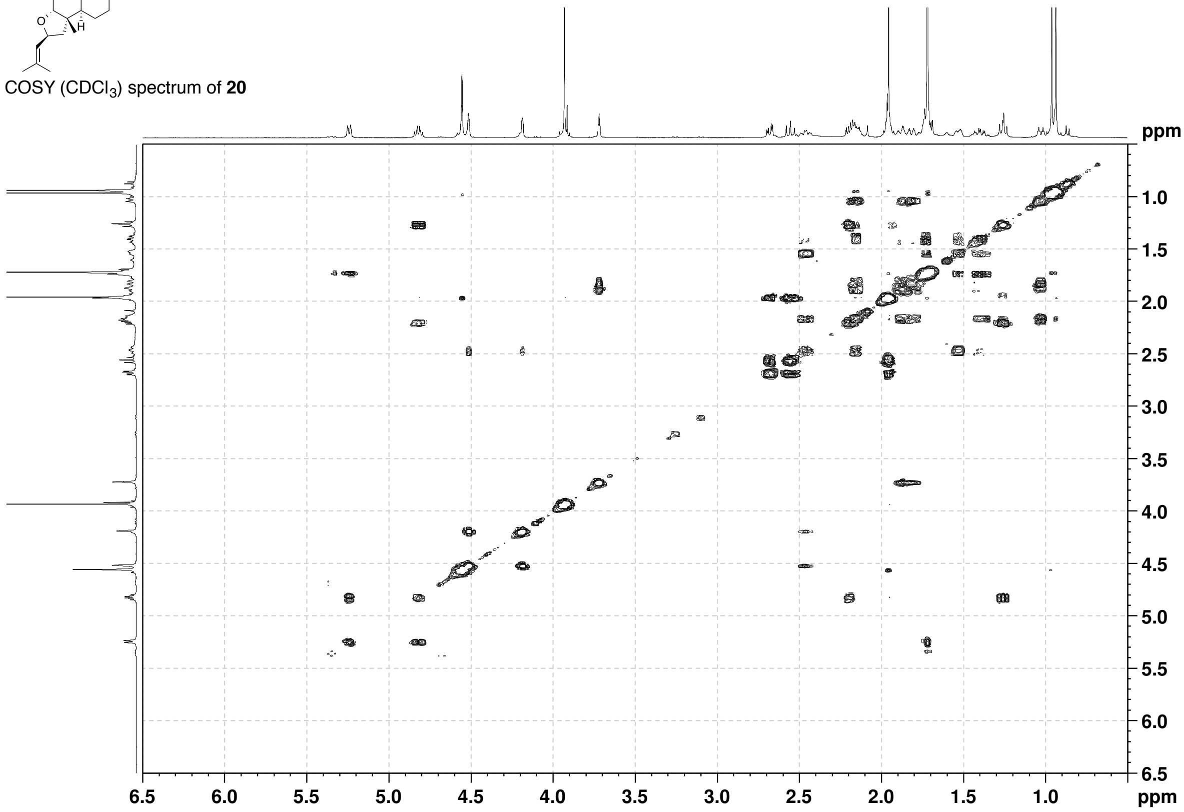

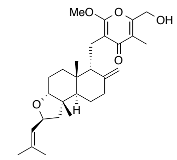

HMBC ($CDCl_3$) spectrum of **20**

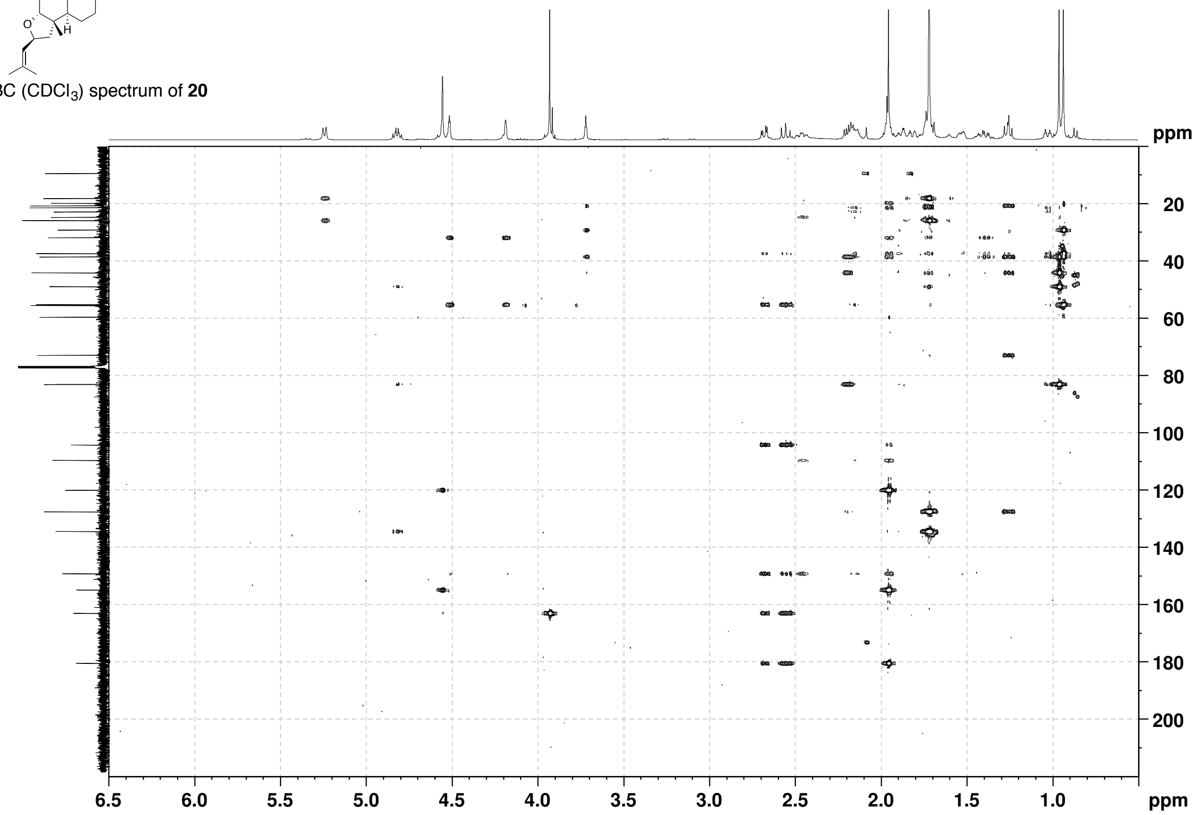

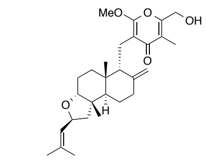

HSQC ($CDCl_3$) spectrum of **20**

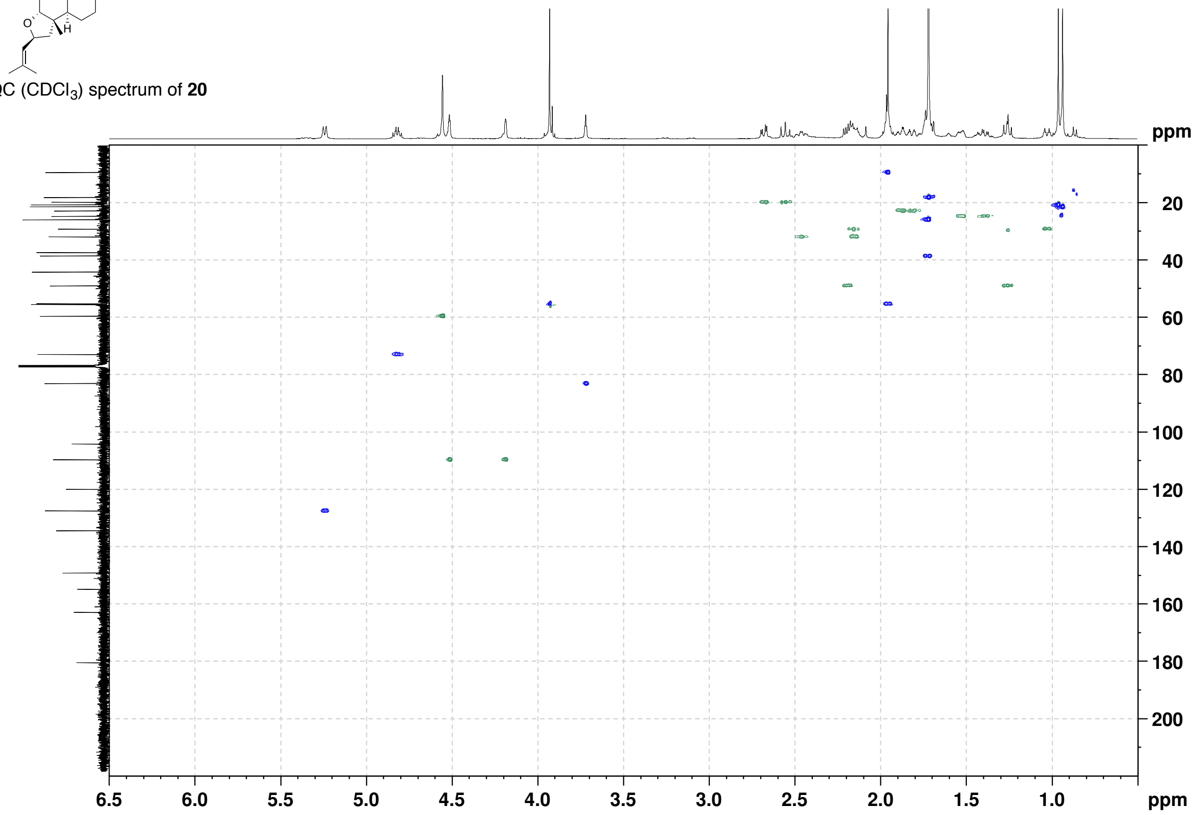

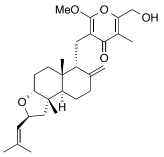

NOESY ($CDCl_3$) spectrum of **20**

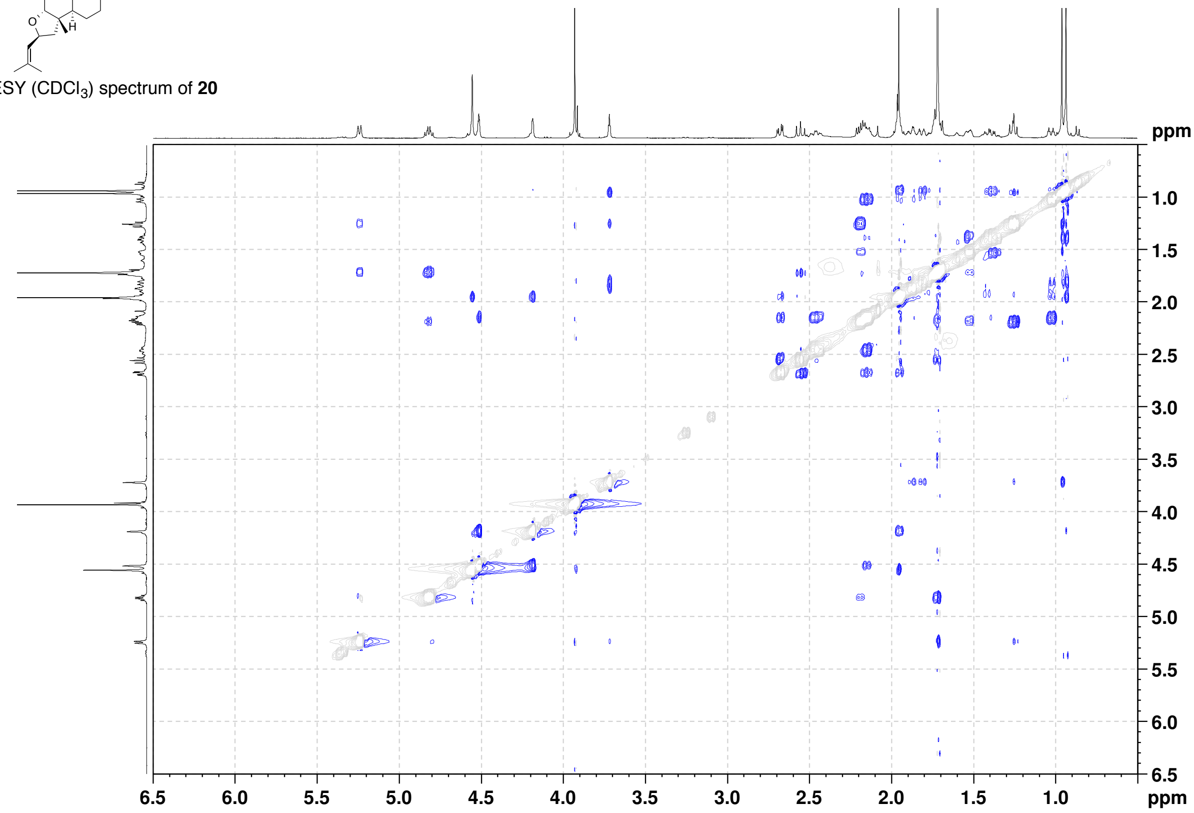

Supplementary Fig. 70. NMR spectra of 20 ($CDCl_3$)

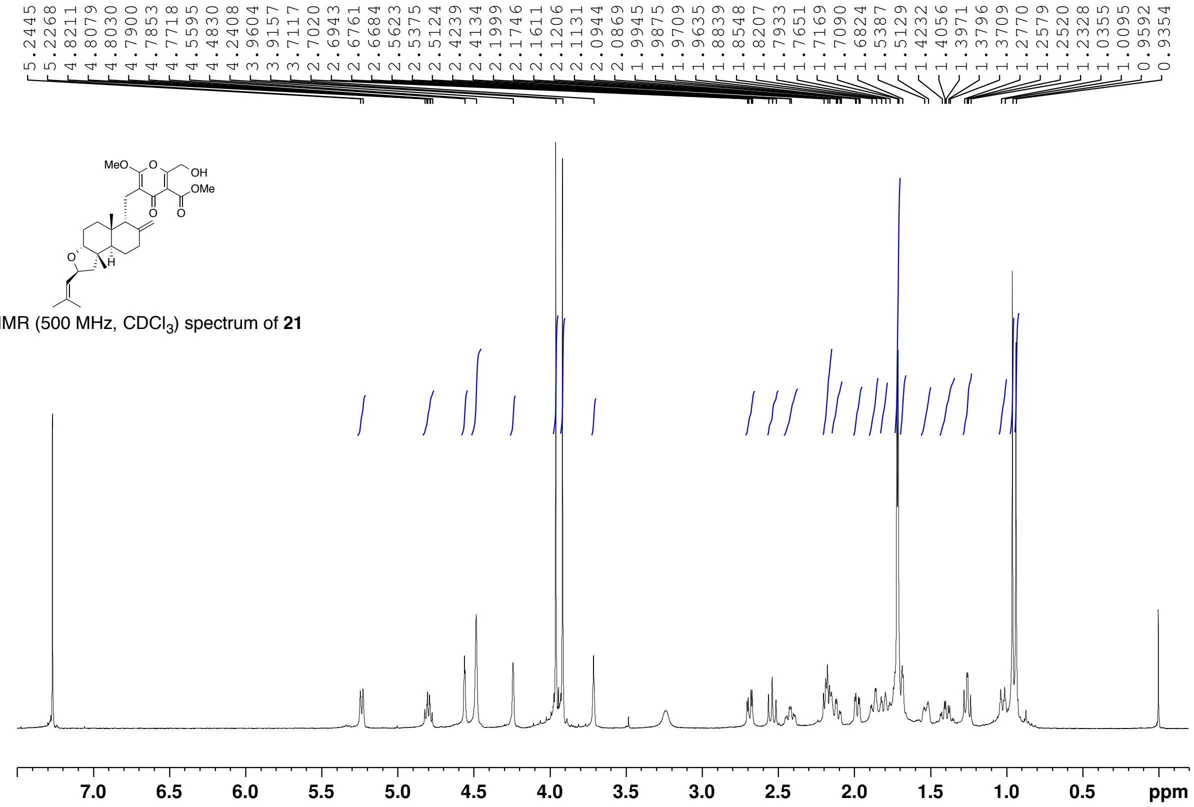

^{1}H NMR (500 MHz, $CDCl_3$) spectrum of **21**

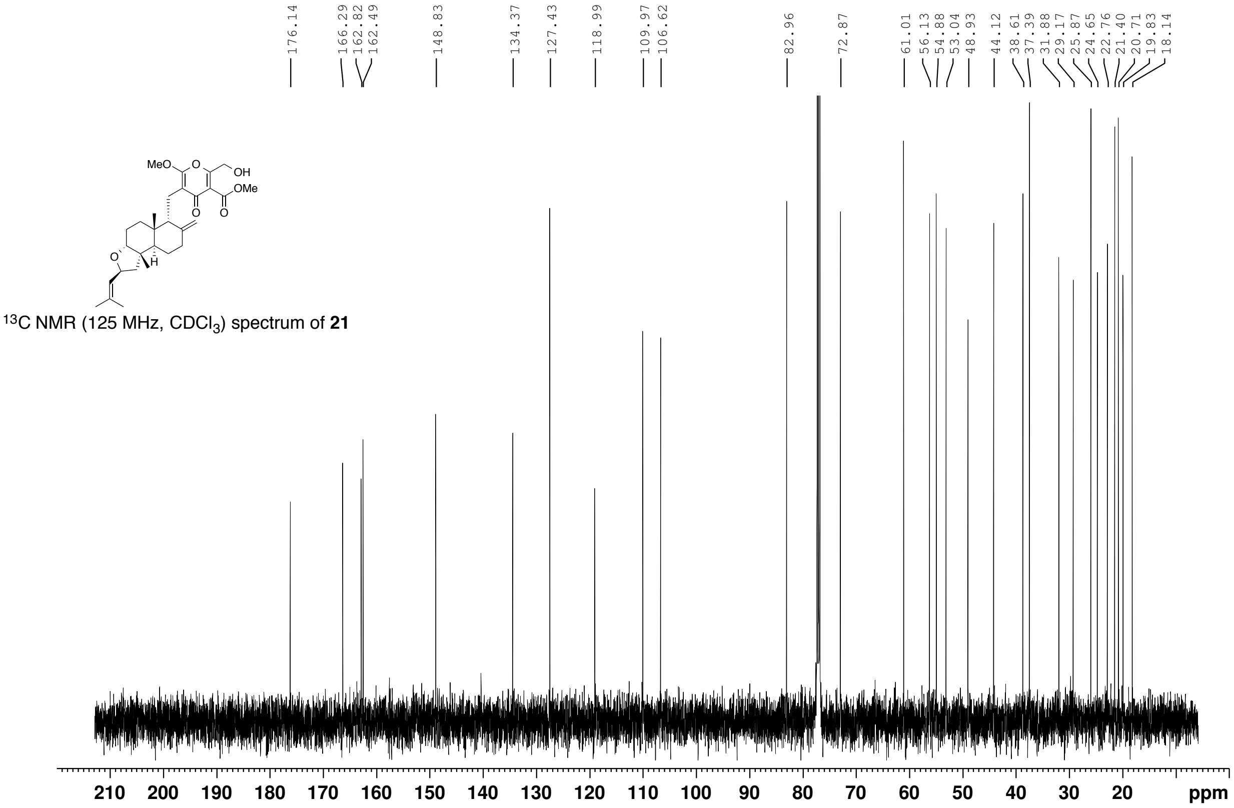

^{13}C NMR (125 MHz, $CDCl_3$) spectrum of **21**

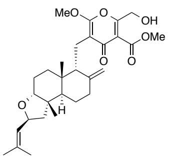

^1H-^1H COSY ($CDCl_3$) spectrum of **21**

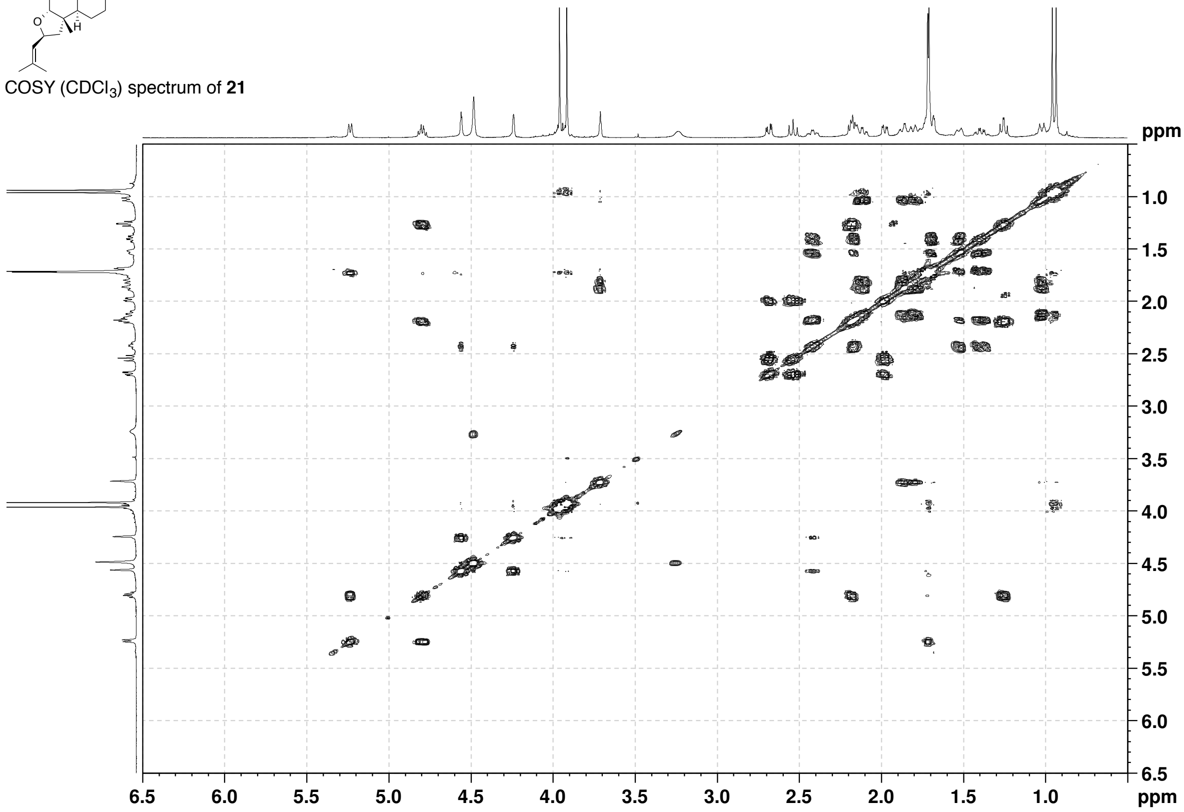

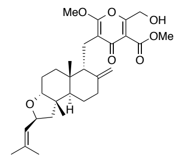

HMBC ($CDCl_3$) spectrum of **21**

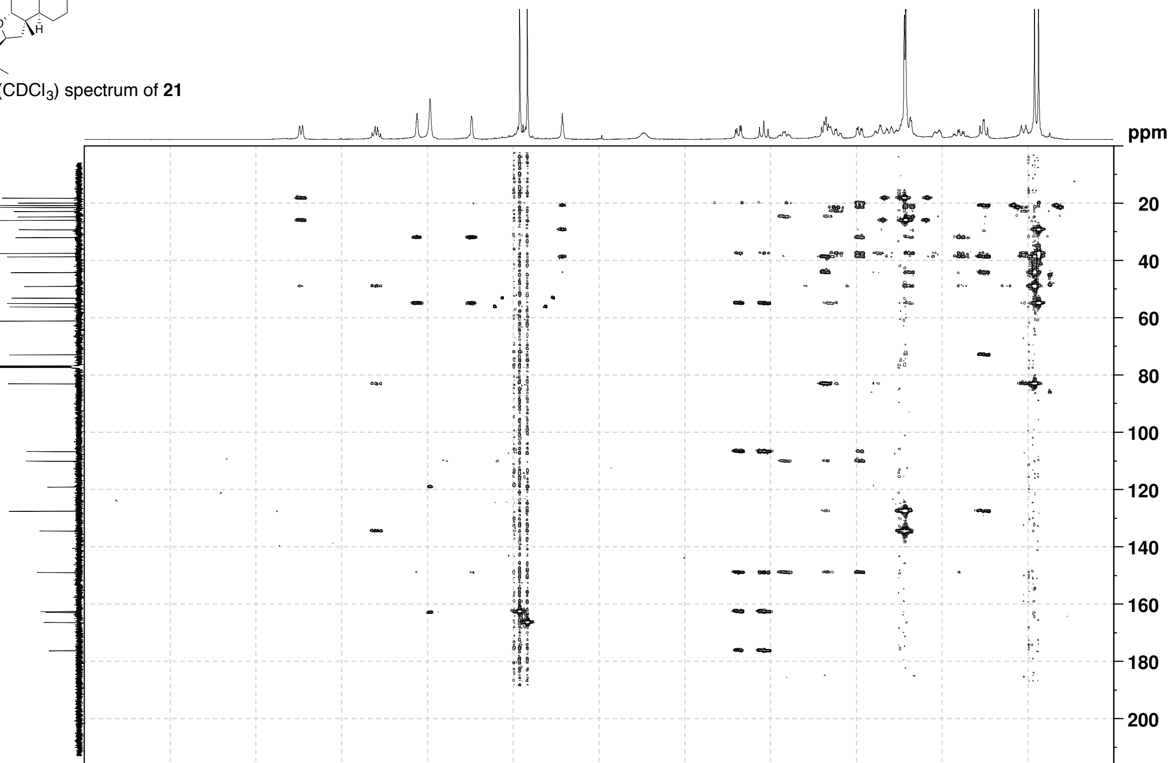

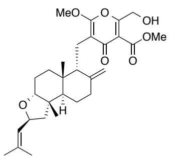

HSQC ($CDCl_3$) spectrum of **21**

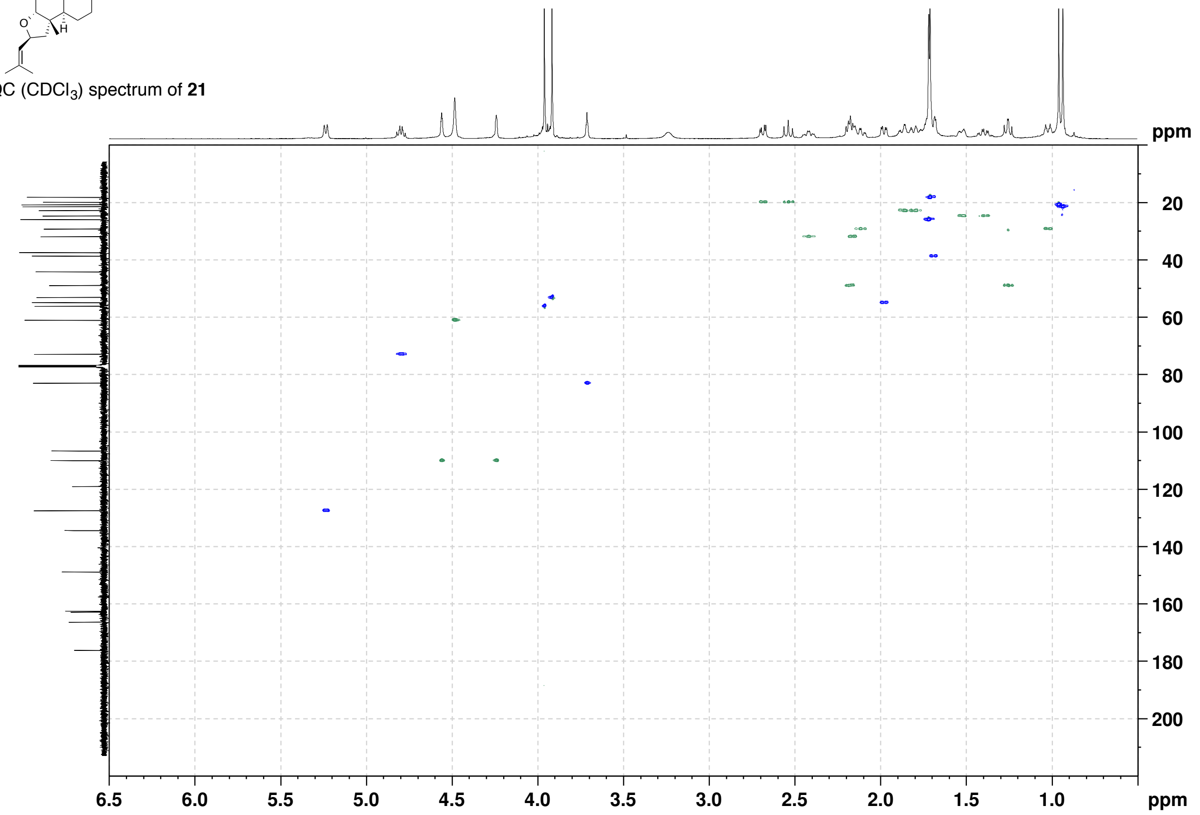

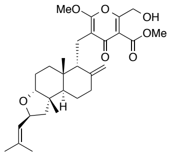

NOESY ($CDCl_3$) spectrum of **21**

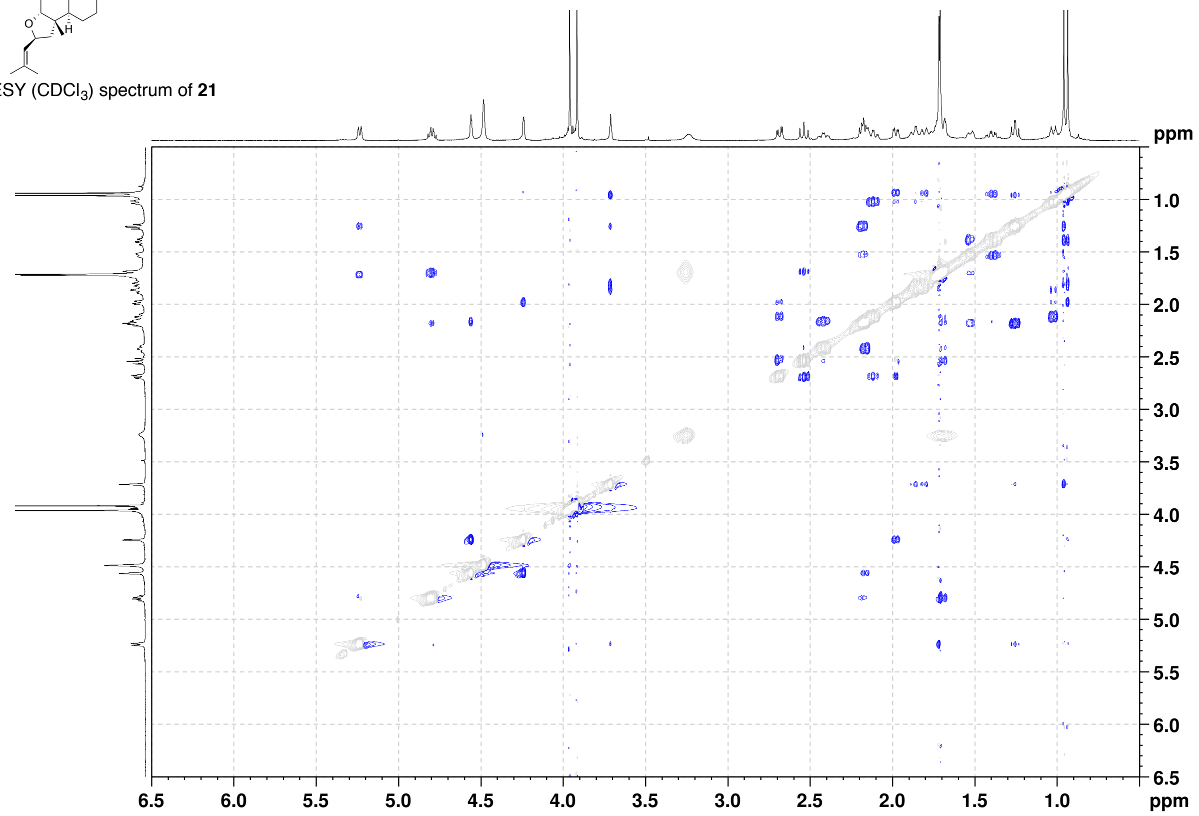

Supplementary Fig. 71. NMR spectra of 21 ($CDCl_3$)

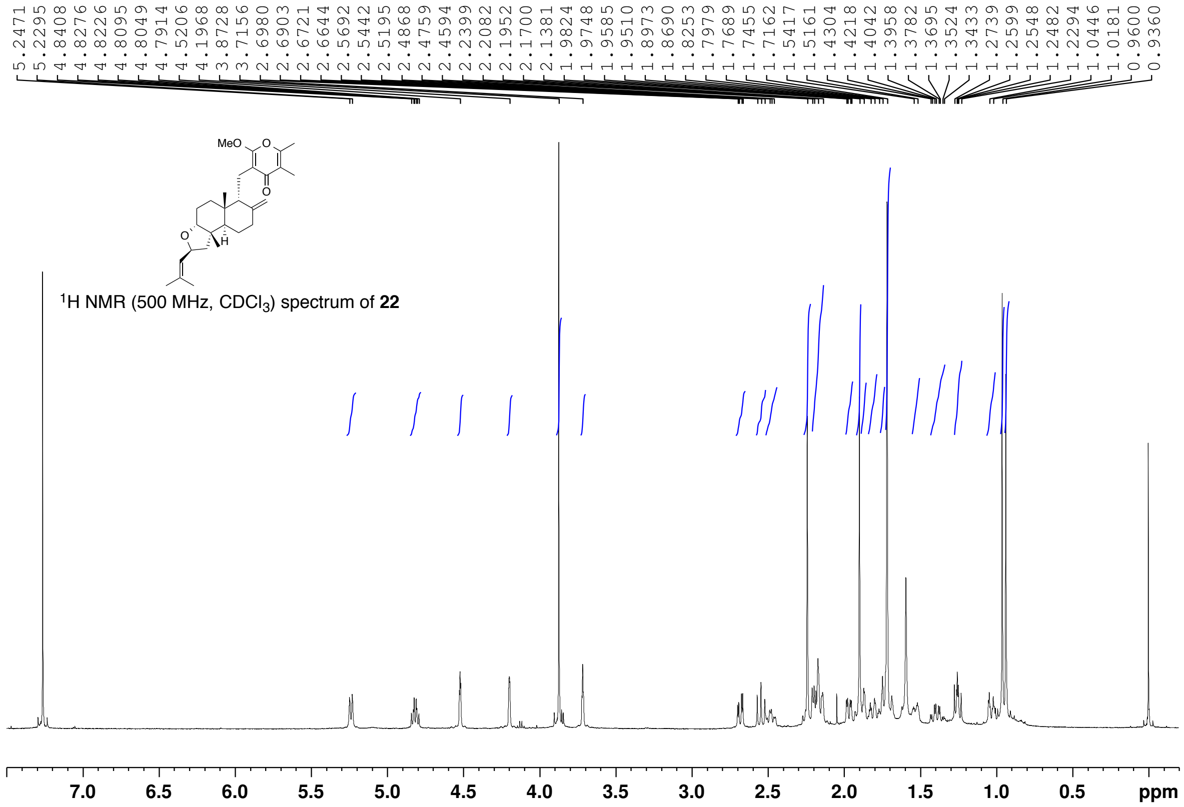

^{1}H NMR (500 MHz, $CDCl_3$) spectrum of **22**

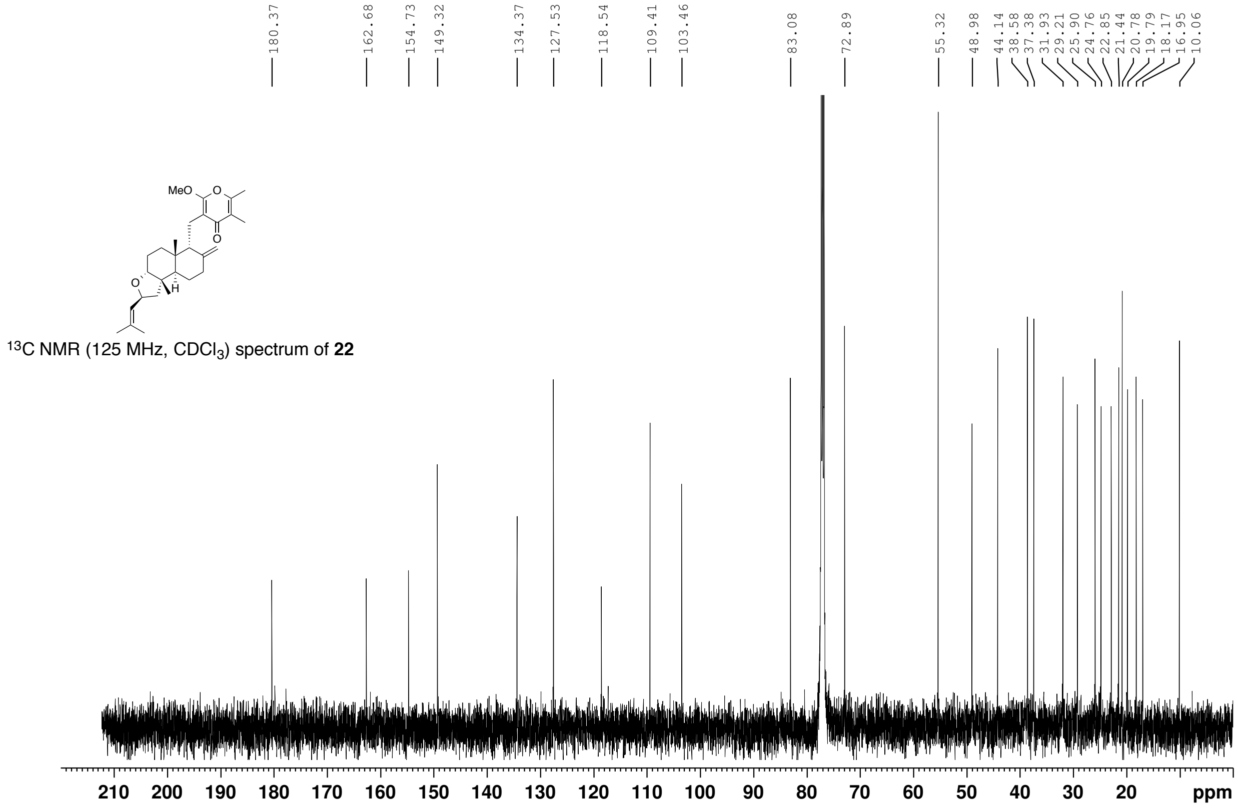

^{13}C NMR (125 MHz, $CDCl_3$) spectrum of **22**

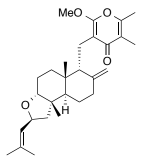

^{1}H-^{1}H COSY ($CDCl_3$) spectrum of **22**

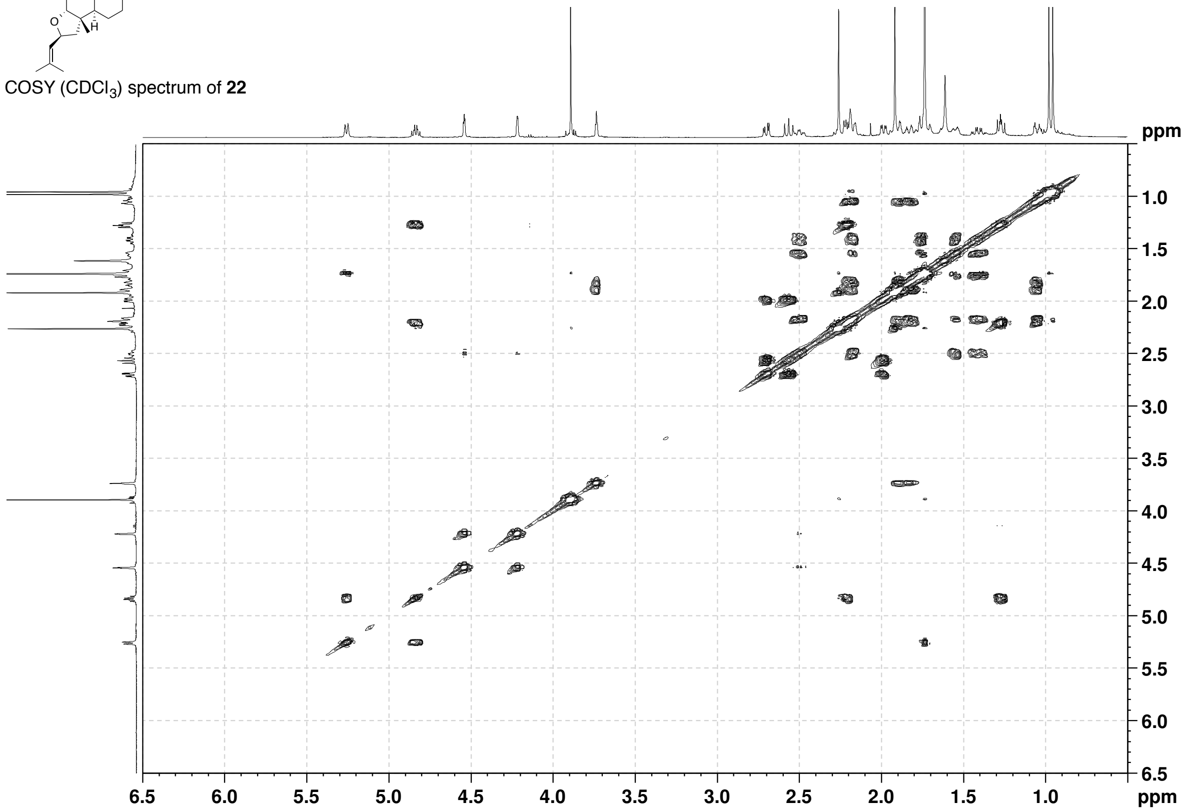

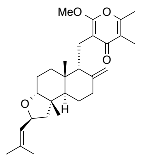

HMBC ($CDCl_3$) spectrum of **22**

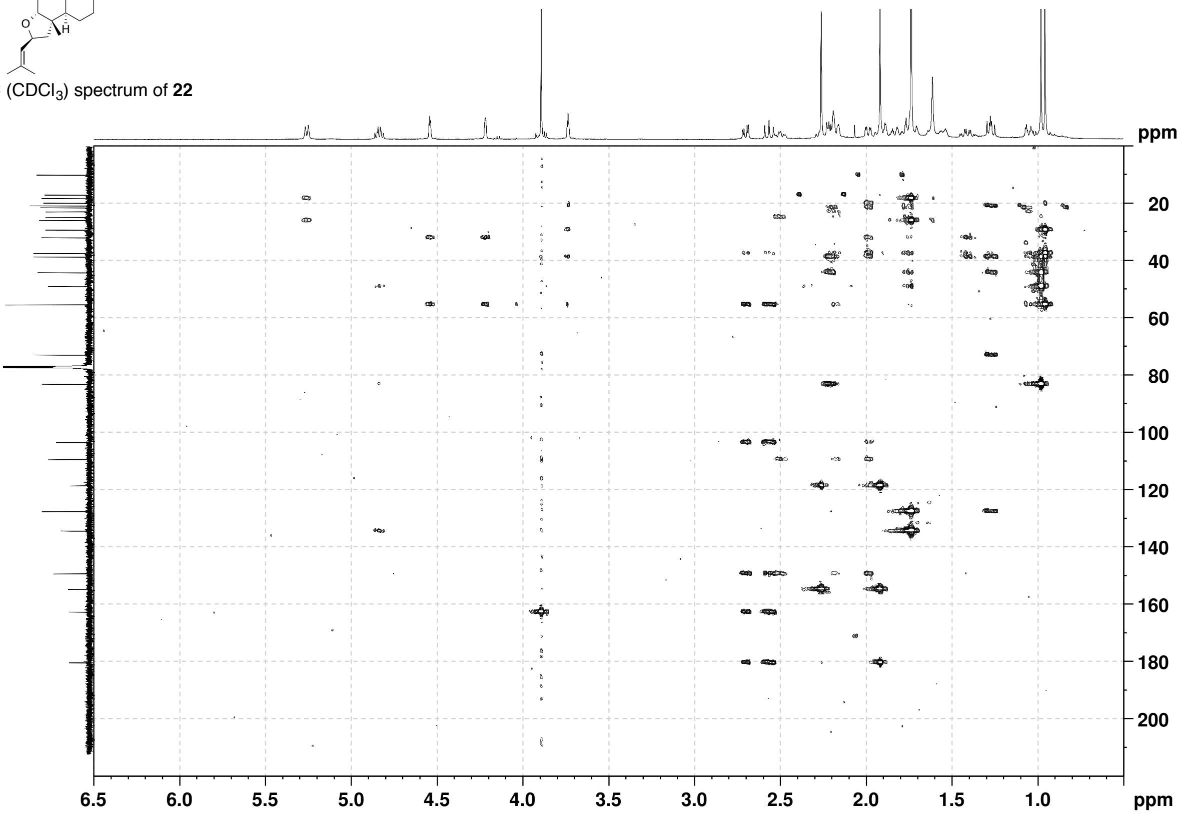

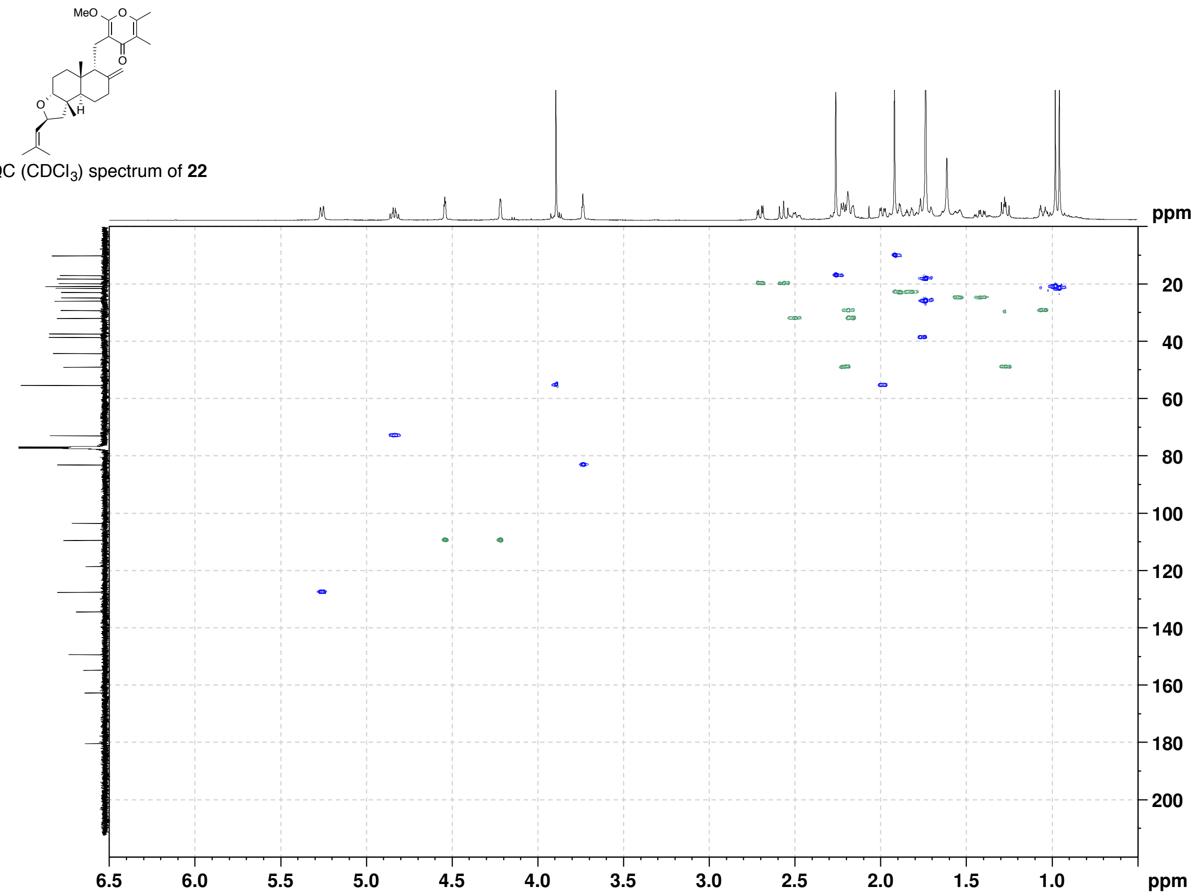

HSQC ($CDCl_3$) spectrum of **22**

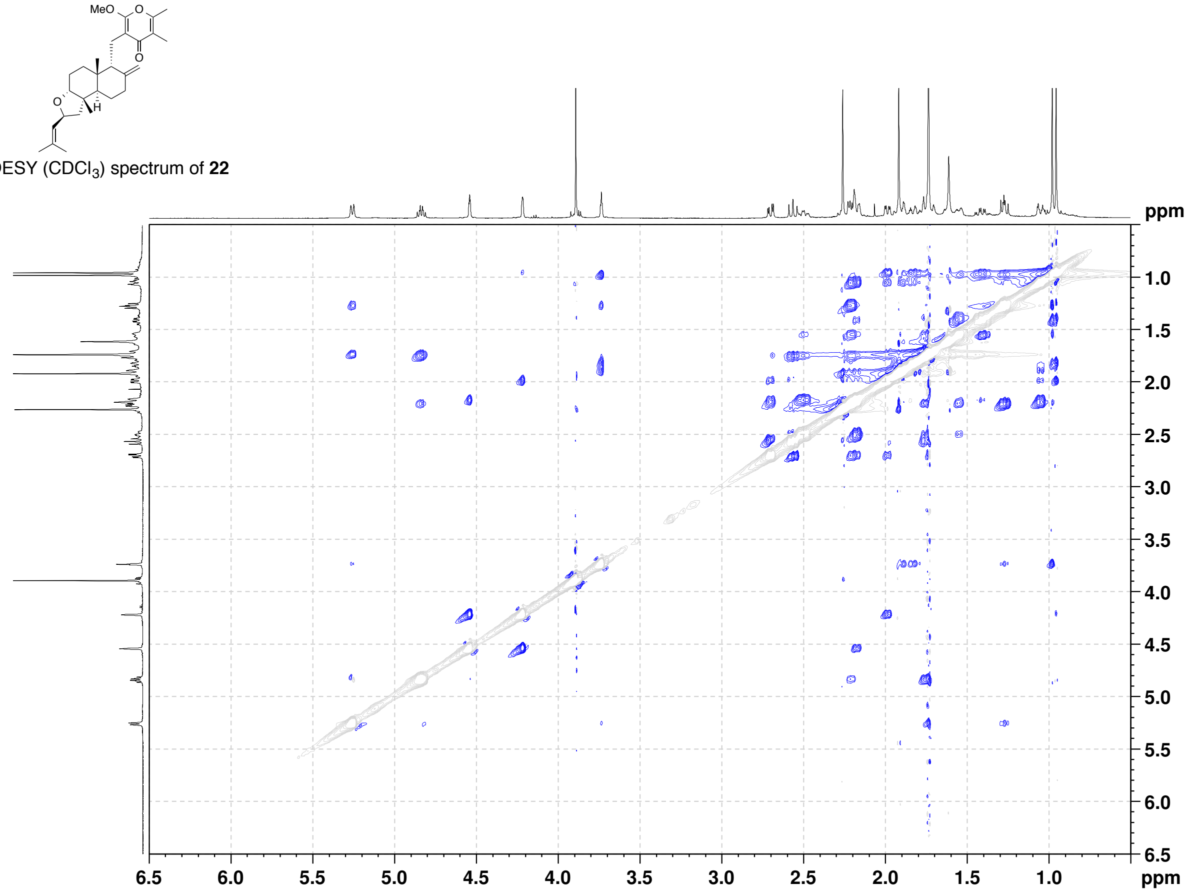

NOESY ($CDCl_3$) spectrum of **22**

Supplementary Fig. 72. NMR spectra of 22 ($CDCl_3$)

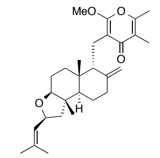

^{1}H NMR (500 MHz, $CDCl_3$) spectrum of **23**

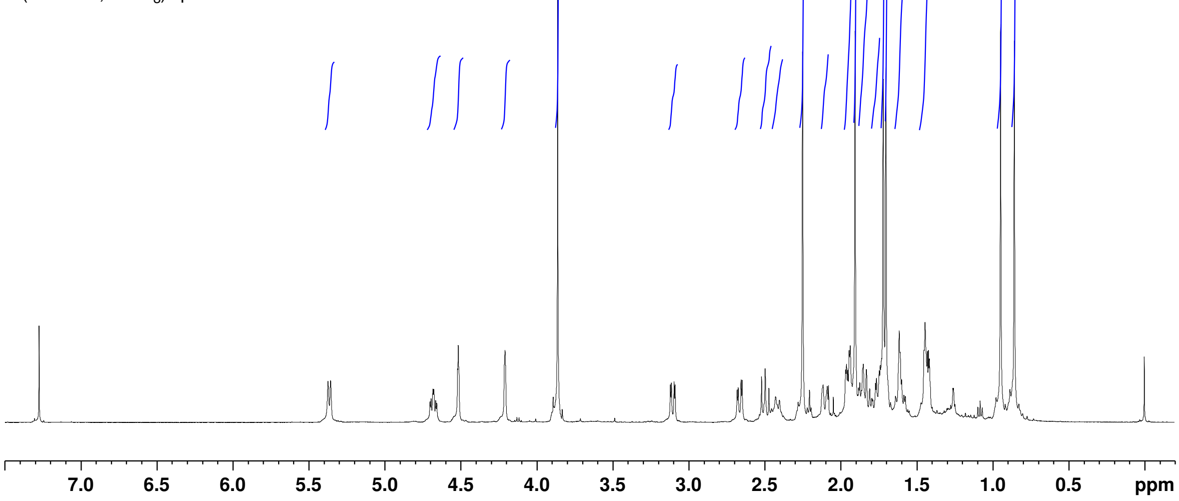

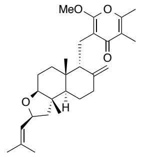

^{13}C NMR (125 MHz, $CDCl_3$) spectrum of **23**

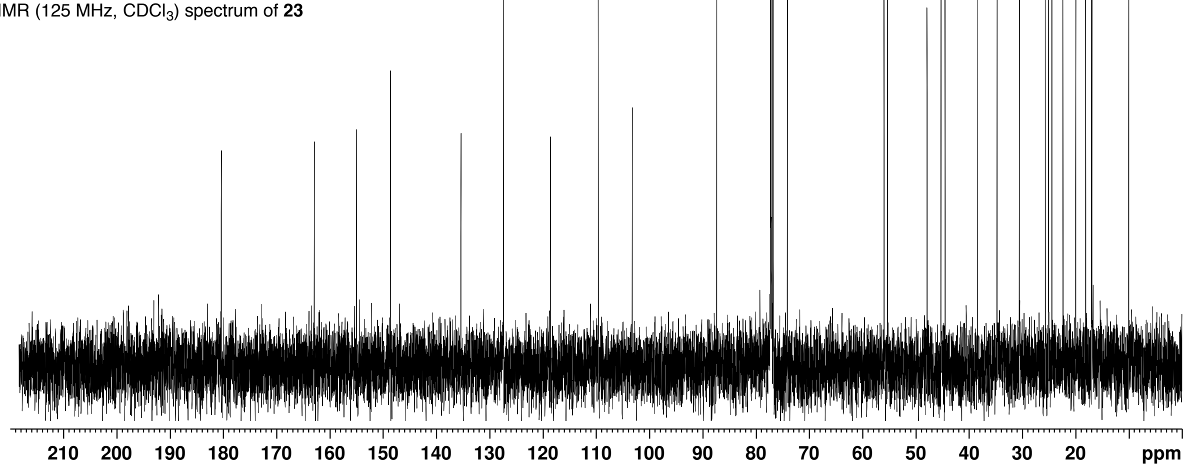

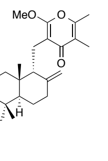

^{1}H-^{1}H COSY ($CDCl_3$) spectrum of **23**

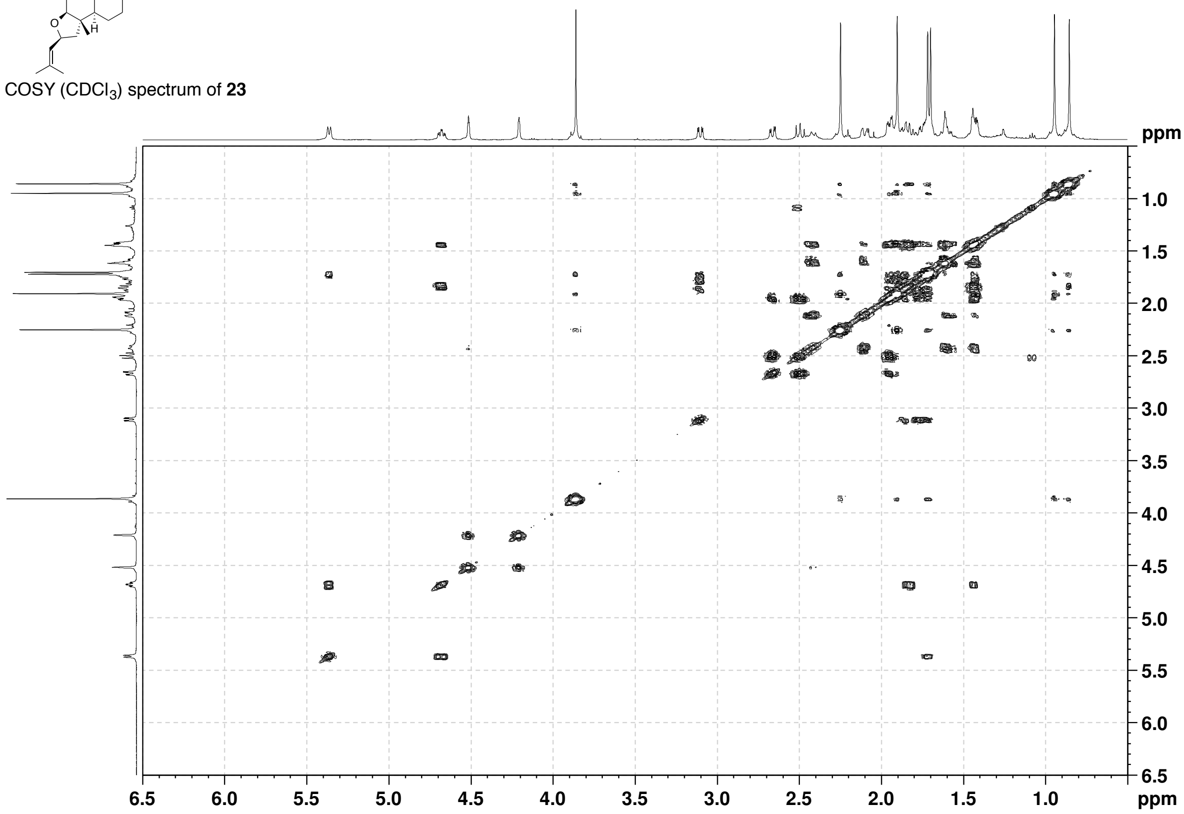

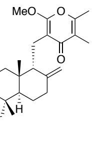

HMBC ($CDCl_3$) spectrum of **23**

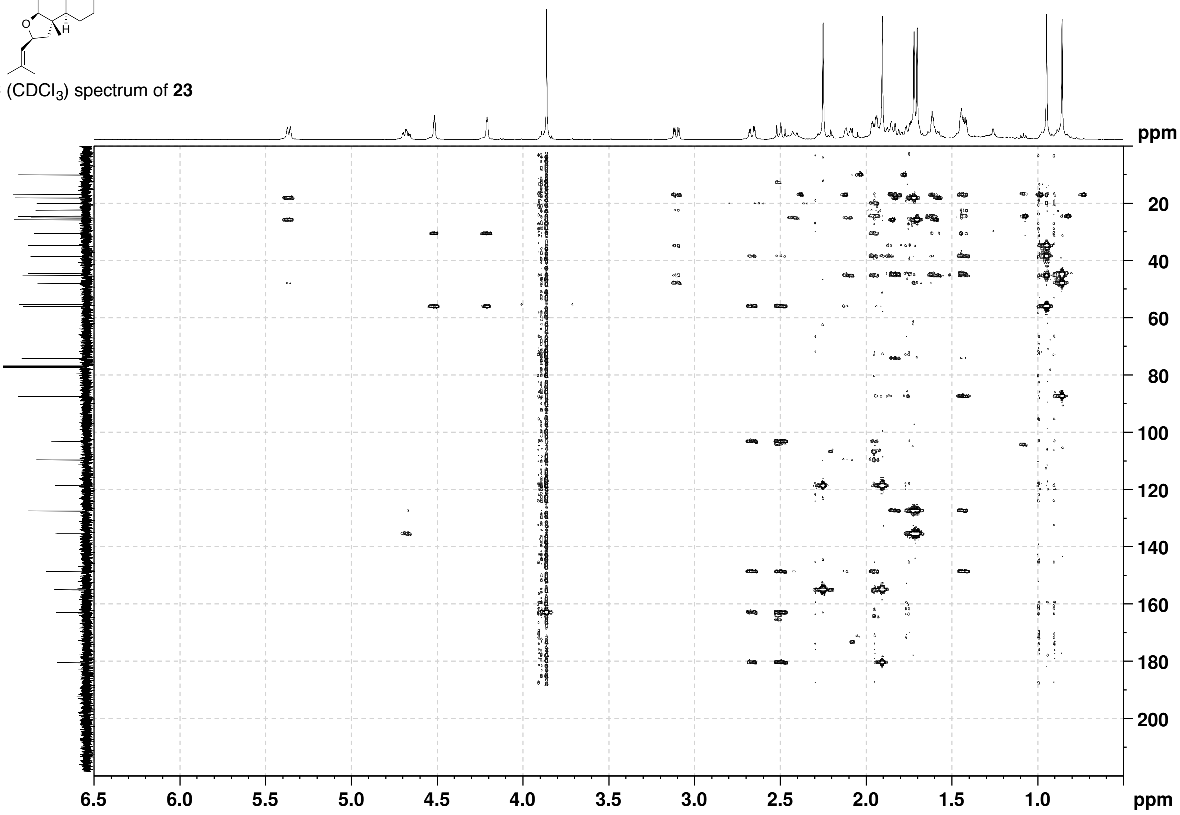

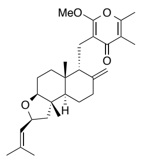

HSQC ($CDCl_3$) spectrum of **23**

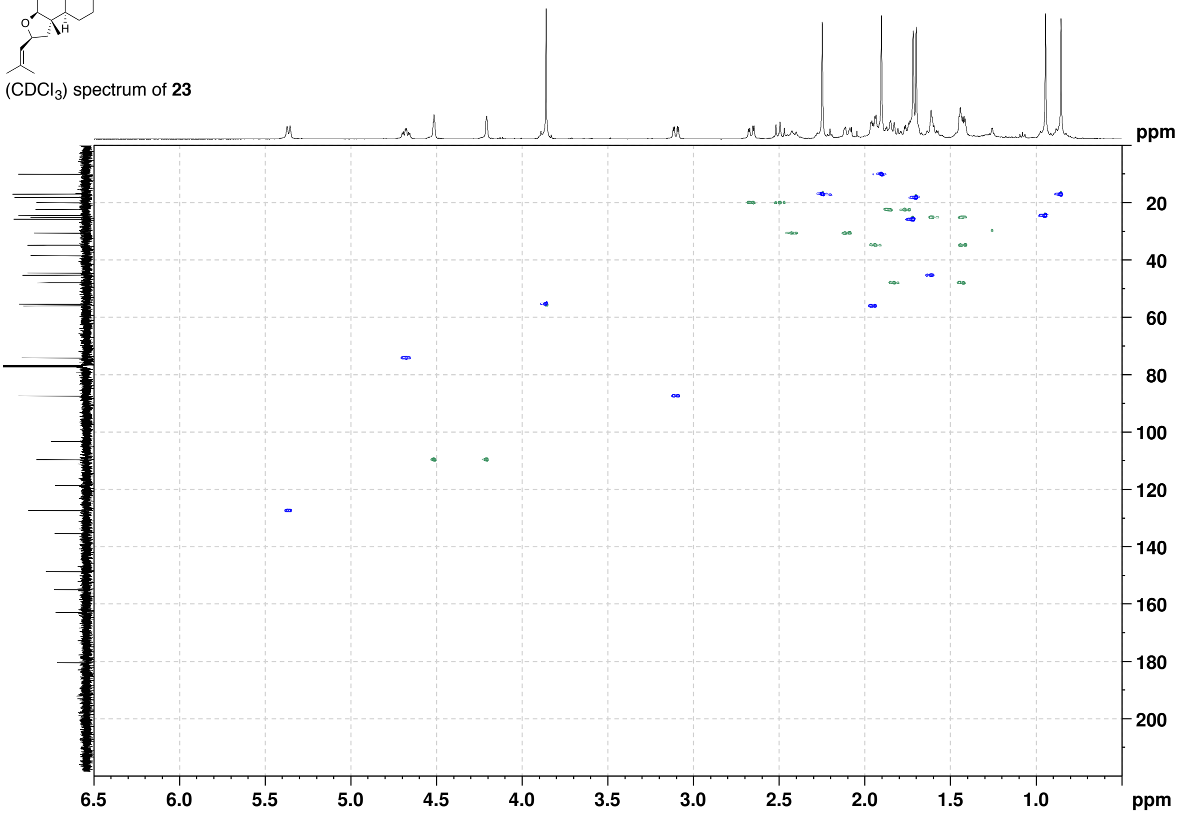

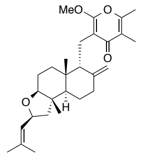

NOESY ($CDCl_3$) spectrum of **23**

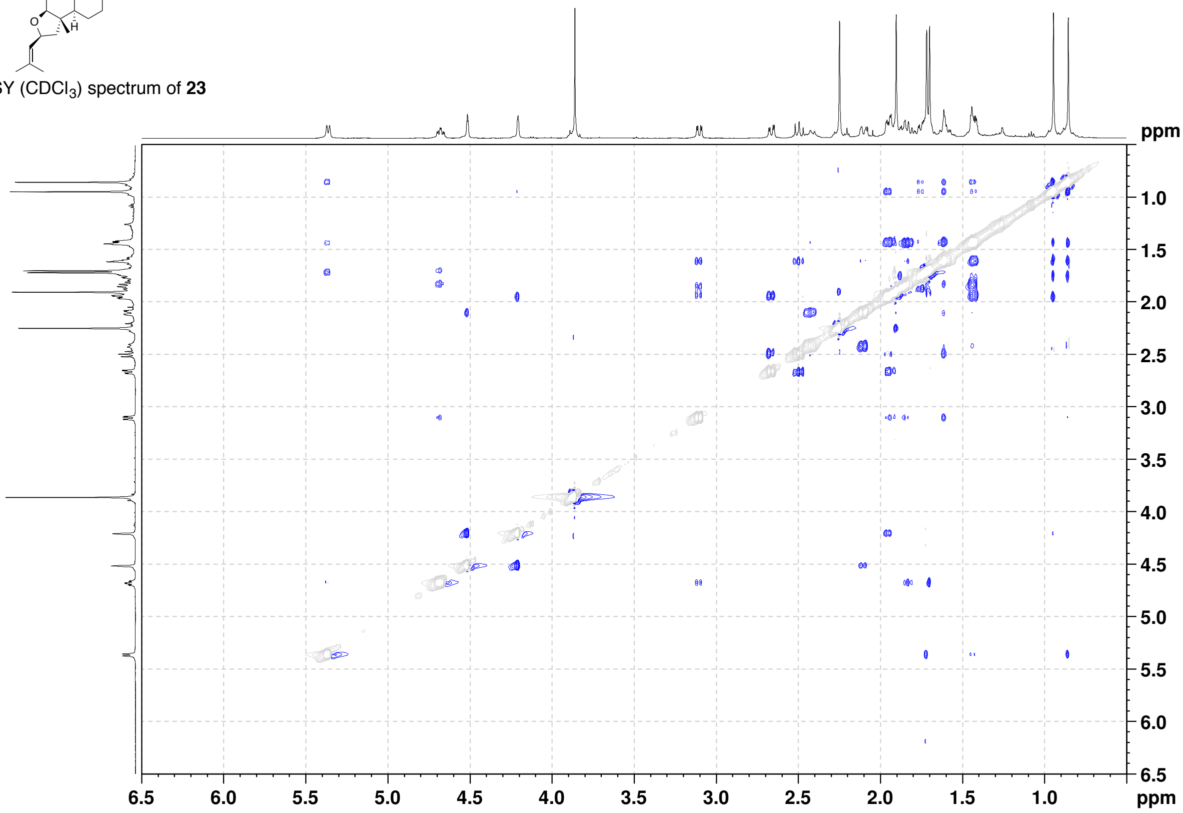

Supplementary Fig. 73. NMR spectra of 23 ($CDCl_3$)

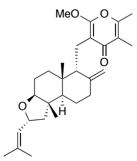

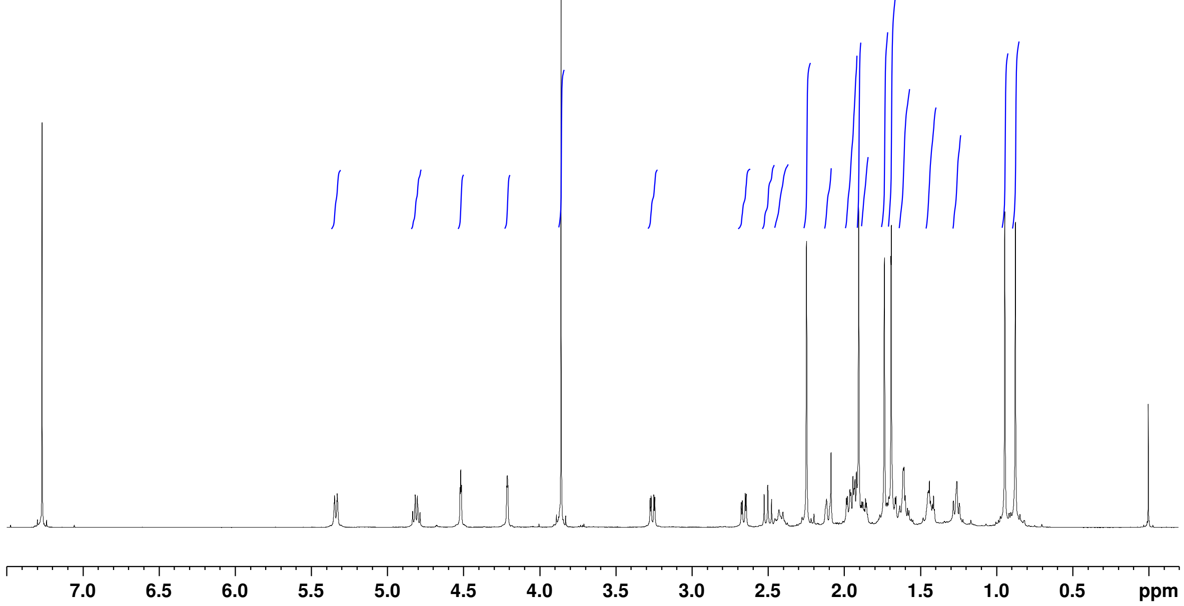

^1H NMR (500 MHz, $CDCl_3$) spectrum of **24**

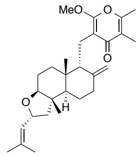

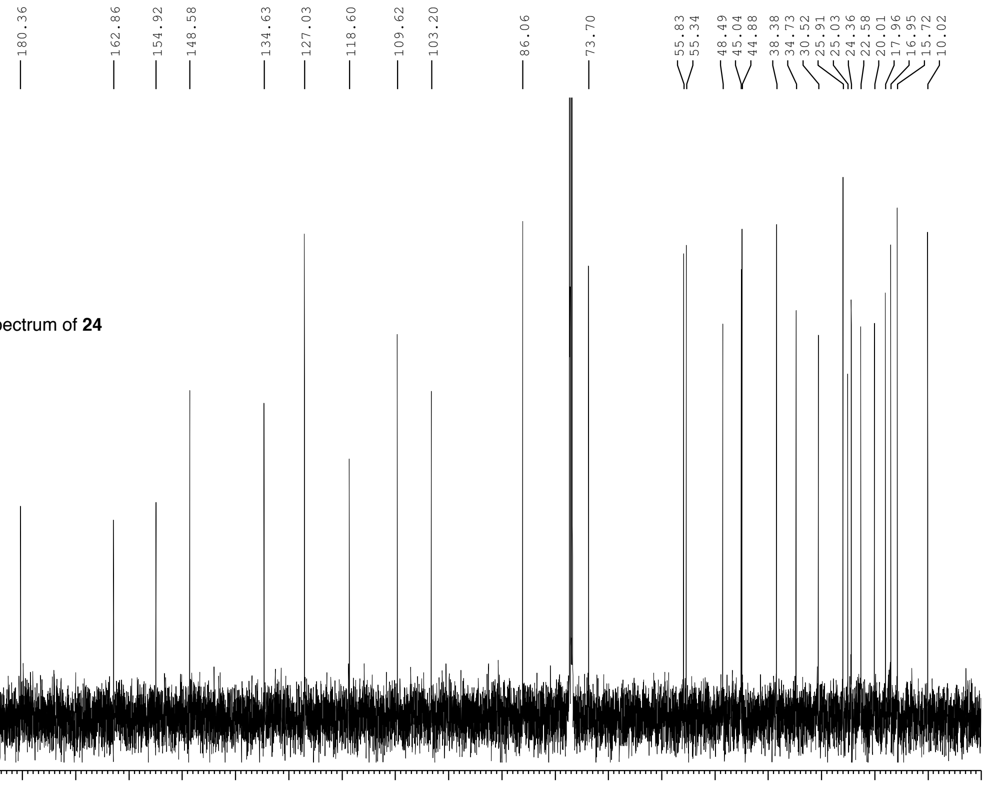

^{13}C NMR (125 MHz, $CDCl_3$) spectrum of **24**

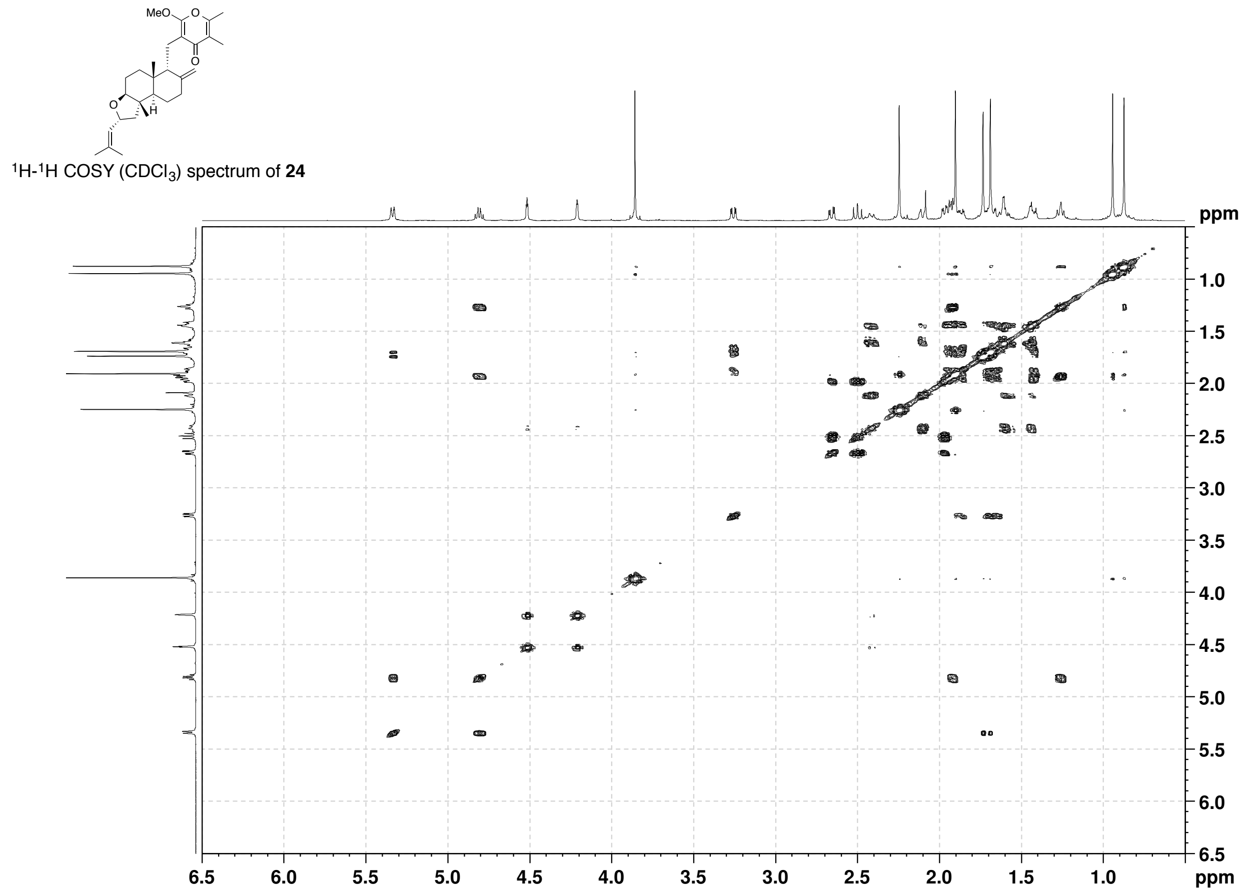

^{1}H-^{1}H COSY ($CDCl_3$) spectrum of **24**

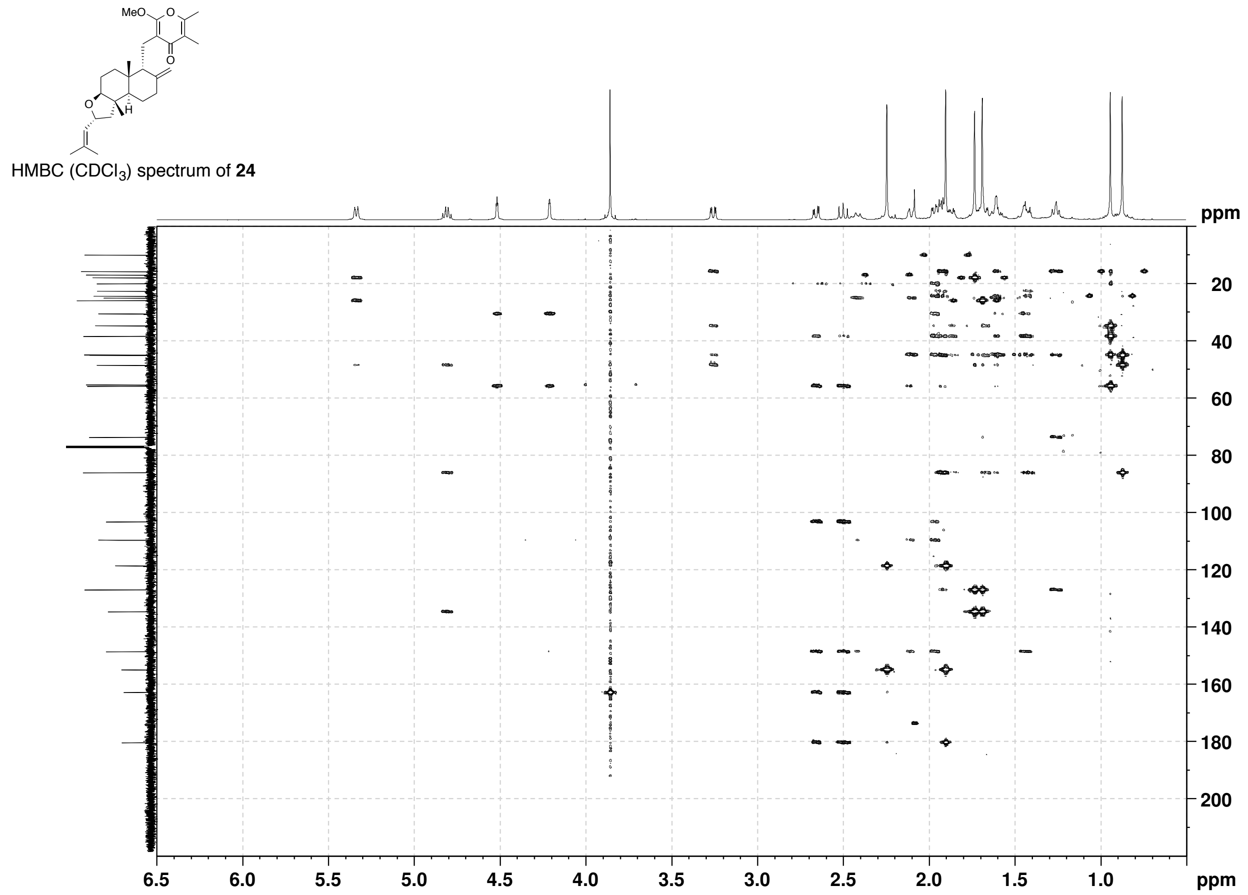

HMBC ($CDCl_3$) spectrum of **24**

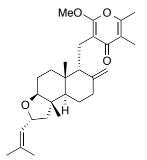

HSQC ($CDCl_3$) spectrum of **24**

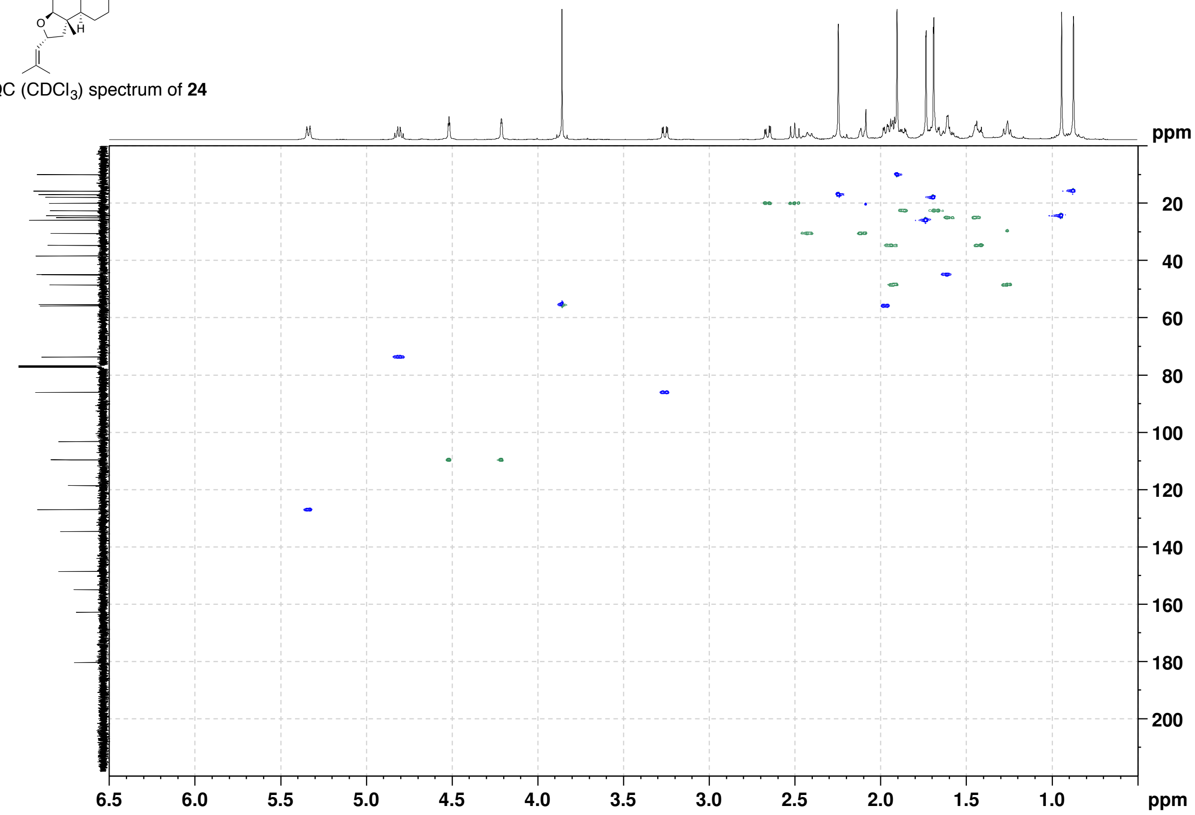

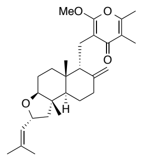

NOESY ($CDCl_3$) spectrum of **24**

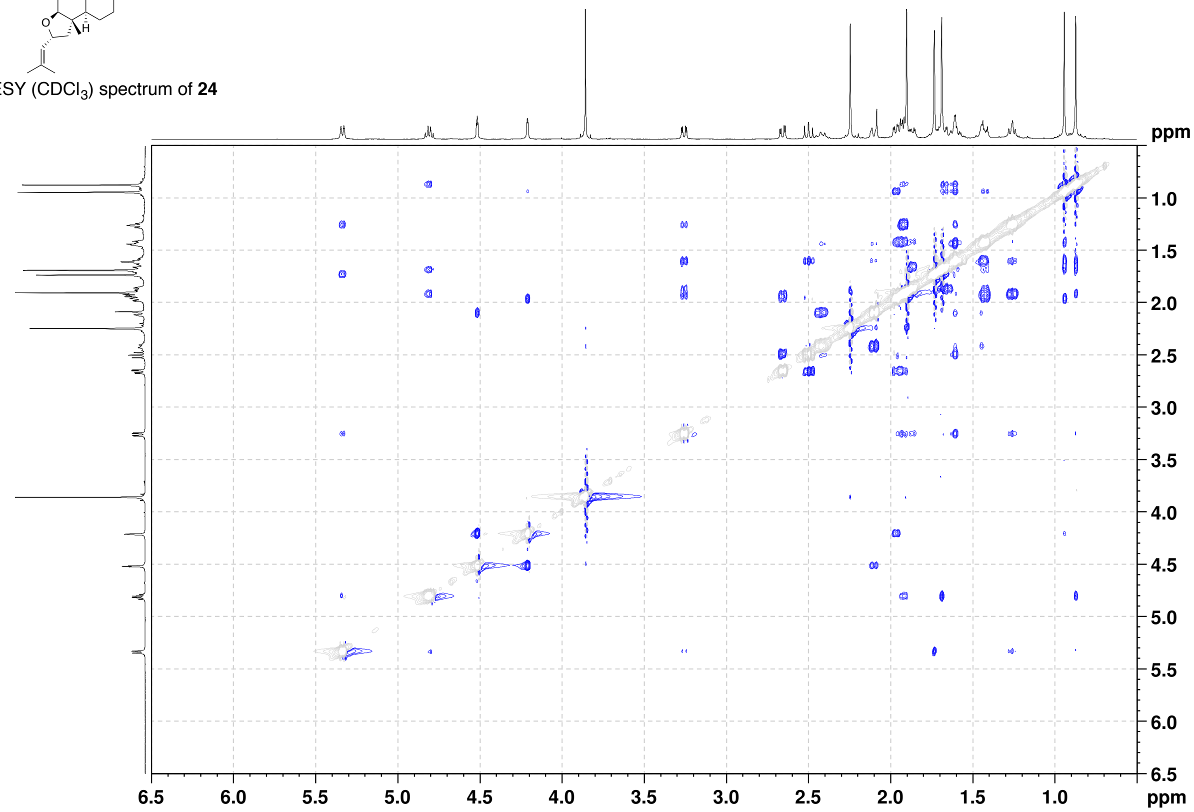

Supplementary Fig. 74. NMR spectra of 24 ($CDCl_3$)

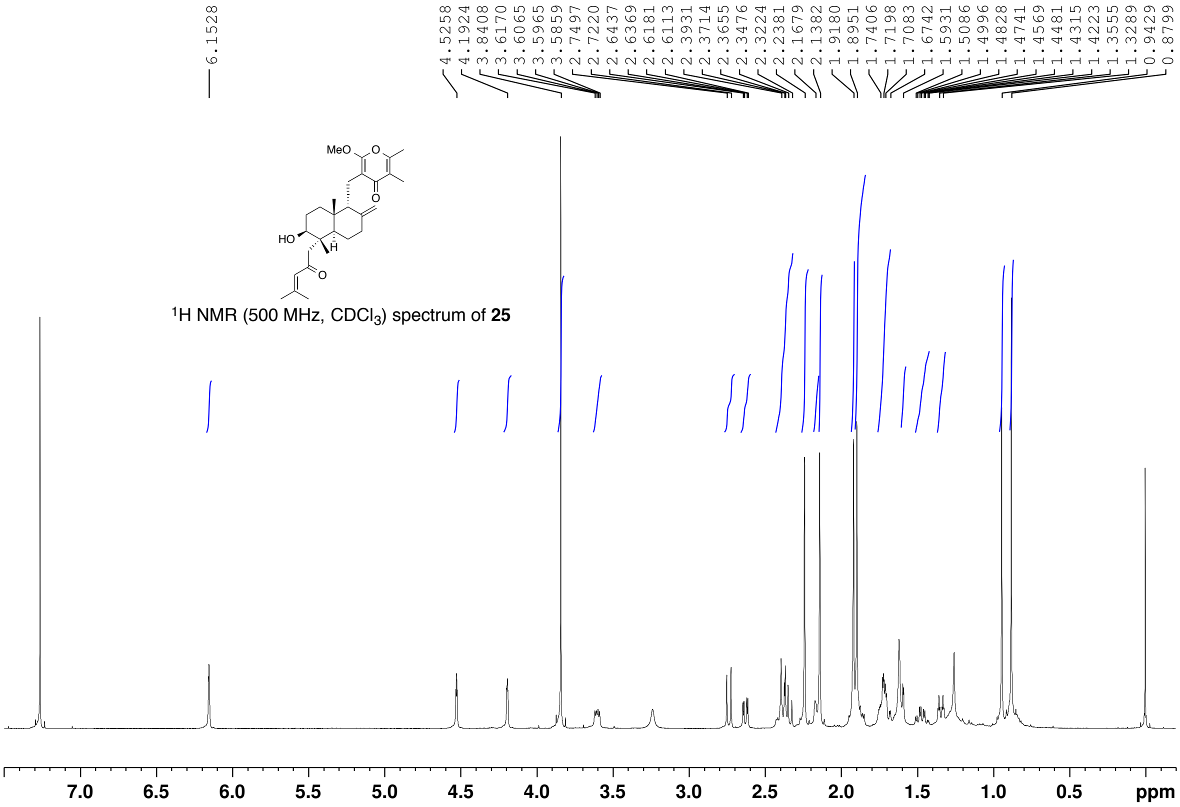

^1H NMR (500 MHz, $CDCl_3$) spectrum of **25**

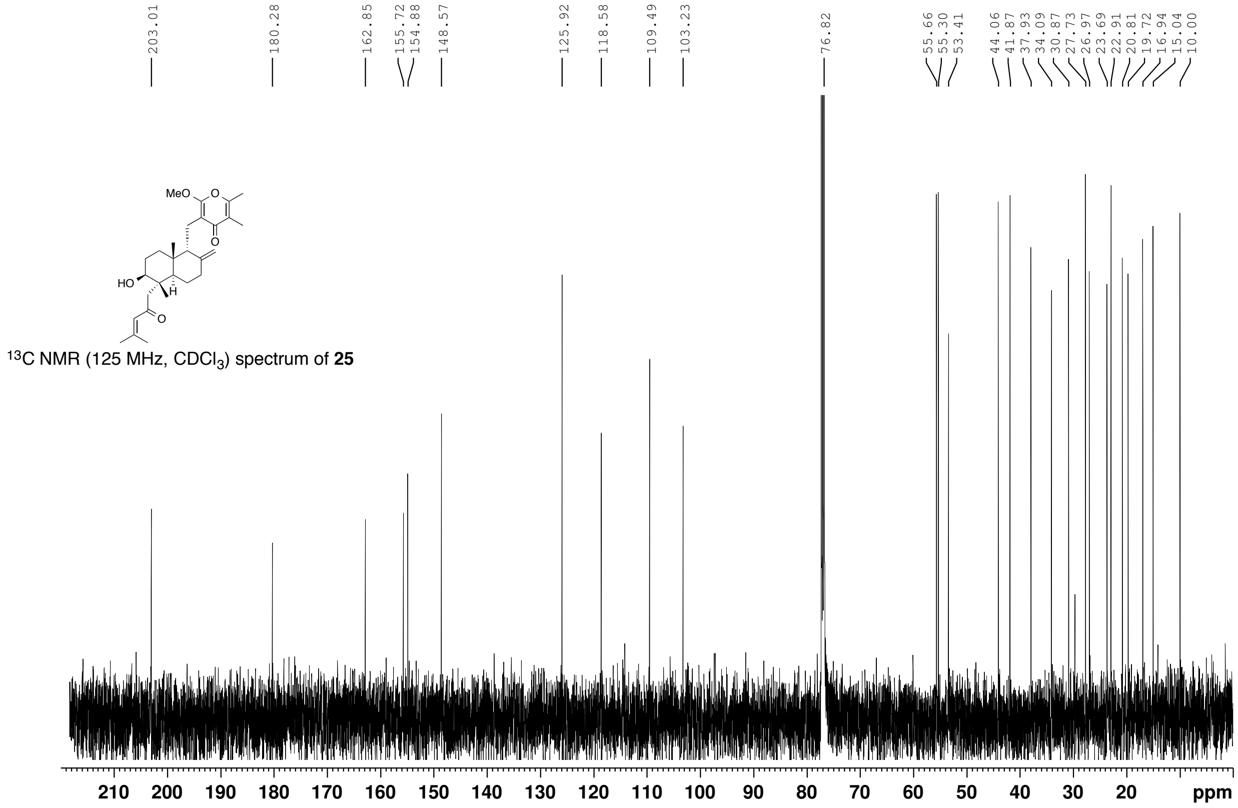

^{13}C NMR (125 MHz, $CDCl_3$) spectrum of **25**

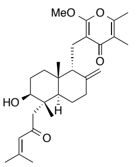

^{1}H-^{1}H COSY ($CDCl_3$) spectrum of **25**

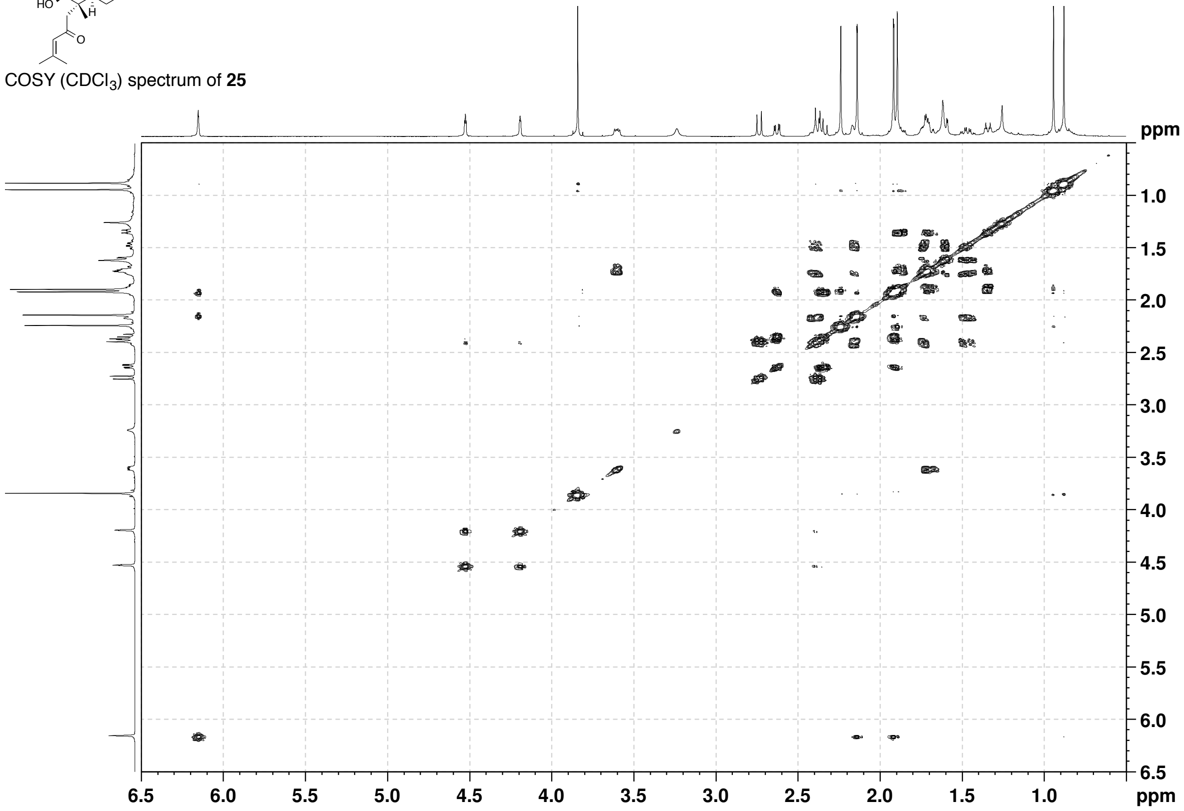

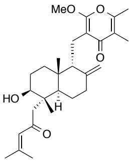

HMBC ($CDCl_3$) spectrum of **25**

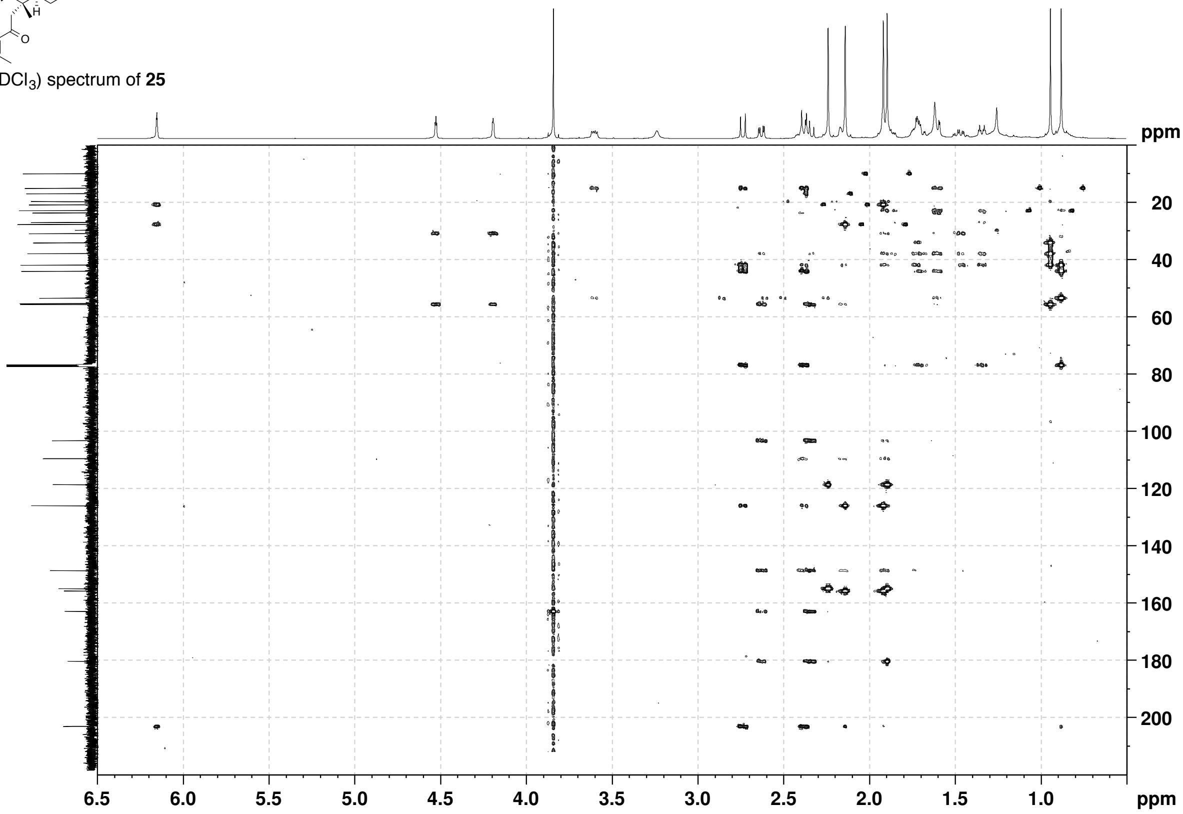

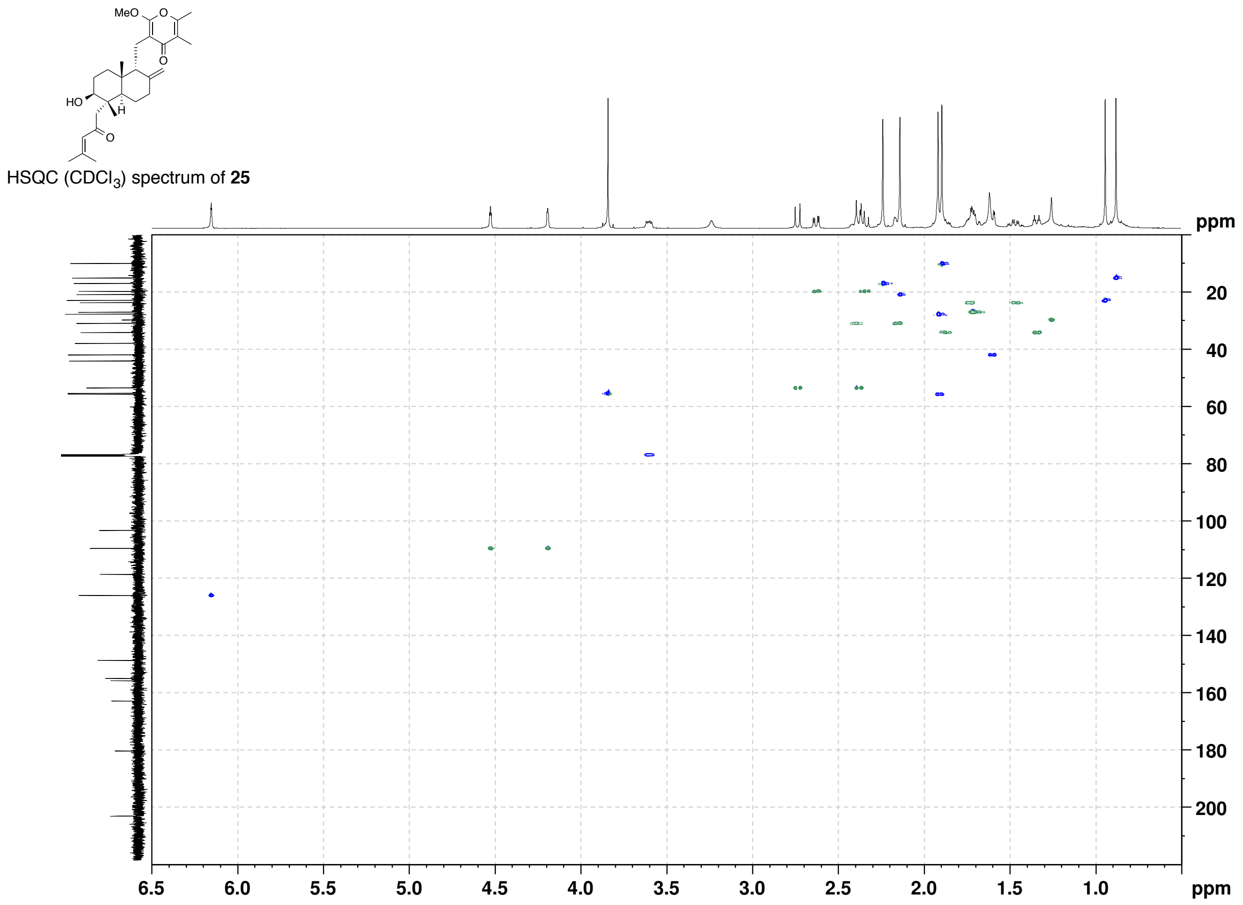

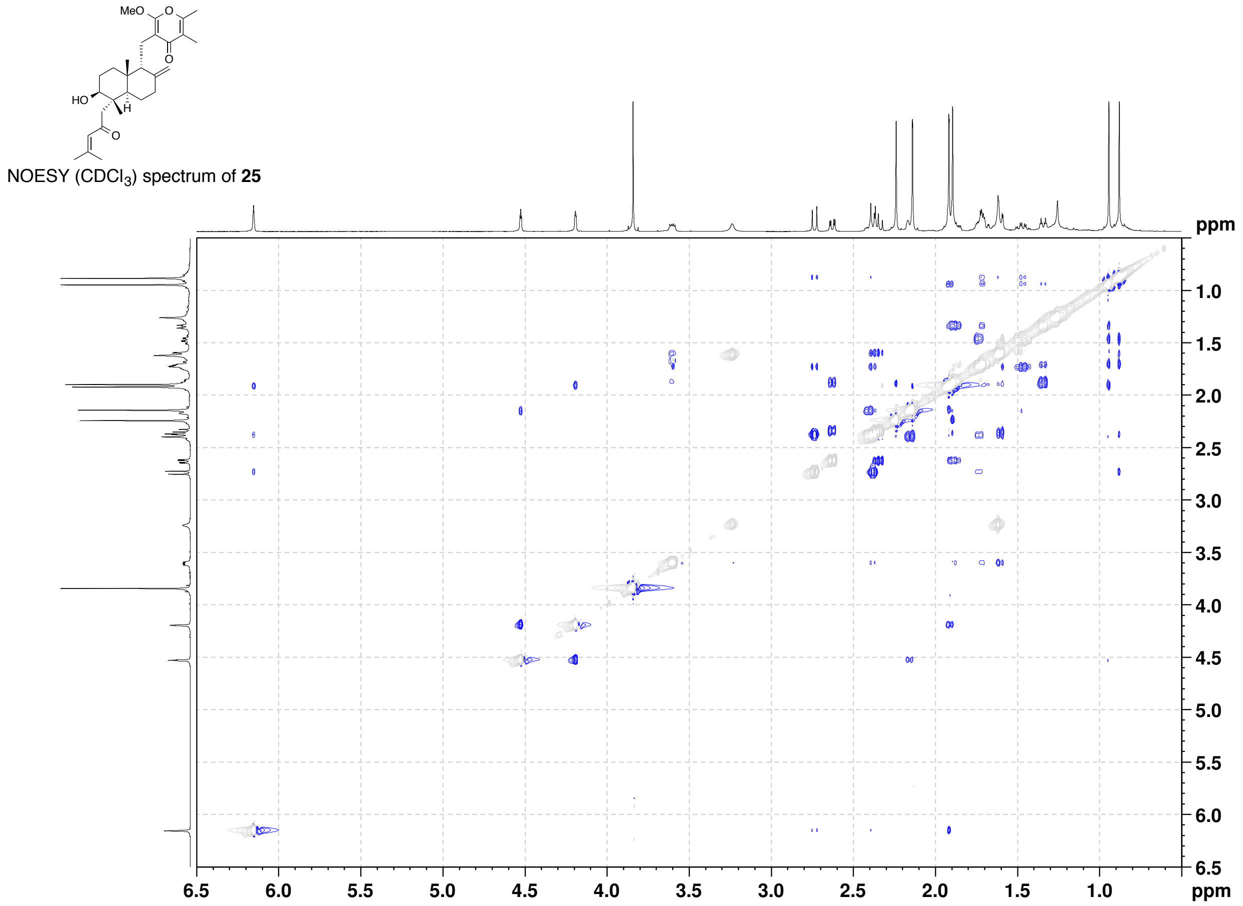

Supplementary Fig. 75. NMR spectra of 25 ($CDCl_3$)

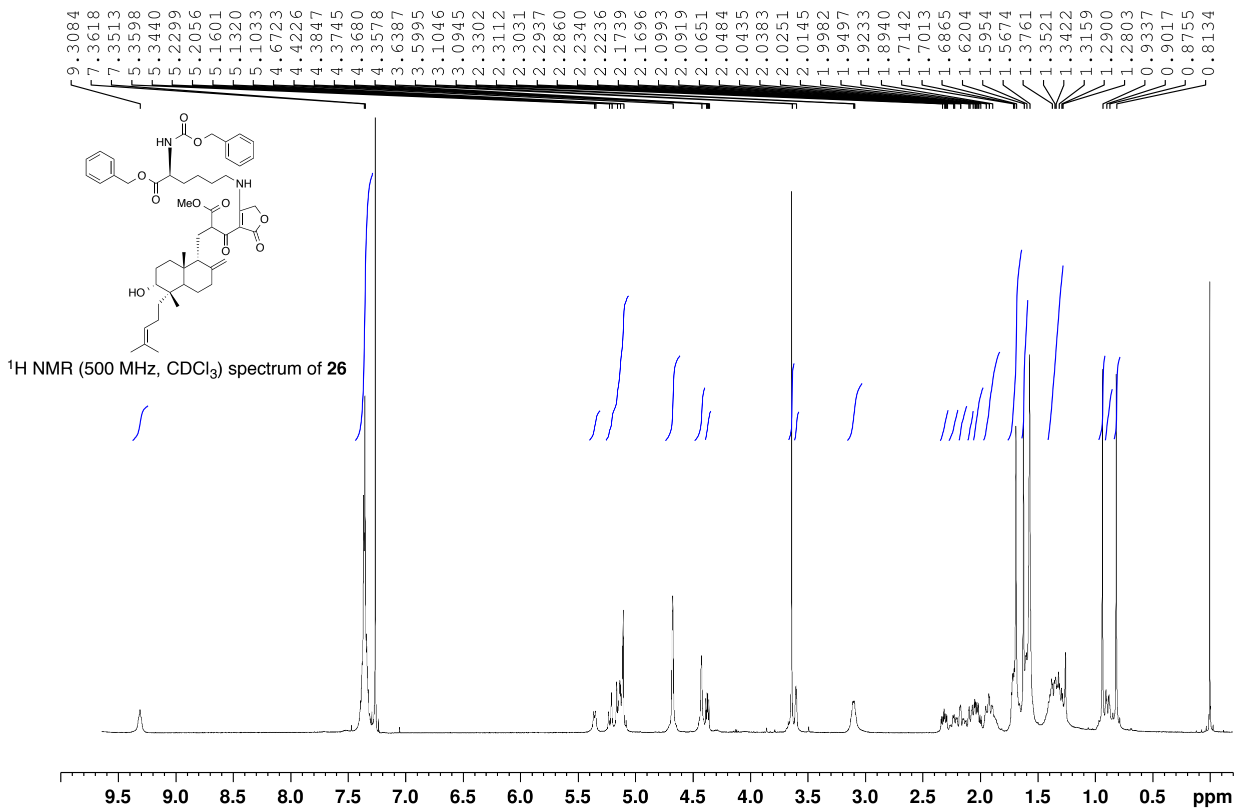

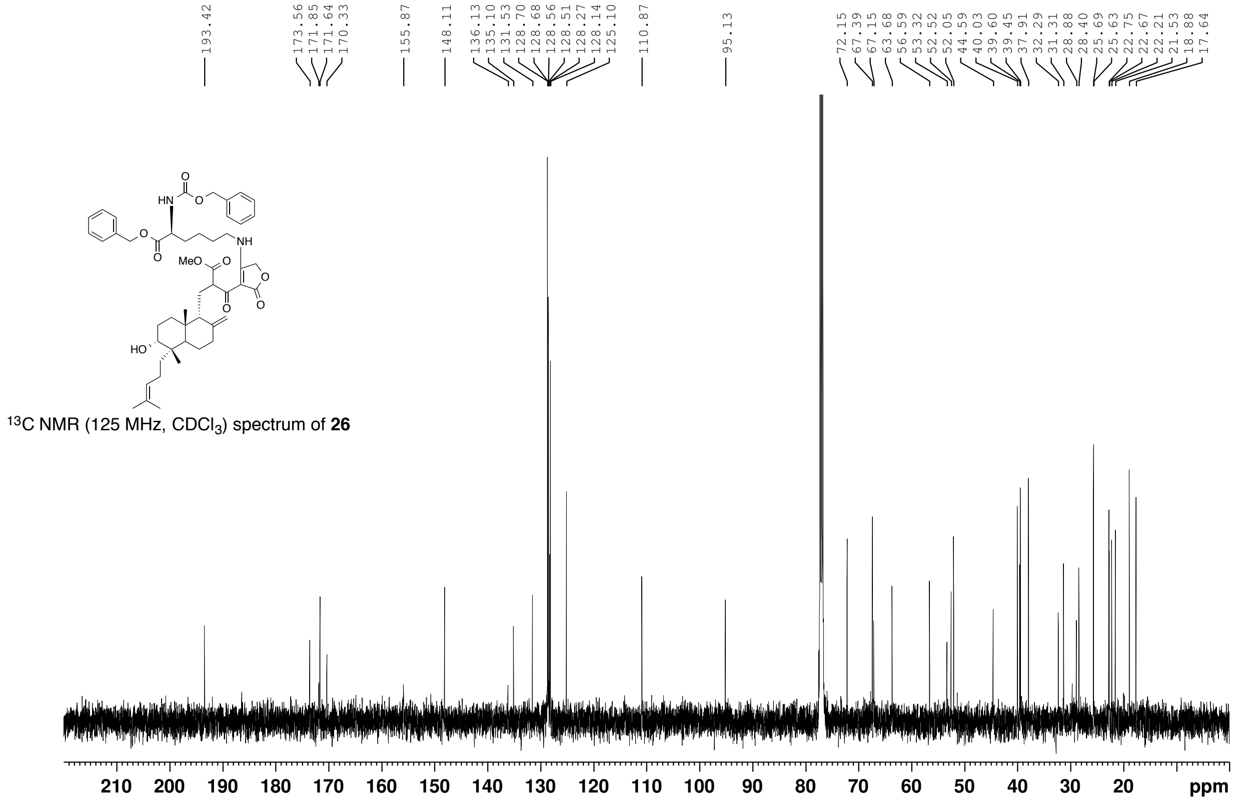

Supplementary Fig. 76. NMR spectra of 26 ($CDCl_3$)

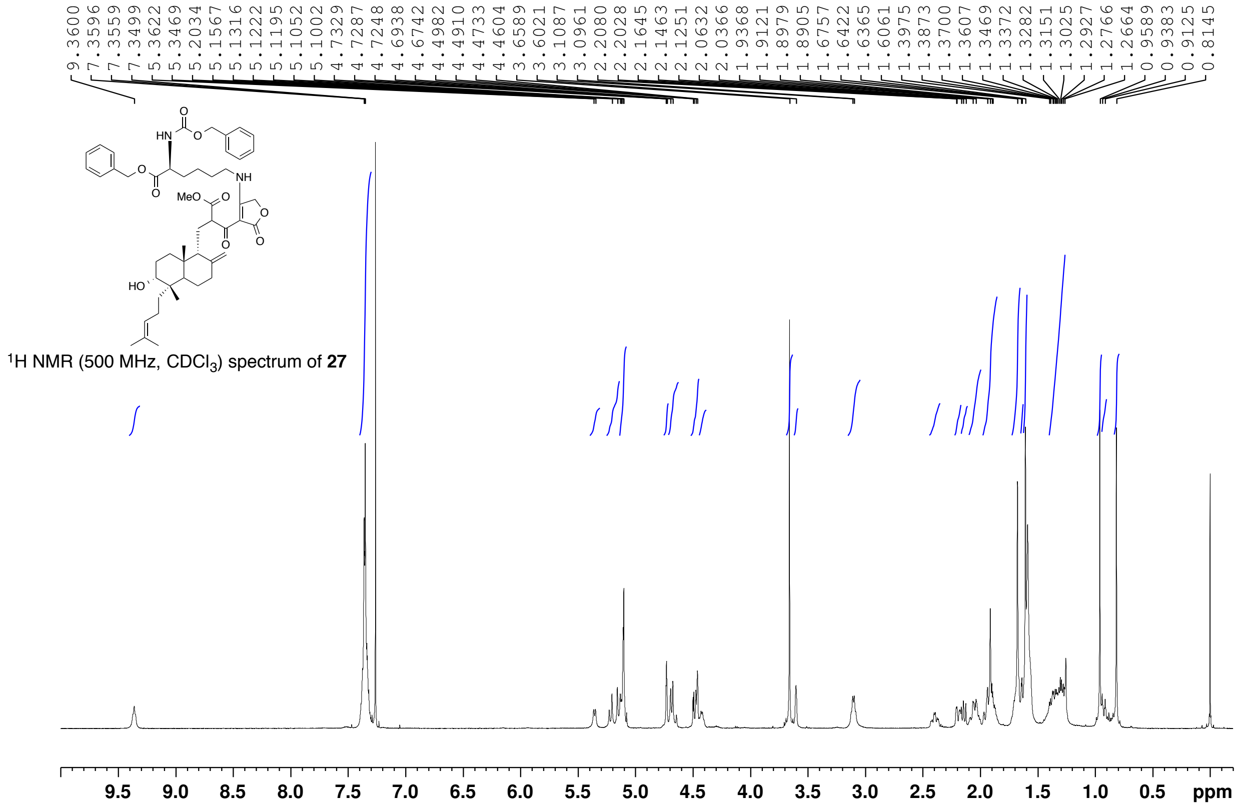

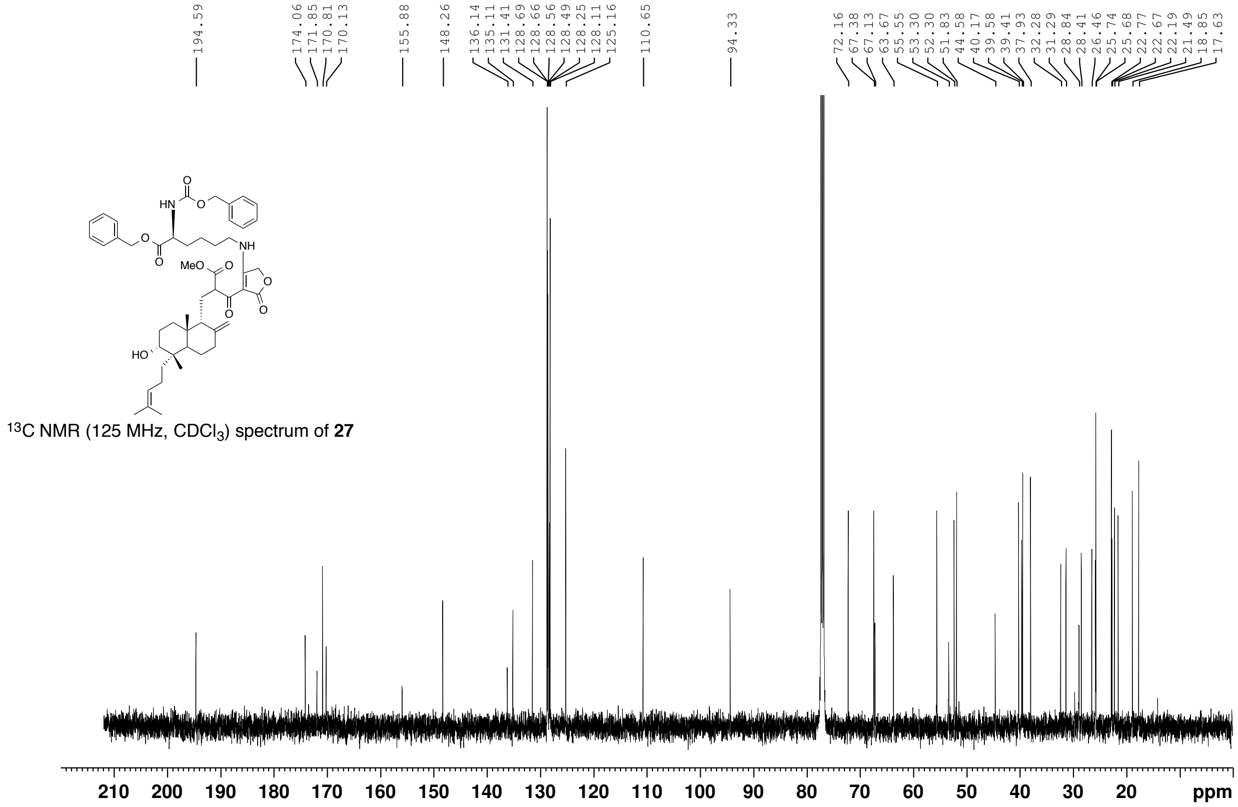

Supplementary Fig. 77. NMR spectra of 27 ($CDCl_3$)

Supplementary Table 1. Predicted functions of genes within the *dpas* cluster. The Gene size (base pairs) and protein size (amino acids) were predicted by 2ndFind (coding regions were not determined experimentally). The predicted function of each gene and amino acid identity/similarity, as compared with protein homologues found by protein BLAST search against NCBI database, are shown.

Gene	Base pairs	Amino acids	Protein homologue, origin (accession number)	Identity/similarity (%)	Predicted function
dpasA	6640	2159	SubA, *Metarhizium robertsii* ARSEF 23 (MAA_07496)	47/63	Non-reducing polyketide synthase (SAT-KS-AT-PT-ACP-MT)
dpasB	811	243	SubB, *Metarhizium robertsii* ARSEF 23 (MAA_07497)	62/74	Terpene cyclase (integral membrane protein)
dpasC	1149	336	SubC, *Metarhizium robertsii* ARSEF 23 (MAA_11696)	61/72	Prenyltransferase
dpasD	1186	332	SubD, *Metarhizium robertsii* ARSEF 23 (MAA_07498)	67/81	GGPP synthase
dpasE	1624	476	SubE, *Metarhizium robertsii* ARSEF 23 (MAA_07499)	51/69	Epoxidase (FAD-dependent monooxygenase)
dpasF	1422	473	Hypothetical protein, *Pseudomassariella vexata* (ORY60729)	37/58	Flavin-dependent oxidoreductase

Supplementary Table 2. Gene constitution of DDP clusters. The predicted function, NCBI accession number (if assigned), gene size (base pairs: bp), and protein size (amino acids: aa) of each gene are shown. The function was annotated based on the amino acid sequence homology (Supplementary Table 3) and functional domains in the amino acid sequence by Conserved Domain Search (NCBI). NR-PKS: non-reducing polyketide synthase, GGPPS: geranylgeranyl diphosphate synthase, PT: prenyltransferase, FMOep: FAD-dependent epoxidase, TC: terpene cyclase, BBE: BBE domain-containing flavin-dependent oxidoreductase, FMO: flavin-dependent monooxygenase, SDR: short-chain dehydrogenase/reductase, MT: methyltransferase, P450: cytochrome P450. Gene/protein sizes were originally re-predicted by 2ndFind (coding regions were not determined experimentally). The PCR primers used for amplification of each gene were designated using genomic sequence of *F. graminearum* PH-1 (*dpfg* genes), *M. phaseolina* MS6 (*dpmp* genes), *C. higginsianum* IMI349063 (*dpch* genes), *A. sacchari* Kumo-3 (*dpas* genes), and *M. anisopliae* E6 (*dpma* genes) as a reference.

Predicted function	Gene	*dpfg*	*dpmp*	*dpch*	*dpas*	*dpma*
NR-PKS (SubA homologue)	*dpxxA*	FGSG_04588<br>6590 bp/2172 aa	MPH_09202<br>6707 aa/2214 aa	CH63R_05473<br>6675 bp/2224 aa	(no accession)<br>6640 bp/2159 aa	MANI_006324<br>6612 aa/2181 aa
TC (SubB homologue)	*dpxxB*	FGSG_12222<br>797 bp/242 aa	MPH_09195<br>799 bp/243 aa	CH63R_05476<br>822 bp/242 aa	(no accession)<br>811 bp/243 aa	MANI_006367<br>799 bp/243 aa
PT (SubC homologue)	*dpxxC*	FGSG_04593<br>1067 bp/315 aa	(no accession)<br>1117 bp/345 aa	CH63R_05478<br>1163 bp/316 aa	(no accession)<br>1149 bp/336 aa	(no accession)<br>1098 bp/260 aa
GGPPs (SubD homologue)	*dpxxD*	FGSG_04591<br>1176 bp/325 aa	(no accession)<br>1145 bp/321 aa	CH63R_05480<br>1210 bp/322 aa	(no accession)<br>1186 bp/332 aa	MANI_006355<br>1190 bp/331 aa
FMOep (SubE homologue)	*dpxxE*	FGSG_04595<br>1563 bp/466 aa	MPH_09197<br>1593 bp/470 aa	CH63R_05475<br>1660 bp/470 aa	(no accession)<br>1624 bp/476 aa	(no accession)<br>1617 bp/479 aa
BBE (SubF homologue)	*dpxxF*					MANI_006343<br>1644 bp/509 aa
FMO	*dpxxF*			CH63R_05482<br>1422 bp/473 aa	(no accession)<br>1422 bp/473 aa	
SDR1	*dpxxG*	FGSG_12223<br>860 bp/270 aa	MPH_09198<br>864 bp/268 aa	CH63R_05477<br>923 bp/268 aa		
SDR2	*dpxxH*	FGSG_04592<br>1322 bp/349 aa	MPH_09200<br>1344 bp/366 aa	CH63R_05479<br>1385 bp/392 aa		
MT1	*dpxxI*	FGSG_04596<br>1458 bp/397 aa	MPH_09194<br>1443 bp/397 aa			
P450 (DpfgJ)<br>P450 (DpmpJ)	*dpxxJ*	FGSG_04590<br>1896 bp/516 aa	MPH_09193<br>1747 bp/546 aa			
MT2	*dpfgK*	FGSG_04589<br>897 bp/298 aa				

Supplementary Table 3. Comparative analyses of amino acid sequences encoded by each DDP biosynthetic gene. **a**, NR-PKS, **b**, GGPPS, **c**, PT, **d**, FMOep, **e**, TC, **f**, SDR1/2, **g**, MT1/2, **h**, P450, **i**, FMO. Identity/similarity (%) of the protein homologues, based on protein BLAST are shown. *MT1 is not similar to MT2, although they joined methyltransferase family. DpfgK (MT2) contains a region annotated as leucine carboxyl methyltransferase (LCM) domain (pfam04072), while DpfgI and DpmpI (MT1s) do not contain the region based on Conserved Domain Search (NCBI).

a. NR-PKS

	DpmpA	DpchA	DpasA	DpmaA
DpfgA	50/65	57/72	51/66	54/70
DpmpA		48/63	45/59	46/63
DpchA			50/65	49/64
DpasA				46/63

b. GGPPS

	DpmpD	DpchD	DpasD	DpmaD
DpfgD	76/89	83/93	66/80	77/89
DpmpD		75/88	66/81	68/85
DpchD			69/86	71/86
DpasD				69/84

c. PT

	DpmpC	DpchC	DpasC	DpmaC
DpfgC	58/71	73/81	59/73	62/70
DpmpC		58/73	53/64	51/63
DpchC			59/73	58/68
DpasC				52/65

d. FMOep

	DpmpE	DpchE	DpasE	DpmaE
DpfgE	55/70	64/81	51/69	59/75
DpmpE		52/70	47/65	52/68
DpchE			52/69	59/75
DpasE				49/66

e. TC

	DpmpB	DpchB	DpasB	DpmaB
DpfgB	73/84	78/86	62/77	77/85
DpmpB		67/82	61/75	71/82
DpchB			58/75	69/82
DpasB				61/73

f. SDR1/2

	DpmpG	DpchG	DpfgH	DpmpH	DpchH
DpfgG	61/75	63/77	25/45	22/42	24/42
DpmpG		60/73	22/39	21/37	23/43
DpchG			26/44	27/40	27/43
DpfgH				61/74	67/79
DpmpH					62/75

g. MT1/2

	DpmpI	DpfgK
DpfgI	62/78	*
DpmpI		*

h. P450

	DpmpJ
DpfgJ	21/34

i. FMO

	DpasF
DpchF	34/50

Supplementary Table 4. PCR primers used in this study.

Primer	Sequence (5' to 3')
pU-FW	TGCTTGGAGGATAGCAACCG
pU4-P1-NotI-FW	TTTGAGCTAGCGGCCAAGCTCCGGAATTCGAGCTC
PU4-T2-Asp718-RV	ACTACAGATCCCCGGGGTACGACTACCCGGGTCAC
sC-pU-IFSphI-FW	CCAGTGCCAAGCTTGGATCCTCTAGAGTCGATCTTGG
sC-pU-IFXbaI-RV	CGGCAGATCCTCTAGAGCTTCTCTTGGCAATAGCTGC
NadeA-pU-IFSphI-FW	CCAGTGCCAAGCTTGTCGCAGATAAACATGTTGTGG
NadeA-pU-IFXbaI-RV	CGGCAGATCCTCTAGGTGCGGAACTCCGAATCCTC
ptrA-pU-IFSphI-FW	CCAGTGCCAAGCTTGGGGCAATTGATTACGGGATC
ptrA-pU-IFXbaI-RV	CGGCAGATCCTCTAGAAAATGGGGTGACGATGAGC
dpasA-IFpUNotI-FW	TTTGAGCTAGCGGCCATCATGCCTGCGGAATCAAC
dpasA-IFpUNotI-RV	GTCACTAGTGCGGCCATGCGAGACTATGAGCAGTC
dpasA-R1	CTGTCGTGGGATTACTCCTG
dpasB-IFpUAsp718-FW	CCGAATTCGAGCTCGAACCATGGACGTCCACGACC
dpasB-IFpUAsp718-RV	ACTACAGATCCCCGGACTTTGGCTATAGATATGCG
dpasC-IFpUAsp718-FW	CCGAATTCGAGCTCGATGGCATCCGTAGCAAAAGC
dpasC-IFpUAsp718-RV	ACTACAGATCCCCGGATGCAGTTTGACAGAATACC
dpasD-IFpUNotI-FW	TTTGAGCTAGCGGCCATGACGAACAGCACATTACC
dpasD-IFpUNotI-RV	GTCACTAGTGCGGCCACAAGCTTTAGTAGACAGTC
dpasE-IFpUNotI-FW	TTTGAGCTAGCGGCCATCAATATGTCGCAACCAGC
dpasE-IFpUNotI-RV	GTCACTAGTGCGGCCCGCAGACTTAATCGTGACGG
dpasF-IFpUAsp718-FW	CCGAATTCGAGCTCGAAGATGAACAGACTCCTCGC
dpasF-IFpUAsp718-RV	ACTACAGATCCCCGGCGTCTCTTGAGTTGAATGTG
dpfgB-IFpUAsp718-FW	CCGGAATTCGAGCTCGAACATGGAAGTCGCCGACCC
dpfgB-IFpUAsp718-RV	ACTACAGATCCCCGGCACCACAGAACCAGTACAGC
dpfgE-IFpUNotI-FW	TTTGAGCTAGCGGCCCAAAGATGTCTCAAAAACCG
dpfgE-IFpUNotI-RV	GTCACTAGTGCGGCCGCAAAGACTGCCCATTAAGC
dpfgG-IFpUAsp718-FW	CCGGAATTCGAGCTCGACCATGTCGGTTCTCCAGAC
dpfgG-IFpUAsp718-RV	ACTACAGATCCCCGGTTGCAGGTGTCGGCAATCC
dpfgH-IFpUNotI-FW	TTTGAGCTAGCGGCCACCATGTCTCCATTATGGG
dpfgH-IFpUNotI-RV	GTCACTAGTGCGGCCATTACCAAACTTTATTGCAC
dpfgI-IFpUNotI-FW	TTTGAGCTAGCGGCCATCATGGCCCAGCAAGCCCC
dpfgI-IFpUNotI-RV	GTCACTAGTGCGGCCGTAAGATATTAACTACTTAG
dpfgJ-IFpUAsp718-FW	CCGGAATTCGAGCTCGAATATGGAGGACCTTCTTG
dpfgJ-IFpUAsp718-RV	ACTACAGATCCCCGGCTCTCTTCCTATATCCACCC
dpfgJ-R1	GCAATACGCACTACATGACC
dpfgK-IFpUNotI-FW	TTTGAGCTAGCGGCCAAAATGTCCGGCAGCACGC
dpfgK-IFpUNotI-RV	GTCACTAGTGCGGCCTGCCGTTACGCTTGCTAAGG
dpmpB-IFpUAsp718-FW	CCGGAATTCGAGCTCGAAAATGAATATTGTCCCCTTG
dpmpB-IFpUAsp718-RV	ACTACAGATCCCCGGAGGGCCTCTAGTTTACCTCC
dpmpE-IFpUNotI-FW	TTTGAGCTAGCGGCCAAGATGGAATCGTCATCGTCC
dpmpE-IFpUNotI-RV	GTCACTAGTGCGGCCCATTGGAGAGGTGAATCTGC
dpmpG-IFpUAsp718-FW	CCGGAATTCGAGCTCGAAAATGTCCGCTTCCAGCAC
dpmpG-IFpUAsp718-RV	ACTACAGATCCCCGGGCTGACTAGCGTTTCTACAG
dpmpH-IFpUNotI-FW	TTTGAGCTAGCGGCCATGATGCTGCAGCCAATCCG
dpmpH-IFpUNotI-RV	GTCACTAGTGCGGCCGAAAATGAGCATCTACGCCC
dpmpI-IFpUAsp718-FW	CCGGAATTCGAGCTCGAATATGAGCCAGAAAAATAC
dpmpI-IFpUAsp718-RV	ACTACAGATCCCCGGATCAAAAAAGCTCATGAATC
dpmpJ-IFpUNotI-FW	TTTGAGCTAGCGGCCAATATGCTACAGCTTATCC
dpmpJ-IFpUNotI-RV	GTCACTAGTGCGGCCAAGGTCGGCTTTCATTAGAG
dpchB-IFpUAsp718-FW	CCGGAATTCGAGCTCGATCATGAACGTCGCCGACATC
dpchB-IFpUAsp718-RV	ACTACAGATCCCCGGGCTCACTTCTTCAACTTTCC
dpchE-IFpUNotI-FW	TTTGAGCTAGCGGCCAGCATGTCAGAACCCCATTTC
dpchE-IFpUNotI-RV	GTCACTAGTGCGGCCATTCGGTTACATCGGCAACG
dpchF-IFpUNotI-FW	TTTGAGCTAGCGGCCACCATGAAGCTTTCCTTCATCG
dpchF-IFpUNotI-RV	GTCACTAGTGCGGCCAACAGCTCTACGCCACGACC
dpchG-IFpUAsp718-FW	CCGGAATTCGAGCTCGACAATGACTTCCTCGGAAAAG
dpchG-IFpUAsp718-RV	ACTACAGATCCCCGGAGGTCACTTGTTTTGGTGGC
dpchH-IFpUNotI-FW	TTTGAGCTAGCGGCCAGCATGTTGTCAGCGTGGGC
dpchH-IFpUNotI-RV	GTCACTAGTGCGGCCATACCTTCTACCCAATGTGG
dpmaF-IFpUAsp718-FW	CCGGAATTCGAGCTCGAAGATGACACGCCTCTCAC
dpmaF-IFpUAsp718-RV	ACTACAGATCCCCGGTAACAATACATTAGAGTACC

Supplementary Table 5. Expression plasmids constructed in this study.

Plasmid	Insert (restriction site)	Forward primer	Reverse primer	PCR Template
pUPTRA2-*dpasCD*	*dpasC* (*Asp*718)	dpasC-IFpUAsp718-FW	dpasC-IFpUAsp718-RV	Genomic DNA of *A. sacchari* Kumo-3
	dpasD (*Not*I)	dpasD-IFpUNotI-FW	dpasD-IFpUNotI-RV	Genomic DNA of *A. sacchari* Kumo-3
pUARA2-*dpasACD*	*dpasC+D* (*Asp*718)	dpasC-IFpUAsp718-FW	PU4-T2-Asp718-RV	pUPTRA2-*dpasCD*
	dpasA (*Not*I)	dpasA-IFpUNotI-FW	dpasA-IFpUNotI-RV	Genomic DNA of *A. sacchari* Kumo-3
pUADEA2-*dpasBE*	*dpasB* (*Asp*718)	dpasB-IFpUAsp718-FW	dpasB-IFpUAsp718-RV	Genomic DNA of *A. sacchari* Kumo-3
	dpasE (*Not*I)	dpasE-IFpUNotI-FW	dpasE-IFpUNotI-RV	Genomic DNA of *A. sacchari* Kumo-3
pUADEA2-*dpfgBE*	*dpfgB* (*Asp*718)	dpfgB-IFpUAsp718-FW	dpfgB-IFpUAsp718-RV	Genomic DNA of *F. graminearum* 50218
	dpfgE (*Not*I)	dpfgE-IFpUNotI-FW	dpfgE-IFpUNotI-RV	Genomic DNA of *F. graminearum* 50218
pUADEA2-*dpmpBE*	*dpmpB* (*Asp*718)	dpmpB-IFpUAsp718-FW	dpmpB-IFpUAsp718-RV	Genomic DNA of *M. phaseolina* NBRC 7317
	*dpmpE (Not*I)	dpmpE-IFpUNotI-FW	dpmpE-IFpUNotI-RV	Genomic DNA of *M. phaseolina* NBRC 7317
pUADEA2-*dpchBE*	*dpchB* (*Asp*718)	dpchB-IFpUAsp718-FW	dpchB-IFpUAsp718-RV	Genomic DNA of *C. higginsianum* MAFF 305635
	dpchE (*Not*I)	dpchE-IFpUNotI-FW	dpchE-IFpUNotI-RV	Genomic DNA of *C. higginsianum* MAFF 305635
pUSCA2-*dpasF*	*dpasF* (*Asp*718)	dpasF-IFpUAsp718-FW	dpasF-IFpUAsp718-RV	Genomic DNA of *A. sacchari* Kumo-3
pUPTRA2-*dpmaF*	*dpmaF* (*Asp*718)	dpmaF-IFpUAsp718-FW	dpmaF-IFpUAsp718-RV	Genomic DNA of *M. anisopliae* NBRC 103233
pUSCA2-*dpmaF*	*dpmaF* (*Asp*718)	dpmaF-IFpUAsp718-FW	dpmaF-IFpUAsp718-RV	Genomic DNA of *M. anisopliae* NBRC 103233
pUPTRA2-*dpmpG*	*dpmpG* (*Asp*718)	dpfgG-IFpUAsp718-FW	dpfgG-IFpUAsp718-RV	Genomic DNA of *M. phaseolina* NBRC 7317
pUPTRA2-*dpmpGH*	*dpmpG* (*Asp*718)	dpmpG-IFpUAsp718-FW	dpmpG-IFpUAsp718-RV	Genomic DNA of *M. phaseolina* NBRC 7317
	dpmpH (*Not*I)	dpmpH-IFpUNotI-FW	dpmpH-IFpUNotI-RV	Genomic DNA of *M. phaseolina* NBRC 7317
pUPTRA2-*dpfgGH*	*dpfgG* (*Asp*718)	dpfgG-IFpUAsp718-FW	dpfgG-IFpUAsp718-RV	Genomic DNA of *F. graminearum* 50218
	dpfgH (*Not*I)	dpfgH-IFpUNotI-FW	dpfgH-IFpUNotI-RV	Genomic DNA of *F. graminearum* 50218
pUPTRA2-*dpchGH*	*dpchG* (*Asp*718)	dpchG-IFpUAsp718-FW	dpchG-IFpUAsp718-RV	Genomic DNA of *C. higginsianum* MAFF 305635
	dpchH (*Not*I)	dpchH-IFpUNotI-FW	dpchH-IFpUNotI-RV	Genomic DNA of *C. higginsianum* MAFF 305635
pUSCA2-*dpchF*	*dpchF* (*Not*I)	dpchF-IFpUNoTI-FW	dpchF-IFpUNoTI-RV	Genomic DNA of *C. higginsianum* MAFF 305635
pUSCA2-*dpfgI*	*dpfgI* (*Not*I)	dpfgI-IFpUNotI-FW	dpfgI-IFpUNotI-RV	Genomic DNA of *F. graminearum* 50218
pUSCA2-*dpfgIJ*	*dpfgJ* (*Asp*718)	dpfgJ-IFpUAsp718-FW	dpfgJ-IFpUAsp718-RV	Genomic DNA of *F. graminearum* 50218
	dpfgI (*Not*I)	dpfgI-IFpUNotI-FW	dpfgI-IFpUNotI-RV	Genomic DNA of *F. graminearum* 50218
pUSCA2-*dpfgIJK*	*dpfgJ+I* (*Asp*718)	dpfgJ-IFpUAsp718-FW	PU4-T2-Asp718-RV	pUSCA2-*dpfgIJ*
	dpfgK (*Not*I)	dpfgK-IFpUNotI-FW	dpfgK-IFpUNotI-RV	Genomic DNA of *F. graminearum* 50218
pUSCA2-*dpmpI*	*dpmpI* (*Asp*718)	dpmpI-IFpUAsp718-FW	dpmpI-IFpUAsp718-RV	Genomic DNA of *M. phaseolina* NBRC 7317
pUSCA2-*dpmpIJ*	*dpmpI* (*Asp*718)	dpmpI-IFpUAsp718-FW	dpmpI-IFpUAsp718-RV	Genomic DNA of *M. phaseolina* NBRC 7317
	dpmpJ (*Not*I)	dpmpJ-IFpUNotI-FW	dpmpJ-IFpUNotI-RV	Genomic DNA of *M. phaseolina* NBRC 7317
pUSCA2-*dpfgK-dpasF*	*dpasF* (*Asp*718)	dpasF-IFpUAsp718-FW	dpasF-IFpUAsp718-RV	Genomic DNA of *A. sacchari* Kumo-3
	dpfgK (*Not*I)	dpfgK-IFpUNotI-FW	dpfgK-IFpUNotI-RV	Genomic DNA of *F. graminearum* 50218
pUSCA2-*dpfgIJK-dpasF*	*dpfgJ+I* (*Asp*718)	dpfgJ-IFpUAsp718-FW	PU4-T2-Asp718-RV	pUSCA2-*dpfgIJ*
	dpasF+dpfgK (*Not*I)	pU4-P1-NotI-FW	dpfgK-IFpUNotI-RV	pUSCA2-*dpfgK-dpasF*
pUSCA2-*dpmpIJ-dpasF*	*dpmpI+J* (*Asp*718)	dpmpI-IFpUAsp718-FW	PU4-T2-Asp718-RV	pUSCA2-*dpmpIJ*
	dpasF+dpfgK (*Not*I)[a]	pU4-P1-NotI-FW	dpfgK-IFpUNotI-RV	pUSCA2-*dpfgK-dpasF*
pUSCA2-*dpmpI-dpchF*	*dpmpI* (*Asp*718)	dpmpI-IFpUAsp718-FW	dpmpI-IFpUAsp718-RV	Genomic DNA of *M. phaseolina* NBRC 7317
	dpchF (*Not*I)	dpchF-IFpUNoTI-FW	dpchF-IFpUNoTI-RV	Genomic DNA of *C. higginsianum* MAFF 305635
pUPTRA2-*dpmpI*	*dpmpI* (*Asp*718)	dpmpI-IFpUAsp718-FW	dpmpI-IFpUAsp718-RV	Genomic DNA of *M. phaseolina* NBRC 7317

[a]Consideration of their function, *dpfgK* was co-expressed with *dpfgJ*.

Supplementary Table 6. Nutrients and chemicals supplemented for selection of *A. oryzae* transformants.

Original strain	Selectable marker	Resulting transformant	Nutrients (concentration)
niaD$^-$, *sC*$^-$, *adeA*$^-$, Δ*argB*	*argB*	*niaD*$^-$, *sC*$^-$, *adeA*$^-$	adenine (0.01%), L-methionine (0.15%), ammonium sulfate (0.05%)
niaD$^-$, *sC*$^-$, *adeA*$^-$	*adeA*	*niaD*$^-$, *sC*$^-$	L-methionine (0.15%), ammonium sulfate (0.05%)
niaD$^-$, *sC*$^-$	*sC*	*niaD*$^-$	ammonium sulfate (0.05%)
	ptrA	*niaD*$^-$, *sC*$^-$, *ptrA*$^+$	L-methionine (0.15%), ammonium sulfate (0.05%), pyrithiamine (0.1 μg/mL)[a]
niaD$^-$	*ptrA*	*niaD*$^-$, *ptrA*$^+$	ammonium sulfate (0.05%), pyrithiamine (0.1 μg/mL)[a]
niaD$^-$, *sC*$^-$, *ptrA*$^+$	*sC*	*niaD*$^-$, *ptrA*$^+$	ammonium sulfate (0.05%), pyrithiamine (0.1 μg/mL)[a]

[a]Pyrithiamine hydrobromide (Sigma-Aldrich)

Supplementary Table 7. ^{13}C (125 MHz) and ^{1}H HMR (500 MHz) data for **4**.[a,b]

Position	^{13}C	^{1}H (multi, *J* in Hz)	
1	22.7	1.55	(1H, m, Hα)
		1.37	(1H, qd, 13.0, 4.5, Hβ)
2	30.9	2.40	(1H, brtd, 13.8, 5.4, Hα)
		2.16	(1H, m, Hβ)
3	149.1		
4	55.9	2.01	(1H, dd, 10.6, 4.1)
5	37.6		
6	34.0	1.84	(1H, m, Hα)
		1.28	(1H, m, Hβ)
7	27.3	1.70	(2H, m)
8	73.6	3.55	(1H, dd, 9.4, 6.3)
9	40.9		
10	38.9	1.65	(1H, m)
11	37.4	1.52	(1H, m, Ha)
		1.31	(1H, m, Hb)
12	21.6	1.97	(2H, m)
13	124.8	5.12	(1H, brt, 7.0)
14	131.1		
15	25.6	1.69	(3H, s)
16	17.4	1.63	(3H, s)
17	16.9	0.79	(3H, s)
18	22.7	0.94	(3H, s)
19	110.1	4.58	(1H, brs, Ha)
		4.37	(1H, brs, Hb)
20	21.9	2.67	(1H, dd, 13.7, 4.1, Ha)
		2.59	(1H, dd, 13.7, 10.6, Hb)
21	103.2		
22	165.1		
23	106.6		
24	155.3		
25	165.9		
26	17.1	2.19	(3H, s)
27	9.9	1.91	(3H, s)

[a] Assignment for all compounds were based on COSY, HSQC, HMBC experiments.

[b] in $CDCl_3$ (10% CD_3OD)

Supplementary Table 8. ^{1}H NMR (500 MHz) data for **15b** and **15c**, and Δδ(**15b**–**15c**) values.[a,b]

	(*S*)-MTPA ester of **15** (**15b**)		(*R*)-MTPA ester of **15** (**15c**)		$\Delta\delta_{H(S-R)}$
Position	^{1}H (multi, J in Hz)		^{1}H (multi, J in Hz)		
1	1.53	(1H, m, Hα)	1.51	(1H, m, Hα)	+0.02
	1.37	(1H, m, Hβ)	1.36	(1H, qd, 12.8, 4.5 Hβ)	+0.01
2	2.37	(1H, br td, 13.2, 5.5, Hα)	2.36	(1H, br td, 13.7, 5.4)	+0.01
	2.11	(1H, br d, 13.5, Hβ)	2.10	(1H, br d, 14.0, Hβ)	+0.01
4	1.99	(1H, m)	2.00	(1H, m)	−0.01
6	1.98	(1H, m, Hα)	1.99	(1H, m, Hα)	−0.01
	1.40	(1H, br d, 13.5, Hβ)	1.43	(1H, br d, 13.5, Hβ)	−0.03
7	1.75-1.89	(2H, m)	1.85-1.97	(2H, m)	−0.04
8	5.06	(1H, dd, 11.4, 4.5)	5.08	(1H, dd, 10.1, 5.6)	−0.02
10	1.77	(1H, m)	1.77	(1H, dd, 12.6, 2.8)	±0.00
11	1.36	(1H, m, Ha)	1.24	(1H, m, Ha)	+0.12
	1.18	(1H, m, Hb)	1.03	(1H, m, Hb)	+0.15
12	2.02	(1H, m, Ha)	1.98	(1H, m, Ha)	+0.04
	1.95	(1H, m, Hb)	1.90	(1H, m, Hb)	+0.05
13	5.05	(1H, br m)	4.99	(1H, br t, 7.0)	+0.06
15	1.70	(3H, s)	1.70	(3H, s)	±0.00
16	1.61	(3H, s)	1.64	(3H, s)	−0.03
17	0.83	(3H, s)	0.80	(3H, s)	+0.03
18	0.95	(3H, s)	0.97	(3H, s)	−0.02
19	4.52	(1H, br s, Ha)	4.52	(1H, br s, Ha)	±0.00
	4.21	(1H, br s, Hb)	4.21	(1H, br s, Hb)	±0.00
20	2.68	(1H, dd, 12.8, 3.8, Ha)	2.69	(1H, dd, 12.8, 3.7, Ha)	−0.01
	2.44	(1H, t, 12.2, Hb)	2.43	(1H, t, 12.3, Hb)	+0.01
26	2.24	(3H, s)	2.24	(3H, s)	±0.00
27	1.90	(3H, s)	1.90	(3H, s)	±0.00
OMe	3.85	(3H, s)	3.85	(3H, s)	±0.00

[a] Assignment for all compounds were based on COSY, HSQC, HMBC experiments.
[b] in $CDCl_3$

Supplementary Table 9. ^{13}C (125 MHz) and ^{1}H HMR (500 MHz) data for **5**-**7**.[a]

	**5** subglutinol A[b]		**6** subglutinol B[b]		**7**[c]	
Position	^{13}C	^{1}H (multi, *J* in Hz)	^{13}C	^{1}H (multi, *J* in Hz)	^{13}C	^{1}H (multi, *J* in Hz)
1	25.0	1.47 (1H, m, Hα)	24.9	1.48 (1H, m, Hα)	23.4	1.70 (1H, m, Hα)
		1.62 (1H, m, Hβ)		1.63 (1H, m, Hβ)		1.42 (1H, brqd, 13.0, 4.5, Hβ)
2	30.8	2.45 (1H, m, Hα)	30.8	2.45 (1H, m, Hα)	30.8	2.35 (1H, m, Hα)
		2.22 (1H, m, Hβ)		2.24 (1H, m, Hβ)		2.17 (1H, m, Hβ)
3	149.3		149.4		148.0	
4	56.9	1.98 (1H, dd, 10.0, 3.6)	57.1	1.95 (1H, m)	54.8	2.15 (1H, m)
5	38.7		38.7		37.7	
6	34.7	1.94 (1H, m, Hα)	34.7	1.95 (1H, m, Hα)	33.9	1.78 (1H, m, Hα)
		1.35 (1H, brd, 13.5, Hβ)		1.34 (1H, brd, 13.5, Hβ)		1.31 (1H, brd, 12.8, Hβ)
7	22.3	1.86 (1H, m, Hα)	22.5	1.89 (1H, m, Hα)	27.2	1.70 (2H, m)
		1.75 (1H, m, Hβ)		1.67 (1H, m, Hβ)		
8	87.2	3.12 (1H, dd, 12.1, 3.3)	85.9	3.29 (1H, brdd, 12.1, 2.2)	75.6	3.57 (1H, brt, 7.7)
9	44.4		44.9		44.1	
10	45.3	1.59 (1H, m)	44.9	1.57 (1H, m)	40.4	1.78 (1H, m)
11	47.8	1.82 (1H, m, Hα)	48.4	1.26 (1H, brt, 9.0, Hα)	51.6	2.82 (1H, d, 14.0, Ha)
		1.44 (1H, dd, 11.4, 3.5, Hβ)		1.94 (1H, m, Hβ)		2.34 (1H, d, 14.0, Hb)
12	74.2	4.68 (1H, m)	73.8	4.82 (1H, dd, 16.3, 9.0)	204.2	
13	127.2	5.35 (1H, brd, 8.7)	126.9	5.32 (1H, d, 9.0)	126.0	6.25 (1H, brs)
14	135.5		134.7		156.9	
15	25.7	1.71 (3H, s)	25.9	1.73 (3H, s)	27.8	1.94 (3H, s)
16	18.1	1.69 (3H, s)	18.0	1.68 (3H, s)	20.8	2.15 (3H, s)
17	16.9	0.86 (3H, s)	15.6	0.88 (3H, s)	15.7	0.86 (3H, s)
18	24.4	0.95 (3H, s)	24.2	0.95 (3H, s)	22.8	0.95 (3H, s)
19	111.0	4.66 (1H, brs, Ha)	111.2	4.68 (1H, brs, Ha)	110.6	4.58 (1H, brs, Ha)
		4.48 (1H, brs, Hb)		4.50 (1H, brs, Hb)		4.33 (1H, brs, Hb)
20	22.6	2.79 (1H, dd, 14.3, 3.6, Ha)	22.8	2.80 (1H, dd, 14.3, 2.8, Ha)	21.6	2.61 (1H, dd, 13.6, 4.2, Ha)
		2.55 (1H, dd, 14.3, 10.0, Hb)		2.52 (1H, dd, 14.3, 10.1, Hb)		2.52 (1H, dd, 13.6, 11.2, Hb)
21	103.1		103.0		102.2	
22	164.7		164.4		165.1	
23	106.4		106.1		107.0	
24	155.7		155.8		155.1	
25	165.3		165.0		165.7	
26	17.2	2.20 (3H, s)	17.3	2.20 (3H, s)	17.1	2.18 (3H, s)
27	10.0	1.92 (3H, s)	9.9	1.92 (3H, s)	10.0	1.90 (3H, s)

[a] Assignment for all compounds were based on COSY, HSQC, HMBC experiments.

[b] in $CDCl_3$

[c] in $CDCl_3$ (10% CD_3OD)

Supplementary Table 10. ^{13}C (125 MHz) and ^{1}H HMR (500 MHz) data for **8** and **9**.[a]

Position	**8**[b]			**9** higginsianin B[c]		
	^{13}C	^{1}H (multi, *J* in Hz)		^{13}C	^{1}H (multi, *J* in Hz)	
1	24.2	1.60	(1H, m, Hα)	22.5	1.59	(1H, m, Hα)
		1.48	(1H, qd, 12.9, 4.5, Hβ)		1.30	(1H, m, Hβ)
2	30.9	2.43	(1H, m, Hα)	31.8	2.17	(2H, m)
		2.28	(1H, m, Hβ)			
3	148.6			148.8		
4	55.7	2.10	(1H, dd, 10.6, 3.5)	55.4	2.18	(1H, m)
5	37.2			37.9		
6	33.8	2.22	(1H, m, Hα)	28.2	2.17	(1H, m, Hα)
		1.59	(1H, m, Hβ)		0.98	(1H, m, Hβ)
7	35.8	2.43	(1H, ddd, 16.7, 5.5, 3.4, Hα)	25.6	1.60	(1H, m, Hα)
		2.60	(1H, ddd, 16.7, 12.9, 6.3, Hβ)		1.95	(1H, m, Hβ)
8	216.6			72.3	3.67	(1H, brs)
9	50.8			39.1		
10	41.4	2.15	(1H, dd, 12.6, 3.2)	40.1	1.78	(1H, dd, 12.7, 2.8)
11	39.0	1.77	(1H, ddd, 13.7, 11.3, 5.1, Ha)	39.6	1.37	(2H, m)
		1.35	(1H, ddd, 13.7, 10.8, 5.4, Hb)			
12	23.8	1.90	(2H, m)	21.4	2.11	(1H, m, Ha)
					1.93	(1H, m, Hb)
13	124.5	5.08	(1H, brt, 7.1)	125.2	5.14	(1H, brt, 7.1)
14	131.8			131.2		
15	25.7	1.67	(3H, s)	25.6	1.69	(3H, s)
16	17.7	1.61	(3H, s)	17.5	1.63	(3H, s)
17	22.4	1.01	(3H, s)	18.8	0.84	(3H, s)
18	21.4	1.05	(3H, s)	22.6	0.97	(3H, s)
19	111.7	4.72	(1H, brs, Ha)	110.2	4.57	(1H, brs, Ha)
		4.52	(1H, brs, Hb)		4.44	(1H, brs, Hb)
20	22.3	2.77	(1H, dd, 14.0, 3.5, Ha)	22.2	2.96	(1H, dd, 14.2, 5.5, Ha)
		2.54	(1H, dd, 14.0, 10.6, Hb)		2.60	(1H, dd, 14.2, 9.9, Hb)
21	102.8			102.7		
22	164.3			165.0		
23	105.9			107.1		
24	155.9			155.1		
25	165.0			165.8		
26	17.3	2.20	(3H, s)	17.1	2.18	(3H, s)
27	9.9	1.93	(3H, s)	10.0	1.90	(3H, s)

[a] Assignment for all compounds were based on COSY, HSQC, HMBC experiments.
[b] in $CDCl_3$
[c] in $CDCl_3$ (10% CD_3OD)

Supplementary Table 11. ^{13}C (125 MHz) and ^{1}H HMR (500 MHz) data for higginsianin A (**10**).[a,b]

Position	^{13}C	^{1}H (multi, *J* in Hz)
1	24.5	1.57 (1H, m, Hα)
		1.42 (1H, qd, 13.1, 4.4, Hβ)
2	31.9	2.54 (1H, brtd, 13.6, 5.4, Hα)
		2.28 (1H, brd, 13.6, Hβ)
3	148.5	
4	56.2	1.91 (1H, m)
5	37.6	
6	29.4	2.19 (1H, m, Hα)
		0.99 (1H, m, Hβ)
7	22.7	1.91 (1H, m, Hα)
		1.81 (1H, m, Hβ)
8	82.9	3.72 (1H, brs)
9	44.1	
10	38.5	1.70 (1H, m)
11	48.9	2.19 (1H, m, Hα)
		1.27 (1H, m, Hβ)
12	72.9	4.81 (1H, td, 8.8, 6.8)
13	127.3	5.24 (1H, brd, 8.8)
14	134.6	
15	25.9	1.72 (3H, s)
16	18.2	1.71 (3H, s)
17	20.7	0.97 (3H, s)
18	21.4	0.95 (3H, s)
19	111.2	4.65 (1H, brs, Ha)
		4.39 (1H, brs, Hb)
20	21.6	2.73 (1H, dd, 14.3, 4.0, Ha)
		2.67 (1H, dd, 14.3, 11.4, Hb)
21	102.8	
22	163.5	
23	105.7	
24	155.8	
25	165.0	
26	17.3	2.20 (3H, s)
27	9.8	1.92 (3H, s)

[a] Assignment for all compounds were based on COSY, HSQC, HMBC experiments.

[b] in $CDCl_3$

Supplementary Table 12. ^{13}C (125 MHz) and ^{1}H HMR (500 MHz) data for **11** and **12**.[a,b]

	**11**		**12**	
Position	^{13}C	^{1}H (multi, *J* in Hz)	^{13}C	^{1}H (multi, *J* in Hz)
1	22.7	1.61 (1H, m, Hα)	22.7	1.60 (1H, m, Hα)
		1.33 (1H, m, Hβ)		1.33 (1H, m, Hβ)
2	31.4	2.38 (1H, brtd, 13.6, 5.7, Hα)	31.3	2.36 (1H, brtd, 13.6, 5.6, Hα)
		2.11 (1H, m, Hβ)		2.11 (1H, m, Hβ)
3	149.3		149.2	
4	56.0	1.95 (1H, m)	56.0	1.92 (1H, m)
5	37.8		37.8	
6	28.4	2.28 (1H, m, Hα)	28.4	2.27 (1H, brtd, 13.7, 3.1, Hα)
		1.06 (1H, brd, 13.3, Hβ)		1.04 (1H, brd, 13.3, Hβ)
7	25.8	1.64 (1H, m, Hα)	25.8	1.64 (1H, m, Hα)
		1.97 (1H, m, Hβ)		1.97 (1H, m, Hβ)
8	72.3	3.63 (1H, brs)	72.3	3.64 (1H, brs)
9	39.4		39.4	
10	39.9	1.82 (1H, dd, 12.8, 3.0)	40.0	1.82 (1H, brdd, 12.8, 2.7)
11	39.8	1.39 (2H, m)	39.8	1.39 (2H, brt, 8.5)
12	21.6	2.11 (1H, m, Ha)	21.6	2.09 (1H, m, Ha)
		1.95 (1H, m, Hb)		1.94 (1H, m, Hb)
13	125.3	5.15 (1H, brt, 7.2)	125.3	5.15 (1H, brt, 6.9)
14	131.3		131.4	
15	25.7	1.69 (3H, s)	25.7	1.69 (3H, s)
16	17.6	1.63 (3H, s)	17.6	1.63 (3H, s)
17	19.0	0.84 (3H, s)	19.0	0.84 (3H, s)
18	22.7	0.96 (3H, s)	22.7	0.96 (3H, s)
19	108.9	4.50 (1H, brs, Ha)	109.0	4.49 (1H, brs, Ha)
		4.19 (1H, brs, Hb)		4.18 (1H, brs, Hb)
20	20.2	2.79 (1H, dd, 13.0, 3.9, Ha)	20.2	2.77 (1H, dd, 12.8, 3.7, Ha)
		2.47 (1H, dd, 13.0, 11.4, Hb)		2.48 (1H, dd, 12.8, 11.5, Hb)
21	103.6		104.3	
22	180.4		180.5	
23	118.6		120.0	
24	154.9		155.0	
25	162.9		163.2	
26	17.0	2.24 (3H, s)	59.6	4.57 (1H, d, 13.5, Ha)
				4.53 (1H, d, 13.5, Hb)
27	10.0	1.90 (3H, s)	9.5	1.95 (3H, s)
OMe	55.4	3.84 (3H, s)	55.5	3.90 (3H, s)

[a] Assignment for all compounds were based on COSY, HSQC, HMBC experiments.
[b] in $CDCl_3$

Supplementary Table 13. ^{13}C (125 MHz) and ^{1}H (500 MHz) NMR data for **13** and **14**.[a,b]

	**13**		**14**	
Position	^{13}C	^{1}H (multi, *J* in Hz)	^{13}C	^{1}H (multi, *J* in Hz)
1	22.7	1.61 (1H, m, Hα)	22.7	1.59 (1H, m, Hα)
		1.33 (1H, m, Hβ)		1.33 (1H, m, Hβ)
2	31.3	2.33 (1H, m, Hα)	31.4	2.33 (1H, m, Hα)
		2.12 (1H, m, Hβ)		2.12 (1H, m, Hβ)
3	148.8		148.9	
4	56.2	1.95 (1H, m)	55.7	1.95 (1H, m)
5	37.9		37.8	
6	28.4	2.25 (1H, brtd, 13.5, 3.5, Hα)	28.3	2.26 (1H, brtd, 13.7, 3.2, Hα)
		1.05 (1H, brd, 13.3, Hβ)		1.05 (1H, brd, 13.3, Hβ)
7	25.8	1.64 (1H, m, Hα)	25.8	1.62 (1H, m, Hα)
		1.96 (1H, m, Hβ)		1.98 (1H, m, Hβ)
8	72.2	3.64 (1H, brs)	72.2	3.63 (1H, brs)
9	39.4		39.4	
10	40.0	1.78 (1H, brdd, 12.7, 2.9)	40.0	1.78 (1H, dd, 12.7, 3.0)
11	39.7	1.38 (2H, m)	39.8	1.38 (2H, m)
12	21.6	2.09 (1H, m, Ha)	21.6	2.08 (1H, m, Ha)
		1.95 (1H, m, Hb)		1.93 (1H, m, Hb)
13	125.2	5.14 (1H, brt, 7.2)	125.3	5.24 (1H, brt, 7.2)
14	131.4		131.4	
15	25.7	1.69 (3H, s)	25.7	1.69 (3H, s)
16	17.6	1.63 (3H, s)	17.6	1.63 (3H, s)
17	19.0	0.84 (3H, s)	19.0	0.84 (3H, s)
18	22.7	0.96 (3H, s)	22.7	0.95 (3H, s)
19	109.5	4.53 (1H, brs, Ha)	109.4	4.53 (1H, brs, Ha)
		4.23 (1H, brs, Hb)		4.24 (1H, brs, Hb)
20	20.2	2.78 (1H, dd, 12.6, 3.6, Ha)	20.0	2.77 (1H, dd, 12.9, 3.6, Ha)
		2.49 (1H, t, 12.6, Hb)		2.47 (1H, t, 12.9, Hb)
21	106.8		105.6	
22	176.2		176.6	
23	119.1		119.8	
24	162.7		160.4	
25	162.9		162.9	
26	61.1	4.48 (2H, brs)	18.0	2.33 (3H, s)
27	166.4		165.7	
OMe	55.6	3.92 (3H, s)	56.0	3.87 (3H, s)
CO_2Me	53.1	3.92 (3H, s)	52.6	3.88 (3H, s)

[a] Assignment for all compounds were based on COSY, HSQC, HMBC experiments.
[b] in $CDCl_3$

Supplementary Table 14. ^{13}C (125 MHz) and ^{1}H HMR (500 MHz) data for **15** and **16**.[a,b]

	**15**		**16**	
Position	^{13}C	^{1}H (multi, *J* in Hz)	^{13}C	^{1}H (multi, *J* in Hz)
1	22.8	1.55 (1H, m, Hα)	22.8	1.55 (1H, m, Hα)
		1.37 (1H, m, Hβ)		1.37 (1H, m, Hβ)
2	30.7	2.36 (1H, brtd, 13.5, 5.6, Hα)	30.8	2.34 (1H, brtd, 13.5, 5.5, Hα)
		2.10 (1H, brd, 13.5, Hβ)		2.10 (1H, brd, 13.5, Hβ)
3	148.8		148.7	
4	55.7	1.93 (1H, m)	55.7	1.92 (1H, m)
5	37.6		37.6	
6	34.1	1.90 (1H, m, Hα)	34.2	1.87 (1H, m, Hα)
		1.36 (1H, m, Hβ)		1.35 (1H, m, Hβ)
7	27.7	1.72 (2H, m)	27.7	1.72 (2H, m)
8	73.9	3.56 (1H, brt, 7.7)	74.0	3.56 (1H, brt, 7.9)
9	41.0		41.1	
10	38.9	1.65 (1H, m)	39.0	1.64 (1H, m)
11	37.5	1.53 (1H, m, Ha)	37.5	1.53 (1H, m, Ha)
		1.32 (1H, m, Hb)		1.32 (1H, m, Hb)
12	21.8	1.97 (2H, m)	21.8	1.98 (2H, m)
13	125.0	5.14 (1H, brt, 7.1)	124.9	5.14 (1H, brt, 7.1)
14	131.1		131.2	
15	25.7	1.69 (3H, s)	25.7	1.70 (3H, s)
16	17.6	1.64 (3H, s)	17.6	1.64 (3H, s)
17	17.0	0.79 (3H, s)	17.0	0.79 (3H, s)
18	22.9	0.94 (3H, s)	22.9	0.93 (3H, s)
19	109.2	4.51 (1H, brs, Ha)	109.4	4.51 (1H, brs, Ha)
		4.19 (1H, brs, Hb)		4.18 (1H, brs, Hb)
20	19.9	2.69 (1H, dd, 12.8, 3.5, Ha)	19.9	2.69 (1H, dd, 12.9, 3.5, Ha)
		2.41 (1H, t, 12.8. Hb)		2.43 (1H, t, 12.9, Hb)
21	103.3		104.1	
22	180.3		180.5	
23	118.6		120.0	
24	154.9		155.1	
25	162.9		163.2	
26	16.9	2.24 (3H, s)	59.5	4.58 (1H, d, 13.5, Ha)
				4.54 (1H, d, 13.5, Hb)
27	10.0	1.90 (3H, s)	9.5	1.96 (3H, s)
OMe	55.3	3.84 (3H, s)	55.5	3.90 (3H, s)

[a] Assignment for all compounds were based on COSY, HSQC, HMBC experiments.

[b] in $CDCl_3$

Supplementary Table 15. ^{13}C (125 MHz) and ^{1}H HMR (500 MHz) data for **17–19**.[a,b]

	**17**		**18**		**19**	
Position	^{13}C	^{1}H (multi, *J* in Hz)	^{13}C	^{1}H (multi, *J* in Hz)	^{13}C	^{1}H (multi, *J* in Hz)
1	25.1	1.43 (1H, m, Hα)	25.0	1.45 (1H, m, Hα)	23.7	1.72 (1H, m, Hα)
		1.60 (1H, m, Hβ)		1.59 (1H, m, Hβ)		1.45 (1H, qd, m, Hβ)
2	30.6	2.41 (1H, m, Hα)	30.5	2.41 (1H, m, Hα)	30.9	2.37 (1H, m, Hα)
		2.10 (1H, m, Hβ)		2.10 (1H, m, Hβ)		2.16 (1H, m, Hβ)
3	148.5		148.4		148.4	
4	55.9	1.94 (1H, m)	55.8	1.97 (1H, m)	55.6	1.90 (1H, m)
5	38.5		38.4		37.9	
6	34.8	1.92 (1H, m, Hα)	34.7	1.92 (1H, m, Hα)	34.1	1.83 (1H, brtd, 12.1, 5.7, Hα)
		1.42 (1H, m, Hβ)		1.42 (1H, m, Hβ)		1.32 (1H, brd, 13.1, Hβ)
7	22.4	1.87 (1H, m, Hα)	22.6	1.87 (1H, m, Hα)	26.9	1.70 (2H, m)
		1.76 (1H, m, Hβ)		1.67 (1H, m, Hβ)		
8	87.4	3.10 (1H, dd, 12.2, 3.3)	86.1	3.25 (1H, dd, 12.2, 3.2)	77.0	3.56 (1H, brdd, 9.4, 6.4)
9	44.5		45.0		43.9	
10	45.3	1.60 (1H, m)	44.9	1.60 (1H, m)	42.0	1.57 (1H, brdd, 12.7, 2.4)
11	47.9	1.83 (1H, m, Hα)	48.5	1.26 (1H, m, Hα)	53.6	2.78 (1H, d, 14.2, Ha)
		1.44 (1H, m, Hβ)		1.93 (1H, m, Hβ)		2.35 (1H, d, 14.2, Hb)
12	74.2	4.68 (1H, brtd, 9.3, 3.4)	73.7	4.81 (1H, td, 8.7, 7.0)	203.1	
13	127.3	5.36 (1H, brd, 8.8)	127.0	5.33 (1H, brd, 9.1)	125.8	6.16 (1H, brs)
14	135.4		134.7		156.2	
15	25.7	1.72 (3H, s)	25.9	1.73 (3H, s)	27.8	1.92 (3H, s)
16	18.1	1.70 (3H, s)	18.0	1.69 (3H, s)	20.9	2.14 (3H, s)
17	17.0	0.86 (3H, s)	15.7	0.87 (3H, s)	14.8	0.87 (3H, s)
18	24.5	0.95 (3H, s)	24.4	0.94 (3H, s)	22.9	0.94 (3H, s)
19	109.8	4.51 (1H, brs, Ha)	109.8	4.51 (1H, brs, Ha)	109.7	4.52 (1H, brs, Ha)
		4.20 (1H, brs, Hb)		4.20 (1H, brs, Hb)		4.18 (1H, brs, Hb)
20	20.0	2.66 (1H, dd, 12.8, 3.5, Ha)	20.1	2.66 (1H, dd, 12.8, 3.5, Ha)	19.7	2.62 (1H, dd, 12.8, 3.5, Ha)
		2.51 (1H, t, 12.8, Hb)		2.52 (1H, t, 12.8, Hb)		2.37 (1H, t, 12.8, Hb)
21	104.0		103.9		103.9	
22	180.5		180.4		180.4	
23	120.0		120.0		119.9	
24	155.0		155.0		155.2	
25	163.1		163.1		163.1	
26	59.5	4.58 (1H, d, 13.5, Ha)	59.5	4.56 (2H, brs)	59.5	4.55 (2H, brs)
		4.55 (1H, d, 13.5, Hb)				
27	9.5	1.97 (3H, s)	9.5	1.96 (3H, s)	9.5	1.95 (3H, s)
OMe	55.5	3.92 (3H, s)	55.5	3.91 (3H, s)	55.5	3.91 (3H, s)

[a] Assignment for all compounds were based on COSY, HSQC, HMBC experiments.

[b] in $CDCl_3$

Supplementary Table 16. ^{13}C (125 MHz) and ^{1}H HMR (500 MHz) data for **20** and **21**.[a,b]

	**20**			**21**		
Position	^{13}C	^{1}H (multi, J in Hz)		^{13}C	^{1}H (multi, J in Hz)	
1	24.7	1.53	(1H, m, Hα)	24.7	1.53	(1H, m, Hα)
		1.39	(1H, qd, 13.2, 4.4, Hβ)		1.40	(1H, qd, 13.0, 4.3, Hβ)
2	31.9	2.46	(1H, brtd, 13.2, 5.4, Hα)	31.9	2.42	(1H, brtd, 13.4, 5.3, Hα)
		2.16	(1H, m, Hβ)		2.16	(1H, m, Hβ)
3	149.1			148.8		
4	55.2	1.96	(1H, m)	54.9	1.98	(1H, dd, 11.8, 3.5)
5	37.4			37.4		
6	29.2	2.16	(1H, m, Hα)	29.2	2.12	(1H, td, 13.0, 3.8, Hα)
		1.03	(1H, brd, 13.0, Hβ)		1.02	(1H, brd, 13.0, Hβ)
7	22.8	1.88	(1H, m, Hα)	22.8	1.86	(1H, m, Hα)
		1.82	(1H, m, Hβ)		1.81	(1H, m, Hβ)
8	83.1	3.72	(1H, brs)	83.0	3.71	(1H, brs)
9	44.1			44.1		
10	38.6	1.71	(1H, m)	38.6	1.68	(1H, m)
11	49.0	2.20	(1H, m, Hα)	48.9	2.18	(1H, m, Hα)
		1.26	(1H, t, 11.0, Hβ)		1.26	(1H, dd, 12.6, 9.6, Hβ)
12	72.9	4.82	(1H, td, 9.1, 6.5)	72.9	4.80	(1H, td, 9.1, 6.5)
13	127.5	5.24	(1H, brd, 8.8)	127.4	5.24	(1H, brd, 8.9)
14	134.4			134.4		
15	25.9	1.72	(3H, s)	25.9	1.72	(3H, s)
16	18.1	1.72	(3H, s)	18.1	1.71	(3H, s)
17	20.7	0.96	(3H, s)	20.7	0.96	(3H, s)
18	21.4	0.94	(3H, s)	21.4	0.94	(3H, s)
19	109.6	4.51	(1H, brs, Ha)	110.0	4.56	(1H, brs, Ha)
		4.19	(1H, brs, Hb)		4.24	(1H, brs, Hb)
20	19.8	2.68	(1H, dd, 12.8, 3.9, Ha)	19.8	2.68	(1H, dd, 12.8, 3.9, Ha)
		2.55	(1H, t, 12.8, Hb)		2.54	(1H, t, 12.8, Hb)
21	104.2			106.6		
22	180.4			176.1		
23	120.0			119.0		
24	154.8			162.8		
25	162.9			162.5		
26	59.6	4.55	(2H, brs)	61.0	4.48	(2H, brs)
27	9.5	1.96	(3H, s)	166.3		
OMe	55.5	3.93	(3H, s)	56.1	3.96	(3H, s)
CO_2Me				53.0	3.92	(3H, s)

[a] Assignment for all compounds were based on COSY, HSQC, HMBC experiments.
[b] in $CDCl_3$

Supplementary Table 17. ^{13}C (125 MHz) and ^{1}H HMR (500 MHz) data for **22–24**.[a,b]

	**22**		**23**		**24**	
Position	^{13}C	^{1}H (multi, *J* in Hz)	^{13}C	^{1}H (multi, *J* in Hz)	^{13}C	^{1}H (multi, *J* in Hz)
1	24.8	1.53 (1H, m, Hα)	25.1	1.43 (1H, m, Hα)	25.0	1.44 (1H, m, Hα)
		1.39 (1H, qd, 13.2, 4.2, Hβ)		1.61 (1H, m, Hβ)		1.59 (1H, m, Hβ)
2	31.9	2.48 (1H, brtd, 13.2, 5.5, Hα)	30.5	2.42 (1H, m, Hα)	30.5	2.43 (1H, m, Hα)
		2.15 (1H, m, Hβ)		2.10 (1H, m, Hβ)		2.10 (1H, m, Hβ)
3	149.3		148.6		148.6	
4	55.3	1.97 (1H, dd, 12.0, 3.8)	56.0	1.96 (1H, m)	55.8	1.97 (1H, dd, 11.5, 3.5)
5	37.4		38.5		38.4	
6	29.2	2.17 (1H, m, Hα)	34.7	1.95 (1H, m, Hα)	34.7	1.93 (1H, m, Hα)
		1.03 (1H, brd, 13.3)		1.43 (1H, m, Hβ)		1.42 (1H, m, Hβ)
7	22.9	1.88 (1H, m, Hα)	22.4	1.87 (1H, m, Hα)	22.6	1.87 (1H, m, Hα)
		1.81 (1H, m, Hβ)		1.75 (1H, m, Hβ)		1.67 (1H, m, Hβ)
8	83.1	3.72 (1H, brs)	87.3	3.10 (1H, dd, 12.1, 3.3)	86.1	3.26 (1H, dd, 12.1, 3.3)
9	44.1		44.5		45.0	
10	38.6	1.74 (1H, m)	45.3	1.62 (1H, m)	44.9	1.60 (1H, m)
11	49.0	2.19 (1H, m, Hα)	47.9	1.84 (1H, m, Hα)	48.5	1.26 (1H, brt, 9.7, Hα)
		1.25 (1H, m, Hβ)		1.45 (1H, m, Hβ)		1.92 (1H, m, Hβ)
12	72.9	4.82 (1H, td, 9.1, 6.6)	74.1	4.68 (1H, brtd, 9.5, 3.4)	73.7	4.81 (1H, td, 8.7, 7.0)
13	127.6	5.24 (1H, brd, 8.8)	127.4	5.36 (1H, brd, 8.7)	127.0	5.34 (1H, brd, 9.1)
14	134.4		135.4		134.6	
15	25.9	1.72 (3H, s)	25.7	1.72 (3H, s)	25.9	1.73 (3H, s)
16	18.2	1.72 (3H, s)	18.1	1.70 (3H, s)	18.0	1.69 (3H, s)
17	20.8	0.96 (3H, s)	17.0	0.86 (3H, s)	15.7	0.87 (3H, s)
18	21.4	0.94 (3H, s)	24.4	0.95 (3H, s)	24.4	0.94 (3H, s)
19	109.4	4.52 (1H, brs, Ha)	109.6	4.51 (1H, brs, Ha)	109.6	4.52 (1H, brs, Ha)
		4.20 (1H, brs, Hb)		4.21 (1H, brs, Hb)		4.21 (1H, brs, Hb)
20	19.8	2.68 (1H, dd, 12.8, 3.8, Ha)	19.7	2.66 (1H, dd, 12.8, 3.4, Ha)	20.0	2.66 (1H, dd, 12.8, 3.5, Ha)
		2.54 (1H, t, 12.8, Hb)		2.50 (1H, t, 12.8, Hb)		2.50 (1H, t, 12.8, Hb)
21	103.5		103.2		103.2	
22	180.4		180.4		180.4	
23	118.5		118.6		118.6	
24	154.8		155.0		154.9	
25	162.7		162.9		162.9	
26	16.9	2.24 (3H, s)	16.9	2.25 (3H, s)	17.0	2.24 (3H, s)
27	10.1	1.90 (3H, s)	10.0	1.90 (3H, s)	10.0	1.90 (3H, s)
OMe	55.3	3.87 (3H, s)	55.3	3.86 (3H, s)	55.3	3.86 (3H, s)

[a] Assignment for all compounds were based on COSY, HSQC, HMBC experiments.

[b] in $CDCl_3$

Supplementary Table 18. ^{13}C (125 MHz) and ^{1}H HMR (500 MHz) data for **25**.[a,b]

Position	^{13}C	^{1}H (multi, *J* in Hz)
1	23.7	1.73 (1H, m, Hα)
		1.47 (1H, qd, 13.0, 4.4, Hβ)
2	30.9	2.39 (1H, m, Hα)
		2.16 (1H, m, Hβ)
3	148.6	
4	55.7	1.90 (1H, m)
5	37.9	
6	34.1	1.88 (1H, m, Hα)
		1.34 (1H, brd, 13.3, Hβ)
7	27.0	1.71 (2H, m)
8	76.8	3.60 (1H, brdd, 10.3, 5.3)
9	44.1	
10	41.9	1.61 (1H, m)
11	53.4	2.74 (1H, d, 13.9, Ha)
		2.38 (1H, d, 13.9, Hb)
12	203.0	
13	125.9	6.15 (1H, brs)
14	155.7	
15	27.7	1.92 (3H, s)
16	20.8	2.14 (3H, s)
17	15.0	0.88 (3H, s)
18	22.9	0.94 (3H, s)
19	109.5	4.53 (1H, brs, Ha)
		4.19 (1H, brs, Hb)
20	19.7	2.63 (1H, dd, 12.8, 3.4, Ha)
		2.35 (1H, t, 12.8, Hb)
21	103.2	
22	180.3	
23	118.6	
24	154.9	
25	162.9	
26	16.9	2.24 (3H, s)
27	10.0	1.89 (3H, s)
OMe	55.3	3.84 (3H, s)

[a] Assignment for all compounds were based on COSY, HSQC, HMBC experiments.
[b] in $CDCl_3$

Supplementary Table 19. Summary of *A. oryzae* transformants constructed in this study.

Pathway	Transformant	Original strain	Plasmids	Products (titer, mg/L)[a]	Culture
Intermediate **2**	*AO-dpasACD*	*AO*-NSAR1	pUARA2-*dpasACD*	**2** (62)	CPS medium_day 5
Intermediate **4**	*AO-dpasABCDE*	*AO-dpasACD*	pUADEA2-*dpasBE*	**4** (87)	CPS medium_day 6
	AO-dpasACD-dpfgBE	*AO-dpasACD*	pUADEA2-*dpfgBE*	**4** (86)	CPS medium_day 6
	AO-dpasACD-dpmpBE	*AO-dpasACD*	pUADEA2-*dpmpBE*	**4** (55)	CPS medium_day 6
	AO-dpasACD-dpchBE	*AO-dpasACD*	pUADEA2-*dpchBE*	**4** (91)	CPS medium_day 6
dpas (*A. sacchari*)	*AO-dpasABCDEF*	*AO-dpasABCDE*	pUSCA2-*dpasF*	**5** (62), **6** (5), **7** (6)	CPS medium_day 5
dpma (*M. anisopliae*)	*AO-dpasABCDE-dpmaF*	*AO-dpasABCDE*	pUPTRA2-*dpmaF*	**5** (89), **6** (6)	CPS medium_day 6
Intermediate **8**	*AO-dpasABCDE-dpmpG*	*AO-dpasABCDE*	pUPTRA2-*dpmpG*	**8** (23)	CPS medium_day 9
Intermediate **9**	*AO-dpasABCDE-dpmpGH*	*AO-dpasABCDE*	pUPTRA2-*dpmpGH*	**8** (11), **9** (28)	CPS medium_day 9
	AO-dpasABCDE-dpfgGH	*AO-dpasABCDE*	pUPTRA2-*dpfgGH*	**9** (17)	CPS medium_day 9
	AO-dpasACD-dpchBEGH	*AO-dpasACD*	pUADEA2-*dpchBE*<br>pUPTRA2-*dpchGH*	**9** (51)	CPS medium_day 5
dpch (*C. higginsianum*)	*AO-dpasACD-dpchBEFGH*	*AO-dpasACD-dpchBEGH*	pUSCA2-*dpchF*	**10** (28)	CPS medium_day 6
dpch + *dpmpI*	*AO-dpasACD-dpchBEFGH-dpmpI*	*AO-dpasACD-dpchBEGH*	pUSCA2-*dpmpI-dpchF*	**22** (17)	CPS medium_day 8
Intermediate **11**	*AO-dpasABCDE-dpmpGHI*	*AO-dpasABCDE-dpmpGH*	pUSCA2-*dpmpI*	**11** (29)	CPS medium_day 8
	AO-dpasABCDE-dpfgGHI	*AO-dpasABCDE-dpfgGH*	pUSCA2-*dpfgI*	**11** (20)	CPS medium_day 8
Shunt product **15**	*AO-dpasABCDE-dpfgI*	*AO-dpasABCDE*	pUSCA2-*dpfgI*	**15** (42)	CPS medium_day 9
dpmp (*M. phaseolina*)	*AO-dpasABCDE-dpmpGHIJ*	*AO-dpasABCDE-dpmpGH*	pUSCA2-*dpmpIJ*	**12** (21)	CPS medium_day 7
Shunt product **16**	*AO-dpasABCDE-dpmpIJ*	*AO-dpasABCDE*	pUSCA2-*dpmpIJ*	**16** (6)	1/2 CPS medium_day 5
dpmp + *dpasF*	*AO-dpasABCDEF-dpfgGH-dpmpIJ*	*AO-dpasABCDE-dpfgGH*	pUSCA2-*dpmpIJ-dpasF*	**17** (5), **18** (3), **19** (16), **20** (5)	CPS medium_day 8
dpfg (*F. graminearum*)	*AO-dpasABCDE-dpfgGHIJK*	*AO-dpasABCDE-dpfgGH*	pUSCA2-*dpfgIJK*	**13** (5), **14** (2)	CPS medium_day 6
	AO-dpasABCDE-dpfgIJK	*AO-dpasABCDE*	pUSCA2-*dpfgIJK*	**15** (40)	CPS medium_day 5
dpfg + *dpasF*	*AO-dpasABCDEF-dpmpGH-dpfgIJK*	*AO-dpasABCDE-dpmpGH*	pUSCA2-*dpfgIJK-dpasF*	**21** (6)	CPS medium_day 8
dpas + *dpmpI*	*AO-dpasABCDEF-dpmpI*	*AO-dpasABCDEF*	pUPTRA2-*dpmpI*	**23** (23), **24** (7), **25** (3)	1/2 CPS medium_day 6
	AO-dpasABCDE-dpchF	*AO-dpasABCDE*	pUSCA2-*dpchF*	**5** (65), **6** (12), **7** (trace)	CPS medium_day 9
	AO-dpasABCDEF-dpmpGH	*AO-dpasABCDE-dpmpGH*	pUSCA2-*dpasF*	**5** (23), **6** (4), **10** (9)	1/2 CPS medium_day 7
	AO-dpasACD-dpchBEGH-dpmaF	*AO-dpasACD-dpchBEGH*	pUSCA2-*dpmaF*	**9** (26)	1/2 CPS medium_day 6

[a] Titer of each compound was calculated from HPLC peak area compared with purified authentic samples (1 μg and 5 μg).

Supplementary Table 20. Inhibition of respiratory enzymes by selected DDPs.

Compound	NADH oxidase (I + III + IV)	Succinate-cyt. *c* oxidoreductase (II + III)
	IC_{50} (nM)	
**5**	21 (± 1.4)	46 (± 8.9)
**7**	7300 (± 140)	19000 (± 2700)
**11**	8.0 (± 0.5)	9.3 (± 0.3)
**12**	11 (± 0.4)	13 (± 1.5)
**14**	11 (± 0.3)	14 (± 1.1)
**16**	160 (± 10)	290 (± 22)
**17**	12 (± 0.9)	11 (± 0.9)
**20**	15 (± 0.6)	16 (± 1.5)
**21**	23 (± 2.1)	51 (± 4.3)
**22**	9.8 (± 0.5)	12 (± 2.0)
**23**	11 (± 0.2)	12 (± 0.8)
**25**	2700 (± 310)	10000 (± 940)
Antimycin A	7.2 (± 0.2)	8.0 (± 0.2)

Source data are provided in a Source Data file.

Supplementary Table 21. Log GI_{50} data of DDP compounds and complex III inhibitors across the JFCR39 panel and the MG-MID concentrations.

Compound	MG-MID	NCI-H23	NCI-H226	NCI-H522	NCI-H460	A549	DMS273	DMS114	HCC2998	KM-12
**7**	-4.355	-4.878	-4.476	-4.717	-4.934	-4.176	-4.850	-5.049	-4.000	-4.252
**11**	-5.193	-5.309	-4.741	-5.711	-7.063	-5.154	-6.702	-7.248	-4.435	-4.816
**12**	-5.359	-5.710	-4.935	-5.836	-7.132	-5.205	-7.063	-7.363	-4.803	-4.888
**13**	-4.817	-4.964	-4.773	-5.164	-4.952	-4.703	-4.789	-5.230	-4.753	-4.720
**14**	-5.183	-5.583	-4.932	-5.812	-7.134	-4.957	-6.539	-7.026	-4.776	-4.850
**16**	-5.160	-5.618	-5.269	-5.659	-6.167	-5.056	-5.822	-6.139	-4.871	-4.862
**17**	-5.138	-4.966	-4.853	-4.978	-7.038	-4.909	-6.147	-7.154	-4.853	-4.840
**19**	-4.470	-4.923	-4.719	-4.905	-5.222	-4.464	-5.001	-5.186	-4.000	-4.250
**20**	-5.210	-5.652	-4.866	-5.806	-7.091	-4.945	-7.049	-7.322	-4.799	-4.830
**21**	-4.812	-4.956	-4.741	-5.059	-4.917	-4.732	-4.779	-5.307	-4.741	-4.739
**22**	-5.515	-6.137	-5.421	-6.321	-7.093	-5.262	-6.649	-7.221	-5.053	-4.976
**23**	-5.441	-6.642	-4.971	-6.142	-7.117	-4.985	-6.577	-7.258	-4.920	-4.985
**24**	-5.364	-6.175	-4.976	-6.123	-7.045	-5.162	-6.315	-6.724	-4.895	-5.023
Antimycin A	-5.632	-7.957	-5.490	-5.947	-8.114	-5.012	-7.567	-8.273	-5.000	-5.000
Myxothiazol	-6.689	-7.011	-6.492	-7.434	-8.156	-6.253	-8.225	-8.261	-6.321	-6.000

Compound	HT-29	HCT-15	HCT-116	St-4	MKN1	MKN-B	MKN-A	MKN45	MKN74	HBC-4
**7**	-4.130	-4.255	-4.428	-4.160	-4.000	-4.424	-4.688	-4.347	-4.381	-4.000
**11**	-4.868	-5.915	-6.133	-4.604	-4.514	-5.432	-6.511	-4.731	-5.322	-4.446
**12**	-4.901	-5.533	-5.580	-4.852	-4.863	-5.948	-6.771	-4.873	-5.381	-4.861
**13**	-4.746	-4.732	-4.736	-4.660	-4.725	-4.804	-5.072	-4.776	-4.829	-4.661
**14**	-4.879	-4.993	-5.548	-4.837	-4.792	-5.697	-6.360	-4.887	-4.967	-4.794
**16**	-4.904	-5.220	-5.292	-4.853	-4.787	-5.237	-5.445	-4.853	-4.995	-4.804
**17**	-4.845	-6.026	-6.021	-4.818	-4.715	-4.998	-6.389	-4.855	-4.886	-4.780
**19**	-4.337	-4.277	-4.643	-4.123	-4.107	-4.818	-5.019	-4.113	-4.509	-4.000
**20**	-4.837	-4.995	-4.984	-4.846	-4.835	-5.975	-7.006	-4.838	-4.890	-4.807
**21**	-4.759	-4.755	-4.824	-4.700	-4.733	-4.774	-4.846	-4.791	-4.703	-4.775
**22**	-5.116	-5.922	-5.750	-4.958	-4.993	-6.268	-6.675	-4.985	-5.477	-4.923
**23**	-4.988	-6.007	-6.002	-4.906	-4.878	-5.814	-6.498	-4.955	-5.375	-4.878
**24**	-4.996	-5.486	-5.888	-4.915	-4.912	-5.892	-6.242	-5.039	-5.429	-4.873
Antimycin A	-5.000	-7.387	-7.118	-5.000	-5.000	-7.053	-5.698	-5.000	-5.220	-5.000
Myxothiazol	-6.405	-7.175	-7.415	-6.173	-6.652	-8.244	-7.276	-6.024	-6.568	-6.000

Compound	BSY-1	HBC-5	MCF-7	MDA-MB-231	OVCAR-3	OVCAR-4	OVCAR-5	OVCAR-8	SK-OV-3	U251
**7**	-4.432	-4.488	-4.645	-4.234	-4.687	-4.446	-4.000	-4.447	-4.216	-4.020
**11**	-4.940	-4.858	-5.726	-4.645	-5.442	-5.043	-4.373	-5.442	-4.912	-4.643
**12**	-5.304	-5.059	-6.069	-4.871	-5.329	-5.003	-4.711	-5.249	-4.900	-4.823
**13**	-4.858	-4.820	-4.860	-4.708	-4.966	-4.811	-4.762	-4.766	-4.789	-4.702
**14**	-4.990	-4.916	-5.620	-4.880	-4.988	-4.924	-4.730	-5.067	-4.866	-4.823
**16**	-4.961	-4.986	-5.351	-4.882	-5.447	-4.966	-4.859	-5.076	-4.993	-4.880
**17**	-4.893	-4.928	-5.209	-4.858	-4.978	-4.913	-4.810	-4.967	-4.916	-4.844
**19**	-4.477	-4.500	-4.596	-4.000	-4.736	-4.279	-4.000	-4.389	-4.080	-4.157
**20**	-4.912	-4.907	-5.395	-4.883	-5.419	-4.925	-4.786	-4.910	-4.876	-4.861
**21**	-4.828	-4.832	-4.849	-4.749	-4.920	-4.820	-4.780	-4.776	-4.761	-4.756
**22**	-5.539	-5.151	-5.825	-4.924	-5.631	-5.557	-4.874	-5.321	-4.974	-4.936
**23**	-4.988	-4.971	-5.995	-4.925	-5.833	-5.152	-4.812	-5.709	-4.968	-4.879
**24**	-5.018	-4.972	-5.817	-4.934	-5.571	-5.188	-4.838	-5.522	-4.971	-4.869
Antimycin A	-5.000	-5.000	-5.827	-5.060	-5.199	-5.000	-5.000	-5.000	-5.000	-5.000
Myxothiazol	-6.108	-6.000	-6.932	-6.014	-6.830	-6.064	-6.157	-7.095	-6.000	-6.000

Compound	SF-268	SF-295	SF-539	SNB-75	SNB-78	RXF-631L	ACHN	LOX-IMVI	DU-145	PC-3
**7**	-4.109	-4.595	-4.212	-4.000	-4.000	-4.181	-4.000	-4.912	-4.000	-4.081
**11**	-4.853	-4.929	-4.917	-4.556	-4.659	-4.715	-4.960	-5.264	-4.782	-5.221
**12**	-4.911	-5.487	-4.939	-4.735	-4.877	-4.793	-4.976	-6.218	-4.929	-5.308
**13**	-4.694	-4.924	-4.713	-4.725	-4.763	-4.774	-4.920	-4.863	-4.819	-4.829
**14**	-4.892	-4.940	-4.907	-4.696	-4.832	-4.838	-4.962	-4.968	-4.929	-4.995
**16**	-4.944	-5.553	-4.953	-4.827	-4.939	-4.839	-5.541	-5.224	-4.962	-5.211
**17**	-4.878	-4.972	-4.913	-4.772	-4.849	-4.841	-4.967	-4.984	-4.880	-4.953
**19**	-4.158	-4.765	-4.182	-4.269	-4.441	-4.000	-4.829	-4.868	-4.368	-4.618
**20**	-4.897	-4.951	-4.909	-4.784	-4.855	-4.770	-4.953	-4.997	-4.896	-4.944
**21**	-4.711	-4.917	-4.736	-4.750	-4.729	-4.761	-4.913	-4.821	-4.805	-4.834
**22**	-5.087	-5.611	-4.978	-5.000	-5.011	-5.094	-5.633	-5.930	-5.286	-5.512
**23**	-5.065	-5.951	-5.254	-4.875	-4.912	-4.992	-4.929	-6.219	-4.909	-4.962
**24**	-5.092	-5.656	-5.139	-4.856	-4.890	-4.961	-4.934	-6.032	-4.912	-4.928
Antimycin A	-5.000	-5.898	-5.000	-5.000	-5.214	-5.000	-5.398	-5.730	-5.000	-5.491
Myxothiazol	-6.383	-8.037	-7.311	-6.000	-6.000	-6.000	-6.000	-7.708	-6.146	-6.000

Supplementary Table 22. *COMPARE* analysis using representative DDP compounds as seeds.

A.	**7**			
Rank	r	JCI#	Chemical Name	Mode of action
1	0.748	-1210	Myxothiazol	Mitochondrial complex III inhibitor
2	0.716	-1528	Diphenyleneiodonium chloride	iNOS (Nitric Oxide Synthase) inhibitor
3	0.700	-1036	Pioglitazone	PPARgamma Agonist
4	0.682	-1525	Antimycin A	Mitochondrial complex III inhibitor
5	0.670	-695	(-)-Deguelin	Mitochondrial complex I inhibitor
6	0.661	-297	Rotenone	Mitochondrial complex I inhibitor
7	0.635	-1444	BIC1	BRD2 inhibitor
8	0.631	-1019	Metformin hydrochloride	Biguanide / Mitochondrial complex I inhibitor
9	0.612	-471	PD150606	Calpain inhibitor
10	0.604	-1020	Buformin	Biguanide / Mitochondrial complex I inhibitor

B.	**11**			
Rank	r	JCI#	Chemical Name	Mode of action
1	0.811	-1525	Antimycin A	Mitochondrial complex III inhibitor
2	0.779	-1528	Diphenyleneiodonium chloride	iNOS (Nitric Oxide Synthase) inhibitor
3	0.767	-1210	Myxothiazol	Mitochondrial complex III inhibitor
4	0.638	-764	TOFA	Acetyl-CoA Carboxylase 1/2 inhibitor
5	0.620	-502	SU1498	HER2 (erbB2) inhibitor
6	0.612	-1547	PAC-1	Procaspase 3 activator
7	0.610	-1621	GMX1778	Nampt inhibitor / NF-kappaB (NFKB) Activation inhibitor / inhibitory kappaB Kinase (IKK) inhibitor
8	0.608	-1471	Mubritinib	HER2/ErbB2 inhibitor
9	0.607	-1379	BMN-673	PARP-1/2 inhibitor
10	0.602	-1165	TW-37	Bcl-2 inhibitor

C.	**12**			
Rank	r	JCI#	Chemical Name	Mode of action
1	0.828	-1210	Myxothiazol	Mitochondrial complex III inhibitor
2	0.806	-1528	Diphenyleneiodonium chloride	iNOS (Nitric Oxide Synthase) inhibitor
3	0.804	-1525	Antimycin A	Mitochondrial complex III inhibitor
4	0.696	-1379	BMN-673	PARP-1/2 inhibitor
6	0.679	-695	(-)-Deguelin	Mitochondrial complex I inhibitor
7	0.644	-1036	Pioglitazone	PPARgamma Agonist
8	0.640	-764	TOFA	Acetyl-CoA Carboxylase 1/2 inhibitor
9	0.621	-1427	Enzalutamide	Androgen Receptor(AR) Antagonist
10	0.617	-1020	Buformin	Biguanide / Mitochondrial complex I inhibitor

D.	**13**			
Rank	r	JCI#	Chemical Name	Mode of action
1	0.714	-1020	Buformin	Biguanide / Mitochondrial complex I inhibitor
2	0.706	-1462	Arctigenin	Adiponectin Receptor 1 (ADIPOR1) agonist
3	0.697	-306	Polypropylene glycol	Agricultural Chemical
4	0.692	-1201	Sulfasalazine	NF-kappaB Activation inhibitor
5	0.688	-1531	Phenformin Hydrochloride	Biguanide / Mitochondrial complex I inhibitor
6	0.687	-515	Olvanil	TRPV1 (Transient Receptor Potential Vanilloid 1) agonist
7	0.685	-297	Rotenone	Mitochondrial complex I inhibitor
8	0.681	-1242	5,15-DPP	STAT3 inhibitor
9	0.677	-1528	Diphenyleneiodonium chloride	iNOS (Nitric Oxide Synthase) inhibitor
10	0.670	-1545	Oligomycin A	Mitochondrial complex V (Mitochondrial F1-ATPase) inhibitor

E.	14			
Rank	r	JCI#	Chemical Name	Mode of action
1	0.810	-1525	Antimycin A	Mitochondrial complex III inhibitor
2	0.792	-1528	Diphenyleneiodonium chloride	iNOS (Nitric Oxide Synthase) inhibitor
3	0.749	-1210	Myxothiazol	Mitochondrial complex III inhibitor
4	0.676	-1379	BMN-673	PARP-1/2 inhibitor
5	0.630	-1462	Arctigenin	Adiponectin Receptor 1 (ADIPOR1) agonist
6	0.618	-1471	Mubritinib	HER2/ErbB2 inhibitor
7	0.616	-1427	Enzalutamide	Androgen Receptor (AR) antagonist
8	0.615	-764	TOFA	Acetyl-CoA Carboxylase 1/2 inhibitor
9	0.599	-297	Rotenone	Mitochondrial complex I inhibitor
10	0.590	-1020	Buformin	Biguanide / Mitochondrial complex I inhibitor

F.	16			
Rank	r	JCI#	Chemical Name	Mode of action
1	0.824	-1525	Antimycin A	Mitochondrial complex III inhibitor
2	0.763	-1210	Myxothiazol	Mitochondrial complex III inhibitor
3	0.733	-1528	Diphenyleneiodonium chloride	iNOS (Nitric Oxide Synthase) inhibitor
4	0.690	-1379	BMN-673	PARP-1/2 inhibitor
5	0.655	-1	Carboquone	DNA alkylating agent
6	0.647	-471	PD150606	Calpain inhibitor
7	0.643	-1458	Clioquinol	Chelating Agent
8	0.632	-1462	Arctigenin	Adiponectin Receptor 1 (ADIPOR1) agonist
9	0.627	-1323	MLN4924	NAE inhibitor
10	0.627	-5	Cytarabine	Antimetabolites / DNA polymerase inhibitor

G.	17			
Rank	r	JCI#	Chemical Name	Mode of action
1	0.788	-1525	Antimycin A	Mitochondrial complex III inhibitor
2	0.747	-1528	Diphenyleneiodonium chloride	iNOS (Nitric Oxide Synthase) inhibitor
3	0.666	-1210	Myxothiazol	Mitochondrial complex III inhibitor
4	0.623	-1471	Mubritinib	HER2/ErbB2 inhibitor
5	0.600	-1379	BMN-673	PARP-1/2 inhibitor
6	0.577	-1621	GMX1778	Nampt) inhibitor
7	0.558	-1601	MPC-3100	Hsp90 inhibitor / non-geldanamycin inhibitor
8	0.546	-1143	DCA	Pyruvate Dehydrogenase Kinase (PDK) inhibitor
9	0.546	-757	Z-VAD-FMK	Caspase inhibitor
10	0.546	-393	Omeprazole sodium	H+/K+-ATPase

H.	19			
Rank	r	JCI#	Chemical Name	Mode of action
1	0.724	-1210	Myxothiazol	Mitochondrial complex III inhibitor
2	0.716	-1525	Antimycin A	Mitochondrial complex III inhibitor
3	0.704	-695	(-)-Deguelin	Mitochondrial complex I inhibitor
4	0.690	-1528	Diphenyleneiodonium chloride	iNOS (Nitric Oxide Synthase) inhibitor
5	0.673	-1427	Enzalutamide	Androgen Receptor(AR) antagonist
6	0.663	-1382	LGK974	Wnt Signaling inhibitor
7	0.638	-200	Flutamide	Androgen Receptor antagonist
8	0.637	-1020	Buformin	Biguanide / Mitochondrial complex I inhibitor
9	0.631	-1531	Phenformin Hydrochloride	Biguanide / Mitochondrial complex I inhibitor
10	0.623	-1444	BIC1	BRD2 inhibitor

I.	20			
Rank	r	JCI#	Chemical Name	Mode of action
1	0.750	-1525	Antimycin A	Mitochondrial complex III inhibitor
2	0.747	-1528	Diphenyleneiodonium chloride	iNOS (Nitric Oxide Synthase) inhibitor
3	0.728	-1210	Myxothiazol	Mitochondrial complex III inhibitor
4	0.645	-1462	Arctigenin	Adiponectin Receptor 1 (ADIPOR1) agonist
5	0.616	-1020	Buformin	Biguanide / Mitochondrial complex I inhibitor
6	0.615	-1427	Enzalutamide	Androgen Receptor(AR) antagonist
7	0.613	-1379	BMN-673	PARP-1/2 inhibitor
8	0.610	-297	Rotenone	Mitochondrial complex I inhibitor
9	0.603	-1471	Mubritinib	HER2/ErbB2 inhibitor

J.	**21**			
Rank	r	JCI#	Chemical Name	Mode of action
1	0.797	-1242	5,15-DPP	STAT3 inhibitor
2	0.777	-1201	Sulfasalazine	NF-kappaB activation inhibitor
3	0.767	-515	Olvanil	TRPV1 (Transient Receptor Potential Vanilloid 1) agonist
4	0.752	-1014	Bendamustine hydrochloride	DNA alkylating drug
5	0.738	-1462	Arctigenin	Adiponectin Receptor 1 (ADIPOR1) agonist
6	0.725	-1143	DCA	Pyruvate Dehydrogenase Kinase (PDK) inhibitor
7	0.725	-757	Z-VAD-FMK	Caspase inhibitor
8	0.725	-393	Omeprazole sodium	H+/K+-ATPase
9	0.720	-1531	Phenformin Hydrochloride	Biguanide / Mitochondrial complex I inhibitor
10	0.718	-1528	Diphenyleneiodonium chloride	iNOS (Nitric Oxide Synthase) inhibitor

K.	**22**			
Rank	r	JCI#	Chemical Name	Mode of action
1	0.847	-1525	Antimycin A	Mitochondrial complex III inhibitor
2	0.806	-1210	Myxothiazol	Mitochondrial complex III inhibitor
3	0.780	-1528	Diphenyleneiodonium chloride	iNOS (Nitric Oxide Synthase) inhibitor
4	0.670	-1020	Buformin	Biguanide / Mitochondrial complex I inhibitor
5	0.668	-695	(-)-Deguelin	Mitochondrial complex I inhibitor
6	0.666	-1427	Enzalutamide	Androgen Receptor(AR) antagonist
7	0.661	-1462	Arctigenin	Adiponectin Receptor 1 (ADIPOR1) agonist
8	0.620	-764	TOFA	Acetyl-CoA Carboxylase 1/2 inhibitor
9	0.611	-1379	BMN-673	PARP-1/2 inhibitor
10	0.607	-1036	Pioglitazone	PPARgamma agonist

L.	**23**			
Rank	r	JCI#	Chemical Name	Mode of action
1	0.876	-1210	Myxothiazol	Mitochondrial complex III inhibitor
2	0.865	-1525	Antimycin A	Mitochondrial complex III inhibitor
3	0.796	-1528	Diphenyleneiodonium chloride	iNOS (Nitric Oxide Synthase) inhibitor
4	0.667	-695	(-)-Deguelin	Mitochondrial complex I inhibitor
5	0.647	-1458	Clioquinol	Chelating Agent
6	0.640	-764	TOFA	Acetyl-CoA Carboxylase 1/2 inhibitor
7	0.630	-1379	BMN-673	PARP-1/2 inhibitor
8	0.625	-1020	Buformin	Biguanide / Mitochondrial complex I inhibitor
9	0.618	-1531	Phenformin Hydrochloride	Biguanide / Mitochondrial complex I inhibitor
10	0.615	-297	Rotenone	Mitochondrial complex I inhibitor

M.	**24**			
Rank	r	JCI#	Chemical Name	Mode of action
1	0.877	-1210	Myxothiazol	Mitochondrial complex III inhibitor
2	0.842	-1525	Antimycin A	Mitochondrial complex III inhibitor
3	0.790	-1528	Diphenyleneiodonium chloride	iNOS (Nitric Oxide Synthase) inhibitor
4	0.700	-764	TOFA	Acetyl-CoA Carboxylase 1/2 inhibitor
5	0.677	-695	(-)-Deguelin	Mitochondrial complex I inhibitor
6	0.671	-1379	BMN-673	PARP-1/2 inhibitor
7	0.665	-1458	Clioquinol	Chelating Agent
8	0.656	-1036	Pioglitazone	PPARgamma agonist
9	0.629	-1020	Buformin	Biguanide / Mitochondrial complex I inhibitor
10	0.612	-1531	Phenformin Hydrochloride	Biguanide / Mitochondrial complex I inhibitor

Supplementary Table 23. Anti-proliferative activity against *Drosophila* cell lines. *Drosophila* l(2)mbn cells or DL1 cells were treated with indicated compounds and the viability were measured by CellTiter-Glo Luminescent Cell Viability Assay. Data are means ± SEM from a single experiment and are representative of more than two independent experiments.

Compound	Mbn	DL-1
	IC_{50} (μM)	
**4**	6.44±0.94	1.54±0.08
**5**	0.19±0.01	0.012±0.001
**6**	0.85±0.01	0.23±0.07
**7**	> 20	8.75±0.64
**8**	5.14±0.30	5.02±0.22
**9**	1.54±0.01	0.36±0.03
**10**	0.63±0.10	0.13±0.01
**11**	0.47±0.21	0.40±0.02
**12**	3.29±0.16	0.34±0.03
**13**	8.46±0.98	0.29±0.02
**14**	3.74±0.14	0.44±0.06
**15**	5.23±0.19	6.10±1.57
**17**	1.78±0.43	2.78±0.68
**18**	6.00±1.09	0.25±0.02
**19**	> 20	10.56±0.23
**20**	1.45±0.13	0.27±0.03
**21**	2.66±0.22	0.015±0.001
**23**	1.58±0.35	0.13±0.04
**24**	3.04±0.16	0.24±0.01
**25**	> 20	17.16±2.06

Source data are provided in a Source Data file.

Supplementary Table 24. Selected anti-HIV-1 activity of DDPs on MAGI assay.

Compound	EC_{50} (nM)	CC_{50} (nM)	S.I. (CC_{50}/EC_{50})
**5**	38.9	>10000	>257
**10**	162.6	>10000	>62
**11**	48.3	6650.3	138
**12**	23.2	8815.7	380
**14**	53.2	>10000	>188
**17**	45.7	>10000	>219
**18**	68.8	>10000	>145
**20**	8.5	>10000	>1176
**23**	76.0	9041.5	118
**24**	156.8	>10000	>64
AZT	29.7	>10000	>337

Source data are provided in a Source Data file.

Supplementary Table 25. ^{13}C (125 MHz) and ^{1}H HMR (500 MHz) data for **26** and **27**.[a,b]

	**26**		**27**	
Position	^{13}C	^{1}H (multi, *J* in Hz)	^{13}C	^{1}H (multi, *J* in Hz)
1	22.7	1.57 (1H, m, Ha)	22.7	1.58 (1H, m, Ha)
		1.30 (1H, m, Hb)		1.28 (1H, m, Hb)
2	31.3	2.23 (1H, m, Ha)	31.3	2.40 (1H, brtd, 13.4, 5.4, Ha)
		2.17 (1H, m, Hb)		2.19 (1H, m, Hb)
3	148.1		148.3	
4	56.6	1.71 (1H, m)	55.6	1.91 (1H, m)
5	37.9		37.9	
6	28.4	2.08 (1H, m, Ha)	28.4	2.06 (1H, m, Ha)
		0.89 (1H, m, Hb)		0.92 (1H, m, Hb)
7	25.6	1.92 (1H, m, Ha)	25.7	1.96 (1H, m, Ha)
		1.59 (1H, m, Hb)		1.58 (1H, m, Hb)
8	72.1	3.60 (1H, brs)	72.2	3.60 (1H, brs)
9	39.5		39.4	
10	40.0	1.71 (1H, m)	40.2	1.66 (1H, m)
11	39.6	1.35 (2H, m)	39.6	1.25-1.40 (2H, m)
12	21.5	2.04 (1H, m, Ha)	21.5	2.04 (1H, m, Ha)
		1.93 (1H, m, Hb)		1.91 (1H, m, Hb)
13	125.1	5.13 (1H, m)	125.2	5.10 (1H, m)
14	131.5		131.4	
15	25.7	1.69 (3H, s)	25.7	1.68 (3H, s)
16	17.6	1.62 (3H, s)	17.6	1.61 (3H, s)
17	18.9	0.81 (3H, s)	18.9	0.82 (3H, s)
18	22.8	0.93 (3H, s)	22.8	0.96 (3H, s)
19	110.9	4.67 (1H, brs, Ha)	110.7	4.73 (1H, brs, Ha)
		4.42 (1H, brs, Hb)		4.46 (1H, brs, Hb)
20	25.6	2.31 (1H, m, Ha)	26.5	2.15 (1H, dd, 9.1, 10.6, Ha)
		2.10 (1H, m, Hb)		1.91 (1H, m, Hb)
21	52.5	4.37 (1H, dd, 8.4, 5.2)	52.3	4.48 (1H, m)
22	193.4		194.6	
23	95.1		94.3	
24	173.6		174.1	
25	171.6		170.8	
26	63.7	4.67 (2H, brs)	63.7	4.71 (1H, d, 16.0, Ha)
				4.68 (1H, d, 16.0, Hb)
27	170.3		170.1	
OMe	52.0	3.64 (3H, s)	51.8	3.66 (3H, s)
1'	171.9		171.9	
2'	53.3	4.42 (1H, m)	53.3	4.42 (1H, m)
3'	32.3	1.86 (1H, m, Ha)	32.3	1.88 (1H, m, Ha)
		1.67 (1H, m, Hb)		1.69 (1H, m, Hb)
4'	22.2	1.24-1.45 (2H, m)	22.2	1.36 (2H, m)
5'	28.9	1.67 (1H, m, Ha)	28.8	1.68 (1H, m, Ha)
		1.60 (1H, m, Hb)		1.60 (1H, m, Hb)
6'	44.6	3.10 (2H, m)	44.6	3.10 (2H, m)
7'-NH		9.31 (1H, brs)		9.36 (1H, brs)
8'-NH		5.36 (1H, brd, 7.5)		5.35 (1H, brd, 7.5)
1"	67.4	5.22 (1H, d, 12.1, Ha)	67.4	5.22 (1H, d, 12.5, Ha)
		5.15 (1H, d, 12.1, Hb)		5.14 (1H, d, 12.5, Hb)
2"	135.1		135.1	
3"-5"[c]	128.1-128.7	7.30-7.40	128.1-128.7	7.30-7.40
1'''	155.9		155.9	
2'''	67.1	5.12 (2H, m)	67.1	5.10 (2H, m)
3'''	136.1		136.1	
4'''-6'''[c]	128.1-128.7	7.30-7.40	128.1-128.7	7.30-7.40

[a] Assignment for all compounds were based on COSY, HSQC, HMBC experiments.
[b] in $CDCl_3$

Supplementary Note 1. Construction of fungal expression vector pUADEA2, pUSCA2 and pUPTRA2

To construct the fungal expression vectors with *adeA*, *sC* and *ptrA* gene as selectable marker, the region encoding *argB* gene of pUARA2[1] was replaced with that of *adeA*, *sC* and *ptrA* respectively (Supplementary Fig. 3). The region encoding *adeA* gene was amplified by PCR from genomic DNA of *A. nidulans* FGSC A4 with primers NadeA-pU-IFSphI-FW and NadeA-pU-IFXbaI-RV (Supplementary Table 4) as previously reported[2]. The region encoding *sC* gene was amplified by PCR from pUSA vector[3] with primers sC-pU-IFSphI-FW and sC-pU-IFXbaI-RV (Supplementary Table 4). The region encoding *ptrA* gene was amplified by PCR from pPTRI (Takara Bio) vector with primers ptrA-pU-IFSphI-FW and ptrA-pU-IFXbaI-RV (Supplementary Table 4). Linearized pUARA2 lacking the region encoding *argB* gene was prepared by digestion with *Xba*I and *Sph*I. The marker genes and the linearized vector were ligated by In-Fusion® HD Cloning Kit (Takara Bio) to yield the plasmid designated as pUADEA2, pUSCA2, and pUPTRA2.

Supplementary Note 2. Construction of *AO-dpasACD* and *AO-dpasACD-dpxxBE*

The NR-PKS, GGPPS and PT genes, *dpasACD* were introduced into *A. oryzae* NSAR1 wild type strain with pUARA2-*dpasACD* to construct *AO-dpasACD* (α-pyrone **2** producing strain). The FMOep and TC genes, *dpasBE*, *dpfgBE*, *dpmpBE*, and *dpchBE* were introduced into *AO-dpasACD* with pUADEA2-*dpasBE*, pUADEA2-*dpfgBE*, pUADEA2-*dpmpBE*, or pUADEA2-*dpchBE*, respectively, to construct *AO-dpasABCDE*, *AO-dpasACD-dpfgBE*, *AO-dpasACD-dpmpBE*, and *AO-dpasACD-dpchBE*.

Supplementary Note 3. Isolation and characterization of 2 and 4

AO-dpasABCDE was cultivated (150 mL x 20) for 1 week (30 °C, 150 rpm) and the mycelium (20 g dry weight) was extracted with EtOAc (20% MeOH). The extract was subjected to silica gel column chromatography (*n*-Hexane/EtOAc, 4:1 to 1:2) afforded **2** (13 mg) and **4** containing fraction (almost pure). A portion of crude **4** was further purified by preparative TLC (SiO_2, $CHCl_3$/MeOH, 40 : 1) to afford **4** (20.0 mg). Compound **2** was identified by comparing its MS data and NMR data with those reported.[4]

Compound **2**: ESIMS: *m/z* 413 $[M+H]^+$. ^{1}H NMR (500 MHz, $CDCl_3$) δ_H 5.34 (1H, brt, 7.5 Hz), 5.10 (2H, m), 5.07 (1H, m), 3.29 (2H, d, 7.5 Hz), 2.20 (3H, s), 2.13 (4H, m), 2.05 (4H, m), 1.98 (4H, m), 1.88 (3H, s), 1.80 (3H, s), 1.68 (3H, s), 1.61 (3H, s), 1.60 (3H, s), 1.59 (3H, s) ; ^{13}C NMR (125 MHz, $CDCl_3$) δ_C 165.9, 165.2, 156.0, 141.8, 136.1, 135.0, 131.2, 124.3, 124.1, 123.1, 120.4, 107.0, 100.4, 39.7, 39.6 (x2), 26.7, 26.5, 26.0, 25.7, 23.2, 17.6, 17.1, 16.3, 16.1, 16.0, 9.6.

Compound **4**: Colorless oil; $[\alpha]_D^{23}$ −38.7 (*c* 0.60, EtOH); UV (MeOH) λ_{max} nm (log ε) 292 (3.80); IR (KBr) ν_{max} (cm^{-1}) 3328, 2935, 2872, 1671, 1645, 1566; HRESIMS: *m/z* 429.3007 $[M+H]^+$ (429.3005 calcd

for $C_{27}H_{41}O_4$); ^{1}H and ^{13}C NMR data are shown in Supplementary Table 7.

Supplementary Note 4. Determination of absolute stereochemistry of 4

The relative configuration of **4** was assigned as 4*R**, 5*R**, 8*S**, 9*S**, 10*R** based on the NOE correlation as described above. The absolute stereochemistry at C-8 in **4** was determined to be *S* by applying modified Mosher's method.[5] (*S*)- and (*R*)-MTPA esters of **15** (**15b**, **15c**) were prepared from **4** and Δδ values for **15b** and **15c** were calculated from the ^{1}H NMR date (Supplementary Table 8), revealing the stereochemistry of 8*S*. Thus, all the stereochemistry could be determined to be 4*R*, 5*R*, 8*S*, 9*S*, 10*R*.

Supplementary Note 5. Conversion of 4 into γ-pyrone 15a

To a solution of **4** (2.5 mg, 5.8 μmol) in MeOH (20 μL), 10% trimethylsilyldiazomethane in hexane (30 μL) was added at room temperature. After being stirred for 1 hour, the mixture was concentrated *in vacuo*. The crude residue was subjected to purification by preparative TLC on silica gel ($CHCl_3$-MeOH 40:1) to afford **15a** (1.3 mg) as colorless oil.

Supplementary Note 6. Preparation of (*S*)- and (*R*)-MTPA esters of 15a (15b, 15c)

15a (1.0 mg, 2.3 μmol) was reacted with (*R*)-MTPA chloride (2.5 μL, 13.4 μmol) in 20 μL of pyridine at room temperature. After being stirred for 2 hours, the mixture was extracted with EtOAc and water, and the organic layer was concentrated *in vacuo*. The crude residue was subjected to purification by preparative TLC on silica gel ($CHCl_3$-EtOAc 16:1) to afford (*S*)-MTPA ester **15b** (1.0 mg) as colorless oil.

15a (1.3 mg, 2.9 μmol) was reacted with (*S*)-MTPA chloride (2.0 μL, 10.7 μmol) in pyridine (20 μL) at room temperature. After being stirred for 2 hours, the mixture was extracted with EtOAc and water, and the organic layer was concentrated *in vacuo*. The crude residue was subjected to purification by preparative TLC on silica gel ($CHCl_3$-EtOAc 16:1) to afford (*R*)-MTPA ester **15c** (2.0 mg) as colorless oil.

(*S*)-MTPA ester of **15** (**15b**): Colorless oil; HRESIMS: *m*/*z* 659.3531 $[M+H]^+$ (659.3554 calcd for $C_{38}H_{50}O_6F_3$); ^{1}H NMR data are shown in Supplementary Table 8.

(*R*)-MTPA ester of **15** (**15c**): Colorless oil; HRESIMS: *m*/*z* 659.3508 $[M+H]^+$ (659.3554 calcd for $C_{38}H_{50}O_6F_3$); ^{1}H NMR data are shown in Supplementary Table 8.

Supplementary Note 7. Construction of *AO-dpasABCDEF* and *AO-dpasABCDE-dpmaF*

The FMO gene *dpasF* and BBE gene *dpmaF* was introduced into *AO-dpasABCDE* (common intermediate **4** producing strain) with pUSCA2-*dpasF* or pUPTRA2-*dpmaF*, respectively, to construct *AO-dpasABCDEF* and *AO-dpasABCDE-dpmaF*.

Supplementary Note 8. Isolation and characterization of 5, 6 and 7

AO-dpasABCDE-dpmaF was cultivated (150 mL x 10) for 1 week (30 °C, 150 rpm) and the mycelium of (10 g dry weight) was extracted with EtOAc (20% MeOH). The extract was subjected to silica gel column chromatography (*n*-Hexane/EtOAc, 4 : 1 to 1 : 4) to give crude **5** and **6**. A portion of crude **5** and **6** were further purified by preparative TLC (SiO_2, *n*-hexane/EtOAc, 1 : 1) to afford subglutinol A (**5**, 10.4 mg) and subglutinol B (**6**, 3.7 mg). *AO-dpasABCDEF* was cultivated (150 mL x 20) for 1 week (30 °C, 150 rpm) and the culture media was extracted with EtOAc. The EtOAc extract was subjected to silica gel column chromatography (*n*-Hexane/EtOAc, 2 : 1 to 1 : 4) and preparative TLC (SiO_2, *n*-hexane/EtOAc, 1 : 2) to afford **7** (4.5 mg).

Subglutinol A (**5**): Colorless amorphous; $[\alpha]_D^{24}$ −32.1 (*c* 1.30, $CHCl_3$), lit. $[\alpha]_D^{25}$ −59.5 (*c* 0.12, MeOH)[6]; UV (MeOH) λ_{max} nm (log ε) 292 (3.90); IR (KBr) ν_{max} (cm^{-1}) 3271, 2968, 2927, 2869, 1668, 1643, 1563; HRESIMS: *m/z* 427.2841 $[M+H]^+$ (427.2843 calcd for $C_{27}H_{39}O_4$); ^1H and ^{13}C NMR data are shown in Supplementary Table 9.

Subglutinol B (**6**): Colorless amorphous; $[\alpha]_D^{24}$ −33.8 (*c* 0.27, $CHCl_3$), lit. $[\alpha]_D^{26.8}$ −73.3 (*c* 0.11, MeOH)[6]; UV (MeOH) λ_{max} nm (log ε) 292 (3.88); IR (KBr) ν_{max} (cm^{-1}) 3274, 2928, 2867, 1669, 1643, 1566; HRESIMS: *m/z* 449.2662 $[M+Na]^+$ (449.2662 calcd for $C_{27}H_{38}O_4Na$); ^1H and ^{13}C NMR data are shown in Supplementary Table 9.

Compound **7** (FDDP A): Colorless amorphous; $[\alpha]_D^{23}$ −102.2 (*c* 0.33, MeOH); UV (MeOH) λ_{max} nm (log ε) 242 (4.13), 293 (3.91); IR (KBr) ν_{max} (cm^{-1}) 3384, 2973, 2941, 2874, 1671, 1644, 1610, 1567; HRESIMS: *m/z* 465.2614 $[M+Na]^+$ (465.2611 calcd for $C_{27}H_{38}O_5Na$); ^1H and ^{13}C NMR data are shown in Supplementary Table 9.

Supplementary Note 9. Construction of *AO-dpasABCDE-dpmpG*, *AO-dpasABCDE-dpmpGH*, *AO-dpasABCDE-dpfgGH* and *AO-dpasACD-dpchBEGH*

The SDR1 gene, *dpmpG* was introduced into *AO-dpasABCDE* with pUPTRA2-*dpmpG* to construct *AO-dpasABCDE-dpmpG*. The SDR1 and SDR2 genes, *dpmpGH* and *dpfgGH* were introduced into *AO-dpasABCDE* with pUPTRA2-*dpmpGH* or pUPTRA2-*dpfgGH*, respectively, to construct *AO-dpasABCDE-dpmpGH* and *AO-dpasABCDE-dpfgGH*, while *dpchGH* were introduced into *AO-dpasACD-dpchBE*, which also produces common intermediate **4**, with pUPTRA2-*dpchGH* to construct *AO-dpasACD-dpchBEGH*.

Supplementary Note 10. Isolation and characterization of 8 and 9

AO-dpasABCDE-dpmpGH was cultivated (150 mL x 24) for 1 week (at 30 °C, 150 rpm) and the mycelium (24 g dry weight) was extracted with EtOAc (20% MeOH). The extract was subjected to silica gel column

chromatography (*n*-Hexane/EtOAc, 4:1 to 1:4) to give crude **8** and **9**. Finally, a portion of clude **8** and **9** were further purified by preparative TLC (ODS, CH_3CN/H_2O, 18 : 7 for **8**, CH_3CN/H_2O, 7 : 1 for **9**) to afford **8** (10.1 mg) and higginsianin B (**9**) (26.5 mg).

Compound **8**: Colorless oil; $[\alpha]_D^{23}$ −20.0 (*c* 0.10, $CHCl_3$); UV (MeOH) λ_{max} nm (log ε) 292 (3.79); IR (KBr) ν_{max} (cm^{-1}) 3296, 2965, 2932, 2875, 1697, 1669, 1648, 1567; HRESIMS: *m/z* 449.2663 $[M+Na]^+$ (449.2662 calcd for $C_{27}H_{38}O_4Na$); ^1H and ^{13}C NMR data are shown in Supplementary Table 10.

Higginsianin B (**9**): Colorless amorphous; $[\alpha]_D^{23}$ −57.9 (*c* 0.17, $CHCl_3$), lit. $[\alpha]_D^{25}$ −68.8 (*c* 0.25, MeOH)[7]; UV (MeOH) λ_{max} nm (log ε) 293 (3.96); IR (KBr) ν_{max} (cm^{-1}) 3216, 2962, 2928, 2887, 1666, 1644, 1571; HRESIMS: *m/z* 451.2813 $[M+Na]^+$ (451.2819 calcd for $C_{27}H_{40}O_4Na$); ^1H and ^{13}C NMR data are shown in Supplementary Table 10.

Supplementary Note 11. Construction of *AO-dpasACD-dpchBEFGH*

The FMO gene, *dpchF*, was introduced into *AO-dpasACD-dpchBEGH* (higginsianin B (**9**) producing strain) with pUSCA2-*dpchF* to construct *AO-dpasACD-dpchBEFGH*.

Supplementary Note 12. Isolation and characterization of 10

AO-dpasACD-dpchBEFGH was cultivated (150 mL x 12) for 1 week (30 °C, 150 rpm) and the mycelium (11 g dry weight) was extracted with EtOAc (20% MeOH). The extract was subjected to silica gel column chromatography (*n*-Hexane/EtOAc, 4 : 1 to 1 : 2). A portion of crude **10** was further purified by preparative TLC (SiO_2, *n*-hexane/EtOAc, 1 : 1) to give higginsianin A (**10**) (10.4 mg).

Higginsianin A (**10**): Colorless amorphous; $[\alpha]_D^{24}$ −41.3 (*c* 0.13, MeOH), lit. $[\alpha]_D^{25}$ −67.3 (*c* 0.30, MeOH)[7]; UV (MeOH) λ_{max} nm (log ε) 293 (3.88); IR (KBr) ν_{max} (cm^{-1}) 3297, 2929, 2862, 1673, 1644, 1568; HRESIMS: *m/z* 449.2661 $[M+Na]^+$ (449.2662 calcd for $C_{27}H_{38}O_4Na$); ^1H and ^{13}C NMR data are shown in Supplementary Table 11.

Supplementary Note 13. Construction of *AO-dpasABCDE-dpmpGHI*, *AO-dpasABCDE-dpmpGHIJ*, *AO-dpasABCDE-dpfgGHI* and *AO-dpasABCDE-dpfgGHIJK*

The MT1 gene *dpmpI* was introduced into *AO-dpasABCDE-dpmpGH* (higginsianin B (**9**) producing strain) with pUSCA2-*dpmpI* to construct *AO-dpasABCDE-dpmpGHI*. In the same manner, The MT1 gene *dpfgI* was introduced into *AO-dpasABCDE-dpfgGH* (higginsianin B (**9**) producing strain) with pUSCA2-*dpfgI* to construct *AO-dpasABCDE-dpfgGHI*. The MT1 gene *dpmpI* and the P450 gene *dpmpJ* were introduced into *AO-dpasABCDE-dpmpGH* with pUSCA2-*dpmpIJ* to construct *AO-dpasABCDE-dpmpGHIJ*. The MT1 gene *dpfgI*, P450 gene *dpfgJ* and MT2 gene *dpfgK* were introduced

into *AO-dpasABCDE-dpfgGH* with pUSCA2-*dpfgIJK* to construct *AO-dpasABCDE-dpfgGHIJK*.

Supplementary Note 14. Isolation and characterization of 11-14

AO-dpasABCDE-dpmpGHI was cultivated (150 mL x 3) for 1 week (30 ^{o}C, 150 rpm) and the mycelium (5 g dry weight) was extracted with EtOAc (20% MeOH). The extract was subjected to silica gel column chromatography (*n*-Hexane/EtOAc, 4 : 1 to 1 : 4) to give crude **11**. Finally, it was further purified by preparative TLC (SiO_2, *n*-Hexane/EtOAc, 1 : 1) to afford **11** (6.0 mg).

AO-dpasABCDE-dpmpGHIJ was cultivated (150 mL x 2) for 1 week (30 ^{o}C, 150 rpm) and the mycelium (3 g dry weight) was extracted with EtOAc (20% MeOH). The extract was subjected to silica gel column chromatography (*n*-Hexane/EtOAc, 4 : 1 to EtOAc) to give crude **12**. Finally, a portion of crude **12** was further purified by preparative TLC (SiO_2, *n*-Hexane/EtOAc, 1:4) to afford **12** (5.3 mg).

AO-dpasABCDE-dpfgGHIJK was cultivated (150 mL x 16) for 1 week (30 ^{o}C, 150 rpm) and the mycelium (16 g dry weight) was extracted with EtOAc (20% MeOH). The extract was subjected to silica gel column chromatography (*n*-Hexane/EtOAc, 4:1 to EtOAc) to give crude **13** and **14**. Finally, a portion of them were further purified by preparative TLC (SiO_2, *n*-Hexane/EtOAc, 1:4) to afford **13** (4.9 mg) and **14** (2.9 mg).

Compound **11** (FDDP B): Colorless oil; $[\alpha]_D^{24}$ −56.4 (*c* 0.14, MeOH); UV (MeOH) λ_{max} nm (log ε) 260 (3.90); IR (KBr) ν_{max} (cm^{-1}) 3418, 2925, 2881, 1669, 1586; HRESIMS: *m/z* 443.3155 $[M+H]^+$ (443.3156 calcd for $C_{28}H_{43}O_4$); ^{1}H and ^{13}C NMR data are shown in Supplementary Table 12.

Compound **12** (FDDP C): Colorless oil; $[\alpha]_D^{24}$ −64.0 (*c* 0.23, MeOH); UV (MeOH) λ_{max} nm (log ε) 266 (3.81); IR (KBr) ν_{max} (cm^{-1}) 3383, 2955, 2925, 2880, 2857, 1662, 1578; HRESIMS: *m/z* 459.3103 $[M+H]^+$ (459.3105 calcd for $C_{28}H_{43}O_5$); ^{1}H and ^{13}C NMR data are shown in Supplementary Table 12.

Compound **13** (FDDP D): Colorless oil; $[\alpha]_D^{23}$ −71.4 (*c* 0.29, $CHCl_3$); UV (MeOH) λ_{max} nm (log ε) 264 (3.73); IR (KBr) ν_{max} (cm^{-1}) 3420, 2951, 2925, 2881, 1737, 1661, 1618, 1577; HRESIMS: *m/z* 503.3007 $[M+H]^+$ (503.3009 calcd for $C_{29}H_{43}O_7$); ^{1}H and ^{13}C NMR data are shown in Supplementary Table 13.

Compound **14** (FDDP E): Colorless oil; $[\alpha]_D^{24}$ −78.6 (*c* 0.17, $CHCl_3$); UV (MeOH) λ_{max} nm (log ε) 262 (3.78); IR (KBr) ν_{max} (cm^{-1}) 3460, 2950, 2926, 2880, 1735, 1666, 1623, 1584; HRESIMS: *m/z* 509.2874 $[M+Na]^+$ (509.2874 calcd for $C_{29}H_{42}O_6Na$); ^{1}H and ^{13}C NMR data are shown in Supplementary Table 13.

Supplementary Note 15. Investigation of substrate selectivities of the modification enzymes in the DDP pathways

The three oxidase genes *dpmaF* (BBE), *dpchF* (FMO) and *dpasF* (FMO) are involved in the formation of the THF ring at the C_5 unit in each pathway. The two genes *dpchF* and *dpasF* encode FMOs, whose

producing enzymes show moderate homology. To investigate the substrate selectivities of DpmaF, DpchF and DpasF, we introduced *dpmaF* and *dpasF* into a **9**-producing transformant, and *dpchF* into a **4**-producing transformant (Supplementary Fig. 29). The results revealed that the two FMOs, DpchF and DpasF, accepted both **4** (8*S*) and **9** (8*R*) as substrates, whereas DpmaF accepted only **4** (8*S*). DpchF also constructed the enone at C_5 unit of **4** (8*S*) in the same manner as DpasF, although the conversion rate was much lower than that of DpasF. Neither DpchF and DpasF could introduce the enone system into the 8*R* substrate. We also investigated the substrate selectivities of MT1s (DpmpI and DpfgI) and P450s (DpmpJ and DpfgJ) by a heterologous expression study and constructed *AO-dpasABCDE-dpfgI*, *AO-dpasABCDE-dpmpIJ* and *AO-dpasABCDE-dpfgIJK*. *AO-dpasABCDE-dpfgI* and *AO-dpasABCDE-dpmpIJ,* which encode the shunt pathway, provided **15** (42 mg L^{-1}) and **16** (6 mg L^{-1}), respectively, while *AO-dpasABCDE-dpfgIJK* accumulated **15** (Supplementary Fig. 31). The results indicated that the MT1s, DpmpI and DpfgI, accepted both **4** (8*S*) and **9** (8*R*), and one P450, DpmpJ, catalysed hydroxylation at the C-26 methyl group of **15** (8*S*), but DpfgJ could not accept **15** (8*S*) as a substrate. Thus, DpmaF and DpfgJ strictly recognized the C-8 configuration, while substrate selectivity of DpasF, DpchF, DpmpI, DpfgI and DpmpJ showed tolerant substrate selectivity, which became an advantage for the generation of diversity in combinatorial biosynthesis.

Supplementary Note 16. Construction of *AO-dpasACD-dpchBEGH-dpmaF*, *AO-dpasABCDEF-dpmpGH* and *AO-dpasABCDE-dpchF*

The BBE gene *dpmaF* was introduced in **9** producing transformant, *AO-dpasACD-dpchBEGH*, to provide *AO-dpasACD-dpchBEGH-dpmaF*. Similarly, the FMO gene *dpasF* was introduced in **9** producing transformant, *AO-dpasABCDE-dpmpGH*, to provide *AO-dpasABCDEF-dpmpGH*. The FMO gene, *dpchF* was introduced in **4** producing transformant *,AO-dpasABCDE* to give *AO-dpasABCDE-dpchF*.

Supplementary Note 17. Construction of *AO-dpasABCDE-dpfgI*, *AO-dpasABCDE-dpmpIJ* and *AO-dpasABCDE-dpfgIJK*

The MT1 gene *dpfgI* was introduced in **4** producing transformant *AO-dpasABCDE* to give *AO-doasABCDE-dpmpI*. Similarly, *dpmpIJ* and *dpfgIJK* were introduced in *AO-dpasABCDE* to give *AO-dpasABCDE-dpmpIJ* and *AO-dpasABCDE-dpfgIJK,* respectively.

Supplementary Note 18. Isolation and characterization of 15 and 16

AO-dpasABCDE-dpfgI was cultivated (150 mL x 24) for 1 week (30 ^{o}C. 150 rpm) and the mycelium (24 g dry weight) was extracted with EtOAc (20% MeOH). The extract was subjected to silica gel column chromatography (*n*-Hexane/EtOAc, 4 : 1 to 1 : 2) to give clude **15**. A portion of it was further purified by PTLC (SiO_2, *n*-hexane/EtOAc, 1 : 1) to give **15** (40.0 mg).

AO-dpasABCDE-dpmpIJ was cultivated (150 mL x 20) for 1 week (30 ^{o}C, 150 rpm) and the mycelium (20

g dry weight) was extracted with EtOAc (20% MeOH). The extract was subjected to silica gel column chromatography (*n*-Hexane/EtOAc, 4:1 to EtOAc). A portion of it was further purified by preparative TLC (SiO_2, *n*-hexane/EtOAc, 1 : 4) to give **16** (8.0 mg).

Compound **15 (viridoxin A hydrolysate)**: Colorless oil; $[\alpha]_D^{24}$ −52.2 (*c* 1.30, $CHCl_3$); UV (MeOH) λ_{max} nm (log ε) 259 (3.86); IR (KBr) ν_{max} (cm^{-1}) 3419, 2969, 2938, 2873, 1669, 1588; HRESIMS: *m/z* 443.3153 $[M+H]^+$ (443.3156 calcd for $C_{28}H_{43}O_4$); ^1H and ^{13}C NMR data are shown in Supplementary Table 14.

Compound **16** (FDDP F): Colorless oil; $[\alpha]_D^{24}$ −48.2 (*c* 0.70, $CHCl_3$); UV (MeOH) λ_{max} nm (log ε) 266 (3.83); IR (KBr) ν_{max} (cm^{-1}) 3363, 2933, 2873, 1662, 1579; HRESIMS: *m/z* 459.3098 $[M+H]^+$ (459.3105 calcd for $C_{28}H_{43}O_5$); ^1H and ^{13}C NMR data are shown in Supplementary Table 14.

Supplementary Note 19. Construction of *AO-dpasABCDEF-dpfgGH-dpmpIJ* and *AO-dpasABCDEF-dpmpGH-dpfgIJK*

The three genes *dpasF* (FMO), *dpmpI* (MT1) and *dpmpJ* (P450) were introduced in a **9** producing transformant *AO-dpasABCDE-dpfg-GH* to provide a transformant *(dpmp* path + *dpasF*) (*AO-dpasABCDEF-dpfgGH-dpmpIJ*). Similarly, the four genes *dpasF* (FMO), *dpfgI* (MT1), *dpfgJ* (P450) and *dpfgK* (MT2) were introduced in another **9** producing transformant *AO-dpasABCDE-dpmp-GH* to provide a transformant *(dpfg* path + *dpasF*) (*AO-dpasABCDEF-dpmpGH-dpfgIJK*).

Supplementary Note 20. Isolation and characterization of 17-21

AO-dpasABCDEF-dpfgGH-dpmpIJ was cultivated (150 mL x 24) for 1 week (30 °C, 150 rpm) and the mycelium (28 g dry weight) was extracted with EtOAc (20% MeOH). The culture media was also extracted with EtOAc. The extracts were combined and subjected to silica gel column chromatography ($CHCl_3$/MeOH, 19:1 to 5:1) to give the mixture of **17-19**. Repeated preparative TLC (SiO_2, $CHCl_3$/EtOAc, 1 : 1, ODS, CH_3CN/H_2O, 3 : 1) allowed us to purify **17** (3.4 mg), **18** (3.1 mg), **19** (10.0 mg), and **20** (3.2 mg).

AO-dpasABCDEF-dpmpGH-dpfgIJK was cultivated (150 mL x 30) for 1 week (30 °C, 150 rpm) and the mycelium (43 g dry weight) was extracted with EtOAc (20% MeOH). The extract was subjected to silica gel column chromatography ($CHCl_3$/EtOAc, 9 : 1 to 1 : 9) and flash column chromatography ($CHCl_3$/MeOH, 40 : 1 to 9 :1). Finally, preparative TLC (SiO_2, *n*-hexane/EtOAc, 2 : 3) allowed us to purify **21** (4.3 mg).

Compound **17** (FDDP G): Colorless oil; $[\alpha]_D^{22}$ −68.8 (*c* 0.48, $CHCl_3$); UV (MeOH) λ_{max} nm (log ε) 265 (3.75); IR (KBr) ν_{max} (cm^{-1}) 3377, 2951, 2928, 2870, 1662, 1578; HRESIMS: *m/z* 457.2949 $[M+H]^+$ (457.2949 calcd for $C_{28}H_{41}O_5$); ^1H and ^{13}C NMR data are shown in Supplementary Table 15.

Compound **18** (FDDP H): Colorless oil; $[\alpha]_D^{24}$ −77.6 (*c* 0.16, MeOH); UV (MeOH) λ_{max} nm (log ε) 265

(3.85); IR (KBr) ν_{max} (cm^{-1}) 3367, 2952, 2927, 2867, 1663, 1580; HRESIMS: *m/z* 457.2948 $[M+H]^+$ (457.2949 calcd for $C_{28}H_{41}O_5$); ^1H and ^{13}C NMR data are shown in Supplementary Table 15.

Compound **19** (FDDP I): Colorless oil; $[\alpha]_D^{24}$ −60.8 (*c* 1.10, $CHCl_3$); UV (MeOH) λ_{max} nm (log ε) 244 (4.07); IR (KBr) ν_{max} (cm^{-1}) 3373, 2943, 2872, 1663, 1578; HRESIMS: *m/z* 473.2898 $[M+H]^+$ (473.2898 calcd for $C_{28}H_{41}O_6$); ^1H and ^{13}C NMR data are shown in Supplementary Table 15.

Compound **20** (FDDP J): Colorless oil; $[\alpha]_D^{22}$ −54.3 (*c* 0.27, $CHCl_3$); UV (MeOH) λ_{max} nm (log ε) 266 (3.81); IR (KBr) ν_{max} (cm^{-1}) 3357, 2955, 2927, 2862, 1662, 1579; HRESIMS: *m/z* 457.2949 $[M+H]^+$ (457.2949 calcd for $C_{28}H_{41}O_5$); ^1H and ^{13}C NMR data are shown in Supplementary Table 16.

Compound **21** (FDDP K): Colorless oil; $[\alpha]_D^{24}$ −60.0 (*c* 0.19, MeOH); UV (MeOH) λ_{max} nm (log ε) 264 (3.80); IR (KBr) ν_{max} (cm^{-1}) 3361, 2953, 2928, 2862, 1740, 1661, 1623, 1577; HRESIMS: *m/z* 523.2664 $[M+Na]^+$ (523.2666 calcd for $C_{29}H_{40}O_7Na$); ^1H and ^{13}C NMR data are shown in Supplementary Table 16.

Supplementary Note 21. Construction of *AO-dpasACD-dpchBEFGH-dpmpI* and *AO-dpasABCDEF-dpmpI*

The two genes *dpchF* (FMO) and *dpmpI* (MT1) were introduced in **9** producing transformant, *AO-dpasACD-dpchBEGH*, to give *AO-dpasACD-dpchBEFGH-dpmpI*, while *AO-dpasABCDEF-dpmpI* was constructed by introduction of *dpmpI* (MT1) to whole *dpas* pathway expressed transformant *AO-dpasABCDEF*.

Supplementary Note 22. Isolation and characterization of 22–25

AO-dpasACD-dpchBEFGH-dpmpI was cultivated (150 mL x 16) for 1 week (30 °C, 150 rpm) and the mycelium (19 g dry weight) was extracted with EtOAc (20% MeOH). The extract was subjected to silica gel column chromatography (*n*-Hexane/EtOAc, 4 : 1 to 1 : 2) to give mixture of **22**. A portion of it was further purified by preparative TLC (ODS, CH_3CN/H_2O, 10 : 1) to afford **22** (2.9 mg).

AO-dpasABCDEF-dpmpI was cultivated (150 mL x 24) for 1 week (30 °C, 150 rpm) and the mycelium (23.5 g dry weight) was extracted with EtOAc (20% MeOH). The extract was subjected to silica gel column chromatography (*n*-Hexane/EtOAc, 4 : 1 to 1 : 4) to a mixture of **23** and **24**, and crude **25**. A portion of the mixture of **23** and **24** was further purified by preparative TLC (SiO_2, *n*-hexane/EtOAc, 1 : 1) to afford **23** (6.6 mg), **24** (4.1 mg). A portion of crude **25** was also further purified by preparative TLC (SiO_2, *n*-hexane/EtOAc, 1 : 2) to give **25** (1.7 mg).

Compound **22** (FDDP L): Colorless oil; $[\alpha]_D^{24}$ −72.1 (*c* 0.10, MeOH); UV (MeOH) λ_{max} nm (log ε) 259 (3.87); IR (KBr) ν_{max} (cm^{-1}) 2954, 2926, 2859, 1671, 1600; HRESIMS: *m/z* 441.2995 $[M+H]^+$ (441.2999 calcd for $C_{28}H_{41}O_4$); ^1H and ^{13}C NMR data are shown in Supplementary Table 17.

Compound **23** (FDDP M): Colorless oil; $[\alpha]_D^{23}$ −90.5 (*c* 0.10, $CHCl_3$); UV (MeOH) λ_{max} nm (log ε) 258 (3.88); IR (KBr) ν_{max} (cm^{-1}) 2949, 2926, 2867, 1671, 1601; HRESIMS: *m/z* 441.2998 $[M+H]^+$ (441.2999 calcd for $C_{28}H_{41}O_4$); ^1H and ^{13}C NMR data are shown in Supplementary Table 17.

Compound **24** (FDDP N): Colorless oil; $[\alpha]_D^{24}$ −71.0 (*c* 0.23, MeOH); UV (MeOH) λ_{max} nm (log ε) 259 (3.87); IR (KBr) ν_{max} (cm^{-1}) 2951, 2927, 2867, 1671, 1599; HRESIMS: *m/z* 441.2996 $[M+H]^+$ (441.2999 calcd for $C_{28}H_{41}O_4$); ^1H and ^{13}C NMR data are shown in Supplementary Table 17.

Compound **25** (FDDP O): Colorless oil; $[\alpha]_D^{23}$ −84.1 (*c* 0.11, MeOH); UV (MeOH) λ_{max} nm (log ε) 246 (4.26); IR (KBr) ν_{max} (cm^{-1}) 3410, 2970, 2927, 2871, 2859, 1670, 1589; HRESIMS: *m/z* 479.2770 $[M+Na]^+$ (479.2768 calcd for $C_{28}H_{40}O_5Na$); ^1H and ^{13}C NMR data are shown in Supplementary Table 18.

Supplementary Note 23. Antiproliferative effects across the panel of JFCR39 cancer cell lines and inhibitory activities on mitochondrial respiratory

DDPs, such as metarhizin A and sesquicillin A, have been reported to have potent cytotoxicity against several cancer cell lines[8,9]. Therefore, we evaluated selected new DDPs (**7**, **11-14**, **16**, **17**, **19–24**) for their antiproliferative activities across the panel of 39 human cancer cell lines, JFCR39 (Supplementary Table 21). The mean log GI_{50} concentrations across the JFCR39 panel for compounds **11**, **12**, **14**, **16**, **17**, **20**, **22–24** were less than -5, i.e., 10 μM, suggesting that these compounds had strong antitumour activity, whereas **7**, **13**, **19** and **21** displayed weak growth inhibition. These results suggest that the two structural features, an enone at the C_5 unit in **7** and **19** and the highly modified γ-pyrone moiety observed in **13** and **21**, contributed to the decrease in their cytotoxic effects. Using these data, we performed *COMPARE* analysis of the GI_{50} patterns across the JFCR39 cell lines, or "*fingerprints*", of representative DDPs with those of reference compounds. Antimycin A[10] and Myxothiazol[11], which are known as potent inhibitors of mitochondrial respiratory complex III (ubiquinol-cytochrome *c* oxidoreductase), were highly ranked, suggesting that the DDPs have similar modes of action to these complex III inhibitors (Supplementary Table 22). To correlate the antitumour fingerprints of DDPs with those of the complex III inhibitors, we performed hierarchical clustering analysis of these compounds and visualized the fingerprints of antitumour activities across the panel *via* a heatmap (Supplementary Fig. 44). In fact, the DDPs and the complex III inhibitors displayed similar fingerprints; they exhibited strong antitumour activities in some of the JFCR39 cell lines, *e.g.*, lung cancer cell lines DMS114, NCI-H460 and DMS273. In other words, these cell lines were commonly susceptible to DDPs, antimycin A and myxothiazol.

Previously, sesquicillin (a natural DDP) was reported to inhibit either mitochondrial respiratory complex II (succinate-ubiquinone oxidoreductase) or III[12]. Therefore, we examined the effects of twelve DDPs (**5**, **7**, **11**, **12**, **14**, **16**, **17**, **20–23**, and **25**), which were selected by considering SARs on respiratory enzymes in bovine heart submitochondrial particles (SMPs) using antimycin A as a reference. As summarized in Supplementary Table 20, DDPs with antiproliferative activities (**5**, **11**, **12**, **14**, **16**, **17**, **20**, **22**, and **23**)

significantly inhibited both the NADH oxidase (covering complexes I, III, and IV) and succinate-cytochrome *c* oxidoreductase (covering complexes II and III) activities in SMPs, with comparable inhibitory potencies in terms of IC_{50} values (Supplementary Table 20). As both assay include electron flux through complex III, these results indicate that the cytotoxic DDPs target complex III. Moreover, their inhibitory potencies against complex III (in terms of IC_{50} values) were well correlated with those against cancer cell proliferation (GI_{50} values) (Supplementary Fig. 45), with the exception that **21** exhibited less effective inhibition on cell proliferation than that expected from its potent inhibition against complex III. Notably, **12** exhibited approximately 20-fold greater inhibition of complex III activity and the proliferation of DMS114 cells as its epimer **16**, implying that the *R* configuration of the hydroxy group at C-8 is important for the tight binding to the enzyme, which is also reflected by the difference in their cytotoxic effects (Supplementary Fig. 44). Taken together, these results strongly suggested that the inhibition of cell growth in the susceptible cell lines is largely attributable to the inhibition of mitochondrial complex III.

Supplementary Note 24. Antiproliferative effects across the panel of JFCR39 cancer cell lines

A panel of 39 human cancer cell lines, termed JFCR39, were previously described[13-16]. Cells were grown in RPMI-1640 (FUJIFILM Wako Pure Chemical Corporation, Tokyo, Japan) supplemented with 1 μg mL^{-1} kanamycin and 5% (v/v) fetal bovine serum (Nichirei Biosciences Inc, Tokyo, Japan) and incubated at 37°C in a humidified atmosphere supplemented with 5% CO_2. Drug efficacy was assessed as changes in total cellular protein after 48 hours of drug treatment using a sulforhodamine B assay. Assays were done in duplicate and the GI_{50} was calculated as described previously[13,16]. The antitumor profile across the JFCR39 panel, or fingerprints were then compared to reference database containing ~2000 compounds by COMPARE algorithm[16,17].

Supplementary Note 25. Effects of DDPs on mitochondrial respiratory enzymes

Preparation of bovine heart submitochondrial particles (SMPs): Mitochondria were isolated from bovine heart. SMPs were prepared by the method of Matsuno-Yagi and Hatefi using a sonication medium containing 0.25 M sucrose, 1.0 mM potassium succinate, 1.5 mM ATP, 10 mM $MgCl_2$, 10 mM $MnCl_2$, and 10 mM Tris/HCl (pH 7.4), and stored in a buffer containing 250 mM sucrose and 10 mM Tris-HCl (pH 7.4) at -80 ˚C until used[18].

Measurement of respiratory enzyme activities in SMPs: NADH oxidase activity (covering complexes I, III and IV) in SMPs was followed by the oxidation of NADH with a Shimadzu UV-3000 instrument (340 nm, $\varepsilon = 6.2\ mM^{-1}cm^{-1}$) at 30˚C[19]. The reaction medium (2.5 mL) contained 0.25 M sucrose, 1.0 mM $MgCl_2$, and 50 mM KPi buffer (pH 7.4), and the protein concentration was set to be 30 μg of proteins/mL. The reaction was initiated by the addition of 50 μM NADH after the equilibration of SMP with the tested compounds for 4 min. The averaged control activity was 750 ± 28 nmol NADH/min/mg of proteins. The IC_{50} values (molar concentration needed to reduce the control enzyme activity by half) were calculated by Prism (version 6,

GraphPad, La Jolla, CA) using sigmoid dose response curve fitting. They were shown as mean ± standard error.

Succinate-cytochrome c (cyt. *c*) oxidoreductase activity (covering complexes II and III) was followed by the reduction of cytochrome. c with a Shimadzu UV-3000 instrument (550-540 nm, ε = 21 $mM^{-1}cm^{-1}$) at 30˚C. To improve the accessibility of cytochrome c, SMPs (7.5 mg/mL) were treated with sodium deoxycholate (0.9% (w/v), 60 min, on ice) before dilution with reaction medium[20]. Then, the SMPs (30 µg of proteins mL^{-1}) were incubated with the tested compounds for 4 min in a buffer containing 0.25 M sucrose, 1.0 mM $MgCl_2$, 4 mM KCN, 1.0 µM rotenone, 60 µM cyt. *c* (from horse heart), and 50 mM KPi (pH 7.4). The reaction was initiated by the addition of 5.0 mM sodium succinate. The averaged control activity was 290 ± 12 nmol cyt. *c*/min/mg of proteins.

Supplementary Note 26. Antiproliferative effects on cancer stem cell-like population in MCF-7 cell

Cell culture: MCF-7 cells (American Type Culture Collection) were cultured in Dulbecco's modified Eagle's medium (DMEM; Sigma-Aldrich) supplemented with 10% heat-inactivated fetal bovine serum (FBS; HyClone), 100 U ml^{-1} penicillin and 100 µg ml^{-1} streptomycin (Gibco BRL).

MTS assay: We carried out MTS assay using the CellTiter 96 AQueous cell proliferation assay kit (Promega), according to the manufacturer's instruction. Briefly, MCF-7 cells were plated in triplicate on 96 well culture plate at a density of 5,000 cells $well^{-1}$. After overnight culture, the cells were treated with DDPs or anti-cancer agent (5-FU, doxorubicin). After 72 h, the cells were treated with CellTiter 96 AQueous One Solution Reagent for 1.5 h and the absorbance of 490 nm was measured using iMark microplate reader (Bio-Rad).

Mammosphere-forming assay: A sphere-forming assay was performed as previously described with slight modifications[21]. Briefly, MCF-7 cells were plated as single cells in triplicate on ultra-low attachment 24-well plates (Corning) at a concentration of 5,000 cells $well^{-1}$ in serum-free DMEM supplemented with N_2 supplement (Gibco) and 20 ng/ml basic Fibroblast Growth Factor (R&D Systems) with DPPs or anti-cancer agents (5-FU, doxorubicin). After 7 days, the number of mammospheres was microscopically counted and the percentage of mammosphere-forming cells was determined as mammosphere-forming efficiency (%).

ALDEFLUOR assay: The ALDEFLUOR kit (Stem Cell Technologies) was used to detect CSC population with high ALDH enzyme activity, as previously described[21]. The cells were plated at a density of 1×10^5 cells in 60 mm culture dishes. After overnight culture, the cells were treated with DPPs for 3 days. The cells were suspended at a concentration of 1×10^6 cells ml^{-1} in ALDH assay buffer containing the ALDH substrate BODIPY-aminoacetaldehyde (BAAA, 1 µM) and incubated for 30 min at 37°C. As a negative control, cells were treated with a specific ALDH inhibitor diethylaminobenzaldehyde (DEAB, 15 µM). A FACS Aria II

cell sorter (BD Biosciences) was used to detect the ALDH-positive and ALDH-negative cells.

Statistics: Data are shown as the mean±s.d from three independent replicate experiments. Statistical analysis was performed using EZR ver. 1.4.1 software (Jichi Medical University, Saitama, Japan), which is a graphical user interface for R[22].

P-values were calculated using a two-sided unpaired Student's t-test (Fig. 5a, c, d and S46a). $P < 0.05$ and $p < 0.01$ were considered significant.

Supplementary Note 27. Biological activity against *Drosophila*

Cell culture: Drosophila DL1 and l(2)mbn cells stably expressing the Drosomycin- and Attacin-firefly luciferase reporter ector, respectively, were maintained at 25 °C in Schneider's Drosophila medium (Thermo Fisher SCIENTIFIC) containing 10% (v/v) heat-inactivated FBS, 100 units mL^{-1} penicillin, 100 μg mL^{-1} streptomycin, and 10 μg mL^{-1} blasticidin.

Cytotoxicity assay: Cells (1.5 x 10^5 cells in 100 μL) were inoculated in 96-well plate. 1 μL of compounds were added and incubated at 25 °C for 12 h. Cytotoxicity activity was measured with the CellTiter-Glo Luminescent Cell Viability Assay (Promega) and Spark 10 M plate reader (TECAN). l(2)mbn cells at about 80% confluence were incubated with 20-hydroxyecdysone (Sigma) at final concentration of 1 μM for 48 h before before being used in this assay. DL1 cells were inoculated in 96-wells plate for 12 h prior being added with the compounds[23].

Luciferase assay: Cells (1.5 x 10^5 cells in 100 μL) were inoculated in 96-well plate. 1 μL of compounds were added and incubated at 25 °C for 30 min. Then, l(2)mbn or DL-1 cells were stimulated with 1μL of heat-killed E. coli or Drosophila larval extract[24,25], respectively. Luciferase activity was measured with the ONE-Glo Luciferase Assay System (Promega) and Spark 10 M plate reader (TECAN). l(2)mbn cells at about 80% confluence were incubated with 20-hydroxyecdysone (Sigma) at final concentration of 1 μM for 48 h before before being used in this assay. DL1 cells were inoculated in 96-wells plate for 12 h prior being added with the compounds[23].

Toxicity assay for adult flies: Oregon R wild-type flies were maintained in standard cornmeal-yeast-agar medium vials at 25℃. Adult flies (around 10 days after eclosion) were injected with 70 nL of solvent (1% DMSO in PBS), 1 mM compound **5** in 1% DMSO/PBS, or 1 mM compound **21** in 1% DMSO/PBS by using NANOJECT II (DRUMMOND)[26]. After 1 h of injection, flies that do not show movement or only faint shaking of their legs are counted as unmoving flies.

Supplementary Note 28. Anti-HIV assay

Azidothymidine (AZT), were purchased from Sigma (St. Louis, MO). A laboratory wild type strain, HIV-1LAI, was used. HeLa-CD4-LTR/β-galactosidase (MAGI) cells provided by Dr. J. Overbaugh through the NIH AIDS Research and Reference Reagent Program, Division of AIDS, NIAID[27] were employed for anti-HIV-1 assays as described previously[28,29]. Drug concentrations reducing to 50% of the stained cell number compared with the drug-free control (EC_{50}) were determined by referring to the dose-response curve. Cytotoxic effect of drugs for the MAGI cells were examined with tetrazolium-based colorimetric assay in parallel[28].

Drug susceptibility with counting was examined with multi-nuclear activated beta-galactosidase indicator assay. Briefly, HeLa-CD4-LTR-β-gal cells were plated in flat 96-well culture plates (104 cells well^{-1}). On the following day, the cells were inoculated with HIV-1IIIB (60 blue cell-forming units (BFU) well^{-1} resulting into 60 blue cells after 48 h incubation) and cultured in the presence of purified agents. Forty-eight hours after virus inoculation, the cells were fixed with phosphate-buffered saline (PBS) containing 1% formaldehyde and 0.2% glutaldehide for 5 min, washed with PBS three times, and incubated with 0.4 mg mL^{-1} X-Gal, 4 mM potassium ferricyanide, 4 mM potassium ferrocyanide, and 2 mM of $MgCl_2$ in PBS for 1 h at 37°C. All the X-gal-stained blue cells stained with X-gal were counted in each well. The activity of compounds was determined as the concentration that reduced HIV-1IIIB infection by 50% (EC_{50}).

Supplementary Note 29. Screening of amyloid β42 aggregation inhibitor

Thioflavin-T assay: A fluorescence assay using Th-T (Sigma, St.Louis, MO, USA) was performed as described previously, with a slight modification[30]. In brief, 445 µL of phosphate-buffered saline (PBS: 50 mM sodium phosphate and 100 mM NaCl, pH 7.4) was aliquoted into a 1.5 mL tube, followed by the addition of 5 µL of each test sample, which was dissolved with ethanol (5 mM). Then, a 50 µL solution of Aβ42 (250 µM in 0.1% NH_4OH), synthesized as reported previously[31], was added to the tube, so that the final concentration was 20 µM of each sample and 10 µM of Aβ42, respectively. Alternatively, Aβ42 dissolved in HFIP at 250 µM stood at room temperature for 30 min, as reported previously[32]. After sonication for 5 min, the solution was air-dried and centrifuged for 10 min under reduced pressure to evacuate HFIP completely. The HFIP-treated Aβ42 was dissolved in 0.1% NH_4OH and added to the reaction mixtures, as mentioned above. The resultant reaction mixture was incubated at room temperature for the desired period, and then 2.5 µL was mixed periodically with 250 µL of Th-T (5.0 µM in 5.0 mM Gly-NaOH, pH 8.5). Fluorescence intensity was measured at 430 nm excitation and 485 nm emission using a micro plate reader (Fluoroskan Ascent,Thermo Scientific). Eight points were measured at each time interval.

Transmission Electron Micrographs (TEM): The procedure was previously reported[32] with slight modifications. The aggregates of each Aβ solution (25 µM) in PBS after 24 or 48 h incubation in the absence or presence of E22P-AbD43 (50 µM) were examined under a TEM (JEM-1400, JEOL, Tokyo; Japan). After each Aβ aggregate was centrifuged (4°C, 17,900 g, 10 min), the supernatant was removed from the pellet.

The resultant pellet was gently resuspended in water (100 μL) using a vortex for 1 min just before TEM analysis. The sample suspension (15 μL) was applied to a 400-mesh carbon–coated copper grid (thickness: 20–25 nm; Veco, Eerbeek, Netherlands) and incubated for 5 min before being negatively stained twice with 2% uranyl acetate. Stained samples were subjected to TEM.

LC–MS and LC–MS/MS analysis: Solution containing either 25 μM Aβ42 or 40 μM E22P, M35M_{ox}-Aβ9-35, synthesized previously[33], 250 μM of each sample in 50% CH_3CN/H_2O containing 0.01% NH_4OH at 25 ^oC for 1 h. After being centrifuged at 17,860g for 5 min at 4 ^oC, an aliquot of the solution was subjected to a LC/Q Tof-MS on an Aqcuity BEH C-18 column (2.1 mm i.d. 100 mm, S-0.17 μm; Waters) at 25 ^oC under a gradient of 25–40% CH_3CN / H_2O containing 0.1% formic acid (0.2 mL min^{-1}) in the case of Aβ42, or 20–30% CH_3CN /H_2O containing 0.1% formic acid (0.3 mL min^{-1}) in the case of E22P, M35M_{ox}-Aβ9-35 for 10 min. UV spectra were monitored at 220 nm. The electron spray ionization at the positive mode was performed under the following parameters: capillary voltage, 3.0 kV; cone voltage, 40 V; cone offset 80 V; source temperature, 120 ^oC; desolvation temperature, 450 ^oC; cone gas, 50 L h^{-1}; desolvation gas, 800 L/h. MS/MS experiments were conducted with a collision energy of 38 V. The precursor ion was set at 1069.9 Da, corresponding to the adduct of E22P, M35M_{ox}-Aβ9-35 with each sample as a +3 ion. Data processing was performed using the software MassLynx version 4.1.

Supplementary Note 30. Reaction of lysine derivative and DDPs

Preparation of benzyl ((benzyloxy)carbonyl)-L-lysinate (28): Benzyl ((benzyloxy)carbonyl)-L-lysinate benzenesulfonate was dissolved in aq. ammonia, and extracted with EtOAc two times. The combined organic layer was washed with water, dried over with anhydrous Na_2SO_4, and concentrated *in vacuo*. The residue was used for following reactions as benzyl ((benzyloxy)carbonyl)-L-lysinate (**28**).

Reaction of 13, 21, 14 or 20 with benzyl ((benzyloxy)carbonyl)-L-lysinate (28): A mixture of **13**, **21**, **14** or **20** (0.1 mg, 0.2 μmol) and **28** (0.37 mg, 1.0 μmol) in tetrahydrofuran (50 μL) was stirred at room temperature for 10 hours, respectively. Each of the reaction was monitored by reverse phase HPLC and LC-MS analysis. 1 μL of the reaction mixture was dissolved in 50 μL of MeOH, and 20 μL of this sample was used for HPLC analysis. The same sample was used for LC-MS analysis. The HPLC analysis was performed on COSMOSIL $5C_{18}$ Packed Column (4.6 mm I.D. x 150 mm, Nacalai Tesque) with acetonitrile and water containing 0.01% trifluoroacetic acid (0-1.5 min: 20:80, 1.5- 11.5 min: a linear gradient from 20:80 to 100:0, 11.5-20 min: 100:0) at a flow rate of 1.0 mL min^{-1}. The LC-MS analysis was performed on COSMOSIL $5C_{18}$ Packed Column (4.6 mm I.D. x 150 mm, Nacalai Tesque) with acetonitrile containing 0.1% formic acid and water containing 0.1% formic acid (0-2 min: 20:80, 2-12 min: a linear gradient from 20:80 to 100:0, 12-20 min: 100:0) at a flow rate of 1.0 mL min^{-1} using positive mode electro spray ionization.

Supplementary Note 31. Isolation and characterization of 26 and 27

Compound **13** and **21** were added to **28** under neutral condition (THF), whereas the reaction proceeded slowly. We also performed the reaction in CH_3CN with triethylamine and demonstrated that the reaction was accelerated under basic condition. Compound **14** and **20** could not be reacted with **28** even under basic condition. We adopted the basic condition to isolate the adducts, **26** and **27**. To a solution of **13** (2.8 mg, 5.6 μmol) and **28** (9.3 mg, 17.6 μmol) in CH_3CN (300 μL), 13.8 μL of triethylamine was added at room temperature. After being stirred for 1 hour, the mixture was concentrated *in vacuo*. The crude residue was fractionated by silica gel column chromatography (EtOAc), and subjected to further purification by preparative TLC on silica gel ($CHCl_3$-EtOAc 4:1, developed three times) to afford **26** (1.8 mg) and **27** (2.8 mg) as colorless oil.

Compound **26**: Colorless oil; HRESIMS: *m/z* 863.4455 $[M+Na]^+$ (863.4459 calcd for $C_{49}H_{64}O_{10}N_2Na$); ^1H and ^{13}C NMR data are shown in Supplementary Table 25.

Compound **27**: Colorless oil; HRESIMS: *m/z* 863.4458 $[M+Na]^+$ (863.4459 calcd for $C_{49}H_{64}O_{10}N_2Na$); ^1H and ^{13}C NMR data are shown in Supplementary Table 25.

Supplementary Note 32. Reaction of 13 with methyl (*tert*-butoxycarbonyl)-L-cysteinate, methyl (*tert*-butoxycarbonyl)-L-serinate, or *N*α-(*tert*-butoxycarbonyl)-L-arginine

A mixture of **13** (0.05 mg, 0.1 μmol) and methyl (*tert*-butoxycarbonyl)-L-cysteinate (0.12 mg, 0.5 μmol) or methyl (*tert*-butoxycarbonyl)-L-serinate (0.11 mg, 0.5 μmol) in tetrahydrofuran (50 μL) was stirred at room temperature for 10 hours, respectively. A mixture of **13** (0.015 mg, 0.03 μmol) and *N*α-(*tert*-butoxycarbonyl)-L-arginine (0.04 mg, 0.15 μmol) in EtOH (30 μL) was stirred at room temperature for 10 hours. Each reaction was monitored by reverse phase HPLC and LC-MS analysis. 4 μL of the reaction mixture was dissolved in 50 μL of MeOH, and 20 μL of this sample was used for HPLC analysis. The HPLC analysis was performed on COSMOSIL $5C_{18}$ Packed Column (4.6 mm I.D. x 150 mm, Nacalai Tesque) with acetonitrile and water containing 0.01% trifluoroacetic acid (0-1.5 min: 20:80, 1.5- 11.5 min: a linear gradient from 20:80 to 100:0, 11.5-20 min: 100:0) at a flow rate of 1.0 mL min^{-1}. The same sample was also used for LC-MS analysis. The LC-MS analysis was performed on COSMOSIL $5C_{18}$ Packed Column (4.6 mm I.D. x 150 mm, Nacalai Tesque) with acetonitrile containing 0.1% formic acid and water containing 0.1% formic acid (0-2 min: 20:80, 2-12 min: a linear gradient from 20:80 to 100:0, 12-20 min: 100:0) at a flow rate of 1.0 mL min^{-1} using positive mode electro spray ionization.

Supplementary References

1. Tagami, K., Minami, A., Fujii, R., Liu, C., Tanaka, M., Gomi, K., Dairi, T. & Oikawa, H. Rapid reconstitution of biosynthetic machinery for fugal metabolites in *Aspergillus oryzae*: total biosynthesis of aflatrem. *ChemBioChem* **15,** 2076-2080 (2014).

2. Ugai, T., Minami, A., Fujii, R., Tanaka, M., Oguri, H., Gomi, K. & Oikawa, H. Heterologous expression of highly reducing polyketide synthase involved in betaenone biosynthesis. *Chem. Commun.* **51,** 1878-1881 (2015).

3. Yamada, O., Na Nan, S., Akao, T., Tominaga, M., Watanabe, H., Satoh, T., Enei, H. & Akita, O. *dffA* Gene from *Aspergillus oryzae* encodes L-ornithine N^5-oxygenase and is indispensable for deferriferrichrysin biosynthesis. *J. Biosci. Bioeng.* **95,** 82-88 (2003).

4. Kato, H. *et al*. New natural products isolated from *Metarhizium robertsii* ARSEF 23 by chemical screening and identification of the gene cluster through engineered biosynthesis in *Aspergillus nidulans* A1145. *J. Antibiot.* **69**, 561-566 (2016).

5. Ohtani, I., Kusumi, T., Kashman, Y. & Kakisawa, H. High-field FT NMR application of Mosher's method. The absolute configurations of marine terpenoids. *J. Am. Chem. Soc.* **113,** 4092-4096 (1991).

6. Kim, H. *et al*. Stereoselective synthesis and osteogenic activity of subglutinols A and B. *J. Am. Chem. Soc.* **131**, 3192-3194 (2009).

7. Cimmino, A., Mathiew, V., Masi, M., Baroncelli, R., Boari, A., Pescitelli, G., Ferderin, M., Lisy, R., Evidente, M., Tuzi, A., Zonno, M. C., Kornienko, A., Kiss, R. & Evidente, A. Higginsianins A and B, two diterpenoid α-pyrone produced by *Colletotrichum higginsianum*, with *in vitro* cytostatic activity. *J. Nat. Prod.* **79,** 116-125 (2016).

8. Engel, B., Erkel, G., Anke, T., & Sterner, O. *J. Antibiot.* Sesquicillin, an inhibitor of glucocorticoid mediated signal transduction. **51**, 518-521 (1998).

9. Kikuchi, H. *et al*. New diterpene pyrone-type compounds, metarhizins A and B, isolated from entomopathogenic fungus, *Metarhizium flavoviride* and their inhibitory effects on cellular proliferation. *Tetrahedron* **65**, 469 (2009).

10. Ma, X., Jin, M., Cai, Y., Xia, H., Long, K., Liu, J., Yu, Q., Yuan, J. Mitochondrial electron transport chain complex III is required for antimycin A to inhibit autophagy. *Chem. Biol.* **18**, 1474-1481 (2011).

11. Starkov, A. A. & Fiskum, G. Myxothiazol induces H_2O_2 production from mitochondrial respiratory chain. *Biochem. Biophys. Res. Commun.* **281**, 645-650 (2001).

12. Lai, K.*et al*. Integrated compound profiling screens identify the mitochondrial electron transport chain as the molecular target of the natural products manassantin, sesquicillin, and arctigenin. *ACS Chem. Biol.* **8**, 257-267 (2013).

13. Yamori T. Panel of human cancer cell lines provides valuable database for drug discovery and

bioinformatics. *Cancer Chemother. Pharmacol.* **52**, S74–79 (2003).

14. Dan S, Tsunoda T, Kitahara O, et al. An integrated database of chemosensitivity to 55 anticancer drugs and gene expression profiles of 39 human cancer cell lines. *Cancer Res*. **62**, 1139–1147 (2002).

15. Kitamura, K., Itoh, H., Ssakurai, K., Dan, S. & Inoue, M. Target Identification of Yaku'amide B and its two distinct activities against mitochondrial F0F1-ATP synthase. *J. Am. Chem. Soc.* **140**, 12189-12199 (2018).

16. Shoemaker RH. The NCI60 human tumour cell line anticancer drug screen. *Nat. Rev. Cancer* **6**, 813–23 (2006).

17. Paull KD, Shoemaker RH, Hodes L, et al. Display and analysis of patterns of differential activity of drugs against human tumor cell lines: development of mean graph and COMPARE algorithm. *J. Natl. Cancer Inst.* **81**, 1088–1092 (1989).

18. Matsuno-Yagi, A., and Hatefi, Y. Studies on the mechanism of oxidative phosphorylation. Catalytic site cooperativity in ATP synthesis. *J. Biol. Chem.* **260**, 14424–14427(1985).

19. Uno, S., Kimura, H., Murai, M., and Miyoshi, H. Exploring the quinone/inhibitor-binding pocket in mitochondrial respiratory complex I by chemical biology approaches, *J. Biol. Chem.* **294**, 679-696 (2019).

20. Yabunaka, H., Kenmochi, A., Nakatogawa, Y., Sakamoto, K., and Miyoshi, H. Hybrid ubiquinone: novel inhibitor of mitochondrial complex I, *Biochim. Biophys. Acta* **1556**, 106-112 (2002).

21. Hirata, N., Yamada, S., Shoda, T., Kurihara, M., Sekino, Y. & Kanda, Y. Sphingosine-1-phosphate promotes expansion of cancer stem cells via S1PR3 by a ligand-independent Notch activation. *Nat. Commun*. **5**, 4806 (2014).

22. Kanda, Y. Investigation of the freely available easy-to-use software 'EZR' for medical statistics. Bone Marrow Transplant. 48, 452–458 (2013).

23. Manaka, J., Kuraishi, T., Shiratsuchi, A., Nakai, Y., Higashida, H., Henson, P., & Nakanishi, Y. Draper-mediated and Phosphatidylserine-independent Phagocytosis of Apoptotic Cells by Drosophila Hemocytes/Macrophages. *J. Biol. Chem.* **279**, 48466–48476 (2004).

24. Kanoh, H., Kuraishi, T., Tong, L.-L., Watanabe, R., Nagata, S., & Kurata, S. Ex vivo genome-wide RNAi screening of the Drosophila Toll signaling pathway elicited by a larva-derived tissue extract. *Biochem. Biophysi. Res. Commu.* **467**, 400–406 (2015).

25. Nonaka, S., Kawamura, K., Hori, A., Salim, E., Fukushima, K., Nakanishi, Y., & Kuraishi, T. Characterization of Spz5 as a novel ligand for Drosophila Toll-1 receptor. *Biochem. Biophysi. Res. Commu.* **506**, 510–515 (2018).

26. Hori, A., Kurata, S., & Kuraishi, T. (2017). Unexpected role of the IMD pathway in Drosophila gut defense against Staphylococcus aureus. *Biochem. Biophysi. Res. Commu.* **495**, 395-400 (2018).

27. Chackerian B., Long E. M., Luciw P. A., Overbaugh Human immunodeficiency virus type 1 coreceptors participate in postentry stages in the virus replication cycle and function in simian immunodeficiency virus infection. *J. Virol.* **71**, 3932–3939 (1997).

28. A. Kawamoto, E. Kodama, S.G. Sarafianos, Y. Sakagami, S. Kohgo, K. Kitano, N. Ashida, Y. Iwai, H. Hayakawa, H. Nakata, H. Mitsuya, E. Arnold, M. Matsuoka, 2'-deoxy-4'-C-ethynyl-2-halo-adenosines active

against drug-resistant human immunodeficiency virus type 1 variants, *Int. J. Biochem Cell Biol.* **40** 2410-2420 (2008).

29. Shimura K., Nameki D., Kajiwara K., Watanabe K., Sakagami Y., Oishi S., Fujii N., Matsuoka M., Sarafianos S. G., Kodama E. N. Resistance profiles of novel electrostatically constrained HIV-1 fusion inhibitors. *J. Biol. Chem.* **285** 39471-39480 (2010).

30. Yoshioka, T., Murakami, K., Ido, K., Hanaki, M., Yamaguchi, K., Midorikawa, S., Taniwaki, S., Gunji, H., Irie, K. Semisynthesis and structure-activity studies of uncarinic acid C isolated from uncaria rhynchophylla as a specific inhibitor of the nucleation phase in amyloid β42 aggregation. *J. Nat. Prod.* **79**, 2521-2529 (2016).

31. Sato, M., Murakami, K., Uno, M., Nakagawa, Y., Katayama, S., Akagi, K., Masuda, Y., Takegoshi, K., Irie, K. Site-specific inhibitory mechanism for amyloid β42 aggregation by catechol-type flavonoids targeting the Lys residues. *J. Biol. Chem.* **288**, 23212−23224 (2013).

32. Murakami, K. *et al.*, An RNA aptamer with potent affinity for a toxic dimer of amyloid b42 has potential utility for histochemical studies of Alzheimer's disease. *J. Biol. Chem.* in press, doi: 10.1074/jbc.RA119.010955 (2020).

33. Murakami, K., Horikoshi-Sakuraba, Y., Murata, N., Noda, Y., Masuda, Y., Kinoshita, N., Hatsuta, H., Murayama, S., Shirasawa, T., Shimizu, T., Irie, K. Monoclonal antibody against the turn of the 42-residue amyloid β-protein at positions 22 and 23. *ACS Chem. Neurosci.* **1**, 747-756 (2010).
