# Supplementary material for: Synthetic biology based construction of biological activity-related library of fungal decalin-containing diterpenoid pyrones
Source: Nat Commun. 2020 Apr 14;11:1830. doi: 10.1038/s41467-020-15664-4 (PMC7156458; doi:10.1038/s41467-020-15664-4)
Supplement: Supplementary file 3 — Reporting Summary [file 41467_2020_15664_MOESM3_ESM.pdf]

nature research

Corresponding author(s): Teigo Asai

Last updated by author(s): Mar 17, 2020

Reporting Summary

Nature Research wishes to improve the reproducibility of the work that we publish. This form provides structure for consistency and transparency in reporting. For further information on Nature Research policies, see Authors & Referees and the Editorial Policy Checklist .

Statistics

For all statistical analyses, confirm that the following items are present in the figure legend, table legend, main text, or Methods section.

n/a	Confirmed	
☐	☒	The exact sample size (*n*) for each experimental group/condition, given as a discrete number and unit of measurement
☐	☒	A statement on whether measurements were taken from distinct samples or whether the same sample was measured repeatedly
☐	☒	The statistical test(s) used AND whether they are one- or two-sided<br>*Only common tests should be described solely by name; describe more complex techniques in the Methods section.*
☒	☐	A description of all covariates tested
☐	☒	A description of any assumptions or corrections, such as tests of normality and adjustment for multiple comparisons
☐	☒	A full description of the statistical parameters including central tendency (e.g. means) or other basic estimates (e.g. regression coefficient) AND variation (e.g. standard deviation) or associated estimates of uncertainty (e.g. confidence intervals)
☐	☒	For null hypothesis testing, the test statistic (e.g. *F*, *t*, *r*) with confidence intervals, effect sizes, degrees of freedom and *P* value noted<br>*Give P values as exact values whenever suitable.*
☒	☐	For Bayesian analysis, information on the choice of priors and Markov chain Monte Carlo settings
☒	☐	For hierarchical and complex designs, identification of the appropriate level for tests and full reporting of outcomes
☒	☐	Estimates of effect sizes (e.g. Cohen's *d*, Pearson's *r*), indicating how they were calculated

Our web collection on statistics for biologists contains articles on many of the points above.

Software and code

Policy information about availability of computer code

Data collection	Shimadzu UV-3000, Spark 10 M plate reader (TECAN). (It is described in Materials and Methods section.), Burker AVANCE III spectrometer (500 MHz, NMR), HITACHI LaChrom Elite series equipped with L-2130 pump, L-2200 autosampler, L-2455 Diode Array Detector, HITACHI Chromaster series equipped with 5110 pump, 5430 Diode Array Detector, 5610 MS Detector, Exactive 1.1 SP6 Tune application for Benchtop Orbitrap MS ver. 1.1 SP6 Build 1360 (Thermo Fischer Scientific), JASCO-V-730, JASCO-FT/IR-4200, JASCO-P-1030, TEM (JEM-1400, JEOL, Tokyo; Japan), BD FACSDiva software ver. 6.1.3 was used for flow cytometry data collection. Fluoroskan Ascent (ThermoFisher). Acquity UPLC system H-class with Xevo G2-S (Waters). Benchmark Plus microplate reader/Microplate manager 5.2.1 software (Bio-Rad Laboratories, Inc., Hercules, CA) were used for measuring OD525 of samples stained with sulforhodamine B
Data analysis	Graphpad Prism 6.0, Topspin 3.6.0 (NMR), MSD System manager 2.1 (LC-MS), Spectra Manger 2.08.01 (UV, IR), iRM -1000 (UV), Hitachi Model D-2000 Elite Chromatography Data Station Software HPLC System Manager ver.3.0. Qual Browser Thermo Xcalibur 3.0.63 ver. 3.0 (HR-ESIMS), MassLynx version 4.1., EZR ver. 1.4.1 was used for statistical analysis. Ascent software. MassLynx v4.1. TEM Center for JEM-1400. Cluster 3.0 software (Stanford University, Palo Alto, CA, USA) and Java TreeView software version 1.1.6r4 (http://jtreeview.sourceforge.net/) were used for heatmap analysis of antiproliferative fingerprints of DDP compounds across the JFCR39 cell lines

For manuscripts utilizing custom algorithms or software that are central to the research but not yet described in published literature, software must be made available to editors/reviewers. We strongly encourage code deposition in a community repository (e.g. GitHub). See the Nature Research guidelines for submitting code & software for further information.

Data

Policy information about availability of data

All manuscripts must include a data availability statement. This statement should provide the following information, where applicable:

- Accession codes, unique identifiers, or web links for publicly available datasets
- A list of figures that have associated raw data
- A description of any restrictions on data availability

NCBI non-redundant database, BLAST, Sci finder® , All the data supporting this report is available in maintext and supplementary information.

Field-specific reporting

Please select the one below that is the best fit for your research. If you are not sure, read the appropriate sections before making your selection.

- [x] Life sciences
- [ ] Behavioural & social sciences
- [ ] Ecological, evolutionary & environmental sciences

For a reference copy of the document with all sections, see nature.com/documents/nr-reporting-summary-flat.pdf

Life sciences study design

All studies must disclose on these points even when the disclosure is negative.

Sample size	Sample sizes for Fig. 6 were determined based on previous publications in similar fields (Kanoh, H. et al. Genome-wide RNAi screening implicates the E3 ubiquitin ligase Sherpa in mediating innate immune signaling by Toll in Drosophila adults. Sci. Signal. 8, ra107–ra107.).<br>JFCR39 screening was performed in duplicate, as described in NCI60 five-dose screen (https://dtp.cancer.gov/discovery_development/nci-60/methodology.htm)<br>All biological experiments were performed with n = 3, and they were successfully repeated at least twice.
Data exclusions	No data was excluded from the analysis.
Replication	Biological assays were replicated at least twice. All attempts at replication were successful.
Randomization	All strains for heterologous expression study were randomly picked from the corresponding agar plate.<br>All samples and animals for Drosophila study were randomly picked up.<br>The other experiments are not applicable.
Blinding	No data was blindly corrected in Drosophila because of no room for subjectivity in data collection in this study.

Reporting for specific materials, systems and methods

We require information from authors about some types of materials, experimental systems and methods used in many studies. Here, indicate whether each material, system or method listed is relevant to your study. If you are not sure if a list item applies to your research, read the appropriate section before selecting a response.

Materials & experimental systems

n/a	Involved in the study	
x		Antibodies
	x	Eukaryotic cell lines
x		Palaeontology
	x	Animals and other organisms
x		Human research participants
x		Clinical data

Methods

n/a	Involved in the study	
x		ChIP-seq
	x	Flow cytometry
x		MRI-based neuroimaging

Eukaryotic cell lines

Policy information about cell lines

Cell line source(s)	JFCR39 (MCF-7, NCI-H226, NCI-H522, NCI-H460, A549, DMS273, DMS114, HCC2998, KM-12, HT-29, HCT-14, HCT-116, St-4, MKN1, MKN-B, MKN-A, MKN45, MKN74, HBC-4, BSY-1, HBC-5, MDA-MB-231, OVCAR-3, OVCAR-4, OVCAR-5, OVCAR-8, SK-OV-3, U251, SF-268, SF-295, SF-539, SNB-75, SNB-78, RXF-631L, ACHN, LOX-IMVI, DU-145, PC-3). NCI-H23, NCI-H226, NCI-H522, NCI-H460, A549, DMS273, DMS114, HCC2998, KM-12, HCT-15, HCT,-116, MDA-MB231, OVCAR-3, OVCAR-4, OVCAR-5, OCCAR-8, SK-OV-3, U251, SF-268, SF-295, SF-539, SNB-75, SNB-78, RXF-631L, ACHN, LOX-IMVI were distributed from National Cancer Institute (Frederick, MD). HT-29, MCF-7, DU-145 and PC-3 were purchased from American Type Culture Collection (Baltimore, MD). MKN-1, MKN-74, MKN-45, MKN-A and MKN-B were purchased from Immuno-Biological Laboratories, Co., Ltd., Gumma, Japan). St-4, HBC-4, HBC-5 and BSY-1 were established in Japanese Foundation for Cancer Research (Tokyo, Japan).<br>DL-1 and l(2)mbn were gifted from Dr. Kurata in Tohoku University.<br>MCF-7 (Fig.5 and Supplementary Fig. 46) were obtained from American Type Culture Collection.<br>HeLa-CD4-LTR/β-galactosidase (MAGI) cells provided by Dr. J. Overbaugh through the NIH AIDS Research and Reference Reagent Program.
Authentication	39 human cancer cell lines, JFCR39 (MCF-7, NCI-H226, NCI-H522, NCI-H460, A549, DMS273, DMS114, HCC2998, KM-12, HT-29, HCT-14, HCT-116, St-4, MKN1, MKN-B, MKN-A, MKN45, MKN74, HBC-4, BSY-1, HBC-5, MDA-MB-231, OVCAR-3, OVCAR-4, OVCAR-5, OVCAR-8, SK-OV-3, U251, SF-268, SF-295, SF-539, SNB-75, SNB-78, RXF-631L, ACHN, LOX-IMVI, DU-145, PC-3) were authenticated by STR analysis.<br>DL-1 and l(2)mbn are Drosophila cell lines, and were not authenticated.<br>MCF-7 cells (for Fig. 5 and Supplementary Fig. 46) were authenticated by STR analysis to evaluate antiproliferative activity against CSCs.<br>MAGI cells were authenticated by the NIH.
Mycoplasma contamination	All the cell lines tested negative for mycoplasma contamination.
Commonly misidentified lines<br>(See ICLAC register)	No commonly mis-identified cell lines.

Animals and other organisms

Policy information about studies involving animals; ARRIVE guidelines recommended for reporting animal research

Laboratory animals	Oregon R is a wild-type fruit fly, Drosophila melanogaster. Adult female flies around 10 days after eclosion were used.
Wild animals	*Provide details on animals observed in or captured in the field; report species, sex and age where possible. Describe how animals were caught and transported and what happened to captive animals after the study (if killed, explain why and describe method; if released, say where and when) OR state that the study did not involve wild animals.*
Field-collected samples	No ethical approval is required for using Drosophila melanogseter.
Ethics oversight	*Identify the organization(s) that approved or provided guidance on the study protocol, OR state that no ethical approval or guidance was required and explain why not.*

Note that full information on the approval of the study protocol must also be provided in the manuscript.

Flow Cytometry

Plots

Confirm that:

- [x] The axis labels state the marker and fluorochrome used (e.g. CD4-FITC).
- [x] The axis scales are clearly visible. Include numbers along axes only for bottom left plot of group (a 'group' is an analysis of identical markers).
- [x] All plots are contour plots with outliers or pseudocolor plots.
- [x] A numerical value for number of cells or percentage (with statistics) is provided.

Methodology

Sample preparation	The ALDEFLUOR kit (Stem Cell Technologies) was used to detect CSC populations with high ALDH enzyme activity. The breast cancer cell line MCF-7 were plated on 60 mm culture dishes at a density of 1 × 10^5 cells. After overnight culture, cells were treated with DPPs for 3 days. The cells were suspended at a concentration of 1× 10^6 cells ml-1 in ALDH assay buffer containing the ALDH substrate BODIPY-aminoacetaldehyde (BAAA, 1 μM) and incubated for 30 min at 37°C. As a negative control, cells were treated with diethylaminobenzaldehyde (DEAB, 15 μM), a specific ALDH inhibitor.

Instrument	A FACS Aria II cell sorter (BD Biosciences) was used for data collection.
Software	A BD FACSDiva Software ver. 6.1.3 (BD Biosciences) was used to collect and analyze the ALDH-positive cells.
Cell population abundance	Data was collected at 1 × 10^5 cells. ALDH-positive cells were 1% or lower.
Gating strategy	All samples were analyzed by sequential gating including main population (FSC-A/SSC-A gates), single cells (FSC-H/FSC-W, SSC-H/SSC-W). Negative controls with DEAB were used to establish gates (ALDH-positive and ALDH-negative) at BAA-A/SSC-A plots (Fig. S46b).

☒ Tick this box to confirm that a figure exemplifying the gating strategy is provided in the Supplementary Information.
