# Supplementary material for: Synthetic biology based construction of biological activity-related library of fungal decalin-containing diterpenoid pyrones
Source: Nat Commun. 2020 Apr 14;11:1830. doi: 10.1038/s41467-020-15664-4 (PMC7156458; doi:10.1038/s41467-020-15664-4)
Supplement: Supplementary file 4 — Description of Additional Supplementary Files [file 41467_2020_15664_MOESM4_ESM.docx]

**Description of Additional Supplementary Files**

File name: Supplementary Data 1
Description: The sequences data of *dpasA-F* and Dpas A-F.
