# Supplementary material for: Synthetic biology based construction of biological activity-related library of fungal decalin-containing diterpenoid pyrones
Source: Nat Commun. 2020 Apr 14;11:1830. doi: 10.1038/s41467-020-15664-4 (PMC7156458; doi:10.1038/s41467-020-15664-4)
Supplement: Supplementary file 5 — Supplementary Data 1 [file 41467_2020_15664_MOESM5_ESM.zip › Supplementary Data 1/Supplementary Data 1 (Sequence of dpas cluster).docx]

**Sequence of *dpas* cluster (base and amino acid)*.* Letters in red showed start and stop codons. Underline showed primer region.**

***dpasA***

CGACATCTTACAACTCAACATCGTTCGATGAATCGCTTAATACTCTAATCATTAGCGAATGTATTTCTGTGTGTTTTCTTTTATTGTTTAACAGGTGTCA**ATCATGCCTGCGGAATCAAC**TTCCATGCTGGTGTGCGGCTCGCTCATCGCGAGCCACAACGTAGGCAGTCTCTCGCACCTCCGCTCCAGTCTCGTACATGACCCCAGCTTTGCGGGACTGAGGCAACAACTTACCGAGCTTCCCGACGTCTGGTCGCTCTTGGTCGACCGCGAACCGTCACTGGCAGCTGTCGACGCCGCACCCCTATTCCATTCTTTATCCAGCTGGCTCCAGGGTAACAGTTCCTCCGAGGCCCTTTCACTACCAGAGGGAGCACCCAAGAACATCCTCTACGCCATACTCACAGTCCTCACTCACATACTGGAATATGCGACGTTTTTGGA***CAGGAGTAATCCCACGACAG***CTGGAGACGATGATGCACACAGTAGGAGGCTGGAGGACTTCCAGGATGGAGGCGTCCAGGGACTGTGCATCGGTCTTCTTTCTGGCATCGCCATAGCGTGCTCCAAGAGCAGGGTGGAGCTTGGCAAGAATGCAGCCATCGCGGTGCGACTGGCGATATGCGCTGGCGCGTGTGTTGATCTTGCCGAGCTGCAGTCCGCAGAACCCACAGTCTGTTTGAGTGCGCGTTGGTCACGGCATGAGGAGAGCCAAACCAACAATGATTGTGTCGTTGCGGCCACACTCAAGTACTACCCAGGGGTAAGCAGCCGCCGTCTAGGATTCTCTCGATAAGCAATCATTGCTAACAACTCTGTCGTAACAGGCATACATCAGCGTCCGCTCGGATGTCTGCAGTGCCACCATCACCACTAACAAGGGATCGGTGCCCGCCCTCATCAAGGCACTCGAAGAAAAGGGGGCAGTGGCCAAGAGGATCAATCTTTCTGGTCGCTACCACCACTCAATGTATACGCCCATGTTCGAGAAACTGCTCGACATCTGCGGTTCTCAACCTATATTTCAGTTCCCCCAGACTGCGCGACCACTCGTGCCCCTGAGGCGGAGCGATAGCGGAGAGCTGGTAGCTCAGGACGAGACACCACTCCACGAGATCGCCCTGCGCTGCATCCTGGTCGAGACGGCGGACTGGCACAAGACCATGGTCAAGACCTTAGAAACCATGGCAGCCAAAGCAGCGTCAAAGTCGGCCGGTGCTGAGAGCTTGAAGTTGCAGAAGTTCGTGCTGGGGCCCATGGACTGCGCGCCCAAGCCCGCCTTTGCGCCTCTACCAATCCACACTATCCGACCCGCCGCGGTACCCGAGTCATCCTATTCCTACCCAGACGACGCCATCGCCGTTATAGGTCTGTCGTGCCGGTTCCCCAATGCTGAGACCCCGGCCAAGTTCTGGGAGATGCTCAAATCCAAACAGACATCCACATTACTAGGCCCCGTCGACTCCTTCGACTGCGGCCTCTTCCGCAAGTCCCCGCGCGAAGCCGAGTTCCTGGACCCGCAGCAGCGCCTGGGCCTGCACCTCGCCTACGAGGCGTTAGAGTCGGGCGGCTACTTCCAGCCCTCCGCGAACTCAACGGATAACGTAGGCTGCTACGTAGGCGTCTCGTCGTGCGACTACGAGGCCAACGTCAACTCGCACGACCCCACGGCCTTCTCATACACGGGCACCGCCCGCGCCTTTGTCGGCGGCCGCATCAGCCACTTCTTCGGGCTCACGGGGCCCTCCCTGGCCGTCGACACGGCGTGCTCGTCGTCCGGGGTGGCCATCCACACGGCGTGCCGCGCGATCCGGGCGGGCGAGTGCGCGATGGCGCTCGCGGGCGGCATCAACCTGATGACAGAGGAAGGGCGGGCGCACGCGAACCTGGGCGCGGCGTCATTCCTCAGCTCGACGGGGGAGTGTCGGCCGTTCGACGCCGCGGCCAATGGGTACCGGCGCGGCGAGGGCGGCGGCTTCGTTCTGCTGAAGCGTCTGTCCGCGGCGGTGGCGGATAACGATAAGATCCTCGGTGTTGTGGCGGCATCGGCCGTGAACAACAGTAAGGGGAATAAGTCCATCACCTTGCCCGCATCCGGGTCGCAGAGCGACCTATATGAGCAGGTCCTCCAGGCCGCGGGAATGCAGCCGTCGCAGATTAGCTACGTGGAGGCGCACGGGACCGGCACCACCAAGGGGGACCCCATCGAGTGCGAGAGCATCCGCAAGGTCCTCGGCAAGTCGCAGCGGCCGAACGCGCCTCCGCTCATCTTCGGGTCGGTTAAGGGCAACTTTGGCCATAGCGAGGCCGCCTCGGGCGTGTCGGCGTTCATCAAGACGATCCTGATGCTGCAGCGTGGGCAGATCCCGCCGCAGGCCAACTTCACCGTGCTCAACCCGGCCATCCCCTGCGTCGAGGAGGCAAACATGGAGGTGTCCACGCGGATGCAGTCCTGGGAGACGCCGTTCCGCGCAGCGCTGGTGAATAACTACGGCGCGTCTGGTACCAATGCGGCCATGGTCGTTTGCCAGCCCCCACCGCAGCGGGTGGCGAAGCAGCAGGTGATTGAAGCCCAGTCAACGAGAACTCACAAATACCCCGTCATTATCAGCGCCAACTCCCCAACCAGCATTCGCAAGTACTGCGAGTCAGTCCTCGAGCTGGTGGACACGAAGCAAGCCGCGCTCGGCGAGTCCATCGTCCCCGCCATCGCCTGCAAGCTGGCGCGGTCGCAGAACCATGCGCATGCCTACCGTCGCGTCTTCGCCGCGGGCTCGATCGAGGAGCTGAAGGCAGGCCTTCGAGGCGACGGCCAGGGACGCTCCGCGGCCGCCATCTTCCAAATGCCGCCGGGAAGCGTCGCGAAGAAGCCCGTGGTGCTCGTCTTCGCGGGCCAGACGGGGCGGGAGGTACGCTTGAGCGAGGAGGCCTACCTCGGCTGCGCGCTGCTGCGGCGCCGCCTGGACGCGTGCGACCGCGCTCTGCAGTCGCTGGGTCTTGGCGATTTGATCCCTCGCATATTCCGCGCAGAGCCAATTGATGACTTGGCCTACCTTCACTGCATGCACTTCTCGGTGCAGTATGCGGTGGCCATGTCGTGGATCGACGCGGGACTGCAGGTCAGCGCGCTGGTCGGGCACAGTCTCGGCCAGTTGACGTCGCTGTGCATCAGTGGCGTCCTGAGCTTGCGCGACGCGCTGAAACTCGTCGCTGGTCGCGCGCGACTGATCCAGACCAAATGGGGCCCCGAGAGCGGCTGCATGCTGTCCGTGGACGCCGACGCCGCCACGGTGGAAGCGCTGATCCAGTCGATGCCCGGGGACGACAGGGTGGAGATCGCATGCTACAACTCTTCCGTGCACCACATCCTCGCCGGCACCGAGACGGCCATCGCCGCCTTCGCGGAGATCGCGCACGCCAAGGGCGTCTCCTTCCAGCGCCTCGAGGTGACTCACGGATTCCACTCGCACTTGGTCAACTCCATCCTACCCGAGTACCTAGAGCTCATCGAGGGCCTCACTTTGCGCAAAGCCAAGATCCCTATCGAGGCGTGCTCGTCCTCACAGCAGTGCTGGTCTAAGGTGACCCCGCAGATGATTGCCAACCAGTCACGGCAGTCAGTGTACTGGAGCCAGGCCATCGCTCGGGTCGAGGAGCGCCTCGGCCCCAACTGTGTGTGGCTTGAGGCTGGCTCGAGGGCCGTTGGCGTCACCATGGCCCGTCGCGCTCTAGCGGCTCGTCCAGCGACGATACCCGAGTCAAATTCCTCCTTCCACTCGGCCCGGCTCTACGGTGCTGATTCTCTGGACCATCTCACCCAGACAGCCCTTGACTTGTGGAGGGAAGGGGTGCAAGTCCAGTCCTGGATGTTCCATGGCGCCCAAGCACACTCATACGCCCCTCTGGAGCTGCCGTCTTACTGCTTCGAGAACTCTCATCTCTGGCTTCCTCTGATTGAAAACAGCAAGGGCTCTGATGGGATCAACACGGCAGCAGCAAGACCAGTCCAGTTCGTGTCTCTCTCCGAGCTCTCGGAGCGCGGCAGTGAGCAGGTTGCCAAGTTTGAGATCAACCAAGATAACGAAGAGTACTCGCTCTTCGTCCAAGGACGCACTGTCTTCGGGCAGACTCTGGCGCCCTCGTCCGTCTGGATGGAAGCGGCCTCTAGGGCGCTGGATCTGCTCCCTGACCAATCAGCGGACCGCACACCTGCCGTTGTCCACCAGCTACGCCTGCATGCGCCATTCGGCTTAGACTACCAGCGAAAACTCATTTTAGTGTTACGAAGGAGCAACACGTCTTCCCCCGCATGGGAGTTCACCGTGGAGAGCCAGCTGCTTAAAGACAGCAGCAACAGCAGCGACCTCCACGCTTCCGGCACCGTCGGACGCCCCGCGCGACAAACAGACACCAGACAATACCAGTCGCTGCTCCGCCACCTGCGTGAACGGTGTCAGACCTTGCGGCAGGATCCCGATGCATCAGTCGTCAACGGCGCTTTCATCTCCAAAATGATGGCGCAAGTGGCCGACTACGACAAGAGCTACATGGGTATCCGGTCCATCGCGTGCAAGGATTTCGAGGCCGTGGGCGAGGTCGACATACCTGCCATCGCCGTGGAGAAGTGTGCGACGACGGCGTTCATACCGCCCCTGTTTGATAATCTGCTCATTGTCGGCGAGCTGCACGCTAGCAGTCTCGAGGGGCTGGTTCGAGACAACTTGTACATCTGCAAAAGTATTGACTCTGTCATAACCCACGACGGTCTCCAAGGCCCCTTAGGTTCTGAGAAGAGAGGACCTTGGACTGTTCACTCTAGTCTCGAGAGGGAAAGCGCTAAAGATCTGGTCAGCGACATGATTGTTTTCCGACCAGACCAAAAGGCGCCCGTTCTGTCCATTCTCGGCGCTAGGTTCACTCAAATCTCTACGCGGTCGTTGCGAAGGGCTTTAGAGTCTGTCAACGGCGCTCCTGCAGAGACCTCCAACGAGTTCTCGGCACCAACCACTACCAGGTTTGGACTTCCGAGCGGCATCAACTCCGGGTCCCAAGCGTACTCCAATCCCGGGATGATTGAGTCAGATGAAGGAGTTCGCCCCCGCCTTAACAACCCGATCCGCTCTCATAGTGCATCCGATCTCATCTTAGATAACTGCAGCGACACATCTGCGCTGTCTTCTACGACGTCGCCTTCGTCGGTCGGCATTGCGACGCCAGAGGACGAGGAGAACGTGAGAATCCTCACGAATCTGCTTTCCGATCACCTAAACTGCAGTCAAGGCATCCCGCCCGACACACCCTTGGTCATGCTGGGCCTCGACTCATTGGTTATGATGCAGCTCAAATCCGACATCAAGAAGGCGTTCGGCAGTCACATGAATGTGAGCAAGATCGACGAGAACTGTACTCTCTCCGATCTTTGCAGCATGCTATTCCCGAACGAGCCTACAACCCAGCTTCTAAGCAGCACCACCATCACCGAAAAGAAGGCTGTCTTATCACAATCGGCGTCAAAGTATGAGGAGAACCCCATGCCTCTTATGCGAGCGCTGGGTACCACGTCTCACACCCGTAGCGCCTTCATCGAACGCGCCGCGCAGGAGTTCGCCACCCTAAAGCAATCCACGAGCGCCGTCACGCGCGAGACCCAATTCGCCAACTTCTTCGCAGAAGTGTACCCGGACCAGCAGCGCCTGGTAACGACCTACATCCTCGAGGCCTTCAGCAAGCTCGGCTGCGACCTGCGCAACATGCAGGCGGGCGAGATCCTGCCCCCGATCGCGTACCTGCCCAAGTACGAAAAGCTCATGTCCCGGTTCTACGCCATATTGGAGGCCGCGGGCATCATCAGCGCGTATAATTGCCAGAAGCTGCGCTTCAAGACGATCAACAATGGCGGCGGAGACAAAGCCTCGTCCGTAGACCTGTACCGTGACATTCTCGCCAAACACCCCTATTACCATCCGGACCACAAGCTTCTGGCGGTCACGGGACCCTATTTGGCAGAGTGTCTTTCCGGGCAGGATGACGGACTCCAGCTCATCTTCCAGGAGGCCGAGTCGCGCAAGCTGTTGGAGGACGTGTACCGCAGCTCGCCCATGTTCGCCACCGGCAACGCGCTCCTGGGTCACTTCATGACCCAGCTCCTGGAGCAGCAAGCCACCTTCGCCGACGCCGCCGACGATGTTCTCCGCATCCTCGAGATCGGCGGTGGCACCGGCGGCACCGCATACCTCATGATGGACCTGCTGCTTGCCCAACCTAATGTCAAGTTTCACTACACCTTCACCGACATCTCCGCCGCGCTGGTCGCCAGCACGCGCAAGCAGTTCGAGGCCCGCTACGGCCGCTCCTGCCTCGAGAAGCACATGGAGTTCACCGTCCTCGACGCCGAGCGCCCCCCTCCGACGGAGCGGGTCGGGGTCTACCACATGGTGGTGAGCTCGAACTGCATCCACGCGACGCGGGACCTGCGGCAGTCGTGCGGGCTGATCGAGCGGCTGCTGCGGCCGGACGGCGGGGTGTTGTGCCTGCTGGAGCTGACGCGGCCGCTGCCCTGGCTGGACTGCGTGTTCGGGCTGCTGGACGGCTGGTGGCGGTTCGACGATGGAAGATCGTACGCGCTGCAGGATGAGGGACACTGGAAGAAGGCGCTGCTGGAATCGGGTTTCGGTAGAGTGGATTGGAGCGATGATGGGACGCGGGAGTCGCAGCAGTTCCGGTTGATTACGGCGTGGCGC**TGA**AGATGGAATGAACAAACCAAAAGGGGCAACGGCCGGCAATGTTGGATGAGATTTAATTGGCGCTTTACTGTTGTATATAGTTTCCTATGCTTTTCCTTTTCATTTCTCATTCCTGTAGCACGAATACCAATTTCCTTTAGATAGCGACGCACATAACTTGAAGCATTACAACACATATTTCAGCAAACATTTATTCGAAAGTCGATGACTTACTAAAGAATTGGAACGTGAGTTTAATACTCTTCATGGTAGTTGCTAACTGATCTGGACACTTGCACCCTACACACCCTTTGATACCTCCCAATATGGTCCTAGCCGAAGGTCAACAACACTCAATGCTCAGTGAATCAAT***GACTGCTCATAGTCTCGCAT***ACCTTCCCCACGACGACACACCTCGAAGTTGGTCATCTCCATGAAGAGTCATCAGCAGTCGCGGCTGTATCTTGTTTCTTTACTCTAGTGTTATCTCCGC

**DpasA**

MPAESTSMLVCGSLIASHNVGSLSHLRSSLVHDPSFAGLRQQLTELPDVWSLLVDREPSLAAVDAAPLFHSLSSWLQGNSSSEALSLPEGAPKNILYAILTVLTHILEYATFLDRSNPTTAGDDDAHSRRLEDFQDGGVQGLCIGLLSGIAIACSKSRVELGKNAAIAVRLAICAGACVDLAELQSAEPTVCLSARWSRHEESQTNNDCVVAATLKYYPGAYISVRSDVCSATITTNKGSVPALIKALEEKGAVAKRINLSGRYHHSMYTPMFEKLLDICGSQPIFQFPQTARPLVPLRRSDSGELVAQDETPLHEIALRCILVETADWHKTMVKTLETMAAKAASKSAGAESLKLQKFVLGPMDCAPKPAFAPLPIHTIRPAAVPESSYSYPDDAIAVIGLSCRFPNAETPAKFWEMLKSKQTSTLLGPVDSFDCGLFRKSPREAEFLDPQQRLGLHLAYEALESGGYFQPSANSTDNVGCYVGVSSCDYEANVNSHDPTAFSYTGTARAFVGGRISHFFGLTGPSLAVDTACSSSGVAIHTACRAIRAGECAMALAGGINLMTEEGRAHANLGAASFLSSTGECRPFDAAANGYRRGEGGGFVLLKRLSAAVADNDKILGVVAASAVNNSKGNKSITLPASGSQSDLYEQVLQAAGMQPSQISYVEAHGTGTTKGDPIECESIRKVLGKSQRPNAPPLIFGSVKGNFGHSEAASGVSAFIKTILMLQRGQIPPQANFTVLNPAIPCVEEANMEVSTRMQSWETPFRAALVNNYGASGTNAAMVVCQPPPQRVAKQQVIEAQSTRTHKYPVIISANSPTSIRKYCESVLELVDTKQAALGESIVPAIACKLARSQNHAHAYRRVFAAGSIEELKAGLRGDGQGRSAAAIFQMPPGSVAKKPVVLVFAGQTGREVRLSEEAYLGCALLRRRLDACDRALQSLGLGDLIPRIFRAEPIDDLAYLHCMHFSVQYAVAMSWIDAGLQVSALVGHSLGQLTSLCISGVLSLRDALKLVAGRARLIQTKWGPESGCMLSVDADAATVEALIQSMPGDDRVEIACYNSSVHHILAGTETAIAAFAEIAHAKGVSFQRLEVTHGFHSHLVNSILPEYLELIEGLTLRKAKIPIEACSSSQQCWSKVTPQMIANQSRQSVYWSQAIARVEERLGPNCVWLEAGSRAVGVTMARRALAARPATIPESNSSFHSARLYGADSLDHLTQTALDLWREGVQVQSWMFHGAQAHSYAPLELPSYCFENSHLWLPLIENSKGSDGINTAAARPVQFVSLSELSERGSEQVAKFEINQDNEEYSLFVQGRTVFGQTLAPSSVWMEAASRALDLLPDQSADRTPAVVHQLRLHAPFGLDYQRKLILVLRRSNTSSPAWEFTVESQLLKDSSNSSDLHASGTVGRPARQTDTRQYQSLLRHLRERCQTLRQDPDASVVNGAFISKMMAQVADYDKSYMGIRSIACKDFEAVGEVDIPAIAVEKCATTAFIPPLFDNLLIVGELHASSLEGLVRDNFLERESAKDLVSDMIVFRPDQKAPVLSILGARFTQISTRSLRRALESVNGAPAETSNEFSAPTTTRFGLPSGINSGSQAYSNPGMIESDEGVRPRLNNPIRSHSASDLILDNCSDTSALSSTTSPSSVGIATPEDEENVRILTNLLSDHLNCSQGIPPDTPLVMLGLDSLVMMQLKSDIKKAFGSHMNVSKIDENCTLSDLCSMLFPNEPTTQLLSSTTITEKKAVLSQSASKYEENPMPLMRALGTTSHTRSAFIERAAQEFATLKQSTSAVTRETQFANFFAEVYPDQQRLVTTYILEAFSKLGCDLRNMQAGEILPPIAYLPKYEKLMSRFYAILEAAGIISAYNCQKLRFKTINNGGGDKASSVDLYRDILAKHPYYHPDHKLLAVTGPYLAECLSGQDDGLQLIFQEAESRKLLEDVYRSSPMFATGNALLGHFMTQLLEQQATFADAADDVLRILEIGGGTGGTAYLMMDLLLAQPNVKFHYTFTDISAALVASTRKQFEARYGRSCLEKHMEFTVLDAERPPPTERVGVYHMVVSSNCIHATRDLRQSCGLIERLLRPDGGVLCLLELTRPLPWLDCVFGLLDGWWRFDDGRSYALQDEGHWKKALLESGFGRVDWSDDGTRESQQFRLITAWR

***dpasB***

GCCCAGCCAAACGTCATCTGCCGATCCCCCAAGACTGGGTGTGCACACTTCCAAACCCCCTCACTCGAGCACACACACACACATCCAAACGAAGATTAGC**AACCATGGACGTCCACGACC**TGACCCGCGCCCCTCCAGAATACCTCGAGGTAGTCTGGGTGACCGACGTGTGCAAGCTCGTCATGGCCGTGGGCTGGCTCTCCAACTACATCGGCATGATCGCGAAGTCGATCAAGGAGCAGACGTACAGCATGGCGCTGATGCCGCTGTGCTGCAACTTCGCGTGGGAGTTCACGTACTTCTTCATCTACCCGTACAAGGTGCCCATGGAGCGCAACATCCACACGCTGGCCTTCCTGCTCAACTGCGGCGTCATGTACACGGCCGTCCGCTACGGCGCCCGCGAGTGGGGCCACGCGCCGCTCGTCCAGCGCAACTTGCCCGTGATCTTTGTGGTGTGCATCGCGTGCTGGGTCTCGGCGCACGTCGCGTTCGCGGAGCAGTACGGGCCCAGCCTCGCGCAGGCTGTGAGCGGGTTTGCTTGTCAGATCTTGCTGAGTGCTGGAGGTACTTGCCAGCTGCTTTGCCGTGGGCACTCGCGTGGCGCCTCGTATAAGTTATGGTATGCTTTTCCCCTCCTGTCTCCTCCCTATTCTTATCATGCAACTTACATGATTACTAACTTGTGTCTCTCCATCTAGGCTTGCTCGCTTCATGGGCTCCTTTGCTCTGATCCTACCTAACATGCTTCGCTACAAGTACTGGAGGGATGATCATCAGTACATCGGTTCACCTCTCTATATTTGGTTCCTTGGCATGTTCCTGTTCCTGGACGGCTCCTATGGGTTCGTACTTTGGTACGTGCGCCGTCACGAACGGGAACAGGTGTTGGTTGCCAAGCCCAAGGTCCAG**TAA**ACCAAGCCGCTTAGTGCCGGACTCTATTATTGTGAGTCTTGAGAAAAGCAAGTAGGCTGTTGTAGCTCTCATCGACGCAATAAATGGATGATAGAGTTGGTGAGATAAAGAATGGGGAAGTTAGATCACTACATGGAGCTCCTGTGTCAGCTTATCTTTATGCGTGTTTCCAAAGAAAATAATAATCAATAAACCCAC***CGCATATCTATAGCCAAAGT***TGAAGGTGAGAATCTAAATGGTCATGATTTTATAAGTAATCTATCGCTCGTCTCAGCCCAGTGAGGTCCCACACTTCGCAGACTTAATCGTGACGGAATA

**DpasB**

MDVHDLTRAPPEYLEVVWVTDVCKLVMAVGWLSNYIGMIAKSIKEQTYSMALMPLCCNFAWEFTYFFIYPYKVPMERNIHTLAFLLNCGVMYTAVRYGAREWGHAPLVQRNLPVIFVVCIACWVSAHVAFAEQYGPSLAQAVSGFACQILLSAGGTCQLLCRGHSRGASYKLWLARFMGSFALILPNMLRYKYWRDDHQYIGSPLYIWFLGMFLFLDGSYGFVLWYVRRHEREQVLVAKPKVQ

***dpasC***

ATCCCATCTTGGCTATTCATCCCGTCTCTCAAGACAACTTGCAGTTACACCCTCTTTTCACGCAGTCCCCTCGCTCAGGAGCGTAGAGACAGAAACAGCC**ATGGCATCCGTAGCAAAAGC**AAGGTCGCCCAAGAGCACTCAGAGCAACAGCTTGGTCTATGATCTCCTTGTTCTCTCCCGCTTCACCAAGTACAATCCCCTCTTCACAATATTCGCTGGCGGTATGTTTCTCTTCGTTGCTTCCGTCATTCTCAAAATTTTATCGTCTGTGTCACATCAAATTTGAGCGATGACTAACATATAGTTTCCAACCACAGCTTTCTCTTGCTTGCTTGCCGGTTCAACGCTGGTTGGCAATGGCTCTGATGTGACTCTCACTTGGGTCTTCCGCCAGACGGCGCTCTGCCTGTCGGCCTGCTACTCCTTCTGCGGCGCCGGAATGGTGTGGAACGACTGGATCGACCGCGACATCGATGCCAACGTCGCCCGCACCAAGGACCGGCCTCTGGCATCGGGCAGAGTCACTACGACCCAGGCTATGTTGTGGATGGTCTTCCAGATGGCCGTGTCCTGGTGGCTGCTGCACTTCATGTTGGATGGCAAAGATGTGTAAGTATTCTCCACGAAACCCCTCGATGATTCTCTCTATTGGTACTGGAAACTAACACGCATGACTCATCTCCTTAGGAACAACCACATGCTGCCCGTAGTCATCGGCTCCTTCCTCTATCCCTTCGGCAAGCGTCCCATCTGCAGCAAGTTCTACTTCTACCCCCAGTACATCTTGGGCTTCACCATCGCCTGGCCCGCCGTGCCCGGCCGCACCGCTATCTTTCACGGCCAGGAGACCTTTGCCGAGTCGGTCCAGGCTTGCATGCCGCTGCTCAACATGGTCTTCTTCTGGACCATCTTCCTCAACACGGCGTACAGCTACCAGGACGTTGTTGACGACCGCAAGATGGGCGTCAACTCGTTCTACAACGTGTTGGGCAAGCATGTTCACCTGCTGTTGTGCTTGTTGCTCGTCCCCGTCGCCGTCTGCGTCCCCATGTACCTCAACCAGTTCCACTCGACCTGGCTCTGGGTCAGCTGGGCTGGCGTCTGGGCCCTTAGCTTGTTACGCCAGATCACTCGGTTCGATGAGAAGAACCCTGCTAGTGGTGGCAGTCTGCATGTTGATAACTTCCTTCTTGGGGCTTGGACTGTGGTTGCCTGTACTATTGAGTTGCTTATGCGTTATTACTCT**TAG**GTCTTGACTTAGAAGAGAGATTTGGGTGTAGTAAGAAGACTCTTTATGATACGGATTTTATTATCATATGGCTTATGGATCAAGTCTTAGTGAATTTCCAGAGGATCTAATGATCTCCTCCAACTTAGATCATAGCTACATTCCAATCCATACACATTTATATTATCATC***GGTATTCTGTCAAACTGCAT***TTTCTTGTCATTATCTGTATCTTTCATGCTTGGAACCTGTGCCTCTGTCCTTCTAACTATGAATACTCGATATGATACTTGAGAGATGTAAGATAAATAC

**DpasC**

MASVAKARSPKSTQSNSLVYDLLVLSRFTKYNPLFTIFAGGMFLFVASFPTTAFSCLLAGSTLVGNGSDVTLTWVFRQTALCLSACYSFCGAGMVWNDWIDRDIDANVARTKDRPLASGRVTTTQAMLWMVFQMAVSWWLLHFMLDGKDVNNHMLPVVIGSFLYPFGKRPICSKFYFYPQYILGFTIAWPAVPGRTAIFHGQETFAESVQACMPLLNMVFFWTIFLNTAYSYQDVVDDRKMGVNSFYNVLGKHVHLLLCLLLVPVAVCVPMYLNQFHSTWLWVSWAGVWALSLLRQITRFDEKNPASGGSLHVDNFLLGAWTVVACTIELLMRYYS

***dpasD***

GCTGAATGAAGTCTTGGAAAAACCGAACATCTCCGTACATTGACTTTACAACGAATCACTGGGTGGAGACTTCAATTTATTGCCGGATATTGCAGGCATC**ATGACGAACAGCACATTACC**GCCGGCGCAGCTACACCACCTGCCGGCCTCCAGCATCAAGTCCGGTGCGGTGAATGGAGCAGCGGCAGACCCAGTAGGCACGAATGAGAAGGTCGGTTTCCGTTTTCCTGCTCTATCGAGAGTCACGTTGACACGATTCTGATATGCGAGTTTCTGCAGATCCTGAGGGCACCCATCGACTATCTCCTCACAATCCCGGGGAAGGATGTGAGGGGGAAGATGATGAATGCCTTCAACCAGTGGCTCCAAATCCCTGAAGAGAAGCTCGACATCATCAAGGAGGTGATTAAGCTGCTGCATACTGCCTCTCTCCTGTAAGTCATCCTGGTGTGAGTCGAAATTCAAGGGGGGCCATTACTCATGTGGACTTACTAGCATCGATGATATCCAAGATAACTCCAGGTTACGTCGCGGTCTACCCGTCGCACATAGTATTTTCGGTGTTGCTCAGACGATCAACACTGCCAATTACGCCTACTTCCTGGCCCAGCAGGAGCTGAACAAGCTGGACTGCGCCGCCGCGTATGAGGTCTTCACCGAGGAGCTTCTGCGTCTGCACCAGGGACAGGGCATGGACATCTACTGGCGAGACTCGTCCCTCTGCCCGACCGAGGAGGAGTACTTCGAGATGGTCGGCAACAAGACCGGCGGTCTCTTCCGGCTGGCAGTTCGCCTGATGCAGCTGGCAAGCAACAAGGAGTAAGTCATCCGCATGCCGGCGTATCGTCAGACGTCGTTTGCTAACCTGGGAACCACCCACACACAGCTGTGACTTCGTCCCCTTCGTCAACGTCCTCGGCATCCTCTTCCAGATCCGGGACGACTACCTGAACCTGCAGAGCGACCTGTATACCAAGAACAAGGGCTTCGGCGAGGACCTGACCGAGGGCAAGTTCTCATTCCCCATTATCCACAGCATCCGCGCGGACCCGGCCAGCATCACCTTGACCAGCATCCTCAAGCAGCGCACCGAGGACGAGGATGTGAAGCGGTATGCCATCAGCTACATCGAGTCGACAGGCTCGTTCGAGCACTGCCGCCGCAAGATCGACGAGCTCGTCGGCGAGGCCCGTATGTGTGTGAAGGATATGAGCCCCGAGGACGCCAAGGTGGCGGATGGGATTATGGCGATGGTAGGGTTGGGGGCTGGGGGACTGTCGATA**TAG**GGTTTCTATATATAGTTGCAACCATTGGGATGTGTATATTTATTCAGTTTTTGAGATTAGGGAGTTTTCATCATGGTTATAGGCCATTATCCACCATCGTTGCTATCACCCTATGTCCAAACTGAATGCCAGTTTGAAGGCGGCCATGAGGAACCTTGTGTTACGGCGCCAACCATGGCTCTAGTCAAC*TAA*GCCAGCCTGTCGTCCCCAACCGAACAAAAGGACGACATTGTCGAGTCAGCGTCTT***GACTGTCTACTAAAGCTTGT***CACCAACCCTAATACGGTTGCAGGGTGGTTTGGTATTCGAATGGATCCCAAAAAGGACAAGAGAGAGATTTTCTTCGACTATGTAATGTTTACCTTGGTG

**DpasD**

MTNSTLPPAQLHHLPASSIKSGAVNGAAADPVGTNEKFLQILRAPIDYLLTIPGKDVRGKMMNAFNQWLQIPEEKLDIIKEVIKLLHTASLLIDDIQDNSRLRRGLPVAHSIFGVAQTINTANYAYFLAQQELNKLDCAAAYEVFTEELLRLHQGQGMDIYWRDSSLCPTEEEYFEMVGNKTGGLFRLAVRLMQLASNKDCDFVPFVNVLGILFQIRDDYLNLQSDLYTKNKGFGEDLTEGKFSFPIIHSIRADPASITLTSILKQRTEDEDVKRYAISYIESTGSFEHCRRKIDELVGEARMCVKDMSPEDAKVADGIMAMVGLGAGGLSI

***dpasE***

GTTCCGCGATAGCACTCTTTGTTGTCTGCATTTATCACTATCGTGATTTCTTGGCCTTGATAGACCACCATATTTCTATCACTTGGGATTATTAGAGACC**ATCAATATGTCGCAACCAGC**ATTCAAAATCATAATCGTGGGGTGCTCCGTCACCGGGCTCACCTTGGCGCACTGCCTGGACAAGCTTGGGGTCGAGTACACCATCCTGGAGAAGCGCTCCGCGGTGGTACTCCAGGAGGGCGCCTCGGTCGCCGTCATGCCCAACGGCGGCCGTATCTTGGATCAGCTTGGCCTCTACGACGCCTTCGAGAAGGCCACCGTGCCGCTCGACCTCACAGACGCCTACCTGCCCGACCAAGACTTCCGGTTTACCAGCGACTACCCCAGGAGGGTGCTAGCCACGTACGCCAAGCCACGGACCCTTGCATAACAAGAATGATAAATGACTAACAACATGACACAGGTTTGGATACCCCGTTGCCTTTATGGAAAGGAGAGGACTTCTCGAAATCCTCTATGATGGTATCGCGGACAAATCTAAGATTCACCTCAACAAGGGCGTCACGCATGTCGAGCAGAATGACGACGGCGCCAAGGTGCACACGGAGGATGGTGAGGTCTACGAAGGCGACATCGTCGTCGGCGCCGACGGAATCCACAGCAAGACACTACGCGAGATGTGGCGGATGATGGGCGAGCCCGTGGTAAACGGCATTGCTCAGAGCGAAAGCCAAAGTGAGCACGACCCAAACTTTATTCTGACCGGCGTCCTTGACAGTTATGACTGACACTCCGAACCCTGGTTAGACATGTCGGTAGCCTTCTCCTGCGTCTTCGGCATCTCCCACGACGTGCCCGAGCTGCAGCCCGGCGAGCAGATCCTGCGCATGTGCAACGGCTCGACCATATTCGTCATGGGCAGCAAGGGCGTCGTCTTCTGGTTCATCGTCACGCAACTGAACCGGCGTTACGAGTACCATGATGCGCCACGGTACACGACCGAGGAGGCCGCCGCGTTCTGTGAGGCGCGGAAGGACGCCGAGATCAAGGAGGGGGTCACGTTCGAGTGCATCTGGCGGAAGCAACACGTCTTTAACATGTTGCCACTGCAGGAGAGCTTGTTCCAAACCTGGTCGCATGGTCGGGTGGTCTGCATTGGTGACAGTGTTCACAAGGTAAGTCGTTATCGCAGCCCTTAAGTGTGAAGTCACCACGCTGACTTTTGACTTTCCAAGATGACGATCAATCTCGGCCAGGGCGCCAACTGCGCCATCGAAGACGTGACGGTTCTCTGTAACATGCTGCGCGCCTTCCTCGCCGAGAAGAGGGAGAAGAAGCCCTCATACAGCGAGATCGATACCCTGCTGCGGCGGTTCAACAAGGAGCATCTCCCACGCGCCTCCACCATCGTCGAGACGTCGCGCCTGACGACCCGGGTGCACGCGCAAGTCGGTATCAGTCAGCGCATCATGACGCGGTGGGTCGTGCCGTATTTTGGCAAGTTCCTGCAGGGAAAGCCCCTGGGCCTGATCGCCAGCGGCCCGGTTCTGGACTTCTTGCCGTTGAAGCGCGCCTCGTACCCCGGCTGGGAACGGTACAGGGTTAAGAAGAGTAGCAGAGGTGCTGGATTCTGGATCACGGCTTTCTTATCATTGTCTTTGTTGGCTGTTGCCGCTACGATGTACGGGTGGGGCAACTCCCAGATCTGGGCCGATTGGAATATCCTA**TAG**ATAGATCCAAAGGCCCGATGGGAAAAAAAGAATAACTATAATCTTGTATATAATTAGACTTTCCTTATCGATCTCTGCCACGATTCAAGACACGCATATGACCTGATCACTGGTGTGATGTTTACTTCAGACATACCCCAATGTACTGCACAGGGCCCATTGATAAATATT***CCGTCACGATTAAGTCTGCG***AAGTGTGGGACCTCACTGGGCTGAGACGAGCGATAGATTACTTATAAAATCATGACCATTTAGATTCTCACCTTCAACTTTGGCTATAGATATGCGGTGG

**DpasE**

MSQPAFKIIIVGCSVTGLTLAHCLDKLGVEYTILEKRSAVVLQEGASVAVMPNGGRILDQLGLYDAFEKATVPLDLTDAYLPDQDFRFTSDYPRRVLATFGYPVAFMERRGLLEILYDGIADKSKIHLNKGVTHVEQNDDGAKVHTEDGEVYEGDIVVGADGIHSKTLREMWRMMGEPVVNGIAQSESQNMSVAFSCVFGISHDVPELQPGEQILRMCNGSTIFVMGSKGVVFWFIVTQLNRRYEYHDAPRYTTEEAAAFCEARKDAEIKEGVTFECIWRKQHVFNMLPLQESLFQTWSHGRVVCIGDSVHKMTINLGQGANCAIEDVTVLCNMLRAFLAEKREKKPSYSEIDTLLRRFNKEHLPRASTIVETSRLTTRVHAQVGISQRIMTRWVVPYFGKFLQGKPLGLIASGPVLDFLPLKRASYPGWERYRVKKSSRGAGFWITAFLSLSLLAVAATMYGWGNSQIWADWNIL

***dpasF***

TCGGCCGTTCATGGATGTTTTTCTTCCCCAGGTTTGACTCCGTCCTGTTGTCAGCCAGTCTCTCGTTTTTTTTTGTCGATATAACGACAAAAATAATATC**AAGATGAACAGACTCCTCGC**CTCCGCTCTGCTCGTCGGCTCCGCCGTCGTGGCCCCGGTCTCTGCCGCGTGCATCAAGAACGCCACCGTGACCGAAGTCGATGTCGCCATCATCGGCGGTGGCGCCTCAGGCGTCTACGCCGCCGCCCGCTTGATCGACAACAACAAGACCGTGGTCGTGCTCGAGCGGAACGCCGACCGCATCGGTGGGCAGACCGAGACCTACTACGATCCGACCACGGGCACGCCCGTCAACGTCGGCGTCAAGGTCTTCTCTAACACGACGGTGACGACGGACTTCCTCAAGCGCTTCGACTTCCCCATGGGCACCCTCAACGTCGGCCAGACCCTGGCCACGGGCACCCAGTACGTCGACTTCGCCACCGGCAAGCTCATCCCCAACTTCACCGCGGTGGTCCCGGCGGTCCAGGCCGCGGCCATGCAGCGCTACGCCGCCGAGCTGGCCAAGTACCCCCAGATCAAGTTCGGGTACAACCTGGGCCCACAAGTGCCCGAGGACCTGGTCCTACCGTTCGGCAAGTTCATGGAGAAGCACAACCTCACGGGAATGGCCCAGACCATGTTCGAGTTCAACTCGGGCTACACGCCGCTCCTCGAGATCCCGGCACTGTACATCCTCAAGTACCTCGACACGTACGAGCTGCAGTCCCTGCAGTCCGGTAGTTTCATCGTCGCGGCCAACGGCGACTCTGCCACTCTGTACCGCAACGCCGCCAAGTTCCTGGGCGAGCGCGTCGTCTACGGTGTCAGCGGCATGCACATCCAGCGCTCCTCCGCTGCTGGTGGTCGCGTGACCATCTCCGTCGGCAACTCGACGACGGGCACCCATATGATCCGCGCCAAGAAGCTCATCGTCGCGGCCCCGCCCACCCTCGACAACCTGCGCTCGACGGGCCTGGACCTCGACACGACCGAGGCCGGCCTGTTCGGCAAGCTGTCCGCCGGCGTCTTTTATTCGCTCGTCGTCAAGGACACGGGCGTGGCCAAGGCCAACCTGCGCAACCGCCACCCGGCCAACCCGTACGGCTTCCCCGACCAGCCCTTCATCTACTCGGTGATCCCGCTGCCCAAGACCAGCGGGCTCGCGCAGGTGCTTCTGGGGTCCGCCTCGCCCCTGACCGCGAGCCAGGTCGAGGCGCGCGTCGCCGCCGACATCGGCCGTCTGCCCGCGTCGCTCCGGGGTAACGCGACCTCCGTCCCGAAGGTGGTGACCATGGCTGCGCACACCTGGAACGTGATGGCGCCCGTGGCGGACATCAAGGCCGGCTTCTACGACAAGCTCGAGGGGCTGCAGGGGCTCAAGGATACGTGGTACATTGGCGCGGCGTGGGCTACGCAGAGCAGCACTACCATCTGGGAGGGGCTGGAACAGGAGTTCCTTCCTAAGCTTCTTGCTGCTTTG**TAG**ATGGAGGAGTGTGGACAACGAAGGCCTAGGAGCATCATGTCTGTATGCGCTTTTTCTCAATGTCGGAGTACTAATTACACGACTCTTGTGTGGTGGGCGCGCAACTCTACTCGCACCCGCGCCGCAATTGGACAGGTGTTCCGTAGATACGATGCCTTATTTAATTAACTTACATTT***CACATTCAACTCAAGAGACG***CACGTTTTATAGTTTCTATAAAGCATCAGGTCGAGTTCCGACCAGCACATTATCTACCTTGACTGCCTGCCTATCGTACAAAGTTCATCCCCCCATTCAC

**DpasF**

MNRLLASALLVGSAVVAPVSAACIKNATVTEVDVAIIGGGASGVYAAARLIDNNKTVVVLERNADRIGGQTETYYDPTTGTPVNVGVKVFSNTTVTTDFLKRFDFPMGTLNVGQTLATGTQYVDFATGKLIPNFTAVVPAVQAAAMQRYAAELAKYPQIKFGYNLGPQVPEDLVLPFGKFMEKHNLTGMAQTMFEFNSGYTPLLEIPALYILKYLDTYELQSLQSGSFIVAANGDSATLYRNAAKFLGERVVYGVSGMHIQRSSAAGGRVTISVGNSTTGTHMIRAKKLIVAAPPTLDNLRSTGLDLDTTEAGLFGKLSAGVFYSLVVKDTGVAKANLRNRHPANPYGFPDQPFIYSVIPLPKTSGLAQVLLGSASPLTASQVEARVAADIGRLPASLRGNATSVPKVVTMAAHTWNVMAPVADIKAGFYDKLEGLQGLKDTWYIGAAWATQSSTTIWEGLEQEFLPKLLAAL
