# Supplementary material for: Synthetic biology based construction of biological activity-related library of fungal decalin-containing diterpenoid pyrones
Source: Nat Commun. 2020 Apr 14;11:1830. doi: 10.1038/s41467-020-15664-4 (PMC7156458; doi:10.1038/s41467-020-15664-4)
Supplement: Supplementary file 2 — Peer Review [file 41467_2020_15664_MOESM2_ESM.pdf]

Editorial Note: This manuscript has been previously reviewed at another journal that is not operating a transparent peer review scheme. This document only contains reviewer comments and rebuttal letters for versions considered at *Nature Communications.*

Reviewers' comments:

Reviewer #1 (Remarks to the Author):

Natural products are important sources for drug discovery, but the traditional approaches are facing with an increasingly limited access to unforeseeably structured natural products. Metabolic engineering and synthetic biology methodologies are capable of creating new compounds if the design is creative enough. The manuscript has reported the advantage of synthetic biology based on bioinformatics and metabolic engineering coupled with heterologous biosynthesis in Aspergillus oryzae NSAR1 for construction of the compound library of decalin-containing diterpenoid pyrones (DDP). Overall, 22 DDPs were heterologously biosynthesized, including 15 new analogues. Using a couple of bioassay models, the bioactivity of the DDP analogues was evaluated. Generally speaking, this work is a valuable study using biological and chemical strategies to obtain a collection of unnatural DDP analogs, some of which are bioactive. The overall novelty of this manuscript in chemistry and biology seems to be close to the requirement of Nature Communications although some designs are routine and a portion of results is generally expectable. Listed below is a selection of specific comments on the paper:
(1) The authors reconstructed the pathway of the core intermediate 4 by heterologous expression of the dpasABCDE gene cluster in Aspergillus oryzae NSAR1 (in Fig.3). Bioinformatic analysis signified that four more gene (dpfg, dpmp, dpch, and dpma) clusters could express 4, too. This reviewer is therefore quite curious about why the dpas ABCDE cluster is selected for the biosynthesis of 4. Did authors compare the 4 productivity of these 4-producing gene clusters?
(2) The titers of intermediate 4 is (87 mg/L), the authors reconstituted the pathway of AO-dpasABCDE-dpmaF provided subglutinols A (5, 89 mg/L) and subglutinols B (6, 6 mg/L). Theoretically, the 4 is the precursor for the 5 and 6, it seems like that the reconstituted pathway could not provide sufficient precursor 4 for biosynthesis of 5 and 6.
(3) Would you like to explain why the Flavin adenine dinucleotide (FAD)-dependent epoxidase (FMO) dpasF and dpchF have different stereoselectivity? dpasF could produce 5 (12S) and 6 (12R), while dpchF just expresses 10. Did the methyl transferase genes (MT) have the stereoselectivity?
(4) The bioassay section is wordy and preferred to be shortened.

Reviewer #2 (Remarks to the Author):

The authors have addressed most of my and the other reviewers' comments. The manuscript has been improved by shortening the introduction and conclusion as well as moving some less important bioassay to SI. I only have a few additional comments and suggestions:

-Please add an empty plasmid control to the Supplementary Information HPLC profiles, such as Figs S11 S12, S16, S20, S24, S28, S30, S34, S35, S39 and so on.
-It would be much clearer if the authors would be able to summarize each SAR of different assays by figures that represent structural variation leading to activity alteration.

Reviewer #3 (Remarks to the Author):

The authors describe a construction of a focused library of decalin-containing diterpenoid pyrones (DDPs) using synthetic biology approach. Based on the comparison of five biosynthetic gene clusters (BGCs) of fungal DDPs, the authors dissected and assembled the biosynthetic pathways of DDPs in a very rational and practical manner to produce DDPs with a diversity of substituents on the terpene and pyrone moieties. Using a fungal heterologous host, the authors collected sufficient amount of the DDPs and carried out multiple bioassay, which demonstrates usefulness of such focused libraries for bioactive compound screenings. This is a lot of work and carried out to a high standard. Data

presented fully support the conclusions that authors make, and the manuscript is substantially improved by the reviewers' comments. Thus, this will be well suited for Nature Communications.

Dear Reviewers

Thank you very much for your reviewing for our manuscript. I am returning here with responses to your comments. The points in the revised manuscript are as follows:

Reviewers' comments:

Reviewer #1 (Remarks to the Author):

Natural products are important sources for drug discovery, but the traditional approaches are facing with an increasingly limited access to unforeseeably structured natural products. Metabolic engineering and synthetic biology methodologies are capable of creating new compounds if the design is creative enough. The manuscript has reported the advantage of synthetic biology based on bioinformatics and metabolic engineering coupled with heterologous biosynthesis in *Aspergillus oryzae* NSAR1 for construction of the compound library of decalin-containing diterpenoid pyrones (DDP). Overall, 22 DDPs were heterologously biosynthesized, including 15 new analogues. Using a couple of bioassay models, the bioactivity of the DDP analogues was evaluated. Generally speaking, this work is a valuable study using biological and chemical strategies to obtain a collection of unnatural DDP analogs, some of which are bioactive. The overall novelty of this manuscript in chemistry and biology seems to be
close to the requirement of Nature Communications although some designs are routine and a portion of results is generally expectable. Listed below is a selection of specific comments on the paper:

(1) The authors reconstructed the pathway of the core intermediate 4 by heterologous expression of the dpasABCDE gene cluster in Aspergillus oryzae NSAR1 (in Fig.3). Bioinformatic analysis signified that four more gene (dpfg, dpmp, dpch, and dpma) clusters could express 4, too. This reviewer is therefore quite curious about why the dpas ABCDE cluster is selected for the biosynthesis of 4. Did authors compare the 4 productivity of these 4-producing gene clusters?

R1-A1:
Initially, we constructed *AO-dpasACD* and *AO-dpasABCDE* because the *Arthrinium sacchari* strain was our original sequenced fungous. The bioinformatic analysis of *dpxx* cluster clearly indicated that each *dpxxA* (NR-PKS), *dpxxC* (PT) and *dpxxD* (GGPPS) possessed the same function. The DDP pathways possibly branch at the epoxidation and terpene cyclase steps. Therefore, we checked the function of all the *dpxxBE* by introducing them into *AO-dpasACD*. All the transformants produced **4** in the similar productivities. *AO-dpasABCDE* enough produced common intermediate **4** (> 80 mg/L). Therefore, we used the transformants as platform to produce most DDPs. Because of experimental convenience, we used other **4** producing transformant for DPP production. However, each transformant was almost same.

(2) The titers of intermediate **4** is (87 mg/L), the authors reconstituted the pathway of AO-dpasABCDE-dpmaF provided subglutinols A (5, 89 mg/L) and subglutinols B (6, 6 mg/L). Theoretically,

the 4 is the precursor for the 5 and 6, it seems like that the reconstituted pathway could not provide sufficient precursor 4 for biosynthesis of 5 and 6.

R1-A2:
The titer of each compound in the corresponding transformant is slightly changed in every culture. We used the champion data in this article. Although it is difficult to explain, the productivity of metabolites in fungi is affected a various thing. Thus, we cannot thing it as organic reactions (eq). Of course, stability of metabolite under culture condition affects the titer.

(3) Would you like to explain why the Flavin adenine dinucleotide (FAD)-dependent epoxidase (FMO) dpasF and dpchF have different stereoselectivity? dpasF could produce 5 (12S) and 6 (12R), while dpchF just expresses 10. Did the methyl transferase genes (MT) have the stereoselectivity?
DpasF and DpchF possessed same function, though their selectivity was different.

R1-A3:
According to supplementary fig. 11, 20 and 28, DpasF and DpchF possessed the same function, though their selectivity is different. From the results, DpasF probably preferred 8*S* configuration, while DpchF preferred 8*R* configuration. They showed about 30% similarity in amino acid sequence, thereby they may show different selectivity. The stereoselectivity at C-12 may be depended on the stereochemistry at C-8. DpasF and DpchF installed an oxygen atom to construct enone **7**. Thus, DpasF and DpchF probably catalyzed hydroxylation at C-12, followed by generation of allyl cation with dehydration at C-12. From our results, 8*S* hydroxyl group can access to C-12 carbo cation from both sides, though 12*S* is major. However, 8*R* hydroxyl group can only access the cation from specific side to generate 12*S* configuration. Thus, we thought the selectivity of C-12 were depend on the conformation. However, these results in this study were obtained from in vivo experiments. It is difficult to normalize the expression level in mRNA and protein in the transformants. So, if we argue the selectivity, we should do in vitro experiment. However, the specific discussion is not purpose of this study. In addition, that experiment is not easy and it is next challenge.
The MT1s did not chose the C-8 stereochemistry.

(4) The bioassay section is wordy and preferred to be shortened.

R1-A4:
We shortened the bioassay section. It is difficult to summarize the section more. Other referees didn't comment in this point.

Reviewer #2 (Remarks to the Author):

The authors have addressed most of my and the other reviewers' comments. The manuscript has been

improved by shortening the introduction and conclusion as well as moving some less important bioassay to SI. I only have a few additional comments and suggestions:

(1)-Please add an empty plasmid control to the Supplementary Information HPLC profiles, such as Figs S11 S12, S16, S20, S24, S28, S30, S34, S35, S39 and so on.

R2-A1:

To construct the compound **4** producing transformants, we used *A. oyzae* NSARI strain as heterologous host. Therefore, we made the transformant with empty plasmid control. This result showed *A. oryzae* did not have the DDP biosynthetic machinery.

In this study, we used four plasmid vectors, pUARA2, pUADEA2, pUSCA2, pUPTRA2. As depicted supplementary figure 2, pUADEA2, pUSCA2, pUPTRA2 were constructed by only replacement of marker region of pUARA2. Thus, they share same sequence except for marker region.

When we constructed *AO-dpasABCDEF* (supplementry fig.11), we introduced pUSCA2-*dpasF* into *AO-dpasABCDE*. *AO-dpasABCDE* was constructed by using pUARA2 and pUADEA2, thereby it already possessed the common vector sequence, clearly indicating the conversion from **4** to **5** and **6** was catalyzed by additional modification enzyme not originated from vector. Therefore, we did not need to make *AO-dpasABCDE* with pUASCA2 (empty) for control and its host, *AO-dpasABCDE*, was used as control of this experiment. Other experiments (S12, S16, S20, S24, S28, S30, S34, S35, S39) were in the same situation as supplementary fig. 11.

(2) -It would be much clearer if the authors would be able to summarize each SAR of different assays by figures that represent structural variation leading to activity alteration. SI

R2-A2:

From the SI, the readers can understand the SAR tendency. From the results, we could not discuss the detailed SAR. We can only say the SAR roughly. Thus, we did not summary and emphasize each SAR of different assays by figure.

Reviewer #3 (Remarks to the Author):

The authors describe a construction of a focused library of decalin-containing diterpenoid pyrones (DDPs) using synthetic biology approach. Based on the comparison of five biosynthetic gene clusters (BGCs) of fungal DDPs, the authors dissected and assembled the biosynthetic pathways of DDPs in a very rational and practical manner to produce DDPs with a diversity of substituents on the terpene and pyrone moieties. Using a fungal heterologous host, the authors collected sufficient amount of the DDPs and carried out multiple bioassay, which demonstrates usefulness of such focused libraries for bioactive compound screenings. This is a lot of work and carried out to a high standard. Data presented fully support the conclusions that authors

make, and the manuscript is substantially improved by the reviewers' comments. Thus, this will be well suited for Nature Communications.

I believe the manuscript will be accepted for publication in Nature Communications.

Reviewers' comments:

Reviewer #1 (Remarks to the Author):

The authors have addressed the questions in a point-by-point manner. Most of the answers and explanations are reasonable and acceptable. The manuscript gained additional improvement as suggested by other reviewer(s). Collectively, this article can be recommended for its publication in Nature Communications.

Reviewer #2 (Remarks to the Author):

The paper has been improved a lot and in general I am fine with the changes. However, for one of my previous points, there might have been a misunderstanding:
In my initial revision request I asked for adding appropriate controls into the SI HPLC chromatograms. It is clear how the authors constructed the expression plasmids as described in the SI, but it is important for the authors to add additional information for the chromatograms. For example in Fig.12, what the authors have to clarify are the other (not yet annotated) peaks. In the current form it is not clear to me if the signals in (i) and (iii) are coming from the extra plasmids or the chassis strain rather than being unidentified pathway-related products. I would consider the peak around 11.1 min next to compound 7 is an analog or shunt product if there are no proper controls (empty plasmids ensuring the same selection markers and other plasmid parts) due to close retention time.

Reviewer's comment:

The paper has been improved a lot and in general I am fine with the changes. However, for one of my previous points, there might have been a misunderstanding:
In my initial revision request I asked for adding appropriate controls into the SI HPLC chromatograms. It is clear how the authors constructed the expression plasmids as described in the SI, but it is important for the authors to add additional information for the chromatograms. For example in Fig.12, what the authors have to clarify are the other (not yet annotated) peaks. In the current form it is not clear to me if the signals in (i) and (iii) are coming from the extra plasmids or the chassis strain rather than being unidentified pathway-related products. I would consider the peak around 11.1 min next to compound 7 is an analog or shunt product if there are no proper controls (empty plasmids ensuring the same selection markers and other plasmid parts) due to close retention time.

Ansewer.

I understood the reviewer's comment. We added HPLC chromatograms of AO-*asdpA*, which produced dimethyl pyrone **1**, in supplementary Fig. 12. Because non-assigned peaks, which were observed in Suppmementary Fig. 12 and the other HPLC chromatograms, were contained in the chromatogram AO-*asdpA*. AO-*asdpA* possessed only NR-PKS, thereby the peaks appeared in the chromatogram are not DDP analogs but derivative of dimethyl pyrone **1**. We also added HPLC chromatogram of *A. oryzae* with vacant plasmid vector in supplementary Fig. 12 to support our results. In other supplementary figures, we added the comments "Non-marked peaks were not DDPs. We didn't characterize them, but they may be derived from polyketide **1**, see Supplementary Fig. 12 -(ii)." in each figure legend to help the readers to understand our results.

REVIEWERS' COMMENTS:

Reviewer #2 (Remarks to the Author):

Thanks for adding the changes. Publish as is.
